# The nuclear export inhibitor aminoratjadone is a potent effector in extracellular-targeted drug conjugates†
†Electronic supplementary information (ESI) available. See DOI: 10.1039/c8sc05542d


**DOI:** 10.1039/c8sc05542d

**Published:** 2019-04-15

**Authors:** Philipp Klahn, Verena Fetz, Antje Ritter, Wera Collisi, Bettina Hinkelmann, Tatjana Arnold, Werner Tegge, Katharina Rox, Stephan Hüttel, Kathrin I. Mohr, Joachim Wink, Marc Stadler, Josef Wissing, Lothar Jänsch, Mark Brönstrup

**Affiliations:** a Department of Chemical Biology , Helmholtz Centre for Infection Research , Inhoffenstrasse 7 , 38124 Braunschweig , Germany . Email: mark.broenstrup@helmholtz-hzi.de; b Department of Microbial Drugs , Helmholtz Centre for Infection Research , Inhoffenstrasse 7 , 38124 Braunschweig , Germany; c Institute of Organic Chemistry , Technische Universität Braunschweig , Hagenring 30 , 38106 Braunschweig , Germany . Email: p.klahn@tu-braunschweig.de; d Department of Structure and Function of Proteins , Research Group Cellular Proteomic , Helmholtz Centre for Infection Research , Inhoffenstrasse 7 , 38124 Braunschweig , Germany; e Biomolecular Drug Research Center (BMWZ) , Schneiderberg 38 , 30167 Hannover , Germany; f German Centre of Infection Research (DZIF) , Partner Site Hannover-Braunschweig , Germany

## Abstract

The concept of targeted drug conjugates has been successfully translated to clinical practice in oncology. Whereas the majority of cytotoxic effectors in drug conjugates are directed against either DNA or tubulin, our study aimed to validate nuclear export inhibition as a novel effector principle in drug conjugates. For this purpose, a semisynthetic route starting from the natural product ratjadone A, a potent nuclear export inhibitor, has been developed. The biological evaluation of ratjadones functionalized at the 16-position revealed that oxo- and amino-analogues had very high potencies against cancer cell lines (*e.g.* 16*R*-aminoratjadone **16** with IC_50_ = 260 pM against MCF-7 cells, or 19-oxoratjadone **14** with IC_50_ = 100 pM against A-549 cells). Mechanistically, the conjugates retained a nuclear export inhibitory activity through binding CRM1. To demonstrate a proof-of-principle for cellular targeting, folate- and luteinizing hormone releasing hormone (LHRH)-based carrier molecules were synthesized and coupled to aminoratjadones as well as fluorescein for cellular efficacy and imaging studies, respectively. The Trojan–Horse conjugates selectively addressed receptor-positive cell lines and were highly potent inhibitors of their proliferation. For example, the folate conjugate **FA-7-Val-Cit-*p*ABA-16*R*-aminoratjadone** had an IC_50_ of 34.3 nM, and the LHRH conjugate **d-Orn-Gose-Val-Cit-*p*ABA-16*R*-aminoratjadone** had an IC_50_ of 12.8 nM. The results demonstrate that nuclear export inhibition is a promising mode-of-action for extracellular-targeted drug conjugate payloads.

## Abstract 2

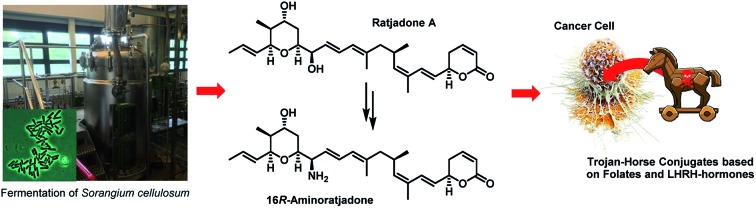
Ratjadone derivatives have been successfully introduced as suitable payloads with new mode of action for targeted drug conjugates.

## Introduction

Extracellular-targeted drug conjugates (EDCs) have recently evolved into an innovative, clinically proven molecular format in tumor therapies.^1^–^11^ The linkage of potent cytotoxins to appropriate carrier molecules, ligands with a high affinity for a cancer-specific biomarker overexpressed on the surface of malignant cells, enables the selective delivery of the cytotoxins to the cancer cells. After endocytosis-mediated uptake of such conjugates, the cytotoxins drive the cell into apoptosis. Compared to conventional chemotherapeutics, EDCs exhibit a broadened therapeutic window, since the exposure of healthy cells to the highly potent cytotoxins is significantly reduced. Most advances have been achieved so far utilizing antibodies as carrier molecules, as three antibody–drug conjugates (ADCs) have obtained marketing authorization, *i.e.* Adcetris® (2011), Kadcyla® (2011), and Besponsa® (2017), and about 60 further ADC candidates are in clinical trials.^9^ In addition, several other types of biomolecular carriers like probodies^12^ or anticalins,^13^–^15^ and small molecule carriers like vitamins^16^ such as folic acid,^17^–^20^ peptide hormone derivatives such as octreotide^21^–^24^ and LHRH^25^–^29^ and integrin binding RGD- and isoDGR peptides^30^ are under preclinical and clinical investigation as part of EDCs. Folic acid (vitamin B_9_) is a crucial growth factor for all cell types,^19^,^31^ because it is a cofactor in C1-transfer reactions especially in the synthesis of DNA and RNA bases.^31^ Several cancer cell types overexpress specific receptors (mainly folate receptor-α, FRα) that have a high folate affinity (<1 nM) and mediate its uptake *via* endocytosis.^31^ Folate–fluorophore conjugates have already been successfully applied for diagnostic cancer imaging^31^,^32^ and for intraoperative fluorescence imaging during tumor surgery.^31^,^33^,^34^ Furthermore, folate–drug conjugates have been demonstrated to selectively deliver cytotoxins to cancer cells.^17^,^31^

**Fig. 1:**
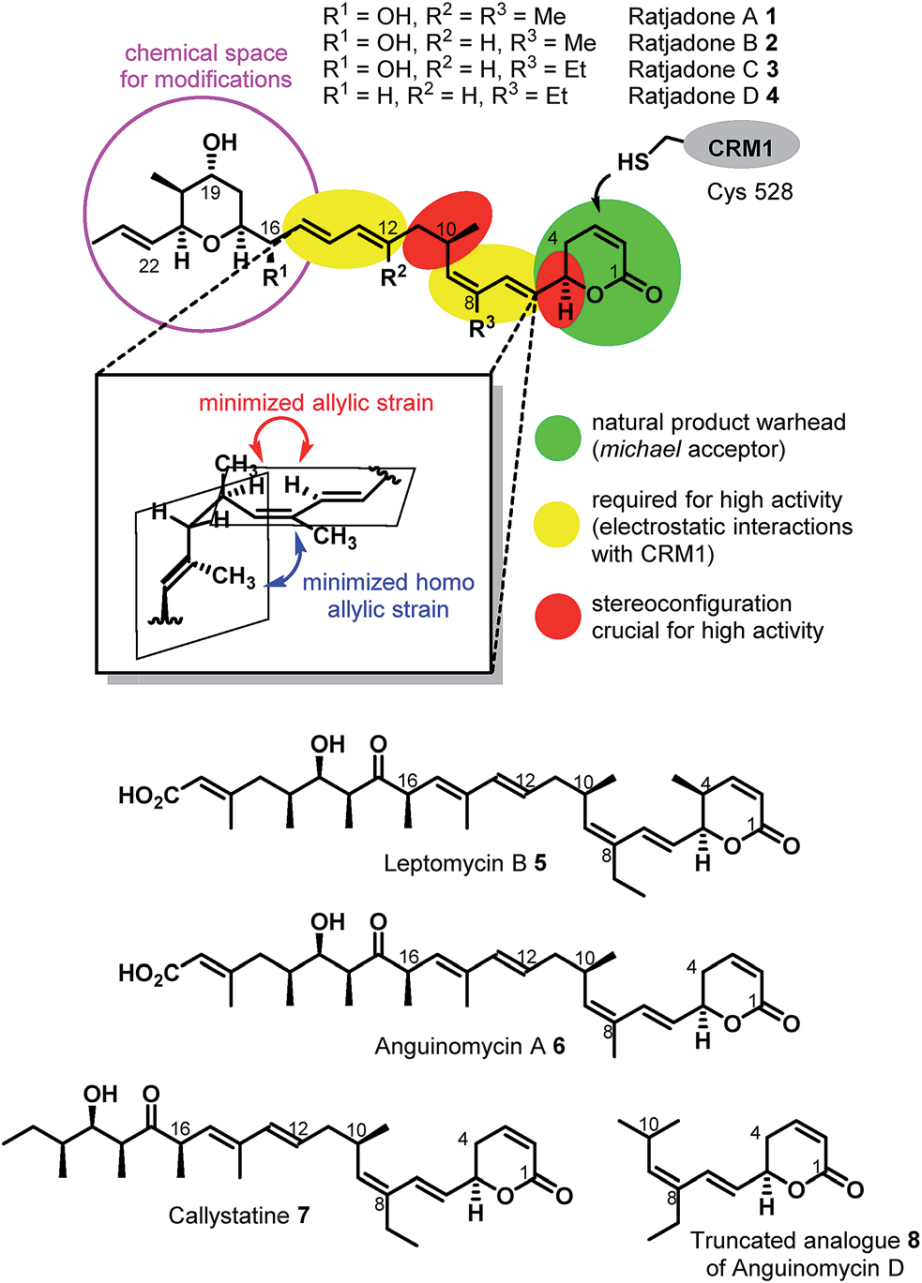
Structures and SAR of ratjadones A–D and related CRM1 inhibitors.

Remarkably, the cytotoxic payloads in approved ADCs as well as in most preclinical and clinical EDCs are all derived from natural products. The far majority of them falls into only two modes of action categories: (1) antimitotic, microtubule binding agents (*e.g.* tubulysines, auristatins, maytansinoids, taxols, epothilones, vincristines *etc.*),^35^–^41^ (2) agents causing DNA damage by alkylation or intercalation (calicheamycins, duocarmycins, PBD's, or anthracyclines).^36^,^37^,^39^,^42^ In addition, few other mechanisms, including RNA polymerase inhibition by α-aminitin^43^ and spliceosome targeting by *e.g.* thailanstatin A,^44^ have been studied as EDCs.^37^ Considering the high incidence of cancer relapse or development of metastasis after seemingly successful chemotherapeutic treatment,^45^–^48^ and the ability of tumors to become multi-drug resistant,^49^,^50^ there is a strong need to investigate new cytotoxic payloads for EDCs with different modes of action to enable more stratified tumor treatments in the future. In this context we got interested in (+)-ratjadone A (**1**) and related analogs (Fig. 1), polyketidic natural products produced by the myxobacterium *Sorangium cellulosum*.^51^,^52^ Ratjadones, structurally related to leptomycins,^53^ show potent, sub-nanomolar antiproliferative activity against several cancer cell lines including multi-drug-resistant ones.^54^ The antiproliferative activity of ratjadones is based on the inhibition of CRM1,^54^,^55^ a protein receptor involved in the nuclear export of substrates larger than 40 kDa from the nucleus into the cytosol of mammalian cells.^56^,^57^ Since the inactivation of CRM1 leads to inhibition of central cellular functions,^58^ reduction of angiogenesis and metastatic ability of tumors,^59^ sensitization of the cancer cell to other drugs^59^–^61^ and finally to cell death, the inhibition of nuclear export mediated by CRM1 is regarded as an attractive anticancer mechanism. However, early clinical trials with the CRM1-inhibitor leptomycin B as a standalone drug failed due to its high systemic toxicity.^62^

For these reasons, we aimed to establish extracellular-targeted conjugates based on highly potent CRM1 inhibitors in order to explore nuclear export as an anticancer strategy. We describe a semisynthetic approach to novel ratjadone A analogues bearing amino moieties for an easy and selective conjugation and their biological evaluation, thereby establishing structure–activity relationships. Furthermore, we report the synthesis of novel folate- and luteinizing hormone releasing hormone (LHRH)-based carrier molecules, their conjugation with aminoratjadone-payloads and their biological evaluation, which led to the discovery of novel EDCs with an antiproliferative activity in the low double-digit nanomolar range.

## Result and discussion

### Derivatization of (+)-ratjadone A

**Fig. 2:**
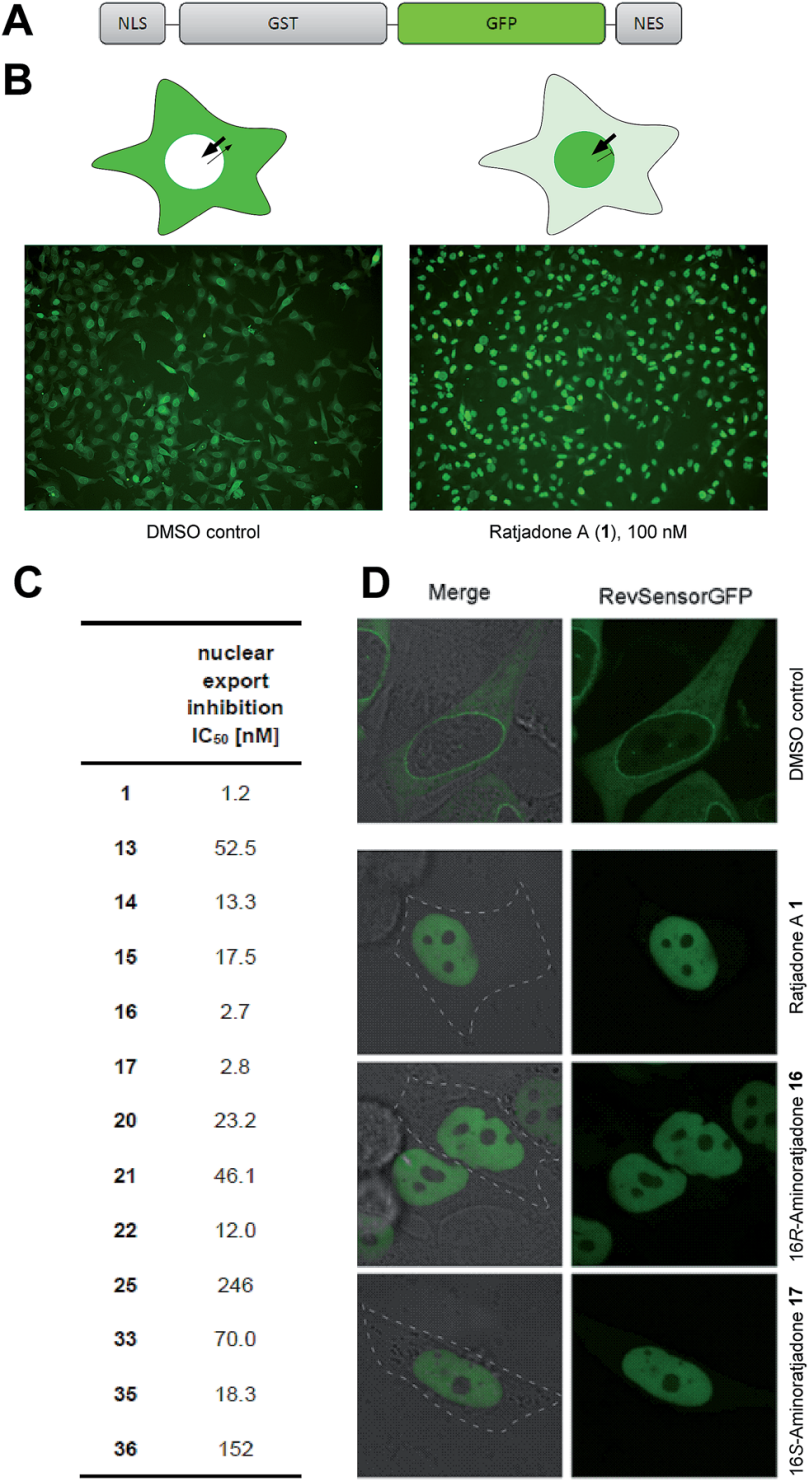
Inhibition of nuclear export by ratjadone A derivatives in recombinant HeLa cells. (A) Functional domains in fluorescent translocation biosensor system. (B) Cellular distribution of biosensor in untreated (left) and ratjadone A treated HeLa cells (right). (C) IC_50_ values of nuclear export inhibitory activity. (D) Fluorescence microscopy pictures of biosensor expressing HeLa cells treated with **1**, **16**, and **17**.

The first total synthesis of (+)-ratjadone A (**1**) was published in 2001 by Kalesse and coworkers^63^ and utilized for the synthesis of several derivatives.^64^–^66^ However, in order to assure an economic, scalable access to gram amounts of the natural product as required for the development of EDCs, we established a reliable fermentation protocol with an optimized strain of the myxobacterial producer *Sorangium cellulosum* (Soce1047). Fermentation titers of up to 32 mg L^–1^ of **1** were obtained after 3–7 days, and an optimized downstream process led to an isolated total amount of 3.646 g of **1** within a few weeks from 150 L of culture (see ESI†). To retain the potent antiproliferative activity of **1** during semisynthetic derivatization, several parts of the molecule need to remain unchanged. Its warhead is a α,β-unsaturated δ-lactone (Fig. 2), which serves as a Michael acceptor and covalently binds to the cysteine 528 of the target protein CRM1, resulting in lacton hydrolysis and irreversible inhibition of CRM1.^55^ Furthermore, structure–activity relationship studies of simplified analogues and stereoisomers of **1** revealed that the absolute stereo configuration at C5 and C10 as well as the two diene moieties are crucial for a high antiproliferative activity, since the minimization of the allylic and homo allylic strain defines the overall shape of the natural product.^64^ These structural features are shared with other potent CRM1 inhibitors such as leptomycin B (**5**) (Fig. 1), anguinomycin A (**6**) and callistatin A (**7**), and their importance has been rationalized by co-crystal structures.^53^,^55^ In 2010 Gademann and coworkers demonstrated that the truncated analogue **8** of anguinomycin D (Fig. 1), missing one of the diene moieties, inhibits nuclear export at concentration of 25 nM.^67^,^68^ However, sub-nanomolar activities require both diene moieties.

**Scheme 1:**
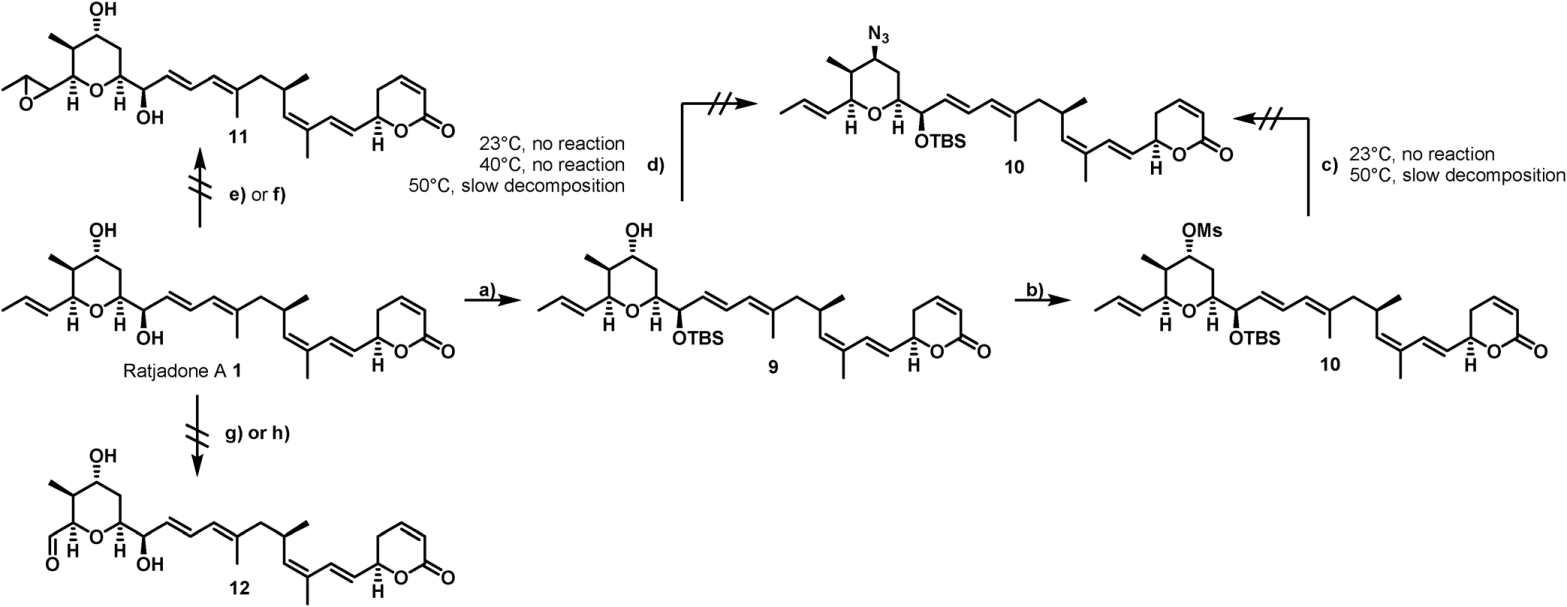
Unsuccessful strategies for the selective derivatization of ratjadone A. Reagents and conditions: (a) TBSOTf (2.3 equiv.), 2,6-lutidine (2.6 equiv.), (CH_2_Cl_2_), –100 °C, 3 h, 62% brsm; (b) MsCI (3.3 equiv.), Py (4.0 equiv.), (CH_2_Cl_2_), 23 °C, 3.5 h, 98%; (c) NaN_3_ (3.0 equiv.), (DMF); (d) DIAD (1.1 equiv.), PPh_3_ (1.15 equiv.), DPPA (1.0 equiv.), (THF); (e) *m*CPBA (1.0 equiv., NaHCO_3_ (1.05 equiv.), (CH_2_Cl_2_ : H_2_O/3 : 1), 0 °C or –10 °C; (f) DMDO (1.1 equiv.), (CH_2_Cl_2_), –30 °C; (g) O_3_, (CH_2_C1_2_ : MeOH/9 : 1), –78 °C, PPh_3_ (xs); (h) OsO_4_ (0.01 equiv.), NMO (1.0 equiv.) or PNO (1.0 equiv.), (CH_2_Cl_2_), 0 °C or –10 °C.

Our derivatization approaches therefore focused on modifications in the pyrane core and the adjacent allylic hydroxy group. Because an acylation of the two hydroxy groups proceeded with low yields and hardly showed any selectivity,^54^ we planned to introduce an amino handle to the molecule to enable selective conjugations with carrier molecules. In a first derivatization strategy (Scheme 1), a selective silylation of the C16 hydroxy function in the presence of 2.3 equivalents of TBSOTf at –100 °C for 3 h gave compound **9** in 62% yield based on recovered starting material (brsm) together with some double TBS-protected material. We assume that the limited yield was a consequence of the low nucleophlic reactivity of the hydroxyl groups.

As TBSCl was completely unreactive, the relatively strong Lewis acid TBSOTf had to be applied for the silylation, which led at least to some decomposition of the natural product. Compound **9** could be further converted into the corresponding C19 O-mesylate **10** in 98% yield.

However, the envisaged nucleophilic substitution with sodium azide did not proceed at ambient temperatures and led to slow decomposition of the starting material without any formation of substitution product at higher temperatures. An alternative Mitsunobu reaction in the presence of diphenylphosphoryl azide showed no conversion at reaction temperatures up to 40 °C, while higher reaction temperature led to slow decomposition of the starting material.

**Scheme 2:**
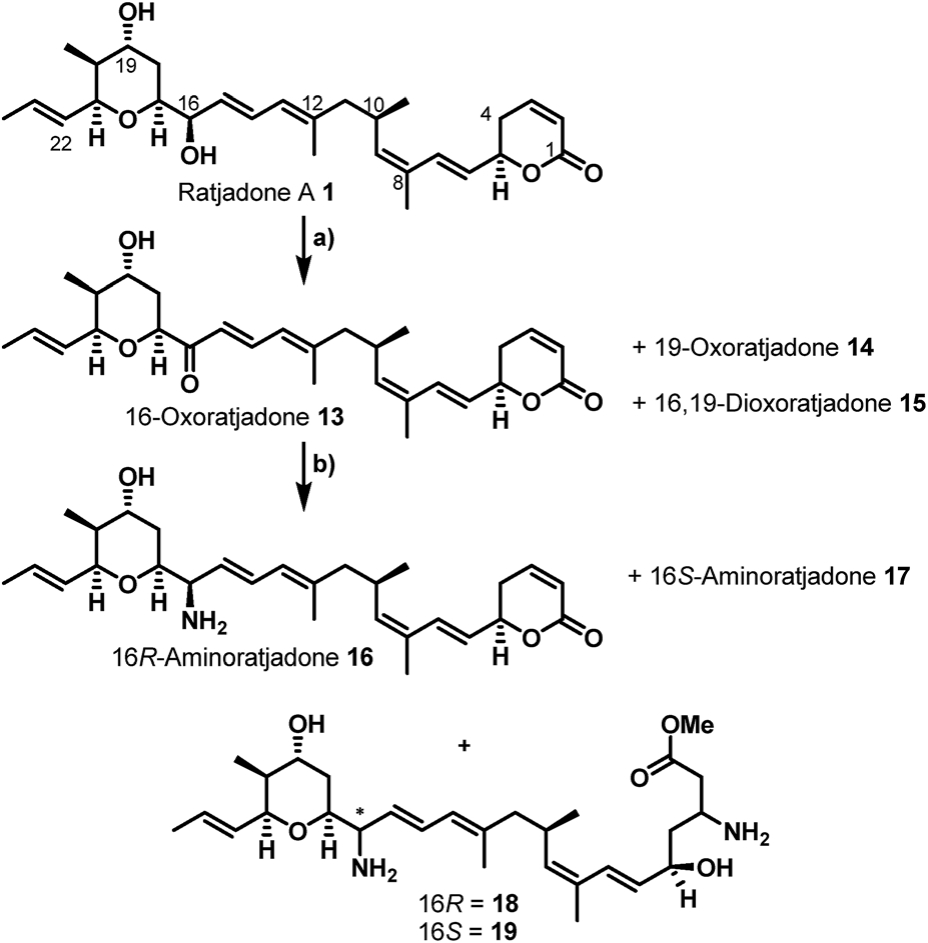
Semi-synthesis of C16-aminoratjadones from (+)-ratjadone A. Reagents and conditions: (a) IBX (1.1 equiv.), (DMSO, 0.075 M), 23 °C. Addition of 16 h + 23 °C. 24 h, 75% brms **13**, 8% brms **14**, 15% brms **15**. (b) (NH_4_)OAc (10 equiv.), NaCNBH_3_ (2.0 equiv.), (MeOH), 23 °C, 13 h + 40 °C, 2 h, 34% brsm **16**, 21% brms **17**, 8% brsm **18**, 2% brsm **19**.

**Scheme 3:**
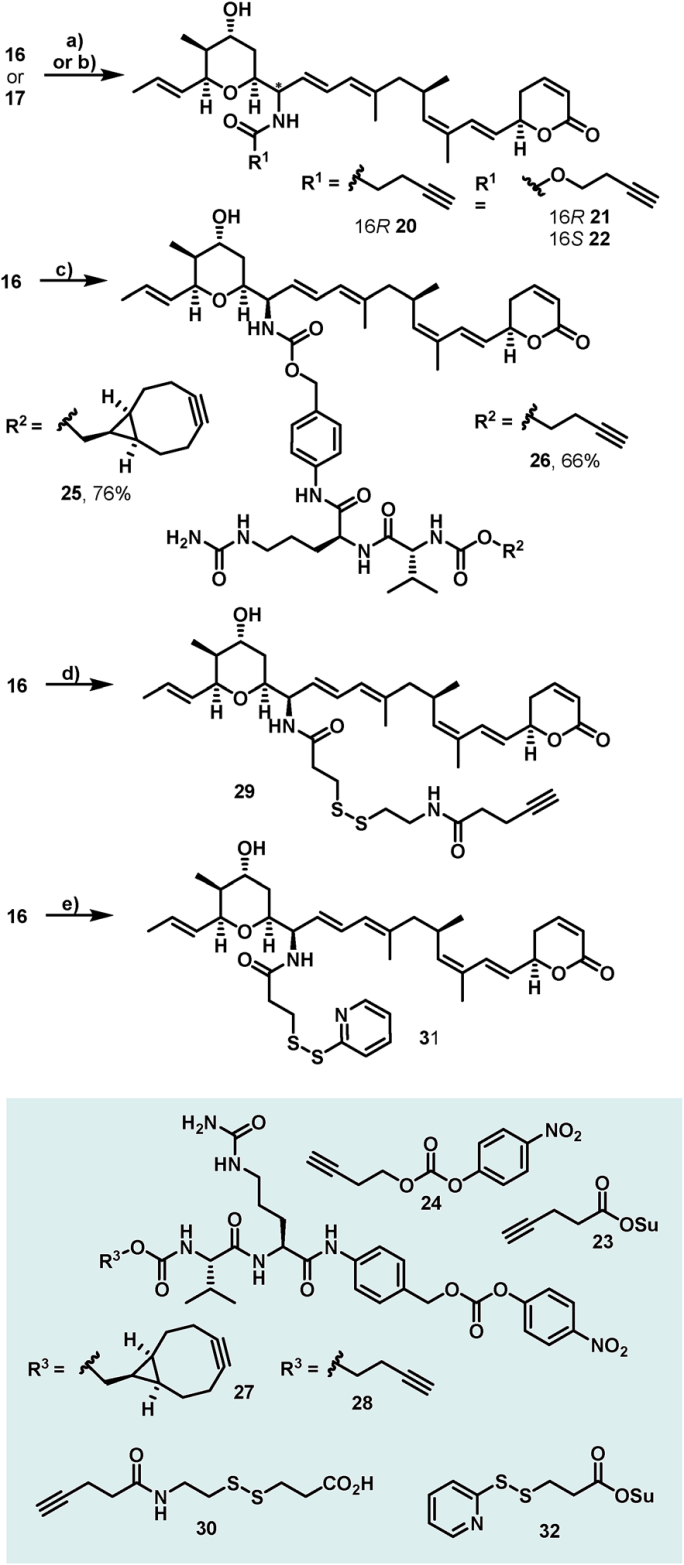
Synthesis of C16-aminoratjadones bearing short terminal alkyne moieties and enzymatically cleavable Val-Cit-*p*ABA or disulphide linkers. Reagents and conditions: (a) **22** (1.1 equiv.), NMM (3.0 equiv.), (CH_2_C1_2_). 23 °C, 1.5 h, 65% for **20**; (b) **24** (1.1 equiv.), NMM (3.0 equiv.), (CH_2_C1_2_), 23 °C, 20 h; 90% for **21** and 69% for **22**; (c) **27** or **28** (1.1 equiv.), NMM (6.0 equiv.), (DMF), 23 °C. 26 h, 76% for **25** and 66% for **26**; (d) **30** (1.0 equiv.), TSTU (1.0 equiv.), NMM (5.0 equiv.), (DMF), 23 °C, 15 h, 65% for **29**; (e) **32** (1.0 equiv.), (MeCN : PBS (pH = 7.45)/1 : 2), 0 °C to 23 °C, 2.5 h, 70% for **31**.

Additionally, attempts to selectively oxidize with ozone, dihydroxylate or epoxidize the double bound between C22 and C23 of **1** were unsuccessful and led to non-selective reactions or decomposition of the natural compound. In a second derivatization strategy (Scheme 2) we envisaged a selective oxidation of the more reactive allylic alcohol at C16, followed by a reductive amination. While no oxidation of **1** was observed in the presence of MnO_2_ (the compound was quantitatively reisolated after 24 h at 50 °C), the use of PCC in methylene dichloride led to fast decomposition. A first success was achieved using Dess–Martin periodinane (DMP) in methylene chloride, yielding the desired 16-oxoratjadone **13** (Scheme 2) in 63% brsm. Improved yields were obtained by dropping a solution of 1.1 equiv. of freshly prepared iodoxybenzoic acid (IBX)^69^ in dimethyl sulfoxide into a diluted solution of **1** over a period of 16 h, giving the 16-oxoratjadone **13** together with the 19-oxoratjadone **14** and the 16,19-dioxoratjadone **15** in 75%, 15% and 8% yields (all brsm) after chromatographic separation, respectively. Higher amounts of IBX or faster addition of the oxidant led to predominant formation of diketone **15**. Finally, a reductive amination of **13** in the presence of an excess of ammonium acetate and sodium cyanoborohydride at 23 °C gave 16*R*-aminoratjadone **16** and 16*S*-aminoratjadone **17** in 32% brms and 21% brsm, respectively, together with small amounts of the compounds **18** and **19**, in which the dihydropyran-2-one warhead was destroyed. All displayed a significantly increased water solubility compared to **1**. Milder reaction conditions at 0 °C resulted in significantly longer reactions times, uncomplete conversion and predominant formation of **18** and **19** (Scheme 2), whereas the use of sodium trimethoxy borohydride led to no conversion at all. The absolute stereo configuration at the C16 position was determined by Mosher's method based on the corresponding Mosher amides of **16** and **17**.^70^–^72^ We then functionalized **16** or **17** with non-cleavable as well as enzymatically cleavable linker moieties at the C16 amino function. The derivatives **20**, **21** and **22** (Scheme 3), bearing amide- and carbamate-linked short terminal alkyne moieties as attachment points for appropriate carrier molecules, could be generated by reaction with the *N*-hydroxysuccinimide ester **23** or the *p*-nitrophenyl carbonate **24**. The aminoratjadone derivatives **25** and **26** (Scheme 3) carried a valine-citrulline-*p*ABA unit that can be cleaved intracellularly by the lysosomal enzyme cathepsin B.^73^–^76^ They were obtained by reaction of **16** with *p*-nitrophenyl carbonates **27** or **28**, respectively (for synthesis of **27** and **28** see ESI†).

**Scheme 4:**
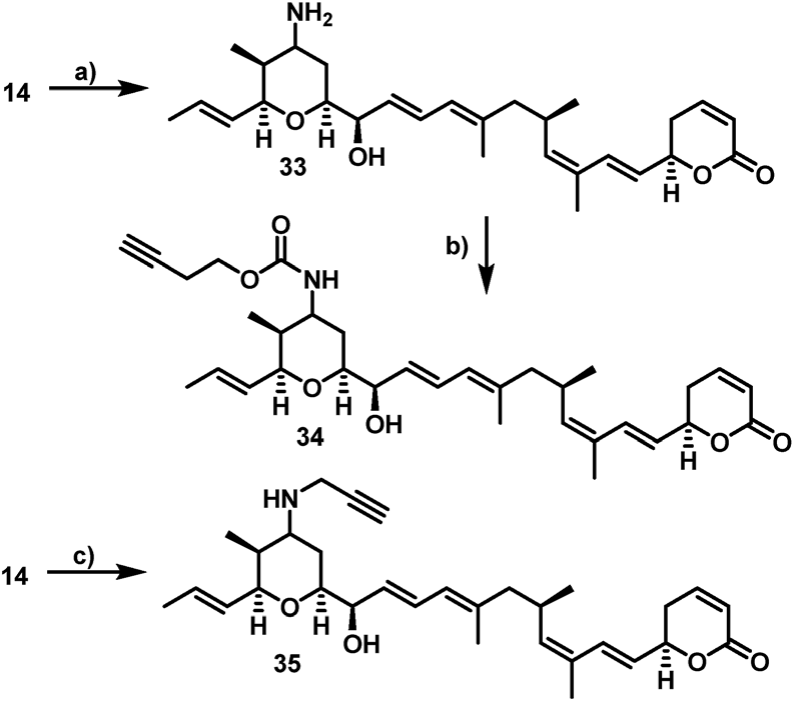
Semi-synthesis of alkyne-linked C16-aminoratjadones. Reagents and conditions: (a) NH_4_OAc (2.0 equiv.), NaBH_3_CN (2.0 equiv.), (MeOH), 23 °C, 4h, 68% of **33** as 2 : 1-mixture of diastereomers; (b) **24** (1.1 equiv.), NMM (6.0 equiv.), (CH_2_Cl_2_), 23 °C, 20 h, 62% of **34** as a 2 : 1 mixture of diastereomers; (c) propargylamine (2.0 equiv.), NaBH_3_CN (2.0 equiv.), (MeOH), 23 °C, 4 h, 99% of **35** as a 2 : 1-mixture of diastereomers.

Furthermore, **29** and **30**, bearing disulfide bridges that are supposed to be reductively cleaved in the cell, were obtained from **16** by amide formation in the presence of **30** (for synthesis of **30** see ESI†) and stoichiometric amounts of TSTU, or by conversion with the commercially available *N*-succinimidyl 3-(2-pyridyldithio)-propionate **32**. In parallel, we used the byproduct 19-oxoratjadone **14** for the synthesis of further C19-aminoratjadone derivatives (Scheme 4).

The direct reductive amination of **14** in the presence of ammonium acetate and sodium cyanoborohydride gave 19-aminoratjadone **33** as an inseparable 2 : 1-mixture of both diastereomers, which could be further converted to **34** in the presence of *p*-nitrophenyl carbonate **24**, or to **35** in the presence of propargylamine.

### Biological evaluation of novel aminoratjadones and derivatives

All ratjadone A derivatives were biologically evaluated for their antiproliferative activity against a panel of cancer cell lines representing human cervix, ovarian, breast and lung carcinomas. While the 16-oxoratjadone **13** and the 16,19-dioxoratjadone **15** showed slightly decreased antiproliferative activity compared to **1**, which could be rationalized by the electronic influence of the 16-oxo function to the second diene system, 19-oxoratjadone **14** was equally active. Fortunately, also 16*R*-aminoratjadone **16** displayed strong antiproliferative activity, equipotent with the natural template. However, the activity of 16*S*-analog **17** was fourfold decreased compared to **16** with the 16*R*-configuration. A significant drop in activity was observed for **18** and **19** bearing a ring-opened warhead, although the compounds are still highly active in absolute terms, with IC_50_ values of 115.6 nM and 165.4 nM, respectively. We speculate that the derivatives might still serve as non-covalent inhibitors of CRM1. Alternatively, an elimination of the β-amino group under assay conditions at C3 would lead to an α,β-unsaturated ester. Based on results for an α,δ-unsaturated aldehyde derivative of anguinomycin D reported by Gademann and coworkers,^67^ such a species is presumed to retain bioactivity. All observed trends in activity, such as the relative decreases for **13** and **14***vs.***1**, **17***vs.***16**, or the absolute levels of activity of **18** and *vs.***17** and **16**, were much more pronounced for the murine fibroblast cell line L929 compared to the tested human cell lines (Table 1).

**Table 1:** Antiproliferative activity of novel aminoratjadone derivatives against a panel of cancer cell lines^*a*^

	Antiproliferative activity IC_50_ [nM]				
	KB-3.1	A-549	SK-OV-3	MCF-7	L-929
**1**	0.46	0.15	0.24	0.11	0.68
**13**	2.57	1.76	0.24	0.22	12.6
**14**	0.36	0.10	0.16	0.48	1.15
**15**	3.98	9.72	1.59	2.87	24.3
**16**	0.39	0.31	0.61	0.26	4.61
**17**	1.59	1.31	0.81	0.36	28.4
**18**	115.6	—	—	—	3307
**19**	165.4	—	—	—	1323
**20**	1.05	0.58	0.44	0.40	—
**21**	0.47	1.25	0.45	0.27	4.50
**22**	1.23	2.45	1.20	0.62	—
**25**	25.1	39.5	34.7	20.2	—
**26**	9.20	25.1	13.6	12.5	—
**29**	1.86	0.86	1.09	0.17	—
**31**	8.80	30.6	24.6	26.0	—
**33**	6.67	4.39	1.40	2.10	—
**34**	7.23	5.22	4.22	3.10	—
**35**	1.23	1.12	2.63	1.31	—

^*a*^Cell line origin: KB-3.1: human cervix carcinoma, A-549: human lung carcinoma, SK-OV-3: human ovarian carcinoma, MCF: human breast carcinoma, L-929: murine fibroblast.

Short amide- or carbamate-linked terminal alkyne moieties at the C16 amino function as in **20**, **21** and **22** were also tolerated, with IC_50_ values in the sub-nanomolar or single-digit nanomolar range. However, longer linkers such as the cathepsin B-cleavable citrulline-valine-*p*ABA moiety (as in **25** and **26**) or disulfide-containing linkers (as in **29** and **31**) at the C16 position led to clear (up to 50-fold) drops in activity compared to **1**. Furthermore, all 19-aminoratjadone derivatives were less active compared to the 16-aminoratjadones, but showed still potent IC_50_ values in the single digit nanomolar range.

Next, we investigated the nuclear export inhibitory activity of selected novel ratjadone derivatives with a fluorescent translocation biosensor system.^77^–^79^ This cellular assay uses HeLa cells that express a recombinant fusion protein consisting of a nuclear localization signal (SV40-NLS), glutathione S-transferase (GST), green fluorescent protein (GFP) and a nuclear export signal (HIV1-RevNES) (Fig. 2A). Due to the two transport signals (NLS/NES), the biosensor is permanently shuttling between nucleus and cytoplasm but resides predominantly in the cytoplasm due to a comparatively stronger NES in cancer cells. Export inhibiting compounds induce a nuclear accumulation of the GFP-signal (Fig. 2B). For all tested derivatives a potent nuclear export inhibitory activity could be demonstrated.

Notably, the aminoratjadones **16** and **17** displayed IC_50_ values comparable to the natural template (Fig. 2C and D).

**Fig. 3:**
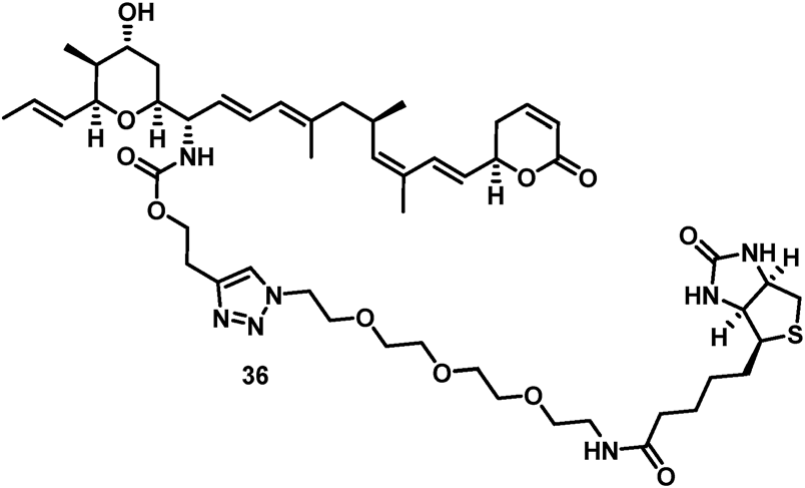
Structure of biotin-PEG_3_-16*S*-aminoratjadone conjugate **36**.

The biotin-PEG_3_-16*S*-aminoratjadone conjugate **36** (Fig. 3, for synthesis of **36** see ESI†) had a nuclear export activity of IC_50_ = 152 nM. To verify that this activity was indeed CRM1-mediated, HeLa cells were incubated with **36** at a concentration of 100 ng mL^–1^ for 5 h and lysed.

The cell lysate was incubated with streptavidin-coupled sepharose beads, and bound proteins were eluted, digested and identified by LC-MS/MS. The target CRM1 was clearly identified in this manner and strongly enriched compared to a control sample that was additionally incubated with an excess of **1**.

The findings demonstrate that conjugates functionalized at the 16 position of **1** indeed bound to CRM1 and retained a nuclear export activity. We therefore assume that the cellular mechanism of **1** was not altered upon derivatization.

### Synthesis and biological evaluation of folate-based carrier molecules and fluorescein conjugates

In order to probe whether ratjadones could serve as cytotoxic payloads for EDCs, they were conjugated to folate-based targeting moieties. In order to minimize uptake of the conjugates by passive diffusion, we incorporated polar amino acids as a hydrophilic spacer that were coupled to the γ-carboxylate of folate. For fast and simple bioorthogonal attachment to the effectors, we incorporated azide or sulfhydryl moieties to all carrier molecules.

**Scheme 5:**
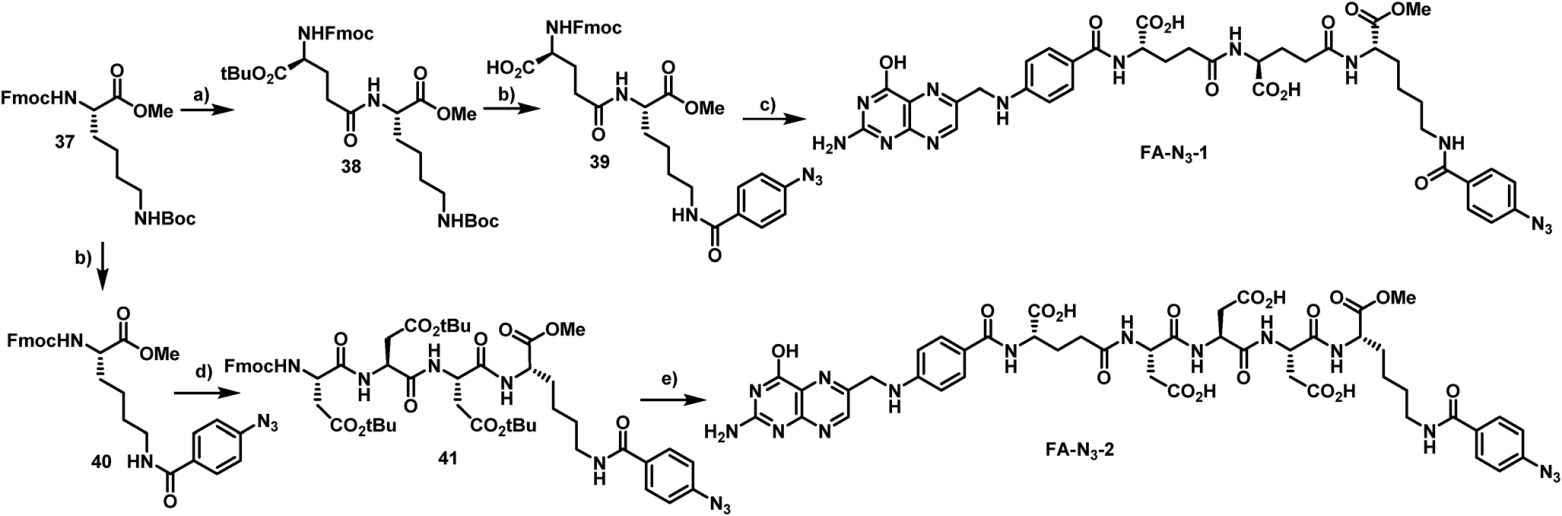
Synthesis of azido-folate carrier molecules in solution. Reagents and conditions: (a) (1) Pip (xs), (CH_2_Cl_2_), 23 °C, 3 h, (2) Fmoc-(Asp(O*t*Bu))_3_-OH (1.2 equiv.), EDCl HCl (1.1 equiv.), HOAt (1.1 equiv.), NMM (6.0 equiv.), (THF), 0 °C + 23 °C, 24 h, 58% of **38**; (b) (1) TFA (30 equiv.), (CH_2_C1_2_), 23 °C, 22 h, (2) *p*-azidobenzoic acid (1.1 equiv.), HATU (1.1 equiv.), HOAt (1.1 equiv.), NMM (10.0 equiv.), (DMF), 23 °C, 24 h, 88% of **39** and 85% of **40**; (c) (1) Et_2_NH (xs), (DMF), 23 °C, 20 h, (2) FA-γ-OSu (6.0 equiv.), NMM (6.0 equiv.), (DMSO), 23 °C, 14 h, 48% of **FA-N_3_-1**; (d) (1) Et_2_NH (xs), (CH_2_Cl_2_), 23 °C, 9 h, (2) Fmoc-(Asp(O*t*Bu))_3_-OH (1.2 equiv.). EDCI (1.2 equiv.), HOAt (1.2 equiv.), NMM (6.0 equiv.), (THF), 30 min preactivation at 23 °C, then 23 °C, 5 h, 75% of **41**; (e) (1) TFA (50 equiv.), (CH_2_Cl_2_), 23 °C, 20 h, (2) Et_2_NH (xs), (DMF), 23 °C, 20 h, (3) FA-γ-OSu (6.0 equiv.), NMM (6.0 equiv.), (DMSO), 23 °C, 16 h, 32% of **FA-N_3_-2**.

The first azide-containing folate carrier molecules (FA-N_3_) were generated by a solution phase synthesis (Scheme 5). Starting from Fmoc-Lys(Boc)-OMe (**37**), the Fmoc cleavage in presence of piperidine and subsequent EDCI-mediated peptide coupling with Fmoc-Glu-O*t*Bu led to formation of the dipeptide **38** in 58% yield. The simultaneous acidic cleavage of the *tert*-butyl ester and Boc group in the presence of TFA, followed by a HATU-mediated peptide coupling with *p*-azido benzoic acid, gave **39** in 88% yield.

Finally, the removal of the Fmoc group and subsequent conversion of the free amine with an excess of folic acid γ-*N*-hydroxysuccinimide ester led to the formation of **FA-N_3_-1**, which could be isolated in 48% yield. Starting from **37**, the Boc protective group was removed in the presence of TFA, and a HATU-mediated peptide coupling with *p*-azido benzoic acid yielded **40** in 85% yield. The removal of the Fmoc group and an EDCI-mediated peptide coupling with Fmoc-Asp(*t*Bu)-d-Asp(*t*Bu)-Asp(*t*Bu)-OH tripeptide (synthesized on solid phase) led to formation of the tetrapeptide **41**. The removal of *tert*-butyl esters and Fmoc and the subsequent conversion of the free amine with an excess of folic acid γ-*N*-hydroxysuccinimide ester gave **FA-N_3_-2** in 32% yield.

**Fig. 4:**
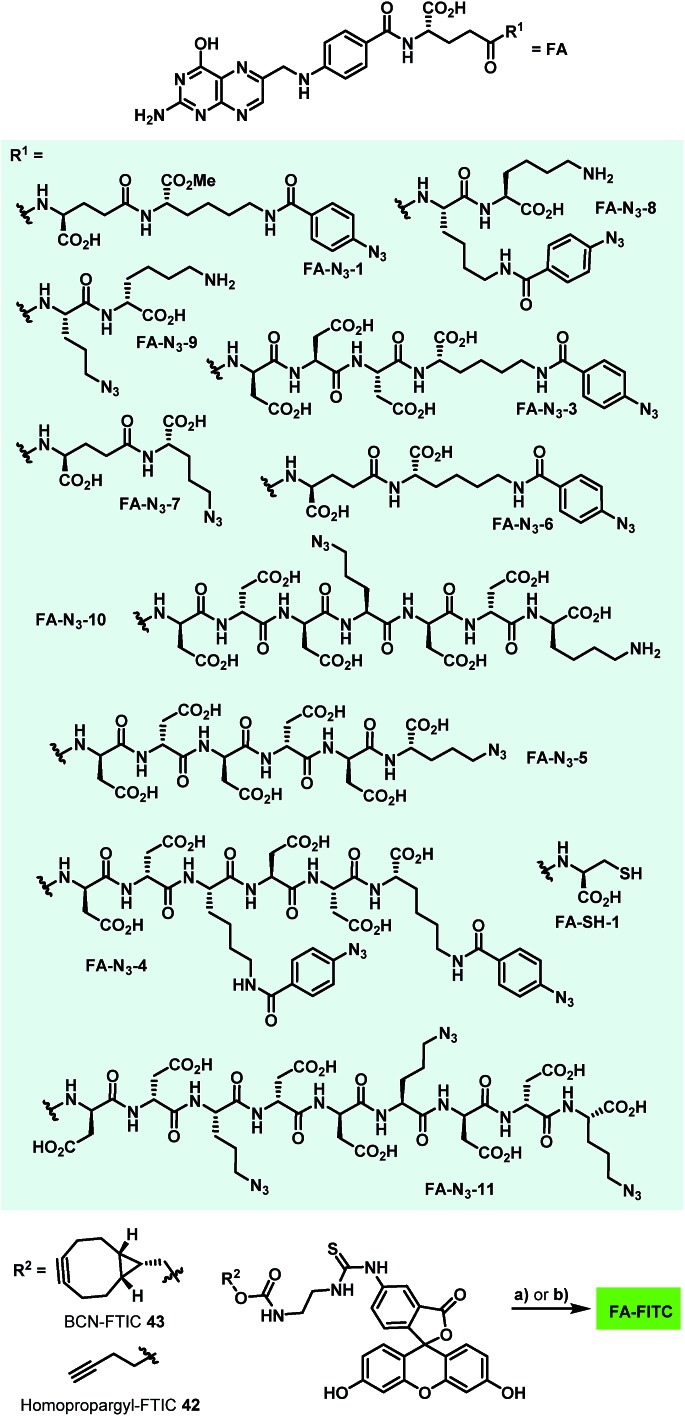
Folate-based carrier molecules and folate–fluorescein conjugates synthesized in this study. Reagents and conditions: (a) FA-N_3_ (1.1 equiv.), CuSO_4_ (0.05 equiv.), TBTA (0.1 equiv.), NaAsc (0.5 equiv.), DiPEA (6.0 equiv.), (DMSO : H_2_O :  ^*t*^BuOH/2 : 1 : 1), 23 °C, 2–24 h; (b) FA-N_3_ (1.1 equiv.), (DMSO), 23 °C, 4–20 h.

In order to avoid the methyl ester in the final folate carrier molecules and to provide a more convenient route to further folates, we developed a second route based on solid phase-supported peptide synthesis with 2-chlorotrityl resins. By applying standard HATU-mediated Fmoc peptide coupling chemistry and capping the resin-bound peptides with folic acid γ-*N*-hydroxysuccinimide ester, the fully assembled folate carrier molecules were obtained in yields ranging from 20–61% after cleavage from the resin, global removal of the protective groups and purification by preparative HPLC. Following this procedure, nine additional azide-containing folate carrier molecules were generated, including **FA-N_3_-4** and **FA-N_3_-11** (Fig. 4), that were designed for a double or triple loading with aminoratjadones, respectively.

Lysine residues were introduced in **FA-N_3_-8** and **FA-N_3_-9** to reduce charge, while increasing hydrophilicity. Additionally, we synthesized **FA-SH-1** as a literature-known example for a sulfhydryl-containing folate carrier molecule.^80^,^81^ We next synthesized fluorescein-labeled folate conjugates (FA-FITC) by copper-mediated or metal-free azide–alkyne click chemistry with either the terminal-alkyne bearing **48** or the BCN-containing **49** (Fig. 4 and ESI†).

**Fig. 5:**
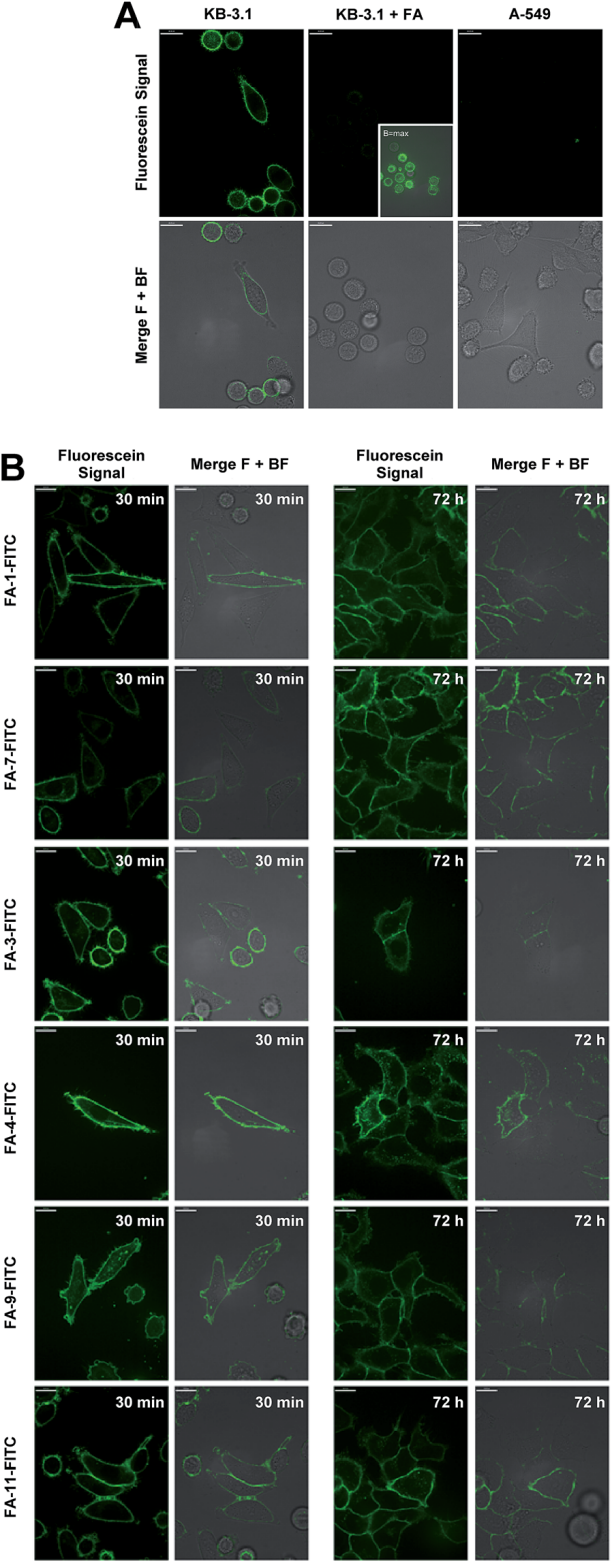
Confocal fluorescence microscopy. (A) Imaging of KB 3.1 and A-549 cells with folate–fluorescein conjugate FA-1-FITC (8.6 μM, 5 min, 37 °C). KB 3.1 cells were imaged in the presence (left) of absence (middle) of an excess of folic acid. (B) Imaging of KB 3.1 cells treated with 1 μM of six folate–fluorescein conjugates at two different time points (30 min and 72 h). All experiments were conducted in folate-free RPMI medium with 10% folate-containing FCS. Merge F + BF: merge fluorescence signal + bright field.

The conjugates were then utilized to verify the ability of the folate carrier molecules to selectively deliver the fluorophore to KB-3.1 cancer cell lines, which express the folate receptor-α on their cell surface under folate-limiting media conditions. As exemplified for the folate–fluorescein conjugate FA-1-FITC (the click product of **FA-N_3_-1** and 43, for exact structure see ESI†), we observed a selective labelling of the FRα-positive cell line KB-3.1, while no fluorescent labelling was observed for the FRα-negative cell line A-549 (Fig. 5A).

A significantly weaker fluorescence labelling occurred when free folic acid as a competitive binder for FRα was added to the cell medium, indicating a receptor-dependent labelling of the cells. This picture was similar for all folate–fluorescein conjugates tested.

Only with maximally enhanced brightness (B = max), a low signal for FA-treated KB 3.1 cells was visible (Fig. 5A, inset).

A strong labelling was also observed at significantly lowered concentration of FA-1-FITC (70 nM, 5 min, see ESI Fig. S11†), and the signal persisted without visible decay over 72 h (Fig. 5B).

**Fig. 6:**
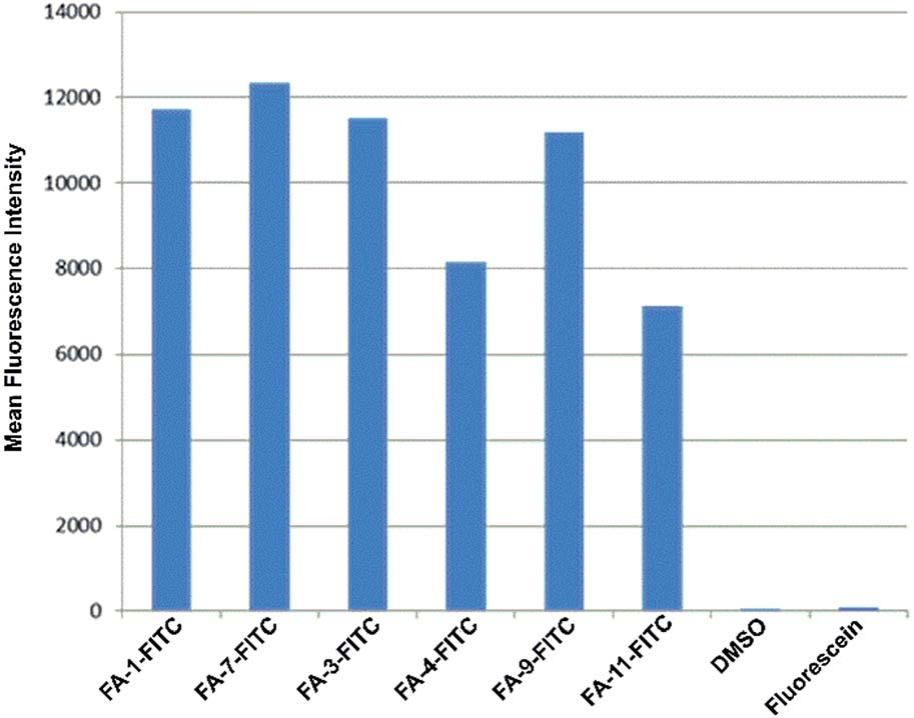
Fluorescence intensity per cell determined by flow cytometry of KB 3.1 cells that were labelled with six different folate–fluorescein conjugates.

The fluorescence intensity per cell was quantified by flow cytometry of KB-3.1 cells that were treated with six folate–fluorescein conjugates. We observed similar mean fluorescence intensities per cell for most of the tested compounds, while free fluorescein did not label the cells (Fig. 6).

However, FA-4-FITC and FA-11-FITC, which are structurally based on the folate carrier molecules FA-N_3_-4 and FA-N_3_-11 and bear two or three fluorophore units, respectively, showed a reduced intensity compared to the other compounds.

### Synthesis and biological evaluation of folate-based ratjadone conjugates

This might be due to an interference of the high fluorophore loading of the compounds with their binding to the receptor. None of the carriers showed any antiproliferative activity up to the highest concentration tested (*ca.* 10 μM) on KB-3.1 as well as A-549 cells, and the cells were completely viable (see ESI, Table S3†).

**Fig. 7:**
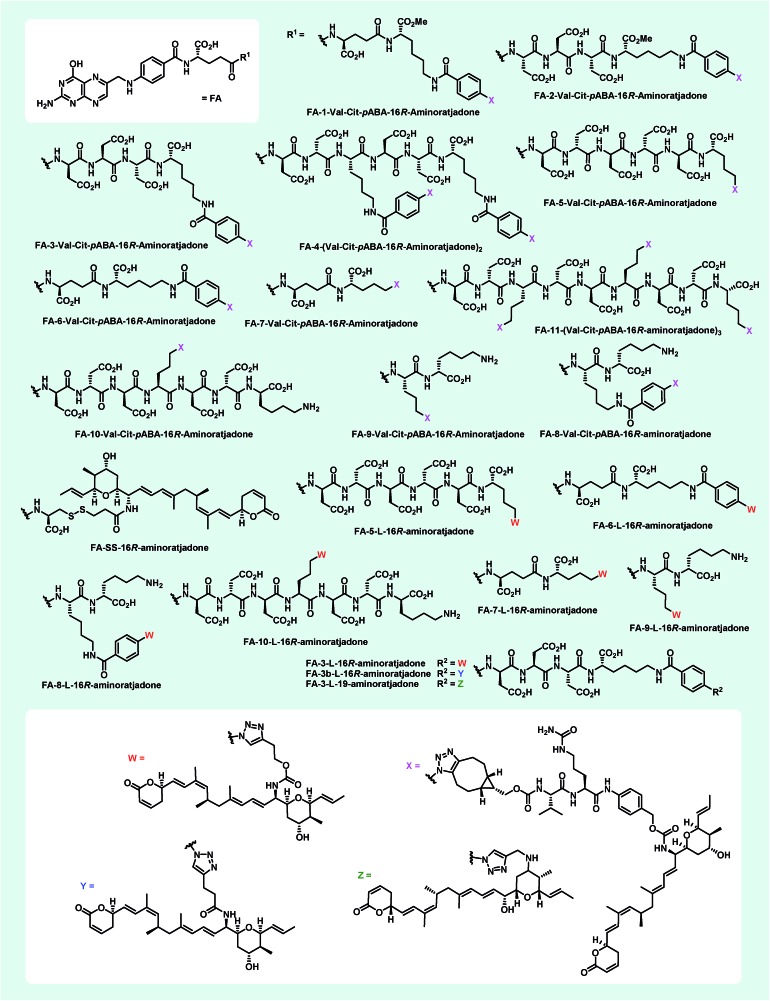
Novel folate–aminoratjadone conjugates with non-cleavable and enzymatically cleavable linkers synthesized in this study.

With appropriate folate carrier molecules in hand, we synthesized 11 novel folate–aminoratjadone conjugates bearing a cathepsin B-cleavable valine-citrulline-*p*ABA unit (Fig. 7). The aminoratjadones **25** and **26** were coupled to different folate carrier molecules either with a metal-free azide–alkyne click reaction or with a copper(i)-mediated azide alkyne click reaction, respectively.

Additionally, 9 folate–aminoratjadone conjugates linked *via* short non-cleavable linkers were synthesized in presence of **21** or **35** (Fig. 7). Furthermore, reacting **31** with **FA-SH-1** in an aqueous buffer system (pH = 7.4) gave the **FA-SS-16*R*-aminoratjadone**, that contained a cleavable disulfide bridge (Fig. 7).

All folate–aminoratjadone conjugates were then evaluated for their antiproliferative activity against the folate receptor-positive cell line KB-3.1 and found to exhibit potent antiproliferative activity (Table 2). However, the conjugation was associated with a moderate loss of activity compared to the free aminoratjadones. This might be due to a reduced target affinity caused by steric interactions, in particular with the nearby helix H12A of CRM-1.^55^ In addition, the internalization efficiency of receptor-mediated, selective uptake might be reduced compared to the freely diffusing unconjugated molecules.

**Table 2:** Antiproliferative activity of novel folate–Ratjadone conjugates against KB-3.1 cancer cells

Folate–ratjadone conjugate	IC_50_ [nM]
**FA-1-Val-Cit-*p*ABA-16*R*-aminoratjadone**	169
**FA-2-Val-Cit-*p*ABA-16*R*-aminoratjadone**	336
**FA-3-Val-Cit-*p*ABA-16*R*-aminoratjadone**	210
**FA-4-(Val-Cit-*p*ABA-16*R*-aminoratjadone)_2_**	148
**FA-5-Val-Cit-*p*ABA-16*R*-aminoratjadone**	237
**FA-6-Val-Cit-*p*ABA-16*R*-aminoratjadone**	223
**FA-7-Val-Cit-*p*ABA-16*R*-aminoratjadone**	34.3
**FA-8-Val-Cit-*p*ABA-16*R*-aminoratjadone**	39.9
**FA-9-Val-Cit-*p*ABA-16*R*-aminoratjadone**	50.9
**FA-10-Val-Cit-*p*ABA-16*R*-aminoratjadone**	35.3
**FA-11-(Val-Cit-*p*ABA-16*R*-aminoratjadone)_3_**	45.0
**FA-3-l-16*R*-aminoratjadone**	29.8
**FA-3-l-19-aminoratjadone**	48.3
**FA-3b-l-16*R*-aminoratjadone**	94.0
**FA-5-l-16*R*-aminoratjadone**	50.3
**FA-6-l-16*R*-aminoratjadone**	150
**FA-7-l-16*R*-aminoratjadone**	190
**FA-8-l-16*R*-aminoratjadone**	129
**FA-9-l-16*R*-aminoratjadone**	47.6
**FA-10-l-16*R*-aminoratjadone**	708
**FA-SS-16*R*-aminoratjadone**	295

The most potent conjugate bearing a cleavable valine-citrulline-*p*ABOH linker was **FA-7-Val-Cit-*p*ABA-16*R*-aminoratjadone** with an IC_50_ value of 34.3 nM. Surprisingly, **FA-SS-16*R*-amino-ratjadone** bearing a disulfide linker showed a significant drop in antiproliferative activity (IC_50_ of 295 nM). This might be due to an inactivation of the drug's warhead by a 1,4-conjugate addition of the released sulfhydryl residue upon cleavage. Obviously, the cathepsin B-specific cleavage site of the conjugates was not essential for a high antiproliferative activity, because several conjugates bearing short, non-cleavable linkers were comparably active; **FA-3-L-16*R*-aminoratjadone** with an IC_50_ of 29.8 nM was even the most potent conjugate in the series. However, we cannot exclude that the hydrophilic peptide spacer in the folate carrier molecules gets (partially) hydrolyzed in the lysosomal compartment after endocytotic uptake. The comparable potency of 19-aminoratjadones and 16-aminoratjadones reported above was also found for the full conjugates, as **FA-3-l-19-aminoratjadone** showed an IC_50_ value of 48.3 nM. The spread in potency between the most potent and the weakest compound was about 20-fold, which underlines the crucial importance of the linker and the need to study a broader set of linkers. We observed that longer linkers were disfavored in the cleavable Val-Cit series (compare **FA-5-Val-Cit-*p*ABA-16*R*-aminoratjadone***vs.***FA-7-Val-Cit-*p*ABA-16*R*-aminoratjadone**). However, other parameters like the net charge of the linker, aryl-azide *vs.* alkyl-azide attachment chemistry, or the number of payloads did not have an obvious positive or negative impact on SAR.

### Synthesis and biological evaluation of LHRH-based ratjadone conjugates

**Fig. 8:**
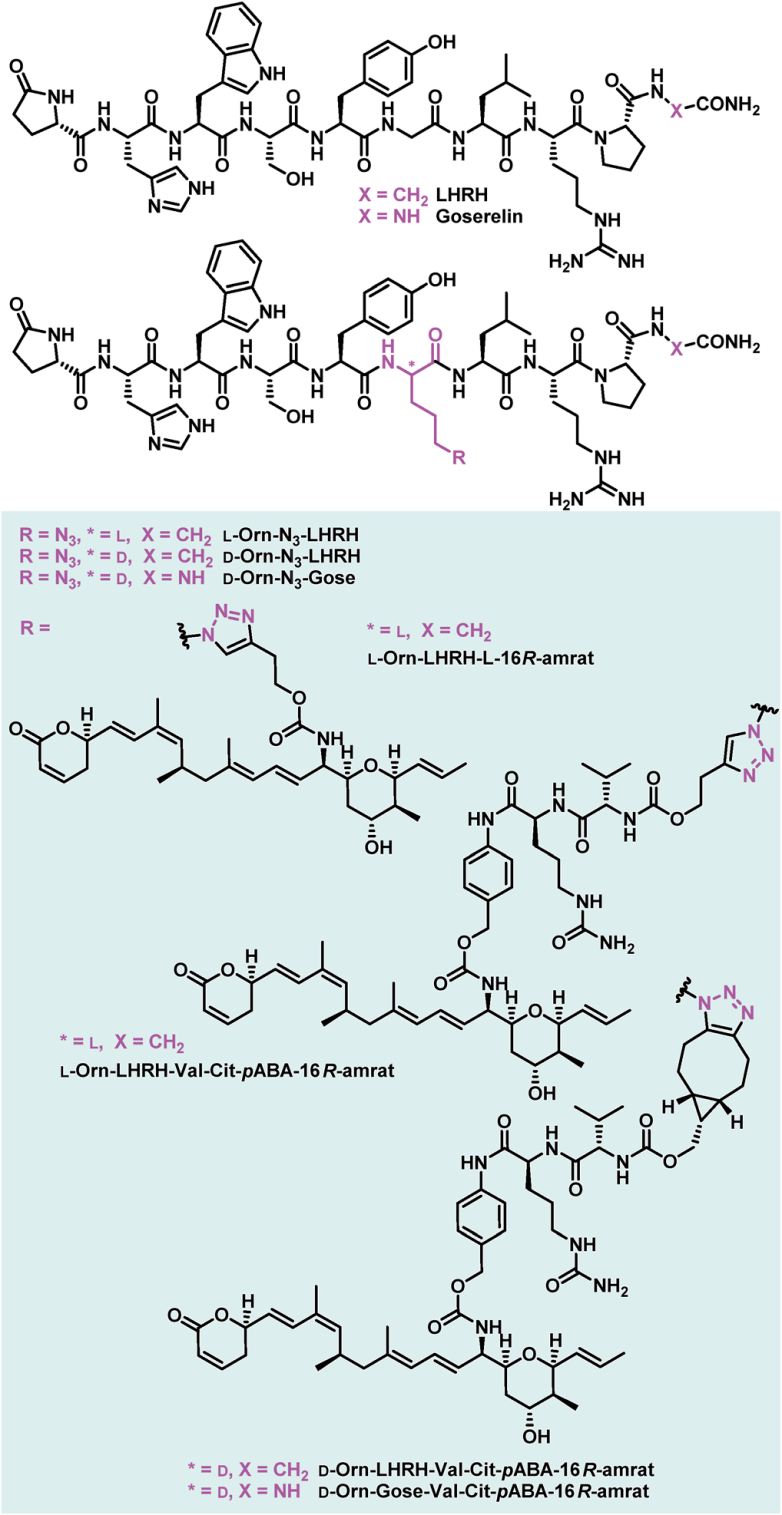
Novel LHRH derivatives and LHRH-aminoratjadone conjugates synthesized in this study.

**Fig. 9:**
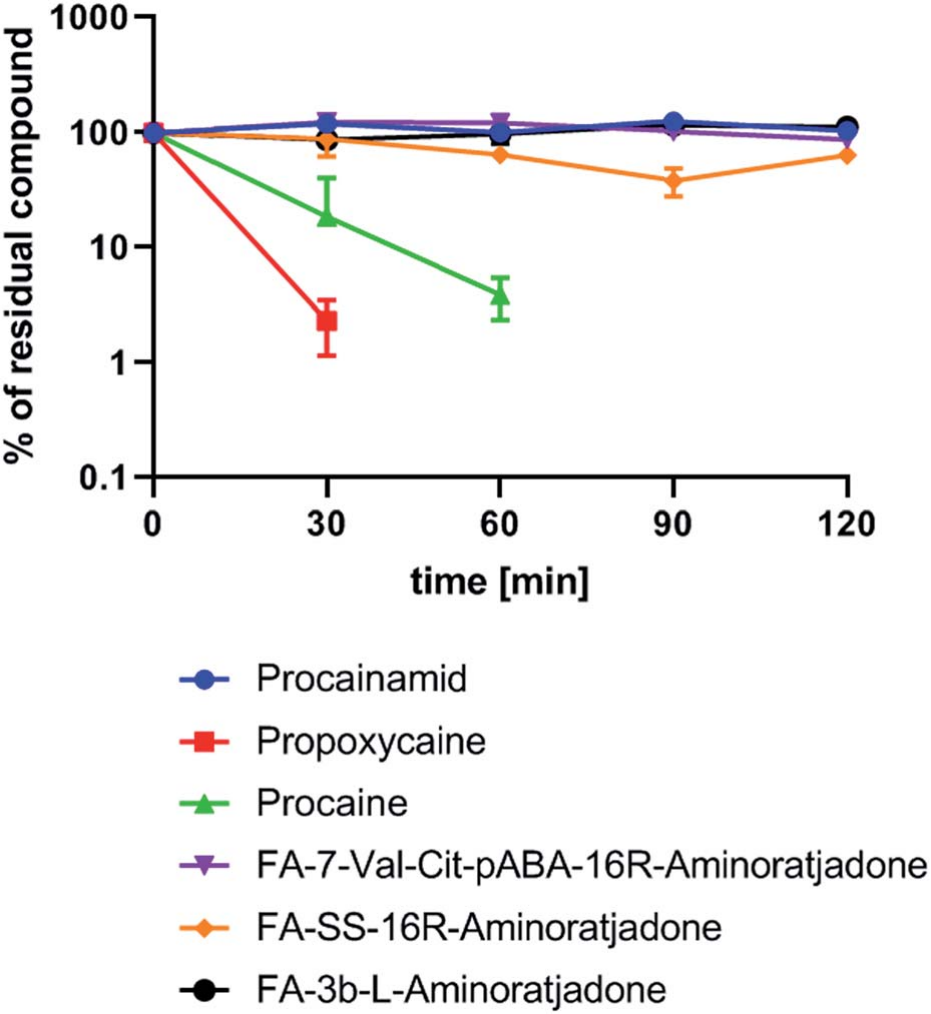
Human plasma stability of selected folate–aminoratjadone conjugates and reference compounds (10 μg mL^–1^ in human plasma at 37 °C and pH = 7.4).

Encouraged by the results from the folate conjugates, we wanted to demonstrate the general applicability of ratjadone payloads by using a second targeting moiety. Luteinizing hormone releasing hormone (LHRH)-based carriers were selected for this purpose, because it has been shown that several cancer cell types overexpress receptors for LHRH, a peptide-hormone involved in regulation of the sex glands of males and females.^25^,^26^,^29^,^82^–^84^ We synthesized three novel derivatives of LHRH that bear azido-ornithine as fifth amino acid of the decapeptide core structure instead of the glycine residue found in the natural peptide (Fig. 8). As the importance of the newly introduced stereocenter was unclear, both diastereoisomers **l-Orn-N_3_-LHRH** and **d-Orn-N_3_-LHRH** were prepared. An additional analog, **d-Orn-N_3_-Gose**, was based on the synthetic LHRH analogue goserelin, that was reported to have higher affinity for the LHRH-receptor^85^ due to a hydrazine carboxamide in the C-terminal position. The compounds were synthesized using TBTU-mediated solid phase-supported peptide coupling on a Rink amide resin. Next, their antiproliferative activities were evaluated in a panel of cancer cell lines, in order to assess a contribution of the drug-free targeting moiety to bioactivity. While **LHRH**, **l-Orn-N_3_-LHRH** and **d-Orn-N_3_-Gose** had no antiproliferative activity, we were surprised to find that **d-Orn-N_3_-LHRH** displayed moderate activities against KB-3.1, A-549, SK-OV-3 and a high (IC_50_ of 47.4 nM) activity against MCF-7 (Table 3 and ESI, Table S3†). To obtain full LHRH-aminoratjadone conjugates, **21**, **25** or **26** were coupled to the LHRH analogs under metal-free or copper-mediated azide–alkyne click conditions (Fig. 9). The conjugates were evaluated for their antiproliferative activity against the LHRH-receptor-positive lung cancer cell line A-549 (Table 3). Cleavable and non-cleavable conjugates based on **l-Orn-N_3_-LHRH** as carrier molecule showed only moderate antiproliferative activity, but **d-Orn-LHRH-Val-Cit-*p*ABA-16*R*-aminoratjadone** and **d-Orn-Gose-Val-Cit-*p*ABA-16*R*-aminoratjadone** were highly potent with IC_50_ values of 23.0 nM and 12.8 nM, respectively. While a contribution of the carrier to bioactivity cannot be excluded for **d-Orn-LHRH-Val-Cit-*p*ABA-16*R*-aminoratjadone**, the effect of the latter compound is ascribed to the ratjadone payload. In fact, **d-Orn-Gose-Val-Cit-*p*ABA-16*R*-aminoratjadone** represents the most active aminoratjadone conjugate synthesized in this study.

**Table 3:** Antiproliferative activities of LHRH-ratjadone conjugates and **d-Orn-N_3_-LHRH** against the LHRH-receptor-positive cancer cell line A-549

LHRH-ratjadone conjugate	IC_50_ [nM]
**l-Orn-N_3_-LHRH**	318
**l-Orn-LHRH-16*R*-aminoratjadone**	1600
**l-Orn-LHRH-Val-Cit-*p*ABA-16*R*-aminoratjadone**	1200
**d-Orn-LHRH-Val-Cit-*p*ABA-16*R*-aminoratjadone**	23.0
**d-Orn-Gose-Val-Cit-*p*ABA-16*R*-aminoratjadone**	12.8

Finally, a first experiment to assess whether aminoratjadone conjugates are sufficiently stable for *in vivo* applications was conducted, given that the amide and carbamate groups in the molecules are potentially labile.

We exposed three selected conjugates, **FA-7-Val-Cit-PABA-16*R*-aminoratjadone**, **FA-SS-16*R*-aminoratjadone** and **FA-3b-16*R*-aminoratjadone**, to human plasma and quantified their relative amount at different time points by HPLC-MS/MS analysis (Fig. 9).

Three reference compounds were included in the set: whereas procain as well as propoxycain are known to degrade rapidly in plasma, procainamide is stable.^86^ Fortunately, all tested conjugates were found to be stable over a period of two hours, whereas the positive controls procain and propoxycain were degraded by more than 90%. Since receptor binding of comparable conjugates normally occurs within <15 minutes after intravenous administration,^87^,^88^ and clearance from the blood stream is >99% completed after 20 minutes,^87^,^89^ a stability over two hours is considered as being sufficient for *in vivo* applications.

## Conclusions

In order to establish nuclear export inhibition as a novel effector principle for extracellular-targeted cancer therapies, we have conjugated the highly potent CRM-1 inhibitor ratjadone A to small molecule targeting ligands. A semi-synthesis program with gram quantities of the natural product led to a reliable access to novel 16- and 19-aminoratjadone derivatives. The compounds fully retained the potent antiproliferative activity and target selectivity of the free natural product at increased water solubility. The ability of aminoratjadones to serve as cytotoxic payloads was addressed by the synthesis and characterization of a series of folate- and LHRH-based EDCs with a broad variety of linkers harboring different cleavage mechanisms. The selective targeting of receptor-positive cells was demonstrated by confocal imaging of fluorescein conjugates. Moreover, aminoratjadon-loaded EDC's showed potent antiproliferative activities against receptor-positive cancer cell lines in the double-digit nM range (*e.g.***FA-7-Val-Cit-*p*ABA-16*R*-aminoratjadone** (IC_50_ = 34.3 nM), **FA-3-L-16*R*-aminoratjadone** (IC_50_ = 29.8 nM), or **d-Orn-Gose-Val-Cit-*p*ABA-16*R*-aminoratjadone** (IC_50_ = 12.8 nM)). A complete profiling of the conjugates in a large cell line panel of varying degrees of folate receptor expression to assess the conjugate's selectivity will be performed in future studies. As the conjugates are stable in human plasma, they appear suited to enter an *in vivo* proof-of-concept in animals. Furthermore, the clickable ratjadone-linker modules can be readily conjugated to other targeting formats like antibodies or aptamers, thereby opening interesting extended applications of the aminoratjadone effector platform.

## Author contributions

The research was planned and the manuscript was written by P. K. and M. B.. P. K. synthesized all new ratjadone derivatives, linkers, drug-conjugates and fluorophore-conjugates. A. R. and T. A. contributed to the isolation of the natural product and supported in the synthesis of linkers and conjugates. V. F. planned and executed CRM1 activity assays of ratjadone derivatives. V. F. did fluorescence microscopy and flowcytometry of fluorophore-conjugates. V. F. carried out biotin-ratjadone pulldown experiments. W. C. and B. H. carried out cytotoxicity assays of compounds. W. T. performed the solid-supported synthesis of LHRH derivatives. K. R. carried out plasma stability tests on drug-conjugates. S. H. and K. I. M. carried out the fermentation of *Sorangium cellulosum.* J. W. and L. J. identified proteins from Biotin-Ratjadone pulldown experiments.

## Conflicts of interest

Parts of this work is part of a (so far unpublished) patent application: new targeted cytotoxic ratjadone derivatives and conjugates thereof, P. Klahn, M. Brönstrup, V. Fetz, S. Hüttel, K. I. Mohr, W. Tegge and W. Collisi, 2017, EP2017 185598.4 (Eur. Patent, Submission: 09.08.2017).

## Supplementary Material

Supplementary information
