# Supplementary material for: The nuclear export inhibitor aminoratjadone is a potent effector in extracellular-targeted drug conjugates
Source: Chem Sci. 2019 Apr 15;10(20):5197–210. doi: 10.1039/c8sc05542d (PMC6540907; doi:10.1039/c8sc05542d)

Electronic Supplementary Material (ESI) for Chemical Science.
This journal is © The Royal Society of Chemistry 2019

The Nuclear Export Inhibitor Aminoratjadone is a Potent Effector in Extracellular-Targeted Drug Conjugates

Philipp Klahn*[a,c], Verena Fetz[a], Antje Ritter[a], Wera Collisi[a,b], Bettina Hinkelmann[a], Tatjana Arnold[a], Werner Tegge[a], Katharina Rox[a], Stephan Hüttel[b], Kathrin I. Mohr[b], Joachim Wink[b], Marc Stadler[b], Josef Wissing[d], Lothar Jänsch[d], Mark Brönstrup*[a,e,f]

[a] Department of Chemical Biology, Helmholtz Centre for Infection Research, Inhoffenstrasse 7, 38124 Braunschweig, Germany

[b] Department of Microbial Drugs, Helmholtz Centre for Infection Research, Inhoffenstrasse 7, 38124 Braunschweig, Germany

[c] Institute of Organic Chemistry, Technische Universität Braunschweig, Hagenring 30, 38106 Braunschweig, Germany

[d] Department of Structure and Function of Proteins; Research Group Cellular Proteomic, Helmholtz Centre for Infection Research, Inhoffenstrasse 7, 38124 Braunschweig, Germany

[e] Biomolecular Drug Research Center (BMWZ), Schneiderberg 38, 30167 Hannover, Germany

[f] German Centre of Infection Research (DZIF), Partner site Hannover-Braunschweig, Germany

Content

Experimental details 2

General Material and Equipment 2

Fermentation and isolation of Ratjadone A 4

Synthesis of the compounds 7

Derivatization of Ratjadone A 7

Synthesis of appropriate carrier molecules 36

Synthesis of imaging probes 60

Conjugation of Ratjadone derivatives to carrier molecules 71

Biological evaluation of the compounds 110

Cell proliferation assay 110

Cell proliferation assay under folate free conditions 112

Monitoring export inhibitory activity 114

Analysis of cell labeling with fluorescein-labeled conjugates 116

Streptavidin-Pull-down with Ratjadone-biotin and identification of exportin by LC-MSMS 118

Plasma stability assay 121

References 124

Appendix 125

^{1}H-NMR spectra, ^{13}C-NMR spectra, IR spectra 125

Experimental details

General Material and Equipment

Unless otherwise noted, all reagents were purchased from commercial suppliers and used without further purification. All solvents used for workup and purification were of HPLC grade. Moisture sensitive reactions were performed under argon atmosphere in dried glass ware. Reactions were monitored by TLC, LCMS or NMR.

Flash chromatography was done either manually using appropriate glass columns filled with silicagel (Merck, Silicagel 60, 1.15111.1000, 15-40 µm) or using the Reveleris® X2 flash chromatography system and prepacked cartridges (Reveleris® Flash Cartridges Silica 40µm) from the company Büchi.

Preparative reversed phase high pressure liquid chromatography (prep. HPLC RP) was performed on a Phenomenex Gemini C18 RP-column 00G-4436-NO, 10 µm, 110 A, 250×10.00 mm (5 mL/min) or a Phenomenex Gemini C18 RP-column 00G-4435-PO-AX, 5 µm, 110 A, 250×21.20 mm (9 mL/min) or a Thermo Fisher Scientific BDS Hypersil C18 RP-column 28105-259370, 5 µm, 250x30 mm, (25 mL/min) or a Macherey-Nagel Nucleosil 100-7 VP C18 RP column715691-1116949, 250×40 mm (45 mL/min) using a Thermo Fisher Scientific Dionex Ultimate 3000 HPLC system. Eluents, gradients and additives are given in parentheses. Product containing fractions were combined diluted with dest. H_2O (min. 1:1/solvent:H_2O), frozen and lyophilized.

Thin-layer chromatography (TLC) was performed on pre-coated glass plates (Merck TLC Silicagel 60 F_{254}, 1.15341.0001, 2.5x7.5 cm) and components were visualized by observation under UV light (λ = 254 nm [UV^{254}] or λ = 366 nm [UV^{366}]), treatment of developed plates in an iodine chamber or by treating the plates with TLC staining solutions (for preparation see list below) followed by heating. Eluent or eluent-mixtures used are reported in parentheses.

$KMnO_4$ staining solution [$KMnO_4$]: 1.5 g $KMnO_4$, 10 g K_2CO_3, and 1.25 mL 10% NaOH in 200 mL H_2O.

PMA staining solution [PMA]: 10 g phosphomolybdic acid in 100 mL abs. EtOH.

CAM staining solution [CAM]: 1 g Ce(IV)$(SO_4)_2$, 2.5 g $(NH_4)_6Mo_4O_7$ in 100 mL 10% H_2SO_4

Anisaldehyd staining solution [AA]: 135 mL abs. EtOH, 5 mL conc. H_2SO_4, 1.5 mL HOAc and 3.7 mL *p*-anisaldehyde.

Ninhydrin staining solution [Ninhydrin]: 1.5 g ninhydrin in 100 mL *abs.* EtOH and 3.0 mL HOAc.

Vanillin staining solution [Van]: 15 g vanillin in 250 mL *abs.* EtOH and 2.5 mL conc. H_2SO_4.

Preparative thin-layer chromatography was performed on pre-coated glass plates (Merck TLC Silicagel 60 F_{254}, 1.05715.0001, 20x20 cm, max. 10-15 mg/plate and Analtech Uniplate Silica gel GF Z51305-9, 20x20 cm x 2 mm, max 100-150 mg/plate). Eluent or eluent-mixtures used and number of developments are reported in parentheses. Compounds were visualized by observation under UV light (λ = 254 or 366 nm). Compound containing silica gel fractions were scratched from the plate with a scapell, crushed to small pieces and compounds were eluated by appropriate solvent mixtures.

NMR spectra were recorded on a Bruker Avance III or a Bruker Avance III HD with cryoprobe system. ^{1}H NMR spectra were recorded at 500 MHz and 700 MHz. ^{13}C NMR spectra were recorded at 126 MHz and 176 MHz. Samples of final conjugates were prepared in *Shigemi*-NMR tubes matched with DMSO-d_6 Chemical shifts are reported in ppm relative to solvent signal. Multiplicity is indicated as follows: s (singlet); bs (broad singlet); d (doublet); t (triplet); q (quartet); m (multiplet); dd (doublet of doublets), etc.

Optical rotation values were determined on a Perkin-Elmer 241 MC and calculated along Lambert-Beer's equation.

IR spectra were recorded on a Bruker ALPHA FT IR spectrometer with ATR-technique. Only the wave numbers of observed absorption peaks are given.

Low resolution mass spectrometry (LRMS) data were recorded using an Agilent 1100 HPLC system equipped with DAD detector and an API 150 EX quadrupole mass detector with electron spray ionization (ESI) (ACN-H_2O + 0.05% TFA) or a Dionex Ultimate 3000 HPLC system equipped with a DAD detector and a Bruker amazon ion trap mass detector with electron spray ionization (ESI).

High resolution mass spectrometry (HRMS) data were recoreded using a Dionex Ultimate 3000 HPLC system equipped with a DAD detector and a Bruker maXis HD QTOF mass detector with electron spray ionization (ESI).

Fermentation and isolation of Ratjadone A

Isolation of Ratjadone A (1) - (R)-6-((1E,3Z,5R,7E,9E,11R)-11-hydroxy-11-((2S,4R,5S,6S)-4-hydroxy-5-methyl-6-((E)-prop-1-en-1-yl)tetrahydro-2H-pyran-2-yl)-3,5,7-trimethylundeca-1,3,7,9-tetraen-1-yl)-5,6-dihydro-2H-pyran-2-one

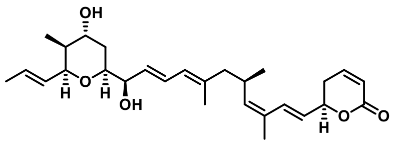

Chemical Formula: $C_{28}H_{40}O_5$
Molecular Weight: 456,62

Ratjadone A **1**

Fermentation and isolation of Ratjadone A was done similar to the published procedures[1–3] with some changes mentioned below and utilizing an optimized strain or the myxobacterium *Sorangium cellulosum* (Soce 1047 = DSM 32464).

The producing organism, the myxobacterium *Sorangium cellulosum* Soce1047, was isolated at the Helmholtz Centre of Infection Research, former GBF in 1989 from a soil sample collected at Cala Ratjada (Mallorca, Spain). Seed cultures on yeast agar were inoculated into 250-mL Erlenmeyer flasks containing 100 mL of medium.

The basic medium for growth and production had the following composition (in g/L distilled water): soybean flour (defatted) 4, glucose monohydrate 2, potato starch 8; $MgSO_4$ 7 H_2O 1; $CaCl_2$ 2 H_2O 1; ethylenediaminetetraacetic acid iron(III)-sodium salt 0.008. The pH of the medium was adjusted to 7.3 with KOH before autoclaving. In both media Soce1047 grew in small lumps, so that growth could not be measured optically or by counting the cell numbers.

A 20-L bioreactor (Giovanola Freres, Monthey, Switzerland) with 6 L of the production medium was inoculated with 1 L of a 4-day old preculture grown in the same medium in 1-L Erlenmeyer flasks with 500 mL medium under shaking (160 rpm, 30°C). For continuous adsorption of the produced ratjadone, 100 mL of the adsorber resin XAD-16 (Rohm and Haas, Frankfurt/M) was added before autoclaving. To prevent foam formation, 10 mL silicone antifoam (Tegosipon, Goldschmidt AG Essen) was added. The fermentation was run for 7 days at 30°C and the pH adjusted to 7.3-7.5 (2.5% H_2SO_4/2.5% KOH) with an aeration rate of 300 Lair per hour and a stirrer speed of 300 rpm. Ratjadon was excreted into the culture broth during the growth phase and became quantitatively adsorbed to the resin the end of the fermentation.

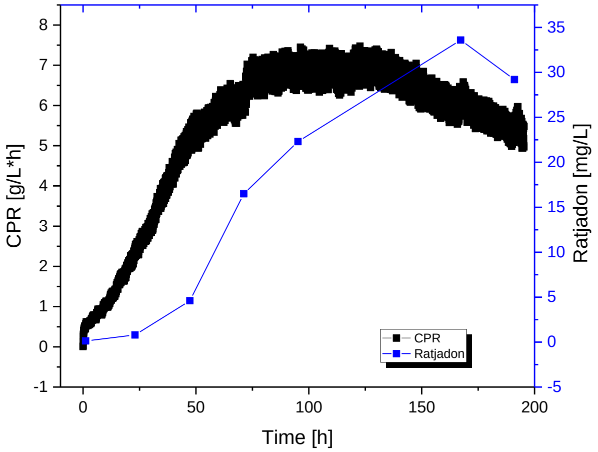

Product titer and CO_2 evolution rate (CPR) over time, product formation is observed during exponential growth phase.

The adsorber resin was separated from the broth by decantation off the cell mass in the counter-flow principle using 2 L of H_2O. After decantion of the remaining water, the XAD-16 adsorber resin was extracted with 2 L of MeOH and the resulting eluent was concentrated under reduced pressure (40°C, 130 mBar). The residue was suspended in 500 mL of H_2O and the mixture was extracted with EtOAc (3x 400 mL). The combined organic layers were dried over anhydrous Na_2SO_4 and concentrated under reduced pressure yielding 1.38 g of raw material. This was further purified by automated flash chromatography through silicagel (Büchi Reveleris® X2, 24 g Büchi Reveleris® Silica Cartridge, flow: 28 mL/min, step gradient: PE:EtOAc/1:1 (5 min), then CH_2Cl_2:MeOH/1:0 (2 min), 99:1 (5 min), 98:2 (5 min), 95:5 (5 min), 9:1 (1 min)) yielding 324 mg (0.709 mmol, 32.4 mg/L of ferments) of Ratjadone A as a pale beige amorph foam.

The fermentation was done on 10L and 70L scale. The best yield was obtained on 10L scale with 32.4 mg of Ratjadone A/L. A total amount of 3.646 g of Ratjadone A was isolated from 150 L Fermentation culture.

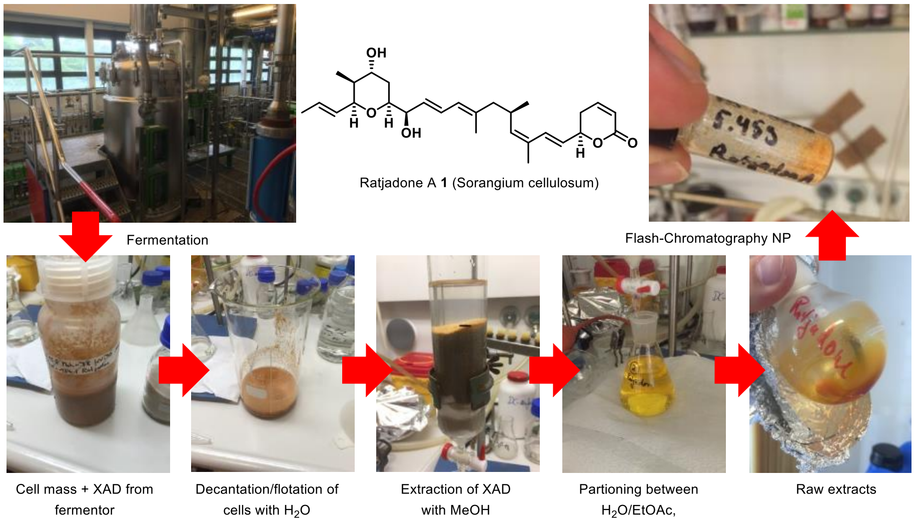

Ratjadone A (1): TLC (CH_2Cl_2:MeOH/95:5) R_f: 0.35 [UV^{254}, CAM], **IR** (ATR) [cm^{-1}]: 3417, 2962, 2921, 2871, 1715, 1653, 1625, 1451, 1438, 1380, 1344, 1295, 1245, 1149, 1121, 1056, 1011, 963, 916, 881, 814, 732, 660. **^{1}H-NMR** (500 MHz, $CDCl_3$) δ [ppm]: 6.88 (ddd, *J* = 9.7, 4.6, 3.9 Hz, 1H), 6.70 (d, *J* = 15.6 Hz, 1H), 6.45 (ddd, *J* = 15.2, 10.9, 1.3 Hz, 1H), 6.09 – 5.99 (m, 1H), 5.76 (d, *J* = 11.0 Hz, 1H), 5.72 – 5.69 (m, 1H), 5.68 – 5.66 (m, 1H), 5.54 – 5.47 (m, 1H), 5.46 – 5.37 (m, 1H), 5.21 (d, *J* = 9.5 Hz, 1H), 5.03 – 4.93 (m, 1H), 4.44 (d, *J* = 6.2 Hz, 1H), 4.32 (dd, *J* = 6.0, 2.8 Hz, 1H), 3.98 (q, *J* = 2.8 Hz, 1H), 3.85 (dt, *J* = 12.2, 2.8 Hz, 1H), 2.79 (dq, *J* = 9.6, 6.9 Hz, 1H), 2.50 – 2.39 (m, 2H), 1.98 (d, *J* = 7.2 Hz, 2H), 1.86 (ddd, *J* = 14.9, 12.3, 2.9 Hz, 1H), 1.77 (d, *J* = 1.2 Hz, 3H), 1.70 (s, 6H), 1.70 – 1.68 (m, 2H), 1.65 – 1.58 (m, 2H), 1.43 – 1.34 (m, 1H), 0.90 (d, *J* = 6.6 Hz, 3H), 0.89 (d, *J* = 7.2 Hz, 3H). **^{13}C-NMR** (126 MHz, $CDCl_3$) δ [ppm]: 164.34, 144.95, 139.55, 137.70, 130.81, 130.37, 129.45, 128.61, 128.51, 127.10, 126.08, 125.52, 121.84, 78.84, 74.97, 74.91, 74.57, 70.42, 47.97, 39.82, 30.74, 30.26, 26.98, 21.20, 20.60, 18.16, 17.29, 11.34. **LRMS** (ESI-Quad) [m/z]: 479.2 $[M+Na]^+$, 421.3 $[M-H_2O+H]^+$, **HRMS** (ESI-IT) [m/z]: 479.277556, calculated 479.276795 for $C_{28}H_{40}NaO_5$ $[M+Na]^+$, err [ppm] 1.588.

Synthesis of the compounds

Derivatization of Ratjadone A

Synthesis of 16-Aminoratjadones

Synthesis of 16-Oxoratjadone (13) - (R)-6-((R,1E,3Z,7E,9E)-11-((2S,4R,5S,6S)-4-hydroxy-5-methyl-6-((E)-prop-1-en-1-yl)tetrahydro-2H-pyran-2-yl)-3,5,7-trimethyl-11-oxoundeca-1,3,7,9-tetraen-1-yl)-5,6-dihydro-2H-pyran-2-one, **19-Oxoratjadone (14)** - (R)-6-((1E,3Z,5R,7E,9E,11R)-11-hydroxy-3,5,7-trimethyl-11-((2S,5R,6S)-5-methyl-4-oxo-6-((E)-prop-1-en-1-yl)tetrahydro-2H-pyran-2-yl)undeca-1,3,7,9-tetraen-1-yl)-5,6-dihydro-2H-pyran-2-one and **16,19-Dioxoratjadone (15)**– (R)-6-((R,1E,3Z,7E,9E)-3,5,7-trimethyl-11-((2S,5R,6S)-5-methyl-4-oxo-6-((E)-prop-1-en-1-yl)tetrahydro-2H-pyran-2-yl)-11-oxoundeca-1,3,7,9-tetraen-1-yl)-5,6-dihydro-2H-pyran-2-one

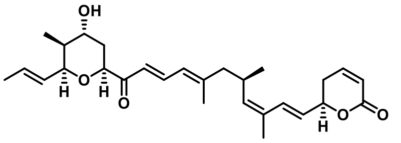

Chemical Formula: $C_{28}H_{38}O_5$
Molecular Weight: 454,61

16-Oxoratjadone **13**

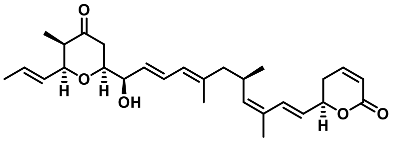

Chemical Formula: $C_{28}H_{38}O_5$
Molecular Weight: 454,61

19-Oxoratjadone **14**

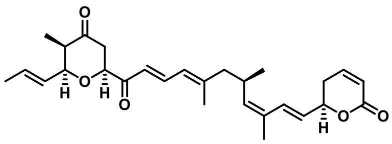

Chemical Formula: $C_{28}H_{36}O_5$
Molecular Weight: 452,59

16,19-Dioxoratjadone **15**

A solution of 33.7 mg (0.1205 mmol, 1.1 equiv) IBX[i] in 750 µL DMSO was added dropwise over a period of 16 h (Syringe Pump) at 23°C to a stirred solution of 55 mg (0.1095 mmol, 1.0 equiv) of Ratjadone A **1** dissolved in 750 µL DMSO. Afterwards the mixture was stirred for further 24 h at 23°C. The reaction was monitored by HPLC-MS. The mixture was diluted with CH_2Cl_2 (15 mL) and stirred for 30 min until IBA precipitates as white solid and could be filtered off using a cellulose filter. The filtrate was washed with H_2O (3x 15 mL)[ii], dried over anhydrous Na_2SO_4 and was concentrated under reduced pressure. The residue was purified by preparative thin-layer chromatography (CH_2Cl_2: MeOH/98:2, 2x Development), yielding 34.5 mg (0.076 mmol, 69%, 75% brsm) 16-oxoratjadone **13** as a pale-yellow solid foam, 3.7 mg (8.1 µmol, 7%, 8% brsm) 19-oxoratjadone **14** as a pale-yellow solid foam, 6.9 mg (15.2 µmol, 14%, 15% brsm) 16,19-

---

[i] IBX was freshly prepare from 2-iodobenzoic acid according to the procedure of *Santagostino* and co-workers.[16]

[ii] Important to get rid of DMSO traces, which have a strongly negative influence to the chromatographic separation of the products.

dioxoratjadone **15** as a pale-yellow solid foam and 4.0 mg (8%) reisolated ratjadone A **1**. The reaction was done in 10 - 600 mg scale, obtaining similar product distributions and yields.

16-Oxoratjadone (13): TLC (CH_2Cl_2:MeOH/98:2) R_f: 0.26 [UV^{254}, CAM], **IR** (ATR) [cm^{-1}]: 3456, 2970, 2922, 2883, 1723, 1682, 1620, 1581, 1451, 1437, 1380, 1362, 1307, 1247, 1218, 1126, 1084, 1056, 1013, 968, 914, 884, 783, 732, 700. **^{1}H-NMR** (700 MHz, $CDCl_3$) δ [ppm]: 7.61 (dd, *J* = 15.1, 11.7 Hz, 1H), 6.88 (ddd, *J* = 9.8, 5.3, 3.1 Hz, 1H), 6.70 (d, *J* = 15.6 Hz, 1H), 6.50 (d, *J* = 15.2 Hz, 1H), 6.04 (ddd, *J* = 9.8, 2.3, 1.3 Hz, 1H), 5.99 (d, *J* = 11.7 Hz, 1H), 5.77 – 5.70 (m, 1H), 5.73 – 5.67 (m, 1H), 5.50 (ddq, *J* = 15.4, 6.1, 1.6 Hz, 1H), 5.19 (d, *J* = 9.6 Hz, 1H), 5.00 (dddd, *J* = 10.2, 6.3, 5.2, 1.1 Hz, 1H), 4.47 – 4.45 (m, 1H), 4.43 (dd, *J* = 11.1, 3.7 Hz, 1H), 4.01 (q, *J* = 2.7 Hz, 1H), 2.86 (ddq, *J* = 13.8, 9.7, 6.8 Hz, 1H), 2.51 – 2.39 (m, 2H), 2.10 (dd, *J* = 7.1, 2.2 Hz, 2H), 1.85 (d, *J* = 1.1 Hz, 3H), 1.78-1.73 (m, 2H), 1.77 (d, *J* = 1.2 Hz, 3H), 1.71 (dt, *J* = 6.5, 1.3 Hz, 3H), 1.68 (dtt, *J* = 6.7, 2.7, 1.5 Hz, 1H), 0.93 (d, *J* = 0.8 Hz, 3H), 0.92 (d, *J* = 1.5 Hz, 3H). **^{13}C-NMR** (176 MHz, $CDCl_3$) δ [ppm]: 199.85, 164.29, 150.49, 144.94, 140.32, 138.65, 130.24, 130.13, 129.89, 127.14, 126.05, 125.99, 122.96, 121.84, 78.54, 75.09, 70.23, 48.47, 39.30, 30.87, 30.76, 30.23, 29.92, 21.26, 20.60, 18.17, 18.13, 11.44. **LRMS** (ESI-Quad) [m/z]: 477.6 $[M+Na]^+$, 455.2 $[M-H_2O+H]^+$, **HRMS** (ESI-IT) [m/z]: 455.279347, calculated 455.279201 for $C_{28}H_{39}O_5$ $[M+H]^+$, err [ppm] - 0.321.

19-Oxoratjadone (14): TLC (CH_2Cl_2:MeOH/98:2) R_f: 0.36 [UV^{254}, CAM], **IR** (ATR) [cm^{-1}]: 3450, 2970, 2923, 2857, 1713, 1653, 1452, 1419, 1380, 1353, 1302, 1245, 1140, 1093, 1030, 1014, 967, 915, 882, 815, 732, 653. **^{1}H-NMR** (500 MHz, $CDCl_3$) δ [ppm]: 6.90 (dt, *J* = 9.8, 4.2 Hz, 1H), 6.71 (d, *J* = 15.6 Hz, 1H), 6.50 (ddd, *J* = 15.2, 11.0, 1.3 Hz, 1H), 6.07 (dt, *J* = 9.8, 1.8 Hz, 1H), 5.82 – 5.74 (m, 2H), 5.71 (dd, *J* = 15.6, 6.9 Hz, 1H), 5.54 – 5.41 (m, 2H), 5.23 (d, *J* = 9.6 Hz, 1H), 5.04 – 4.95 (m, 1H), 4.46 (s, 1H), 4.19 (ddt, *J* = 6.1, 2.6, 1.1 Hz, 1H), 3.74 (dt, *J* = 11.8, 3.0 Hz, 1H), 2.85 – 2.76 (m, 1H), 2.75 (dd, *J* = 14.9, 11.9 Hz, 1H), 2.48 (ddd, *J* = 6.7, 3.8, 1.9 Hz, 2H), 2.39 (qdd, *J* = 7.0, 2.5, 1.1 Hz, 1H), 2.16 (ddd, *J* = 14.9, 2.9, 1.3 Hz, 1H), 2.02 (d, *J* = 7.2 Hz, 2H), 1.80 (d, *J* = 1.2 Hz, 3H), 1.75 (ddd, *J* = 6.5, 1.6, 1.0 Hz, 3H), 1.73 (d, *J* = 1.1 Hz, 3H), 1.13 (d, *J* = 7.2 Hz, 3H), 0.93 (d, *J* = 6.6 Hz, 3H). **^{13}C-NMR** (126 MHz, $CDCl_3$) δ [ppm]: 211.63, 164.07, 144.67, 139.22, 138.44, 130.73, 129.41, 129.36, 128.84, 127.42, 127.00, 125.67, 125.39, 121.70, 79.58, 79.33, 78.72, 73.88, 50.12, 47.86, 37.51, 30.52, 30.09, 21.03, 20.42, 17.94, 17.06, 11.13. **LRMS** (ESI-Quad) [m/z]: 477.3 $[M+Na]^+$, 455.4 $[M+H]^+$, **HRMS** (ESI-IT) [m/z]: 477.261253, calculated 477.261145 for $C_{28}H_{38}NaO_5$ $[M+Na]^+$, err [ppm] - 0,225.

16,19-Dioxoratjadone (15): TLC (CH_2Cl_2:MeOH/98:2) R_f: 0.44 [UV^{254}, CAM], **IR** (ATR) [cm^{-1}]: 2968, 2923, 2855, 1717, 1684, 1620, 1582, 1451, 1419, 1381, 1333, 1305, 1245, 1186, 1148, 1100, 1059, 1013, 968, 932, 915, 883, 862, 814, 787, 732, 662. **^{1}H-NMR** (500 MHz, $CDCl_3$) δ [ppm]: 7.68 (dd, *J* = 15.1, 11.7 Hz, 1H),

6.89 (ddd, *J* = 9.7, 5.2, 3.2 Hz, 1H), 6.72 (d, *J* = 15.6 Hz, 1H), 6.55 (d, *J* = 15.1 Hz, 1H), 6.09 – 6.01 (m, 2H), 5.86 – 5.77 (m, 1H), 5.72 (dd, *J* = 15.6, 6.3 Hz, 1H), 5.52 (ddd, *J* = 15.4, 6.0, 1.7 Hz, 1H), 5.22 (d, *J* = 9.6 Hz, 1H), 5.06 – 4.97 (m, 1H), 4.27 – 4.22 (m, 2H), 2.88 (dtd, *J* = 13.7, 6.9, 3.3 Hz, 1H), 2.66 (dd, *J* = 15.0, 12.0 Hz, 1H), 2.52 – 2.40 (m, 4H), 2.14 (d, *J* = 7.3 Hz, 2H), 1.91 – 1.87 (m, 3H), 1.79 (d, *J* = 1.1 Hz, 3H), 1.76 (dt, *J* = 6.4, 1.2 Hz, 3H), 1.15 (d, *J* = 7.2 Hz, 3H), 0.95 (d, *J* = 6.6 Hz, 3H). **^{13}C-NMR** (126 MHz, $CDCl_3$) δ [ppm]: 209.87, 196.26, 164.03, 151.67, 144.70, 141.39, 138.36, 129.95, 129.09, 127.30, 126.07, 125.98, 122.31, 121.82, 84.92, 80.42, 80.20, 78.31, 50.21, 48.60, 39.92, 30.70, 30.19, 21.13, 20.52, 18.07, 18.00, 11.34. **LRMS** (ESI-Quad) [m/z]: 475.2 $[M+Na]^+$, 453.3 $[M+H]^+$, **HRMS** (ESI-IT) [m/z]: 453.263800, calculated 47453.263600 for $C_{28}H_{37}O_5$ $[M+H]^+$, err [ppm] - 0.7.

Synthesis of 16*R*-Aminoratjadone (16) - (R)-6-((1E,3Z,5R,7E,9E,11R)-11-amino-11-((2S,4R,5S,6S)-4-hydroxy-5-methyl-6-((E)-prop-1-en-1-yl)tetrahydro-2H-pyran-2-yl)-3,5,7-trimethylundeca-1,3,7,9-tetra-en-1-yl)-5,6-dihydro-2H-pyran-2-one, **16*S*-Aminoratjadone (17)** - (R)-6-((1E,3Z,5R,7E,9E,11S)-11-amino-11-((2S,4R,5S,6S)-4-hydroxy-5-methyl-6-((E)-prop-1-en-1-yl)tetrahydro-2H-pyran-2-yl)-3,5,7-trimethylun-deca-1,3,7,9-tetraen-1-yl)-5,6-dihydro-2H-pyran-2-one, **Compound 18** - methyl (5R,6E,8Z,10R,12E,14E,16R)-3,16-diamino-5-hydroxy-16-((2S,4R,5S,6S)-4-hydroxy-5-methyl-6-((E)-prop-1-en-1-yl)tetrahydro-2H-pyran-2-yl)-8,10,12-trimethylhexadeca-6,8,12,14-tetraenoate and **Compound 19** – methyl (5R,6E,8Z,10R,12E,14E,16S)-3,16-diamino-5-hydroxy-16-((2S,4R,5S,6S)-4-hydroxy-5-methyl-6-((E)-prop-1-en-1-yl)tetrahydro-2H-pyran-2-yl)-8,10,12-trimethylhexadeca-6,8,12,14-tetraenoate

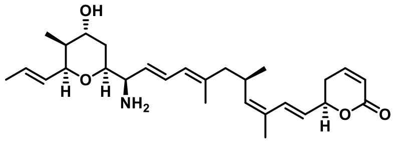

Chemical Formula: $C_{28}H_{41}NO_4$
Molecular Weight: 455,64
16*R*-Aminoratjadone **16**

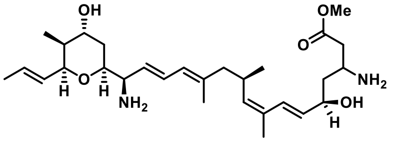

Chemical Formula: $C_{29}H_{48}N_2O_5$
Molecular Weight: 504,71
18

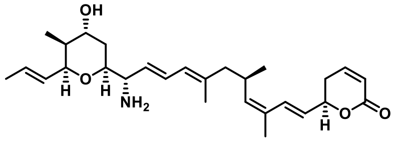

Chemical Formula: $C_{28}H_{41}NO_4$
Molecular Weight: 455,64
16*S*-Amino Ratjadone **17**

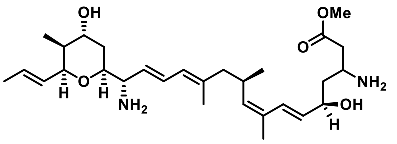

Chemical Formula: $C_{29}H_{48}N_2O_5$
Molecular Weight: 504,71
19

36.6 mg (0.475 mmol, 10 equiv) ammonium acetate were added to a solution of 21.6 mg (47.5 µmol, 1.0 equiv) 16-Oxoratjadone **13** in 950 µL dry MeOH at 23°C and stirred for 3 min, before 5.9 mg (95.0 µmol, 2.0 equiv) sodium cyanoborohydride was added and the mixture was stirred at 23°C for 14 h. Then the mixture was heated for 2 h to 40°C, before the reaction was quenched by addition of 400 µL of ACN:H_2O + TFA/30:70 + 0.05%. This mixture was directly purified by RP prep HPLC (Phenomenex Gemini C18 RP-

column 00G-4435-PO-AX, 5 µm, 110 A, 250×21.20 mm, Flow: 9 mL/min, ACN:H_2O + TFA/ 10:90 + 0.1% → 95:5 + 0.1% in 90 min) yielding after lyophilization 6.9 mg (15.1 µmol, 32%, 34% brsm) 16*R*-aminoratjadone **16** as a pale-yellow solid foam, 4.3 mg (9.4 µmol, 20%, 21% brsm) 16*S*-aminoratjadone **17** as a pale-yellow solid foam, 1.5 mg (3.3 µmol, 7%, 8% brsm) of Compound **18** as a yellow solid foam, 0.4 mg (0.9 µmol, 2%, 2.4% brsm) of Compound **19** as a yellow solid foam and 1.6 mg (7%) of reisolated 16-oxoratjadone **13**. The compounds **16** and **17** were obtained as pure compounds, whereas compounds **18** and **19** were obtained as their TFA salts. The reaction was done in 10 - 100 mg scale, obtaining similar product distributions and yields.

16*R*-Aminoratjadone (16): IR (ATR) [cm^{-1}]: 3403, 2961, 2923, 1674, 1522, 1452, 1436, 1382, 1345, 1253, 1201, 1160, 1134, 1058, 1014, 967, 918, 883, 816, 800, 722, 661. **^{1}H-NMR** (500 MHz, $CDCl_3$) δ [ppm]: 8.05 (s, 2H), 6.92 (dt, *J* = 9.7, 4.2 Hz, 1H), 6.64 (dd, *J* = 24.0, 15.5 Hz, 1H), 6.51 (dd, *J* = 15.1, 10.9 Hz, 1H), 6.03 (dt, *J* = 9.8, 1.7 Hz, 1H), 5.72 (d, *J* = 10.7 Hz, 1H), 5.70 – 5.61 (m, 2H), 5.58 (dd, *J* = 15.2, 9.2 Hz, 1H), 5.36 (ddt, *J* = 15.5, 3.9, 1.6 Hz, 1H), 5.20 (d, *J* = 9.5 Hz, 1H), 5.02 – 4.90 (m, 1H), 4.38 (d, *J* = 5.1 Hz, 1H), 4.17 (d, *J* = 12.2 Hz, 1H), 3.96 – 3.91 (m, 1H), 3.80 (d, *J* = 9.0 Hz, 1H), 2.78 (dt, *J* = 15.2, 8.3 Hz, 1H), 2.48 (ddd, *J* = 7.2, 3.8, 1.6 Hz, 2H), 2.00 (ddt, *J* = 22.4, 14.1, 6.7 Hz, 2H), 1.81 – 1.76 (m, 3H), 1.72 (s, 3H), 1.66 (d, *J* = 6.4 Hz, 3H), 1.69 – 1.53 (m, 2H), 1.40 (d, *J* = 14.0 Hz, 1H), 0.96 (d, *J* = 6.6 Hz, 3H), 0.81 (d, *J* = 7.1 Hz, 3H).**^{13}C-NMR** (126 MHz, $CDCl_3$) δ [ppm]: 164.94, 145.51, 140.31, 139.44, 134.67, 132.19, 129.79, 129.66, 126.71, 125.03, 124.99, 121.45, 120.81, 79.55, 74.77, 71.57, 69.49, 57.54, 47.51, 38.93, 30.57, 30.06, 28.99, 21.55, 20.41, 17.99, 17.48, 11.13. **LRMS** (ESI-Quad) [m/z]: 456.3 $[M+H]^+$, **HRMS** (ESI-IT) [m/z]: 456.311233, calculated 456.310835 for $C_{28}H_{42}NO_4$ $[M+H]^+$, err [ppm] – 0.872.

16*S*-Aminoratjadone (17): IR (ATR) [cm^{-1}]: 3342, 2956, 2925, 1675, 1522, 1436, 1382, 1305, 1254, 1202, 1180, 1136, 1056, 1017, 966, 912, 883, 800, 722, 651. **^{1}H-NMR** (500 MHz, $CDCl_3$) δ [ppm]: 8.07 (s, 2H), 6.98 – 6.87 (m, 1H), 6.69 (d, *J* = 15.5 Hz, 1H), 6.56 (dd, *J* = 15.0, 10.9 Hz, 1H), 6.04 (d, *J* = 9.8 Hz, 1H), 5.75 (d, *J* = 10.8 Hz, 1H), 5.68 (dd, *J* = 15.5, 7.7 Hz, 1H), 5.70 – 5.60 (m, 1H), 5.45 – 5.34 (m, 2H), 5.20 (d, *J* = 9.9 Hz, 1H), 5.02 – 4.91 (m, 1H), 4.41 (d, *J* = 5.5 Hz, 1H), 4.04 – 3.91 (m, 2H), 3.81 (s, 1H), 3.64 (t, *J* = 9.0 Hz, 1H), 2.80 (dh, *J* = 13.0, 6.3 Hz, 1H), 2.56 – 2.43 (m, 2H), 2.10 (dd, *J* = 13.8, 5.6 Hz, 1H), 1.90 (dd, *J* = 13.8, 8.7 Hz, 1H), 1.78 (s, 3H), 1.69 (s, 3H), 1.68 (d, *J* = 6.5 Hz, 3H), 1.67 – 1.64 (m, 1H), 1.57 (d, *J* = 14.0 Hz, 1H), 1.49 (t, *J* = 11.8 Hz, 1H), 0.96 (d, *J* = 6.5 Hz, 3H), 0.86 (d, *J* = 7.1 Hz, 3H). **^{13}C-NMR** (126MHz, $CDCl_3$) δ [ppm]: 165.16, 145.67, 141.69, 139.23, 135.04, 132.47, 129.78, 129.55, 127.42, 124.94, 124.78, 121.42, 120.45, 79.55, 75.20, 71.61, 69.48, 59.36, 47.40, 39.25, 31.49, 30.22, 29.95, 21.44, 20.44, 18.07, 17.99, 11.16. **LRMS** (ESI-Quad) [m/z]: 456.3 $[M+H]^+$, **HRMS** (ESI-IT) [m/z]: 456.310562, calculated 456.310835 for $C_{28}H_{42}NO_4$ $[M+H]^+$, err [ppm] 0.598.

Compound 18: **^{1}H-NMR** (500 MHz, $CDCl_3$) δ [ppm]: 8.35 – 8.08 (m, 2H), 7.96 – 7.72 (m, 2H), 6.55 (dd, *J* = 15.0, 11.2 Hz, 1H), 6.21 (d, *J* = 15.3 Hz, 1H), 5.67 (dd, *J* = 15.3, 6.6 Hz, 1H), 5.57 (d, *J* = 11.0 Hz, 1H), 5.46 (ddd, *J* = 23.5, 15.2, 8.4 Hz, 2H), 5.38 (dd, *J* = 15.4, 4.5 Hz, 1H), 5.05 (d, *J* = 9.5 Hz, 1H), 4.38 (d, *J* = 4.6 Hz, 1H), 4.26 (t, *J* = 10.0 Hz, 1H), 4.12 (d, *J* = 11.0 Hz, 1H), 3.95 (s, 1H), 3.77 (s, 2H), 3.71 (s, 3H), 2.87 – 2.78 (m, 1H), 2.69 (dd, *J* = 17.5, 5.0 Hz, 1H), 2.67 – 2.57 (m, 2H), 2.04 (d, *J* = 11.6 Hz, 1H), 2.02 – 1.93 (m, 1H), 1.87 – 1.79 (m, 1H), 1.78 (s, 3H), 1.74 (s, 3H), 1.69 (d, *J* = 6.1 Hz, 3H), 1.73 – 1.60 (m, 2H), 1.41 (s, 1H), 1.04 (d, *J* = 6.5 Hz, 3H), 0.83 (d, *J* = 7.1 Hz, 3H). **^{13}C-NMR** (126 MHz, $CDCl_3$) δ [ppm]: 207.14, 171.48, 162.84, 162.47, 162.23, 161.97, 140.63, 137.42, 135.75, 130.88, 129.59, 129.44, 127.16, 125.32, 119.95, 74.90, 73.15, 71.35, 69.62, 62.95, 57.40, 52.49, 47.99, 39.08, 37.08, 31.24, 31.09, 29.85, 29.21, 22.65, 20.57, 17.98, 17.66, 11.16. **LRMS** (ESI-Quad) [m/z]: 505.4 $[M+H]^+$, **HRMS** (ESI-IT) [m/z]: 505.363758, calculated 505.363599 for $C_{28}H_{42}NO_4$ $[M+H]^+$, err [ppm] -0.315.

Compound 19: **^{1}H-NMR** (700 MHz, $CDCl_3$) δ [ppm]: 8.44 – 8.17 (m, 2H), 8.15 – 7.85 (m, 2H), 6.56 (dd, *J* = 15.0, 11.0 Hz, 1H), 6.38 (d, *J* = 15.4 Hz, 1H), 5.85 (d, *J* = 10.9 Hz, 1H), 5.66 (ddd, *J* = 15.5, 6.5, 1.2 Hz, 1H), 5.52 (dd, *J* = 15.4, 8.0 Hz, 1H), 5.42 – 5.38 (m, 1H), 5.37 (dd, *J* = 14.9, 9.3 Hz, 1H), 5.08 (d, *J* = 10.1 Hz, 1H), 4.43 (d, *J* = 6.1 Hz, 1H), 4.26 (s, 1H), 3.99 – 3.91 (m, 2H), 3.86 (s, 1H), 3.72 (s, 3H), 3.59 (t, *J* = 9.4 Hz, 1H), 2.79 – 2.71 (m, 2H), 2.65 (dd, *J* = 17.4, 5.0 Hz, 1H), 2.30 (d, *J* = 10.4 Hz, 1H), 2.02 – 1.94 (m, 1H), 1.73 (d, *J* = 1.1 Hz, 3H), 1.69 (s, 1H), 1.68 (s, 3H), 1.66 – 1.65 (m, 2H), 1.63 (s, 3H), 1.57 (d, *J* = 14.1 Hz, 1H), 1.48 (ddd, *J* = 14.3, 12.0, 2.7 Hz, 1H), 1.00 (d, *J* = 6.4 Hz, 3H), 0.87 (d, *J* = 7.2 Hz, 3H). **^{13}C-NMR** (176MHz, $CDCl_3$) δ [ppm]: 207.11, 171.64, 162.76, 162.56, 162.35, 162.15, 143.94, 136.83, 135.91, 130.72, 130.21, 129.58, 129.26, 127.33, 124.62, 119.50, 75.04, 73.72, 71.63, 69.60, 52.54, 46.74, 39.47, 39.38, 36.96, 34.38, 31.09, 30.48, 29.86, 22.06, 20.38, 19.90, 17.97, 11.18. **LRMS** (ESI-Quad) [m/z]: 505.4 $[M+H]^+$, **HRMS** (ESI-IT) [m/z]: 505.363803, calculated 505.363599 for $C_{28}H_{42}NO_4$ $[M+H]^+$, err [ppm] -0.404.

Determination of the absolute stereo configuration at C16 for the 16-Aminoratjadones[4–6]

Synthesis of 16-*R*-Mosher - (R)-3,3,3-trifluoro-N-((1R,2E,4E,7R,8Z,10E)-1-((2S,4R,5S,6S)-4-hydroxy-5-methyl-6-((E)-prop-1-en-1-yl)tetrahydro-2H-pyran-2-yl)-5,7,9-trimethyl-11-((R)-6-oxo-3,6-dihydro-2H-pyran-2-yl)undeca-2,4,8,10-tetraen-1-yl)-2-methoxy-2-phenylpropanamide, **16-*S*-Mosher** - (S)-3,3,3-trifluoro-N-((1R,2E,4E,7R,8Z,10E)-1-((2S,4R,5S,6S)-4-hydroxy-5-methyl-6-((E)-prop-1-en-1-yl)tetrahydro-2H-pyran-2-yl)-5,7,9-trimethyl-11-((R)-6-oxo-3,6-dihydro-2H-pyran-2-yl)undeca-2,4,8,10-tetraen-1-yl)-2-methoxy-2-phenylpropanamide, **17-*R*-Mosher** - (R)-3,3,3-trifluoro-N-((1S,2E,4E,7R,8Z,10E)-1-((2S,4R,5S,6S)-4-hydroxy-5-methyl-6-((E)-prop-1-en-1-yl)tetrahydro-2H-pyran-2-yl)-5,7,9-trimethyl-11-((R)-6-oxo-3,6-dihydro-2H-pyran-2-yl)undeca-2,4,8,10-tetraen-1-yl)-2-methoxy-2-phenylpropanamide and **17-*S*-Mosher** (S)-3,3,3-trifluoro-N-((1S,2E,4E,7R,8Z,10E)-1-((2S,4R,5S,6S)-4-hydroxy-5-methyl-6-((E)-prop-1-en-1-yl)tetrahydro-2H-pyran-2-yl)-5,7,9-trimethyl-11-((R)-6-oxo-3,6-dihydro-2H-pyran-2-yl)undeca-2,4,8,10-tetraen-1-yl)-2-methoxy-2-phenylpropanamide

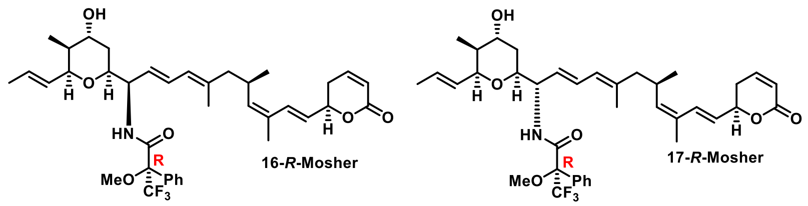

Chemical Formula: $C_{38}H_{48}F_3NO_6$
Molecular Weight: 671,80

Chemical Formula: $C_{38}H_{48}F_3NO_6$
Molecular Weight: 671,80

To a solution of 1.85 mg (7.896 µmol, 1.2 equiv) (R)-3,3,3-trifluoro-2-methoxy-2-phenylpropanoic acid in 10 µL dry CH_2Cl_2 were added a solution of 0.91 mg (7.896 µmol, 1.2 equiv) N-hydroxysuccinimide in 10 µL dry THF and a solution of 1.63 mg (7.896 µmol, 1.2 equiv) DCC in 10 µL dry CH_2Cl_2 and the mixture was stirred at 23°C for 15 h, before 4.4 µL (39.50 µmol, 6.0 equiv) NMM and a solution of 3 mg (6.58 µmol, 1.0 equiv) 16(*R*)-aminoratjadone **16** were added and the mixture was stirred for additional 30 h. The reaction mixture was diluted with 200 µL CH_2Cl_2 and directly purified by preparative thin-layer chromatography (CH_2Cl_2: MeOH/98:2, 1x Development), yielding 2.3 mg (3.42 µmol, 52%) of **16-*R*-Mosher** as a yellow solid foam.

In similar manner reaction in the presence of 16*S*-aminoratjadone **17** led to the formation of **17-*R*-Mosher** isolated in 52% (2.3 mg) as a yellow solid foam.

Both compounds **16-*R*-Mosher** and **17-*R*-Mosher** were slightly impured with DCU and traces of silicon grease.

16-*R*-Mosher: TLC (CH_2Cl_2:MeOH/98:2) R_f: 0.46 [UV^{254}, CAM], **IR** (ATR) [cm^{-1}]: 3421, 2961, 2924, 2854, 1722, 1693, 1625, 1506, 1451, 1380, 1260, 1164, 1080, 1056, 1015, 966, 918, 880, 802, 767, 735, 717, 698, 661. ^{19}F-NMR (471 MHz, $CDCl_3$) δ [ppm]: -68.80 (s, 3F). **^{1}H-NMR** (500 MHz, $CDCl_3$) δ [ppm]: 7.55 – 7.51 (m, 2H), 7.39 – 7.34 (m, 3H), 7.20 (d, *J* = 9.1 Hz, 1H), 6.94 – 6.84 (m, 1H), 6.71 (d, *J* = 15.6 Hz, 1H), 6.32 (ddd, *J* = 15.2, 10.9, 1.0 Hz, 1H), 6.06 (dt, *J* = 9.8, 1.8 Hz, 1H), 5.74 (d, *J* = 11.0 Hz, 1H), 5.70 (dd, *J* = 15.6, 6.5 Hz, 1H), 5.64 (ddd, *J* = 15.4, 6.5, 1.4 Hz, 1H), 5.57 (dd, *J* = 15.2, 7.6 Hz, 1H), 5.42 (ddd, *J* = 15.4, 5.7, 1.7 Hz, 1H), 5.22 (d, *J* = 9.1 Hz, 1H), 5.05 – 4.93 (m, 1H), 4.53 (td, *J* = 8.1, 3.7 Hz, 1H), 4.37 (d, *J* = 5.5 Hz, 1H), 4.02 (s, 1H), 3.98 (d, *J* = 2.5 Hz, 1H), 3.96 – 3.91 (m, 1H), 3.46 (d, *J* = 1.3 Hz, 3H), 2.80 (dq, *J* = 9.7, 7.0 Hz, 1H), 2.50 – 2.41 (m, 2H), 2.02 – 1.96 (m, 2H), 1.97 – 1.90 (m, 2H), 1.79 (d, *J* = 1.2 Hz, 3H), 1.71 (dt, *J* = 6.5, 1.4 Hz, 3H), 1.63 (d, *J* = 1.1 Hz, 3H), 1.60 – 1.57 (m, 1H), 0.92 (d, *J* = 6.6 Hz, 3H), 0.86 (d, *J* = 7.1 Hz, 3H). **^{13}C-NMR** (126 MHz, $CDCl_3$) δ [ppm]: 165.29, 164.20, 144.87, 139.34, 137.84, 133.22, 130.39, 130.27, 130.25, 129.52, 129.41, 128.56, 127.78, 126.42, 125.91, 125.80, 125.59, 121.77, 78.55, 74.68, 74.05, 70.31, 55.22, 54.67, 53.58, 49.35, 47.96, 39.59, 34.12, 25.77, 25.10, 21.02, 20.54, 18.10, 17.11, 11.28. **LRMS** (ESI-Quad) [m/z]: 672.9 $[M+H]^+$, **HRMS** (ESI-IT) [m/z]: 694.332582, calculated 694.332594 for $C_{38}H_{48}F_3NNaO_6$ $[M+Na]^+$, err [ppm] 0.017.

17-*R*-Mosher: TLC (CH_2Cl_2:MeOH/98:2) R_f: 0.41 [UV^{254}, CAM], **IR** (ATR) [cm^{-1}]: 3418, 2961, 2925, 2854, 1721, 1696, 1625, 1506, 1451, 1360, 1260, 1164, 1081, 1054, 1017, 965, 918, 877, 802, 767, 734, 718, 698, 668. ^{19}F-NMR (471 MHz, $CDCl_3$) δ [ppm]: -68.95 (s, 3F). **^{1}H-NMR** (500 MHz, $CDCl_3$) δ [ppm]: 7.60 (dd, *J* = 6.4, 3.0 Hz, 2H), 7.40 – 7.35 (m, 3H), 7.04 (d, *J* = 9.0 Hz, 1H), 6.90 (ddd, *J* = 9.8, 4.5, 3.9 Hz, 1H), 6.67 (d, *J* = 15.6 Hz, 1H), 6.43 (ddd, *J* = 15.2, 10.9, 1.2 Hz, 1H), 6.06 (dt, *J* = 9.8, 1.8 Hz, 1H), 5.74 (d, *J* = 10.8 Hz, 1H), 5.71 (dd, *J* = 15.8, 6.6 Hz, 1H), 5.61 (dd, *J* = 6.5, 1.3 Hz, 1H), 5.58 (dd, *J* = 6.5, 1.4 Hz, 1H), 5.36 (ddq, *J* = 15.3, 5.6, 1.4 Hz, 1H), 5.22 (d, *J* = 9.8 Hz, 1H), 5.04 – 4.95 (m, 1H), 4.42 (t, *J* = 8.0 Hz, 1H), 4.35 (d, *J* = 5.8 Hz, 1H), 4.02 (s, 1H), 3.91 (dt, *J* = 12.1, 2.7 Hz, 1H), 3.86 (d, *J* = 2.7 Hz, 1H), 3.47 (d, *J* = 1.3 Hz, 3H), 2.78 (dq, *J* = 9.7, 6.8 Hz, 1H), 2.47 (ddd, *J* = 7.1, 3.5, 1.6 Hz, 2H), 1.99 (d, *J* = 7.1 Hz, 2H), 1.93 (dt, *J* = 12.1, 3.8 Hz, 2H), 1.79 (d, *J* = 1.2 Hz, 3H), 1.70 (d, *J* = 1.2 Hz, 3H), 1.70 – 1.68 (m, 3H), 1.60 (dt, *J* = 13.1, 3.9 Hz, 1H), 0.93 (d, *J* = 6.6 Hz, 3H), 0.60 (d, *J* = 7.2 Hz, 3H). **^{13}C-NMR** (126 MHz, $CDCl_3$) δ [ppm]: 165.79, 164.15, 144.74, 139.22, 137.41, 133.05, 130.53, 130.17, 129.35, 129.32, 128.73, 128.40, 128.10, 127.52, 126.20, 125.87, 125.34, 121.61, 78.76, 77.18, 74.39, 73.91, 70.19, 55.09, 54.58, 53.41, 49.19, 47.79, 39.21, 33.96, 25.61, 24.94, 20.98, 20.36, 17.95, 16.97, 10.67. **LRMS** (ESI-Quad) [m/z]: 672.9 $[M+H]^+$, **HRMS** (ESI-IT) [m/z]: 694.332578, calculated 694.332594 for $C_{38}H_{48}F_3NNaO_6$ $[M+Na]^+$, err [ppm] 0.022.

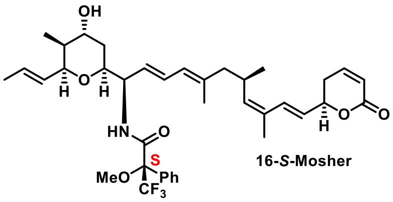

16-*S*-Mosher

Chemical Formula: $C_{38}H_{48}F_3NO_6$
Molecular Weight: 671,80

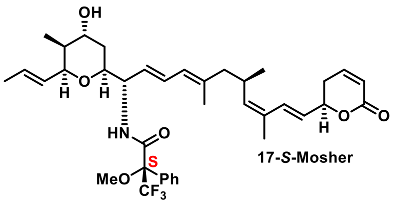

17-*S*-Mosher

Chemical Formula: $C_{38}H_{48}F_3NO_6$
Molecular Weight: 671,80

To a solution of 1.85 mg (7.896 µmol, 1.2 equiv) (S)-3,3,3-trifluoro-2-methoxy-2-phenylpropanoic acid in 10 µL dry CH_2Cl_2 were added a solution of 0.91 mg (7.896 µmol, 1.2 equiv) N-hydroxysuccinimide in 10 µL dry THF and a solution of 1.63 mg (7.896 µmol, 1.2 equiv) DCC in 10 µL dry CH_2Cl_2 and the mixture was stirred at 23°C for 15 h, before 4.4 µL (39.50 µmol, 6.0 equiv) NMM and a solution of 3 mg (6.58 µmol, 1.0 equiv) 16(*R*)-aminoratjadone **16** were added and the mixture was stirred for additional 30 h. The reaction mixture was diluted with 200 µL CH_2Cl_2 and directly purified by preparative thin-layer chromatography (CH_2Cl_2: MeOH/98:2, 1x Development), yielding 2.2 mg (3.27 µmol, 50%) of **16-*S*-Mosher** as a yellow solid foam.

In similar manner reaction in the presence of 16*S*-aminoratjadone **17** led to the formation of **17-*S*-Mosher** isolated in 52% (2.3 mg) as a yellow solid foam.

Both compounds **16-*R*-Mosher** and **16-*S*-Mosher** were slightly impured with DCU and traces of silicon grease.

16-*S*-Mosher: TLC (CH_2Cl_2:MeOH/98:2) R_f: 0.42 [UV^{254}, CAM], **IR** (ATR) [cm^{-1}]: 3417, 2960, 2925, 2854, 1721, 1693, 1552, 1511, 1451, 1380, 1335, 1321, 1260, 1102, 1061, 1056, 1015, 966, 918, 880, 866, 801, 767, 734, 717, 698, 668. ^{19}F-NMR (471 MHz, $CDCl_3$) δ [ppm]: -69.05 (s, 3F). **^{1}H-NMR** (500 MHz, $CDCl_3$) δ [ppm]: 7.56 (dd, *J* = 6.1, 3.2 Hz, 1H), 7.39 (tt, *J* = 3.5, 2.3 Hz, 1H), 6.91 (ddd, *J* = 9.8, 5.1, 3.3 Hz, 1H), 6.72 (d, *J* = 15.7 Hz, 1H), 6.44 (ddd, *J* = 15.1, 10.9, 1.1 Hz, 1H), 6.06 (ddd, *J* = 9.8, 2.2, 1.4 Hz, 1H), 5.78 (d, *J* = 10.8 Hz, 1H), 5.71 (dd, *J* = 15.2, 6.5 Hz, 1H), 5.62 (dd, *J* = 6.5, 1.5 Hz, 1H), 5.59 (dd, *J* = 6.5, 1.5 Hz, 1H), 5.39 (ddq, *J* = 15.5, 5.1, 1.6 Hz, 1H), 5.22 (d, *J* = 9.3 Hz, 1H), 5.02 (dt, *J* = 10.8, 5.4 Hz, 1H), 4.52 (td, *J* = 8.1, 4.0 Hz, 1H), 4.31 (d, *J* = 3.0 Hz, 1H), 4.02 (s, 1H), 3.96 (q, *J* = 2.6 Hz, 1H), 3.93 – 3.87 (m, 1H), 3.37 (d, *J* = 1.2 Hz, 3H), 2.81 (dq, *J* = 9.5, 6.9 Hz, 1H), 2.55 – 2.41 (m, 2H), 2.00 (d, *J* = 8.4 Hz, 2H), 1.94 (dd, *J* = 12.6, 3.5 Hz, 2H), 1.79 (d, *J* = 1.2 Hz, 3H), 1.71 (d, *J* = 1.1 Hz, 3H), 1.70 (dt, *J* = 6.5, 1.4 Hz, 3H), 1.64 – 1.60 (m, 1H), 0.93 (d, *J* = 6.6 Hz, 3H), 0.85 (d, *J* = 7.1 Hz, 3H). **^{13}C-NMR** (126 MHz, $CDCl_3$) δ [ppm]: 165.38, 164.24, 144.93, 139.29, 137.94, 132.68, 130.40, 130.30, 130.23, 129.52, 129.48, 128.65, 128.03, 126.22, 125.94, 125.61, 125.52, 121.75, 78.55, 74.46, 73.64, 70.29, 55.03, 54.92, 53.58, 49.35, 47.98, 39.51, 34.12, 25.77, 25.10, 21.11, 20.50, 18.10,

17.16, 11.26. **LRMS** (ESI-Quad) [m/z]: 672.9 $[M+H]^+$, **HRMS** (ESI-IT) [m/z]: 694.332531, calculated 694.332594 for $C_{38}H_{48}F_3NNaO_6$ $[M+Na]^+$, err [ppm] 0.090.

17-*S*-Mosher: TLC (CH_2Cl_2:MeOH/98:2) R_f: 0.42 [UV^{254}, CAM], **IR** (ATR) [cm^{-1}]: 3419, 3328, 2926, 2852, 1722, 1654, 1624, 1577, 1534, 1503, 1450, 1380, 1343, 1260, 1245, 1164, 1104, 1088, 1056, 1020, 966, 917, 905, 892, 814, 801, 767, 733, 713, 697, 662. ^{19}F-NMR (471 MHz, $CDCl_3$) δ [ppm]: -68.80 (s, 3F). **^{1}H-NMR** (500 MHz, $CDCl_3$) δ [ppm]: 7.56 – 7.50 (m, 2H), 7.40 – 7.33 (m, 3H), 7.22 (d, *J* = 8.8 Hz, 1H), 6.89 (dt, *J* = 9.8, 4.2 Hz, 1H), 6.68 (d, *J* = 15.6 Hz, 1H), 6.33 (ddd, *J* = 15.1, 10.9, 1.2 Hz, 1H), 6.05 (dt, *J* = 9.8, 1.8 Hz, 1H), 5.72 (d, *J* = 12.1 Hz, 1H), 5.71 (dd, *J* = 14.7, 6.1 Hz, 1H), 5.65 (ddd, *J* = 15.4, 6.5, 1.3 Hz, 1H), 5.57 (dd, *J* = 15.3, 7.1 Hz, 1H), 5.43 (ddq, *J* = 15.5, 6.1, 1.6 Hz, 1H), 5.22 (d, *J* = 9.5 Hz, 1H), 4.99 (q, *J* = 6.9 Hz, 1H), 4.48 – 4.41 (m, 1H), 4.41 (d, *J* = 6.1 Hz, 1H), 4.02 (s, 1H), 4.00 (s, 1H), 3.96 (dt, *J* = 12.2, 2.7 Hz, 1H), 3.45 (d, *J* = 1.3 Hz, 3H), 2.78 (dq, *J* = 9.9, 6.8 Hz, 1H), 2.49 – 2.43 (m, 2H), 1.98 (dd, *J* = 7.1, 4.4 Hz, 2H), 1.96 – 1.89 (m, 3H), 1.79 (d, *J* = 1.2 Hz, 3H), 1.70 (dt, *J* = 6.5, 1.3 Hz, 3H), 1.63 (d, *J* = 1.1 Hz, 3H), 0.92 (d, *J* = 9.0 Hz, 3H), 0.90 (d, *J* = 9.5 Hz, 3H). **^{13}C-NMR** (126 MHz, $CDCl_3$) δ [ppm]: 165.95, 164.28, 144.90, 139.39, 137.47, 132.91, 130.69, 130.32, 129.56, 129.48, 128.83, 128.62, 128.39, 127.89, 126.55, 126.00, 125.53, 121.77, 78.91, 74.79, 73.95, 70.45, 55.31, 54.86, 53.58, 49.35, 47.93, 39.53, 34.12, 25.77, 25.10, 21.06, 20.54, 18.08, 17.11, 11.07.

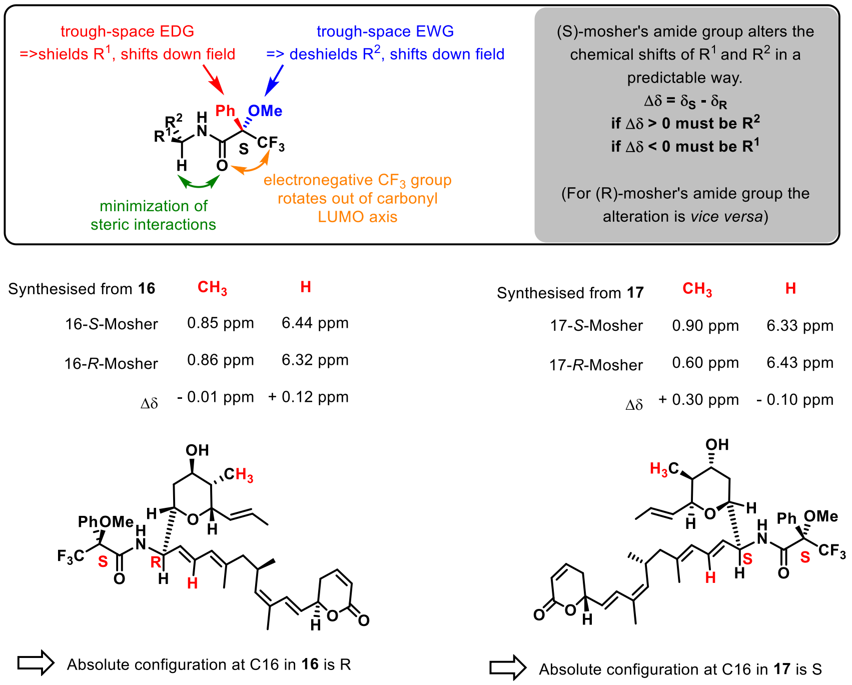

Synthesis of 16-Aminoratjadones with bearing terminal alkynes attached via short non-cleavable linkers

Synthesis of Pent-4-ynoic acid N-hydroxysuccinimid ester (23)[iii]

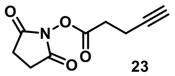

23

Chemical Formula: $C_9H_9NO_4$
Molecular Weight: 195,17

To a solution of 500 mg (5.096 mmol, 1.0 equiv) 3-Butynic acid and 587 mg (5.096 mmol, 1.0 equiv) N-hydroxysuccinimid in 64 mL of 1:1-mixture of dry EtOAc and dry dioxane was added 1.051 g (5.096 mmol, 1.0 equiv) DCC in one portion at 0°C. The mixture was stirred at 0°C for 5 h and additional 15 h at 23°C, before it was filtred and the filtrate was concentrated under reduced pressure. The residue was recrystallized from CH_2Cl_2/Pentane obtaining 800 mg (4.100 mmol, 80%) of **23** as a white amorph solid.

N-hydroxysuccinimid ester (23): ^{1}H-NMR (500 MHz, $CDCl_3$) δ [ppm]: 2.86 (dd, *J* = 8.2, 6.7 Hz, 2H), 2.82 (s, 4H), 2.60 (ddd, *J* = 8.6, 6.7, 2.7 Hz, 2H), 2.03 (t, *J* = 2.7 Hz, 1H). **^{13}C-NMR** (126 MHz, $CDCl_3$) δ [ppm] 169.27, 167.39, 81.23, 70.43, 30.70, 25.97, 14.49.

Synthesis of Compound 20 - N-((1R,2E,4E,7R,8Z,10E)-1-((2S,4R,5S,6S)-4-hydroxy-5-methyl-6-((E)-prop-1-en-1-yl)tetrahydro-2H-pyran-2-yl)-5,7,9-trimethyl-11-((R)-6-oxo-3,6-dihydro-2H-pyran-2-yl)undeca-2,4,8,10-tetraen-1-yl)pent-4-ynamide

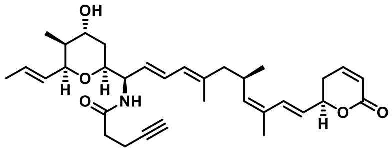

Chemical Formula: $C_{33}H_{45}NO_5$
Molecular Weight: 535,72500
20

To a solution of 9.5 mg (16.67 µmol, 1.0 equiv) 16*R*-aminoratjadone **16** in 167 µL dry CH_2Cl_2 were added 5.5 µL (50.03 µmol, 3.0 equiv) NMM and 3.6 mg (18.34 µmol, 1.1 equiv) 4-pentynoic acid N-hydroxysuccinimid ester[7] and the mixture was stirred for 1.5 h at 23°C. The mixture was diluted with 0.25 mL CH_2Cl_2 and directly purified by preparative thin-layer chromatography (CH_2Cl_2: MeOH/98:2, 1x Development), yielding 5.8 mg (10.82 µmol, 65%) of Compound **20** as a pale-yellow solid foam.

[iii] Synthesized according to a procedure of *Galibert et al*.[7]

Compound 20: TLC (CH_2Cl_2:MeOH/98:2) R_f: 0.12 [UV^{254}, CAM], **^{1}H-NMR** (700 MHz, $CDCl_3$) δ [ppm]: 6.91 (ddd, *J* = 9.7, 4.9, 3.6 Hz, 1H), 6.70 (d, *J* = 15.6 Hz, 1H), 6.41 (d, *J* = 11.8 Hz, 1H), 6.39 (dd, *J* = 10.4, 3.8 Hz, 1H), 6.07 (dt, *J* = 9.8, 1.7 Hz, 1H), 5.76 (d, *J* = 10.9 Hz, 1H), 5.70 (dd, *J* = 15.4, 6.7 Hz, 1H), 5.69 – 5.63 (m, 1H), 5.61 (dd, *J* = 15.2, 8.0 Hz, 1H), 5.43 (ddt, *J* = 13.8, 5.9, 1.7 Hz, 1H), 5.22 (d, *J* = 9.7 Hz, 1H), 5.04 – 4.94 (m, 1H), 4.43 (td, *J* = 8.3, 3.5 Hz, 1H), 4.37 – 4.26 (m, 1H), 3.96 (d, *J* = 2.7 Hz, 1H), 3.87 (dt, *J* = 12.4, 2.7 Hz, 1H), 2.80 (dq, *J* = 9.5, 6.9 Hz, 1H), 2.55 – 2.45 (m, 4H), 2.42 (t, *J* = 7.2 Hz, 2H), 1.99 (dd, *J* = 7.0, 2.0 Hz, 2H), 1.97 (t, *J* = 2.6 Hz, 1H), 1.78 (d, *J* = 1.1 Hz, 3H), 1.72 (dt, *J* = 6.7, 1.3 Hz, 3H), 1.71 – 1.70 (m, 3H), 1.66 – 1.60 (m, 2H), 1.38 (d, *J* = 14.2 Hz, 1H), 0.93 (d, *J* = 6.6 Hz, 3H), 0.86 (d, *J* = 7.1 Hz, 3H). **^{13}C-NMR** (176 MHz, $CDCl_3$) δ [ppm] 169.94, 164.26, 144.89, 139.46, 137.22, 130.88, 130.54, 130.02, 129.43, 126.74, 126.30, 126.18, 125.39, 121.77, 83.26, 78.79, 74.56, 74.28, 70.36, 69.43, 54.83, 47.88, 39.63, 35.63, 30.57, 30.54, 30.17, 21.15, 20.52, 18.06, 17.26, 15.07, 11.30. **LRMS** (ESI-Quad) [m/z]: 536.9 $[M+H]^+$, **HRMS** (ESI-IT) [m/z]: 558.318041, calculated 558.3189994 for $C_{33}H_{45}NNaO_5$ $[M+Na]^+$, err [ppm] 1.707.

Synthesis of But-3-yn-1-yl (4-nitrophenyl) carbonate (24)[iv]

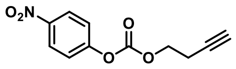

Chemical Formula: $C_{11}H_9NO_5$
Molecular Weight: 235,20

24

To a solution of 200 mg (2.853 mmol, 1.0 equiv) homoallylic alcohol in 71 mL dry CH_2Cl_2, 0.58 mL (7.134 mmol, 2.5 equiv) pyridine and 719 mg (3.567 mmol, 1.25 equiv) *para*-nitrophenylchloroformate were added and the mixture was stirred for 40 min at 23°C. The mixture was quenched by addition of 90 mL of saturated NH_4Cl solution and extracted with CH_2Cl_2 (3x 90 mL). The combined organic layers were dried over Na_2SO_4 and concentrated under reduced pressure. The residue was purified by flash-chromatographie through silicagel (Petroether:EtOAc/95:5 – 9:1) yielding 570 mg (2.423 mmol, 85%) of **24** as a white amorph solid.

But-3-yn-1-yl (4-nitrophenyl) carbonate (24): TLC (Petroether:EtOAc/9:1) R_f: 0.24 [UV^{254}], **^{1}H-NMR** (500 MHz, $CDCl_3$) δ [ppm]: 8.55 – 7.98 (m, 2H), 7.48 – 7.32 (m, 2H), 4.40 (t, *J* = 6.7 Hz, 2H), 2.68 (td, *J* = 6.7, 2.7 Hz, 2H), 2.08 (t, *J* = 2.7 Hz, 1H). **^{13}C-NMR** (126 MHz, $CDCl_3$) δ [ppm] 155.55, 152.39, 145.62, 125.48, 121.89, 79.06, 70.81, 66.81, 19.13.

[iv] Synthesis was based on a procedure of *Dommerholt et al.*.[17]

Synthesis of Compound 21 - But-3-yn-1-yl ((1R,2E,4E,7R,8Z,10E)-1-((2S,4R,5S,6S)-4-hydroxy-5-methyl-6-((E)-prop-1-en-1-yl)tetrahydro-2H-pyran-2-yl)-5,7,9-trimethyl-11-((R)-6-oxo-3,6-dihydro-2H-pyran-2-yl)undeca-2,4,8,10-tetraen-1-yl)carbamate

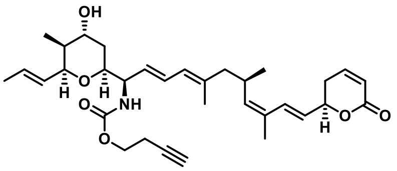

Chemical Formula: $C_{33}H_{45}NO_6$
Molecular Weight: 551,72400

21

To a solution of 4.0 mg (8.77 µmol, 1.0 equiv) 16*R*-aminoratjadone **16** in 88 µL dry CH_2Cl_2 were added 2.9 µL (26.34 µmol, 3.0 equiv) NMM and 2.3 mg (9.657 µmol, 1.1 equiv) but-3-yn-1-yl (4-nitrophenyl) carbonate **24** and the mixture was stirred for 24 h at 23°C. The mixture was diluted with 0.25 mL CH_2Cl_2 and directly purified by preparative thin-layer chromatography (CH_2Cl_2:MeOH/98:2, 1x Development), yielding 4.4 mg (7.90 µmol, 90%) of Compound **21** as a pale-yellow solid foam.

Compound 21: TLC (CH_2Cl_2:MeOH/98:2) R_f: 0.15 [UV^{254}, CAM], **IR** (ATR) [cm^{-1}]: 3442, 3309, 3034, 2963, 2923, 1718, 1653, 1605, 1566, 1497, 1437, 1361, 1335, 1291, 1246, 1136, 1081,. 1056, 1013, 967, 937, 918, 883, 816, 776, 722, 683, 651.**^{1}H-NMR** (500 MHz, $CDCl_3$) δ [ppm]: 6.94 – 6.87 (m, 1H), 6.72 (d, *J* = 15.6 Hz, 1H), 6.39 (dd, *J* = 15.1, 10.8 Hz, 1H), 6.07 (dt, *J* = 9.8, 1.8 Hz, 1H), 5.78 (d, *J* = 10.9 Hz, 1H), 5.76 – 5.67 (m, 1H), 5.66 (ddd, *J* = 15.4, 6.5, 1.4 Hz, 1H), 5.59 (dd, *J* = 15.1, 8.2 Hz, 1H), 5.49 – 5.36 (m, 2H), 5.23 (d, *J* = 9.6 Hz, 1H), 5.09 – 4.97 (m, 1H), 4.35 (d, *J* = 5.5 Hz, 1H), 4.16 (t, *J* = 6.7 Hz, 2H), 4.08 (s, 1H), 3.99 – 3.95 (m, 1H), 3.91 (dt, *J* = 12.3, 2.7 Hz, 1H), 3.49 (s, 1H), 2.88 – 2.70 (m, 1H), 2.55 – 2.45 (m, 4H), 2.10 – 1.94 (m, 3H), 1.79 (d, *J* = 1.2 Hz, 3H), 1.74 – 1.69 (m, 6H), 1.65 – 1.58 (m, 1H), 1.51 (s, 1H), 1.36 (d, *J* = 13.9 Hz, 1H), 0.92 (d, *J* = 6.6 Hz, 3H), 0.85 (d, *J* = 7.1 Hz, 3H). **^{13}C-NMR** (126 MHz, $CDCl_3$) δ [ppm] 164.06, 155.38, 144.70, 139.28, 137.31, 130.35, 130.20, 129.71, 129.20, 126.75, 126.27, 125.93, 125.37, 121.61, 78.50, 74.34, 70.15, 69.70, 62.52, 56.81, 47.78, 39.51, 30.37, 30.22, 30.00, 20.88, 20.36, 19.39, 17.88, 16.99, 11.14. **LRMS** (ESI-Quad) [m/z]: 552.4 $[M+H]^+$, **HRMS** (ESI-IT) [m/z]: 552.332090, calculated 552.331965 for $C_{33}H_{46}NO_6$ $[M+H]^+$, err [ppm] -0.228.

Synthesis of Compound 22 - But-3-yn-1-yl ((1S,2E,4E,7R,8Z,10E)-1-((2S,4R,5S,6S)-4-hydroxy-5-methyl-6-((E)-prop-1-en-1-yl)tetrahydro-2H-pyran-2-yl)-5,7,9-trimethyl-11-((R)-6-oxo-3,6-dihydro-2H-pyran-2-yl)undeca-2,4,8,10-tetraen-1-yl)carbamate

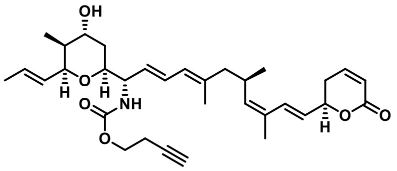

Chemical Formula: $C_{33}H_{45}NO_6$
Molecular Weight: 551,72

22

To a solution of 5.0 mg (10.97 µmol, 1.0 equiv) 16*S*-aminoratjadone **17** in 110 µL dry CH_2Cl_2 were added 7.2 µL (65.82 µmol, 6.0 equiv) NMM and 2.84 mg (12.07 µmol, 1.1 equiv) but-3-yn-1-yl (4-nitrophenyl) carbonate **24** and the mixture was stirred for 20 h at 23°C. The mixture was diluted with 0.25 mL CH_2Cl_2 and directly purified by preparative thin-layer chromatography (CH_2Cl_2:MeOH/98:2, 1x Development), yielding 4.4 mg (7.90 µmol, 90%) of Compound **22** as a pale-yellow solid foam.

Compound 22: TLC (CH_2Cl_2:MeOH/98:2) R_f: 0.16 [UV^{254}, CAM],^{1}**H-NMR** (500 MHz, $CDCl_3$) δ [ppm] 6.90 (dt, *J* = 9.7, 4.2 Hz, 1H), 6.67 (d, *J* = 15.6 Hz, 1H), 6.38 (dd, *J* = 14.7, 11.1 Hz, 1H), 6.10 – 6.00 (m, 1H), 5.74 (d, *J* = 11.0 Hz, 1H), 5.72 – 5.68 (m, 1H), 5.65 (dd, *J* = 14.8, 7.2 Hz, 1H), 5.59 (dd, *J* = 15.2, 7.5 Hz, 1H), 5.40 (dd, *J* = 15.4, 4.0 Hz, 1H), 5.25 (s, 1H) 5.22 (d, *J* = 9.6 Hz, 1H), 4.99 (q, *J* = 7.3 Hz, 1H), 4.39 (s, 1H), 4.23 – 4.12 (m, 2H), 4.05 (s, 1H), 4.02 – 3.96 (m, 1H), 3.88 (d, *J* = 12.0 Hz, 1H), 2.77 (dt, *J* = 13.7, 6.9 Hz, 1H), 2.52 (td, *J* = 6.7, 2.6 Hz, 2H), 2.49 – 2.44 (m, 2H), 2.04 – 1.94 (m, 3H), 1.87 – 1.80 (m, 1H), 1.79 (s, 3H), 1.73 – 1.68 (m, 6H), 1.67 – 1.59 (m, 1H), 1.45 (d, *J* = 14.2 Hz, 1H), 0.92 (d, *J* = 6.6 Hz, 3H), 0.88 (d, *J* = 7.1 Hz, 3H). ^{13}**C-NMR** (126 MHz, $CDCl_3$) δ [ppm] 164.31, 156.17, 144.89, 139.45, 137.17, 130.76, 130.34, 129.52, 129.47, 128.28, 126.32, 126.16, 125.47, 121.78, 80.60, 78.96, 74.53, 74.09, 70.47, 69.85, 62.70, 56.99, 47.97, 39.58, 30.63, 30.58, 30.23, 21.12, 20.52, 19.56, 18.07, 17.13, 11.36.**LRMS** (ESI-Quad) [m/z]: 552.4 $[M+H]^+$, **HRMS** (ESI-IT) [m/z]: 552.332174, calculated 552.331965 for $C_{33}H_{46}NO_6$ $[M+H]^+$, err [ppm] 0.038.

Synthesis of 16-Aminoratjadones bearing terminal alkynes and cyclooctynes attached via intracellular cleavable Val-Cit linker

Synthesis of Fmoc-Cit-*p*ABA – (9H-fluoren-9-yl)methyl (S)-(1-((4-(hydroxymethyl)phenyl)amino)-1-oxo-5-ureidopentan-2-yl)carbamate[8]

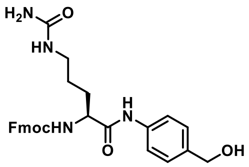

Chemical Formula: $C_{28}H_{30}N_4O_5$
Molecular Weight: 502,57
Fmoc-Cit-*p*ABA

To a solution of 1.0 g (2.516 mmol, 1.0 equiv) Fmoc-Cit-OH in 20 mL dry THF maintained under an argon atm. at -40°C were added 304 µL (2.768 mmol, 1.1 equiv) NMM and 358 µL (2.768 mmol, 1.1 equiv) *iso*-butylchloroformate. The mixture was stirred for 3 h at -40°C before further 332 µL (3.019 mmol, 1.2 equiv) NMM and 372 mg (3.019 mmol, 1.2 equiv) *para*-aminobenzylalcohol were added. The mixture was stirred for an additional 1 h at -40°C and then allowed to warm to 23°C over a period of 20 h. The reaction mixture was concentrated under reduced pressure and the solid residue was purified by flash chromatography through silicagel (CH_2Cl_2:MeOH/95:5 – 9:1 – 8:2) yielding 1.264 g (2.515 mmol, 100%) **Fmoc-Cit-*p*ABA** as an amorph pale-yellow solid.

Fmoc-Cit-*p*ABA:[v] **TLC** (CH_2Cl_2:MeOH/95:5) R_f: 0.10 [UV^{254}, CAM], **IR** (ATR) [cm^{-1}]: 3280, 3128, 3063, 2923, 2864, 1689, 1653, 1600, 1568, 1532, 1478, 1450, 1414, 1387, 1334, 1281, 12551, 1231, 1164, 1153, 1116, 1103, 1085, 1044, 1033, 1016, 989, 938, 823, 797, 778, 756, 737, 701, 674, 662, 640, 610. **^{1}H-NMR** (500 MHz, DMSO-d_6) δ [ppm]: 9.98 (s, 1H), 7.89 (d, *J* = 7.5 Hz, 2H), 7.75 (t, *J* = 6.5 Hz, 2H), 7.67 (d, *J* = 8.0 Hz, 1H), 7.56 (d, *J* = 8.4 Hz, 2H), 7.42 (t, *J* = 7.4 Hz, 2H), 7.33 (q, *J* = 6.8 Hz, 2H), 7.24 (d, *J* = 8.4 Hz, 2H), 5.99 (t, *J* = 5.5 Hz, 1H), 5.43 (s, 2H), 5.10 (t, *J* = 5.7 Hz, 1H), 4.43 (d, *J* = 5.6 Hz, 2H), 4.30 – 4.25 (m, 2H), 4.25 – 4.20 (m, 1H), 4.20 – 4.13 (m, 1H), 3.11 – 2.99 (m, 1H), 3.01 – 2.87 (m, 1H), 1.74 – 1.63 (m, 2H), 1.65 – 1.54 (m, 2H), 1.53 – 1.44 (m, 2H), 1.43 – 1.34 (m, 2H). **^{13}C-NMR** (126 MHz, DMSO-d_6) δ [ppm]: 170.98, 158.90, 156.10, 143.88, 143.79, 140.70, 137.57, 137.39, 127.64, 127.64, 127.08, 127.06, 126.91, 125.36, 120.10, 120.10, 118.85, 66.05, 65.67, 62.60, 55.03, 54.95, 46.66, 46.07, 29.34, 26.93. **LRMS** (ESI-Quad) [m/z]: 503.3[M+H]$^+$, **HRMS** (ESI-IT) [m/z]: 503.2294, calculated 503.2289 for $C_{28}H_{31}N_4O_5$ [M+H]$^+$, err [ppm] -1.00.

---

[v] The obtained analytic data were in completed accordance with prior published ones.[8]

Synthesis of H_2N-Val-Cit-*p*ABA – (S)-2-((S)-2-amino-3-methylbutanamido)-N-(4-(hydroxymethyl)-phenyl)-5-ureidopentanamide

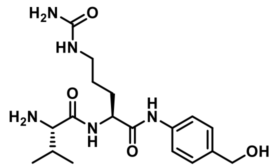

Chemical Formula: $C_{18}H_{29}N_5O_4$
Molecular Weight: 379,46
H_2N-Val-Cit-*p*ABA

To a solution of 100 mg (0.199 mmol, 1.0 equiv) **Fmoc-Cit-*p*ABA** in 3.9 mL dry DMF was added 318 µL (1.6 mL/mmol **Fmoc-Cit-*p*ABA**) piperidine and the mixture was stirred for 3 h at 23°C (TLC (CH_2Cl_2:MeOH/9:1) R_f: 0.05 [Nin]), before the mixture was diluted with 10 mL toluene, concentrated under reduced pressure and co-evaporated three times with 10 mL toluene. The raw solid **H_2N-Cit-PABOH** was re-dissolved in 900 µL dry DMF and added together with 142 µL (0.7956 mmol, 4.0 equiv) DiPEA to a solution of 101 mg (0.199 mmol, 1.0 equiv) Fmoc-Val-OPfp in 900 µL dry DMF at 23°C. The mixture was stirred for 24 h at 23°C (TLC (CH_2Cl_2:MeOH/8:2) R_f: 0.15 [UV^{254}, CAM]), before 318 µL (1.6 mL/mmol **Fmoc-Cit-*p*ABA**) piperidine was added and the mixture was stirred for 1 h at 23°C. The mixture was diluted with 10 mL toluene, concentrated under reduced pressure and co-evaporated three times with 10 mL toluene. The resulting residue was purified by flash chromatography through silicagel (CH_2Cl_2:MeOH/1:0 - 95:5 – 9:1 – 8:2) yielding 70 mg (0.185 mmol, 93%) **H_2N-Val-Cit-*p*ABA** as an amorph, pale-yellow solid.

H_2N-Val-Cit-*p*ABA: TLC (CH_2Cl_2:MeOH/8:2) R_f: 0.05 [UV^{254}, CAM], **^{1}H-NMR** (500 MHz, MeOD-*d_4*) δ [ppm]: 7.54 (d, *J* = 8.6 Hz, 2H), 7.30 (d, *J* = 8.7 Hz, 2H), 4.56 (s, 2H), 4.58 – 4.51 (m, 1H), 3.27 (d, *J* = 5.6 Hz, 1H), 3.21 (dt, *J* = 13.8, 7.0 Hz, 1H), 3.16 – 3.08 (m, 1H), 2.08 – 1.96 (m, 1H), 1.94 – 1.84 (m, 1H), 1.83 – 1.68 (m, 2H), 1.69 – 1.50 (m, 2H), 1.00 (d, *J* = 6.9 Hz, 3H), 0.94 (d, *J* = 6.8 Hz, 3H). **^{13}C-NMR** (126 MHz, MeOD-*d_4*) δ [ppm]: 175.88, 172.35, 162.33, 138.74, 138.65, 128.60, 121.20, 64.82, 61.19, 54.83, 33.19, 30.68, 27.86, 19.68, 17.78. **LRMS** (ESI-Quad) [m/z]: 380.3 $[M+H]^+$, **HRMS** (ESI-IT) [m/z]: 380.229231, calculated 380.229231 for $C_{18}H_{30}N_5O_4$ $[M+H]^+$, err [ppm] -0.071.

Synthesis of BCN-*p*NPC – ((1R,8S,9s)-bicyclo[6.1.0]non-4-yn-9-yl)methyl (4-nitrophenyl) carbonate

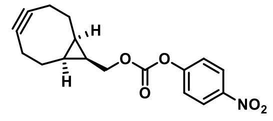

Chemical Formula: $C_{17}H_{17}NO_5$
Molecular Weight: 315,33
BCN-*p*NPC

To a solution of 100 mg (0.666 mmol, 1.0 equiv) ((1R,8S,9s)-bicyclo[6.1.0]non-4-yn-9-yl)methanol in 16.6 mL dry CH_2Cl_2 was added 134 µL (1.664 mmol, 2.5 equiv) pyridine and 168 mg (0.831 mmol, 1.25 equiv) 4-nitrophenyl chloroformiate and the mixture was stirred for 20 min at 23°C, before it was quenched by addition of 20 mL saturated ammonium chloride solution. The mixture was extracted with CH_2Cl_2 (3x 20 mL), the combined organic layers were dried over Na_2SO_4 and concentrated under reduced pressure. The residue was purified by flash chromatography through silicagel (PE:EtOAc/95:5 – 9:1) yielding 196 mg (0.6215 mmol, 93%) of **BNC-pNPC** as a highly viscous liquid, slowly solidifying giving an amorph white solid.

BCN-*p*NPC: TLC (PE:EtOAc/9:1) R_f: 0.50 [UV^{254}, CAM], **IR** (ATR) [cm^{-1}]: 2918, 2852, 1760, 1616, 1595, 1523, 1492, 1470, 1440, 1354, 1340, 1323, 1247, 1203, 1164, 1139, 1108, 1056, 1027, 1013, 989, 944, 921, 859, 817, 776, 733, 703, 670, 628. **^{1}H-NMR** (700 MHz, $CDCl_3$) δ [ppm]: 8.29 – 8.24 (m, 2H), 7.44 – 7.35 (m, 2H), 4.38 (d, *J* = 8.3 Hz, 2H), 2.36 – 2.26 (m, 4H), 2.25 – 2.19 (m, 2H), 1.64 – 1.54 (m, 2H), 1.49 (p, *J* = 8.6 Hz, 1H), 1.09 – 0.99 (m, 2H). **^{13}C-NMR** (176 MHz, $CDCl_3$) δ [ppm]: 155.73, 152.70, 145.49, 125.45, 121.91, 98.85, 68.16, 29.18, 21.50, 20.65, 17.38.

Synthesis of BCN-O(CO)HN-Val-Cit-*p*ABA – (S)-2-((S)-2-amino-3-methylbutanamido)-N-(4-(hydroxymethyl)phenyl)-5-ureidopentanamide

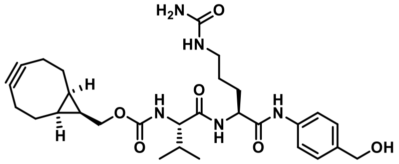

Chemical Formula: $C_{29}H_{41}N_5O_6$
Molecular Weight: 555,68
BCN-O(CO)HN-Val-Cit-*p*ABA **29**

To a solution of 100 mg (0.264 mmol, 1.0 equiv) **H_2N-Val-Cit-PABA** in 2.65 mL dry DMF was added 87 µL (0.791 mmol, 3.0 equiv) NMM and a solution of 87 mg (0.277 mmol, 1.05 equiv) **BCN-*p*NPC** in 2.65 mL dry DMF and the mixture was stirred for 24 h at 23°C, before the reaction was quenched by addition of 50 mL saturated ammonium chloride solution. The mixture was extracted with EtOAc (3x 20 mL). The combined

organic layers were dried over Na_2SO_4, concentrated under reduced pressure and co-evaportaed twice with 10 mL toluene. The resulting solid residue was purified by flash chromatography through silicagel (CH_2Cl_2:MeOH/95:5 – 9:1 – 8:2) yielding 132 mg (0.238 mmol, 90%) **BCN-O(CO)HN-Val-Cit-*p*ABA** as an amorph, white solid.

BCN-O(CO)HN-Val-Cit-*p*ABA: TLC (CH_2Cl_2:MeOH/9:1) R_f: 0.21 [UV^{254}, CAM], **IR** (ATR) [cm^{-1}]: 3444, 3271, 2925, 2853, 2604, 2468, 1694, 1637, 1516, 1439, 1417, 1381, 1335, 1301, 1238, 1172, 1139, 1120, 1090, 1027, 911, 864, 825, 770, 734, 696, 670, 582, 554. **^{1}H-NMR** (500 MHz, MeOD-*d₄*) δ [ppm]: 7.56 (d, *J* = 8.4 Hz, 2H), 7.30 (d, *J* = 8.7 Hz, 2H), 4.56 (s, 2H), 4.52 (dd, *J* = 8.9, 5.1 Hz, 1H), 4.17 (dd, *J* = 8.1, 1.5 Hz, 2H), 3.95 (d, *J* = 6.8 Hz, 1H), 3.20 (dt, *J* = 13.8, 7.0 Hz, 1H), 3.11 (dt, *J* = 13.5, 6.7 Hz, 1H), 2.33 – 2.19 (m, 3H), 2.19 – 2.10 (m, 2H), 2.10 – 2.03 (m, 1H), 1.92 (td, *J* = 14.3, 6.1 Hz, 1H), 1.75 (ddp, *J* = 14.1, 9.7, 4.9 Hz, 1H), 1.58 (dtd, *J* = 16.1, 10.9, 9.8, 6.8 Hz, 4H), 1.40 (p, *J* = 8.4 Hz, 1H), 0.99 (d, *J* = 6.8 Hz, 3H), 0.96 (d, *J* = 6.8 Hz, 2H). **^{13}C-NMR** (126 MHz, MeOD-*d₄*) δ [ppm]: 174.50, 172.14, 162.31, 159.17, 138.74, 128.57, 121.20, 120.41, 99.47, 64.83, 64.15, 62.30, 54.87, 40.30, 31.83, 30.50, 30.13, 27.84, 21.92, 21.42, 19.79, 18.92, 18.66. **LRMS** (ESI-Quad) [m/z]: 556.4 $[M+H]^+$, **HRMS** (ESI-IT) [m/z]: 556.313070, calculated 556.312961 for $C_{29}H_{42}N_5O_6$ $[M+H]^+$, err [ppm] -0.197.

Synthesis of BCN-O(CO)HN-Val-Cit-*p*AB-*p*NPC (27) – ((1R,8S,9s)-bicyclo[6.1.0]non-4-yn-9-yl)methyl ((S)-3-methyl-1-(((S)-1-((4-((((4-nitrophenoxy)carbonyl)oxy)methyl)phenyl)amino)-1-oxo-5-ureidopentan-2-yl)amino)-1-oxobutan-2-yl)carbamate

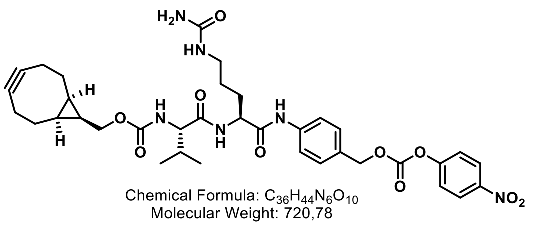

BCN-O(CO)HN-Val-Cit-*p*ABA-*p*NPC **27**

To a solution of 70 mg (0.126 mmol, 1.0 equiv) **BCN-O(CO)HN-Val-Cit-*p*ABA** in 420 µL dry DMF was added 32 µL (0.189 mmol, 1.5 equiv) DiPEA and 77 mg (0.252 mmol, 2.0 equiv) bis-4-nitrophenyl carbonate and the mixture was stirred for 16 h at 23°C. The reaction mixture was diluted with 300 µL CH_2Cl_2 and directly purified by flash chromatography through silicagel (CH_2Cl_2: MeOH/95:5), yielding 78.1 mg (0.108 mmol, 86%) of BCN-O(CO)HN-Val-Cit-PAB-pNPC **27** as an amorph, white solid.

BCN-O(CO)HN-Val-Cit-*p*AB-pNPC (27): TLC (CH_2Cl_2:MeOH/9:1) R_f: 0.62 [UV^{254}], **^{1}H-NMR** (500 MHz, MeOD-*d₄*) δ [ppm]: 8.33 – 8.26 (m, 2H), 7.68 – 7.60 (m, 2H), 7.50 – 7.43 (m, 2H), 7.43 (s, 2H), 5.26 (s, 2H), 4.54 (dt,

J = 9.1, 3.9 Hz, 1H), 4.17 (dd, *J* = 8.1, 6.2 Hz, 2H), 3.95 (d, *J* = 6.8 Hz, 1H), 3.20 (dq, *J* = 15.0, 8.1, 7.5 Hz, 1H), 3.11 (dt, *J* = 13.5, 6.6 Hz, 1H),), 2.29 – 2.00 (m, 7H), 1.98 – 1.86 (m, 0H), 1.75 (dqd, *J* = 14.2, 9.3, 4.9 Hz, 1H), 1.67 – 1.49 (m, 4H), 1.39 (p, *J* = 8.6 Hz, 1H), 0.99 (d, *J* = 6.8 Hz, 3H), 0.96 (d, *J* = 6.8 Hz, 3H), 0.94 – 0.89 (m, 1H). **^{13}C-NMR** (126 MHz, MeOD-*d_4*) δ [ppm]: 202.71, 200.46, 190.48, 187.35, 185.31, 182.15, 175.05, 168.38, 160.13, 158.69, 154.41, 151.45, 149.34, 127.64, 99.74, 92.31, 90.51, 83.05, 68.43, 59.97, 58.59, 58.31, 58.28, 56.02, 50.08, 49.57, 47.95, 47.07, 46.85. **LRMS** (ESI-Quad) [m/z]: 721.4 $[M+H]^+$, **HRMS** (ESI-IT) [m/z]: 721.3198819, calculated 721.319168 for $C_{36}H_{45}N_6O_{10}$ $[M+H]^+$, err [ppm] -0.903.

Synthesis of BCN-O(CO)HN-Val-Cit-*p*ABO(CO)-16*R*-Aminoratjadone(25) – ((1R,8S,9s)-bicyclo[6.1.0]non-4-yn-9-yl)methyl ((S)-1-(((S)-1-((4-(((((1R,2E,4E,7R,8Z,10E)-1-((2S,4R,5S,6S)-4-hydroxy-5-methyl-6-((E)-prop-1-en-1-yl)tetrahydro-2H-pyran-2-yl)-5,7,9-trimethyl-11-((R)-6-oxo-3,6-dihydro-2H-pyran-2-yl)undeca-2,4,8,10-tetraen-1-yl)carbamoyl)oxy)methyl)phenyl)amino)-1-oxo-5-ureidopentan-2-yl)amino)-3-methyl-1-oxobutan-2-yl)carbamate

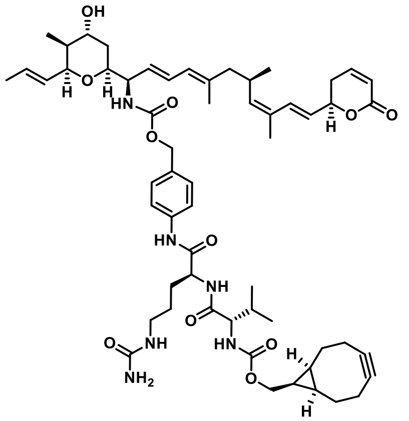

Chemical Formula: $C_{58}H_{80}N_6O_{11}$
Molecular Weight: 1037,31
BCN-O(CO)HN-Val-Cit-*p*ABO(CO)-16*R*-Aminoratjadone **25**

To a solution of 16.2 mg (35.48 µmol, 1.0 equiv) 16*R*-aminoratjadone **16** in 237 µL dry DMF were added 23.4 µL (0.212 mmol, 6.0 equiv) NMM and 28.1 mg (39.03 µmol, 1.1 equiv) BCN-O(CO)HN-Val-Cit-PAB-pNPC **27** and the mixture was stirred for 26 h at 23°C. The reaction mixture was diluted with 1.0 mL CH2Cl2 and directly purified by preparative thin-layer chromatography (CH_2Cl_2:MeOH/95:5, 3x Development), yielding 28.0 mg (26.99 µmol, 76%) of BCN-O(CO)HN-Val-Cit-*p*ABO(CO)-16*R*-Aminoratjadone**25** as an amorph, white solid.

BCN-O(CO)HN-Val-Cit-*p*ABO(CO)-16*R*-Aminoratjadone(25): TLC (CH_2Cl_2:MeOH/95:5) R_f: 0.21 [UV^{254}, CAM], **IR** (ATR) [cm^{-1}]: 3316, 2963, 2927, 1697, 1657, 1609, 1517, 1451, 1415, 1381, 1311, 1248, 1141, 1057, 1018, 967, 912, 819, 777, 732, 656. **^{1}H-NMR** (700 MHz, $CDCl_3$) δ [ppm] 9.24 (s, 1H), 7.57 – 7.47 (m,

2H), 7.24 (d, *J* = 7.2 Hz, 2H), 6.91 (s, 1H), 6.71 (d, *J* = 15.4 Hz, 1H), 6.43 – 6.30 (m, 1H), 6.06 (d, *J* = 9.5 Hz, 1H), 5.77 (d, *J* = 10.8 Hz, 1H), 5.69 (dd, *J* = 15.7, 6.8 Hz, 1H), 5.67 – 5.55 (m, 3H), 5.39 (dd, *J* = 15.4, 4.6 Hz, 1H), 5.23 (d, *J* = 9.1 Hz, 1H), 5.01 (dq, *J* = 29.5, 13.4, 11.7 Hz, 3H), 4.69 (s, 1H), 4.33 (s, 1H), 4.20 – 4.12 (m, 1H), 4.08 (s, 2H), 3.92 (s, 1H), 3.88 (d, *J* = 11.7 Hz, 1H), 3.26 (s, 1H), 3.09 (s, 1H), 2.79 (s, 1H), 2.48 (d, *J* = 5.8 Hz, 2H), 2.30 – 2.13 (m, 7H), 2.13 – 2.06 (m, 1H), 2.03 – 1.94 (m, 2H), 1.92 – 1.83 (m, 1H), 1.78 (s, 3H), 1.72 – 1.58 (m, 10H), 1.51 (s, 4H), 1.40 – 1.27 (m, 3H), 0.95 (d, *J* = 5.9 Hz, 3H), 0.92 (d, *J* = 6.5 Hz, 9H), 0.83 (d, *J* = 7.1 Hz, 3H). **^{13}C-NMR** (176 MHz, $CDCl_3$) δ [ppm]: 172.47, 170.40, 164.57, 161.98, 161.76, 160.52, 157.27, 155.96, 145.30, 139.64, 137.87, 137.40, 132.91, 130.83, 130.44, 129.74, 129.35, 128.91, 127.00, 126.31, 126.11, 125.39, 121.60, 120.20, 120.10, 98.96, 78.96, 74.54, 70.18, 66.37, 63.57, 60.67, 57.03, 53.02, 48.02, 39.48, 31.24, 30.50, 30.38, 30.18, 29.14, 21.55, 21.10, 20.51, 20.31, 19.45, 18.10, 18.05, 17.78, 17.07, 11.31. **LRMS** (ESI-Quad) [m/z]: 1038.5 $[M+H]^+$, **HRMS** (ESI-IT) [m/z]: 1037.5958, calculated 1037.5958 for $C_{58}H_{80}N_6O_{11}$ $[M+H]^+$, err [ppm] 0.0.

Synthesis of Homopropargyl-O(CO)HN-Val-Cit-*p*ABA – But-3-yn-1-yl ((S)-1-(((S)-1-((4-(hydroxymethyl)phenyl)amino)-1-oxo-5-ureidopentan-2-yl)amino)-3-methyl-1-oxobutan-2-yl)carbamate

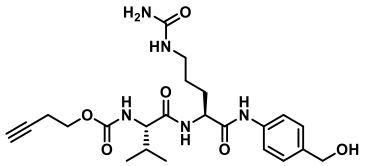

Chemical Formula: $C_{23}H_{33}N_5O_6$
Molecular Weight: 475,55
Homopropargyl-O(CO)HN-Val-Cit-*p*ABA

To a solution of 495 mg (1.304 mmol, 1.0 equiv) **H_2N-Val-Cit-*p*ABA** in 13 mL dry DMF was added 430 µL (3.913 mmol, 3.0 equiv) NMM and a solution of 310 mg (1.318 mmol, 1.01 equiv) but-3-yn-1-yl (4-nitrophenyl) carbonate **24** in 2.65 mL dry DMF and the mixture was stirred for 16 h at 23°C, before the reaction was quenched by addition of 220 mL saturated ammonium chloride solution. The mixture was extracted with EtOAc (3x 220 mL). The combined organic layers were dried over Na_2SO_4, concentrated under reduced pressure and co-evaportaed twice with 60 mL toluene. The resulting solid residue was purified by flash chromatography through silicagel (CH_2Cl_2:MeOH/95:5 – 8:2 – 1:1) yielding 409.2 mg (0.861 mmol, 66%) **Homopropargyl-O(CO)HN-Val-Cit-*p*ABA** as an amorph, white solid.

Homopropargyl-O(CO)HN-Val-Cit-*p*ABA: TLC (CH_2Cl_2:MeOH/9:1) R_f: 0.17 [UV^{254}, CAM], **IR** (ATR) [cm^{-1}]: 3267, 2960, 2925, 2871, 1690, 1639, 1602, 1535, 1465, 1445, 1415, 1386, 1339, 1295, 1248, 1184, 1136, 1117, 1094, 1075, 1035, 1015, 924, 823, 803, 774, 697, 661, 651, 606. **^{1}H-NMR** (500 MHz, DMSO-*d*$_6$) δ [ppm]: 9.96 (s, 1H), 8.06 (d, *J* = 7.6 Hz, 1H), 7.54 (d, *J* = 8.5 Hz, 2H), 7.23 (d, *J* = 8.6 Hz, 3H), 5.99 (t, *J* = 5.6

Hz, 2H), 5.41 (s, 2H), 5.09 (t, *J* = 5.7 Hz, 1H), 4.42 (d, *J* = 5.6 Hz, 3H), 4.43 – 4.36 (m, 1H), 4.08 – 3.96 (m, 3H), 3.89 (dd, *J* = 8.6, 7.0 Hz, 1H), 3.01 (dt, *J* = 13.0, 6.5 Hz, 1H), 2.94 (dq, *J* = 13.0, 6.4 Hz, 1H), 2.86 (t, *J* = 2.6 Hz, 1H), 2.47 (dt, *J* = 6.6, 3.4 Hz, 3H), 1.97 (dq, *J* = 13.5, 6.7 Hz, 1H), 1.77 – 1.63 (m, 1H), 1.58 (dtd, *J* = 13.5, 9.5, 4.8 Hz, 2H), 1.51 – 1.27 (m, 4H), 0.87 (d, *J* = 6.8 Hz, 3H), 0.83 (d, *J* = 6.8 Hz, 3H). **^{13}C-NMR** (126 MHz, MeOD-d_4) δ [ppm]: 171.13, 170.38, 158.85, 155.94, 137.50, 137.43, 126.92, 118.84, 81.16, 72.48, 62.58, 62.10, 60.07, 53.04, 39.52, 39.35, 39.19, 39.02, 38.57, 30.38, 29.50, 26.77, 19.19, 18.78, 18.18. **LRMS** (ESI-Quad) [m/z]: 476.3 [M+H]$^+$, **HRMS** (ESI-IT) [m/z]: 476.250842, calculated 476.250360 for $C_{23}H_{34}N_5O_6$ [M+H]$^+$, err [ppm] -1.012

Synthesis of Homopropargyl-O(CO)HN-Val-Cit-*p*ABA-*p*NPC (28) – But-3-yn-1-yl ((S)-3-methyl-1-(((S)-1-((4-((((4-nitrophenoxy)carbonyl)oxy)methyl)phenyl)amino)-1-oxo-5-ureidopentan-2-yl)amino)-1-oxobutan-2-yl)carbamate

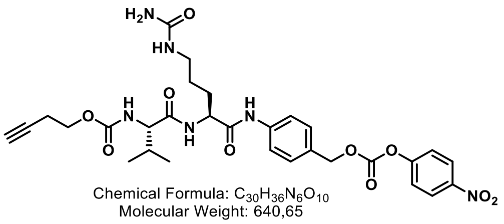

Homopropargyl-O(CO)HN-Val-Cit-*p*ABA-*p*NPC **28**

To a solution of 50 mg (0.105 mmol, 1.0 equiv) **Homopropargyl-O(CO)HN-Val-Cit-*p*ABA** in 350 µL dry DMF was added 27 µL (0.158 mmol, 1.5 equiv) DiPEA and 63.5 mg (0.210, 2.0 equiv) bis-4-nitrophenyl carbonate and the mixture was stirred for 6 h at 23°C. The reaction mixture was diluted with 300 µL CH_2Cl_2 and directly purified by flash chromatography through silicagel (CH_2Cl_2: MeOH/95:5), yielding 43 mg (0.067 mmol, 64%) of Homopropargyl-O(CO)HN-Val-Cit-PAB-pNPC **28** as an amorph, pale-yellow solid.

Homopropargyl-O(CO)HN-Val-Cit-*p*ABA-*p*NPC (28): TLC (CH_2Cl_2:MeOH/95:5) R_f: 0.32 [UV^{254}], **^{1}H-NMR** (700 MHz, DMSO-d_6) δ [ppm]: 10.12 (s, 1H), 8.38 – 8.23 (m, 2H), 8.11 (dd, *J* = 15.3, 8.3 Hz, 1H), 7.64 (d, *J* = 8.5 Hz, 1H), 7.59 – 7.50 (m, 2H), 7.41 (d, *J* = 8.6 Hz, 1H), 7.24 (d, *J* = 8.8 Hz, 1H), 5.97 (d, *J* = 5.1 Hz, 1H), 5.41 (s, 2H), 5.24 (s, 1H), 4.48 – 4.35 (m, 1H), 4.02 (tdd, *J* = 10.5, 6.6, 3.9 Hz, 2H), 3.92 – 3.85 (m, 1H), 3.03 (dq, *J* = 13.0, 6.6 Hz, 1H), 2.95 (dq, *J* = 12.9, 6.3 Hz, 1H), 2.86 (t, *J* = 2.5 Hz, 1H), 2.30 – 2.09 (m, 1H), 1.97 (dq, *J* = 13.6, 6.8 Hz, 1H), 1.69 (dt, *J* = 15.3, 7.7 Hz, 1H), 1.58 (dd, *J* = 13.5, 4.8 Hz, 1H), 1.44 (ddd, *J* = 17.4, 12.6, 7.6 Hz, 2H), 1.35 (dd, *J* = 8.4, 4.7 Hz, 1H), 0.87 (d, *J* = 6.8 Hz, 3H), 0.83 (d, *J* = 6.7 Hz, 3H). **^{13}C-NMR** (176 MHz, DMSO-d_6) δ [ppm]: 171.17, 170.71, 158.84, 155.92, 155.26, 151.93, 145.15, 139.34, 129.47, 129.27, 126.16, 125.38, 122.60, 122.58, 119.00, 115.77, 98.93, 81.14, 72.46, 70.22, 67.60, 62.07, 60.00, 53.55,

53.10, 30.36, 29.36, 28.49, 26.77, 20.77, 19.91, 19.16, 18.76, 18.16, 18.06, 16.92, 16.72, 12.45. **LRMS** (ESI-Quad) [m/z]: 641.7 $[M+H]^+$.

Synthesis of Homopropargyl-O(CO)HN-Val-Cit-*p*ABO(CO)-16*R*-Aminoratjadone(26) – But-3-yn-1-yl ((S)-1-(((S)-1-((4-(((((1R,2E,4E,7R,8Z,10E)-1-((2S,4R,5S,6S)-4-hydroxy-5-methyl-6-((E)-prop-1-en-1-yl)tetrahydro-2H-pyran-2-yl)-5,7,9-trimethyl-11-((R)-6-oxo-3,6-dihydro-2H-pyran-2-yl)undeca-2,4,8,10-tetraen-1-yl)carbamoyl)-oxy)methyl)phenyl)amino)-1-oxo-5-ureidopentan-2-yl)amino)-3-methyl-1-oxobutan-2-yl)carbamate

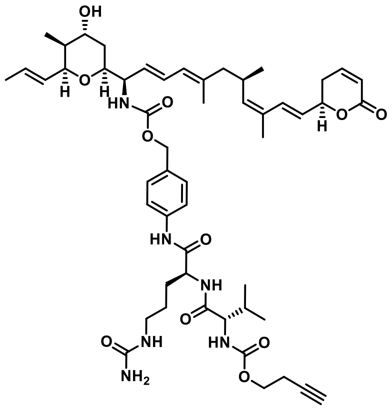

Chemical Formula: $C_{52}H_{72}N_6O_{11}$
Molecular Weight: 957,18
Homopropargyl-O(CO)HN-Val-Cit-pABO(CO)NH-Ratja **26**

To a solution of 7.0 mg (12.28 µmol, 1.0 equiv) 16*R*-aminoratjadone **16** in 123 µL dry DMF were added 8.1 µL (73.7 µmol, 6.0 equiv) NMM and 8.7 mg (13.51 µmol, 1.1 equiv) Homopropargyl-O(CO)HN-Val-Cit-pAB-pNPC **28** and the mixture was stirred for 30 h at 23°C. The reaction mixture was diluted with 1.0 mL CH_2Cl_2 and directly purified by preparative thin-layer chromatography (CH_2Cl_2:MeOH:EtOAc/9:1:1, 2x Development), yielding 7.8 mg (8.104 µmol, 66%) of Homopropargyl-O(CO)HN-Val-Cit-PABO(CO)-16R-Aminoratjadone**26** as an amorph, white solid.

Homopropargyl-O(CO)HN-Val-Cit-*p*ABO(CO)-16R-Aminoratjadone(26): TLC (CH_2Cl_2:MeOH/9:1) R_f: 0.49 [UV^{254}, CAM], **^{1}H-NMR** (700 MHz, MeOD-*d*^{4}) δ [ppm] 7.57 (dd, *J* = 8.8, 2.2 Hz, 2H), 7.36 – 7.25 (m, 2H), 7.02 (ddd, *J* = 9.8, 5.6, 2.8 Hz, 1H), 6.78 (d, *J* = 15.7 Hz, 1H), 6.37 (dd, *J* = 15.2, 10.8 Hz, 1H), 6.01 (ddd, *J* = 9.8, 2.5, 1.2 Hz, 1H), 5.77 (d, *J* = 10.9 Hz, 1H), 5.74 (dd, *J* = 15.6, 6.4 Hz, 1H), 5.67 (ddd, *J* = 15.4, 6.5, 1.5 Hz, 1H), 5.59 (dd, *J* = 15.2, 6.9 Hz, 1H), 5.42 (ddd, *J* = 15.4, 5.6, 1.7 Hz, 1H), 5.22 (d, *J* = 9.8 Hz, 1H), 5.10 – 5.06 (m, 1H), 5.05 (s, 2H), 4.51 (dd, *J* = 9.1, 5.2 Hz, 1H), 4.33 (ddt, *J* = 5.7, 2.8, 1.5 Hz, 1H), 4.18 – 4.08 (m, 3H), 3.96 (d, *J* = 6.8 Hz, 1H), 3.86 (q, *J* = 3.0 Hz, 1H), 3.78 (dd, *J* = 12.4, 5.5 Hz, 1H), 3.23 – 3.16 (m, 1H), 3.11 (dt, *J* = 13.4, 6.7 Hz, 1H), 2.89 (dt, *J* = 16.0, 6.9 Hz, 1H), 2.57 – 2.53 (m, 0H), 2.52 (td, *J* = 6.7, 2.5 Hz, 3H), 2.45 (ddt,

J = 18.5, 10.7, 2.7 Hz, 1H), 2.31 (t, *J* = 2.7 Hz, 1H), 2.11 – 2.03 (m, 2H), 1.99 (dd, *J* = 13.3, 8.1 Hz, 1H), 1.94 – 1.87 (m, 2H), 1.79 (d, *J* = 1.3 Hz, 2H), 1.78 – 1.74 (m, 1H), 1.71 (s, 2H), 1.69 (dt, *J* = 6.5, 1.5 Hz, 3H), 1.65 – 1.53 (m, 6H), 1.43 (d, *J* = 14.3 Hz, 1H), 0.98 (d, *J* = 6.8 Hz, 3H), 0.95 (d, *J* = 6.9 Hz, 3H), 0.94 (d, *J* = 6.6 Hz, 3H), 0.90 (t, *J* = 7.1 Hz, 0H), 0.84 (d, *J* = 7.2 Hz, 3H). **^{13}C-NMR** (176 MHz, MeOD-d^4) δ [ppm]: ^{13}C NMR (176 MHz, MeOD) δ 174.33, 172.24, 166.71, 162.31, 158.58, 158.30, 147.99, 139.98, 139.32, 138.01, 134.36, 131.81, 131.25, 130.90, 129.51, 128.92, 127.27, 127.08, 126.87, 126.24, 123.27, 121.51, 121.09, 81.09, 80.04, 75.72, 71.07, 70.91, 67.06, 64.21, 62.15, 58.17, 54.94, 40.39, 31.92, 31.56, 30.92, 30.74, 30.46, 30.09, 29.53, 27.84, 21.72, 21.42, 20.60, 19.98, 19.77, 18.59, 18.07, 17.05, 11.55. **LRMS** (ESI-Quad) [m/z]: 958.2 $[M+H]^+$, **HRMS** (ESI-IT) [m/z]: 957.532547, calculated 957.533184 for $C_{52}H_{72}N_6O_{11}$ $[M+H]^+$, err [ppm] 0.662.

Synthesis of 16-Aminoratjadones bearing terminal alkynes and cyclooctynes attached via intracellular cleavable disulfide linker

Synthesis of 2-PySS(CH$_2$)$_2$(CO)-16*R*-Aminoratjadone (31) – N-((1R,2E,4E,7R,8Z,10E)-1-((2S,4R,5S,6S)-4-Hydroxy-5-methyl-6-((E)-prop-1-en-1-yl)tetrahydro-2H-pyran-2-yl)-5,7,9-trimethyl-11-((R)-6-oxo-3,6-dihydro-2H-pyran-2-yl)undeca-2,4,8,10-tetraen-1-yl)-3-(pyridin-2-yldisulfaneyl)propanamide

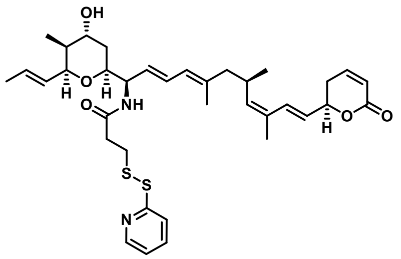

Chemical Formula: $C_{36}H_{48}N_2O_5S_2$
Molecular Weight: 652,91
2-PySS(CH$_2$)$_2$(CO)-16*R*-Aminoratjadone **31**

To a solution of 2.4 mg (4.215 µmol, 1.0 equiv) 16*R*-aminoratjadone **16** in 126 µL of a 1:2 mixture of MeCN and PBS (100 mM, pH = 7.45) was added 1.5 mg (4.64 µmol, 1.1 equiv) 3-(2-Pyridyldithio)propionic acid *N*-hydroxysuccinimide ester **32** and the mixture was stirred for 3.5 h at 23°C, before it was diluted with 1 mL H_2O and extracted with CH_2Cl_2 (3x 1.5 mL). The combined organic layers were dried over Na_2SO_4 and concentrated under reduced pressure. The residue was purified by preparative thin-layer chromatography (CH_2Cl_2:MeOH /95:5), yielding 2.7 mg (4.135 µmol, 98%) of 2-PySS(CH$_2$)$_2$(CO)-16*R*-Aminoratjadone **31** as an amorph, white solid.

2-PySS(CH$_2$)$_2$(CO)-16*R* -Aminoratjadone (31): TLC (CH_2Cl_2:MeOH/95:5) R_f: 0.22 [UV^{254}, CAM], **^{1}H-NMR** (500 MHz, benzene-*d$_6$*) δ [ppm] 8.33 (ddd, *J* = 4.8, 1.8, 0.9 Hz, 1H), 7.42 (d, *J* = 8.1 Hz, 1H), 6.98 (ddd, *J* = 8.1, 7.5, 1.9 Hz, 1H), 6.83 (d, *J* = 9.1 Hz, 1H), 6.72 (ddd, *J* = 15.2, 10.9, 1.0 Hz, 1H), 6.64 (d, *J* = 15.6 Hz, 1H), 6.48 (ddd, *J* = 7.4, 4.8, 1.0 Hz, 1H), 5.99 (dd, *J* = 15.2, 7.3 Hz, 1H), 5.94 – 5.88 (m, 2H), 5.80 (ddd, *J* = 9.8, 2.5, 1.0 Hz, 1H), 5.73 – 5.62 (m, 1H), 5.52 – 5.42 (m, 2H), 5.12 (d, *J* = 9.7 Hz, 1H), 4.90 (td, *J* = 8.0, 4.1 Hz, 1H), 4.56 (d, *J* = 5.8 Hz, 1H), 4.45 – 4.36 (m, 1H), 4.07 (ddd, *J* = 12.3, 3.9, 2.4 Hz, 1H), 3.57 (d, *J* = 2.7 Hz, 1H), 2.92 (t, *J* = 6.6 Hz, 2H), 2.84 – 2.71 (m, 2H), 2.27 (td, *J* = 6.6, 3.2 Hz, 2H), 1.98 – 1.85 (m, 2H), 1.68 (d, *J* = 1.1 Hz, 3H), 1.68 – 1.67 (m, 3H), 1.57 (dt, *J* = 6.5, 1.2 Hz, 3H), 1.56 – 1.53 (m, 1H), 1.52 – 1.48 (m, 2H), 1.26 (d, *J* = 14.1 Hz, 2H), 0.91 (d, *J* = 6.6 Hz, 3H), 0.82 (d, *J* = 7.2 Hz, 3H). **^{13}C-NMR** (126 MHz, benzene-*d$_6$*) δ [ppm]: 169.79, 163.67, 161.09, 150.27, 144.23, 139.44, 137.10, 136.84, 131.75, 130.88, 130.33, 129.96, 127.34, 126.61, 126.20, 122.12, 120.86, 120.07, 78.44, 75.42, 75.28, 70.47, 55.59, 48.34, 40.07, 36.19, 35.80, 31.38, 31.18,

30.57, 30.19, 21.77, 20.86, 18.35, 17.66, 11.76. **LRMS** (ESI-Quad) [m/z]: 653.9 [M+H]$^+$, **HRMS** (ESI-IT) [m/z]: 653.3077, calculated 653.3077 for $C_{36}H_{48}N_2O_5S_2$ [M+H]$^+$, err [ppm] 0.0.

Synthesis of 2-PySS(CH₂)₂NH₂ – 2-(pyridin-2-yldisulfaneyl)ethan-1-amine hydrochloride[vi]

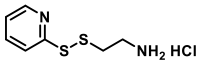

Chemical Formula: $C_7H_{11}ClN_2S_2$
Molecular Weight: 222,75
2-PySS(CH$_2$)$_2$NH$_2$

To a solution of 1.0 g (8.802 mmol, 1.0 equiv) 2-aminothioethanol hydrochloride in 5.3 mL of degassed dry MeOH under Argon atm. was added a solution of 5.0 g (22.695 mmol, 2.5 equiv) 2,2′-dipyridyl disulfide in 10.6 mL of degassed dry MeOH over a period of 1 h at 23°C using a syringe pump. The resulting mixture was stirred for 24 h at 23°C. Upon addition of ice-cold Et_2O (300 mL) to the mixture a white precipitate was formed, which was collected, washed with ice-cold Et_2O and dried in HV yielding 1.478 g (6.634 mmol, 75%) of **36** as a white solid.

2-PySS(CH$_2$)$_2$NH$_2$: ^{1}H-NMR (700 MHz, MeOD-*d$_4$*) 8.54 (ddd, *J* = 4.9, 1.8, 0.9 Hz, 1H), 7.95 – 7.72 (m, 1H), 7.65 (dt, *J* = 8.1, 0.9 Hz, 1H), 7.31 (ddd, *J* = 7.4, 4.9, 1.0 Hz, 1H), 3.30 (t, *J* = 6.2 Hz, 2H), 3.14 (t, *J* = 6.2 Hz, 2H). δ [ppm] **^{13}C-NMR** (176 MHz, MeOD-*d$_4$*) δ [ppm]: 159.48, 150.91, 139.14, 123.30, 122.86, 38.72, 36.85. **LRMS** (ESI-Quad) [m/z]: 187.3 [M+H]$^+$.

Synthesis of 2-PySS(CH$_2$)$_2$NH-(CO)(CH$_2$)$_2$CCH – N-(2-(pyridin-2-yldisulfaneyl)ethyl)pent-4-ynamide

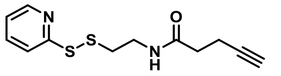

Chemical Formula: $C_{12}H_{14}N_2OS_2$
Molecular Weight: 266,38
2-PySS(CH$_2$)$_2$NH-(CO)(CH$_2$)$_2$CCH

To a solution of 300 mg (1.537 µmol, 1.0 equiv) 4-pentynoic acid N-hydroxysuccinimid ester[7] in 15.4 mL dry CH_2Cl_2 were added 803 µL (4.611 mmol, 3.0 equiv) DiPEA and 342 mg (1.537 mmol, 1.0 equiv) **2-PySS(CH$_2$)$_2$NH$_2$** and the mixture was stirred for 7 h at 23°C, before it was concentrated under reduced pressure and the resulting residue was purified by flash-chromatography through silicagel (EtOac: MeOH/30:1 – 15:1 – 10:1) yielding 271.4 mg (1.019 mmol, 66%) **2-PySS(CH$_2$)$_2$NH-(CO)(CH$_2$)$_2$CCH** as a pale yellow, amorph solid.

[vi] Synthesis was based on a procedure of *Vu et al.*[18]

2-PySS$(CH_2)_2$NH-(CO)$(CH_2)_2$CCH: TLC (EtOAc:MeOH/9:1) R_f: 0.77 [UV^{254}, CAM], **^{1}H-NMR** (500 MHz, $CDCl_3$) δ [ppm] 8.54 – 8.44 (m, 1H), 7.60 (td, *J* = 7.9, 1.8 Hz, 1H), 7.49 (dt, *J* = 8.1, 0.9 Hz, 1H), 7.13 (ddd, *J* = 7.3, 4.9, 0.8 Hz, 1H), 3.56 (q, *J* = 5.9 Hz, 2H), 2.95 – 2.86 (m, 2H), 2.53 (tdd, *J* = 7.2, 2.6, 0.9 Hz, 2H), 2.45 – 2.38 (m, 2H), 1.98 (t, *J* = 2.6 Hz, 1H). **^{13}C-NMR** (126 MHz, $CDCl_3$) δ [ppm]: 171.00, 159.25, 149.95, 137.12, 121.51, 121.36, 83.13, 69.42, 39.10, 37.40, 35.68, 15.07. **LRMS** (ESI-Quad) [m/z]: 267.4 $[M+H]^+$, **HRMS** (ESI-IT) [m/z]: 267.062375, calculated 267.062032 for $C_{12}H_{15}N_2OS_2$ $[M+H]^+$, err [ppm] -1.287.

Synthesis of $HO_2C(CH_2)_2SS(CH_2)_2$NH-(CO)$(CH_2)_2$CCH (30) – 3-((2-(pent-4-ynamido)ethyl)-disulfaneyl)propanoic acid

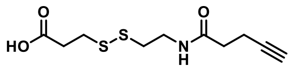

Chemical Formula: $C_{10}H_{15}NO_3S_2$
Molecular Weight: 261,35
$HO_2C(CH_2)_2SS(CH_2)_2$NH-(CO)$(CH_2)_2$CCH **30**

To a solution of 50 mg (0.1877 mmol, 1.0 equiv) **2-PySS$(CH_2)_2$NH-(CO)$(CH_2)_2$CCH** in 3.75 mL dry MeOH under Argon atm. was added 16.4 µL (0.1877 mmol, 1.0 equiv) 3-mercaptopropionic acid and the mixture was stirred for 19 h at 23°C. The solvent was removed under reduced pressure and the residue was dissolved in 400 µL MeOH and purified by preparative HPLC (Phenomenex Gemini C18 RP-column 00G-4435-PO-AX, 5 µm, 110 A, 250×21.20 mm (9 mL/min), ACN:H_2O + TFA/ 5:95 + 0.1% → 95:5 + 0.1% in 15 min) yielding after lyophilization 37.3 mg (0.1427 mmol, 76%) $HO_2C(CH_2)_2SS(CH_2)_2$NH-(CO)$(CH_2)_2$CCH **30** as a white, amorph solid.

$HO_2C(CH_2)_2SS(CH_2)_2$NH-(CO)$(CH_2)_2$CCH (30): TLC (EtOAc:MeOH/98:2 + 1% HOAc) R_f: 0.65 [CAM], **^{1}H-NMR** (500 MHz, MeOH-d_4) 3.49 (t, *J* = 6.7 Hz, 2H), 2.93 (t, *J* = 7.1 Hz, 2H), 2.82 (t, *J* = 6.7 Hz, 2H), 2.69 (t, *J* = 7.1 Hz, 2H), 2.49 – 2.44 (m, 2H), 2.41 – 2.36 (m, 2H), 2.26 (t, *J* = 2.6 Hz, 1H). **LRMS** (ESI-Quad) [m/z]: 262.4 $[M+H]^+$, **HRMS** (ESI-IT) [m/z]: 262.056032, calculated 262.056612 for $C_{10}H_{16}NO_3S_2$ $[M+H]^+$, err [ppm] 2.213.

Synthesis of HCC$(CH_2)_2$(CO)-NH-$(CH_2)_2SS(CH_2)_2$(CO)-16*R*-Aminoratjadone (29) – N-(2-((3-(((1R,2E,4E,7R,8Z,10E)-1-((2S,4R,5S,6S)-4-hydroxy-5-methyl-6-((E)-prop-1-en-1-yl)tetrahydro-2H-pyran-2-yl)-5,7,9-trimethyl-11-((R)-6-oxo-3,6-dihydro-2H-pyran-2-yl)undeca-2,4,8,10-tetraen-1-yl)amino)-3-oxopropyl)disulfaneyl)-ethyl)pent-4-ynamide

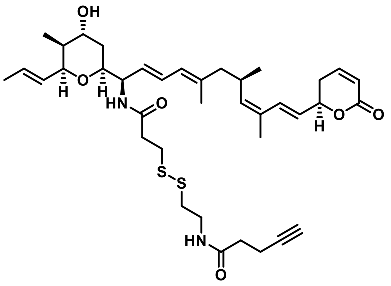

Chemical Formula: $C_{38}H_{54}N_2O_6S_2$
Molecular Weight: 698,98

$HCC(CH_2)_2(CO)$-NH-$(CH_2)_2SS(CH_2)_2(CO)$-16*R*-Aminoratjadone **29**

To a solution of 6.9 mg (26.346 µmol, 1.0 equiv) **$HO_2C(CH_2)_2SS(CH_2)_2NH$-(CO)$(CH_2)_2CCH$** in 131 µL dry DMF were added 14.5 µL (131.73 µmol, 5.0 equiv) NMM and 7.9 mg (26.346 µmol, 1.0 equiv) TSTU. The resulting mixture was stirred at 23°C for 30 min, before a solution of 12.0 mg (26.346 µmol, 1.0 equiv) 16*R*-aminoratjadone **16** in 131 µL dry DMF was added and the mixture was stirred for 16 h at 23°C. The mixture was poured into 20 mL H_2O and extracted with CH_2Cl_2 (3x 10 mL). The combined organic layers were dried over Na_2SO_4 and concentrated under reduced pressure. The residue was purified by preparative thin-layer chromatography (CH_2Cl_2:MeOH/98:2) yielding 12.0 mg (17.12 µmol, 65%) of **29** as a pale yellow, amorph solid.

$HCC(CH_2)_2(CO)$-NH-$(CH_2)_2SS(CH_2)_2(CO)$-16*R*-Aminoratjadone (29): TLC (CH_2Cl_2:MeOH/95:5) R_f: 0.69 [UV^{254}, CAM], **^{1}H-NMR** (700 MHz, benzene-*d*$_6$) δ [ppm] 6.90 (d, *J* = 9.0 Hz, 1H), 6.72 – 6.68 (m, 1H), 6.66 (d, *J* = 15.5 Hz, 1H), 6.42 (t, *J* = 5.9 Hz, 1H), 6.04 (dd, *J* = 15.2, 7.2 Hz, 1H), 5.95 (ddd, *J* = 9.8, 5.7, 2.7 Hz, 1H), 5.93 – 5.90 (m, 1H), 5.80 (ddd, *J* = 9.7, 2.6, 1.1 Hz, 1H), 5.77 (ddd, *J* = 15.4, 6.5, 1.5 Hz, 1H), 5.53 – 5.51 (m, 1H), 5.50 – 5.46 (m, 1H), 5.13 (dd, *J* = 9.9, 1.5 Hz, 1H), 4.90 (dddd, *J* = 8.8, 7.2, 4.5, 1.2 Hz, 1H), 4.64 (ddd, *J* = 5.7, 2.8, 1.5 Hz, 1H), 4.43 (dddd, *J* = 11.2, 7.2, 4.2, 1.1 Hz, 1H), 4.14 (ddd, *J* = 12.2, 4.7, 2.4 Hz, 1H), 3.75 (d, *J* = 2.9 Hz, 1H), 3.50 (dq, *J* = 13.9, 6.1 Hz, 1H), 3.46 – 3.40 (m, 1H), 2.95 – 2.85 (m, 2H), 2.82 (td, *J* = 9.1, 4.5 Hz, 1H), 2.72 – 2.63 (m, 2H), 2.53 – 2.44 (m, 3H), 2.41 – 2.31 (m, 2H), 2.27 – 2.22 (m, 2H), 1.98 (dd, *J* = 14.0, 5.5 Hz, 1H), 1.92 (dd, *J* = 14.0, 8.6 Hz, 1H), 1.86 (t, *J* = 2.7 Hz, 1H), 1.81 – 1.72 (m, 2H), 1.72 (d, *J* = 1.3 Hz, 3H), 1.71 (d, *J* = 1.3 Hz, 3H), 1.61 (dt, *J* = 6.5, 1.5 Hz, 3H), 1.58 – 1.54 (m, 2H), 1.52 – 1.48 (m, 1H), 0.93 (d, *J* = 6.6 Hz, 3H), 0.87 (d, *J* = 7.2 Hz, 3H). **^{13}C-NMR** (176 MHz, benzene-*d*$_6$) δ [ppm]: 171.08, 170.35, 163.75, 144.34, 139.48, 136.41, 131.59, 131.34, 129.93, 129.30, 128.69, 126.89, 126.04, 125.66, 121.63, 83.71, 78.54, 74.99, 74.95, 70.17, 69.60, 55.47, 47.93, 39.75, 38.90, 38.72, 36.61, 35.32, 35.07, 31.17, 30.84, 29.79, 21.59, 20.53, 18.06, 17.41, 15.18, 11.49. **LRMS** (ESI-Quad) [m/z]: 699.9 $[M+H]^+$, **HRMS** (ESI-IT) [m/z]: 699.3490, calculated 699.3496 for $C_{38}H_{54}N_2O_6S_2$ $[M+H]^+$, err [ppm] -0.857.

Synthesis of 19-Aminoratjadones with bearing terminal alkynes attached via short non-cleavable linkers

Synthesis of 19-Aminoratjadone (33) - (6R)-6-((1E,3Z,5R,7E,9E,11R)-11-((2S,5S,6S)-4-amino-5-methyl-6-((E)-prop-1-en-1-yl)tetrahydro-2H-pyran-2-yl)-11-hydroxy-3,5,7-trimethylundeca-1,3,7,9-tetraen-1-yl)-5,6-dihydro-2H-pyran-2-one

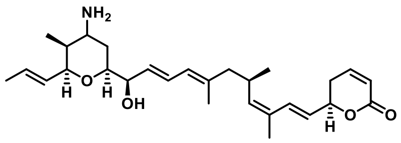

Chemical Formula: $C_{28}H_{41}NO_4$
Molecular Weight: 455,64
19-Aminoratjadone **33**

2.9 mg (39.595 µmol, 2.0 equiv) ammonium acetate were added to a solution of 9.0 mg (19.797 µmol, 1.0 equiv) 19-oxoratjadone **14** in 396 µL dry MeOH at 23°C and stirred for 15 min, before 2.5 mg (39.595 µmol, 2.0 equiv) sodium cyanoborohydride was added and the mixture was stirred at 23°C for 4 h, before the reaction was quenched by addition of 200 µL of ACN:H_2O + TFA/30:70 + 0.05%. This mixture was directly purified by RP prep HPLC (Phenomenex Gemini C18 RP-column 00G-4435-PO-AX, 5 µm, 110 A, 250×21.20 mm, Flow: 9 mL/min, ACN:H_2O + TFA/ 10:90 + 0.1% → 95:5 + 0.1% in 90 min) yielding after lyophilization 6.9 mg (15.1 µmol, 68%) 19-aminoratjadone **33** as an inseparable 1:2 mixture of 2 diastereoisomers as a pale-yellow solid foam.

19-Aminoratjadone (33): ^{1}**H-NMR** (700 MHz, $CDCl_3$) δ [ppm]: 8.21 (s, 1H), 8.04 (s, 2H), 6.95 – 6.87 (m, 1H), 6.70 – 6.54 (m, 1H), 6.48 – 6.39 (m, 1H), 6.05 – 5.97 (m, 1H), 5.77 – 5.62 (m, 3H), 5.54 – 5.29 (m, 2H), 5.19 (dd, *J* = 20.5, 9.7 Hz, 1H), 4.99 – 4.90 (m, 1H), 4.38 – 4.12 (m, 1H), 3.95 (dd, *J* = 8.0, 2.5 Hz, 1H), 3.48 (s, 1H), 3.46 – 3.39 (m, 1H), 2.82 – 2.69 (m, 1H), 2.51 – 2.40 (m, 4H), 2.09 – 1.84 (m, 3H), 1.77 (dd, *J* = 3.5, 1.0 Hz, 3H), 1.72 – 1.68 (m, 6H), 1.68 – 1.65 (m, 2H), 0.99 – 0.89 (m, 6H). ^{13}**C-NMR** (176 MHz, $CDCl_3$) δ [ppm]: 165.49, 165.10, 165.03, 165.00, 162.32, 162.12, 146.02, 145.72, 145.58, 139.72, 139.67, 139.60, 139.40, 138.13, 137.76, 137.70, 137.49, 131.82, 131.71, 131.57, 131.52, 131.08, 129.78, 129.57, 129.54, 129.50, 129.48, 129.32, 129.27, 129.16, 128.85, 128.76, 128.68, 128.15, 128.06, 127.94, 127.67, 127.52, 125.90, 125.87, 125.69, 125.06, 124.97, 121.51, 121.43, 121.32, 83.16, 80.01, 79.66, 79.52, 79.48, 79.45, 78.54, 78.09, 77.97, 74.41, 74.37, 74.24, 74.09, 74.04, 73.74, 73.33, 54.45, 53.57, 52.11, 51.70, 50.95, 47.72, 47.68, 47.64, 47.58, 39.41, 36.77, 35.62, 35.37, 30.99, 30.94, 30.85, 30.41, 30.26, 30.18, 29.99, 29.63, 24.08, 23.90, 21.65, 21.45, 20.48, 20.45, 18.02, 17.98, 17.62, 17.60, 13.37, 13.28, 11.99, 5.43.**LRMS** (ESI-Quad) [m/z]: 456.3 $[M+H]^+$, **HRMS** (ESI-IT) [m/z]: 456.310615, calculated 456.310835 for $C_{28}H_{42}NO_4$ $[M+H]^+$, err [ppm] – 0.043.

Synthesis of Compound 34 - But-3-yn-1-yl ((2S,3S,6S)-6-((1R,2E,4E,7R,8Z,10E)-1-hydroxy-5,7,9-trimethyl-11-((R)-6-oxo-3,6-dihydro-2H-pyran-2-yl)undeca-2,4,8,10-tetraen-1-yl)-3-methyl-2-((E)-prop-1-en-1-yl)tetrahydro-2H-pyran-4-yl)carbamate

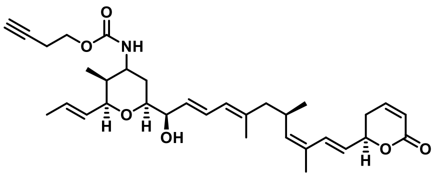

Chemical Formula: $C_{33}H_{45}NO_6$
Molecular Weight: 551,72
34

To a solution of 4.0 mg (7.02 µmol, 1.0 equiv) 19-aminoratjadone **33** in 70 µL dry CH_2Cl_2 were added 2.31 µL (21.06 µmol, 6.0 equiv) NMM and 1.82 mg (7.72 µmol, 1.1 equiv) but-3-yn-1-yl (4-nitrophenyl) carbonate **24** and the mixture was stirred for 36 h at 23°C. The mixture was diluted with 0.25 mL CH_2Cl_2 and directly purified by preparative thin-layer chromatography (CH_2Cl_2:MeOH/98:2, 1x Development), yielding 2.4 mg (4.35 µmol, 65%) of Compound **34** as a mixture of C-19 diastereomers as a pale-yellow solid foam.

Compound 34: TLC (CH_2Cl_2:MeOH/98:2) R_f: 0.21 [UV^{254}, CAM],^{1}**H-NMR** (700 MHz, $CDCl_3$) δ [ppm] 6.95 – 6.83 (m, 1H), 6.71 (t, *J* = 15.3 Hz, 1H), 6.47 (ddd, *J* = 15.2, 11.0, 1.5 Hz, 1H), 6.07 (ddt, *J* = 9.7, 3.6, 1.9 Hz, 1H), 5.81 – 5.74 (m, 1H), 5.73 – 5.65 (m, 2H), 5.52 (ddd, *J* = 15.5, 10.6, 6.6 Hz, 1H), 5.48 – 5.39 (m, 1H), 5.23 (d, *J* = 9.7 Hz, 1H), 5.02 – 4.97 (m, 1H), 4.31 (d, *J* = 29.2 Hz, 1H), 4.22 – 3.98 (m, 3H), 3.94 – 3.73 (m, 1H), 3.64 – 3.49 (m, 1H), 2.81 (dq, *J* = 14.2, 7.0 Hz, 1H), 2.54 – 2.46 (m, 4H), 2.41 – 2.29 (m, 1H), 2.03 – 1.98 (m, 3H), 1.79 (s, 3H), 1.72 (d, *J* = 2.4 Hz, 3H), 1.71 (d, *J* = 6.5 Hz, 3H), 1.54 – 1.49 (m, 2H), 0.93 (dd, *J* = 6.6, 2.3 Hz, 3H), 0.81 (d, *J* = 7.0 Hz, 3H). ^{13}**C-NMR** (176 MHz, $CDCl_3$) δ [ppm] 164.24, 164.19, 155.35, 144.84, 144.82, 139.43, 138.01, 137.85, 130.95, 130.78, 129.82, 129.71, 129.46, 128.76, 128.14, 128.07, 127.28, 126.00, 125.91, 125.49, 125.42, 121.83, 121.82, 80.02, 79.09, 78.87, 78.82, 75.72, 75.61, 74.59, 74.23, 69.93, 69.89, 62.65, 51.37, 47.99, 47.91, 36.83, 32.08, 30.60, 30.56, 30.24, 30.19, 29.86, 29.84, 29.82, 29.82, 29.80, 29.75, 29.60, 29.52, 29.40, 29.23, 25.58, 24.49, 22.85, 21.17, 21.13, 20.53, 19.56, 18.04, 17.21, 17.16, 14.28, 12.08, 6.12. **LRMS** (ESI-Quad) [m/z]: 552.9 $[M+H]^+$, **HRMS** (ESI-IT) [m/z]: 574.312547, calculated 574.313909 for $C_{33}H_{45}NNaO_6$ $[M+Na]^+$, err [ppm] 1.675.

Synthesis of N-Propargyl-19-aminoratjadone (35) - (6R)-6-((1E,3Z,5R,7E,9E,11R)-11-hydroxy-3,5,7-trimethyl-11-((2S,5S,6S)-5-methyl-6-((E)-prop-1-en-1-yl)-4-(prop-2-yn-1-ylamino)tetrahydro-2H-pyran-2-yl)undeca-1,3,7,9-tetraen-1-yl)-5,6-dihydro-2H-pyran-2-one

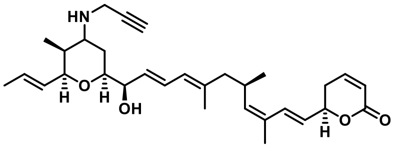

Chemical Formula: $C_{31}H_{43}NO_4$
Molecular Weight: 493,6880
N-Propargyl-19-aminoratjadone **35**

2.54 µL (39.595 µmol, 2.0 equiv) propargylamine and 2.2 µL (39.595 µmol, 2.0 equiv) acetic acid were added to a solution of 9.0 mg (19.797 µmol, 1.0 equiv) 19-oxoratjadone **9** in 396 µL dry MeOH at 23°C and stirred for 15 min, before 2.5 mg (39.595 µmol, 2.0 equiv) sodium cyanoborohydride was added and the mixture was stirred at 23°C for 4 h, before the reaction was quenched by addition of 200 µL of ACN:H_2O + TFA/30:70 + 0.05%. This mixture was directly purified by RP prep HPLC (Phenomenex Gemini C18 RP-column 00G-4435-PO-AX, 5 µm, 110 A, 250×21.20 mm, Flow: 9 mL/min, ACN:H_2O + TFA/ 10:90 + 0.1% → 95:5 + 0.1% in 90 min) yielding after lyophilization 9.7 mg (19.648 µmol, 99%) *N*-Propargyl-19-aminoratjadone **35** as an inseparable 1:2 mixture of 2 diastereoisomers in its TFA salts as a pale-yellow solid foam.

N-Propargyl-19-aminoratjadone (35): ^{1}**H-NMR** (500 MHz, $CDCl_3$) δ [ppm]: 6.95 – 6.88 (m, 1H), 6.73 – 6.58 (m, 1H), 6.54 – 6.44 (m, 1H), 6.08 – 6.03 (m, 1H), 5.81 – 5.66 (m, 3H), 5.58 – 5.31 (m, 2H), 5.28 – 5.16 (m, 1H), 4.98 (dddd, *J* = 15.3, 11.7, 7.4, 4.7 Hz, 1H), 4.34 (ddt, *J* = 33.7, 10.0, 5.3 Hz, 1H), 4.04 – 3.63 (m, 6H), 3.50 (ddt, *J* = 13.8, 9.9, 5.5 Hz, 1H), 2.78 (tt, *J* = 17.7, 8.7 Hz, 1H), 2.54 – 2.44 (m, 3H), 2.17 – 2.05 (m, 1H), 2.00 (td, *J* = 13.1, 12.0, 6.4 Hz, 2H), 1.94 – 1.83 (m, 1H), 1.79 – 1.78 (m, 3H), 1.74 – 1.71 (m, 6H), 1.71 – 1.67 (m, 2H), 1.06 – 0.92 (m, 6H). ^{13}**C-NMR** (126 MHz, $CDCl_3$) δ [ppm]: 165.16, 164.64, 164.62, 164.49, 162.69, 162.49, 162.28, 162.08, 145.67, 145.20, 145.17, 145.13, 139.53, 139.46, 139.43, 138.27, 137.82, 137.71, 137.67, 131.63, 131.46, 131.35, 131.24, 131.17, 129.68, 129.53, 129.46, 129.38, 129.27, 129.19, 128.82, 128.77, 128.68, 128.54, 128.49, 128.48, 128.30, 128.20, 127.88, 127.82, 127.49, 125.99, 125.90, 125.81, 125.30, 125.27, 125.11, 121.67, 121.65, 121.43, 118.95, 117.29, 115.64, 113.98, 83.23, 79.74, 79.52, 79.15, 79.11, 78.59, 77.95, 77.86, 77.67, 77.63, 77.55, 74.64, 74.58, 73.97, 73.89, 73.70, 73.64, 73.61, 73.32, 73.25, 73.15, 73.12, 59.19, 57.97, 57.26, 56.71, 53.57, 47.87, 47.83, 47.80, 38.58, 37.67, 36.00, 35.46, 34.40, 34.26, 33.74, 33.68, 30.76, 30.74, 30.72, 30.70, 30.32, 30.21, 30.19, 29.85, 26.95, 26.50, 22.38, 21.56, 21.51, 21.28, 21.23, 20.50, 20.49, 18.01, 17.98, 17.41, 17.31, 17.29, 13.63, 13.44, 12.04, 5.86, 1.17. **LRMS** (ESI-Quad) [m/z]: 494.7 $[M+H]^+$, **HRMS** (ESI-IT) [m/z]: 494.3268, calculated 494.3265 for $C_{31}H_{44}NO_4$ $[M+H]^+$, err [ppm] – 0.6.

Synthesis of appropriate carrier molecules

Synthesis of Folate derivatives in solution

Synthesis of methyl N^2-(((9H-fluoren-9-yl)methoxy)carbonyl)-N^6-(tert-butoxycarbonyl)-L-lysinate (37)

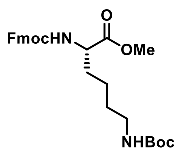

Chemical Formula: $C_{27}H_{34}N_2O_6$
Molecular Weight: 482,58
37

To a solution of 1.0 g (2.134 mmol, 1.0 equiv) N^2-(((9H-fluoren-9-yl)methoxy)carbonyl)-N^6-(tert-butoxycarbonyl)-L-lysine in 10.7 mL dry DMF were added 590 mg (4.268 mmol, 2.0 equiv) K_2CO_3 and 200 µL (3.201 mmol, 1.5 equiv) methyl iodide and the mixture was stirred for 3.5 h at 23°C. The reaction was diluted with Et_2O (30 mL) and the mixture was washed with saturated aqueous NH_4Cl solution (2x 150 mL) and H_2O (2x 50 mL). The organic layer was dried over Na_2SO_4, concentrated under reduced pressure and dried in HV yielding 885 mg (1.835 mmol, 86%) of **37** as pale-yellow solid.

N^2-(((9H-fluoren-9-yl)methoxy)carbonyl)-N^6-(tert-butoxycarbonyl)-L-lysinate (37): TLC (CH_2Cl_2:MeOH/95:5) R_f: 0.90 [UV^{254}], **IR** (ATR) [cm^{-1}]: 3343, 2976, 2951, 2932, 2866, 1653, 1609, 1510, 1478, 1450, 1392, 1366, 1341, 1248, 1211, 1166, 1106, 1081, 1044, 1005, 909, 865, 780, 755, 728, 674, 646, 621. **^{1}H-NMR** (700 MHz, $CDCl_3$) δ [ppm]: 7.74 (d, *J* = 7.4 Hz, 2H), 7.62 – 7.55 (m, 2H), 7.38 (t, *J* = 7.2 Hz, 2H), 7.29 (t, *J* = 6.9 Hz, 2H), 5.42 (s, 1H), 4.58 (s, 1H), 4.38 (dt, *J* = 17.8, 8.6 Hz, 3H), 4.20 (t, *J* = 6.5 Hz, 1H), 3.73 (s, 3H), 3.09 (s, 2H), 1.83 (s, 1H), 1.68 (s, 1H), 1.41 (s, 9H), 1.52 – 1.29 (m, 5H). **^{13}C-NMR** (176 MHz, $CDCl_3$) δ [ppm]: 173.04, 156.18, 156.09, 144.00, 143.85, 141.40, 127.80, 127.17, 125.20, 120.08, 79.26, 67.10, 53.82, 52.52, 47.27, 40.15, 32.22, 29.70, 28.53, 22.47. **LRMS** (ESI-Quad) [m/z]: 483.2 [M+H]$^+$, **HRMS** (ESI-IT) [m/z]: 483.2493, calculated 483.2490 for $C_{27}H_{34}N_2O_6$ [M+H]$^+$, err [ppm] -0.600.

Synthesis of methyl N[2]-((S)-4-((((9H-fluoren-9-yl)methoxy)carbonyl)amino)-5-(tert-butoxy)-5-oxopentanoyl)-N[6]-(tert-butoxycarbonyl)-L-lysinate (38)

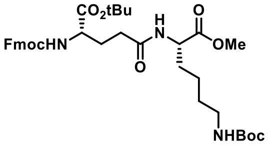

Chemical Formula: $C_{36}H_{49}N_3O_9$
Molecular Weight: 667,80

38

To a solution of 100 mg (0.207 mmol, 1.2 equiv) methyl N[2]-(((9H-fluoren-9-yl)methoxy)carbonyl)-N[6]-(tert-butoxycarbonyl)-L-lysinate **37** in 4.1 mL CH_2Cl_2 was added 332 µL (1.6 mL/mmol) piperidine and the mixture was stirred for 3 h at 23°C until the starting material was consumed. The mixture was diluted with toluene (10 mL), concentrated and co-evaporated with toluene (3x 10 mL) under reduced pressure. The residue containing the free amine was dissolved in 1.2 mL dry DMF and 57 mL (0.517 mmol, 3.0 equiv) NMM, 74 mg (0.1725 mmol, 1.0 equiv) (S)-4-((((9H-fluoren-9-yl)methoxy)carbonyl)amino)-5-(tert-butoxy)-5-oxopentanoic acid and 28.2 mg (0.207 mmol, 1.0 equiv) HOAt were added and the mixture was cooled to 0°C. 39.7 mg (0.207 mmol, 1.0 equiv) EDCI was added and the mixture was stirred for 12 h at 23°C, before it was poured into 5 mL of H_2O and extracted with EtOAc (3x 15 mL). The combined organic layers were dried over Na_2SO_4 and concentrated under reduced pressure. The residue was purified by flash-chromatography through silicagel (PE:EtOAc/8:2 – 1:1 -0:1) yielding 80 mg (0.1197 mmol, 58%) of **38** as a colorless, amorph solid.

Methyl N[2]-((S)-4-((((9H-fluoren-9-yl)methoxy)carbonyl)amino)-5-(tert-butoxy)-5-oxopentanoyl)-N[6]-(tert-butoxycarbonyl)-L-lysinate (38): TLC (PE:EtOAc/1:1) R_f: 0.16 [UV^{254}], **IR** (ATR) [cm^{-1}]: 3329, 2977, 2952, 2931, 2868, 171 1663, 1525, 1479, 1451, 1392, 1367, 1346, 1249, 1158, 1105, 1082, 1052, 912, 848, 760, 738, 647, 621, 591, 575, 526. **^{1}H-NMR** (500 MHz, $CDCl_3$) δ [ppm]: 7.77 (d, *J* = 7.6 Hz, 2H), 7.61 (d, *J* = 7.3 Hz, 2H), 7.40 (td, *J* = 7.4, 2.9 Hz, 2H), 7.35 – 7.30 (m, 2H), 6.40 (d, *J* = 6.4 Hz, 1H), 5.56 (d, *J* = 8.1 Hz, 1H), 4.64 (s, 1H), 4.58 (q, *J* = 7.6 Hz, 1H), 4.48 – 4.35 (m, 2H), 4.23 (q, *J* = 7.7, 7.0 Hz, 2H), 3.72 (s, 3H), 3.09 (d, *J* = 5.7 Hz, 2H), 2.29 (t, *J* = 6.8 Hz, 2H), 2.26 – 2.18 (m, 1H), 1.94 – 1.80 (m, 2H), 1.70 (q, *J* = 13.4 Hz, 1H), 1.47 (s, 9H), 1.42 (s, 9H), 1.52 – 1.28 (m, 3H), 0.94 – 0.75 (m, 2H).. **^{13}C-NMR** (126 MHz, $CDCl_3$) δ [ppm]: 172.97, 171.86, 171.23, 156.50, 156.21, 144.06, 143.83, 141.47, 127.87, 127.23, 125.31, 125.25, 120.14, 120.12, 82.73, 79.24, 67.15, 53.97, 52.52, 52.23, 47.36, 40.16, 32.40, 32.04, 29.69, 29.16, 28.57, 28.15, 22.56. . **LRMS** (ESI-Quad) [m/z]: 668.4 $[M+H]^+$, **HRMS** (ESI-IT) [m/z]: 668.354552, calculated 668354157 for $C_{36}H_{50}N_3O_9$ $[M+H]^+$, err [ppm] -0.592

Synthesis of methyl N²-(((9H-fluoren-9-yl)methoxy)carbonyl)-N⁵-((S)-6-(4-azidobenzamido)-1-methoxy-1-oxohexan-2-yl)-L-glutamine (39)

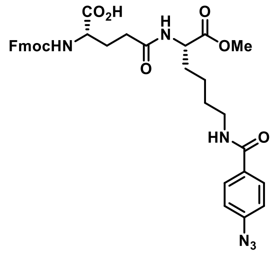

Chemical Formula: $C_{34}H_{36}N_6O_8$
Molecular Weight: 656,70

39

To a solution of 100 mg (0.149 mmol, 1.0 equiv) of methyl N^2-((S)-4-((((9H-fluoren-9-yl)methoxy)carbonyl)amino)-5-(tert-butoxy)-5-oxopentanoyl)-N^6-(tert-butoxycarbonyl)-L-lysinate **38** in 2.99 mL dry CH_2Cl_2 was added 344 µL (4.492 mmol, 30 equiv) TFA at 23°C and the mixture was stirred for 22 h at 23°C until the starting material was completely consumed. The reaction mixture was diluted with toluene (20 mL), concentrated and co-evaporated with toluene (3x 10 mL) under reduced pressure and dried in HV. The residue containing the free amine was dissolved together with 83 µL (0.749 mmol, 5.0 equiv) NMM in 499 µL dry DMF and added to a preactivated[vii] solution of 26.9 mg (0.165 mmol, 1.0 equiv) 4-azidobenzoic acid, 83 µL (0.749 mmol, 5.0 equiv) NMM, 22.4 mg (0.165 mmol, 1.0 equiv) HOAt and 62.6 mg (0.165 mmo, 1.0 equiv) HATU in 499 µL dry DMF. The resulting solution was stirred for 3 h at 23°C, before was diluted with toluene (20 mL), concentrated and co-evaporated with toluene (3x 20 mL) under reduced pressure. The residue was purified by flash-chromatography through silicagel (CH_2Cl_2:MeOH:HOAc/95:4.75:0.25) and the product containing fractions were coevaporated with toluene under reduced pressure and dried in HV yielding 86 mg (0.131 mmol, 88%) of **39** as a yellow, amorph solid.

Methyl N²-(((9H-fluoren-9-yl)methoxy)carbonyl)-N⁵-((S)-6-(4-azidobenzamido)-1-methoxy-1-oxohexan-2-yl)-L-glutamine (39): TLC (PE:EtOAc/1:1) R_f: 0.16 [UV^{254}], **IR** (ATR) [cm^{-1}]: 3415, 2931, 2860, 2123, 1686, 1641, 1604, 1573, 1537, 1500, 1450, 1281, 1205, 1160, 1132, 1064, 1053, 992, 910, 840, 801, 761, 741, 724, 689, 647, 621. **^{1}H-NMR** (500 MHz, MeOD-*d₄*) δ [ppm]: 7.85 – 7.79 (m, 1H), 7.78 (d, *J* = 7.5 Hz, 1H), 7.66 (t, *J* = 7.4 Hz, 1H), 7.38 (t, *J* = 7.5 Hz, 1H), 7.30 (td, *J* = 7.5, 1.0 Hz, 1H), 7.14 – 7.05 (m, 1H), 4.39 (dd, *J* = 9.1, 5.0 Hz, 1H), 4.38 – 4.26 (m, 1H), 4.20 (t, *J* = 6.9 Hz, 1H), 4.11 (dd, *J* = 8.6, 4.6 Hz, 1H), 3.85 – 3.76 (m, 2H), 3.68 (s, 2H), 3.36 (t, *J* = 6.9 Hz, 1H), 3.01 (s, 2H), 2.32 (t, *J* = 7.4 Hz, 1H), 2.17 (td, *J* = 13.0, 8.0 Hz, 1H), 1.97 – 1.81 (m, 2H), 1.72 (dtd, *J* = 14.2, 9.3, 5.7 Hz, 1H), 1.62 (dhept, *J* = 13.6, 6.9 Hz, 2H), 1.51 – 1.38 (m, 2H)..

[vii] For preactivation the mixture was stirred for 30 min at 23°C.

^{13}C-NMR (126 MHz, MeOD-*d4*) δ [ppm]: 177.07, 175.41, 174.25, 169.13, 158.42, 145.39, 145.20, 144.73, 142.55, 132.24, 130.14, 128.76, 128.17, 126.27, 126.24, 120.89, 119.95, 67.91, 65.84, 56.03, 55.09, 53.70, 52.64, 48.42, 44.67, 40.63, 33.15, 32.12, 29.95, 29.55, 24.28. **LRMS** (ESI-Quad) [m/z]: 657.3 $[M+H]^+$, **HRMS** (ESI-IT) [m/z]: 657.267199, calculated 657.266739 for $C_{34}H_{36}N_6O_8$ $[M+H]^+$, err [ppm] -0.700.

Synthesis of Fa-N$_3$-1 - N^2-(4-(((2-amino-4-hydroxypteridin-6-yl)methyl)amino)benzoyl)-N^5-((S)-4-(((S)-6-(4-azidobenzamido)-1-methoxy-1-oxohexan-2-yl)amino)-1-carboxy-4-oxobutyl)-L-glutamine

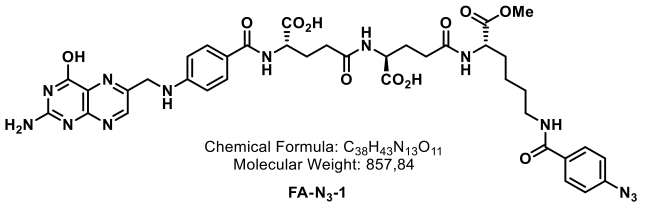

FA-N$_3$-1

To a solution of 60 mg (0.091 mmol, 1.0 equiv) methyl N^2-(((9H-fluoren-9-yl)methoxy)carbonyl)-N^5-((S)-6-(4-azidobenzamido)-1-methoxy-1-oxohexan-2-yl)-L-glutamine **39** in 456 µL dry DMF was added 146 µL (1.6 mL/mmol) diethylamine and the mixture was stirred for 20 h at 23°C, before it was diluted with toluene (20 mL), concentrated and co-evaporated with toluene (3x 10 mL) under reduced pressure and dried in HV obtaining a residue containing the free amine.

In parallel, 63 mg (0.548 mmol, 6.0 equiv) N-hydroxysuccinimide and 113 mg (0.548 mmol, 6.0 equiv) DCC were added to a solution of 242 mg (0.548 mmol, 6.0 equiv) folic acid in 2.7 mL dry DMSO and the mixture was stirred for 24 h at 23°C under light exclusion to form the corresponding FA-NHS ester in a white suspension. This suspension was then filtered and the filtrate was poured onto the residue containing the free amine (described above). 90 µL (0.823 mmol, 9.0 equiv) NMM were added and the mixture was stirred for 14 h at 23°C. The mixture was diluted with 1.2 mL H_2O, filtered through a Whatman® filter (45 µm) and directly purified RP prep HPLC (Phenomenex Gemini C18 RP-column 00G-4435-PO-AX, 5 µm, 110 A, 250×21.20 mm, Flow: 9 mL/min, ACN:H_2O + TFA/ 10:90 + 0.1% → 95:5 + 0.1% in 60 min) yielding after lyophilization 35.3 mg (41.1 µmol, 45%) of **Fa-N$_3$-1** as a deep-yellow solid.

Fa-N$_3$-1: ^{1}H-NMR (500 MHz, DMSO-*d*$_6$) δ [ppm]: 12.54 (s, 2H), 8.67 (s, 1H), 8.57 – 8.38 (m, 1H), 8.28 – 8.18 (m, 2H), 8.15 (dd, *J* = 15.1, 7.8 Hz, 1H), 7.90 – 7.85 (m, 2H), 7.66 (dd, *J* = 8.6, 6.3 Hz, 2H), 7.47 – 6.85 (m, 2H), 7.18 (d, *J* = 8.6 Hz, 2H), 6.64 (d, *J* = 7.6 Hz, 2H), 4.50 (s, 2H), 4.36 – 4.26 (m, 1H), 4.23 – 4.06 (m, 3H), 3.27 – 3.12 (m, 3H), 2.99 (s, 1H), 2.33 – 2.22 (m, 2H), 2.19 (dd, *J* = 18.2, 9.1 Hz, 2H), 2.06 (d, *J* = 13.6 Hz, 1H), 1.99 – 1.83 (m, 2H), 1.77 – 1.63 (m, 2H), 1.63 – 1.54 (m, 1H), 1.48 (s, 2H), 1.31 (s, 2H). **^{13}C-NMR** (126 MHz, MeOD-*d4*) δ [ppm]: ^{13}C NMR (126 MHz, DMSO) δ 173.71, 173.38, 172.73, 171.76, 171.65, 171.50, 166.37, 166.27, 165.09, 160.54, 153.36, 150.68, 149.25, 148.37, 142.04, 131.16, 128.98, 127.92, 121.34,

118.80, 111.16, 52.20, 51.83, 51.69, 51.44, 45.84, 31.32, 31.27, 30.50, 28.59, 26.87, 26.66, 22.81. **LRMS** (ESI-Quad) [m/z]: 858.3 $[M+H]^+$, **HRMS** (ESI-IT) [m/z]: 858.327991, calculated 858.327776 for $C_{38}H_{44}N_{13}O_{11}$ $[M+H]^+$, err [ppm] -0.25.

Synthesis of methyl N^2-(((9H-fluoren-9-yl)methoxy)carbonyl)-N^6-(4-azidobenzoyl)-L-lysinate (40)

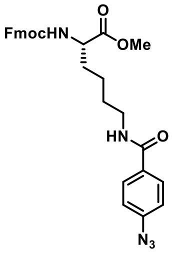

Chemical Formula: $C_{29}H_{29}N_5O_5$
Molecular Weight: 527,58
40

To a solution of 40.5 mg (0.248 mmol, 1.2 equiv) 4-azidobenzoic acid and 29.8 mg (0.258 mmol, 1.25 equiv) N-hydroxysuccinimide in 1.2 mL dry THF was added 53.4 mg (0.258 mmol, 1.25 equiv) DCC and the mixture was stirred for 9 h at 23°C to form the corresponding 4-azidobenzoic acid NHS ester in a white suspension. In parallel, 237 µL (3.108 mmol, 15 equiv) TFA was added to a solution of 100 mg (0.207 mmol, 1.0 equiv) methyl N^2-(((9H-fluoren-9-yl)methoxy)carbonyl)-N^6-(tert-butoxycarbonyl)-L-lysinate **37** in 4.2 mL CH_2Cl_2 and the mixture was stirred for 3 h at 23°C until the starting material was consumed. The mixture was diluted with toluene (20 mL), concentrated and coevaporated with toluene (3x 10 mL) under reduced pressure. The residue containing the corresponding free amine was dissolved in 0.9 mL dry THF, 136.5 µL (1.242 mmol, 6.0 equiv) NMM was added and the filtrate of the suspension containing the 4-azidobenzoic acid NHS ester added. The resulting mixture was stirred for 20 h at 23°C. The solvents were removed under reduced pressure and the residue was purified by flash-chromatography through silicagel (CH_2Cl_2:MeOH/1:0 – 97.5:2.5) yielding 98 mg (0.186 mmol, 90%) of **40** as an amorph, yellow solid.

Methyl N^2-(((9H-fluoren-9-yl)methoxy)carbonyl)-N^6-(4-azidobenzoyl)-L-lysinate (40): ^{1}H-NMR (500 MHz, $CDCl_3$) δ [ppm]: 7.78 – 7.74 (m, 4H), 7.56 (d, *J* = 7.4 Hz, 2H), 7.40 (td, *J* = 7.4, 3.1 Hz, 2H), 7.32 – 7.26 (m, 2H), 6.95 (d, *J* = 8.4 Hz, 2H), 6.36 (s, 1H), 5.54 (d, *J* = 8.2 Hz, 1H), 4.43 – 4.34 (m, 2H), 4.30 (dd, *J* = 10.5, 7.3 Hz, 1H), 4.18 (t, *J* = 7.1 Hz, 1H), 3.75 (s, 3H), 3.44 (q, *J* = 6.4 Hz, 2H), 1.93 – 1.84 (m, 1H), 1.80 – 1.59 (m, 4H), 1.46 (dt, *J* = 16.3, 7.9 Hz, 2H), 1.32 – 1.24 (m, 1H). **^{13}C-NMR** (126 MHz, $CDCl_3$) δ [ppm]: 173.02, 166.78, 156.30, 143.91, 143.75, 143.28, 141.40, 131.07, 128.82, 127.87, 127.19, 125.15, 120.14, 119.00, 67.22, 53.55, 52.63, 47.24, 39.71, 32.84, 32.54, 28.86, 25.22, 24.51, 22.62. **LRMS** (ESI-Quad) [m/z]: 528.2 $[M+H]^+$, **HRMS** (ESI-IT) [m/z]: 528.224467, calculated 528.224146 for $C_{29}H_{30}N_5O_5$ $[M+H]^+$, err [ppm] -0.608.

Synthesis of Fmoc-(Asp(tBu))$_3$-OH - (5S,8S,11S)-5,8,11-tris(2-(tert-butoxy)-2-oxoethyl)-1-(9H-fluoren-9-yl)-3,6,9-trioxo-2-oxa-4,7,10-triazadodecan-12-oic acid

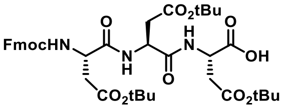

Chemical Formula: $C_{39}H_{51}N_3O_{12}$
Molecular Weight: 753,85
Fmoc-(Asp(tBu))$_3$-OH

The solid-phase synthesis of the tripeptide **Fmoc-(Asp(tBu))$_3$-OH** was carried out manually on a scale of 100 µmol on Rapp 2-chlorotrityl resin (Rapp Polymere, Tübingen, Germany, 0.91 mmol/g) using fritted glas peptide synthesis vessels. For the loading of the first amino acid to the resin a solution 41.2 mg (100 µmol, 1.0 equiv) Fmoc-Asp(OtBu)-OH and 110 µL (1.0 mmol, 10 equiv) NMM in 4 mL dry DMF was reacted with the resin shaking it for 24 h at 23°C. The solvent was removed from the resin and the resin was reacted shaking it for 1 h at 23°C with a solution of 1 mL MeOH and 220 µL (2.0 mmol, 20 equiv) NMM in 4 mL dry DMF. The resin was washed with DMF, CH_2Cl_2 and DMF (each 3x 4 mL, 2 min). Fmoc cleavage achieved by reapeated reaction of the resin shaking it with a mixture of Piperidin in DMF (Pip:DMF/1:4, 3x 4 mL, 10 min) and subsequent washing of the resin with DMF, CH_2Cl_2 and DMF (each 3x 4 mL, 2 min). The coupling of the amino acids was performed shaking with preactivated solutions of 84.4 mg (200 µmol, 2.0 equiv) Fmoc-D-Asp(OtBu)-OH or Fmoc-Asp(OtBu)-OH, 27.2 (200 µmol, 2.0 equiv) HOAt, 55 µL (600 µmol, 6.0 equiv) NMM and 165 mg (200 µmol, 2.0 equiv) HATU in 4 mL of dry DMF with coupling times of 4 h at 23°C. Subsequently the resin was washed with DMF, CH_2Cl_2 and DMF (each 3x 4 mL, 2 min). The cleavage of the tripeptide from the resin was managed by repeated treatment of the resin with a mixture of CH_2Cl_2:HFIP/4:1 (3x 4 mL, 10 min). The combined cleavage solutions were concentrated under reduced pressure and the residue was purified by flash-chromatography through silicagel (PE:EtOAc/8:2 – 1:1) yielding **Fmoc-(Asp(tBu))$_3$-OH** as a white, amorph solid.

Fmoc-(Asp(tBu))$_3$-OH: **TLC:** **^{1}H-NMR** (500 MHz, DMSO-d_6) δ [ppm]: 8.28 – 8.20 (m, 1H), 8.09 (s, 2H), 7.89 (d, J = 7.6 Hz, 2H), 7.74 – 7.62 (m, 3H), 7.41 (t, J = 7.5 Hz, 2H), 7.32 (t, J = 7.4 Hz, 2H), 5.16 (p, J = 6.8 Hz, 2H), 4.64 – 4.54 (m, 1H), 4.53 – 4.43 (m, 1H), 4.38 (td, J = 9.7, 4.3 Hz, 1H), 4.32 – 4.19 (m, 1H), 2.64 (ttd, J = 14.3, 7.6, 6.3, 3.9 Hz, 2H), 2.50 (dt, J = 3.7, 1.8 Hz, 2H), 1.42 – 1.32 (m, 29H). **^{13}C-NMR** (126 MHz, $CDCl_3$) δ [ppm]: 171.15, 171.03, 170.80, 170.50, 170.04, 163.19, 156.20, 143.75, 141.38, 127.86, 127.24, 125.24, 120.09, 82.16, 82.06, 82.00, 67.57, 51.65, 49.67, 49.27, 47.16, 37.50, 37.10, 36.95, 28.09. **LRMS** (ESI-Quad) [m/z]: 776.3 $[M+Na]^+$, **HRMS** (ESI-IT) [m/z]: 776.3395 , calculated 776.3365 for $C_{23}H_{34}N_5NaO_6$ $[M+Na]^+$, err [ppm] 3.86

Synthesis of (7S,10S,13S,16S)-16-((((9H-fluoren-9-yl)methoxy)carbonyl)amino)-1-(4-azidophenyl)-10,13-bis(carboxymethyl)-7-(methoxycarbonyl)-1,9,12,15-tetraoxo-2,8,11,14-tetraazaoctadecan-18-oic acid (41)

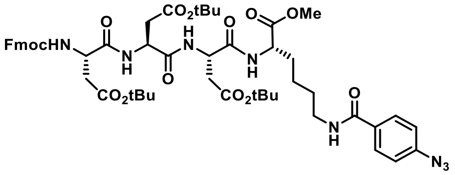

Chemical Formula: $C_{53}H_{68}N_8O_{14}$
Molecular Weight: 1041,17

41

To a solution of 53 mg (99.5 µmol, 1.5 equiv) of methyl N^2-(((9H-fluoren-9-yl)methoxy)carbonyl)-N6-(4-azidobenzoyl)-L-lysinate **40** in 1.6 mL CH_2Cl_2 was added 159 µL (1.6 mL/mmol) diethylamine and the mixture was stirred for 9 h at 23°C. The mixture was diluted with toluene (5 mL), concentrated and coevaporated with toluene (3x 5 mL) under reduced pressure and dried in HV. The residue was dissolved in 201 µL dry DMF and the resulting solution was added to a preactivated[viii] mixture of 50 mg (60.3 µmol, 1.0 equiv) **Fmoc-(Asp(OtBu))₃-OH**, 9.9 mg (72.5 µmol, 1.2 equiv) HOAt, 437 µl (0.397 mmol, 6.0 equiv) NMM and 13.9 mg (72.5 µmol, 1.2 equiv) HATU and the mixture was stirred for 5.5 h at 23°C. The mixture was poured into 50 mL H_2O and extracted with Et_2O (3x 20 mL). The combined organic extracts were dried over Na_2SO_4 and concentrated under reduced pressure. The residue was purified by flash-chromatography through silicagel (PE:EtOAc/9:1 – 7:3 – 1:1 – 0:1) yielding 35.6 mg (34.1 µmol, 52%) of **41** as a pale-yellow, amorph solid.

(7S,10S,13S,16S)-16-((((9H-fluoren-9-yl)methoxy)carbonyl)amino)-1-(4-azidophenyl)-10,13-bis(carboxy-methyl)-7-(methoxycarbonyl)-1,9,12,15-tetraoxo-2,8,11,14-tetraazaoctadecan-18-oic acid (41): TLC (PE:EtOAc/1:1) R_f: 0.19 [UV^{254}, Ninhydrin], **^{1}H-NMR** (500 MHz, DMSO-*d₆*) δ [ppm]: 8.44 (q, *J* = 5.8 Hz, 1H), 8.37 – 8.20 (m, 1H), 8.10 – 7.93 (m, 2H), 7.91 – 7.83 (m, 4H), 7.77 – 7.70 (m, 1H), 7.69 (t, *J* = 8.3 Hz, 2H), 7.41 (t, *J* = 7.5 Hz, 2H), 7.31 (t, *J* = 7.5 Hz, 2H), 7.21 – 7.12 (m, 2H), 4.66 – 4.46 (m, 2H), 4.41 – 4.14 (m, 4H), 3.59 (s, 3H), 3.27 – 3.15 (m, 2H), 2.73 – 2.58 (m, 3H), 2.48 – 2.38 (m, 3H), 1.78 – 1.58 (m, 2H), 1.54 – 1.44 (m, 2H), 1.39 – 1.30 (m, 29H). **^{13}C-NMR** (126 MHz, DMSO-*d₆*) δ [ppm]: 171.54, 170.22, 170.18, 169.48, 168.88, 168.79, 168.69, 164.86, 155.31, 143.41, 143.34, 141.59, 140.32, 131.27, 128.58, 127.12, 126.54, 124.66, 119.52, 118.31, 79.97, 79.84, 65.70, 54.25, 51.76, 51.73, 51.31, 51.16, 49.65, 49.61, 49.51, 49.30, 49.22, 46.40, 38.61, 37.80, 37.13, 37.06, 36.93, 36.84, 36.79, 30.36, 30.33, 28.19, 28.13, 27.35, 27.30, 22.28. **LRMS** (ESI-Quad) [m/z]: 1042.3 $[M+Na]^+$.

[viii] For preactivation the mixture was stirred for 15 min at 23°C.

Synthesis of FA-N_3-2 - N[2]-(4-(((2-amino-4-hydroxypteridin-6-yl)methyl)amino)benzoyl)-N[5]-((S)-1-(((S)-1-(((S)-1-(((S)-6-(4-azidobenzamido)-1-methoxy-1-oxohexan-2-yl)amino)-3-carboxy-1-oxopropan-2-yl)amino)-3-carboxy-1-oxopropan-2-yl)amino)-3-carboxy-1-oxopropan-2-yl)-L-glutamine

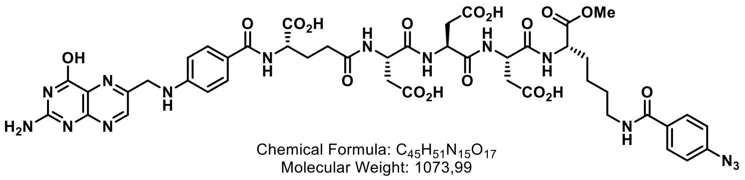

Chemical Formula: $C_{45}H_{51}N_{15}O_{17}$
Molecular Weight: 1073,99
FA-N_3-2

To a solution of 20 mg (0.0233 mmol, 1.0 equiv) (7S,10S,13S,16S)-16-((((9H-fluoren-9-yl)methoxy)carbonyl)amino)-1-(4-azidophenyl)-10,13-bis(carboxymethyl)-7-(methoxycarbonyl)-1,9,12,15-tetraoxo-2,8,11,14-tetraazaoctadecan-18-oic acid **41** in 466 µL dry CH_2Cl_2 was added 89 µL (1.166 mmol, 50.0 equiv) TFA and the mixture was stirred for 28 h at 23°C. The reaction was diluted with toluene (5 mL), concentrated and coevaporated with toluene (3x 5 mL) under reduced pressure. The resulting residue was dried in HV, re-dissolved in 466 µL CH_2Cl_2 and 75 µL (3.2 mL/mmol) diethylamine was added and the reactions mixture was stirred for 20 h at 23°C. The mixture was diluted with toluene (5 mL), concentrated and coevaporated with toluene (3x 5 mL) under reduced pressure. The resulting residue containing the raw (7S,10S,13S,16S)-16-amino-1-(4-azidophenyl)-10,13-bis(carboxymethyl)-7-(methoxycarbonyl)-1,9,12,15-tetraoxo-2,8,11,14-tetraazaoctadecan-18-oic acid was dried in HV.

In parallel, 16 mg (0.1398 mmol, 6.0 equiv) N-hydroxysuccinimide and 29 mg (0.1398 mmol, 6.0 equiv) DCC were added to a solution of 62 mg (0.1398 mmol, 6.0 equiv) folic acid in 699 µL dry DMSO and the mixture was stirred for 24 h at 23°C under light exclusion to form the corresponding FA-NHS ester in a white suspension. This suspension was then filtered and the filtrate was poured onto the residue containing the (7S,10S,13S,16S)-16-amino-1-(4-azidophenyl)-10,13-bis(carboxymethyl)-7-(methoxycarbonyl)-1,9,12,15-tetraoxo-2,8,11,14-tetraazaoctadecan-18-oic acid (described above). 23 µL (0.209 mmol, 9.0 equiv) NMM were added and the mixture was stirred for 20 h at 23°C. The mixture was diluted with 600 µL H_2O:ACN/70:30 + 0.05% TFA, filtered through a Whatman® filter (45 µm) and directly purified RP prep HPLC (Phenomenex Gemini C18 RP-column 00G-4435-PO-AX, 5 µm, 110 A, 250×21.20 mm, Flow: 9 mL/min, ACN:H_2O + TFA/ 10:90 + 0.1% → 95:5 + 0.1% in 60 min) yielding after lyophilization 8.0 mg (7.45 µmol, 32%) of **FA-N_3-2** as its TFA salt as a deep-yellow solid.

Fa-N_3-2: ^{1}H-NMR (500 MHz, DMSO-d_6) δ [ppm]: 12.33 (s, 4H), 8.66 (s, 1H), 8.41 (dd, *J* = 27.8, 6.8 Hz, 1H), 8.27 – 8.10 (m, 3H), 8.06 – 7.91 (m, 1H), 7.89 – 7.86 (m, 2H), 7.86 – 7.76 (m, 1H), 7.66 (d, *J* = 8.7 Hz, 2H), 7.21 – 7.13 (m, 2H), 7.07 (s, 2H), 6.64 (d, *J* = 8.5 Hz, 2H), 4.61 – 4.43 (m, 5H), 4.36 – 4.25 (m, 1H), 4.26 – 4.13 (m, 2H), 3.62 – 3.49 (m, 3H), 3.29 – 3.14 (m, 4H), 2.79 – 2.59 (m, 4H), 2.33 – 2.14 (m, 2H), 2.10 – 2.00

(m, 1H), 1.95 – 1.82 (m, 1H), 1.76 – 1.58 (m, 2H), 1.49 (d, *J* = 5.0 Hz, 2H), 1.29 (d, *J* = 30.6 Hz, 2H). **^{13}C-NMR** (126 MHz, DMSO-d_6) δ [ppm]: 173.71, 173.70, 172.23, 172.20, 171.85, 171.84, 171.63, 170.49, 170.42, 170.34, 170.27, 166.30, 165.12, 160.67, 158.29, 158.10, 157.90, 157.71, 153.46, 150.71, 149.06, 148.43, 142.03, 131.19, 129.02, 127.93, 121.28, 118.80, 111.16, 52.06, 51.78, 49.64, 45.85, 40.01, 35.99, 35.82, 31.85, 30.41, 28.58, 28.46, 26.53, 22.69, 22.65, 14.01, 13.05. **LRMS** (ESI-Quad) [m/z]: 1074.4 $[M+H]^+$, **HRMS** (ESI-IT) [m/z]: 1074.36278, calculated 1074.36601 for $C_{45}H_{51}N_{15}O_{17}$ $[M+H]^+$, err [ppm] -3.006.

Synthesis of Folate derivatives by Solid-Phase Synthesis

Synthesis of N^2-(((9H-fluoren-9-yl)methoxy)carbonyl)-N^6-(4-azidobenzoyl)-L-lysine

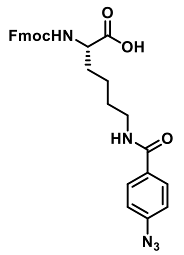

Chemical Formula: $C_{28}H_{27}N_5O_5$
Molecular Weight: 513,55

To a solution of 1.5 g (3.201 mmol, 1.0 equiv) N^2-(((9H-fluoren-9-yl)methoxy)carbonyl)-N^6-(tert-butoxycarbonyl)-L-lysine in 64 mL CH_2Cl_2 was added 3.68 mL (48.020 mmol, 15 eq)TFA and the mixture was stirred for 3 h at 23°C. The mixture was diluted with toluene (25 mL), concentrated and co-evaporated with toluene (2x 25 mL) under reduced pressure. The residue containing the free amine was dried in HV.

In parallel, 278 µL (3.841 mmol, 1.2 equiv) thionyl chloride and 20 µL of dry DMF were added to a solution of 523 mg (3.201 mmol, 1.0 equiv) 4-azidobenzoic acid in 10.7 mL CH_2Cl_2 at 0°C and the mixture was stirred at 23°C for 4 h. The reaction mixture was concentrated under reduced pressure and dried in HV. The residue was redissolved in 2.6 mL dry dioxane and added dropwise to a mixture of the free amine (described above) in 13.3 mL dioxane and 13.3 mL of aqueous 25w% K_2CO_3 solution. The mixture was then stirred for 41 h at 23°C. The mixture was washed with tert-butylmethylether (3x 25 mL), the aqueous layer was acidified to pH = 2 with concentrated HCl and extracted with $CHCl_3$ (3x 25 mL). The combined organic layers were dried over Na_2SO_4 and concentrated under reduced pressure. The residue was purified by flash-chromatography through silicagel (CH_2Cl_2:MeOH/1:0 – 95:5 – 9:1) yielding 1.214 g (2.365 mmol, 74%) of N^2-(((9H-fluoren-9-yl)methoxy)carbonyl)-N^6-(4-azidobenzoyl)-L-lysine as a white, amorph solid.

N^2-(((9H-fluoren-9-yl)methoxy)carbonyl)-N^6-(4-azidobenzoyl)-L-lysine: ^{1}H-NMR (500 MHz, DMSO-d_6) δ [ppm]: 12.56 (s, 1H), 8.47 (t, *J* = 5.6 Hz, 1H), 7.93 – 7.83 (m, 4H), 7.71 (d, *J* = 7.5 Hz, 2H), 7.63 (d, *J* = 8.0 Hz, 1H), 7.41 (t, *J* = 7.5 Hz, 2H), 7.31 (t, *J* = 7.4 Hz, 2H), 7.22 – 7.11 (m, 2H), 4.32 – 4.13 (m, 3H), 3.98 – 3.86 (m, 1H), 3.24 (q, *J* = 6.5 Hz, 2H), 1.79 – 1.69 (m, 1H), 1.64 (dtd, *J* = 14.2, 9.6, 5.2 Hz, 1H), 1.51 (dhept, *J* = 13.0, 6.7 Hz, 2H), 1.38 (dt, *J* = 15.8, 8.1 Hz, 2H). **^{13}C-NMR** (126 MHz, DMSO-d_6) δ [ppm]: 173.96, 165.07, 156.13, 143.79, 142.03, 140.67, 131.19, 129.00, 127.60, 127.03, 125.24, 120.09, 118.79, 65.58, 53.74, 46.63, 30.45, 28.68, 23.16. **LRMS** (ESI-Quad) [m/z]: 514.3 $[M+H]^+$, **HRMS** (ESI-IT) [m/z]: 514.2076, calculated 514.2085 for $C_{28}H_{28}N_5O_5$ $[M+H]^+$, err [ppm] -1.750.

General procedure A for the solid-supported synthesis of FA-N_3

The solid-phase syntheses of the **FA-N_3-3-11** were carried out manually on a scale of 218 µmol on Rapp 2-chlorotrityl resin (Rapp Polymere, Tübingen, Germany, 1.09 mmol/g) using fritted glas peptide synthesis vessels and Fmoc-protected amino acids. The side chain protections of the amino acids were as follows: Lys(Boc) and Asp(OtBu). Furthermore Fmoc-azidoornithine, (S)-4-((((9H-fluoren-9-yl)methoxy)carbonyl)amino)-5-(tert-butoxy)-5-oxopentanoic acid and N^2-(((9H-fluoren-9-yl)methoxy)carbonyl)-N^6-(4-azidobenzoyl)-L-lysine were utilized. For the loading of the resin a solution the first Fmoc-protected amino acid (218 µmol, 1.0 equiv) Fmoc-Cys(Trt)-OH and 497 µL (4.36 mmol, 20 equiv) NMM in 4 mL dry DMF was reacted shaking it with the resin for 24 h at 23°C. The solvent was removed from the resin and the resin was reacted shaking it for 1 h at 23°C with a solution of 1 mL MeOH and 497 µL (4.36 mmol, 20 equiv) NMM in 4 mL dry DMF. The resin was washed with DMF, CH_2Cl_2 and DMF (each 3x 4 mL, 2 min). Fmoc cleavage was achieved by repeated reaction of the resin shaking it with a mixture of Piperidin in DMF (Pip:DMF/1:4, 3x 5 mL, 10 min) and subsequent washing of the resin with DMF, CH_2Cl_2 and DMF (each 3x 4 mL, 2 min). The coupling of the amino acids was performed shaking with preactivated solutions of Fmoc-protected amino acids (436 µmol, 2.0 eq), 59 mg (436 µmol, 2.0 equiv) HOAt, 144 µL (1.308 mmol, 6.0 equiv) NMM and 166 mg (436 µmol, 2.0 equiv) HATU in 4 mL of dry DMF with coupling times of 4 h at 23°C. Subsequently the resin was washed with DMF, CH_2Cl_2 and DMF (each 3x 4 mL, 2 min). Fmoc cleavage was achieved by repeated reaction of the resin shaking it with a mixture of Piperidin in DMF (Pip:DMF/1:4, 3x 5 mL, 10 min) and subsequent washing of the resin with DMF, CH_2Cl_2 and DMF (each 3x 4 mL, 2 min).

For the attachment of the folic acid unit in parallel with the last amino acid coupling 150 mg (1.308 mmol, 6.0 equiv) N-hydroxysuccinimide and 270 mg (1.308 mmol, 6.0 equiv) DCC, were added to a solution of 577 mg (1.308 mmol, 6.0 equiv) folic acid in 4 mL dry DMSO and stirred under light exclusion for 24 h at 23°C. The resulting suspension was filtered through a Whatman® filter (45 µm) onto the peptide-loaded resin presenting free amino groups (described above) and was reacted shaking it for 24 h at 23°C under light exclusion. The resin was washed with DMF, CH_2Cl_2, DMF, CH_2Cl_2 (each 3x 4 mL, 2 min) and the assembled folic acid derivative was cleaved from the resin by repeated treatment with a 4:1-mixture of CH_2Cl_2:HFIP (3x 5 mL, 10 min). The cleavage solution was concentrated under reduced pressure and the residue treated with an Argon-flow-degassed mixture of TFA:TIPS:H_2O:nPrSH/100:3:3:3 at 23°C (2x 5 mL, 1 h). Upon treatment with ice-cold Et_2O (20 mL) a yellow precipitate formed, which was collected, redissolved in DMSO:H_2O/1:3, filtered through a Whatman® filter (45 µm) and purified by RP prep HPLC (Phenomenex Gemini C18 RP-column 00G-4435-PO-AX, 5 µm, 110 A, 250×21.20 mm, Flow: 9 mL/min,

ACN:H_2O + TFA/ 10:90 + 0.1% → 95:5 + 0.1% in 60 min) yielding after lyophilization the corresponding **FA-N$_3$-3-11** as deep yellow, amorph solids.

FA-N$_3$-3

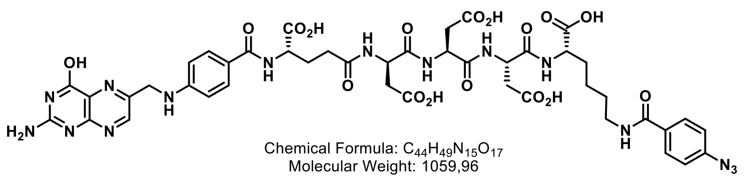

FA-N$_3$-3

Following the general procedure A, 114 mg of **FA-N$_3$-3** as a deep yellow, amorph TFA salt were obtained.

FA-N$_3$-3: ^{1}H-NMR (700 MHz, DMSO-d_6) δ [ppm]: 12.36 (s, 5H), 8.66 (s, 1H), 8.44 (s, 1H), 8.33 – 8.24 (m, 1H), 8.23 (s, 2H), 8.06 – 7.93 (m, 1H), 7.88 (d, *J* = 8.4 Hz, 2H), 7.68 (d, *J* = 8.9 Hz, 1H), 7.66 (dd, *J* = 8.7, 2.1 Hz, 2H), 7.17 (d, *J* = 8.5 Hz, 2H), 7.15 – 6.78 (m, 2H), 6.64 (dd, *J* = 8.8, 4.1 Hz, 2H), 4.62 – 4.41 (m, 6H), 4.31 (td, *J* = 8.7, 7.9, 4.9 Hz, 1H), 4.12 (tt, *J* = 7.9, 4.9 Hz, 1H), 3.26 – 3.16 (m, 2H), 2.82 – 2.58 (m, 4H), 2.56 – 2.50 (m, 2H), 2.34 – 2.16 (m, 2H), 2.10 – 1.83 (m, 2H), 1.76 – 1.68 (m, 1H), 1.66 – 1.55 (m, 1H), 1.54 – 1.44 (m, 2H), 1.38 – 1.27 (m, 2H). **^{13}C-NMR** (126 MHz, DMSO-d_6) δ [ppm]: 174.13, 173.71, 173.25, 172.16, 172.10, 171.88, 171.76, 171.11, 171.01, 170.33, 166.34, 165.15, 160.65, 158.44, 158.24, 158.05, 157.85, 153.44, 150.89, 150.73, 149.18, 148.43, 142.05, 131.22, 129.05, 127.96, 121.30, 118.82, 111.19, 52.01, 51.97, 49.77, 49.47, 45.87, 36.05, 35.99, 35.85, 35.79, 31.93, 31.83, 30.65, 30.28, 28.69, 26.55, 22.77. **LRMS** (ESI-Quad) [m/z]: 1060.4 $[M+H]^+$, **HRMS** (ESI-IT) [m/z]: 530.678558, calculated 530.678819 for $C_{44}H_{51}N_{15}O_{17}$ $[M+2H]^{2+}$, err [ppm] 0.491

FA-N$_3$-4

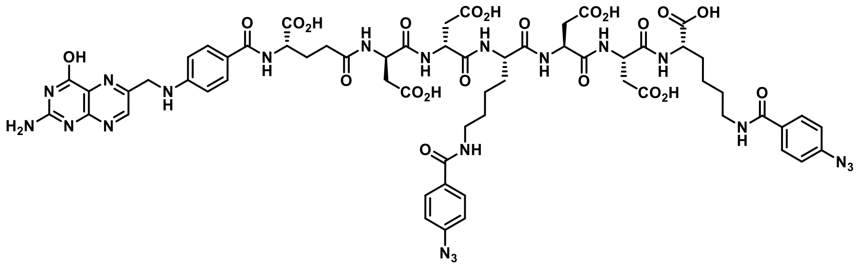

Chemical Formula: $C_{61}H_{69}N_{21}O_{22}$
Molecular Weight: 1448,35
FA-N$_3$-4

Following the general procedure A, 90 mg of **FA-N$_3$-4** as a deep yellow, amorph poly TFA salt were obtained.

FA-N$_3$-4: **^{1}H-NMR** (700 MHz, DMSO-*d*$_6$) δ [ppm]: 12.37 (s, 5H), 8.66 (d, *J* = 2.3 Hz, 1H), 8.46 – 8.36 (m, 2H), 8.31 – 8.11 (m, 3H), 8.10 – 7.91 (m, 2H), 7.87 (dd, *J* = 8.4, 3.3 Hz, 4H), 7.83 – 7.68 (m, 2H), 7.68 – 7.59 (m, 2H), 7.18 – 7.11 (m, 4H), 7.28 – 6.76 (m, 2H), 6.63 (t, *J* = 7.4 Hz, 2H), 4.63 – 4.43 (m, 6H), 4.37 – 3.97 (m, 3H), 3.24 – 3.14 (m, 4H), 2.77 – 2.62 (m, 5H), 2.34 – 2.17 (m, 3H), 2.09 – 2.02 (m, 1H), 1.93 – 1.84 (m, 1H), 1.77 – 1.65 (m, 2H), 1.65 – 1.52 (m, 2H), 1.53 – 1.40 (m, 5H), 1.37 – 1.22 (m, 5H). **^{13}C-NMR** (176 MHz, DMSO-*d*$_6$) δ [ppm]: 174.24, 174.13, 173.76, 173.30, 172.09, 172.06, 171.96, 171.87, 171.79, 171.75, 171.67, 171.60, 171.03, 170.97, 170.67, 170.47, 170.35, 166.38, 165.18, 160.65, 158.30, 158.10, 153.45, 150.74, 149.21, 148.43, 142.05, 131.21, 129.05, 127.96, 118.82, 111.20, 52.95, 52.08, 52.01, 49.97, 49.90, 49.66, 49.64, 49.57, 49.35, 45.88, 35.97, 35.85, 31.88, 31.28, 30.67, 30.29, 28.73, 28.69, 28.55, 26.54, 26.46, 22.83. **LRMS** (ESI-Quad) [m/z]: 1448.7 $[M+H]^+$, **HRMS** (ESI-IT) [m/z]: 724.7536, calculated 724.7536 for $C_{61}H_{71}N_{21}O_{22}$ $[M+2H]^{2+}$, err [ppm] 0.00.

FA-N$_3$-5

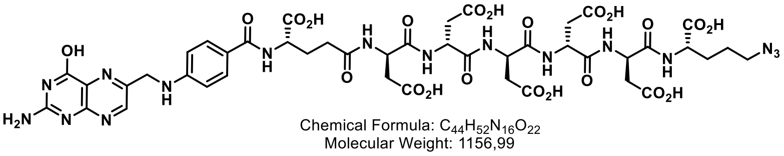

Chemical Formula: $C_{44}H_{52}N_{16}O_{22}$
Molecular Weight: 1156,99
FA-N$_3$-5

Following the general procedure A, 85 mg of **FA-N$_3$-5** as a deep yellow, amorph TFA salt were obtained.

FA-N$_3$-5: **^{1}H-NMR** (700 MHz, DMSO-*d*$_6$) δ [ppm]: 12.37 (s, 7H), 8.69 (s, 1H), 8.24 – 8.12 (m, 2H), 8.08 (d, *J* = 5.7 Hz, 3H), 7.80 (d, *J* = 8.0 Hz, 1H), 7.66 (d, *J* = 8.6 Hz, 2H), 7.44 (s, 1H), 6.64 (d, *J* = 8.7 Hz, 2H), 4.63 – 4.43 (m, 9H), 4.35 – 4.23 (m, 3H), 4.18 (td, *J* = 8.9, 4.9 Hz, 2H), 3.31 (t, *J* = 6.9 Hz, 2H), 2.75 – 2.63 (m, 6H), 2.32

– 2.14 (m, 2H), 2.10 – 2.00 (m, 1H), 1.95 – 1.84 (m, 1H), 1.78 (ddd, *J* = 14.2, 11.2, 6.3 Hz, 1H), 1.65 (dp, *J* = 13.8, 4.9 Hz, 1H), 1.58 – 1.45 (m, 2H). **^{13}C-NMR** (176 MHz, DMSO-*d*$_6$) δ [ppm]: 174.09, 173.74, 173.73, 172.95, 172.93, 171.90, 171.87, 171.85, 171.82, 171.77, 171.57, 170.95, 170.89, 170.54, 170.30, 170.26, 170.21, 166.30, 160.24, 158.60, 158.39, 158.19, 157.98, 153.12, 150.68, 149.90, 148.24, 129.17, 129.02, 127.97, 121.41, 121.36, 118.19, 116.53, 114.87, 113.22, 111.21, 54.91, 52.15, 52.12, 51.40, 50.23, 50.18, 49.68, 49.58, 45.83, 40.43, 40.02, 36.08, 35.85, 31.95, 30.26, 28.14, 26.58, 24.73. **LRMS** (ESI-Quad) [m/z]: 1157.3 [M+H]$^+$, **HRMS** (ESI-IT) [m/z]: 579.179534, calculated 579.179380 for $C_{44}H_{54}N_{16}O_{22}$ [M+2H]$^{2+}$, err [ppm] -0.266.

FA-N$_3$-6

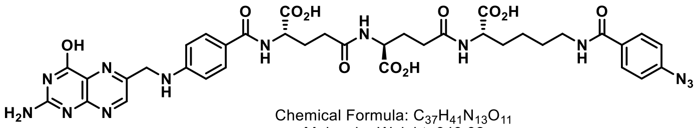

Chemical Formula: $C_{37}H_{41}N_{13}O_{11}$
Molecular Weight: 843,82
FA-N$_3$-6

Following the general procedure A, 60 mg of **FA-N$_3$-6** as of a deep yellow, amorph TFA salt were obtained.

FA-N$_3$-6: **^{1}H-NMR** (500 MHz, DMSO-*d*$_6$) δ [ppm]: 12.50 (s, 3H), 8.66 (d, *J* = 2.7 Hz, 1H), 8.51 – 8.42 (m, 1H), 8.26 – 7.90 (m, 3H), 7.87 (dd, *J* = 8.7, 2.0 Hz, 2H), 7.67 (dd, *J* = 8.3, 6.1 Hz, 2H), 7.18 (dd, *J* = 8.8, 2.3 Hz, 2H), 7.08 (s, 2H), 6.64 (dd, *J* = 8.8, 4.1 Hz, 2H), 4.50 (s, 2H), 4.47 – 4.27 (m, 1H), 4.14 (tt, *J* = 9.5, 4.8 Hz, 2H), 3.26 – 3.16 (m, 2H), 2.26 (ddd, *J* = 21.8, 15.0, 7.2 Hz, 2H), 2.16 (dt, *J* = 15.7, 7.3 Hz, 2H), 2.09 – 1.86 (m, 3H), 1.80 – 1.62 (m, 2H), 1.62 – 1.54 (m, 1H), 1.50 (s, 2H), 1.33 (s, 2H). **^{13}C-NMR** (176 MHz, DMSO-*d*$_6$) δ [ppm]: 174.13, 173.79, 173.76, 173.75, 173.43, 173.40, 173.13, 171.80, 171.79, 171.68, 171.39, 171.37, 166.29, 165.10, 160.64, 158.18, 157.99, 153.43, 150.71, 149.16, 148.42, 142.05, 131.20, 129.06, 129.01, 127.95, 121.34, 118.82, 111.17, 52.62, 52.24, 52.00, 51.74, 51.49, 51.36, 45.87, 31.88, 31.71, 31.46, 31.41, 31.32, 30.71, 30.47, 30.43, 28.72, 28.70, 28.68, 27.20, 26.98, 26.96, 26.68, 26.51, 22.96. **LRMS** (ESI-Quad) [m/z]: 844.3 [M+H]$^+$, **HRMS** (ESI-IT) [m/z]: 844.311777, calculated 844.312126 for $C_{37}H_{42}N_{13}O_{11}$ [M+H]$^+$, err [ppm] 0.413

FA-N$_3$-7

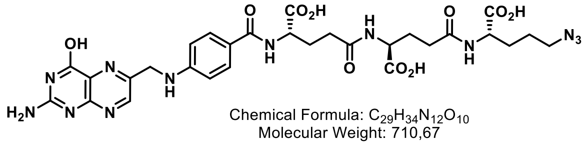

FA-N$_3$-7

Following the general procedure A, 52 mg of **FA-N$_3$-7** as a deep yellow, amorph TFA salt were obtained.

FA-N$_3$-7: **^{1}H-NMR** (500 MHz, DMSO-*d*$_6$) δ [ppm]: 12.54 (s, 3H), 8.69 (s, 1H), 8.26 – 8.17 (m, 1H), 8.13 (ddd, *J* = 18.4, 7.7, 3.5 Hz, 2H), 7.67 (dd, *J* = 8.1, 6.1 Hz, 2H), 7.47 (s, 2H), 6.64 (dd, *J* = 8.8, 3.5 Hz, 2H), 4.52 (s, 2H), 4.49 – 4.37 (m, 1H), 4.35 – 4.25 (m, 1H), 4.21 – 4.09 (m, 2H), 3.31 (t, *J* = 6.5 Hz, 2H), 2.27 (ddt, *J* = 20.7, 13.2, 6.9 Hz, 2H), 2.22 – 2.11 (m, 2H), 2.02 – 1.85 (m, 3H), 1.80 – 1.66 (m, 2H), 1.65 – 1.48 (m, 3H). **^{13}C-NMR** (176 MHz, DMSO-*d*$_6$) δ [ppm]: 174.17, 174.14, 173.80, 173.76, 173.45, 173.25, 173.14, 172.91, 171.80, 171.66, 171.40, 166.39, 166.29, 161.02, 160.24, 158.77, 158.56, 158.36, 158.16, 153.13, 150.69, 149.91, 148.24, 129.08, 129.03, 129.00, 127.97, 121.41, 116.65, 114.99, 111.21, 52.63, 52.26, 51.99, 51.47, 51.31, 50.26, 50.24, 50.15, 49.95, 45.84, 31.88, 31.68, 31.45, 31.38, 31.32, 30.48, 30.44, 28.35, 28.23, 27.20, 27.10, 27.04, 26.97, 26.69, 26.50, 24.91, 24.77. **LRMS** (ESI-Quad) [m/z]: 711.3 $[M+H]^+$, **HRMS** (ESI-IT) [m/z]: 711.2589, calculated 711.2594 for $C_{29}H_{35}N_{12}O_{10}$ $[M+H]^+$, err [ppm] 0.600.

FA-N$_3$-8

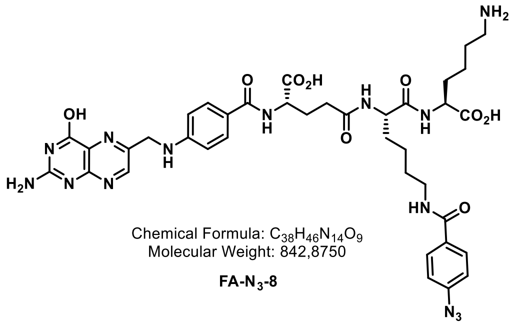

FA-N$_3$-8

Following the general procedure A, 49 mg of **FA-N$_3$-8** as a deep yellow, amorph TFA salt were obtained.

FA-N$_3$-8: **^{1}H-NMR** (700 MHz, DMSO-*d*$_6$) δ [ppm]: 12.61 (s, 2H), 11.52 (s, 1H), 8.68 – 8.63 (m, 1H), 8.45 (q, *J* = 6.9, 6.3 Hz, 1H), 8.26 – 8.13 (m, 1H), 8.17 – 8.05 (m, 1H), 8.02 – 7.92 (m, 1H), 7.90 – 7.83 (m, 2H), 7.66 (dd, *J* = 8.8, 1.8 Hz, 2H), 7.62 (s, 3H), 7.26 – 7.13 (m, 2H), 6.95 (s, 2H), 6.64 (d, *J* = 8.9 Hz, 2H), 4.49 (s, 2H), 4.35 – 4.22 (m, 2H), 4.22 – 4.12 (m, 1H), 3.26 – 3.16 (m, 3H), 2.80 – 2.70 (m, 2H), 2.35 – 2.19 (m, 2H), 2.11 – 1.82 (m, 2H), 1.73 – 1.65 (m, 1H), 1.65 – 1.54 (m, 2H), 1.55 – 1.42 (m, 5H), 1.36 – 1.20 (m, 5H). **^{13}C-NMR** (176 MHz, DMSO-*d*$_6$) δ [ppm]: 173.99, 173.81, 173.77, 173.41, 173.38, 173.32, 172.10, 171.83, 171.80,

171.75, 171.55, 171.45, 166.70, 166.36, 166.29, 165.11, 158.18, 158.00, 157.81, 157.63, 153.66, 150.88, 150.77, 150.73, 148.71, 148.52, 142.07, 131.15, 128.99, 127.92, 121.33, 121.24, 120.91, 118.82, 117.60, 115.91, 111.15, 64.90, 52.55, 52.30, 52.07, 51.99, 51.31, 45.88, 39.87, 38.63, 32.03, 31.81, 31.79, 31.25, 30.47, 30.45, 30.34, 30.28, 28.82, 28.80, 28.65, 26.74, 26.59, 26.44, 26.42, 26.31, 26.28, 22.89, 22.83, 22.22, 22.20, 22.07, 15.16. **LRMS** (ESI-Quad) [m/z]: 843.4 $[M+H]^+$, **HRMS** (ESI-IT) [m/z]: 843.364120, calculated 843.364496 for $C_{38}H_{47}N_{14}O_9$ $[M+H]^+$, err [ppm] 0.445.

FA-N_3-9

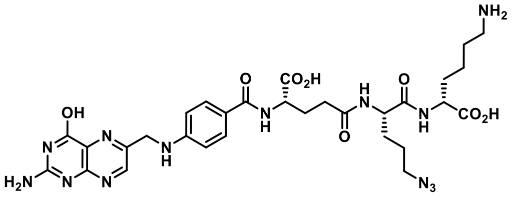

Chemical Formula: $C_{30}H_{39}N_{13}O_8$
Molecular Weight: 709,73
FA-N_3-9

Following the general procedure A, 53 mg of **FA-N_3-9** as a deep yellow, amorph TFA salt were obtained.

FA-N_3-9: **^{1}H-NMR** (700 MHz, DMSO-d_6) δ [ppm]: 12.60 (s, 2H), 11.50 (s, 1H), 8.70 – 8.59 (m, 1H), 8.28 – 8.06 (m, 2H), 8.02 (td, *J* = 14.9, 13.9, 8.2 Hz, 1H), 7.67 – 7.65 (m, 2H), 7.62 (s, 3H), 6.94 (s, 2H), 6.64 (d, *J* = 8.8 Hz, 2H), 4.49 (s, 2H), 4.39 – 4.27 (m, 2H), 4.22 – 4.10 (m, 1H), 3.29 (q, *J* = 6.7 Hz, 2H), 2.80 – 2.70 (m, 2H), 2.35 – 2.19 (m, 2H), 2.11 – 1.84 (m, 2H), 1.80 – 1.56 (m, 3H), 1.57 – 1.44 (m, 5H), 1.37 – 1.21 (m, 2H).**^{13}C-NMR** (176 MHz, DMSO-d_6) δ [ppm]: 174.14, 173.99, 173.82, 173.76, 173.37, 173.34, 173.28, 173.27, 172.12, 171.80, 171.76, 171.61, 171.45, 171.41, 171.21, 171.12, 166.72, 166.31, 166.28, 157.97, 157.79, 153.72, 150.79, 150.74, 148.64, 129.14, 129.09, 129.00, 127.93, 121.36, 121.25, 120.93, 117.88, 116.18, 111.16, 107.52, 64.91, 52.05, 52.00, 51.79, 51.41, 51.35, 50.42, 50.32, 50.30, 48.60, 45.90, 38.63, 38.60, 31.81, 31.77, 30.57, 30.49, 30.46, 30.33, 30.28, 29.60, 29.42, 28.95, 26.72, 26.59, 26.44, 26.43, 26.30, 24.80, 24.74, 22.25, 22.23, 22.10, 15.17. **LRMS** (ESI-Quad) [m/z]: 710.3 $[M+H]^+$, **HRMS** (ESI-IT) [m/z]: 710.311272, calculated 710.311732 for $C_{30}H_{40}N_{13}O_8$ $[M+H]^+$, err [ppm] 0.647.

FA-N$_3$-10

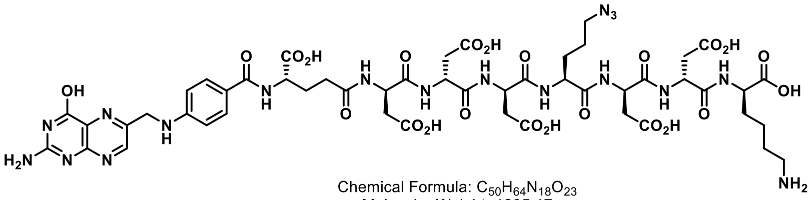

Chemical Formula: $C_{50}H_{64}N_{18}O_{23}$
Molecular Weight: 1285,17
FA-N$_3$-10

Following the general procedure A, 93 mg of **FA-N$_3$-10** as a deep yellow, amorph TFA salt were obtained.

FA-N$_3$-10: ^{1}**H-NMR** (700 MHz, DMSO-d_6) δ [ppm]: 12.38 (s, 7H), 11.50 (s, 1H), 8.65 (s, 1H), 8.50 – 7.72 (m, 8H), 7.66 (d, *J* = 6.8 Hz, 2H), 7.60 (s, 3H), 6.94 (s, 2H), 6.64 (d, *J* = 8.8 Hz, 2H), 4.61 – 4.39 (m, 7H), 4.35 – 4.26 (m, 1H), 4.27 – 4.07 (m, 2H), 3.32 – 3.25 (m, 2H), 2.82 – 2.65 (m, 8H), 2.58 – 2.51 (m, 2H), 2.34 – 2.16 (m, 2H), 2.11 – 1.82 (m, 2H), 1.77 – 1.65 (m, 2H), 1.65 – 1.42 (m, 6H), 1.38 – 1.28 (m, 2H). ^{13}**C-NMR** (176 MHz, DMSO-d_6) δ [ppm]: 174.08, 173.74, 173.72, 173.20, 173.18, 171.92, 171.89, 171.73, 171.70, 170.51, 170.36, 166.35, 160.89, 158.18, 157.99, 157.81, 157.62, 153.66, 150.77, 148.73, 148.53, 129.16, 129.01, 127.94, 121.30, 121.25, 117.56, 115.87, 111.17, 51.60, 50.33, 49.92, 49.69, 49.47, 45.89, 40.43, 38.69, 36.21, 35.97, 35.72, 35.67, 31.92, 31.88, 30.28, 30.25, 28.89, 26.57, 26.47, 26.41, 26.37, 24.40, 22.08. **LRMS** (ESI-Quad) [m/z]: 1285.6 $[M+H]^{+}$, **HRMS** (ESI-IT) [m/z]: 643.226691, calculated 643.226862 for $C_{50}H_{66}N_{18}O_{23}$ $[M+2H]^{2+}$, err [ppm] 0.265.

FA-N$_3$-11

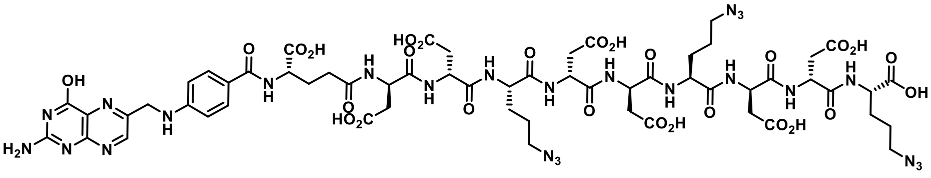

Chemical Formula: $C_{58}H_{73}N_{25}O_{27}$
Molecular Weight: 1552,37
FA-N$_3$-11

Following the general procedure A, 53 mg of **FA-N$_3$-11** as a deep yellow, amorph TFA salt were obtained.

FA-N$_3$-11: ^{1}**H-NMR** (700 MHz, DMSO-d_6) δ [ppm]: 12.40 (s, 8H), 11.15 (s, 1H), 8.74 – 8.54 (m, 1H), 8.41 – 8.03 (m, 8H), 7.92 – 7.71 (m, 2H), 7.66 (d, *J* = 8.6 Hz, 2H), 7.32 – 6.80 (m, 2H), 6.64 (d, *J* = 8.6 Hz, 2H), 4.64 – 4.50 (m, 7H), 4.50 (s, 2H), 4.35 – 4.28 (m, 1H), 4.29 – 4.10 (m, 4H), 3.34 – 3.23 (m, 6H), 2.81 – 2.64 (m, 7H), 2.58 – 2.53 (m, 2H), 2.33 – 2.17 (m, 1H), 2.09 – 1.86 (m, 2H), 1.81 – 1.74 (m, 1H), 1.73 – 1.61 (m, 4H), 1.59 – 1.41 (m, 9H). ^{13}**C-NMR** (176 MHz, DMSO-d_6) δ [ppm]: 173.75, 173.73, 172.97, 171.92, 171.81, 171.69, 171.66, 171.55, 171.03, 170.53, 170.44, 170.38, 170.30, 166.48, 166.33, 160.70, 158.33, 158.15,

157.95, 157.74, 153.58, 153.50, 150.91, 150.74, 149.07, 148.45, 129.20, 129.02, 127.95, 121.30, 117.16, 111.18, 52.08, 51.57, 51.36, 50.33, 50.18, 49.97, 49.62, 49.38, 49.32, 47.76, 45.87, 45.75, 36.25, 36.05, 32.35, 31.84, 30.25, 29.09, 28.96, 28.16, 27.59, 26.52, 25.99, 25.23, 24.72, 24.38, 23.74, 23.43. **LRMS** (ESI-Quad) [m/z]: 1552.5 $[M+H]^+$, **HRMS** (ESI-IT) [m/z]: 776.762091, calculated 776.762663 for $C_{58}H_{75}N_{25}O_{27}$ $[M+2H]^{2+}$, err [ppm] 0.736.

Synthesis of FA-SH-1

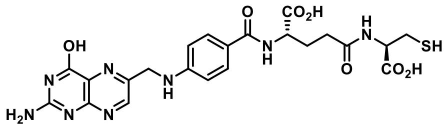

Chemical Formula: $C_{22}H_{24}N_8O_7S$
Molecular Weight: 544,54
FA-SH-1

The solid-phase synthesis of the **FA-SH-1** was carried out manually on a scale of 484 µmol on Rapp 2-chlorotrityl resin (Rapp Polymere, Tübingen, Germany, 1.21 mmol/g) using fritted glas peptide synthesis vessels. For the loading of the resin a solution 567 mg (968 µmol, 2.0 equiv) Fmoc-Cys(Trt)-OH and 2.12 mL (19.36 mmol, 20 equiv) NMM in 7.5 mL dry DMF was reacted shaking it with the resin for 24 h at 23°C. The solvent was removed from the resin and the resin was reacted shaking it for 1 h at 23°C with a solution of 1 mL MeOH and 2.12 mL (19.36 mmol, 20 equiv) NMM in 7.5 mL dry DMF. The resin was washed with DMF, CH_2Cl_2 and DMF (each 3x 10 mL, 2 min). Fmoc cleavage achieved by repeated reaction of the resin shaking it with a mixture of Piperidin in DMF (Pip:DMF/1:4, 3x 10 mL, 10 min) and subsequent washing of the resin with DMF, CH_2Cl_2 and DMF (each 3x 10 mL, 2 min).

In parallel, 220 mg (1.94 mmol, 4.0 equiv) N-hydroxysuccinimide and 400 mg (1.94 mmol, 4.0 equiv) DCC, were added to a solution of 853 mg (1.94 mmol, 4.0 equiv) folic acid in 7.5 M dry DMSO and stirred under light exclusion for 24 h at 23°C. The resulting suspension was filtered through a Whatman® filter (45 µm) onto the Cys-loaded resin presenting free amino groups (described above) and was reacted shaking it for 24 h at 23°C under light exclusion. The resin was washed with DMF, CH_2Cl_2, DMF, CH_2Cl_2 (each 3x 10 mL, 2 min) and the assembled folic acid derivative was cleaved from the resin by repeated treatment with an Argon-flow-degassed mixture of TFA:TIPS:H_2O:nPrSH/100:3:3:3 at 23°C (2x 5 mL, 1 h). Upon treatment with ice-cold Et_2O (20 mL) a yellow precipitate formed, which was collected, redissolved in DMSO:H_2O/1:3, filtered through a Whatman® filter (45 µm) and purified by RP prep HPLC (Thermo Fisher Scientific BDS Hypersil C18 RP-column 28105-259370, 5 µm, 250x30 mm, Flow: 25 mL/min, ACN:H_2O + TFA/ 10:90 + 0.1% → 95:5 + 0.1% in 60 min) yielding after lyophilization 155.0 mg (285.6 µmol, 59%) of **FA-SH** as a deep-yellow solid.

FA-SH-1: **^{1}H-NMR** (700 MHz, DMSO-d_6) δ [ppm]: 12.62 (s, 3H), 8.19 (d, *J* = 7.7 Hz, 1H), 8.16 (d, *J* = 8.0 Hz, 1H), 7.66 (d, *J* = 8.8 Hz, 2H), 7.46 (s, 2H), 6.65 (d, *J* = 8.8 Hz, 2H), 4.52 (s, 2H), 4.39 (td, *J* = 7.6, 4.7 Hz, 1H), 4.32 (ddd, *J* = 9.8, 7.8, 4.7 Hz, 1H), 2.83 (ddd, *J* = 13.3, 8.4, 4.6 Hz, 1H), 2.76 – 2.66 (m, 1H), 2.44 (t, *J* = 8.5 Hz, 1H), 2.28 (t, *J* = 7.7 Hz, 2H), 2.08 (dtd, *J* = 12.3, 7.9, 4.7 Hz, 1H), 1.91 (ddd, *J* = 13.7, 9.7, 7.2 Hz, 1H).. **^{13}C-NMR** (176 MHz, DMSO-d_6) δ [ppm]: 173.82, 171.78, 171.68, 166.33, 160.36, 153.24, 150.68, 149.84, 148.28, 129.02, 127.98, 121.45, 111.24, 54.34, 52.05, 45.86, 31.77, 26.56, 25.60. **LRMS** (ESI-Quad) [m/z]: 1086.5 $[M+H]^+$, **HRMS** (ESI-IT) [m/z]: 1086.441323, calculated 1086.442337 for $C_{22}H_{24}N_8O_7S$ $[M+H]^+$, err [ppm] -0.933.

Synthesis of LHRH derivatives

General procedure B for the solid-supported synthesis of LHRH-derivatives

Solid-phase synthesis of the peptide was carried out on a scale of 150 µmol with a Syro Multiple Peptide Synthesizer (MultiSynTech, Witten, Germany) on Rapp S RAM resin (Rapp Polymere, Tübingen, Germany, 0.23 mmol/g). Fmoc-protected amino acids were coupled to the resin using a fivefold excess of Fmoc-protected amino acid:TBTU: DiPEA/1:1:2 and coupling times of 1 h at 23°C. The side chain protections of the amino acids were as follows: Arg: Pbf; His: Trt; Ser and Tyr: t-Bu; Trp: Boc. The peptide was cleaved from the resin and deprotected by a treatment with TFA:TIPS:H_2O/95:3:2 (10 ml/g resin) over 3 h at 23°C. After precipitation with t-butylmethyl ether, the resulting crude peptide was purified by reparative HPLC (RP-18) with water/acetonitrile gradients containing 0.1% TFA and characterized by analytical HPLC and MALDI-MS.

Synthesis of LHRH

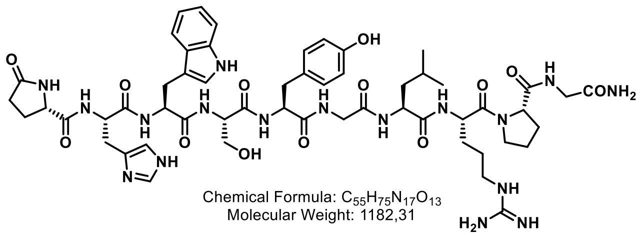

LHRH

Following the general procedure B, 68 mg of **LHRH** as a white, amorph TFA salt were obtained.

LHRH: **^{1}H-NMR** (700 MHz, DMSO-d_6) δ [ppm]: 14.06 (s, 2H), 10.79 (d, J = 2.4 Hz, 1H), 9.16 (s, 1H), 8.92 (s, 1H), 8.28 (d, J = 7.6 Hz, 1H), 8.26 – 8.23 (m, 2H), 8.20 (d, J = 7.5 Hz, 1H), 8.13 (d, J = 8.3 Hz, 1H), 8.07 (d, J = 7.6 Hz, 1H), 7.95 (d, J = 7.8 Hz, 1H), 7.90 (d, J = 8.2 Hz, 1H), 7.68 (s, 1H), 7.61 (d, J = 7.9 Hz, 1H), 7.48 (d, J = 5.4 Hz, 1H), 7.31 (d, J = 8.1 Hz, 1H), 7.27 (s, 1H), 7.13 (dd, J = 6.4, 2.2 Hz, 2H), 7.08 (d, J = 2.2 Hz, 1H), 7.05 – 7.01 (m, 3H), 6.94 – 6.90 (m, 1H), 6.80 (s, 0H), 6.64 – 6.60 (m, 2H), 5.03 (s, 1H), 4.62 (dtd, J = 24.4, 8.2, 4.9 Hz, 2H), 4.46 (qd, J = 9.3, 8.8, 5.9 Hz, 2H), 4.34 (ddt, J = 13.6, 7.7, 5.7 Hz, 2H), 4.27 (dd, J = 8.4, 4.9 Hz, 1H), 3.97 (dd, J = 8.8, 4.2 Hz, 1H), 3.73 (d, J = 5.7 Hz, 2H), 3.68 (dt, J = 9.5, 6.5 Hz, 1H), 3.65 – 3.50 (m, 5H), 3.15 (dd, J = 15.0, 4.4 Hz, 1H), 3.11 – 3.06 (m, 1H), 3.03 (dd, J = 15.4, 5.4 Hz, 1H), 2.99 – 2.93 (m, 2H), 2.89 (dd, J = 15.2, 8.3 Hz, 1H), 2.73 (dd, J = 14.1, 8.6 Hz, 1H), 2.18 – 2.11 (m, 1H), 2.10 – 1.99 (m, 3H), 1.98 – 1.89 (m, 1H), 1.81 (tdd, J = 9.5, 5.2, 2.5 Hz, 2H), 1.70 (ddt, J = 13.0, 9.1, 5.0 Hz, 2H), 1.58 (dq, J = 8.6, 6.5 Hz, 1H), 1.54 – 1.48 (m, 2H), 1.45 – 1.37 (m, 2H), 0.87 (d, J = 6.6 Hz, 3H), 0.84 (d, J = 6.6 Hz, 3H). **^{13}C-NMR** (176 MHz, DMSO-d_6) δ [ppm]: 177.42, 172.44, 171.85, 171.81, 171.57, 171.24, 170.98, 169.90, 169.76, 169.55,

168.29, 157.99, 157.81, 157.63, 157.46, 156.63, 155.77, 136.01, 133.76, 130.09, 129.09, 127.55, 127.28, 123.67, 120.82, 118.54, 118.18, 116.92, 114.84, 111.20, 109.66, 73.11, 69.76, 65.60, 63.45, 61.71, 59.75, 58.19, 55.39, 55.09, 54.36, 53.26, 51.23, 50.66, 50.06, 48.71, 46.93, 42.05, 41.95, 41.78, 41.03, 40.57, 36.59, 29.07, 28.10, 27.71, 27.10, 26.82, 24.96, 24.57, 24.52, 24.08, 23.90, 23.24, 23.12, 21.49, 21.41, 20.43, 7.58. **LRMS** (MALDI) [m/z]: 1182.5 $[M+H]^+$, **HRMS** (ESI-IT) [m/z]: 1182.5802, calculated 1182.5803 for $C_{55}H_{76}N_{17}O_{13}$ $[M+H]^+$, err [ppm] 0.1.

Synthesis of L-Orn-N$_3$-LHRH

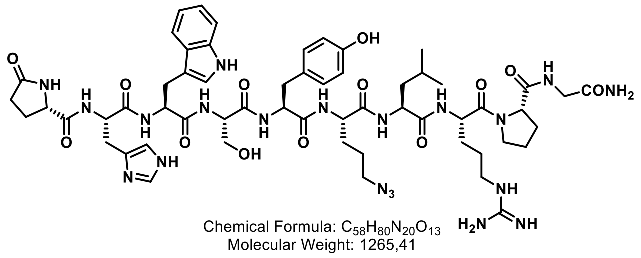

Following the general procedure B, 78 mg of **L-N$_3$-Orn-LHRH** as a white, amorph TFA salt were obtained.

L-Orn-N$_3$-LHRH: **^{1}H-NMR** (700 MHz, DMSO-d_6) δ [ppm]: 14.03 (s, 1H), 13.99 (s, 1H), 10.79 (d, J = 2.4 Hz, 1H), 9.16 (s, 1H), 8.93 (s, 1H), 8.29 (d, J = 7.6 Hz, 1H), 8.23 (t, J = 5.9 Hz, 1H), 8.21 (d, J = 7.4 Hz, 1H), 8.13 (d, J = 8.3 Hz, 1H), 8.11 (d, J = 8.3 Hz, 1H), 8.09 (d, J = 8.3 Hz, 1H), 8.05 (d, J = 7.6 Hz, 1H), 7.99 (d, J = 7.8 Hz, 1H), 7.68 (s, 1H), 7.62 (d, J = 8.0 Hz, 1H), 7.42 (d, J = 6.2 Hz, 1H), 7.35 – 7.29 (m, 1H), 7.27 (s, 1H), 7.14 (s, 1H), 7.12 (d, J = 2.4 Hz, 1H), 7.08 (d, J = 2.3 Hz, 1H), 6.91 (ddd, J = 7.9, 6.9, 1.0 Hz, 1H), 6.66 – 6.61 (m, 2H), 5.03 (s, 1H), 4.64 (td, J = 8.0, 4.5 Hz, 1H), 4.60 (td, J = 8.4, 5.4 Hz, 1H), 4.51 (q, J = 7.7 Hz, 1H), 4.45 (q, J = 7.4, 6.9 Hz, 1H), 4.36 – 4.30 (m, 3H), 4.27 (dd, J = 8.2, 4.9 Hz, 1H), 3.99 – 3.95 (m, 1H), 3.70 (dt, J = 9.9, 6.5 Hz, 1H), 3.64 – 3.55 (m, 4H), 3.54 – 3.49 (m, 2H), 3.24 (td, J = 6.8, 3.5 Hz, 2H), 3.17 – 3.13 (m, 1H), 3.08 (p, J = 6.5 Hz, 1H), 3.03 (dd, J = 15.3, 5.2 Hz, 1H), 2.98 (dd, J = 14.9, 9.1 Hz, 1H), 2.89 (dd, J = 15.1, 7.7 Hz, 2H), 2.73 (dd, J = 13.8, 8.6 Hz, 1H), 2.53 – 2.51 (m, 1H), 2.19 – 2.12 (m, 1H), 2.11 – 2.00 (m, 2H), 1.93 (td, J = 13.5, 12.2, 7.7 Hz, 1H), 1.80 (td, J = 12.3, 6.1 Hz, 2H), 1.74 – 1.66 (m, 2H), 1.63 – 1.42 (m, 4H), 1.41 – 1.31 (m, 2H), 0.86 (d, J = 6.6 Hz, 3H), 0.82 (d, J = 6.5 Hz, 3H). **^{13}C-NMR** (176 MHz, DMSO-d_6) δ [ppm]: 177.43, 172.48, 171.88, 171.84, 171.55, 171.00, 170.74, 170.71, 169.98, 169.71, 169.55, 158.13, 157.94, 157.76, 157.57, 156.60, 155.84, 136.02, 133.77, 130.10, 127.45, 127.30, 123.69, 120.85, 118.56, 118.19, 117.50, 116.93, 115.81, 114.85, 111.21, 109.65, 61.75, 59.73, 55.41, 54.96, 54.49, 53.27, 51.85, 51.25, 50.55, 50.27, 50.14, 46.97, 41.96, 40.82, 40.61, 36.93, 29.75, 29.09, 28.09, 27.06, 24.97, 24.59, 24.53, 24.47,

24.16, 23.21, 21.18. **LRMS** (MALDI) [m/z]: 1256.6 $[M+H]^+$, **HRMS** (ESI-IT) [m/z]: 633.3182, calculated 633.3180 for $C_{58}H_{82}N_{20}O_{13}$ $[M+2H]^{2+}$, err [ppm] -0.300.

Synthesis of D-Orn-N$_3$-LHRH

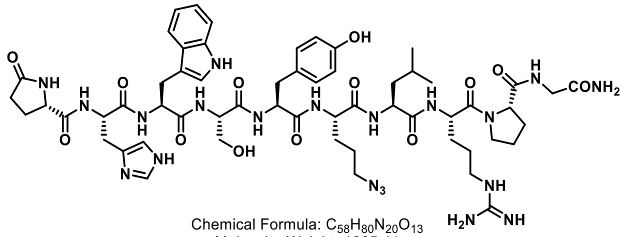

Following the general procedure B, 77 mg of **D-N$_3$-Orn-LHRH** as a white, amorph TFA salt were obtained.

D- Orn-N$_3$-LHRH: **^{1}H-NMR** (700 MHz, DMSO-*d*$_6$) δ [ppm]: 14.08 (s, 2H), 10.79 (d, *J* = 2.5 Hz, 1H), 9.17 (s, 1H), 8.93 (d, *J* = 1.5 Hz, 1H), 8.29 (d, *J* = 7.6 Hz, 1H), 8.23 (t, *J* = 5.9 Hz, 1H), 8.20 (d, *J* = 7.4 Hz, 1H), 8.14 (d, *J* = 8.3 Hz, 1H), 8.11 (d, *J* = 8.2 Hz, 1H), 8.09 (d, *J* = 8.1 Hz, 1H), 8.05 (d, *J* = 7.6 Hz, 1H), 8.00 (d, *J* = 7.8 Hz, 1H), 7.68 (s, 1H), 7.62 (dd, *J* = 7.9, 1.1 Hz, 1H), 7.49 (q, *J* = 6.1 Hz, 1H), 7.35 (s, 0H), 7.31 (dd, *J* = 8.0, 0.9 Hz, 1H), 7.27 (d, *J* = 1.3 Hz, 1H), 7.14 (d, *J* = 2.2 Hz, 1H), 7.12 (d, *J* = 2.4 Hz, 1H), 7.08 (s, 1H), 7.06 – 7.02 (m, 3H), 6.91 (ddd, *J* = 7.9, 6.9, 1.0 Hz, 1H), 6.80 (s, 1H), 6.64 – 6.61 (m, 2H), 5.05 (s, 1H), 4.64 (ddd, *J* = 9.1, 7.7, 4.5 Hz, 1H), 4.60 (td, *J* = 8.3, 5.3 Hz, 1H), 4.51 (td, *J* = 8.0, 5.8 Hz, 1H), 4.45 (q, *J* = 7.4, 6.9 Hz, 1H), 4.38 – 4.30 (m, 3H), 4.26 (dd, *J* = 8.2, 4.9 Hz, 1H), 3.97 (ddd, *J* = 8.7, 4.2, 1.1 Hz, 1H), 3.70 (dt, *J* = 9.8, 6.5 Hz, 1H), 3.64 – 3.55 (m, 3H), 3.54 – 3.49 (m, 2H), 3.24 (td, *J* = 6.8, 3.6 Hz, 2H), 3.15 (d, *J* = 10.6 Hz, 1H), 3.07 (dt, *J* = 12.3, 6.5 Hz, 2H), 3.03 (dd, *J* = 15.3, 5.2 Hz, 1H), 2.98 (dd, *J* = 14.9, 9.1 Hz, 1H), 2.93 – 2.86 (m, 2H), 2.73 (dd, *J* = 13.8, 8.5 Hz, 1H), 2.18 – 2.12 (m, 1H), 2.11 – 2.06 (m, 1H), 2.06 – 1.99 (m, 2H), 1.96 – 1.90 (m, 1H), 1.80 (ddt, *J* = 12.8, 10.7, 6.2 Hz, 2H), 1.73 – 1.66 (m, 2H), 1.64 – 1.41 (m, 7H), 1.36 (dddd, *J* = 25.3, 13.5, 9.5, 5.7 Hz, 2H), 0.86 (d, *J* = 6.6 Hz, 3H), 0.82 (d, *J* = 6.6 Hz, 3H). **^{13}C-NMR** (176 MHz, DMSO-*d*$_6$) δ [ppm]: 177.43, 172.48, 171.89, 171.84, 171.56, 171.01, 170.75, 170.71, 170.00, 169.72, 169.57, 158.42, 158.23, 158.04, 157.84, 156.66, 155.85, 136.03, 133.77, 130.10, 129.11, 127.46, 127.30, 123.70, 120.85, 118.56, 118.19, 117.18, 116.94, 115.51, 114.86, 111.21, 109.66, 61.75, 59.74, 55.41, 54.96, 54.50, 53.28, 51.86, 51.26, 50.56, 50.28, 50.16, 46.97, 41.96, 40.81, 40.61, 36.94, 29.75, 29.09, 29.05, 28.08, 27.75, 27.07, 24.98, 24.59, 24.54, 24.47, 24.16, 23.21, 21.18. **LRMS** (MALDI) [m/z]: 1265.7 $[M+H]^+$, **HRMS** (ESI-IT) [m/z]: 633.3182, calculated 633.3180 for $C_{58}H_{82}N_{20}O_{13}$ $[M+2H]^{2+}$, err [ppm] -0.300.

Synthesis of D-Orn-N_3-Gose

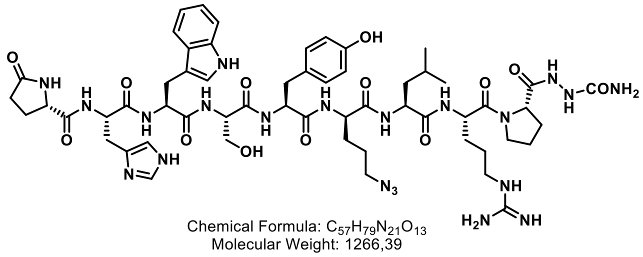

Rapp S RAM resin (100 µmol, 0.23 mmol/g) was treated for 10 min with 20% piperidine in DMF at 23°C to remove the Fmoc group, followed by washing with DMF (5x 5 mL, 2 min) and DCM (5x 5 mL, 2 min). 162 mg (1.0 mmol, 10.0 equiv) carbonyldiimidazol in dry DCM (3 mL) were added and the mixture was incubated for 3 h at 23°C with shaking, followed by washing with DCM (5x 5 mL, 2 min) and DMF (5x 5 mL, 2 min). The resin was then treated with hydrazine in DMF (5 mL, ca. 2 M)[ix] for 1 h at 23°C.[x] Afterwards the resin was washed with DMF (5x 5 mL, 2 min) and the peptide was assembled on a Syro Multiple Peptide Synthesizer, cleaved, purified and characterized as described in the general procedure B, yielding 20 mg of **D-Orn-N_3-Gose** as a TFA salt.

D-Orn-N_3-Gose: ^{1}H-NMR (700 MHz, DMSO-d_6) δ [ppm]: 14.09 (s, 2H), 10.80 (d, *J* = 2.4 Hz, 1H), 10.68 (s, 1H), 9.76 (d, *J* = 21.4 Hz, 1H), 9.18 (s, 1H), 8.93 (d, *J* = 1.4 Hz, 1H), 8.29 (d, *J* = 7.6 Hz, 2H), 8.23 (d, *J* = 7.3 Hz, 1H), 8.14 (d, *J* = 8.3 Hz, 1H), 8.10 (t, *J* = 7.7 Hz, 2H), 8.06 (d, *J* = 7.5 Hz, 1H), 8.00 (d, *J* = 7.8 Hz, 1H), 7.80 (s, 1H), 7.68 (s, 1H), 7.62 (d, *J* = 8.0 Hz, 1H), 7.51 (t, *J* = 5.5 Hz, 1H), 7.33 – 7.29 (m, 1H), 7.27 (d, *J* = 1.3 Hz, 1H), 7.19 (s, 1H), 7.14 – 7.09 (m, 2H), 7.07 – 7.00 (m, 4H), 6.91 (ddd, *J* = 7.9, 6.9, 1.0 Hz, 1H), 6.65 – 6.62 (m, 2H), 5.89 (s, 2H), 5.05 (s, 1H), 4.64 (td, *J* = 8.0, 4.4 Hz, 1H), 4.59 (dd, *J* = 8.4, 5.3 Hz, 1H), 4.51 (td, *J* = 8.1, 5.8 Hz, 1H), 4.46 – 4.41 (m,3), 4.34 (ddt, *J* = 15.8, 7.8, 5.5 Hz, 1H), 4.19 (s, 1H), 3.97 (ddd, *J* = 8.7, 4.2, 1.1 Hz, 1H), 3.73 (d, *J* = 6.9 Hz, 1H), 3.59 (dd, *J* = 10.6, 6.1 Hz, 2H), 3.51 (dp, *J* = 11.1, 6.1, 5.3 Hz, 2H), 3.28 – 3.19 (m, 1H), 3.15 (dd, *J* = 14.7, 4.2 Hz, 1H), 3.10 – 3.05 (m, 1H), 3.03 (dd, *J* = 15.3, 5.1 Hz, 1H), 2.98 (dd, *J* = 14.9, 9.1 Hz, 1H), 2.89 (dt, *J* = 11.2, 6.6 Hz, 1H), 2.77 – 2.69 (m, 1H), 2.55 (t, *J* = 5.5 Hz, 3H), 2.15 (ddt, *J* = 12.4, 10.1, 8.0 Hz, 1H), 2.11 – 1.99 (m, 3H), 1.84 – 1.76 (m, 2H), 1.73 – 1.64 (m, 2H), 1.63 – 1.41 (m, 7H), 1.41 – 1.30 (m, 3H), 0.86 (d, *J* = 6.6 Hz, 3H), 0.82 (d, *J* = 6.5 Hz, 3H). **^{13}C-NMR** (176 MHz, DMSO-d_6) δ [ppm]: 177.42, 172.47, 171.90, 171.54, 171.45, 170.73, 170.69, 170.07, 169.71, 169.56, 168.77, 158.29, 158.11, 157.92, 157.73, 156.67, 155.84, 136.02, 133.76, 130.09, 129.10, 127.43, 127.29, 123.69, 120.83, 118.55, 118.18,

---

[ix] 5 mL Hydrazine-DMF solution were prepared from 10 mL 1 M hydrazine solution in THF by adding 5 mL DMF and and evaopartion of the THF at 20 mmHg and 37°C over 30 min.
[x] Synthesis was designed analog to a procedure of *Verhelst et al*.[19]

117.51, 116.93, 115.82, 114.85, 111.20, 109.64, 61.74, 58.35, 55.39, 54.94, 54.50, 53.27, 51.82, 51.25, 50.51, 50.27, 50.14, 46.98, 40.80, 40.59, 36.93, 34.33, 29.76, 29.08, 28.91, 28.07, 27.73, 27.06, 24.96, 24.68, 24.46, 24.14, 23.19, 21.17, 20.41, 20.10. **LRMS** (MALDI) [m/z]: 1266.8 $[M+H]^+$, **HRMS** (ESI-IT) [m/z]: 633.8156, calculated 633.8156 for $C_{57}H_{75}N_{21}O_{13}$ $[M+ 2H]^{2+}$, err [ppm] 0.0.

Synthesis of imaging probes

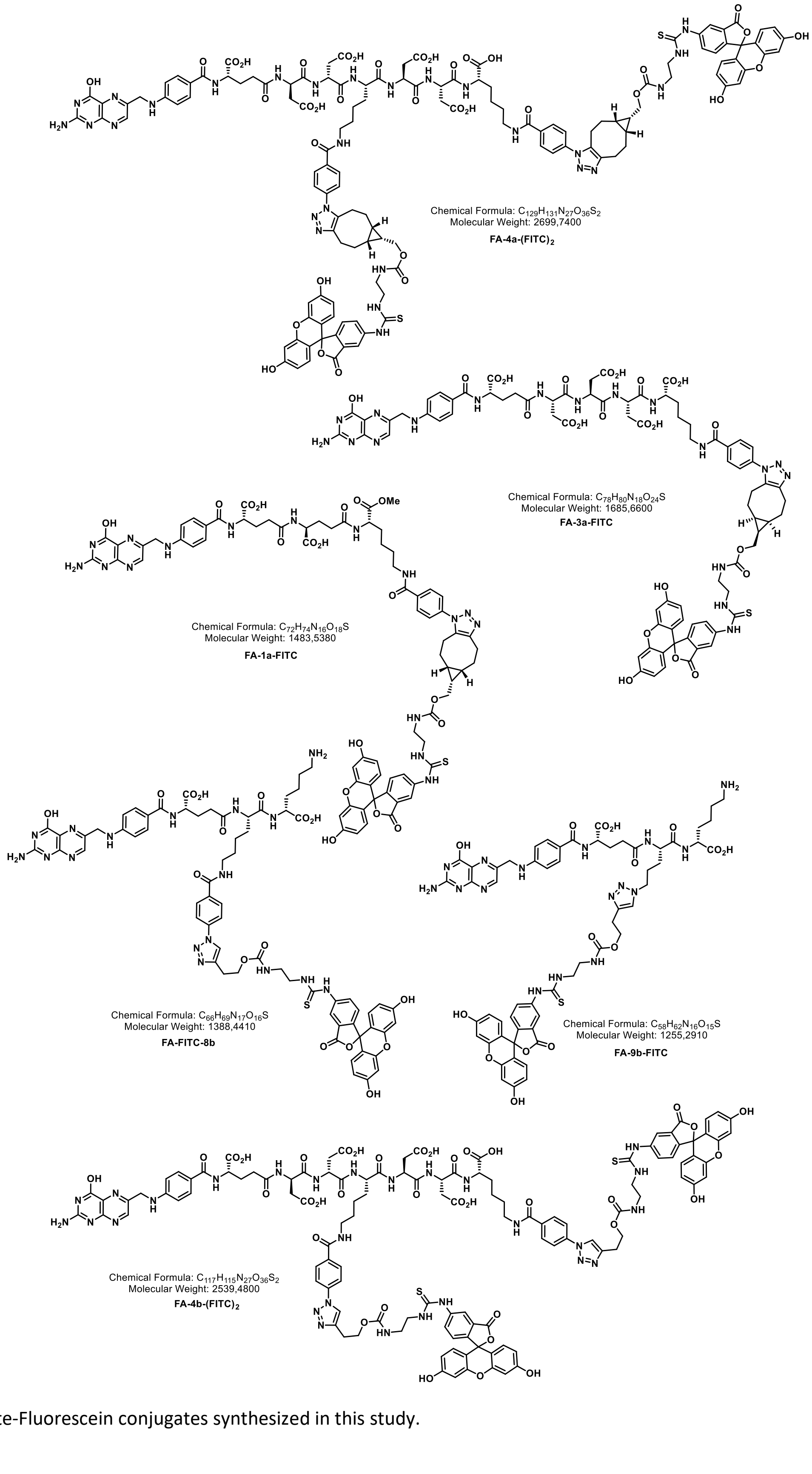

Figure S1: Folate-Fluorescein conjugates synthesized in this study.

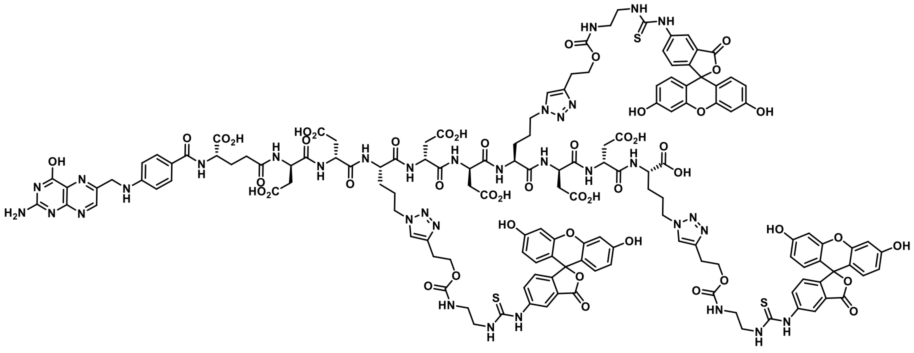

Chemical Formula: $C_{142}H_{142}N_{34}O_{48}S_3$
Molecular Weight: 3189,0680

FA-11b-(FITC)$_3$

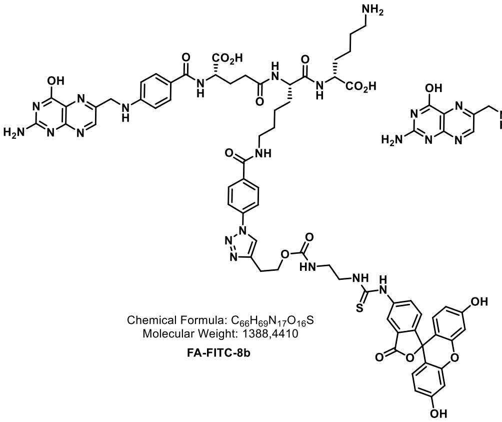

Chemical Formula: $C_{66}H_{69}N_{17}O_{16}S$
Molecular Weight: 1388,4410

FA-FITC-8b

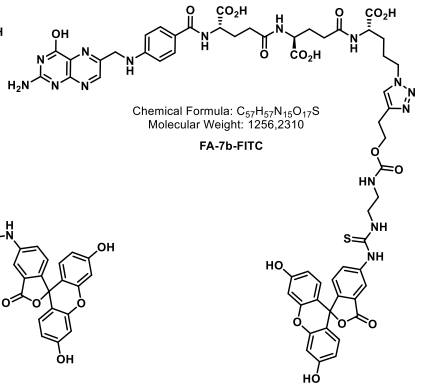

Chemical Formula: $C_{57}H_{57}N_{15}O_{17}S$
Molecular Weight: 1256,2310

FA-7b-FITC

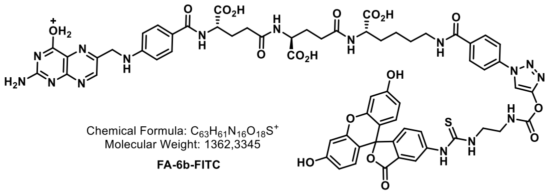

Chemical Formula: $C_{63}H_{61}N_{16}O_{18}S^+$
Molecular Weight: 1362,3345

FA-6b-FITC

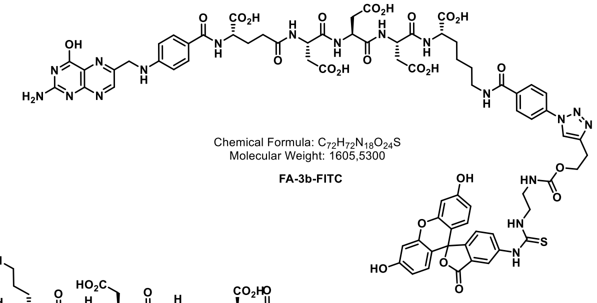

Chemical Formula: $C_{72}H_{72}N_{18}O_{24}S$
Molecular Weight: 1605,5300

FA-3b-FITC

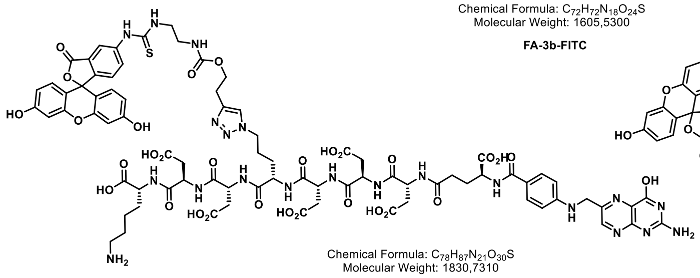

Chemical Formula: $C_{78}H_{87}N_{21}O_{30}S$
Molecular Weight: 1830,7310

FA-10b-FITC

Figure S2: Continuation 1: Folate-Fluorescein conjugates synthesized in this study.

Synthesis of clickable Fluorescence dyes

Synthesis of BCN-FTIC (43) - ((1R,8S,9s)-bicyclo[6.1.0]non-4-yn-9-yl)methyl (2-(3-(3',6'-dihydroxy-3-oxo-3H-spiro[isobenzofuran-1,9'-xanthen]-5/6-yl)thioureido)ethyl)carbamate

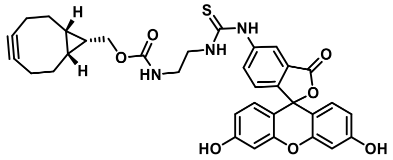

Chemical Formula: $C_{34}H_{31}N_3O_7S$
Molecular Weight: 625,70
BCN-FTIC **43**

To a solution of 50 mg (128 µmol, 1.0 equiv) 5/6-fluoresceinthioisocyanate (FTIC) in 1.3 mL dry DMF was added 20.5 mg (128 µmol, 1.0 equiv) N-Boc-1,2-ethylendiamine and the mixture was stirred for 19 h at 23°C. The reaction mixture was diluted with toluene (5 mL), concentrated and coevaporated with toluene (4x 5 mL) under reduced pressure. The residue was taken up in 130 µL TFA and stirred for 1.5 h at 23°C. The mixture was diluted with toluene (5 mL), concentrated and coevaporated with toluene (3x 4 mL) under reduced pressure and the residue was dried in HV. The residue was then dissolved in 1.3 mL dry DMF, 43 µL (384 µmol, 3.0 equiv) NMM and 40 mg (128 µmol, 1.0 equiv) **BNC-pNPC** were added and the mixture was stirred for 48 h at 23°C. The reaction mixture was diluted with toluene (5 mL), concentrated and coevaporated with toluene (5x 5 mL) under reduced pressure. The residue was purified by preparative thin-layer chromatography (CH_2Cl_2:MeOH/9:1) yielding 36.6 mg (58.49 µmol, 46%) of BCN-FTIC **43** as a deep orange, amorph solid.

BCN-FTIC (43): TLC (CH_2Cl_2:MeOH/9:1) R_f: 0.35 [UV^{254}, Vis],**^{1}H-NMR** (500 MHz, MeOD-*d*$_4$) δ [ppm]: 8.07 (s, 1H), 7.75 (d, *J* = 7.8 Hz, 1H), 7.17 (d, *J* = 8.2 Hz, 1H), 6.74 (d, *J* = 8.7 Hz, 2H), 6.68 (d, *J* = 2.4 Hz, 2H), 6.55 (dd, *J* = 8.7, 2.4 Hz, 2H), 4.10 (d, *J* = 8.1 Hz, 2H), 3.80 – 3.66 (m, 2H), 3.37 (t, *J* = 5.8 Hz, 1H), 3.35 (s, 1H), 2.17 (dq, *J* = 32.4, 14.9, 14.3 Hz, 6H), 1.53 (q, *J* = 8.5 Hz, 2H), 1.34 (dt, *J* = 14.5, 7.3 Hz, 1H), 0.93 – 0.82 (m, 2H). **^{13}C-NMR** (126 MHz, MeOD-*d*$_4$) δ [ppm]: 183.13, 171.18, 162.56, 159.80, 154.60, 141.86, 131.82, 130.55, 126.33, 120.97, 114.26, 111.85, 103.55, 99.50, 92.45, 66.78, 63.97, 55.78, 40.93, 30.15, 21.92, 21.40, 18.91. **LRMS** (ESI-Quad) [m/z]: 626.2 $[M+H]^+$, **HRMS** (ESI-IT) [m/z]: 626.194754, calculated 626.195548 for $C_{34}H_{32}N_3O_7S$ $[M+H]^+$, err [ppm] 1.268.

Synthesis of Homopropargyl-FTIC (42) - But-3-yn-1-yl (2-(3-(3',6'-dihydroxy-3-oxo-3H-spiro[isobenzofuran-1,9'-xanthen]-5/6-yl)thioureido)ethyl)carbamate

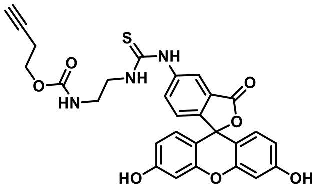

Chemical Formula: $C_{28}H_{23}N_3O_7S$
Molecular Weight: 545,57
Homopropargyl-FTIC **48**

To a solution of 50 mg (128 µmol, 1.0 equiv) 5/6-fluoresceinthioisocyanate (FTIC) in 1.3 mL dry DMF was added 20.5 mg (128 µmol, 1.0 equiv) N-Boc-1,2-ethylendiamine and the mixture was stirred for 19 h at 23°C. The reaction mixture was diluted with toluene (5 mL), concentrated and coevaporated with toluene (4x 5 mL) under reduced pressure. The residue was taken up in 130 µL TFA and stirred for 1.5 h at 23°C. The mixture was diluted with toluene (5 mL), concentrated and coevaporated with toluene (3x 4 mL) under reduced pressure and the residue was dried in HV. The residue was then dissolved in 1.3 mL dry DMF, 43 µL (384 µmol, 3.0 equiv) NMM and 30 mg (128 µmol, 1.0 equiv) **24** were added and the mixture was stirred for 48 h at 23°C. The reaction mixture was diluted with toluene (5 mL), concentrated and coevaporated with toluene (5x 5 mL) under reduced pressure. The residue was purified by preparative thin-layer chromatography (CH_2Cl_2:MeOH/9:1) followed by purification by RP prep HPLC (Phenomenex Gemini C18 RP-column 00G-4435-PO-AX, 5 µm, 110 A, 250×21.20 mm, Flow: 9 mL/min, ACN:H_2O/ 20:90 → 95:5 in 45 min) yielding after lyophilization 43.6 mg (80.0 µmol, 63%) of Homopropargyl-FTIC **42** as a deep orange, amorph solid.

Homopropargyl-FTIC (42): TLC (CH_2Cl_2:MeOH/9:1) R_f: 0.20 [UV^{254}, Vis],**^{1}H-NMR** (500 MHz, DMSO-d_6) δ [ppm]: 8.13 (s, 1H), 7.80 – 7.66 (m, 1H), 7.17 (d, *J* = 8.2 Hz, 1H), 6.73 – 6.63 (m, 4H), 6.55 (ddd, *J* = 8.7, 3.9, 2.4 Hz, 2H), 4.15 – 4.05 (m, 2H), 3.76 (d, *J* = 20.0 Hz, 2H), 3.38 (t, *J* = 6.0 Hz, 2H), 2.81 (s, 10H), 2.48 (td, *J* = 6.8, 2.6 Hz, 1H). **LRMS** (ESI-Quad) [m/z]: 546.1 $[M+H]^+$, **HRMS** (ESI-IT) [m/z]: 546.132346, calculated 546.132948 for $C_{28}H_{24}N_3O_7S$ $[M+H]^+$, err [ppm] 1.102.

Synthesis of Folate-Fluorescein Conjugates

General procedure C for the synthesis of Folate-Fluorescein Conjugates via copper-free click reaction.

The corresponding **FA-N₃** (1.1 equiv) was dissolved in DMSO (0.2 M), a solution of BCN-FTIC **43** (1.0 equiv) in DMSO (0.2 M) was added and the mixture was stirred for 4-20 h at 23°C under light exclusion until complete conversion was monitored by LCMS. The reaction mixture was diluted with 100 µL of MeOH, filtered through a Whatman filter (45 µm) and directly purified by RP prep HPLC (Phenomenex Gemini C18 RP-column 00G-4435-PO-AX, 5 µm, 110 A, 250×21.20 mm, Flow: 9 mL/min, ACN:H_2O + 0.1% TFA/ 10:90 → 95:5 in 45 min) yielding the Folate-Fluorescein Conjugates after lyophilization as a deep orange, amorph solids.

FA-4a-(FITC)₂

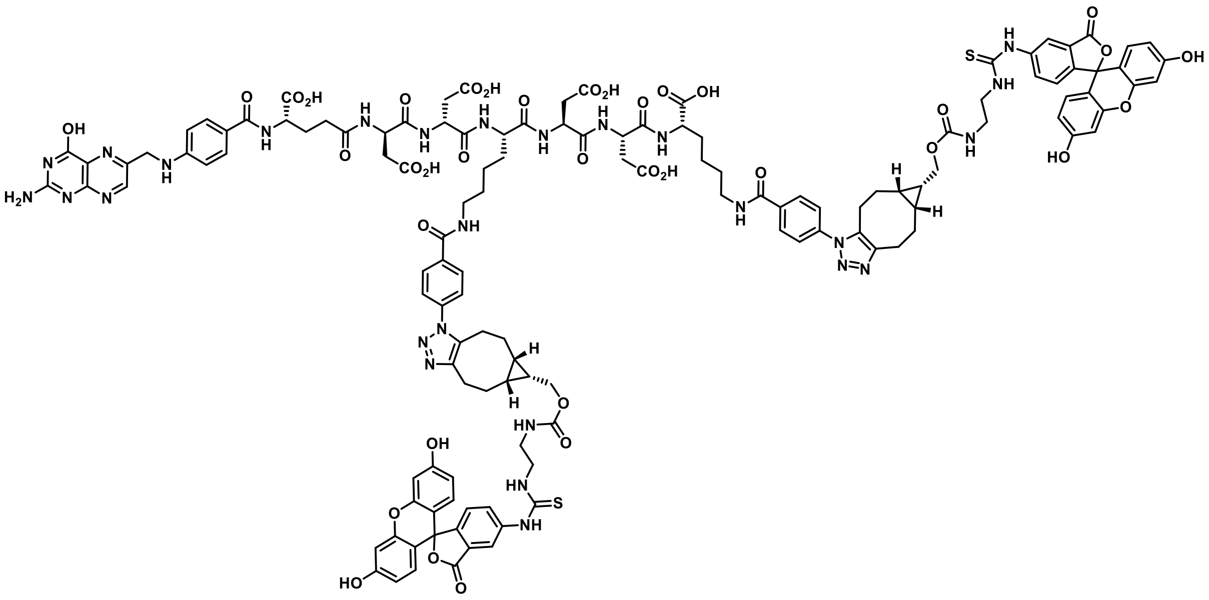

Chemical Formula: $C_{129}H_{131}N_{27}O_{36}S_2$
Molecular Weight: 2699,74
FA-4a-(FITC)₂

Applying **FA-N₃-4** to the general procedure C, 1.4 mg (0.518 µmol, 17%) **FA-4a-(FITC)₂** were obtained as a mixture of diastereomers as deep-orange solid.

FA-4a-(FITC)₂: LRMS (ESI-Quad) [m/z]: 1350.4 $[M+2H]^{2+}$, **HRMS** (ESI-IT) [m/z]: 898.61675, calculated 898.61689 for $C_{129}H_{128}N_{27}O_{36}S_2$ $[M-3H]^{3-}$, err [ppm] -0.155.

FA-3a-FITC

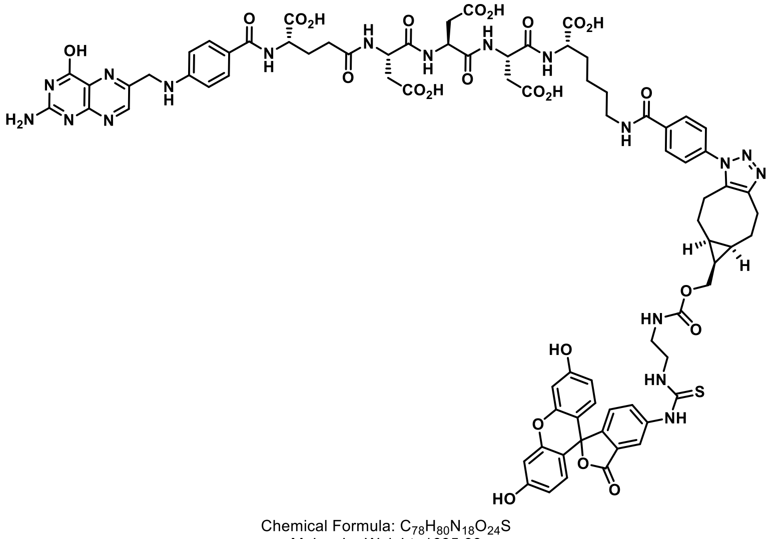

Chemical Formula: $C_{78}H_{80}N_{18}O_{24}S$
Molecular Weight: 1685,66
FA-3a-FITC

Applying **FA-N₃-3** to the general procedure C, 4.5 mg (2.669 µmol, 30%) **FA-3a-FITC** were obtained as a mixture of diastereomers as a deep-orange solid.

FA-3a-FITC: LRMS (ESI-Quad) [m/z]: 1685.5 $[M+H]^+$, **HRMS** (ESI-IT) [m/z]: 841.25711, calculated 841.25840 for $C_{78}H_{78}N_{18}O_{24}S$ $[M-2H]^{2-}$, err [ppm] -1.53.

FA-1a-FITC

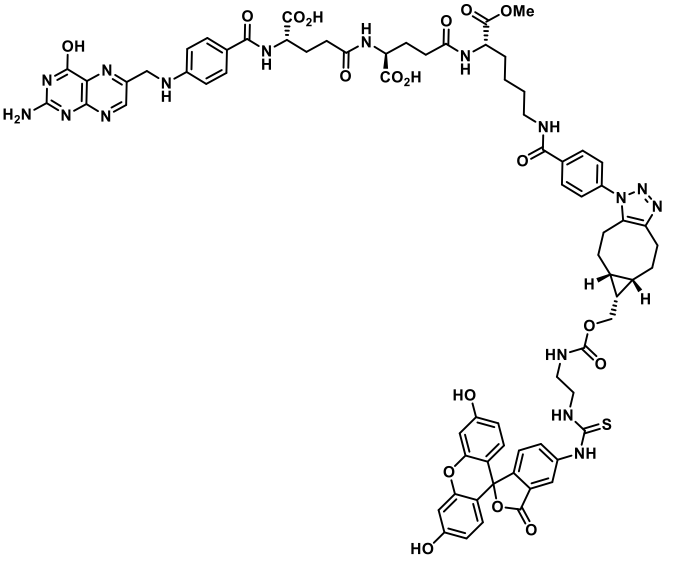

Chemical Formula: $C_{72}H_{74}N_{16}O_{18}S$
Molecular Weight: 1483,54
FA-1a-FITC

Applying **FA-N$_3$-1** to the general procedure C, 1.7 mg (1.145 µmol, 25%) **FA-1a-FITC** were obtained as a mixture of diastereomers as a deep-orange solid.

FA-1a-FITC: LRMS (ESI-Quad) [m/z]: 1484.6 $[M+H]^+$, **HRMS** (ESI-IT) [m/z]:742.2617, calculated 742.2617 for $C_{72}H_{76}N_{16}O_{18}S$ $[M+2H]^{2+}$, err [ppm] 0.0.

General procedure D for the synthesis of Folate-Fluorescein Conjugates via copper-mediated click reaction.

The corresponding **FA-N$_3$** (1.1 equiv) and Homopropargyl-FTIC **42** (1.0 equiv) were dissolved in a mixture of DMSO:H_2O:tBuOH/2:1:1 (0.025 M) were added DiPEA (6.0 eq), TBTA (0.1 eq, 10 µL from a stock solution in DMSO), $CuSO_4$ (0.05 eq, 10 µL from a stock solution in H_2O) and sodium ascorbate (0.5 eq, 10 µL from a stock solution in H_2O) and the mixture was stirred under light exclusion for 4-24 h at 23°C until complete conversion was monitored by LCMS. The reaction mixture was diluted with 100 µL of MeOH, filtered through a Whatman filter (45 µm) and directly purified by RP prep HPLC (Phenomenex Gemini C18 RP-column 00G-4435-PO-AX, 5 µm, 110 A, 250×21.20 mm, Flow: 9 mL/min, ACN:H_2O + 0.1% TFA/ 10:90 → 95:5 in 45 min) yielding the Folate-Fluorescein Conjugates after lyophilization as a deep orange, amorph solids.

FA-9b-FITC

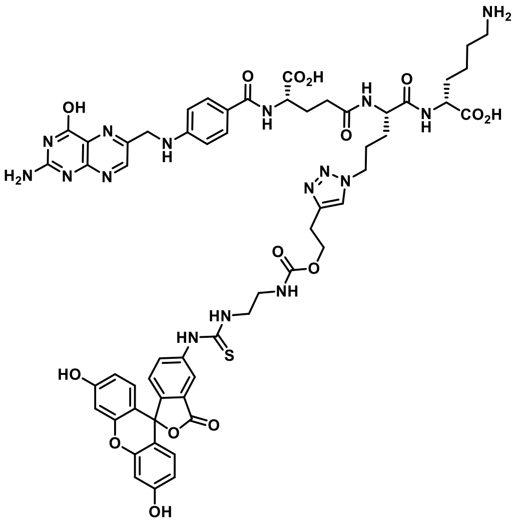

Chemical Formula: $C_{58}H_{62}N_{16}O_{15}S$
Molecular Weight: 1255,29

FA-9b-FITC

Applying **FA-N$_3$-9** to the general procedure D, 2.9 mg (2.36 µmol, 65%) **FA-FITC-9b** were obtained as a TFA salt as a deep-orange solid.

FA-9b-FITC: LRMS (ESI-Quad) [m/z]: 1256.3 $[M+H]^+$, **HRMS** (ESI-IT) [m/z]: 628.22296, calculated 628.22234 for $C_{58}H_{64}N_{16}O_{15}S$ $[M+2H]^{2+}$, err [ppm] 0.986.

FA-11b-(FITC)₃

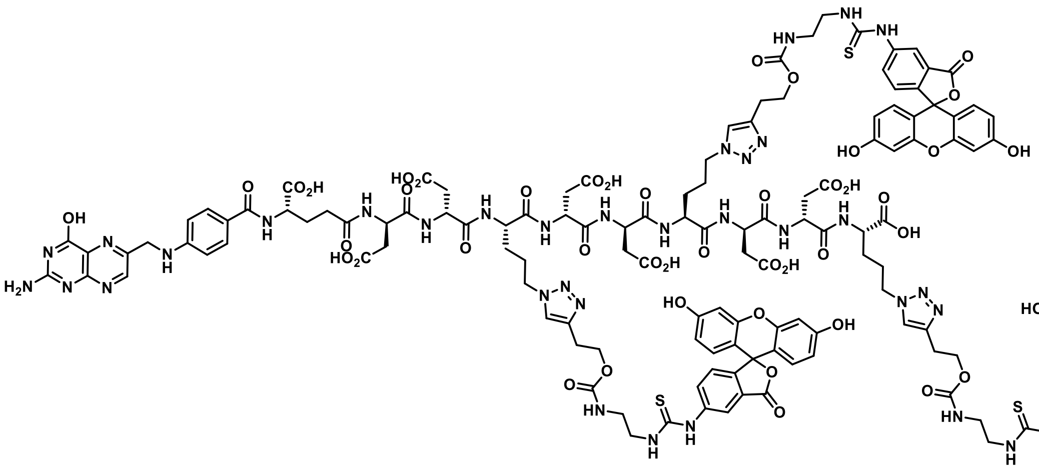

Chemical Formula: $C_{142}H_{142}N_{34}O_{48}S_3$
Molecular Weight: 3189,07

FA-11b-(FITC)₃

Applying **FA-N₃-11** to the general procedure D, 3.0 mg (0.94 µmol, 25%) **FA-11b-(FITC)₃** were obtained as a deep-orange solid.

FA-11b-(FITC)₃: LRMS (ESI-Quad) [m/z]: 1064.32 $[M+3H]^{3+}$, **HRMS** (ESI-IT) [m/z]: 1063.9719, calculated 1063.9715 for $C_{142}H_{145}N_{34}O_{48}S_3$ $[M+3H]^{3+}$, err [ppm] 0.376.

FA-8b-FITC

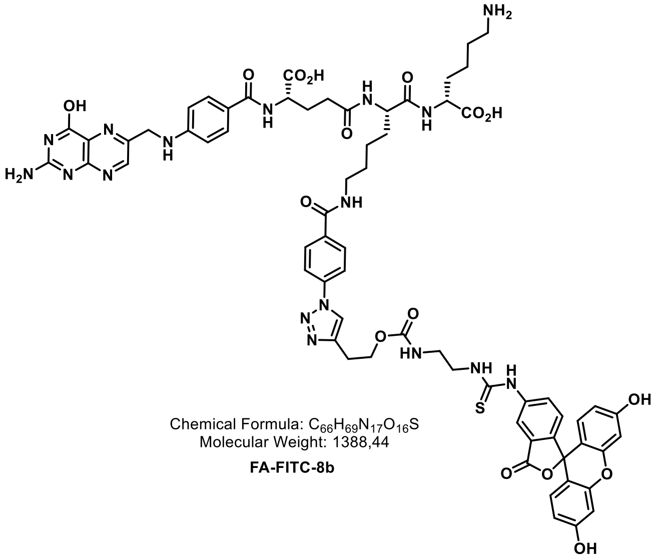

Chemical Formula: $C_{66}H_{69}N_{17}O_{16}S$
Molecular Weight: 1388,44

FA-FITC-8b

Applying **FA-N$_3$-8** to the general procedure D, 1.7 mg (1.25 µmol, 34%) **FA-8b-FITC** were obtained as a TFA salt as a deep-orange solid.

FA-8b-FITC: LRMS (ESI-Quad) [m/z]: 694.6 $[M+2H]^{2+}$, **HRMS** (ESI-IT) [m/z]: 694.7487, calculated 694.7487 for $C_{66}H_{71}N_{17}O_{16}S$ $[M+2H]^{2+}$, err [ppm] 0.0.

FA-10b-FITC

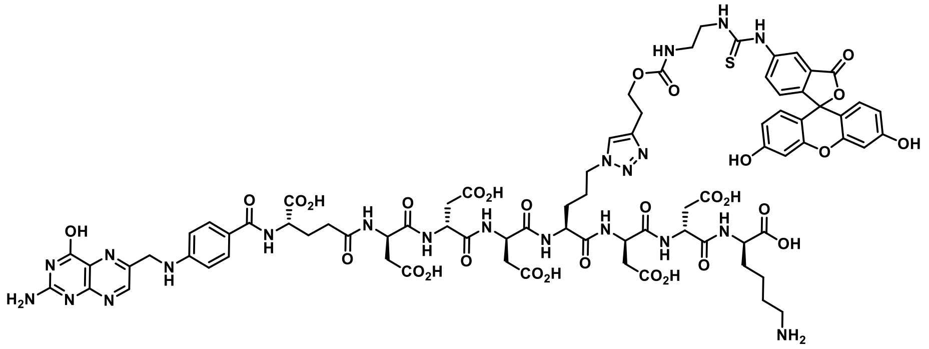

Chemical Formula: $C_{78}H_{87}N_{21}O_{30}S$
Molecular Weight: 1830,73

FA-10b-FITC

Applying **FA-N$_3$-10** to the general procedure D, 3.8 mg (2.08 µmol, 57%) **FA-10b-FITC** were obtained as a deep-orange solid.

FA-10b-FITC: LRMS (ESI-Quad) [m/z]: 915.9 $[M+2H]^{2+}$, **HRMS** (ESI-IT) [m/z]: 915.7897, calculated 915.7897 for $C_{78}H_{89}N_{21}O_{30}S$ $[M+2H]^{2+}$, err [ppm] 0.0.

FA-7b-FITC

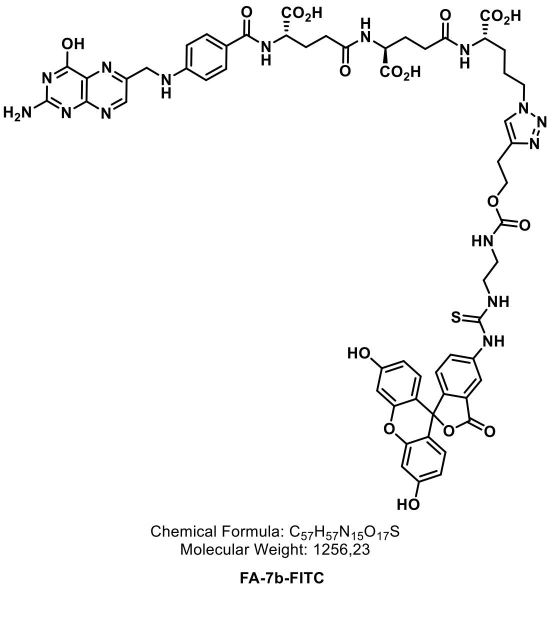

Chemical Formula: $C_{57}H_{57}N_{15}O_{17}S$
Molecular Weight: 1256,23

FA-7b-FITC

Applying **FA-N$_3$-7** to the general procedure D, 3.8 mg (2.08 µmol, 57%) **FA-FITC-7b** were obtained as a deep-orange solid.

FA-7b-FITC: LRMS (ESI-Quad) [m/z]: 915.8 $[M+2H]^{2+}$, **HRMS** (ESI-IT) [m/z]: 1256.3869, calculated 1256.3850 for $C_{57}H_{58}N_{15}O_{17}S$ $[M+H]^+$, err [ppm] -1.5.

FA-6b-FITC

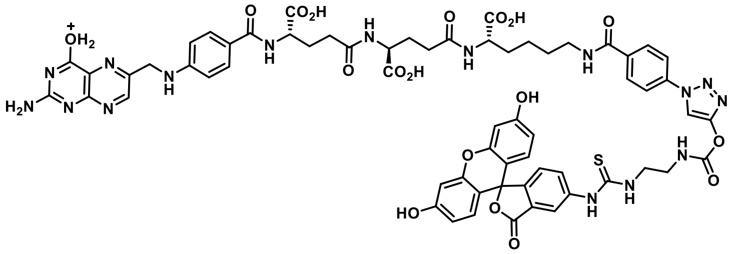

Chemical Formula: $C_{63}H_{61}N_{16}O_{18}S^+$
Molecular Weight: 1362,33
FA-6b-FITC

Applying **FA-N$_3$-6** to the general procedure D, 3.8 mg (2.08 µmol, 57%) **FA-FITC-6b** were obtained as a deep-orange solid.

FA-6b-FITC: LRMS (ESI-Quad) [m/z]: 681.3 $[M+2H]^{2+}$. **HRMS** (ESI-IT) [m/z]: 1361.4061, calculated 1361.4065 for $C_{63}H_{61}N_{16}O_{18}S$ $[M+H]^+$, err [ppm] -0.29.

FA-3b-FITC

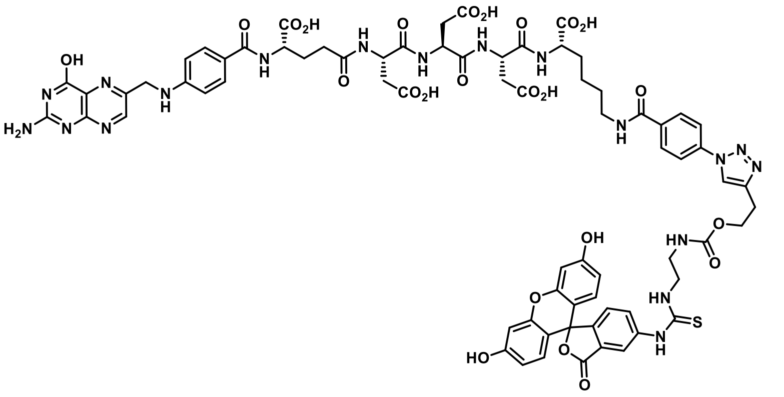

Chemical Formula: $C_{72}H_{72}N_{18}O_{24}S$
Molecular Weight: 1605,53
FA-3b-FITC

Applying **FA-N$_3$-3** to the general procedure D, 2.0 mg (1.24 µmol, 52%) **FA-3b-FITC** were obtained as a deep-orange solid.

FA-3b-FITC: LRMS (ESI-Quad) [m/z]: 803.2 $[M+2H]^{2+}$, **HRMS** (ESI-IT) [m/z]: 803.2417, calculated 803.2417 for $C_{72}H_{74}N_{18}O_{24}S$ $[M+2H]^{2+}$, err [ppm] 0.0.

FA-4b-(FITC)$_2$

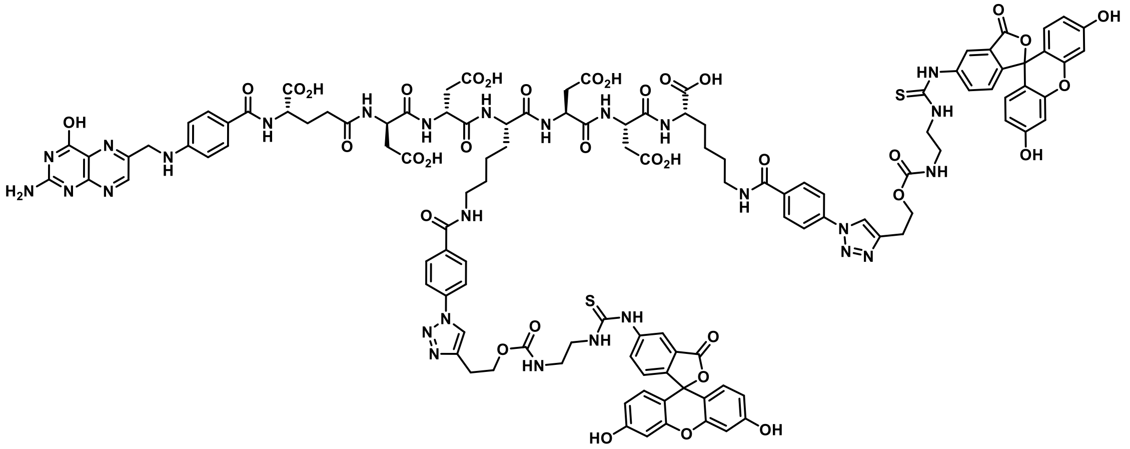

Chemical Formula: $C_{117}H_{115}N_{27}O_{36}S_2$
Molecular Weight: 2539,48
FA-4b-(FITC)$_2$

Applying **FA-N$_3$-4** to the general procedure D, 1.4 mg (0.551 µmol, 27%) **FA-4b-(FITC)$_2$** were obtained as a deep-orange solid.

FA-4b-(FITC)$_2$: LRMS (ESI-Quad) [m/z]: 1270.3 $[M+2H]^{2+}$, **HRMS** (ESI-IT) [m/z]: 1269.8793, calculated 1269.8792 for $C_{117}H_{117}N_{27}O_{36}S_2$ $[M+H]^{+}$, err [ppm] -0.078.

Conjugation of Ratjadone derivatives to carrier molecules

Biotin-Ratjadone Conjugate

Synthesis of Biotin-PEG$_3$-16*S*-Aminoratjadone (36)

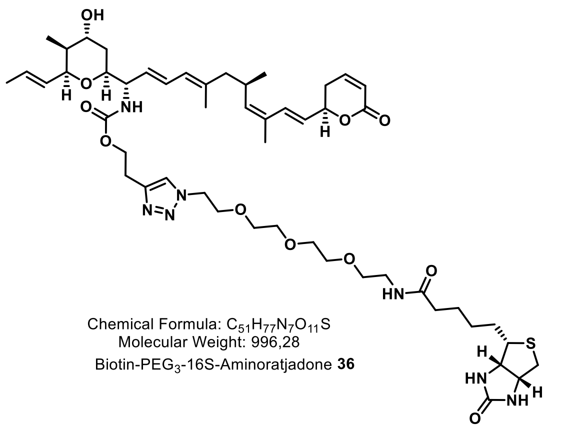

Chemical Formula: $C_{51}H_{77}N_7O_{11}S$
Molecular Weight: 996,28
Biotin-PEG$_3$-16S-Aminoratjadone **36**

To a solution of 3.6 mg (6.53 µmol, 1.0 equiv) of 16*S*-aminoratjadone derivative **22** and 3.2 mg (7.18 µmol, 1.1 equiv) N-(2-(2-(2-(2-azidoethoxy)ethoxy)ethoxy)ethyl)-5-((3aS,4S,6aR)-2-oxohexahydro-1H-thieno[3,4-d]imidazol-4-yl)pentanamide in a mixture of DMSO:H_2O:tBuOH/2:1:1 were added 9.5 µL (19.6mol, 3.0 equiv) DiPEA, 10 µL (0.345 mg, 0.1 equiv) of a stock solution (34.5 mg/1 mL) of TBTA in DMSO, 10 µL (0.052 mg, 0.05 equiv) of a stock solution (5.2 mg/1 mL) of $CuSO_4$ in H_2O and 10 µL (0.646 mg, 0.1 equiv) of a stock solution (64.6 mg/1 mL) of sodium ascorbate in H_2O. The mixture was stirred for 24 h at 23°C, before it was diluted with 100 µL of and 200 µL od DMSO and was purified by RP prep HPLC (Phenomenex Gemini C18 RP-column 00G-4435-PO-AX, 5 µm, 110 A, 250×21.20 mm, Flow: 9 mL/min, ACN:H_2O + 0.1% TFA/ 10:90 → 95:5 in 60 min) yielding after lyophilization 2.5 mg (2.51 µmol, 39%) of Biotin-PEG$_3$-16*S*-aminoratjadone **36** as a pale-yellow, amorph solid.

Biotin-PEG$_3$-16*S*-Aminoratjadone (36): **^{1}H-NMR** (700 MHz, DMSO-*d*$_6$) δ [ppm]: 7.90 (s, 1H), 7.81 (t, *J* = 5.6 Hz, 1H), 7.13 (d, *J* = 8.9 Hz, 1H), 7.04 (ddd, *J* = 9.7, 5.6, 2.8 Hz, 1H), 6.75 (d, *J* = 15.6 Hz, 1H), 6.43 – 6.28 (m, 3H), 6.01 – 5.93 (m, 1H), 5.79 – 5.72 (m, 2H), 5.61 – 5.51 (m, 1H), 5.48 (dd, *J* = 15.2, 6.8 Hz, 1H), 5.36 (ddd, *J* = 15.4, 5.4, 1.6 Hz, 1H), 5.24 (d, *J* = 9.5 Hz, 1H), 5.10 (dt, *J* = 10.5, 5.1 Hz, 1H), 4.46 (t, *J* = 5.3 Hz, 2H), 4.30 (dd, *J* = 7.5, 5.1 Hz, 1H), 4.26 – 4.22 (m, 1H), 4.17 (t, *J* = 6.9 Hz, 2H), 4.12 (dd, *J* = 7.6, 4.5 Hz, 1H), 4.07 – 3.99 (m, 1H), 3.79 (t, *J* = 5.3 Hz, 2H), 3.73 – 3.69 (m, 1H), 3.65 (dd, *J* = 10.5, 5.0 Hz, 1H), 3.51 (dd, *J* = 5.9, 3.3 Hz, 4H), 3.48 (d, *J* = 2.8 Hz, 4H), 3.47 (s, 3H), 3.38 (t, *J* = 6.0 Hz, 2H), 3.17 (q, *J* = 5.9 Hz, 2H), 3.11 – 3.06 (m, 1H), 2.91 (t, *J* = 7.0 Hz, 2H), 2.88 – 2.83 (m, 1H), 2.81 (dd, *J* = 12.4, 5.1 Hz, 1H), 2.57 (d, *J* = 12.4 Hz, 1H), 2.56 – 2.51 (m, 1H), 2.45 (ddt, *J* = 18.6, 10.6, 2.7 Hz, 1H), 2.05 (t, *J* = 7.4 Hz, 2H), 1.98 (d, *J* = 7.1 Hz, 2H),

1.75 (s, 3H), 1.68 (s, 3H), 1.63 (d, *J* = 6.5 Hz, 3H), 1.62 – 1.56 (m, 1H), 1.53 – 1.38 (m, 5H), 1.34 – 1.21 (m, 4H), 0.87 (d, *J* = 6.6 Hz, 3H), 0.74 (d, *J* = 7.1 Hz, 3H). **^{13}C-NMR** (176 MHz, DMSO-d_6) δ [ppm]: 172.08, 163.52, 162.67, 155.75, 146.69, 143.07, 138.49, 136.28, 131.20, 129.35, 129.04, 127.25, 126.78, 125.80, 124.59, 122.92, 120.33, 107.51, 77.81, 73.62, 73.52, 69.68, 69.61, 69.56, 69.53, 69.15, 68.73, 68.18, 62.78, 61.02, 59.17, 56.48, 55.40, 49.27, 47.25, 40.02, 38.65, 38.42, 35.08, 29.58, 29.40, 29.21, 28.18, 28.03, 25.49, 25.24, 20.78, 20.07, 17.62, 16.44, 11.09. **LRMS** (ESI-Quad) [m/z]: 996.5 $[M+H]^+$, **HRMS** (ESI-IT) [m/z]: 996.546028, calculated 996.547454 for $C_{51}H_{78}N_7O_{11}S$ $[M+H]^+$, err [ppm] -1.431

Folate-Aminoratjadone Conjugates

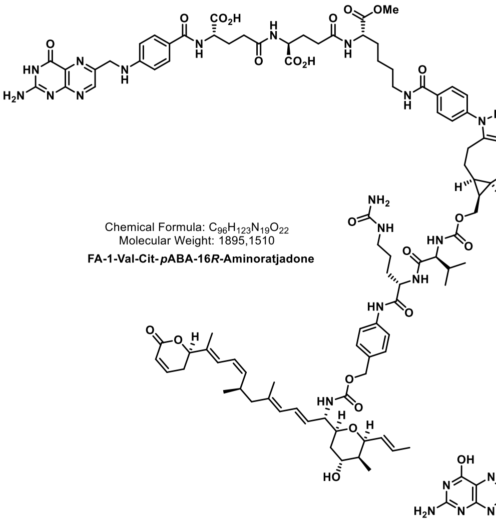

Chemical Formula: $C_{96}H_{123}N_{19}O_{22}$
Molecular Weight: 1895,1510
FA-1-Val-Cit-*p*ABA-16*R*-Aminoratjadone

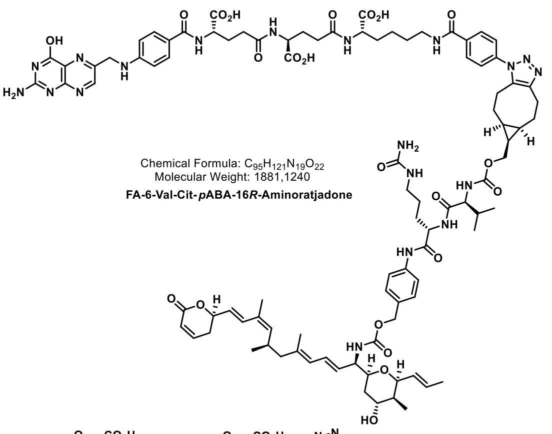

Chemical Formula: $C_{95}H_{121}N_{19}O_{22}$
Molecular Weight: 1881,1240
FA-6-Val-Cit-*p*ABA-16*R*-Aminoratjadone

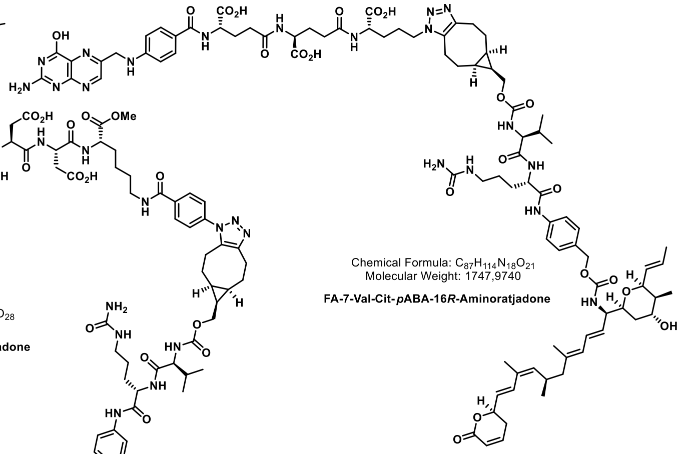

Chemical Formula: $C_{87}H_{114}N_{18}O_{21}$
Molecular Weight: 1747,9740
FA-7-Val-Cit-*p*ABA-16*R*-Aminoratjadone

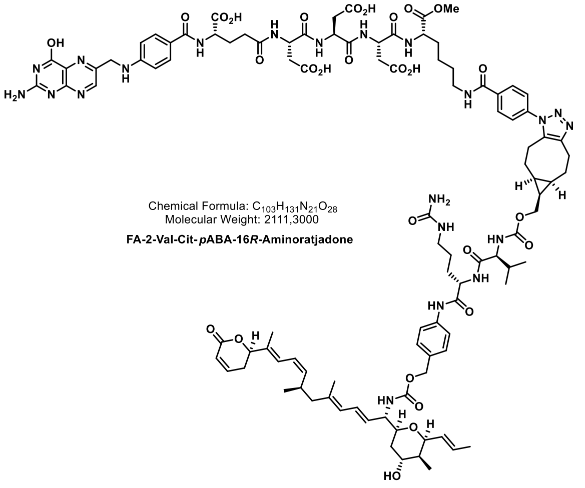

Chemical Formula: $C_{103}H_{131}N_{21}O_{28}$
Molecular Weight: 2111,3000
FA-2-Val-Cit-*p*ABA-16*R*-Aminoratjadone

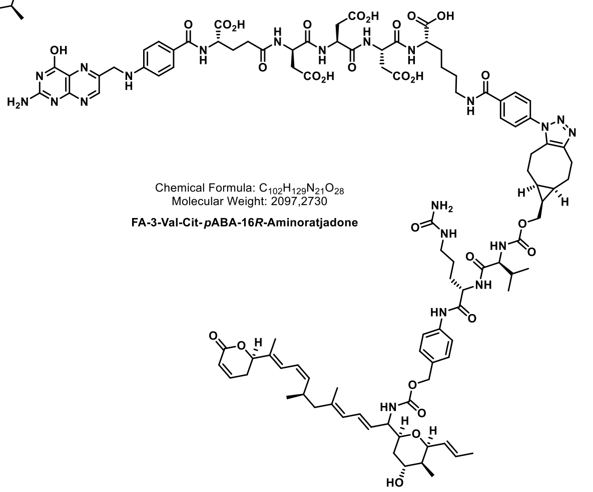

Chemical Formula: $C_{102}H_{129}N_{21}O_{28}$
Molecular Weight: 2097,2730
FA-3-Val-Cit-*p*ABA-16*R*-Aminoratjadone

Figure S3: Folate-Aminoratjadone conjugates synthesized in this study.

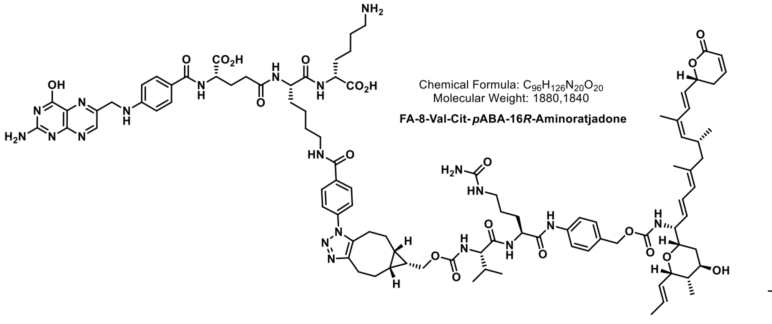

Chemical Formula: $C_{96}H_{126}N_{20}O_{20}$
Molecular Weight: 1880,1840
FA-8-Val-Cit-*p*ABA-16*R*-Aminoratjadone

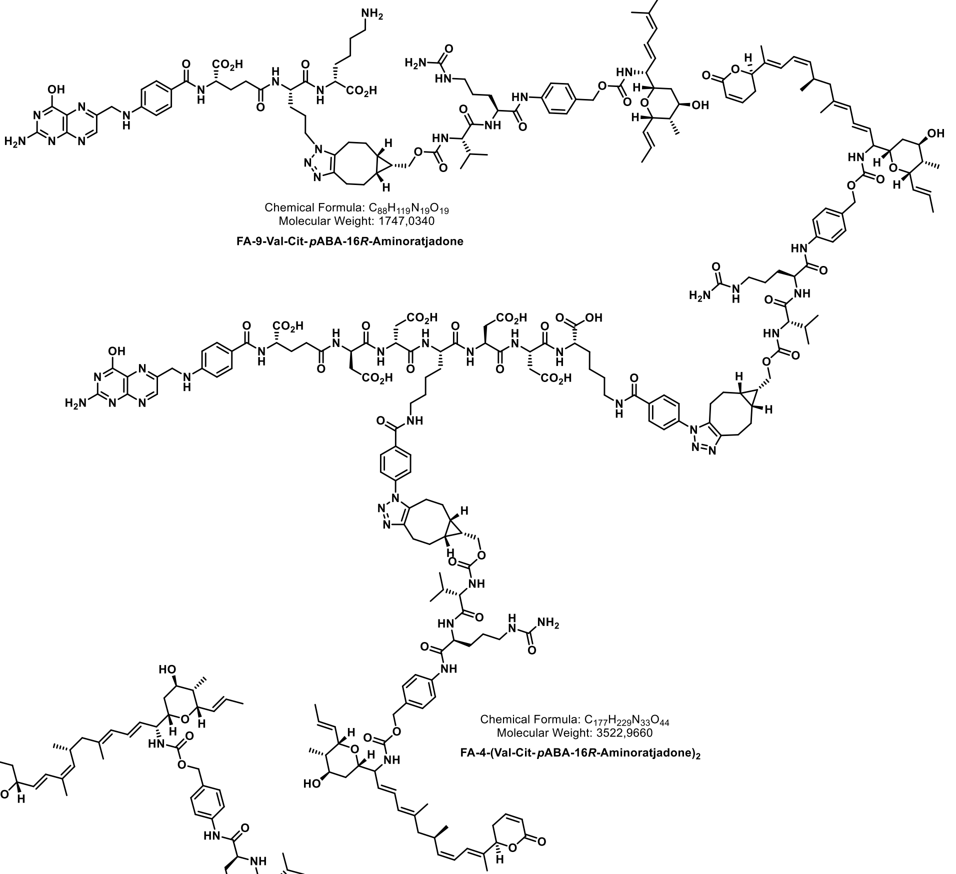

Chemical Formula: $C_{88}H_{119}N_{19}O_{19}$
Molecular Weight: 1747,0340
FA-9-Val-Cit-*p*ABA-16*R*-Aminoratjadone

Chemical Formula: $C_{177}H_{229}N_{33}O_{44}$
Molecular Weight: 3522,9660
FA-4-(Val-Cit-*p*ABA-16*R*-Aminoratjadone)$_2$

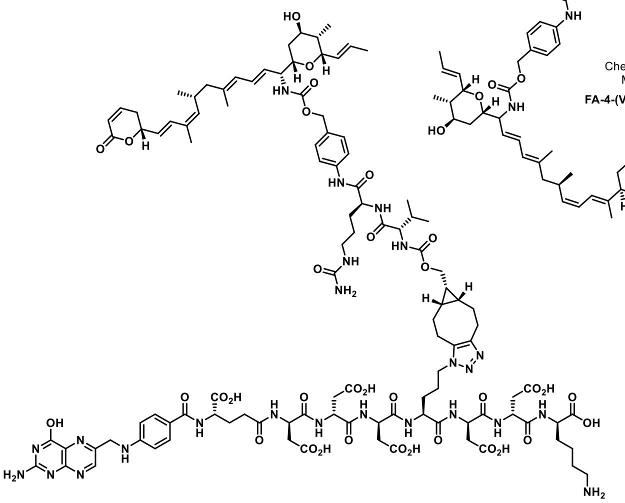

Chemical Formula: $C_{108}H_{144}N_{24}O_{34}$
Molecular Weight: 2322,4740
FA-10-Val-Cit-*p*ABA-16*R*-Aminoratjadone

Figure S4: Continuation 1: Folate-Aminoratjadone conjugates synthesized in this study.

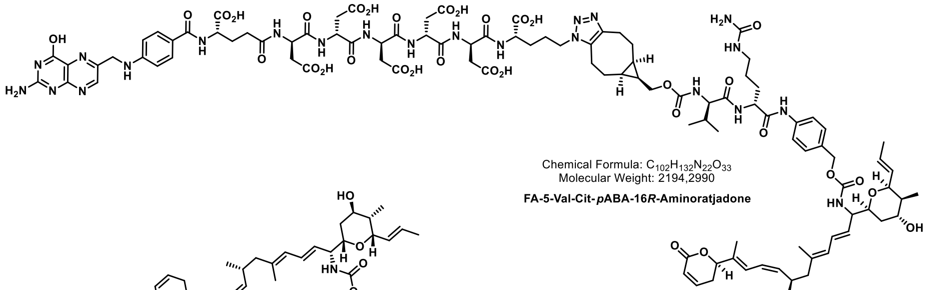

Chemical Formula: $C_{102}H_{132}N_{22}O_{33}$
Molecular Weight: 2194,2990
FA-5-Val-Cit-*p*ABA-16*R*-Aminoratjadone

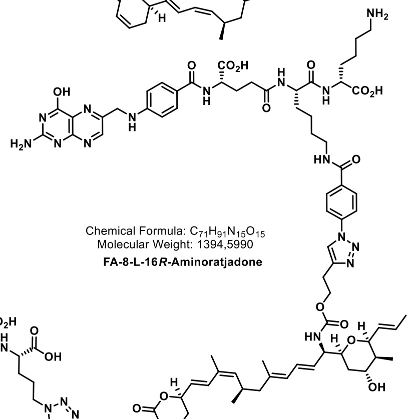

Chemical Formula: $C_{71}H_{91}N_{15}O_{15}$
Molecular Weight: 1394,5990
FA-8-L-16*R*-Aminoratjadone

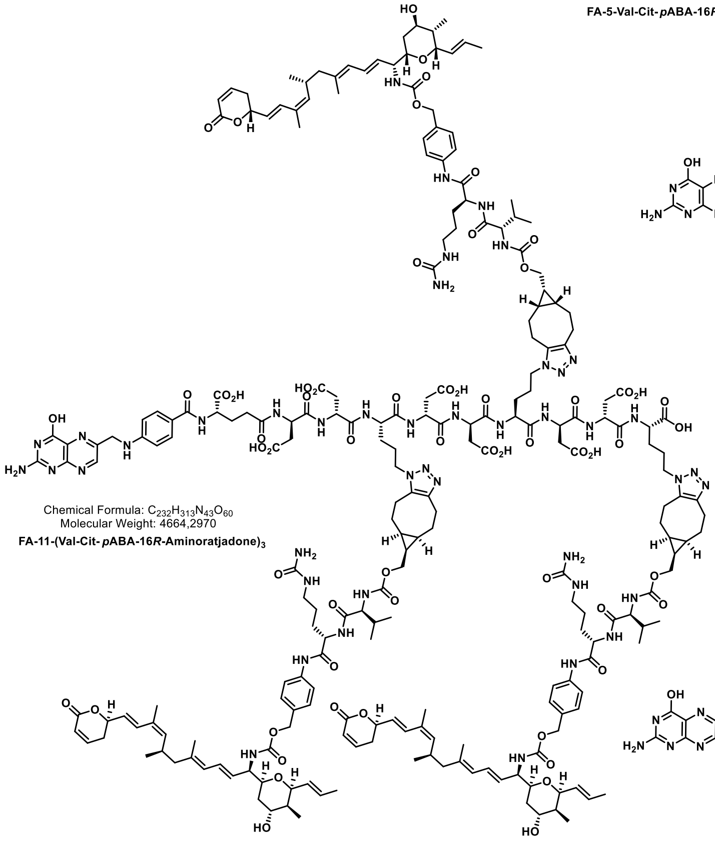

Chemical Formula: $C_{232}H_{313}N_{43}O_{60}$
Molecular Weight: 4664,2970
FA-11-(Val-Cit-*p*ABA-16*R*-Aminoratjadone)$_3$

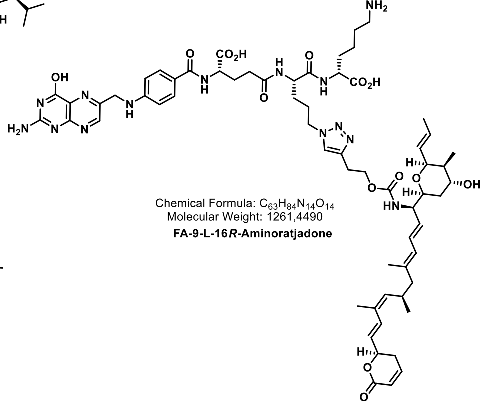

Chemical Formula: $C_{63}H_{84}N_{14}O_{14}$
Molecular Weight: 1261,4490
FA-9-L-16*R*-Aminoratjadone

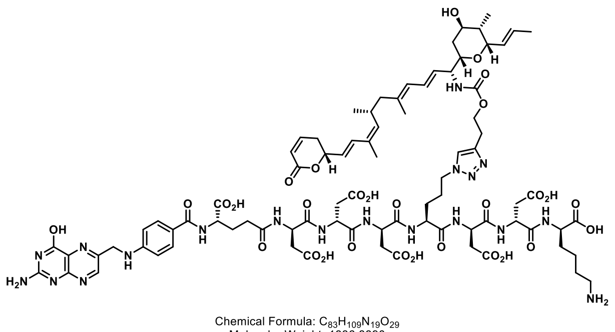

Chemical Formula: $C_{83}H_{109}N_{19}O_{29}$
Molecular Weight: 1836,8890
FA-10-L-16*R*-Aminoratjadone

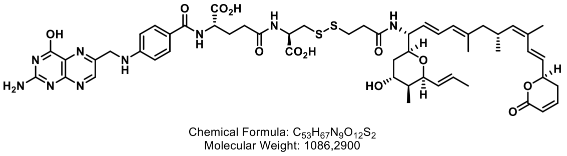

Chemical Formula: $C_{53}H_{67}N_9O_{12}S_2$
Molecular Weight: 1086,2900
FA-SS-16*R*-Aminoratjadone

Figure S5: Continuation 2: Folate-Aminoratjadone conjugates synthesized in this study.

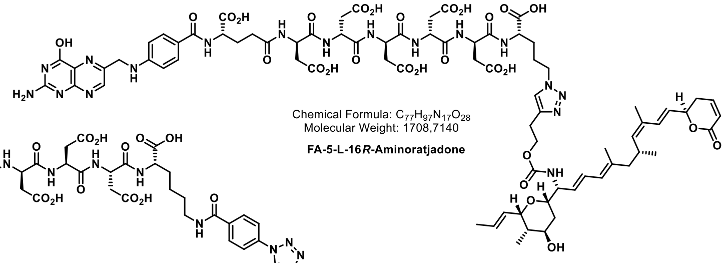

Chemical Formula: $C_{77}H_{97}N_{17}O_{28}$
Molecular Weight: 1708,7140
FA-5-L-16*R*-Aminoratjadone

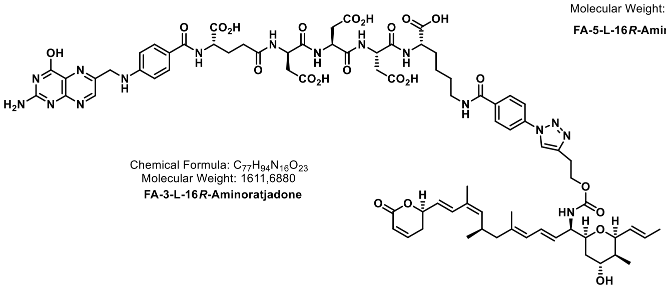

Chemical Formula: $C_{77}H_{94}N_{16}O_{23}$
Molecular Weight: 1611,6880
FA-3-L-16*R*-Aminoratjadone

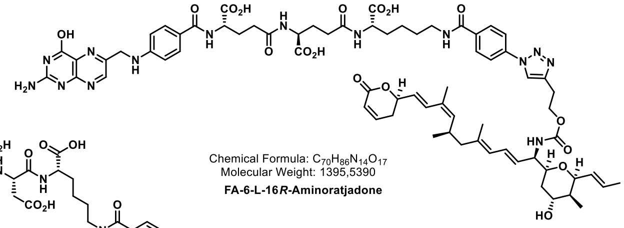

Chemical Formula: $C_{70}H_{86}N_{14}O_{17}$
Molecular Weight: 1395,5390
FA-6-L-16*R*-Aminoratjadone

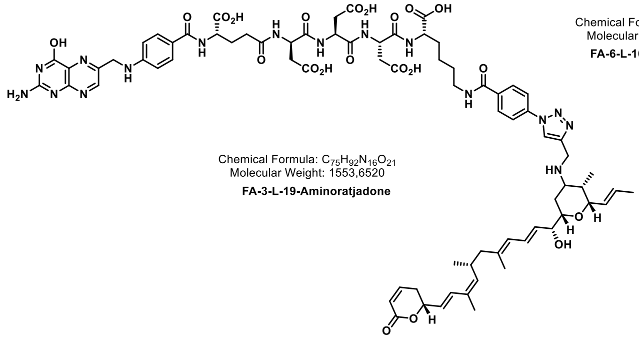

Chemical Formula: $C_{75}H_{92}N_{16}O_{21}$
Molecular Weight: 1553,6520
FA-3-L-19-Aminoratjadone

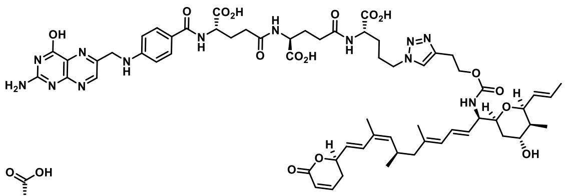

Chemical Formula: $C_{62}H_{79}N_{13}O_{16}$
Molecular Weight: 1262,3890
FA-7-L-16*R*-Aminoratjadone

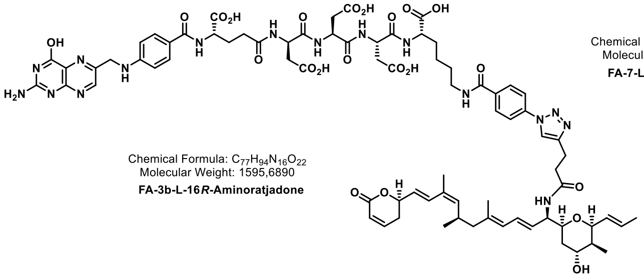

Chemical Formula: $C_{77}H_{94}N_{16}O_{22}$
Molecular Weight: 1595,6890
FA-3b-L-16*R*-Aminoratjadone

Figure S6: Continuation 3: Folate-Aminoratjadone conjugates synthesized in this study.

General procedure E for the synthesis of Folate-Aminoratjadone Conjugates via copper-free click reaction.

The corresponding **FA-N$_3$** (1.1 equiv) was dissolved in DMSO (0.2 M), a solution of BCN-O(CO)HN-Val-Cit-*p*ABO(CO)-16*R*-Aminoratjadone**25** (1.0 equiv) in DMSO (0.2 M) was added and the mixture was stirred for 4-20 h at 23°C under light exclusion until complete conversion was monitored by LCMS. The reaction mixture was diluted with 100 µL of MeOH, filtered through a Whatman filter (45 µm) and directly purified by RP prep HPLC (Phenomenex Gemini C18 RP-column 00G-4435-PO-AX, 5 µm, 110 A, 250×21.20 mm, Flow: 9 mL/min, ACN:H_2O + 0.1% TFA/ 10:90 → 95:5 in 45 min) yielding the Folate-Ratjadone Conjugates after lyophilization as a yellow, amorph solids.

FA-1-Val-Cit-*p*ABA-16*R*-Aminoratjadone

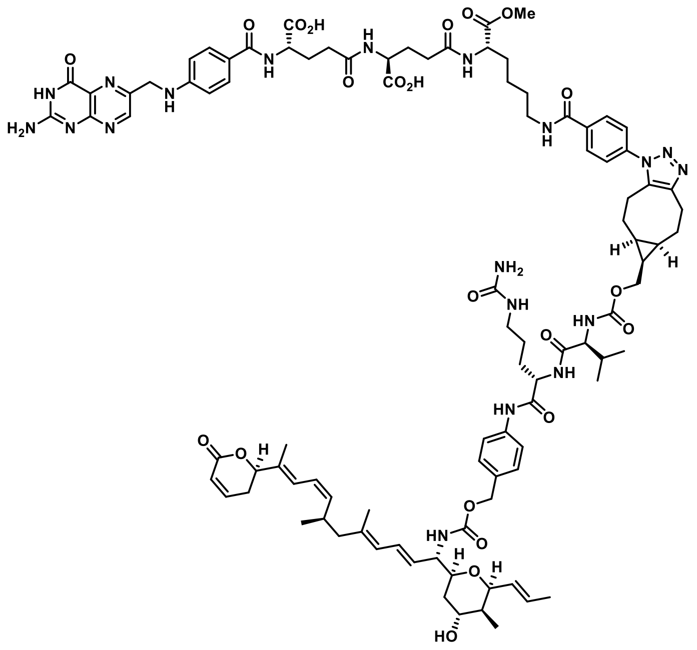

Chemical Formula: $C_{96}H_{123}N_{19}O_{22}$
Molecular Weight: 1895,15
FA-1-Val-Cit-*p*ABA-16*R*-Aminoratjadone

Applying **FA-N$_3$-1** to the general procedure E, 3.4 mg (17.49 µmol, 39%) **FA-1-Val-Cit-*p*ABA-16*R*-Aminoratjadone** was obtained as a mixture of diastereomers as a yellow, amorph solid.

FA-1-Val-Cit-*p*ABA-16*R*-Aminoratjadone: ^{1}H-NMR (700 MHz, DMSO-*d*$_6$) δ [ppm]: 12.67 – 12.39 (m, 2H), 12.08 (s, 1H), 11.40 (s, 1H), 10.02 (d, *J* = 11.7 Hz, 1H), 8.68 – 8.61 (m, 2H), 8.27 – 8.19 (m, 1H), 8.16 – 8.09 (m, 1H), 8.04 (s, 1H), 8.03 (d, *J* = 8.4 Hz, 2H), 7.66 (t, *J* = 9.3 Hz, 2H), 7.59 (d, *J* = 8.2 Hz, 2H), 7.58 – 7.54 (m, 2H), 7.32 – 7.23 (m, 3H), 7.13 (s, 1H), 7.03 (ddd, *J* = 9.7, 5.5, 2.8 Hz, 1H), 6.92 (q, *J* = 5.9, 4.8 Hz, 1H), 6.75 (d, *J* = 15.6 Hz, 1H), 6.66 – 6.61 (m, 2H), 6.31 (dd, *J* = 14.9, 10.8 Hz, 1H), 6.01 – 5.92 (m, 2H), 5.80 – 5.71 (m, 2H), 5.56 (dd, *J* = 15.0, 7.0 Hz, 2H), 5.40 (s, 2H), 5.34 (dd, *J* = 15.5, 3.7 Hz, 1H), 5.23 (d, *J* = 9.6 Hz, 1H), 5.09

(t, *J* = 5.4 Hz, 1H), 4.96 (d, *J* = 24.1 Hz, 2H), 4.72 (s, 1H), 4.48 (s, 2H), 4.41 (d, *J* = 7.7 Hz, 1H), 4.36 – 4.27 (m, 1H), 4.26 – 4.22 (m, 1H), 4.22 – 4.18 (m, 1H), 4.18 – 4.13 (m, 1H), 4.08 (d, *J* = 6.9 Hz, 4H), 4.03 (d, *J* = 7.2 Hz, 1H), 3.89 (d, *J* = 8.4 Hz, 1H), 3.71 (s, 1H), 3.65 (dd, *J* = 11.8, 6.5 Hz, 1H), 3.60 (t, *J* = 1.7 Hz, 3H), 3.29 – 3.23 (m, 2H), 3.17 (s, 3H), 3.11 – 3.05 (m, 1H), 3.01 (d, *J* = 6.7 Hz, 1H), 2.93 (dq, *J* = 13.3, 7.2, 6.4 Hz, 3H), 2.89 – 2.80 (m, 2H), 2.64 (t, *J* = 12.4 Hz, 1H), 2.53 – 2.51 (m, 1H), 2.47 – 2.41 (m, 1H), 2.32 – 2.22 (m, 2H), 2.21 – 2.13 (m, 3H), 2.11 – 2.03 (m, 2H), 1.98 (d, *J* = 7.0 Hz, 3H), 1.97 – 1.85 (m, 4H), 1.75 – 1.73 (m, 3H), 1.67 (s, 3H), 1.63 (s, 3H), 1.62 (s, 3H), 1.54 – 1.41 (m, 8H), 1.39 – 1.29 (m, 6H), 0.98 (s, 3H), 0.89 – 0.84 (m, 6H), 0.83 (dd, *J* = 6.5, 3.1 Hz, 6H), 0.73 (d, *J* = 7.1 Hz, 3H). **^{13}C-NMR** (176 MHz, DMSO-d_6) δ [ppm]: 174.46, 174.15, 174.13, 173.79, 173.74, 173.43, 173.39, 173.22, 173.12, 172.76, 172.75, 171.84, 171.81, 171.79, 171.66, 171.57, 171.53, 171.51, 171.27, 170.52, 166.42, 166.31, 165.11, 163.52, 160.79, 158.84, 157.69, 157.52, 156.56, 156.37, 155.69, 153.68, 150.75, 148.68, 148.45, 146.70, 144.19, 138.49, 138.21, 136.17, 135.33, 134.28, 132.03, 131.16, 129.62, 129.34, 129.05, 129.02, 128.99, 128.42, 128.31, 127.92, 127.20, 126.79, 125.79, 125.47, 124.53, 121.37, 121.34, 121.30, 121.28, 120.33, 118.85, 118.24, 116.53, 111.14, 77.82, 74.03, 73.55, 69.78, 68.19, 64.91, 61.70, 60.05, 60.01, 56.52, 53.00, 52.66, 52.53, 52.22, 51.94, 51.89, 51.87, 51.72, 51.47, 51.33, 51.30, 48.59, 47.21, 45.90, 41.37, 40.43, 38.65, 33.64, 31.87, 31.67, 31.35, 31.31, 31.28, 30.56, 30.46, 30.42, 30.37, 30.32, 29.58, 29.51, 29.48, 29.21, 29.00, 28.97, 28.88, 28.72, 28.68, 28.58, 28.56, 28.53, 26.74, 25.22, 24.48, 23.06, 22.86, 22.08, 21.99, 21.40, 20.79, 20.09, 19.33, 19.20, 18.84, 18.78, 18.17, 18.15, 17.64, 17.50, 16.42, 13.95, 11.07, 11.04. **LRMS** (ESI-Quad) [m/z]: 1896.0 $[M+H]^+$, **HRMS** (ESI-IT) [m/z]: 948.46350, calculated 948.46328 for $C_{96}H_{125}N_{19}O_{22}$ $[M+2H]^{2+}$, err [ppm] 0.231.

FA-2-Val-Cit-*p*ABA-16*R*-Aminoratjadone

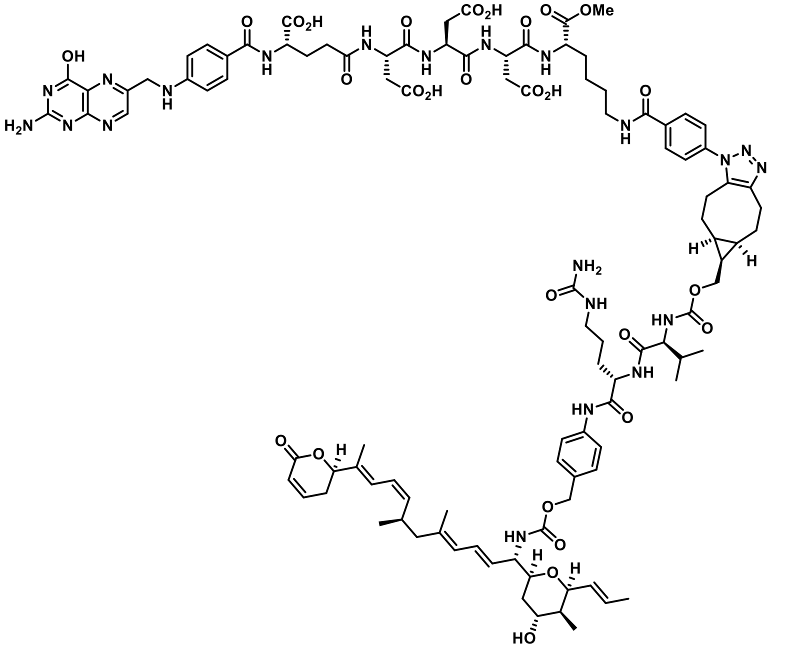

Chemical Formula: $C_{103}H_{131}N_{21}O_{28}$
Molecular Weight: 2111,30
FA-2-Val-Cit-*p*ABA-16*R*-Aminoratjadone

Applying **FA-N$_3$-2** to the general procedure E, 1.7 mg (0.805 µmol, 28%) **FA-2-Val-Cit-*p*ABA-16*R*-Aminoratjadone** was obtained as a mixture of diastereomers as a yellow, amorph solid.

FA-2-Val-Cit-*p*ABA-16*R*-Aminoratjadone: LRMS (ESI-Quad) [m/z]: 2112.9 $[M+H]^+$, **HRMS** (ESI-IT) [m/z]: 1056.4847, calculated 1056.4825 for $C_{103}H_{133}N_{21}O_{28}$ $[M+2H]^{2+}$, err [ppm] 2.082.

FA-3-Val-Cit-*p*ABA-16*R*-Aminoratjadone

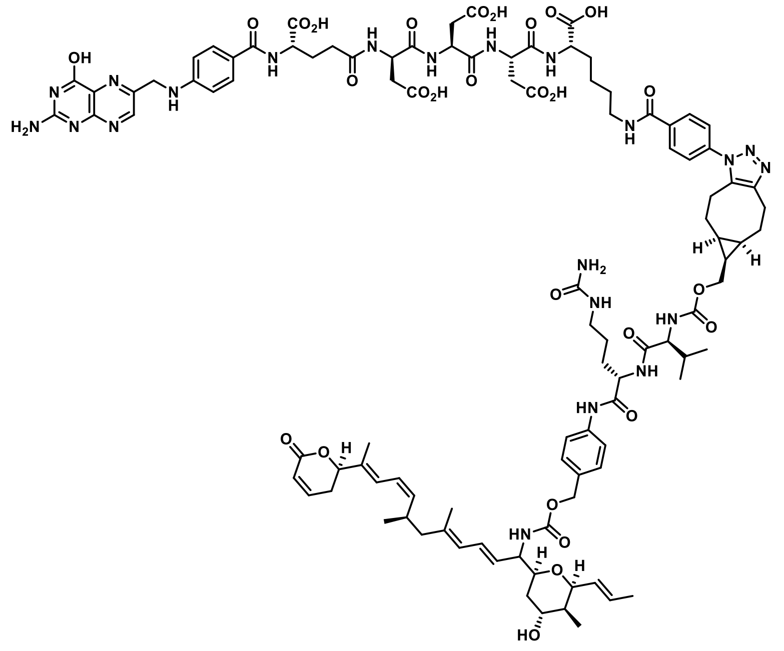

Chemical Formula: $C_{102}H_{129}N_{21}O_{28}$
Molecular Weight: 2097,2730

FA-3-Val-Cit-*p*ABA-16*R*-Aminoratjadone

Applying **FA-N$_3$-3** to the general procedure E, 3.3 mg (1.573 µmol, 54%) **FA-3-Val-Cit-*p*ABA-16*R*-Aminoratjadone** was obtained as a mixture of diastereomers as a yellow, amorph solid.

FA-3-Val-Cit-*p*ABA-16*R*-Aminoratjadone: ^{1}H-NMR (700 MHz, DMSO-d_6) δ [ppm]: 12.35 (s, 5H), 10.02 (d, *J* = 11.6 Hz, 1H), 8.67 (s, 1H), 8.63 (s, 1H), 8.48 – 8.10 (m, 3H), 8.03 (d, *J* = 8.2 Hz, 2H), 8.09 – 7.98 (m, 2H), 7.71 – 7.68 (m, 1H), 7.68 – 7.64 (m, 2H), 7.59 (d, *J* = 8.4 Hz, 3H), 7.56 (d, *J* = 8.9 Hz, 2H), 7.30 (d, *J* = 9.3 Hz, 1H), 7.27 (t, *J* = 8.2 Hz, 2H), 7.13 (t, *J* = 9.9 Hz, 1H), 7.03 (ddd, *J* = 9.6, 5.5, 2.8 Hz, 1H), 6.75 (d, *J* = 15.6 Hz, 1H), 6.64 (dd, *J* = 8.8, 3.3 Hz, 2H), 6.31 (dd, *J* = 15.0, 11.0 Hz, 1H), 6.02 – 5.92 (m, 2H), 5.75 (s, 2H), 5.79 – 5.71 (m, 2H), 5.57 (dt, *J* = 14.9, 7.3 Hz, 2H), 5.35 (dd, *J* = 15.4, 3.6 Hz, 1H), 5.23 (d, *J* = 9.5 Hz, 1H), 5.09 (dt, *J* = 10.4, 5.0 Hz, 1H), 4.98 (d, *J* = 11.9 Hz, 1H), 4.93 (d, *J* = 9.5 Hz, 1H), 4.60 – 4.52 (m, 2H), 4.50 (s, 2H), 4.48 – 4.43 (m, 1H), 4.41 (dt, *J* = 14.9, 8.4 Hz, 1H), 4.32 (dd, *J* = 13.3, 8.7 Hz, 1H), 4.24 (s, 1H), 4.23 – 4.19 (m, 1H), 4.17 – 4.11 (m, 2H), 4.10 – 4.07 (m, 2H), 4.03 (dd, *J* = 14.2, 6.8 Hz, 1H), 3.89 (dd, *J* = 15.3, 7.9 Hz, 1H), 3.71 (s, 2H), 3.68 – 3.61 (m, 1H), 3.36 – 3.22 (m, 2H), 3.13 – 3.04 (m, 1H), 3.04 – 2.98 (m, 1H), 2.96 – 2.88 (m,2), 2.85 (dt, *J* = 13.9, 6.9 Hz, 2H), 2.79 – 2.67 (m, 2H), 2.64 (dq, *J* = 13.1, 8.2, 7.0 Hz, 1H), 2.46 – 2.38 (m, 2H), 2.32 – 2.20 (m, 2H), 2.18 – 2.12 (m, 1H), 2.07 (t, *J* = 7.6 Hz, 2H), 1.98 (d, *J* = 7.1 Hz, 2H), 1.97 – 1.92 (m, 2H), 1.91 – 1.84 (m, 1H), 1.74 (s, 3H), 1.67 (s, 3H), 1.63 (d, *J* = 6.4 Hz, 6H), 1.60 – 1.56 (m, 2H), 1.55 – 1.51 (m, 2H), 1.51 – 1.47 (m, 2H), 1.45 (d, *J* = 12.8 Hz, 2H), 1.40 – 1.30 (m, 5H), 1.14 (dt, *J* = 14.3, 7.5 Hz, 2H), 0.98 (s, 3H), 0.87 (d, *J* = 6.6 Hz, 6H), 0.83 (dd, *J* = 6.5, 2.9 Hz, 6H), 0.73 (d, *J* = 7.1 Hz, 3H). **^{13}C-NMR** (176

MHz, DMSO-d_6) δ [ppm]: 206.11, 174.16, 174.12, 173.71, 173.69, 173.29, 173.25, 172.42, 172.15, 172.09, 172.05, 171.98, 171.88, 171.87, 171.85, 171.79, 171.75, 171.74, 171.69, 171.27, 171.26, 171.10, 171.00, 170.78, 170.52, 170.34, 170.32, 170.19, 166.32, 165.13, 163.53, 160.49, 158.87, 158.45, 158.24, 158.04, 157.84, 156.37, 155.70, 153.31, 150.86, 150.70, 149.41, 148.35, 146.71, 144.19, 138.50, 138.46, 138.20, 136.17, 135.35, 134.28, 132.04, 131.16, 129.34, 129.03, 128.45, 128.32, 127.95, 127.21, 126.80, 125.79, 125.46, 124.54, 121.34, 121.31, 120.33, 118.86, 118.82, 116.74, 115.07, 111.18, 77.82, 74.03, 73.55, 68.19, 64.91, 61.68, 60.05, 59.98, 56.52, 54.91, 53.00, 51.96, 49.77, 49.46, 47.22, 45.84, 40.02, 39.88, 39.76, 39.64, 39.52, 39.40, 39.28, 39.16, 38.65, 36.05, 35.99, 35.85, 35.79, 31.93, 31.15, 30.65, 30.39, 30.33, 30.27, 29.82, 29.58, 29.52, 29.48, 29.21, 29.01, 28.63, 26.74, 26.54, 26.34, 25.21, 23.08, 22.75, 21.99, 21.41, 20.80, 20.10, 19.34, 19.20, 18.84, 18.77, 18.16, 17.65, 17.51, 16.43, 15.72, 13.95, 11.08. **LRMS** (ESI-Quad) [m/z]: 1049.8 $[M+2H]^{2+}$, **HRMS** (ESI-IT) [m/z]: 1049.47480, calculated 1049.47475 for $C_{102}H_{131}N_{21}O_{28}$ $[M+2H]^{2+}$, err [ppm] 0.047.

FA-4-(Val-Cit-*p*ABA-16*R*-Aminoratjadone)$_2$

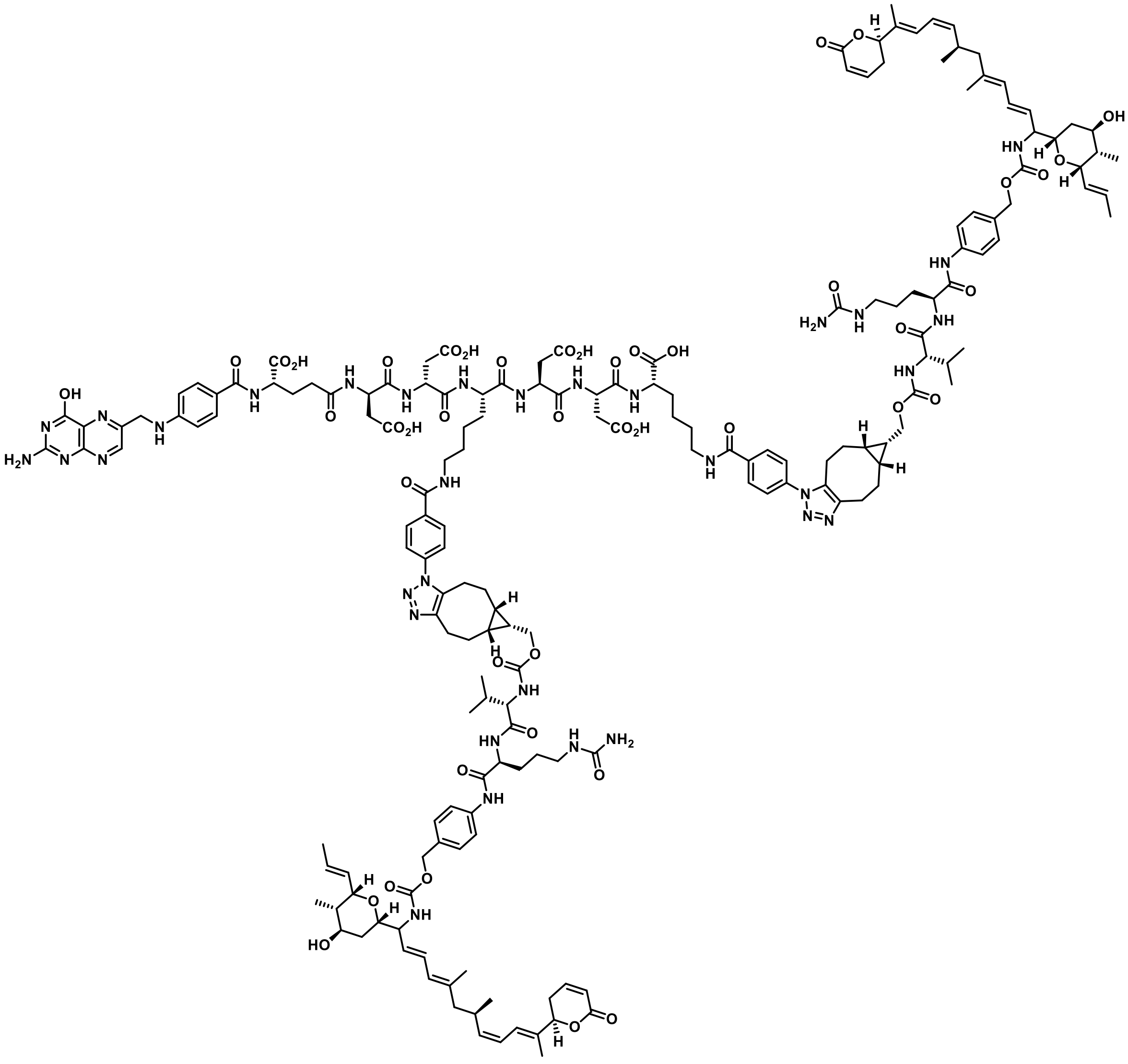

Chemical Formula: $C_{177}H_{229}N_{33}O_{44}$
Molecular Weight: 3522,97
FA-4-(Val-Cit-*p*ABA-16*R*-Aminoratjadone)$_2$

Applying **FA-N$_3$-4** to the general procedure E (modification: 2.2 equivof BCN-O(CO)HN-Val-Cit-*p*ABO(CO)-16*R*-Aminoratjadone**25**), 2.9 mg (0.80 µmol, 36%) **FA-4-(Val-Cit-*p*ABA-16*R*-Aminoratjadone)$_2$** was obtained as a mixture of diastereomers as a yellow, amorph solid.

FA-4-(Val-Cit-*p*ABA-16*R*-Aminoratjadone)$_2$: ^{1}H-NMR (500 MHz, DMSO-*d*$_6$) δ [ppm]: 12.37 (s, 6H), 10.03 (s, 1H), 10.01 (s, 1H), 8.66 (s, 1H), 8.64 – 8.62 (m, 1H), 8.62 – 8.59 (m, 1H), 8.43 – 7.92 (m, 4H), 8.02 (d, *J* = 8.1 Hz, 4H), 7.90 – 7.67 (m, 1H), 7.66 (d, *J* = 8.4 Hz, 2H), 7.60 – 7.54 (m, 6H), 7.43 – 7.22 (m, 5H), 7.19 – 7.08 (m, 2H), 7.03 (dq, *J* = 8.8, 2.8 Hz, 2H), 6.75 (d, *J* = 15.6 Hz, 2H), 6.64 (d, *J* = 8.3 Hz, 2H), 6.31 (dd, *J* = 14.9, 11.2 Hz, 2H), 5.97 (d, *J* = 9.8 Hz, 4H), 5.79 – 5.71 (m, 8H), 5.57 (dt, *J* = 14.7, 7.2 Hz, 4H), 5.38 – 5.29 (m, 2H),

5.23 (d, *J* = 9.5 Hz, 2H), 5.09 (dt, *J* = 10.4, 5.3 Hz, 2H), 4.98 (d, *J* = 11.9 Hz, 2H), 4.93 (d, *J* = 9.2 Hz, 2H), 4.58 (d, *J* = 6.1 Hz, 1H), 4.53 (s, 2H), 4.50 (s, 2H), 4.41 (dt, *J* = 14.7, 7.6 Hz, 2H), 4.32 (s, 1H), 4.24 (s, 2H), 4.17 (s, 1H), 4.14 (s, 2H), 4.08 (s, 3H), 4.06 – 4.01 (m, 3H), 3.92 – 3.86 (m, 6H), 3.85 – 3.73 (m, 8H), 3.71 (s, 4H), 3.66 (dd, *J* = 10.9, 5.0 Hz, 4H), 3.27 (s, 4H), 3.07 (s, 1H), 3.01 (s, 2H), 2.90 (d, *J* = 21.3 Hz, 3H), 2.88 – 2.81 (m, 3H), 2.79 – 2.57 (m, 5H), 2.46 – 2.37 (m, 4H), 2.33 – 2.20 (m, 2H), 2.14 (s, 2H), 2.07 (s, 2H), 1.98 (d, *J* = 7.0 Hz, 2H), 1.98 – 1.85 (m, 4H), 1.74 (s, 6H), 1.67 (s, 6H), 1.63 (d, *J* = 6.2 Hz, 6H), 1.60 – 1.31 (m, 22H), 1.18 – 1.10 (m, 4H), 0.97 (s, 4H), 0.86 (t, *J* = 7.0 Hz,12), 0.84 – 0.81 (m, 6H), 0.73 (d, *J* = 7.0 Hz, 6H). **^{13}C-NMR** (176 MHz, DMSO-*d*$_6$) δ [ppm]: 174.47, 174.23, 174.11, 173.74, 173.33, 173.28, 172.39, 172.06, 172.04, 172.02, 171.94, 171.86, 171.79, 171.73, 171.72, 171.66, 171.62, 171.59, 171.27, 170.67, 170.63, 170.58, 170.52, 170.45, 170.41, 170.36, 170.34, 166.34, 165.12, 163.53, 160.58, 158.86, 158.34, 158.14, 157.94, 157.74, 156.37, 155.69, 153.39, 150.82, 150.71, 149.27, 149.22, 148.39, 146.70, 144.18, 138.49, 138.19, 136.17, 135.34, 134.26, 132.04, 131.98, 131.29, 131.16, 129.46, 129.34, 129.20, 129.02, 128.44, 128.31, 128.23, 127.94, 127.20, 126.79, 125.84, 125.79, 125.54, 125.43, 124.53, 124.39, 121.33, 121.30, 120.33, 118.86, 111.17, 77.82, 74.03, 73.63, 73.55, 68.27, 68.18, 64.91, 63.07, 61.67, 60.04, 59.97, 56.52, 54.91, 53.00, 52.90, 52.88, 52.06, 52.03, 51.99, 49.95, 49.94, 49.92, 49.63, 49.62, 49.59, 49.56, 49.51, 49.46, 49.34, 47.33, 47.21, 45.85, 40.43, 38.65, 35.98, 35.86, 34.48, 33.65, 31.89, 31.86, 31.28, 31.19, 31.15, 30.65, 30.39, 30.36, 30.33, 30.27, 30.15, 29.88, 29.82, 29.58, 29.52, 29.47, 29.42, 29.31, 29.26, 29.21, 29.00, 28.97, 28.89, 28.72, 28.69, 28.63, 28.53, 28.50, 26.82, 26.73, 26.53, 25.20, 24.48, 23.07, 22.80, 22.08, 21.98, 21.40, 20.79, 20.09, 20.03, 19.36, 19.32, 19.20, 18.81, 18.78, 18.76, 18.18, 18.15, 17.65, 17.50, 16.42, 15.72, 13.95, 11.12, 11.08. **LRMS** (ESI-Quad) [m/z]: 1175.4 $[M+3H]^{3+}$, **HRMS** (ESI-IT) [m/z]: 1174.89793, calculated 1174.89827 for $C_{177}H_{232}N_{33}O_{44}$ $[M+3H]^{3+}$, err [ppm] -0.289.

FA-5-Val-Cit-*p*ABA-16*R*-Aminoratjadone

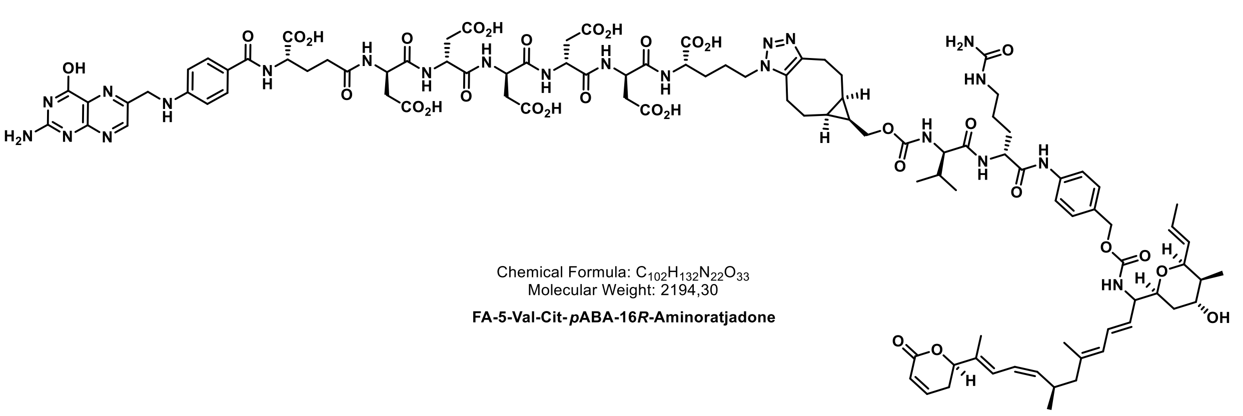

Applying **FA-N$_3$-5** to the general procedure E, 3.9 mg (1.78 µmol, 61%) **FA-5-Val-Cit-*p*ABA-16*R*-Aminoratjadone** was obtained as a mixture of diastereomers as a yellow, amorph solid.

FA-5-Val-Cit-*p*ABA-16*R*-Aminoratjadone: **^{1}H-NMR** (700 MHz, DMSO-d_6) δ [ppm]: 12.36 (s, 7H), 10.10 – 9.88 (m, 1H), 8.69 (s, 1H), 8.24 – 8.11 (m, 2H), 8.11 – 7.95 (m, 4H), 7.90 – 7.75 (m, 1H), 7.66 (d, *J* = 8.5 Hz, 2H), 7.60 – 7.51 (m, 2H), 7.48 – 7.34 (m, 1H), 7.30 (d, *J* = 9.2 Hz, 1H), 7.27 (d, *J* = 8.3 Hz, 2H), 7.17 – 7.08 (m, 1H), 7.04 (ddd, *J* = 9.6, 5.5, 2.8 Hz, 1H), 6.75 (d, *J* = 15.6 Hz, 1H), 6.64 (d, *J* = 8.6 Hz, 2H), 6.31 (dd, *J* = 14.4, 11.0 Hz, 1H), 6.01 (s, 1H), 5.98 – 5.95 (m, 1H), 5.77 (d, *J* = 11.2 Hz, 1H), 5.75 – 5.72 (m, 1H), 5.57 (dt, *J* = 14.8, 7.4 Hz, 2H), 5.38 – 5.32 (m, 1H), 5.23 (d, *J* = 9.5 Hz, 1H), 5.09 (dt, *J* = 10.4, 5.0 Hz, 1H), 4.99 (d, *J* = 12.4 Hz, 1H), 4.93 (d, *J* = 12.2 Hz, 1H), 4.59 – 4.52 (m, 2H), 4.54 – 4.47 (m, 4H), 4.41 (s, 2H), 4.30 (dd, *J* = 13.5, 8.3 Hz, 1H), 4.26 – 4.22 (m, 2H), 4.21 (s, 5H), 4.08 (d, *J* = 7.0 Hz, 1H), 4.04 (d, *J* = 6.3 Hz, 2H), 3.90 (dd, *J* = 14.9, 6.8 Hz, 1H), 3.71 (s, 2H), 3.66 (ddd, *J* = 11.9, 5.7, 2.2 Hz, 1H), 3.06 – 2.98 (m, 1H), 2.98 – 2.89 (m, 4H), 2.85 (dq, *J* = 14.0, 7.0 Hz, 1H), 2.79 – 2.63 (m, 8H), 2.45 (ddt, *J* = 18.6, 10.6, 2.7 Hz, 2H), 2.31 – 2.16 (m, 2H), 2.11 (s, 1H), 2.06 (d, *J* = 9.9 Hz, 2H), 1.99 (d, *J* = 7.1 Hz, 2H), 1.95 (dd, *J* = 13.6, 6.9 Hz, 2H), 1.91 – 1.86 (m, 1H), 1.74 (s, 3H), 1.67 (s, 3H), 1.63 (d, *J* = 6.5 Hz, 3H), 1.61 – 1.53 (m, 4H), 1.52 – 1.40 (m, 4H), 1.40 – 1.34 (m, 1H), 1.32 (d, *J* = 15.2 Hz, 1H), 1.15 – 1.09 (m, 1H), 0.97 – 0.88 (m, 3H), 0.89 – 0.85 (m, 6H), 0.83 (d, *J* = 6.7 Hz, 3H), 0.73 (d, *J* = 7.1 Hz, 3H). **^{13}C-NMR** (176 MHz, DMSO-d_6) δ [ppm]: 174.23, 174.10, 173.75, 173.73, 172.92, 172.13, 171.87, 171.83, 171.77, 171.59, 171.30, 170.95, 170.90, 170.79, 170.74, 170.70, 170.59, 170.54, 170.28, 170.26, 166.31, 163.54, 160.26, 158.93, 158.58, 158.37, 158.17, 157.96, 156.39, 155.71, 153.13, 150.81, 150.68, 149.88, 148.25, 146.72, 143.26, 138.50, 136.19, 132.95, 132.89, 132.05, 131.17, 129.35, 129.17, 129.03, 128.33, 127.97, 127.22, 126.80, 125.80, 124.55, 121.36, 120.34, 118.87, 116.56, 114.90, 111.21, 77.83, 74.04, 73.56, 68.20, 64.92, 61.71, 60.00, 59.95, 56.53, 55.06, 53.39, 53.04, 52.12, 51.36, 51.33, 49.72, 49.59, 49.40, 48.60, 47.22, 46.68, 45.83, 40.43, 40.02, 38.66, 36.08, 35.86, 35.83, 31.95, 31.29, 31.16, 30.41, 30.37, 30.26, 29.83, 29.59, 29.49, 29.22, 29.01, 28.70, 27.95, 26.73, 26.58, 25.88, 25.34, 25.25, 22.12, 21.78, 21.70, 21.04, 20.80, 20.10, 20.05, 19.21, 18.58, 18.42, 18.17, 17.66, 17.27, 16.44, 13.96, 11.09. **LRMS** (ESI-Quad) [m/z]: 1097.7 $[M+2H]^{2+}$, **HRMS** (ESI-IT) [m/z]: 1097.97430, calculated 1097.9753 for $C_{102}H_{134}N_{22}O_{33}$ $[M+2H]^{2+}$, err [ppm] -0.919.

FA-6-Val-Cit-*p*ABA-16*R*-Aminoratjadone

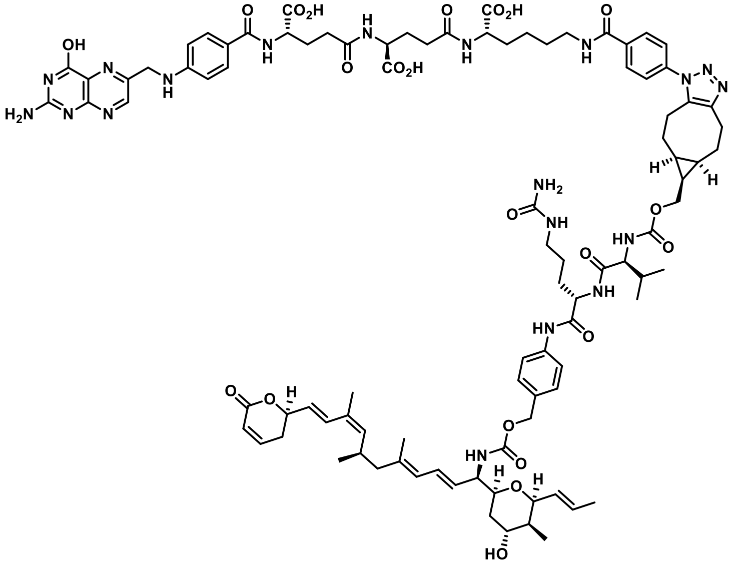

Chemical Formula: $C_{95}H_{121}N_{19}O_{22}$
Molecular Weight: 1881,12
FA-6-Val-Cit-*p*ABA-16*R*-Aminoratjadone

Applying **FA-N$_3$-6** to the general procedure E, 2.2 mg (1.17 µmol, 49%) **FA-6-Val-Cit-*p*ABA-16*R*-Aminoratjadone** was obtained as a mixture of diastereomers as a yellow, amorph solid.

FA-6-Val-Cit-*p*ABA-16*R*-Aminoratjadone: ^{1}H-NMR (700 MHz, DMSO-*d*$_6$) δ [ppm]: 12.57 (s, 3H), 10.11 – 9.94 (m, 1H), 8.66 (s, 1H), 8.22 (dd, *J* = 17.1, 7.7 Hz, 1H), 8.17 – 8.09 (m, 2H), 8.06 (s, 1H), 7.95 (d, *J* = 7.6 Hz, 1H), 7.67 (t, *J* = 7.5 Hz, 2H), 7.62 – 7.51 (m, 2H), 7.30 (d, *J* = 9.2 Hz, 1H), 7.27 (d, *J* = 6.8 Hz, 2H), 7.14 – 7.11 (m, 1H), 7.04 (dq, *J* = 9.4, 2.8 Hz, 2H), 6.75 (d, *J* = 15.5 Hz, 1H), 6.64 (d, *J* = 8.4 Hz, 2H), 6.31 (dd, *J* = 14.7, 11.0 Hz, 1H), 5.97 (d, *J* = 9.8 Hz, 2H), 5.77 (d, *J* = 11.3 Hz, 1H), 5.76 – 5.69 (m, 1H), 5.57 (dt, *J* = 14.8, 7.1 Hz, 2H), 5.42 (s, 1H), 5.35 (dd, *J* = 14.9, 4.2 Hz, 1H), 5.23 (d, *J* = 9.6 Hz, 1H), 5.09 (dt, *J* = 10.6, 5.4 Hz, 1H), 4.99 (d, *J* = 12.5 Hz, 1H), 4.93 (d, *J* = 12.1 Hz, 1H), 4.50 (s, 2H), 4.41 (s, 2H), 4.35 – 4.27 (m, 1H), 4.24 (s, 2H), 4.23 – 4.12 (m, 6H), 4.10 – 4.01 (m, 4H), 3.90 (dd, *J* = 14.4, 6.3 Hz, 2H), 3.71 (s, 2H), 3.01 (s, 1H), 2.94 (s, 3H), 2.85 (dt, *J* = 16.2, 7.1 Hz, 1H), 2.80 – 2.63 (m, 4H), 2.47 – 2.38 (m, 2H), 2.33 – 2.20 (m, 2H), 2.20 – 2.13 (m, 2H), 2.08 (d, *J* = 8.6 Hz, 2H), 1.99 (d, *J* = 7.1 Hz, 2H), 1.97 – 1.85 (m, 4H), 1.74 (s, 3H), 1.67 (s, 3H), 1.63 (d, *J* = 6.3 Hz, 3H), 1.60 – 1.52 (m, 6H), 1.51 – 1.47 (m, 3H), 1.45 (d, *J* = 12.2 Hz, 2H), 1.40 – 1.30 (m, 4H), 1.15 – 1.08 (m, 2H), 0.96 – 0.86 (m, 3H), 0.88 – 0.85 (m, 6H), 0.83 (d, *J* = 6.7 Hz, 3H), 0.73 (d, *J* = 7.1 Hz, 3H). **^{13}C-NMR** (176 MHz, DMSO-*d*$_6$) δ [ppm]: 174.17, 174.14, 173.82, 173.79, 173.76, 173.44, 173.43, 173.41, 173.13, 171.80, 171.41, 171.29, 170.53, 166.40, 166.30, 163.53, 160.67, 158.88, 158.09, 157.90, 156.38, 155.70, 153.48, 150.73, 149.07, 148.44, 148.37, 147.14, 146.71, 143.34, 138.49, 136.17, 132.83, 132.77, 132.03, 131.16, 129.34, 129.06, 129.02, 128.32, 127.95, 127.21, 126.80, 125.79, 124.54,

121.37, 121.35, 121.34, 121.31, 120.33, 118.86, 111.17, 77.82, 74.03, 73.55, 68.19, 64.91, 61.70, 59.99, 59.97, 56.52, 55.92, 53.03, 52.24, 51.99, 51.43, 51.28, 51.25, 47.22, 46.66, 45.87, 38.65, 33.65, 31.86, 31.68, 31.44, 31.38, 31.28, 30.46, 30.36, 29.58, 29.48, 29.31, 29.21, 29.01, 28.89, 28.72, 28.69, 28.53, 28.01, 27.17, 26.95, 26.74, 26.66, 26.50, 26.03, 25.36, 25.28, 24.48, 22.08, 21.81, 21.75, 21.16, 21.09, 20.79, 20.10, 19.20, 19.14, 18.63, 18.49, 18.17, 17.65, 17.27, 16.43, 13.95, 11.08. **LRMS** (ESI-Quad) [m/z]: 1882.6 $[M+H]^+$, **HRMS** (ESI-IT) [m/z]: 940.9543, calculated 940.9540 for $C_{95}H_{123}N_{19}O_{22}$ $[M+2H]^{2+}$, err [ppm] 0.318.

FA-7-Val-Cit-*p*ABA-16*R*-Aminoratjadone

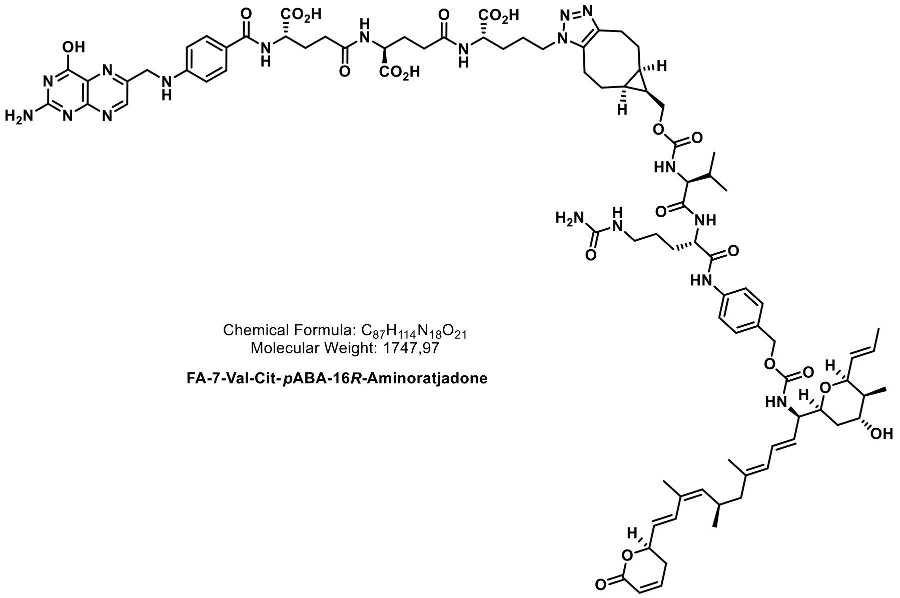

Applying **FA-N_3-7** to the general procedure E, 1.1 mg (0.629µmol, 26%) **FA-7-Val-Cit-*p*ABA-16*R*-Aminoratjadone** was obtained as a mixture of diastereomers as a yellow, amorph solid.

FA-7-Val-Cit-*p*ABA-16*R*-Aminoratjadone: ^{1}H-NMR (700 MHz, DMSO-d_6) δ [ppm]: 12.50 (s, 3H), 10.02 (d, *J* = 11.7 Hz, 1H), 8.66 (s, 1H), 8.66 – 8.62 (m, 1H), 8.24 – 7.93 (m, 5H), 8.03 (d, *J* = 8.3 Hz, 2H), 7.66 (d, *J* = 8.4 Hz, 1H), 7.70 – 7.60 (m, 2H), 7.59 (d, *J* = 8.2 Hz, 2H), 7.56 (d, *J* = 8.9 Hz, 2H), 7.30 (d, *J* = 9.3 Hz, 1H), 7.29 – 7.24 (m, 2H), 7.13 (t, *J* = 9.1 Hz, 1H), 7.03 (ddt, *J* = 8.5, 5.6, 2.8 Hz, 1H), 6.75 (d, *J* = 15.6 Hz, 1H), 6.66 – 6.61 (m, 2H), 6.31 (dd, *J* = 15.0, 11.1 Hz, 1H), 5.97 (d, *J* = 9.7 Hz, 2H), 5.77 (d, *J* = 11.0 Hz, 1H), 5.75 – 5.72 (m, 1H), 5.57 (dt, *J* = 14.8, 7.2 Hz, 2H), 5.40 (s, 1H), 5.34 (dd, *J* = 15.5, 3.5 Hz, 1H), 5.23 (d, *J* = 9.5 Hz, 1H), 5.09 (dt, *J* = 10.5, 5.2 Hz, 1H), 4.98 (d, *J* = 11.9 Hz, 1H), 4.93 (d, *J* = 9.6 Hz, 1H), 4.49 (s, 1H), 4.44 – 4.37 (m, 2H), 4.35 – 4.28 (m, 1H), 4.24 (s, 1H), 4.15 (dt, *J* = 14.9, 7.9 Hz, 2H), 4.08 (d, *J* = 7.2 Hz, 2H), 4.03 (d, *J* = 6.9 Hz, 1H), 3.89 (q, *J* = 7.6 Hz, 1H), 3.71 (s, 1H), 3.65 (ddd, *J* = 11.6, 5.4, 1.8 Hz, 2H), 3.27 (s, 2H), 3.11 – 3.05 (m, 1H), 3.04 – 2.98 (m, 1H), 2.92 (s, 2H), 2.88 – 2.81 (m, 2H), 2.67 – 2.60 (m, 1H), 2.48 – 2.41 (m, 2H), 2.32 –

2.22 (m, 2H), 2.23 – 2.13 (m, 3H), 2.07 (d, *J* = 3.0 Hz, 1H), 1.98 (d, *J* = 7.2 Hz, 2H), 1.92 (ddd, *J* = 43.1, 14.6, 6.9 Hz, 3H), 1.74 (s, 3H), 1.67 (s, 3H), 1.63 (d, *J* = 6.4 Hz, 3H), 1.60 – 1.40 (m, 10H), 1.39 – 1.30 (m, 5H), 1.18 – 1.09 (m, 1H), 0.98 (s, 2H), 0.87 (d, *J* = 6.5 Hz, 6H), 0.83 (dd, *J* = 6.4, 3.0 Hz, 3H), 0.73 (d, *J* = 7.0 Hz, 3H). **^{13}C-NMR** (176 MHz, DMSO-d_6) δ [ppm]: 174.14, 173.78, 173.76, 173.45, 173.42, 173.25, 173.15, 171.85, 171.81, 171.80, 171.68, 171.46, 171.41, 171.38, 171.27, 170.52, 166.39, 166.31, 165.10, 163.53, 160.73, 160.68, 158.86, 158.23, 158.04, 157.85, 157.65, 156.38, 155.70, 155.08, 154.90, 153.48, 150.72, 149.14, 149.08, 148.44, 146.71, 144.19, 138.50, 138.46, 138.21, 136.18, 135.35, 134.29, 132.92, 132.04, 131.29, 131.16, 129.35, 129.06, 129.03, 129.00, 128.43, 128.32, 128.21, 127.95, 127.78, 127.65, 127.21, 126.80, 125.79, 125.47, 124.54, 121.39, 121.35, 120.33, 118.86, 111.17, 77.82, 74.03, 73.55, 68.27, 68.19, 64.91, 61.68, 60.05, 59.99, 56.52, 53.00, 52.62, 52.24, 51.98, 51.76, 51.51, 51.36, 47.33, 47.22, 45.87, 38.65, 33.65, 31.87, 31.70, 31.47, 31.43, 31.34, 31.28, 31.20, 30.72, 30.47, 30.43, 30.38, 30.33, 30.15, 29.58, 29.51, 29.48, 29.26, 29.21, 29.01, 28.97, 28.89, 28.73, 28.69, 28.66, 28.64, 28.53, 27.20, 27.06, 26.98, 26.85, 26.75, 26.68, 25.22, 24.49, 23.07, 22.98, 22.09, 22.00, 21.43, 21.41, 20.80, 20.10, 20.04, 19.35, 19.34, 19.20, 19.14, 18.84, 18.78, 18.18, 18.16, 17.65, 17.51, 16.43, 15.72, 13.96, 11.08. **LRMS** (ESI-Quad) [m/z]: 1748.6 $[M+H]^+$, **HRMS** (ESI-IT) [m/z]: 874.4274, calculated 874.4276 for $C_{87}H_{116}N_{18}O_{21}$ $[M+2H]^{2+}$, err [ppm] -0.228.

FA-8-Val-Cit-*p*ABA-16*R*-Aminoratjadone

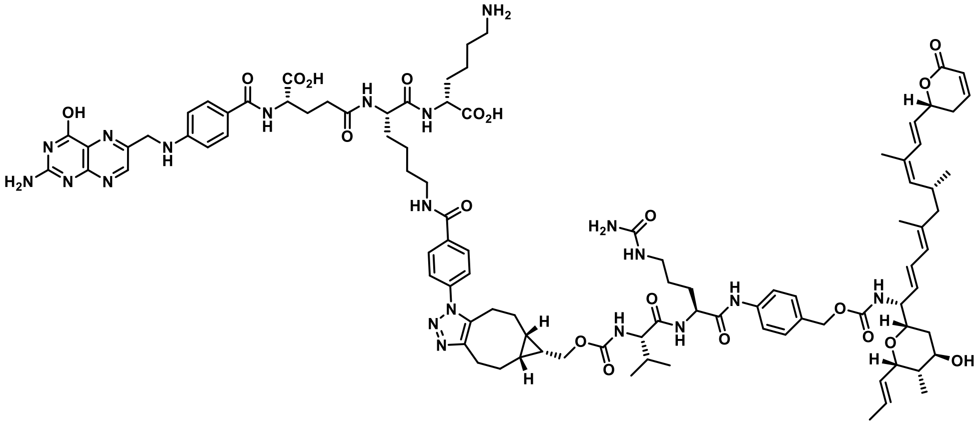

Chemical Formula: $C_{96}H_{126}N_{20}O_{20}$
Molecular Weight: 1880,18
FA-8-Val-Cit-*p*ABA-16*R*-Aminoratjadone

Applying **FA-N₃-8** to the general procedure E, 1.3 mg (0.691µmol, 32%) **FA-8-Val-Cit-*p*ABA-16*R*-Aminoratjadone** was obtained as a mixture of diastereomers of TFA salts as a yellow, amorph solid.

FA-8-Val-Cit-*p*ABA-16*R*-Aminoratjadone: ^{1}H-NMR (700 MHz, DMSO-*d*$_6$) δ [ppm]: 12.62 (s, 1H), 12.32 (d, *J* = 258.2 Hz, 1H), 11.48 (s, 1H), 10.02 (d, *J* = 13.1 Hz, 1H), 8.75 – 8.52 (m, 2H), 8.31 – 7.85 (m, 6H), 7.66 (d, *J* = 8.7 Hz, 2H), 7.64 – 7.52 (m, 6H), 7.32 – 7.22 (m, 3H), 7.13 (t, *J* = 9.3 Hz, 1H), 7.06 – 7.00 (m, 1H), 6.93 (s, 2H), 6.75 (d, *J* = 15.6 Hz, 1H), 6.64 (d, *J* = 8.6 Hz, 2H), 6.31 (dd, *J* = 15.0, 11.0 Hz, 1H), 5.99 – 5.94 (m, 2H), 5.77 (d, *J* = 11.0 Hz, 1H), 5.75 – 5.72 (m, 1H), 5.57 (dt, *J* = 14.9, 7.2 Hz, 2H), 5.41 (s, 2H), 5.34 (dd, *J* = 15.4, 3.6 Hz, 1H), 5.23 (d, *J* = 9.6 Hz, 1H), 5.09 (dt, *J* = 10.4, 4.9 Hz, 1H), 4.98 (d, *J* = 12.2 Hz, 1H), 4.93 (d, *J* = 9.2 Hz, 1H), 4.72 (s, 1H), 4.48 (s, 2H), 4.41 (dt, *J* = 13.7, 8.0 Hz, 1H), 4.36 – 4.14 (m, 4H), 4.07 (d, *J* = 17.7 Hz, 2H), 4.06 – 4.00 (m, 1H), 3.89 (q, *J* = 7.5 Hz, 1H), 3.71 (s, 1H), 3.66 (ddd, *J* = 11.8, 5.7, 2.0 Hz, 1H), 3.28 – 3.21 (m, 2H), 3.07 (s, 1H), 3.05 – 2.97 (m, 1H), 2.96 – 2.88 (m, 1H), 2.89 – 2.81 (m, 2H), 2.79 – 2.72 (m, 2H), 2.67 – 2.60 (m, 1H), 2.47 – 2.42 (m, 2H), 2.35 – 2.20 (m, 2H), 2.19 – 2.12 (m, 1H), 2.11 – 2.03 (m, 1H), 1.98 (d, *J* = 7.1 Hz, 2H), 1.97 – 1.82 (m, 2H), 1.74 (s, 3H), 1.67 (s, 3H), 1.63 (d, *J* = 6.4 Hz, 3H), 1.60 – 1.39 (m, 12H), 1.38 – 1.26 (m, 6H), 1.15 (dt, *J* = 14.9, 7.6 Hz, 2H), 0.98 (s, 2H), 0.89 – 0.84 (m, 6H), 0.84 – 0.81 (m, 3H), 0.73 (d, *J* = 7.1 Hz, 3H). **^{13}C-NMR** (176 MHz, DMSO-*d*$_6$) δ [ppm]: 174.47, 174.17, 174.01, 173.84, 173.78, 173.43, 173.40, 173.34, 172.14, 171.84, 171.81, 171.77, 171.58, 171.26, 170.52, 166.37, 166.30, 165.11, 163.53, 158.86, 158.05, 157.87, 157.69, 156.37, 155.69, 153.71, 150.79, 150.75, 148.60, 148.54, 146.71, 144.21, 138.50, 138.46, 138.23, 136.18, 135.32, 134.27, 132.05, 131.16, 129.35, 129.13, 129.09, 129.02, 128.42, 128.32, 127.93, 127.21, 126.80, 125.79, 125.49, 124.54, 121.24, 120.33, 118.85, 111.15, 77.83, 74.03, 73.55, 68.19, 64.90, 61.68, 60.04, 59.99, 56.52, 53.30, 52.99, 52.59, 52.33, 52.08, 51.98, 51.33, 47.22, 45.89, 40.02, 38.65, 31.82, 31.28, 30.48, 30.39, 30.34, 29.58, 29.52, 29.48, 29.21, 29.01, 28.89,

28.78, 28.69, 28.53, 26.75, 26.64, 26.43, 26.29, 25.21, 23.07, 22.92, 22.86, 22.23, 22.09, 22.00, 21.42, 20.79, 20.10, 19.35, 19.20, 18.86, 18.17, 18.14, 17.65, 17.51, 16.42, 15.72, 13.95, 11.08. **LRMS** (ESI-Quad) [m/z]: 940.6 $[M+2H]^{2+}$, **HRMS** (ESI-IT) [m/z]: 940.98325, calculated 940.98163 for $C_{96}H_{128}N_{20}O_{20}$ $[M+2H]^{2+}$, err [ppm] 1.76.

FA-9-Val-Cit-*p*ABA-16*R*-Aminoratjadone

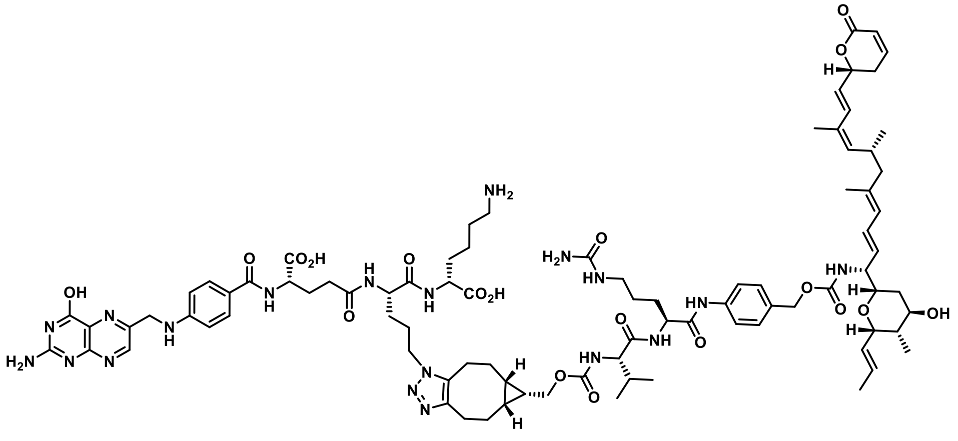

Chemical Formula: $C_{88}H_{119}N_{19}O_{19}$
Molecular Weight: 1747,03
FA-9-Val-Cit-*p*ABA-16*R*-Aminoratjadone

Applying **FA-N$_3$-9** to the general procedure E, 1.0 mg (0.572µmol, 26%) **FA-9-Val-Cit-PABA-16R-Aminoratjadone** was obtained as a mixture of diastereomers of TFA salts as a yellow, amorph solid.

FA-9-Val-Cit-*p*ABA-16*R*-Aminoratjadone: **^{1}H-NMR** (700 MHz, DMSO-*d*$_6$) δ [ppm]: 12.63 (s, 2H), 12.40 – 10.86 (m, 1H), 10.02 (s, 1H), 8.65 (s, 1H), 8.32 – 7.90 (m, 4H), 7.68 – 7.61 (m, 4H), 7.56 (d, *J* = 8.4 Hz, 2H), 7.30 (d, *J* = 8.8 Hz, 1H), 7.27 (d, *J* = 7.9 Hz, 2H), 7.14 – 7.09 (m, 1H), 7.03 (dq, *J* = 8.7, 2.8 Hz, 1H), 7.02 – 6.81 (m, 2H), 6.75 (d, *J* = 15.6 Hz, 1H), 6.64 (d, *J* = 8.5 Hz, 1H), 6.31 (dd, *J* = 14.9, 11.0 Hz, 1H), 5.98 (s, 1H), 5.97 (d, *J* = 9.9 Hz, 1H), 5.77 (d, *J* = 11.3 Hz, 1H), 5.75 – 5.71 (m, 1H), 5.57 (dt, *J* = 14.6, 7.3 Hz, 2H), 5.42 (s, 1H), 5.37 – 5.31 (m, 1H), 5.23 (d, *J* = 9.6 Hz, 1H), 5.09 (dt, *J* = 10.4, 5.4 Hz, 1H), 4.98 (d, *J* = 12.4 Hz, 1H), 4.93 (d, *J* = 12.4 Hz, 1H), 4.49 (s, 2H), 4.41 (s, 1H), 4.38 – 4.27 (m, 2H), 4.24 (s, 1H), 4.16 (d, *J* = 24.0 Hz, 3H), 4.09 (s, 2H), 4.03 (d, *J* = 7.3 Hz, 2H), 3.93 – 3.86 (m, 1H), 3.71 (s, 1H), 3.68 – 3.63 (m, 1H), 3.02 (s, 1H), 2.94 (s, 2H), 2.90 – 2.81 (m, 2H), 2.75 (s, 3H), 2.64 (s, 1H), 2.47 – 2.41 (m, 2H), 2.33 – 2.20 (m, 2H), 2.13 – 2.01 (m, 2H), 2.00 – 1.82 (m, 4H), 1.74 (s, 3H), 1.73 – 1.67 (m, 2H), 1.67 (s, 3H), 1.63 (d, *J* = 6.4 Hz, 3H), 1.60 – 1.41 (m, 10H), 1.40 – 1.25 (m, 4H), 1.16 – 1.05 (m, 8H), 0.94 – 0.84 (m, 3H), 0.83 (d, *J* = 6.6 Hz, 3H), 0.73 (d, *J* = 7.1 Hz, 3H). **^{13}C-NMR** (176 MHz, DMSO-*d*$_6$) δ [ppm]: 173.82, 173.76, 173.35, 173.32, 173.28, 173.27, 171.81, 171.64, 171.43, 171.39, 171.29, 171.07, 170.53, 166.31, 163.53, 160.85, 158.89, 158.19, 158.01, 157.82, 157.63, 156.36, 155.69, 153.63, 150.73, 148.78, 148.51, 146.71, 143.37, 138.49, 138.46, 136.18, 132.75,

132.05, 131.16, 129.35, 128.99, 128.32, 127.92, 127.22, 126.79, 125.79, 124.54, 121.35, 121.23, 120.33, 118.86, 117.43, 115.74, 111.15, 77.83, 74.03, 73.55, 68.19, 64.90, 61.69, 59.97, 59.92, 56.52, 53.01, 52.02, 51.96, 51.77, 51.35, 47.22, 46.82, 45.88, 40.15, 38.63, 33.65, 31.79, 31.75, 31.28, 30.50, 30.43, 30.38, 29.58, 29.48, 29.21, 29.00, 28.89, 28.72, 28.69, 28.53, 26.74, 26.63, 26.59, 26.48, 26.32, 26.00, 25.92, 25.37, 25.28, 24.48, 24.01, 22.20, 22.09, 21.81, 21.16, 20.79, 20.10, 20.04, 19.20, 19.14, 18.63, 18.54, 18.49, 18.14, 17.65, 17.28, 16.42, 13.95, 11.08. **LRMS** (ESI-Quad) [m/z]: 1748.0 $[M+H]^+$, **HRMS** (ESI-IT) [m/z]: 873.9538, calculated 873.9538 for $C_{88}H_{121}N_{19}O_{19}$ $[M+2H]^{2+}$, err [ppm] 0.0.

FA-10-Val-Cit-*p*ABA-16*R*-Aminoratjadone

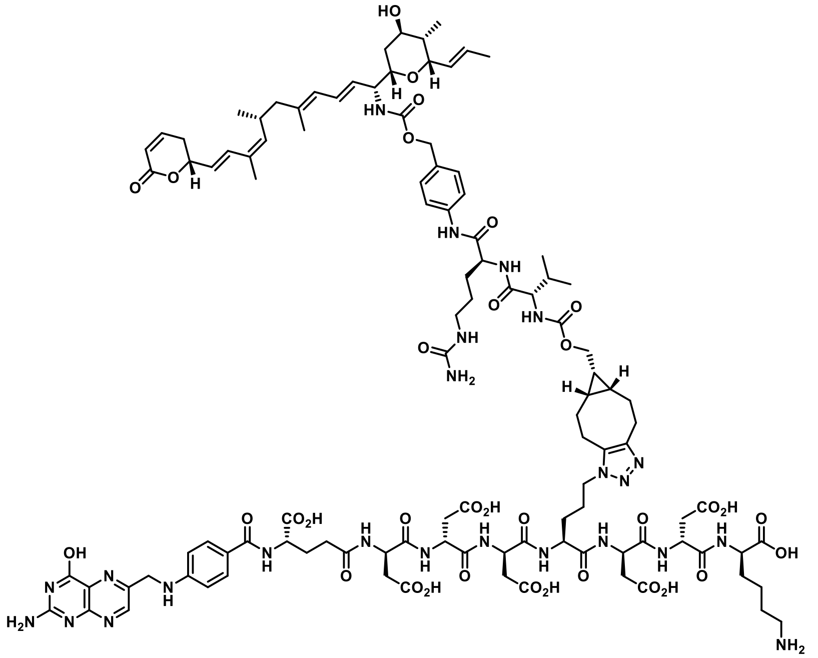

Chemical Formula: $C_{108}H_{144}N_{24}O_{34}$
Molecular Weight: 2322,47
FA-10-Val-Cit-*p*ABA-16*R*-Aminoratjadone

Applying **FA-N$_3$-10** to the general procedure E, 1.8 mg (0.78 µmol, 24%) **FA-10-Val-Cit-*p*ABA-16*R*-Aminoratjadone** was obtained as a mixture of diastereomers of TFA salts as a yellow, amorph solid.

FA-10-Val-Cit-*p*ABA-16*R*-Aminoratjadone: ^{1}H-NMR (700 MHz, DMSO-*d*$_6$) δ [ppm]: ^{1}H NMR (700 MHz, DMSO-*d*$_6$) δ 12.39 (s, 6H), 11.50 (s, 1H), 10.03 (s, 1H), 8.65 (s, 1H), 8.49 – 7.96 (m, 6H), 7.79 (s, 1H), 7.69 – 7.64 (m, 2H), 7.60 (s, 2H), 7.58 – 7.56 (m, 2H), 7.32 – 7.22 (m, 1H), 7.27 (d, *J* = 7.5 Hz, 2H), 7.11 (s, 1H), 7.04 (dq, *J* = 9.5, 2.8 Hz, 1H), 6.94 (s, 1H), 6.75 (d, *J* = 15.6 Hz, 1H), 6.64 (d, *J* = 8.7 Hz, 2H), 6.36 – 6.27 (m, 1H), 6.00 (s, 1H), 5.97 (d, *J* = 9.8 Hz, 1H), 5.77 (d, *J* = 11.4 Hz, 1H), 5.76 – 5.72 (m, 1H), 5.56 (dd, *J* = 14.8, 7.0 Hz, 2H), 5.43 (s, 1H), 5.36 (d, *J* = 3.7 Hz, 1H), 5.23 (d, *J* = 9.6 Hz, 1H), 5.15 – 5.06 (m, 1H), 4.99 (d, *J* = 12.3 Hz, 1H), 4.93 (d, *J* = 12.2 Hz, 1H), 4.56 (s, 3H), 4.49 (s, 2H), 4.41 (s, 1H), 4.31 (s, 1H), 4.24 (s, 2H), 4.17 (s, 2H),

4.10 (s, 1H), 4.04 (s, 2H), 3.90 (d, *J* = 7.4 Hz, 1H), 3.71 (s, 1H), 3.65 (s, 1H), 3.02 (s, 1H), 2.94 (s, 2H), 2.85 (dt, *J* = 16.1, 7.2 Hz, 1H), 2.81 – 2.62 (m, 9H), 2.46 – 2.36 (m, 2H), 2.20 (d, *J* = 33.7 Hz, 1H), 2.05 (s, 2H), 1.99 (d, *J* = 7.0 Hz, 2H), 1.96 (d, *J* = 6.3 Hz, 2H), 1.74 (s, 3H), 1.71 (s, 4H), 1.67 (s, 3H), 1.63 (d, *J* = 6.3 Hz, 3H), 1.60 – 1.40 (m, 10H), 1.39 – 1.28 (m, 4H), 1.12 (s, 2H), 0.94 (s, 3H), 0.87 (d, *J* = 6.4 Hz, 6H), 0.83 (d, *J* = 6.6 Hz, 3H), 0.73 (d, *J* = 7.0 Hz, 3H). **^{13}C-NMR** (176 MHz, DMSO-d_6) δ [ppm]: ^{13}C NMR (176 MHz, DMSO) δ 173.73, 173.21, 171.94, 171.94, 171.91, 171.86, 171.84, 171.76, 171.73, 171.71, 171.62, 171.30, 171.05, 170.54, 170.47, 170.40, 170.36, 166.48, 166.34, 163.54, 160.88, 159.42, 158.93, 158.73, 158.16, 157.98, 157.80, 157.61, 156.37, 155.70, 153.64, 151.84, 150.88, 150.75, 148.78, 148.76, 148.72, 148.52, 147.49, 146.72, 143.27, 138.50, 138.50, 138.47, 136.19, 136.19, 131.16, 129.35, 129.03, 128.32, 127.94, 127.22, 126.80, 125.79, 124.54, 120.33, 118.86, 111.17, 77.83, 74.03, 73.56, 68.19, 64.91, 61.72, 59.94, 59.92, 59.87, 56.53, 54.91, 53.03, 52.50, 52.40, 52.02, 51.66, 51.60, 51.49, 49.89, 49.85, 49.82, 49.79, 49.73, 49.65, 49.60, 49.48, 47.22, 46.85, 45.88, 38.65, 35.95, 35.90, 35.71, 31.93, 31.88, 31.29, 30.44, 30.38, 30.28, 29.59, 29.49, 29.22, 29.01, 28.69, 26.73, 26.57, 26.46, 25.37, 25.31, 22.20, 22.08, 21.75, 21.70, 21.05, 20.80, 20.10, 19.20, 18.38, 18.15, 17.66, 17.24, 16.43, 13.96, 13.05, 11.08. **LRMS** (ESI-Quad) [m/z]: 1162.1 $[M+H]^+$, **HRMS** (ESI-IT) [m/z]: 775.01786, calculated 775.01762 for $C_{108}H_{147}N_{24}O_{34}$ $[M+3H]^{3+}$, err [ppm] 0.309.

FA-11-(Val-Cit-*p*ABA-16*R*-Aminoratjadone)$_3$

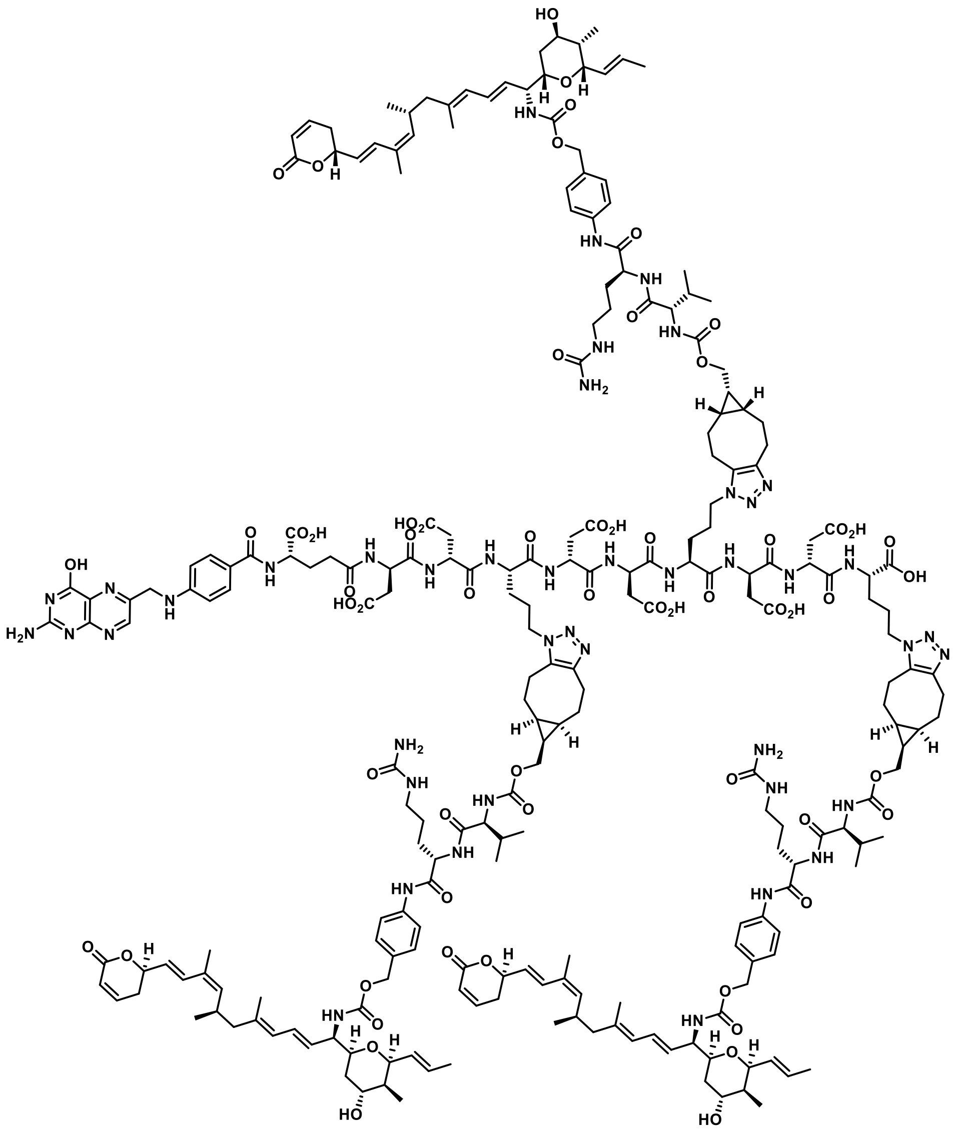

Chemical Formula: $C_{232}H_{313}N_{43}O_{60}$
Molecular Weight: 4664,30
FA-11-(Val-Cit- *p*ABA-16*R*-Aminoratjadone)$_3$

Applying **FA-N$_3$-11** to the general procedure E (modification 3.3 equivof BCN-O(CO)HN-Val-Cit-*p*ABO(CO)-16*R*-Aminoratjadone**25**), 4.7 mg (1.007 µmol, 54%) **FA-11-(Val-Cit-*p*ABA-16*R*-Aminoratjadone)$_3$** was obtained as a mixture of diastereomers as a yellow, amorph solid.

FA-11-(Val-Cit-*p*ABA-16*R*-Aminoratjadone)$_3$: ^{1}H-NMR (700 MHz, DMSO-*d*$_6$) δ [ppm]: 12.41 (s, 8H), 10.03 (s, 4H), 8.70 (s, 1H), 8.22 (s, 8H), 8.11 – 8.03 (m, 4H), 7.87 (s, 3H), 7.78 (s, 2H), 7.66 (d, *J* = 8.1 Hz, 2H), 7.57 (d, *J* = 8.2 Hz, 3H), 7.32 – 7.22 (m, 8H), 7.17 (d, *J* = 7.1 Hz, 12H), 7.16 – 7.13 (m, 1H), 7.13 – 7.07 (m, 1H), 7.03 (dq, *J* = 8.5, 2.8 Hz, 3H), 7.00 (s, 1H), 6.75 (d, *J* = 15.6 Hz, 3H), 6.64 (d, *J* = 7.4 Hz, 2H), 6.31 (dd, *J* = 14.7, 11.2 Hz, 3H), 6.03 (s, 3H), 5.97 (d, *J* = 9.7 Hz, 3H), 5.77 (d, *J* = 11.4 Hz, 2H), 5.73 (d, *J* = 6.6 Hz, 3H), 5.57 (dt,

J = 14.6, 7.1 Hz, 6H), 5.38 – 5.30 (m, 3H), 5.23 (d, *J* = 9.5 Hz, 3H), 5.09 (dt, *J* = 10.3, 5.4 Hz, 3H), 4.99 (d, *J* = 12.3 Hz, 3H), 4.93 (d, *J* = 12.1 Hz, 3H), 4.79 – 4.66 (m, 2H), 4.58 (s, 3H), 4.53 (s, 3H), 4.41 (s, 3H), 4.31 (d, *J* = 31.4 Hz, 3H), 4.24 (s, 1H), 4.22 (s, 3H), 4.17 (s, 3H), 4.09 (s, 3H), 4.06 – 3.97 (m, 3H), 3.90 (s, 7H), 3.71 (s, 3H), 3.66 (dd, *J* = 10.8, 4.5 Hz, 3H), 3.01 (s, 3H), 2.98 – 2.90 (m, 9H), 2.85 (dt, *J* = 13.9, 7.0 Hz, 3H), 2.78 – 2.72 (m, 5H), 2.70 – 2.64 (m, 7H), 2.57 – 2.50 (m, 3H), 2.46 – 2.39 (m, 6H), 2.15 – 2.01 (m, 6H), 2.00 – 1.92 (m, 10H), 1.74 (s, 9H), 1.72 – 1.68 (m, 6H), 1.67 (s, 9H), 1.63 (d, *J* = 6.4 Hz, 9H), 1.60 – 1.52 (m, 14H), 1.51 – 1.47 (m, 6H), 1.45 (d, *J* = 12.3 Hz, 6H), 1.40 – 1.35 (m, 3H), 1.32 (d, *J* = 13.3 Hz, 3H), 1.11 (s, 6H), 0.97 – 0.89 (m, 6H), 0.87 (d, *J* = 6.4 Hz, 18H), 0.83 (d, *J* = 6.3 Hz, 9H), 0.73 (d, *J* = 7.0 Hz, 9H). **^{13}C-NMR** (176 MHz, DMSO-d_6)) δ [ppm]: 173.76, 172.97, 171.96, 171.87, 171.75, 171.72, 171.69, 171.62, 171.58, 171.46, 171.44, 171.30, 171.20, 171.12, 171.06, 171.03, 171.00, 170.95, 170.91, 170.89, 170.84, 170.54, 170.42, 170.40, 170.34, 170.21, 170.16, 166.32, 163.54, 160.05, 158.96, 158.88, 158.60, 158.39, 158.18, 157.97, 156.37, 156.17, 155.71, 152.98, 150.65, 150.27, 150.23, 148.15, 146.71, 143.24, 143.18, 138.85, 138.50, 137.34, 136.19, 132.92, 132.05, 132.00, 131.30, 131.26, 131.17, 129.47, 129.35, 129.24, 129.20, 129.17, 129.16, 129.04, 129.00, 128.90, 128.83, 128.33, 128.24, 128.20, 128.11, 127.96, 127.22, 126.80, 126.55, 126.53, 125.85, 125.80, 125.31, 124.55, 124.40, 121.34, 120.34, 118.87, 118.07, 116.42, 114.77, 113.11, 111.21, 77.83, 74.25, 74.04, 73.64, 73.56, 68.29, 68.20, 64.92, 61.71, 59.94, 59.89, 56.53, 54.91, 54.22, 53.04, 52.11, 52.08, 52.06, 52.04, 52.00, 51.33, 49.93, 49.89, 49.85, 49.81, 49.75, 49.73, 49.69, 49.64, 49.60, 49.54, 49.46, 49.42, 49.38, 49.36, 49.32, 47.34, 47.23, 47.15, 46.92, 46.70, 46.68, 45.81, 40.02, 38.66, 36.31, 36.28, 36.15, 36.08, 36.02, 36.00, 35.96, 35.92, 35.58, 31.88, 31.29, 31.16, 30.45, 30.40, 30.00, 29.89, 29.83, 29.60, 29.49, 29.33, 29.23, 29.01, 28.90, 28.73, 28.70, 28.54, 27.97, 26.81, 26.72, 26.54, 25.85, 25.67, 25.63, 25.31, 25.26, 22.22, 22.14, 21.76, 21.70, 21.13, 21.05, 20.92, 20.80, 20.61, 20.56, 20.11, 20.05, 19.20, 18.59, 18.55, 18.43, 18.16, 17.66, 17.27, 16.44, 15.73, 13.96, 11.13, 11.09. **LRMS** (ESI-Quad) [m/z]: 1555.5 $[M+3H]^{3+}$, **HRMS** (ESI-IT) [m/z]: 1167.07950, calculated 1166.82785 for $C_{232}H_{317}N_{43}O_{60}$ $[M+4H]^{4+}$, 933.864661, calculated 933.663738 for $C_{232}H_{318}N_{43}O_{60}$ $[M+5H]^{5+}$

General procedure F for the synthesis of Folate-Aminoratjadone Conjugates via copper-mediated click reaction.

The corresponding **FA-N$_3$** (1.1 equiv) and the corresponding Ratjadone payload (**26**, **21, 34** or **20**) (1.0 equiv) were dissolved in a mixture of DMSO:H_2O:tBuOH/2:1:1 were added DiPEA (6.0 eq), TBTA (0.1 eq, 10 µL from a stock solution in DMSO), $CuSO_4$ (0.05 eq, 10 µL from a stock solution in H_2O) and sodium ascorbate (0.5 eq, 10 µL from a stock solution in H_2O) and the mixture was stirred under light exclusion for 4-24 h at 23°C until complete conversion was monitored by LCMS. The reaction mixture was diluted with 100 µL of MeOH, filtered through a Whatman filter (45 µm) and directly purified by RP prep HPLC

(Phenomenex Gemini C18 RP-column 00G-4435-PO-AX, 5 µm, 110 A, 250×21.20 mm, Flow: 9 mL/min, ACN:H_2O + 0.1% TFA/ 10:90 → 95:5 in 45 min) yielding the Folate-Ratjadone Conjugates after lyophilization as yellow, amorph solids.

FA-3-L-16*R*-Aminoratjadone

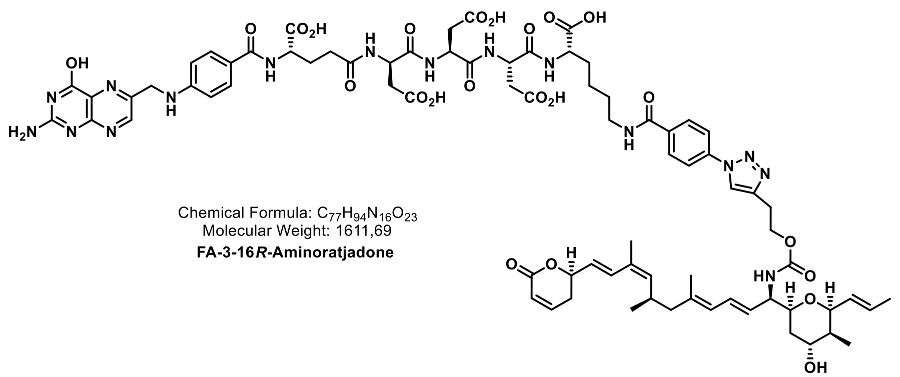

Applying **FA-N$_3$-3** and 16R-Aminoratjadone derivative **21** to the general procedure F, 2.7 mg (1.58 µmol, 42%) **FA-3-16*R*-Aminoratjadone** was obtained as a mixture of diastereomers a yellow, amorph solid.

FA-3-L-16R-Aminoratjadone: ^{1}H-NMR (700 MHz, DMSO-*d*$_6$) δ [ppm]: 12.31 (s, 5H), 11.42 (s, 1H), 8.70 (s, 1H), 8.64 (s, 1H), 8.59 (s, 1H), 8.39 – 8.10 (m, 3H), 8.04 (d, *J* = 8.2 Hz, 2H), 8.02 – 7.97 (m, 2H), 7.96 (d, *J* = 8.3 Hz, 2H), 7.73 – 7.57 (m, 3H), 7.25 (d, *J* = 9.2 Hz, 1H), 7.09 – 6.82 (m, 3H), 6.74 (d, *J* = 15.7 Hz, 1H), 6.64 (d, *J* = 8.3 Hz, 2H), 6.34 – 6.27 (m, 1H), 5.97 (d, *J* = 9.5 Hz, 2H), 5.74 (dt, *J* = 15.9, 6.5 Hz, 2H), 5.62 – 5.49 (m, 2H), 5.34 (d, *J* = 14.4 Hz, 1H), 5.22 (d, *J* = 9.6 Hz, 1H), 5.09 (dt, *J* = 10.4, 5.1 Hz, 1H), 4.72 (s, 1H), 4.62 – 4.51 (m, 2H), 4.51 – 4.42 (m, 3H), 4.36 – 4.20 (m, 4H), 4.14 (s, 1H), 4.00 (s, 1H), 3.70 (s, 1H), 3.67 – 3.56 (m, 2H), 3.04 – 3.01 (m, 2H), 2.88 – 2.80 (m, 1H), 2.79 – 2.64 (m, 2H), 2.46 (s, 4H), 2.33 – 2.16 (m, 2H), 1.97 (d, *J* = 6.3 Hz, 2H), 1.88 (d, *J* = 39.7 Hz, 2H), 1.74 (s, 1H), 1.73 (s, 3H), 1.64 (s, 3H), 1.62 (d, *J* = 5.7 Hz, 3H), 1.57 – 1.41 (m, 5H), 1.40 – 1.27 (m, 5H), 0.86 (d, *J* = 6.2 Hz, 3H), 0.84 – 0.77 (m, 2H), 0.70 (d, *J* = 6.8 Hz, 3H). **^{13}C-NMR** (176 MHz, DMSO-*d*$_6$) δ [ppm]: 174.52, 174.16, 173.76, 173.29, 172.79, 172.43, 172.30, 172.16, 172.03, 171.93, 171.91, 171.81, 171.26, 171.13, 170.37, 166.37, 164.99, 163.58, 163.05, 160.89, 160.86, 157.77, 157.60, 157.43, 156.58, 156.54, 155.65, 153.74, 150.99, 150.96, 150.79, 149.97, 148.71, 148.46, 148.45, 146.74, 144.90, 138.53, 138.35, 136.24, 134.23, 131.19, 129.39, 129.03, 128.92, 127.94, 127.31, 126.81, 125.79, 124.60, 121.05, 120.34, 119.31, 118.26, 116.55, 111.20, 77.87, 74.08, 73.60, 68.21, 62.49, 56.52, 52.06, 52.01, 49.84, 49.80, 49.79, 49.54, 49.52, 47.23, 45.91, 35.81, 33.68, 31.30, 31.22, 30.69, 30.32, 30.17, 29.72, 29.59, 29.24, 29.04, 29.03, 28.99, 28.91, 28.74, 28.71, 28.68, 28.55, 26.58, 25.53, 24.51, 22.81, 22.11, 20.82, 20.11, 17.65, 16.40, 13.98, 11.08. **LRMS** (ESI-Quad) [m/z]: 1612.4 [M+H]$^{+}$, **HRMS** (ESI-IT) [m/z]: 806.3405, calculated 806.3412 for $C_{77}H_{96}N_{16}O_{23}$ [M+2H]$^{2+}$, err [ppm] -0.639.

FA-3-L-19-Aminoratjadone

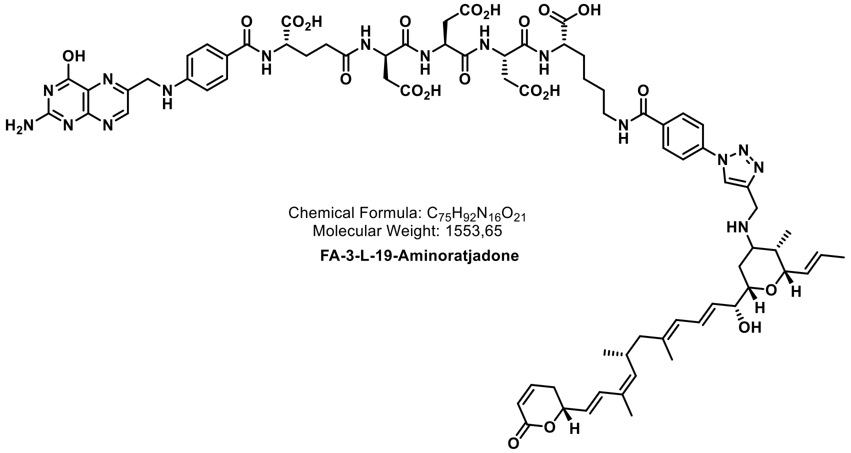

Applying **FA-N$_3$-3** and 19-Aminoratjadone derivative **34** to the general procedure F, 1.2 mg (0.77 µmol, 10%) **FA-3-L-19-Aminoratjadone** was obtained as a yellow, amorph solid.

FA-3-L-19-Aminoratjadone: LRMS (ESI-Quad) [m/z]: 777.8 $[M+2H]^{2+}$, **HRMS** (ESI-IT) [m/z]: 777.3384, calculated 777.3384 for $C_{75}H_{94}N_{16}O_{21}$ $[M+2H]^{2+}$, err [ppm] 0.0.

FA-3b-L-16*R*-Aminoratjadone

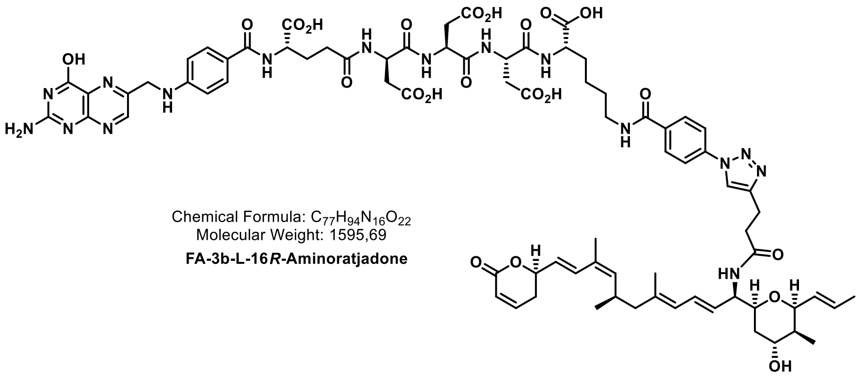

Applying **FA-N$_3$-3** and 16R-Aminoratjadone derivative **21** to the general procedure F, 1.9 mg (1.19 µmol, 17%) **FA-3b-L-16R-Aminoratjadone** was obtained as a yellow, amorph solid.

FA-3b-L-16R-Aminoratjadone: ^{1}H-NMR (700 MHz, DMSO-d_6) δ [ppm]: 12.33 (s, 5H), 11.58 (s, 1H), 8.65 (s, 1H), 8.58 (s, 1H), 8.57 (s, 1H), 8.48 – 8.11 (m, 3H), 8.03 (d, *J* = 8.3 Hz, 2H), 8.00 (d, *J* = 8.6 Hz, 1H), 7.97 (d, *J* = 9.0 Hz, 1H), 7.95 (d, *J* = 8.6 Hz, 2H), 7.70 – 7.68 (m, 1H), 7.67 – 7.65 (m, 2H), 7.04 (ddd, *J* = 9.6, 5.6, 2.8 Hz, 1H), 6.94 (s, 2H), 6.74 (d, *J* = 15.6 Hz, 1H), 6.67 – 6.57 (m, 1H), 6.27 (dd, *J* = 15.1, 11.0 Hz, 1H), 6.02 – 5.95 (m, 1H), 5.75 (d, *J* = 7.1 Hz, 1H), 5.73 (d, *J* = 6.7 Hz, 1H), 5.62 – 5.52 (m, 2H), 5.34 (ddd, *J* = 15.4, 5.5, 1.6 Hz, 1H), 5.21 (d, *J* = 9.5 Hz, 1H), 5.09 (dt, *J* = 10.5, 5.5 Hz, 1H), 4.62 – 4.43 (m, 6H), 4.35 – 4.29 (m, 1H),

4.24 – 4.21 (m, 1H), 4.16 – 4.11 (m, 1H), 3.68 – 3.65 (m, 1H), 3.65 – 3.59 (m, 1H), 3.30 – 3.23 (m, 2H), 2.95 (t, *J* = 7.5 Hz, 2H), 2.86 – 2.62 (m, 6H), 2.45 (ddt, *J* = 18.6, 10.6, 2.6 Hz, 2H), 2.32 – 2.17 (m, 2H), 2.10 – 2.03 (m, 1H), 1.96 (d, *J* = 7.1 Hz, 2H), 1.89 – 1.81 (m, 1H), 1.73 (s, 3H), 1.62 (d, *J* = 9.3 Hz, 6H), 1.56 – 1.50 (m, 3H), 1.47 (q, *J* = 9.1, 7.6 Hz, 2H), 1.44 – 1.39 (m, 2H), 1.38 – 1.32 (m, 3H), 1.30 – 1.28 (m, 1H), 1.27 (s, 1H), 0.85 (d, *J* = 6.6 Hz, 3H), 0.83 (d, *J* = 6.7 Hz, 1H), 0.71 (d, *J* = 7.1 Hz, 3H). **^{13}C-NMR** (176 MHz, DMSO-*d*$_6$) δ [ppm]: 174.16, 174.12, 173.74, 173.71, 173.70, 173.25, 172.26, 172.14, 172.09, 171.98, 171.93, 171.89, 171.87, 171.76, 171.74, 171.09, 170.99, 170.77, 170.63, 170.33, 170.25, 167.02, 166.33, 164.95, 163.55, 160.78, 158.26, 158.04, 157.85, 157.66, 153.57, 150.91, 150.74, 148.90, 148.49, 147.49, 146.73, 138.54, 138.36, 136.06, 134.09, 131.24, 129.41, 129.02, 128.99, 128.87, 127.95, 127.08, 126.78, 125.79, 124.61, 121.31, 121.28, 120.33, 120.24, 119.17, 111.17, 77.88, 74.09, 73.67, 68.21, 53.94, 52.00, 51.96, 49.76, 49.46, 47.21, 45.87, 38.74, 36.05, 35.99, 35.86, 35.80, 34.67, 31.93, 31.83, 31.28, 30.65, 30.27, 29.81, 29.56, 29.23, 29.01, 28.67, 26.55, 22.78, 21.44, 20.79, 20.08, 17.64, 16.38, 15.62, 13.96, 11.06. **LRMS** (ESI-Quad) [m/z]: 1596.7 [M+H]$^+$, **HRMS** (ESI-IT) [m/z]: 798.34173, calculated 798.34370 for $C_{77}H_{96}N_{16}O_{22}$ [M+H]$^+$, err [ppm] -2.41.

FA-5-L-16*R*-Aminoratjadone

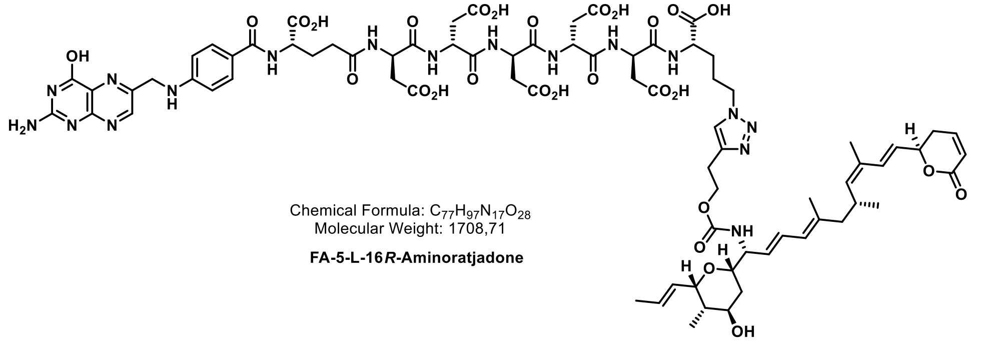

Applying **FA-N$_3$-5** and 16R-Aminoratjadone derivative **21** to the general procedure F, 1.1 mg (0.68 µmol, 18%) **FA-5-L-16*R*-Aminoratjadone** was obtained as a yellow, amorph solid.

FA-5-L-16*R*-Aminoratjadone: ^{1}H-NMR (700 MHz, DMSO-*d*$_6$) δ [ppm]: 12.37 (s, 7H), 11.55 (s, 1H), 8.66 (s, 1H), 8.24 – 8.12 (m, 3H), 8.12 – 7.95 (m, 4H), 7.88 (s, 2H), 7.86 – 7.78 (m, 1H), 7.66 (d, *J* = 8.0 Hz, 2H), 7.21 (d, *J* = 9.0 Hz, 1H), 7.07 – 7.01 (m, 3H), 7.10 – 6.86 (m, 1H), 6.75 (d, *J* = 15.6 Hz, 1H), 6.67 – 6.60 (m, 2H), 6.31 (dd, *J* = 15.1, 11.0 Hz, 1H), 6.03 – 5.93 (m, 1H), 5.77 (d, *J* = 10.2 Hz, 1H), 5.76 – 5.72 (m, 1H), 5.62 – 5.53 (m, 2H), 5.38 – 5.33 (m, 1H), 5.24 (d, *J* = 9.6 Hz, 1H), 5.10 (dt, *J* = 10.4, 5.4 Hz, 1H), 4.59 – 4.50 (m, 4H), 4.33 – 4.24 (m, 3H), 4.24 (s, 2H), 4.21 – 4.12 (m, 4H), 3.98 (d, *J* = 7.3 Hz, 1H), 3.72 (s, 1H), 3.64 (s, 1H), 2.90 (q, *J* = 6.9 Hz, 2H), 2.85 (dt, *J* = 14.0, 7.1 Hz, 1H), 2.81 – 2.63 (m, 6H), 2.56 – 2.51 (m, 2H), 2.44 (ddd, *J* =

18.6, 8.0, 5.4 Hz, 2H), 2.34 – 2.15 (m, 3H), 2.10 – 2.02 (m, 1H), 1.99 (d, *J* = 7.0 Hz, 2H), 1.95 – 1.85 (m, 2H), 1.84 – 1.75 (m, 2H), 1.74 (s, 3H), 1.67 (s, 3H), 1.63 (d, *J* = 6.4 Hz, 3H), 1.62 – 1.54 (m, 2H), 1.52 – 1.41 (m, 4H), 1.33 (d, *J* = 12.6 Hz, 1H), 0.87 (d, *J* = 6.6 Hz, 3H), 0.85 (d, *J* = 7.2 Hz, 1H), 0.72 (d, *J* = 7.1 Hz, 3H). **^{13}C-NMR** (176 MHz, DMSO-*d*$_6$) δ [ppm]: 174.21, 174.08, 173.74, 173.72, 172.83, 171.87, 171.83, 171.81, 171.57, 170.53, 170.27, 170.22, 166.80, 166.31, 163.53, 160.76, 158.07, 157.88, 155.62, 153.55, 150.85, 150.72, 148.92, 148.48, 146.71, 143.14, 138.51, 136.18, 132.92, 131.18, 129.34, 129.15, 129.01, 128.78, 127.94, 127.30, 126.79, 125.81, 124.54, 122.38, 120.33, 111.16, 77.82, 74.05, 73.53, 68.19, 62.78, 56.52, 51.28, 49.64, 49.58, 49.54, 48.73, 47.21, 45.86, 40.42, 38.65, 36.07, 35.84, 31.93, 31.28, 30.25, 29.73, 29.56, 29.20, 29.00, 28.69, 27.93, 26.56, 26.17, 25.52, 22.08, 20.77, 20.09, 20.03, 17.64, 16.41, 11.08. **LRMS** (ESI-Quad) [m/z]: 1709.9 [M+H]$^+$, **HRMS** (ESI-IT) [m/z]: 854.8417, calculated 854.8417 for $C_{77}H_{99}N_{17}O_{28}$ [M+2H]$^{2+}$, err [ppm] 0.0.

FA-6-L-16*R*-Aminoratjadone

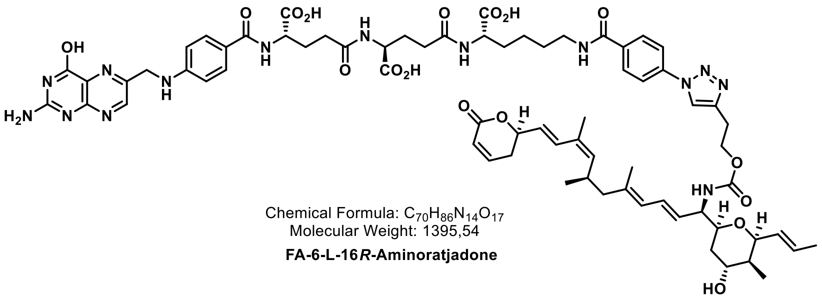

Chemical Formula: $C_{70}H_{86}N_{14}O_{17}$
Molecular Weight: 1395,54
FA-6-L-16*R*-Aminoratjadone

Applying **FA-N$_3$-6** and 16R-Aminoratjadone derivative **21** to the general procedure F, 3.6 mg (2.58 µmol, 64%) **FA-6-L-16*R*-Aminoratjadone** was obtained as a yellow, amorph solid.

FA-6-L-16*R*-Aminoratjadone: ^{1}H-NMR (700 MHz, DMSO-*d*$_6$) δ [ppm]: 12.51 (s, 3H), 11.60 (s, 1H), 8.71 (s, 1H), 8.66 (s, 1H), 8.63 – 8.53 (m, 1H), 8.21 (dd, *J* = 12.0, 7.9 Hz, 1H), 8.17 – 8.13 (m, 1H), 8.12 (t, *J* = 8.1 Hz, 1H), 8.10 – 8.05 (m, 1H), 8.04 (d, *J* = 8.6 Hz, 2H), 7.96 (d, *J* = 8.5 Hz, 2H), 7.67 (t, *J* = 8.5 Hz, 2H), 7.25 (d, *J* = 8.9 Hz, 1H), 7.06 – 6.99 (m, 2H), 7.20 – 6.85 (m, 1H), 6.74 (d, *J* = 15.6 Hz, 1H), 6.64 (t, *J* = 7.3 Hz, 2H), 6.30 (dd, *J* = 14.7, 11.2 Hz, 1H), 6.01 – 5.93 (m, 1H), 5.75 (s, 2H), 5.75 – 5.71 (m, 1H), 5.61 – 5.53 (m, 2H), 5.34 (d, *J* = 15.5 Hz, 1H), 5.22 (d, *J* = 9.6 Hz, 1H), 5.09 (dt, *J* = 10.5, 5.4 Hz, 1H), 4.49 (s, 2H), 4.35 – 4.29 (m, 1H), 4.29 – 4.24 (m, 2H), 4.23 (s, 1H), 4.15 (s, 2H), 4.00 (d, *J* = 7.0 Hz, 1H), 3.70 (s, 1H), 3.64 (s, 1H), 3.31 – 3.21 (m, 2H), 3.03 (t, *J* = 6.5 Hz, 2H), 2.84 (dt, *J* = 14.9, 6.8 Hz, 1H), 2.55 – 2.52 (m, 1H), 2.44 (ddt, *J* = 18.5, 10.6, 2.6 Hz, 2H), 2.33 – 2.22 (m, 2H), 2.17 (ddd, *J* = 16.4, 12.5, 8.4 Hz, 2H), 2.11 – 2.03 (m, 1H), 1.97 (d, *J* = 6.5 Hz, 2H), 1.95 – 1.84 (m, 2H), 1.73 (s, 3H), 1.71 (s, 1H), 1.64 (s, 3H), 1.62 (d, *J* = 6.0 Hz, 3H), 1.57 – 1.41 (m, 5H), 1.39 – 1.31 (m, 3H), 0.86 (d, *J* = 6.3 Hz, 3H), 0.82 (s, 1H), 0.71 (d, *J* = 7.0 Hz, 3H). **^{13}C-NMR** (176 MHz,

DMSO-d_6) δ [ppm]: 174.16, 174.13, 173.78, 173.76, 173.45, 173.41, 173.25, 173.15, 171.85, 171.81, 171.80, 171.69, 171.45, 171.41, 171.39, 166.39, 166.31, 164.92, 163.53, 160.69, 158.37, 158.18, 157.98, 157.79, 155.62, 153.49, 150.72, 149.07, 148.44, 146.70, 144.88, 138.51, 138.33, 136.20, 134.20, 131.18, 129.37, 129.07, 129.01, 128.87, 127.95, 127.27, 126.79, 125.78, 124.56, 121.40, 121.34, 121.03, 120.33, 119.28, 111.18, 74.06, 73.58, 68.19, 62.46, 56.50, 54.91, 52.62, 52.24, 51.76, 51.52, 51.37, 47.22, 45.87, 40.02, 38.66, 31.88, 31.71, 31.43, 31.34, 30.73, 30.47, 29.71, 29.57, 29.22, 29.02, 28.70, 27.21, 26.98, 26.68, 26.52, 25.51, 23.01, 20.79, 20.09, 17.63, 16.37, 11.06. **LRMS** (ESI-Quad) [m/z]: 1396.3 $[M+H]^+$, **HRMS** (ESI-IT) [m/z]: 698.32208, calculated 698.32204 for $C_{70}H_{87}N_{14}O_{17}$ $[M+2H]^{2+}$, err [ppm] 0.057.

FA-7-L-16*R*-Aminoratjadone

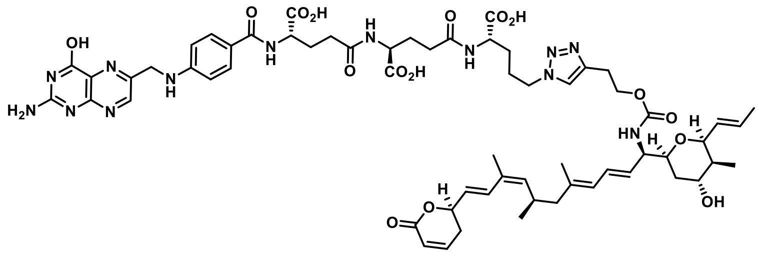

Chemical Formula: $C_{62}H_{79}N_{13}O_{16}$
Molecular Weight: 1262,39
FA-7-L-16*R*-Aminoratjadone

Applying **FA-N$_3$-7** and 16R-Aminoratjadone derivative **21** to the general procedure F, 2.4 mg (1.90 µmol, 48%) **FA-7-L-16*R*-Aminoratjadone** was obtained as a yellow, amorph solid.

FA-7-L-16*R*-Aminoratjadone: ^{1}H-NMR (700 MHz, DMSO-d_6) δ [ppm]: 12.52 (s, 3H), 8.67 (s, 1H), 8.26 – 8.03 (m, 3H), 7.99 – 7.81 (m, 2H), 7.70 – 7.58 (m, 2H), 7.21 (d, *J* = 8.8 Hz, 1H), 7.04 (ddd, *J* = 9.5, 5.5, 2.8 Hz, 1H), 6.75 (d, *J* = 15.6 Hz, 1H), 6.64 (t, *J* = 6.8 Hz, 2H), 6.31 (dd, *J* = 15.0, 11.1 Hz, 1H), 5.97 (d, *J* = 9.7 Hz, 1H), 5.80 – 5.67 (m, 2H), 5.63 – 5.52 (m, 2H), 5.35 (dd, *J* = 15.4, 3.6 Hz, 1H), 5.23 (d, *J* = 9.5 Hz, 1H), 5.09 (dt, *J* = 10.4, 5.4 Hz, 1H), 4.51 (s, 2H), 4.47 – 4.38 (m, 1H), 4.29 (s, 3H), 4.24 (s, 2H), 4.21 – 4.14 (m, 4H), 4.01 – 3.96 (m, 1H), 3.72 (s, 1H), 3.64 (d, *J* = 6.0 Hz, 1H), 2.90 (t, *J* = 6.5 Hz, 2H), 2.85 (dt, *J* = 15.8, 7.1 Hz, 1H), 2.48 – 2.40 (m, 2H), 2.33 – 2.12 (m, 4H), 1.99 (d, *J* = 6.9 Hz, 2H), 1.97 – 1.86 (m, 2H), 1.81 (d, *J* = 6.8 Hz, 2H), 1.74 (s, 3H), 1.67 (s, 3H), 1.63 (d, *J* = 6.1 Hz, 3H), 1.56 – 1.40 (m, 4H), 1.33 (d, *J* = 13.2 Hz, 1H), 0.87 (d, *J* = 6.5 Hz, 3H), 0.82 (s, 1H), 0.72 (d, *J* = 7.1 Hz, 3H). **^{13}C-NMR** (176 MHz, DMSO-d_6) δ [ppm]: 174.20, 173.40, 171.87, 171.47, 166.36, 163.60, 155.69, 153.42, 150.74, 148.43, 146.77, 143.20, 138.56, 136.25, 131.20, 129.40, 129.04, 127.97, 127.34, 126.82, 125.82, 124.60, 122.50, 122.45, 121.44, 121.39, 121.36, 121.32, 120.35, 111.22, 77.88, 74.08, 73.59, 68.23, 62.81, 56.55, 54.93, 51.50, 51.32, 48.85, 47.26, 45.87, 38.69, 31.46, 31.39, 31.34, 30.48, 29.76, 29.60, 29.24, 28.04, 26.95, 26.45, 25.56, 20.82, 20.13, 17.68, 16.44, 11.11.

LRMS (ESI-Quad) [m/z]: 1263.8 $[M+H]^+$, **HRMS** (ESI-IT) [m/z]: 631.79626, calculated 631.79566 for $C_{62}H_{81}N_{13}O_{16}$ $[M+2H]^{2+}$, err [ppm] -0.949.

FA-8-L-16*R*-Aminoratjadone

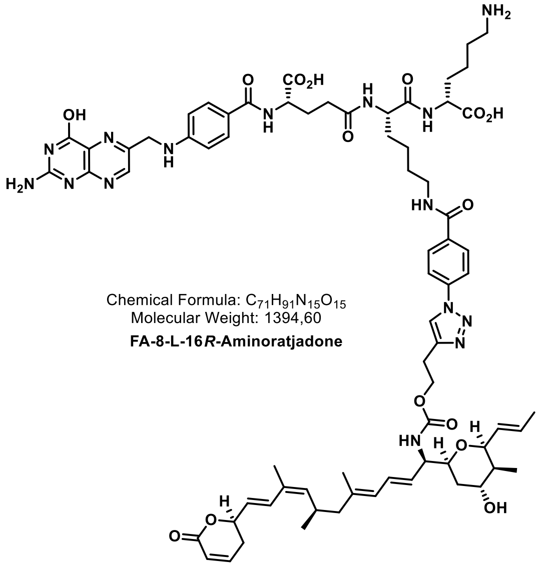

Applying **FA-N$_3$-8** and 16R-Aminoratjadone derivative **21** to the general procedure F, 1.4 mg (1.00 µmol, 28%) **FA-8-16*R*-Aminoratjadone** was obtained as a TFA salt as a yellow, amorph solid.

FA-8-L-16*R*-Aminoratjadone: LRMS (ESI-Quad) [m/z]: 698.2 $[M+2H]^{2+}$, **HRMS** (ESI-IT) [m/z]: 697.84802, calculated 697.84823 for $C_{23}H_{34}N_5O_6$ $[M+H]^+$, err [ppm] -0.288.

FA-9-L-16*R*-Aminoratjadone

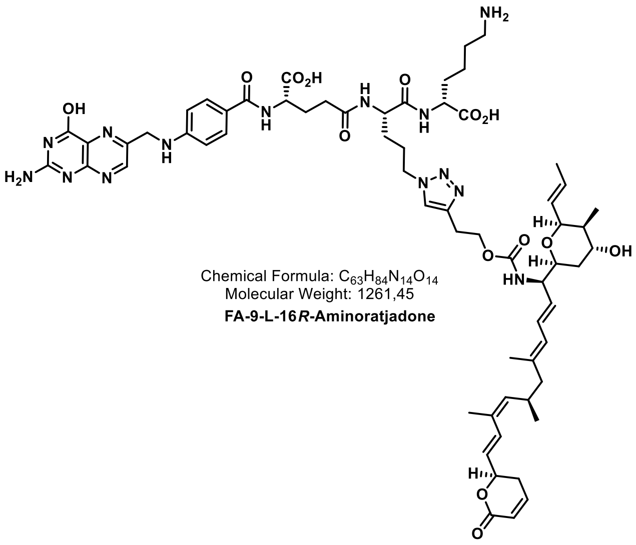

Applying **FA-N$_3$-9** and 16*R*-Aminoratjadone derivative **21** to the general procedure F, 1.6 mg (1.26 µmol, 35%) **FA-9-L-16*R*-Aminoratjadone** was obtained as a TFA salt as a yellow, amorph solid.

FA-9-L-16*R*-Aminoratjadone: LRMS (ESI-Quad) [m/z]: 1261.9 $[M+H]^+$, **HRMS** (ESI-IT) [m/z]: 613.3218, calculated 613.3218 for $C_{63}H_{86}N_{14}O_{14}$ $[M+H]^+$, err [ppm] 0.0.

FA-10-L-16*R*-Aminoratjadone

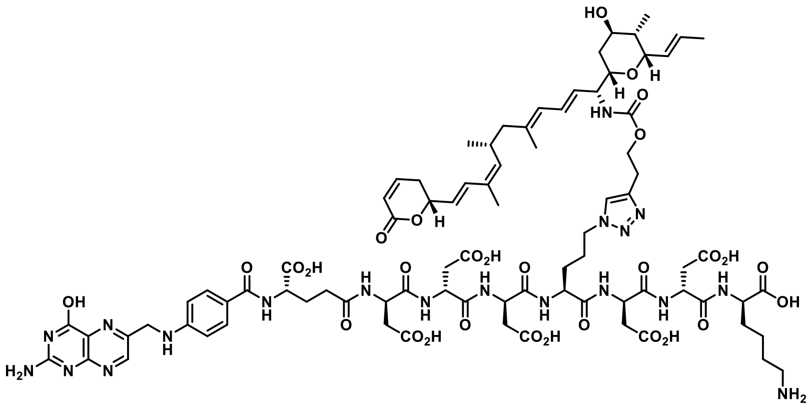

Chemical Formula: $C_{83}H_{109}N_{19}O_{29}$
Molecular Weight: 1836,89
FA-10-L-16*R*-Aminoratjadone

Applying **FA-N$_3$-10** and 16R-Aminoratjadone derivative **21** to the general procedure F, 2.5 mg (1.36 µmol, 34%) **FA-10-L-16*R*-Aminoratjadone** was obtained as a TFA salt as a yellow, amorph solid.

FA-10-L-16*R*-Aminoratjadone: ^{1}H-NMR (700 MHz, DMSO-d_6) δ [ppm]: 12.37 (s, 7H), 11.49 (s, 1H), 8.65 (s, 1H), 8.49 – 7.95 (m, 7H), 7.85 (s, 2H), 7.79 (s, 2H), 7.66 (d, *J* = 8.6 Hz, 2H), 7.60 (s, 1H), 7.38 (t, *J* = 7.3 Hz, 1H), 7.33 (dd, *J* = 17.4, 6.9 Hz, 1H), 7.20 (s, 1H), 6.92 (s, 1H), 6.75 (d, *J* = 15.4 Hz, 2H), 6.64 (d, *J* = 8.7 Hz, 1H), 6.36 – 6.25 (m, 1H), 5.97 (d, *J* = 9.6 Hz, 1H), 5.77 (d, *J* = 10.1 Hz, 1H), 5.74 (d, *J* = 6.8 Hz, 1H), 5.65 (s, 1H), 5.61 – 5.53 (m,2), 5.35 (ddd, *J* = 14.9, 4.6, 2.0 Hz, 1H), 5.24 (d, *J* = 9.7 Hz, 1H), 5.13 – 5.04 (m, 1H), 4.49 (s, 7H), 4.33 (s, 1H), 4.28 – 4.21 (m, 5H), 4.20 – 4.12 (m, 4H), 4.01 – 3.94 (m, 1H), 3.72 (s, 1H), 3.67 – 3.62 (m, 1H), 2.91 (s, 2H), 2.88 – 2.82 (m, 1H), 2.80 – 2.64 (m, 7H), 2.49 – 2.44 (m, 2H), 2.33 – 2.17 (m, 3H), 2.05 (s, 1H), 1.99 (d, *J* = 6.8 Hz, 2H), 1.94 – 1.84 (m, 2H), 1.74 (s, 3H), 1.73 – 1.69 (m, 2H), 1.67 (s, 3H), 1.63 (d, *J* = 6.2 Hz, 3H), 1.62 – 1.57 (m, 2H), 1.55 – 1.47 (m, 5H), 1.48 – 1.41 (m, 1H), 1.33 (d, *J* = 13.7 Hz, 3H), 0.87 (d, *J* = 6.4 Hz, 3H), 0.82 (s, 1H), 0.72 (d, *J* = 7.0 Hz, 3H). **^{13}C-NMR** (176 MHz, DMSO-d_6) δ [ppm]: 174.24, 174.08, 173.74, 173.20, 172.39, 171.91, 171.83, 171.76, 171.71, 170.98, 170.84, 170.58, 170.47, 170.35, 166.91, 166.35, 163.54, 160.89, 158.14, 157.96, 157.77, 157.59, 155.63, 153.69, 150.77, 148.68, 148.54, 146.72, 143.06, 138.52, 136.21, 135.81, 131.18, 129.36, 129.18, 129.02, 128.78, 128.19, 127.92, 127.39, 126.81, 125.81, 124.55, 122.28, 121.24, 120.33, 111.17, 77.84, 74.06, 73.54, 68.20, 62.82, 56.58, 54.91, 52.91, 52.10, 52.01, 51.60, 49.90, 49.77, 49.66, 49.47, 48.90, 47.22, 46.30, 45.89, 38.69, 36.26, 35.97,

35.72, 31.89, 30.27, 29.74, 29.57, 29.22, 29.00, 28.69, 26.57, 26.46, 25.85, 25.52, 22.07, 20.77, 20.10, 17.65, 16.42, 11.08. **LRMS** (ESI-Quad) [m/z]: 919.8 $[M+2H]^{2+}$, **HRMS** (ESI-IT) [m/z]: 918.89078, calculated 918.88920 for $C_{83}H_{111}N_{19}O_{29}$ $[M+2H]^{2+}$, err [ppm] 1.719.

Synthesis of FA-SS-16*R*-Aminoratjadone

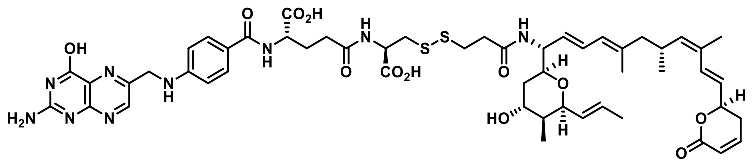

Chemical Formula: $C_{53}H_{67}N_9O_{12}S_2$
Molecular Weight: 1086,29
FA-SS-16*R*-Aminoratjadone

To a solution of 2.6 mg (4.72 µmol, 1.1 equiv) **FA-SH-1** in 1.22 mL ACN under Argon atm. was added a solution of 2.8 mg (4.28 µmol, 1.0 equiv) 2-PySS(CH2)2(CO)-16R-Aminoratjadone **31** in 1.22 mL PBS buffer (pH = 7.4) and the mixture was stirred for 4 h at 23°C. The ACN was removed by a nitrogen flow and 50 µL DMSO were added. The mixture was filtered through a Whatman filter (45 µm) and directly purified by RP prep HPLC (Phenomenex Gemini C18 RP-column 00G-4435-PO-AX, 5 µm, 110 A, 250×21.20 mm, Flow: 9 mL/min, ACN:H_2O + 0.1% TFA/ 10:90 → 95:5 in 45 min) yielding after lyophilization 1.5 mg (1.38 µmol, 32%) **FA-SS-16*R*-Aminoratjadone** as yellow, amorph solid.

FA-SS-16*R*-Aminoratjadone: ^{1}H-NMR (700 MHz, DMSO-*d*$_6$) δ [ppm]: 12.85 (s, 1H), 12.46 (s, 1H), 11.40 (s, 1H), 8.64 (s, 1H), 8.30 (t, *J* = 7.5 Hz, 1H), 8.19 (d, *J* = 7.3 Hz, 1H), 7.98 (d, *J* = 8.8 Hz, 1H), 7.66 (d, *J* = 8.6 Hz, 2H), 7.07 – 7.01 (m, 1H), 6.92 (s, 1H), 6.75 (d, *J* = 15.6 Hz, 1H), 6.64 (d, *J* = 8.7 Hz, 2H), 6.30 (dd, *J* = 15.1, 11.2 Hz, 1H), 5.97 (d, *J* = 9.7 Hz, 1H), 5.78 (d, *J* = 10.4 Hz, 1H), 5.74 (dd, *J* = 15.6, 6.6 Hz, 1H), 5.59 (td, *J* = 14.9, 6.6 Hz, 2H), 5.36 (dd, *J* = 15.4, 5.4 Hz, 1H), 5.23 (d, *J* = 9.6 Hz, 1H), 5.09 (dt, *J* = 10.4, 5.4 Hz, 1H), 4.74 (s, 3H), 4.47 (dd, *J* = 13.1, 4.2 Hz, 2H), 4.30 (p, *J* = 9.3, 8.9 Hz, 1H), 4.24 (s, 1H), 3.71 (s, 1H), 3.63 (dd, *J* = 11.8, 5.7 Hz, 1H), 3.52 (s, 2H), 3.13 – 3.05 (m, 1H), 2.93 – 2.84 (m, 2H), 2.71 (s, 1H), 2.32 – 2.20 (m, 2H), 2.09 – 2.01 (m, 2H), 1.98 (d, *J* = 6.9 Hz, 2H), 1.95 – 1.85 (m, 3H), 1.74 (s, 3H), 1.67 (s, 3H), 1.64 (d, *J* = 5.5 Hz, 3H), 1.52 – 1.41 (m, 4H), 1.34 (d, *J* = 11.9 Hz, 2H), 0.87 (d, *J* = 6.5 Hz, 3H), 0.82 (s, 1H), 0.74 (d, *J* = 7.1 Hz, 3H). **^{13}C-NMR** (176 MHz, DMSO-*d*$_6$) δ [ppm]: 173.74, 171.95, 169.26, 166.31, 163.54, 150.74, 146.72, 138.55, 136.08, 131.23, 129.41, 128.98, 128.88, 127.93, 127.14, 126.77, 125.84, 124.64, 124.52, 121.32, 120.32, 111.13, 77.87, 74.05, 73.66, 68.22, 54.03, 52.18, 51.28, 47.23, 45.91, 35.03, 33.96, 33.65, 31.92, 31.28, 31.20, 29.90, 29.82, 29.58, 29.22, 29.00, 28.89, 28.69, 26.57, 24.48, 22.09, 20.79, 20.09, 17.66, 16.45, 13.96, 11.08. **LRMS** (ESI-Quad) [m/z]: 1086.5 $[M+H]^+$, **HRMS** (ESI-IT) [m/z]: 1086.44132, calculated 1086.44233 for $C_{53}H_{68}N_9O_{12}S_2$ $[M+H]^+$, err [ppm] -0.929.

LHRH-Aminoratjadone Conjugates

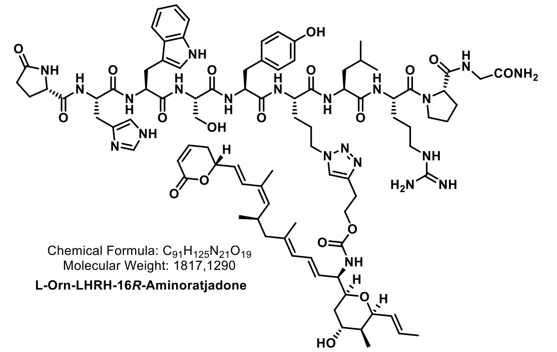

Chemical Formula: $C_{91}H_{125}N_{21}O_{19}$
Molecular Weight: 1817,1290
L-Orn-LHRH-16*R*-Aminoratjadone

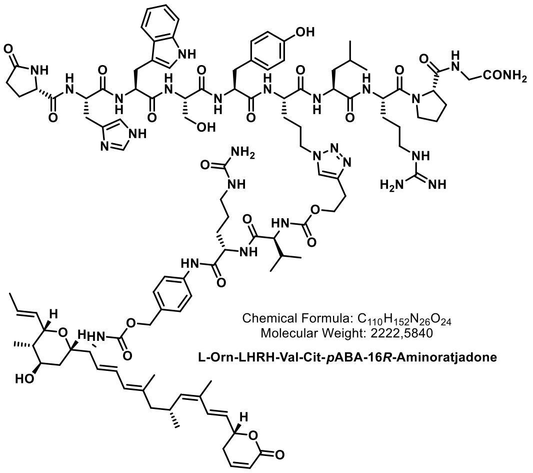

Chemical Formula: $C_{110}H_{152}N_{26}O_{24}$
Molecular Weight: 2222,5840
L-Orn-LHRH-Val-Cit-*p*ABA-16*R*-Aminoratjadone

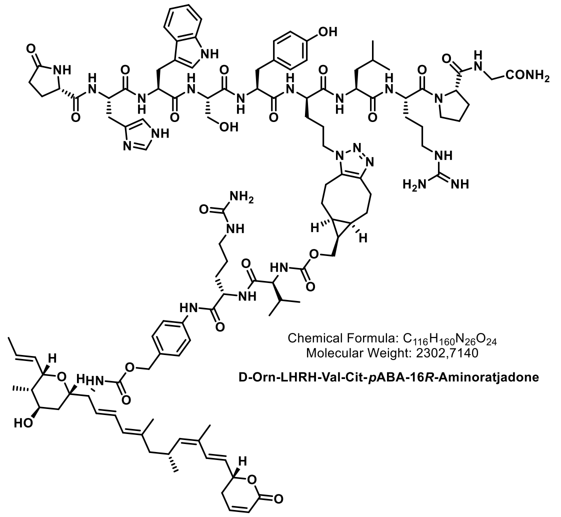

Chemical Formula: $C_{116}H_{160}N_{26}O_{24}$
Molecular Weight: 2302,7140
D-Orn-LHRH-Val-Cit-*p*ABA-16*R*-Aminoratjadone

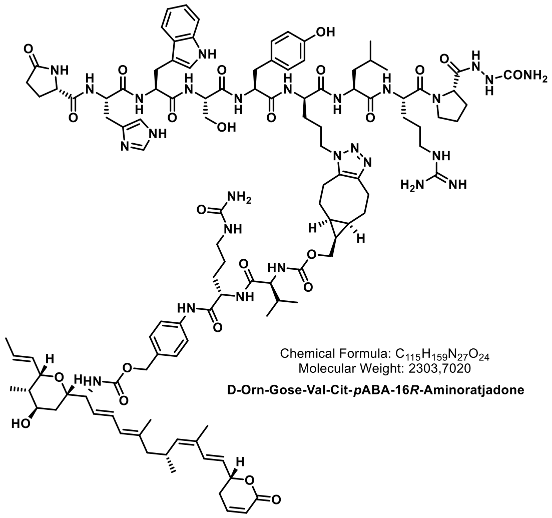

Chemical Formula: $C_{115}H_{159}N_{27}O_{24}$
Molecular Weight: 2303,7020
D-Orn-Gose-Val-Cit-*p*ABA-16*R*-Aminoratjadone

Figure S7: LHRH-Aminoratjadone conjugates synthesized in this study.

Synthesis of L-Orn-LHRH-16*R*-Aminoratjadone

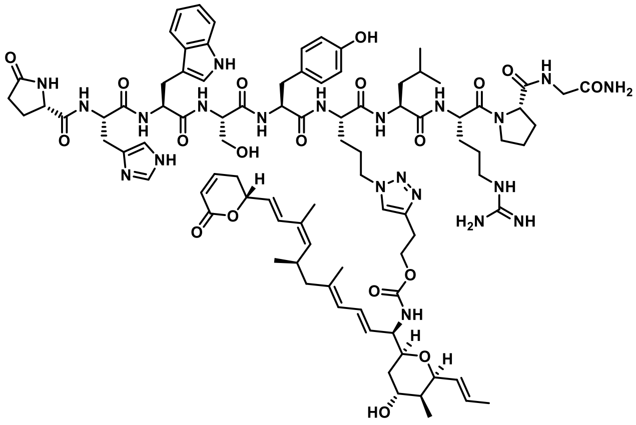

Chemical Formula: $C_{91}H_{125}N_{21}O_{19}$
Molecular Weight: 1817,13
L-Orn-LHRH-16*R*-Aminoratjadone

To a solution of 5.4 mg (3.63 µmol, 1.0 equiv) **L-Orn-N_3-LHRH** and 2.0 mg (3.63 µmol, 1.0 equiv) the 16R-Aminoratjadone derivative **21** in a mixture of DMSO:pH =7 phosphate buffer (100nM):tBuOH/2:2:1 (72 µL) were added 0.48 mg (0.9065µmol, 0.25 eq, 10 µL from a stock solution in DMSO) TBTA, 66 µg (0.363 µmol, 0.1 eq, 10 µL from a stock solution in H_2O) CuOAc, 2.39 mg (10.875 µmol, 3.0 equiv) zinc acetate and 718 µg (3.63 µmol, 1.0 eq, 10 µL from a stock solution in H_2O) sodium ascorbate and the mixture was stirred under light exclusion for 1 h at 23°C until complete conversion was monitored by LCMS. The reaction mixture was diluted with 100 µL of MeOH, filtered through a Whatman filter (45 µm) and directly purified by RP prep HPLC (Phenomenex Gemini C18 RP-column 00G-4435-PO-AX, 5 µm, 110 A, 250×21.20 mm, Flow: 9 mL/min, ACN:H_2O + 0.1% TFA/ 10:90 → 95:5 in 45 min) yielding after lyophilization 4.6 mg (2.53 µmol, 70%) **L-Orn-LHRH-16*R*-Aminoratjadone** TFA salt as a white, amorph solid.

L-Orn-LHRH-16*R*-Aminoratjadone: ^{1}H-NMR (700 MHz, DMSO-d_6) δ [ppm]: 13.98 (s, 2H), 10.78 (s, 1H), 9.18 (s, 1H), 8.91 (s, 1H), 8.32 – 8.26 (m, 1H), 8.23 (t, *J* = 5.8 Hz, 1H), 8.20 (d, *J* = 7.2 Hz, 1H), 8.11 (s, 2H), 8.07 (d, *J* = 8.1 Hz, 1H), 8.05 – 8.02 (m, 1H), 7.99 (d, *J* = 7.5 Hz, 1H), 7.84 (d, *J* = 6.6 Hz, 1H), 7.68 (s, 1H), 7.61 (d, *J* = 7.9 Hz, 1H), 7.41 – 7.37 (m, 1H), 7.36 (d, *J* = 7.6 Hz, 1H), 7.30 (q, *J* = 10.6, 9.5 Hz, 3H), 7.26 (s, 1H), 7.20 (d, *J* = 9.0 Hz, 2H), 7.14 (d, *J* = 5.9 Hz, 1H), 7.12 (d, *J* = 1.9 Hz, 1H), 7.08 (d, *J* = 4.0 Hz, 1H), 7.06 – 7.02 (m, 4H), 6.91 (t, *J* = 7.5 Hz, 1H), 6.75 (d, *J* = 15.6 Hz, 1H), 6.64 (d, *J* = 8.4 Hz, 3H), 6.31 (dd, *J* = 14.8, 10.6 Hz, 1H), 6.03 – 5.93 (m, 1H), 5.77 (d, *J* = 11.6 Hz, 1H), 5.76 – 5.70 (m, 1H), 5.58 (ddd, *J* = 20.1, 14.2, 6.9 Hz, 3H), 5.38 – 5.30 (m, 2H), 5.24 (d, *J* = 9.6 Hz, 1H), 5.09 (dt, *J* = 10.4, 5.2 Hz, 1H), 5.03 (s, 1H), 4.73 (s, 1H), 4.67 – 4.61 (m, 1H), 4.62 – 4.56 (m, 1H), 4.51 (q, *J* = 7.5 Hz, 1H), 4.46 – 4.41 (m, 1H), 4.37 – 4.32 (m, 2H), 4.32 – 4.20 (m, 6H), 4.18 – 4.13 (m, 2H), 4.01 – 3.94 (m, 2H), 3.70 (d, *J* = 15.5 Hz, 2H), 3.64 (s, 2H), 3.62 – 3.54 (m, 4H), 3.51 (s, 2H), 3.15 (d, *J* = 10.8 Hz, 1H), 3.10 – 3.05 (m, 2H), 3.02 (d, *J* = 14.1 Hz, 1H), 2.98 (dd, *J* = 14.8, 9.1

Hz, 1H), 2.92 – 2.83 (m, 2H), 2.18 – 2.12 (m, 1H), 2.06 – 2.02 (m, 2H), 1.99 (d, *J* = 8.0 Hz, 2H), 1.95 – 1.91 (m, 1H), 1.81 (dq, *J* = 13.4, 7.2, 6.7 Hz, 2H), 1.74 (s, 3H), 1.73 – 1.67 (m, 2H), 1.67 (s, 3H), 1.63 (d, *J* = 6.5 Hz, 3H), 1.61 – 1.57 (m, 2H), 1.54 – 1.39 (m, 6H), 1.37 – 1.29 (m, 4H), 0.87 (d, *J* = 6.6 Hz, 3H), 0.82 (d, *J* = 6.5 Hz, 3H), 0.76 (d, *J* = 6.4 Hz, 3H), 0.72 (d, *J* = 7.1 Hz, 3H). **^{13}C-NMR** (176 MHz, DMSO-d_6) δ [ppm]: 177.44, 174.22, 172.47, 171.88, 171.85, 171.55, 171.00, 170.77, 170.66, 169.98, 169.74, 163.53, 157.92, 157.75, 157.57, 157.40, 156.57, 155.82, 146.72, 142.90, 138.51, 136.21, 136.02, 133.80, 131.18, 130.15, 129.63, 129.38, 129.02, 128.74, 127.78, 127.30, 126.80, 125.79, 124.53, 123.69, 122.36, 120.85, 120.32, 118.56, 118.20, 116.95, 114.87, 111.21, 109.65, 77.85, 74.05, 73.53, 68.19, 62.79, 61.74, 59.71, 56.55, 55.41, 54.97, 54.48, 53.28, 51.67, 50.49, 50.13, 48.85, 47.21, 46.97, 41.95, 36.94, 35.10, 31.26, 29.70, 29.56, 29.22, 29.08, 28.81, 28.71, 28.68, 28.57, 28.55, 28.10, 27.74, 26.59, 26.55, 26.01, 25.53, 25.09, 24.99, 24.59, 24.52, 24.10, 23.18, 22.08, 21.10, 20.78, 20.09, 17.64, 16.41, 13.95, 11.08. **LRMS** (ESI-Quad) [m/z]: 909.6 $[M+H]^+$, **HRMS** (ESI-IT) [m/z]: 908.97884, calculated 908.98031 for $C_{23}H_{34}N_5O_6$ $[M+H]^+$, err [ppm] -1.617.

L-Orn-LHRH-Val-Cit-*p*ABA-16*R*-Aminoratjadone

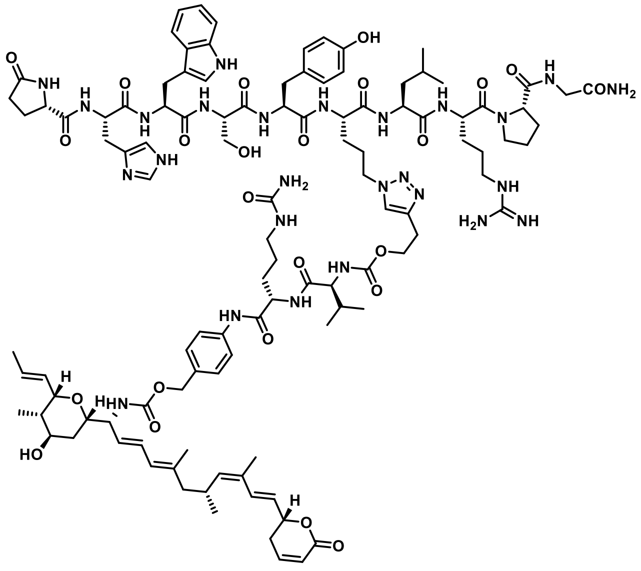

Chemical Formula: $C_{110}H_{152}N_{26}O_{24}$
Molecular Weight: 2222,58
L-Orn-LHRH-Val-Cit-*p*ABA-16*R*-Aminoratjadone

To a solution of 2.97 mg (1.99 µmol, 1.0 equiv) **L-Orn-N$_3$-LHRH** and 2.0 mg (2.09 µmol, 1.05 equiv) Homopropargyl-O(CO)HN-Val-Cit-PABO(CO)-16R-Aminoratjadone**26** in a mixture of DMSO:pH =7 phosphate buffer:tBuOH/2:2:1 (80 µL) were added 0.443 µg (0.835 µmol, 0.25 eq, 10 µL from a stock solution in DMSO) TBTA, 36 µg (0.199 µmol, 0.1 eq, 10 µL from a stock solution in H_2O) CuOAc, 1.31 mg (5.97 µmol, 3.0 equiv) zinc acetate and 394 µg (1.99 µmol, 1.0 eq, 10 µL from a stock solution in H_2O)

sodium ascorbate and the mixture was stirred under light exclusion for 2 h at 23°C until complete conversion was monitored by LCMS. The reaction mixture was diluted with 100 µL of MeOH, filtered through a Whatman filter (45 µm) and directly purified by RP prep HPLC (Phenomenex Gemini C18 RP-column 00G-4435-PO-AX, 5 µm, 110 A, 250×21.20 mm, Flow: 9 mL/min, ACN:H_2O + 0.1% TFA/ 10:90 → 95:5 in 45 min) yielding after lyophilization 2.2 mg (0.898 µmol, 45%) **L-Orn-LHRH-Val-Cit-*p*ABA-16*R*-Aminoratjadone** TFA salt as a white, amorph solid.

L-Orn-LHRH-Val-Cit-*p*ABA-16*R*-Aminoratjadone: **^{1}H-NMR** (700 MHz, DMSO-d_6) δ [ppm]: 13.98 (s, 2H), 10.79 (d, *J* = 2.3 Hz, 1H), 10.03 (s, 1H), 9.20 (s, 1H), 9.03 (s, 2H), 8.31 (s, 1H), 8.24 (t, *J* = 5.9 Hz, 1H), 8.21 (d, *J* = 7.2 Hz, 1H), 8.11 (d, *J* = 7.3 Hz, 3H), 8.07 (t, *J* = 7.4 Hz, 2H), 8.00 (d, *J* = 7.7 Hz, 1H), 7.87 (s, 1H), 7.69 (s, 1H), 7.61 (d, *J* = 8.0 Hz, 1H), 7.57 (d, *J* = 8.2 Hz, 2H), 7.47 (t, *J* = 5.7 Hz, 1H), 7.30 (dd, *J* = 8.5, 4.2 Hz, 2H), 7.27 (d, *J* = 8.2 Hz, 2H), 7.19 (s, 1H), 7.15 – 7.10 (m, 3H), 7.08 (s, 1H), 7.06 – 7.00 (m, 4H), 6.91 (t, *J* = 7.5 Hz, 1H), 6.75 (d, *J* = 15.6 Hz, 2H), 6.64 (d, *J* = 8.0 Hz, 1H), 6.35 – 6.26 (m, 1H), 6.02 – 5.97 (m, 1H), 5.97 (ddd, *J* = 9.7, 2.5, 1.2 Hz, 1H), 5.77 (d, *J* = 11.5 Hz, 2H), 5.75 – 5.72 (m, 2H), 5.57 (dt, *J* = 15.3, 7.9 Hz, 1H), 5.43 (s, 1H), 5.39 – 5.32 (m, 1H), 5.23 (d, *J* = 9.6 Hz, 1H), 5.09 (dt, *J* = 10.8, 5.3 Hz, 1H), 5.05 (s, 1H), 4.99 (d, *J* = 12.4 Hz, 1H), 4.93 (d, *J* = 12.5 Hz, 1H), 4.73 (s, 1H), 4.63 (dd, *J* = 12.8, 7.0 Hz, 1H), 4.60 – 4.54 (m, 2H), 4.53 – 4.47 (m, 2H), 4.46 – 4.38 (m, 5H), 4.34 (q, *J* = 6.1 Hz, 2H), 4.31 – 4.20 (m, 5H), 4.20 – 4.13 (m, 2H), 4.06 – 4.00 (m, 1H), 3.97 (dd, *J* = 8.8, 4.2 Hz, 1H), 3.92 (t, *J* = 7.9 Hz, 1H), 3.71 (d, *J* = 6.9 Hz, 2H), 3.68 – 3.54 (m, 5H), 3.51 (s, 3H), 3.05 – 2.83 (m, 8H), 2.73 (q, *J* = 8.4 Hz, 1H), 2.44 (ddt, *J* = 18.5, 10.6, 2.7 Hz, 2H), 2.19 – 1.92 (m, 9H), 1.80 (p, *J* = 6.8, 6.2 Hz, 2H), 1.74 (d, *J* = 1.3 Hz, 3H), 1.72 – 1.68 (m, 2H), 1.67 (s, 3H), 1.63 (dt, *J* = 6.5, 1.5 Hz, 3H), 1.60 (d, *J* = 8.0 Hz, 3H), 1.54 – 1.39 (m, 1H), 1.38 – 1.30 (m, 1H), 0.87 (d, *J* = 7.3 Hz, 3H), 0.86 (d, *J* = 7.2 Hz, 4H), 0.83 – 0.82 (m, 3H), 0.81 (d, *J* = 2.4 Hz, 3H), 0.76 (d, *J* = 6.4 Hz, 3H), 0.73 (d, *J* = 7.2 Hz, 3H). **^{13}C-NMR** (176 MHz, DMSO-d_6) δ [ppm]: 177.44, 172.44, 171.89, 171.85, 171.57, 171.21, 171.02, 170.78, 170.69, 170.54, 170.02, 169.77, 163.53, 158.90, 158.03, 157.85, 157.68, 157.51, 156.65, 156.04, 155.82, 155.69, 146.71, 142.94, 138.50, 136.18, 136.02, 132.05, 131.16, 130.14, 129.35, 129.03, 128.98, 128.32, 127.49, 127.30, 127.22, 126.80, 125.79, 124.54, 123.70, 122.47, 120.84, 120.33, 118.86, 118.53, 118.20, 116.49, 114.88, 111.21, 109.69, 77.83, 74.03, 73.55, 68.18, 64.91, 62.99, 61.70, 59.83, 59.74, 56.53, 55.41, 55.00, 54.52, 53.33, 53.04, 51.71, 50.51, 50.14, 48.86, 47.22, 46.98, 41.96, 40.75, 40.60, 38.65, 36.91, 30.47, 29.62, 29.58, 29.45, 29.22, 29.09, 29.05, 28.07, 27.69, 26.76, 26.02, 25.46, 25.01, 24.59, 24.53, 24.10, 23.17, 21.11, 21.05, 20.80, 20.10, 20.04, 19.17, 18.13, 17.65, 16.43, 11.08. **LRMS** (ESI-Quad) [m/z]: 1112.3 $[M+2H]^{2+}$, **HRMS** (ESI-IT) [m/z]: 112.0826, calculated 1112.0827 for $C_{110}H_{154}N_{26}O_{24}$ $[M+2H]^{2+}$, err [ppm] -0.089.

D-Orn-LHRH-Val-Cit-*p*ABA-16*R*-Aminoratjadone

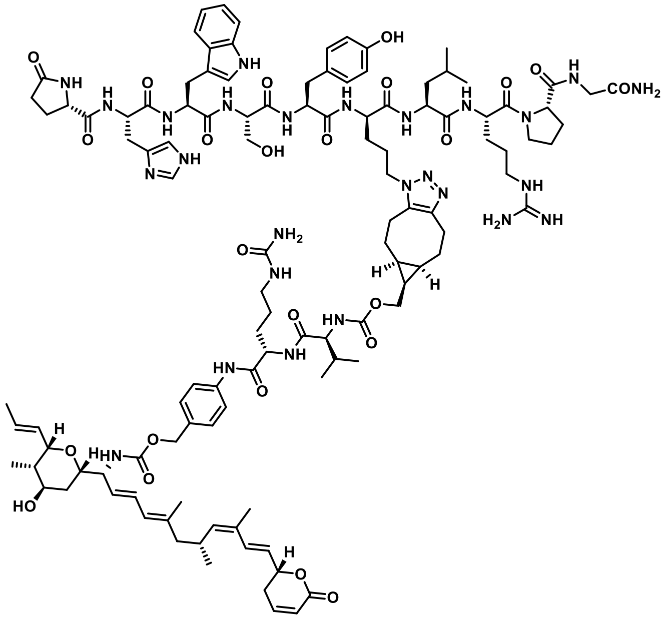

Chemical Formula: $C_{116}H_{160}N_{26}O_{24}$
Molecular Weight: 2302,71

D-Orn-LHRH-Val-Cit-*p*ABA-16*R*-Aminoratjadone

To a solution of 4 mg (2.678 µmol, 1.0 equiv) **D-Orn-N$_3$-LHRH** in 54 µL dry DMSO was added a solution of 3.0 mg (2.946 µmol, 1.1 equiv) BCN-O(CO)HN-Val-Cit-*p*ABO(CO)-16*R*-Aminoratjadone**25** in 54 µL dry DMSO and the mixture was stirred for 2 h at 23°C under light exclusion until complete conversion was monitored by LCMS. The reaction mixture was diluted with 100 µL of MeOH, filtered through a Whatman® filter (45 µm) and directly purified by RP prep HPLC (Phenomenex Gemini C18 RP-column 00G-4435-PO-AX, 5 µm, 110 A, 250×21.20 mm, Flow: 9 mL/min, ACN:H_2O + 0.1% TFA/ 10:90 → 95:5 in 45 min) yielding after lyophilization 3.7 mg (1.6 µmol, 60%) of **D-Orn-LHRH-Val-Cit-*p*ABA-16*R*-Aminoratjadone** as a mixture of diastereomers of TFA salts as a white, amorph solid.

D-Orn-LHRH-Val-Cit-*p*ABA-16*R*-Aminoratjadone: ^{1}H-NMR (700 MHz, DMSO-*d*$_6$) δ [ppm]: 14.05 (s, 2H), 10.90 – 10.35 (m, 1H), 10.03 (d, *J* = 6.1 Hz, 1H), 9.18 (s, 1H), 8.92 (s, 1H), 8.29 (d, *J* = 7.5 Hz, 1H), 8.25 – 8.18 (m, 1H), 8.13 (d, *J* = 8.4 Hz, 1H), 8.04 (td, *J* = 9.3, 5.1 Hz, 2H), 7.98 (d, *J* = 9.3 Hz, 3H), 7.68 (s, 1H), 7.61 (d, *J* = 8.0 Hz, 1H), 7.57 (dd, *J* = 8.8, 3.0 Hz, 2H), 7.46 (t, *J* = 5.7 Hz, 1H), 7.33 – 7.24 (m, 5H), 7.14 (s, 1H), 7.12 (d, *J* = 2.3 Hz, 1H), 7.10 (s, 1H), 7.08 (s, 1H), 7.06 – 7.01 (m, 4H), 6.91 (t, *J* = 7.5 Hz, 1H), 6.75 (d, *J* = 15.6 Hz, 1H), 6.64 (d, *J* = 8.1 Hz, 2H), 6.31 (dd, *J* = 15.3, 10.9 Hz, 1H), 5.99 (s, 1H), 5.97 (dd, *J* = 10.0, 2.5 Hz, 1H), 5.77 (d, *J* = 11.4 Hz, 1H), 5.76 – 5.72 (m, 1H), 5.57 (dt, *J* = 15.2, 7.7 Hz, 2H), 5.42 (s, 1H), 5.38 – 5.28 (m, 1H), 5.23 (d, *J* = 9.7 Hz, 1H), 5.09 (dq, *J* = 10.7, 5.3, 4.8 Hz, 1H), 5.04 (s, 1H), 4.99 (d, *J* = 12.5 Hz, 1H), 4.93 (d, *J* = 12.6 Hz, 1H), 4.64 (td, *J* = 8.1, 4.5 Hz, 1H), 4.60 (td, *J* = 8.3, 5.3 Hz, 1H), 4.50 (q, *J* = 7.5 Hz, 1H), 4.46 – 4.38 (m,

2H), 4.36 – 4.23 (m, 5H), 4.15 (s, 2H), 4.08 (d, *J* = 8.4 Hz, 1H), 4.03 (p, *J* = 7.4 Hz, 2H), 3.97 (dd, *J* = 8.7, 4.2 Hz, 1H), 3.90 (q, *J* = 6.9 Hz, 1H), 3.73 – 3.55 (m, 8H), 3.51 (dd, *J* = 10.5, 6.1 Hz, 4H), 3.16 – 3.12 (m, 1H), 3.12 – 3.01 (m, 4H), 3.00 – 2.81 (m, 7H), 2.76 – 2.68 (m, 2H), 2.66 – 2.59 (m, 1H), 2.53 (dd, *J* = 13.7, 5.0 Hz, 1H), 2.44 (ddt, *J* = 18.5, 10.5, 2.7 Hz, 2H), 2.17 – 2.00 (m, 6H), 1.99 (d, *J* = 7.2 Hz, 2H), 1.97 – 1.90 (m, 2H), 1.80 (dq, *J* = 16.6, 5.9 Hz, 2H), 1.74 (s, 3H), 1.70 (dq, *J* = 8.7, 4.2, 3.6 Hz, 2H), 1.67 (s, 3H), 1.63 (d, *J* = 6.4 Hz, 3H), 1.60 – 1.39 (m, 16H), 1.39 – 1.30 (m, 2H), 1.11 (q, *J* = 8.4 Hz, 1H), 0.87 (t, *J* = 5.7 Hz, 7H), 0.83 (d, *J* = 6.5 Hz, 6H), 0.77 (d, *J* = 6.4 Hz, 3H), 0.73 (d, *J* = 7.1 Hz, 3H). **^{13}C-NMR** (176 MHz, DMSO-d_6) δ [ppm]: 177.44, 172.48, 171.85, 171.57, 171.26, 171.01, 170.82, 170.71, 170.53, 170.01, 169.75, 169.58, 163.53, 158.89, 158.21, 158.03, 157.85, 157.67, 156.64, 156.35, 155.84, 155.69, 143.33, 143.26, 138.50, 138.46, 136.18, 136.03, 133.78, 132.66, 132.56, 132.06, 131.16, 130.12, 129.35, 129.12, 129.03, 128.98, 128.32, 127.52, 127.30, 127.22, 126.80, 125.79, 124.54, 123.70, 120.84, 120.33, 118.86, 118.56, 118.20, 117.78, 116.94, 116.08, 114.87, 111.21, 109.65, 77.83, 74.03, 73.55, 68.19, 64.90, 61.76, 61.67, 59.95, 59.91, 59.72, 56.53, 55.41, 54.99, 54.52, 53.27, 53.00, 51.83, 51.26, 50.50, 50.15, 48.59, 47.22, 46.97, 46.82, 41.96, 40.81, 40.61, 38.65, 36.92, 30.45, 30.40, 29.76, 29.72, 29.58, 29.50, 29.22, 29.09, 29.05, 28.07, 27.75, 27.08, 26.74, 25.72, 25.42, 25.35, 24.97, 24.59, 24.52, 24.11, 23.18, 22.25, 22.17, 21.82, 21.67, 21.22, 21.17, 21.05, 20.80, 20.10, 20.04, 19.31, 19.20, 19.05, 18.63, 18.43, 18.12, 17.65, 17.32, 17.20, 16.43, 11.08. **HRMS** (ESI-IT) [m/z]: 2302.2203, calculated 2302.2171 for $C_{116}H_{161}N_{26}O_{24}$ [M+H]$^+$, err [ppm] 1.389.

D-Orn-Gose-Val-Cit-*p*ABA-16*R*-Aminoratjadone

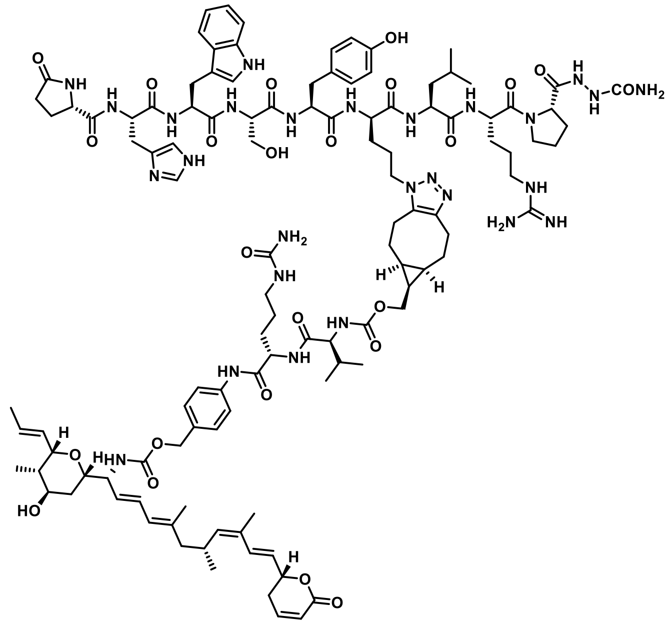

Chemical Formula: $C_{115}H_{159}N_{27}O_{24}$
Molecular Weight: 2303,70
D-Orn-Gose-Val-Cit-*p*ABA-16*R*-Aminoratjadone

To a solution of 4 mg (2.487 µmol, 1.0 equiv) **D-Orn-N$_3$-Gose** in 25 µL dry DMSO was added a solution of 2.84 mg (2.735 µmol, 1.1 equiv) BCN-O(CO)HN-Val-Cit-*p*ABO(CO)-16*R*-Aminoratjadone**25** in 25 µL dry DMSO and the mixture was stirred for 16 h at 23°C under light exclusion until complete conversion was monitored by LCMS. The reaction mixture was diluted with 100 µL of MeOH, filtered through a Whatman® filter (45 µm) and directly purified by RP prep HPLC (Phenomenex Gemini C18 RP-column 00G-4435-PO-AX, 5 µm, 110 A, 250×21.20 mm, Flow: 9 mL/min, ACN:H_2O + 0.1% TFA/ 10:90 → 95:5 in 45 min) yielding after lyophilization 3.9 mg (1.69 µmol, 68%) of **D-Orn-Goserellin-Val-Cit-*p*ABA-16*R*-Aminoratjadone** as a mixture of diastereomers of TFA salts as a white, amorph solid.

D-Orn-Gose-Val-Cit-*p*ABA-16*R*-Aminoratjadone: ^{1}H-NMR (700 MHz, DMSO-*d*$_6$) δ [ppm]: 14.03 (s, 2H), 10.78 (d, *J* = 2.5 Hz, 1H), 10.03 (d, *J* = 7.0 Hz, 1H), 9.78 (s, 1H), 9.18 (s, 1H), 8.92 (s, 1H), 8.29 (d, *J* = 7.6 Hz, 1H), 8.24 (d, *J* = 7.0 Hz, 1H), 8.13 (d, *J* = 8.7 Hz, 2H), 8.05 (p, *J* = 8.0 Hz, 2H), 7.98 (t, *J* = 9.1 Hz, 1H), 7.79 (s, 1H), 7.68 (s, 1H), 7.61 (d, *J* = 8.0 Hz, 1H), 7.59 – 7.55 (m, 2H), 7.44 (t, *J* = 5.7 Hz, 1H), 7.29 (dd, *J* = 23.2, 7.7 Hz, 5H), 7.14 – 7.07 (m, 2H), 7.06 – 7.00 (m, 4H), 6.90 (t, *J* = 7.5 Hz, 1H), 6.75 (d, *J* = 15.6 Hz, 1H), 6.64 (d, *J* = 8.2 Hz, 2H), 6.31 (dd, *J* = 15.2, 10.9 Hz, 1H), 6.03 – 5.94 (m, 2H), 5.90 (s, 2H), 5.77 (d, *J* = 11.6 Hz, 1H), 5.76 – 5.72 (m, 1H), 5.57 (dt, *J* = 15.1, 7.7 Hz, 2H), 5.49 – 5.38 (m, 2H), 5.38 – 5.30 (m, 1H), 5.23 (d, *J* = 9.7 Hz, 1H), 5.09 (dq, *J* = 10.8, 5.3, 4.7 Hz, 1H), 5.05 (d, *J* = 6.9 Hz, 0H), 4.99 (d, *J* = 12.2 Hz, 1H), 4.93 (d, *J* = 12.8 Hz, 1H), 4.64 (q, *J* = 7.8 Hz, 1H), 4.60 (td, *J* = 8.3, 5.3 Hz, 1H), 4.53 – 4.46 (m, 1H), 4.42 (q, *J* = 7.4, 6.8 Hz, 2H),

4.37 – 4.26 (m, 3H), 4.24 (d, *J* = 4.3 Hz, 1H), 4.17 (d, *J* = 20.7 Hz, 3H), 4.09 (d, *J* = 19.4 Hz, 1H), 4.05 – 3.99 (m, 2H), 3.97 (dd, *J* = 8.8, 4.2 Hz, 1H), 3.90 (q, *J* = 6.9 Hz, 1H), 3.77 – 3.70 (m, 3H), 3.66 (ddd, *J* = 11.9, 5.8, 2.5 Hz, 2H), 3.58 (s, 2H), 3.54 – 3.44 (m, 3H), 3.18 – 3.12 (m, 1H), 3.11 – 2.82 (m, 11H), 2.77 – 2.67 (m, 2H), 2.67 – 2.57 (m, 1H), 2.55 – 2.52 (m, 1H), 2.44 (ddt, *J* = 18.5, 10.6, 2.7 Hz, 1H), 2.20 – 2.01 (m, 6H), 1.99 (d, *J* = 7.1 Hz, 2H), 1.96 (d, *J* = 13.3 Hz, 2H), 1.80 (s, 2H), 1.74 (s, 3H), 1.70 (dt, *J* = 8.3, 4.4 Hz, 2H), 1.67 (s, 3H), 1.65 – 1.62 (m, 3H), 1.60 – 1.40 (m, 16H), 1.38 – 1.30 (m, 3H), 1.10 (p, *J* = 7.6, 6.9 Hz, 2H), 0.93 – 0.85 (m, 8H), 0.83 (d, *J* = 6.5 Hz, 6H), 0.77 (d, *J* = 6.3 Hz, 3H), 0.73 (d, *J* = 7.1 Hz, 3H). **^{13}C-NMR** (176 MHz, DMSO-d_6) δ [ppm]: 177.43, 172.47, 171.89, 171.55, 171.46, 171.25, 170.79, 170.68, 170.51, 170.07, 169.74, 169.56, 163.52, 158.88, 158.18, 158.00, 157.81, 157.63, 156.61, 156.33, 155.83, 155.69, 146.70, 143.32, 143.25, 138.49, 138.44, 136.17, 136.02, 135.30, 133.77, 132.55, 132.05, 131.15, 130.12, 129.34, 129.10, 129.02, 128.97, 128.31, 127.50, 127.30, 127.21, 126.79, 125.78, 124.53, 123.69, 120.84, 120.32, 118.85, 118.55, 118.19, 117.64, 116.93, 114.86, 111.20, 109.64, 77.82, 74.02, 73.54, 68.18, 64.89, 61.76, 61.65, 59.89, 58.33, 56.52, 55.41, 54.98, 54.51, 53.26, 52.99, 51.80, 51.25, 50.46, 50.12, 47.21, 46.99, 46.82, 40.81, 40.60, 38.65, 36.93, 33.65, 31.27, 31.19, 30.45, 30.40, 29.58, 29.49, 29.21, 29.08, 29.00, 28.92, 28.72, 28.68, 28.08, 27.75, 27.07, 26.74, 25.73, 25.71, 25.41, 25.35, 24.97, 24.69, 24.48, 24.11, 23.18, 22.25, 22.21, 22.18, 22.08, 21.82, 21.67, 21.17, 21.15, 21.04, 20.79, 20.09, 20.02, 19.31, 19.20, 19.05, 18.62, 18.13, 17.64, 17.33, 17.19, 16.42, 15.72, 13.95, 11.07. **HRMS** (ESI-IT) [m/z]: 768.4090, calculated 768.4090 for $C_{115}H_{162}N_{27}O_{24}$ $[M+3H]^{3+}$, err [ppm] 0.0.

Biological evaluation of the compounds

Cell proliferation assay

The corresponding cells were cultivated at 37 °C and 10 % CO_2 in the medium given in table 1. 60 µL of serial dilutions of the test compound were given to 120 µL of suspended cells (50.000/mL) in wells of 96-well plates. After 5 days of incubation growth inhibition (IC_{50}) was determined using an MTT assay.[9]

Table S1:Medium conditions for different cell types.

Cell type	Medium	Additives
L-929 (DSMZ ACC 2)	DME medium (high glucose) (Gibco)	10% fetal calf serum (Gibco)
SKOV-3 DSMZ ATCC HTB 77)	Mc Coys-Medium (Gibco)	10% fetal calf serum (Gibco)
MCF-7 (DSMZ ACC 115)	RPMI-Medium (Gibco)	10% fetal calf serum (Gibco), 1% MEM NEAA, 0,25% Human Insulin (Gibco)
A549 (DSMZ ACC 107)	DME medium (high glucose) (Gibco)	10% fetal calf serum (Gibco)
KB 3.1 (DSMZ ACC 158)	DME medium (high glucose) (Gibco)	10% fetal calf serum (Gibco)

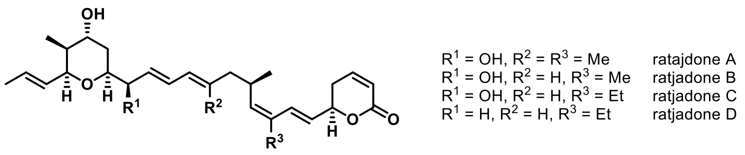

Ratjadone	Compound	Antiproliferative activity IC_{50} [nM]				
		KB-3.1	KB-V1	K-562	PC-3	L-929
A	**1**	0.30	0.15	0.30	0.08	0.15
B	**2**	1	0.15	0.50	0.15	0.35
C	**3**	0.35	0.08	0.15	0.08	0.2
D	**4**	2	0.30	0.50	0.40	1
KB-3.1: human cervix carcinoma, KB-V1: multi-drug-resistant human cervix carcinoma, K-562: human chronic myelogenous leukemia cell, PC-3: human prostate carcinoma, L-929: murine fibroblast						

Figure S8: Structures of Ratjadone A-D and their antiproliferative activity (IC50 values in nM). Data published by *Köster et al.*[10]

Table S2: Antiproliferative activities of novel Ratjadone derivatives.

**Compound**	**IC_{50} [nM]**				
	**KB-3.1**	**A-549**	**SK-OV-3**	**MCF-7**	**L-929**
Ratjadone A **1**	0.46	0.15	0.24	0.11	0.68
16-Oxoratjadone **13**	2.57	1.76	0.24	0.22	12.56
19-Oxoratjadone **14**	0.36	0.10	0.16	0.48	1.15
16,19-Dioxoratjadone **15**	3.98	9.72	1.59	2.87	24.30
16*R*-Aminoratjadone **16**	0.39	0.31	0.61	0.26	4.61
16*S*-Aminoratjadone **17**	1.59	1.31	0.81	0.36	28.38
Compound **18**	115.6				3307.38
Compound **19**	165.4				1322.95
Compound **20**	1.05	0.58	0.44	0.40	
Compound **21**	0.47	1.25	0.45	0.27	4.50
Compound **22**	1.23	2.45	1.20	0.62	
BCN-O(CO)HN-Val-Cit-*p*ABO(CO)-16*R*-	25.06	39.52	34.70	20.24	
Homopropargyl-O(CO)HN-Val-Cit-*p*ABO(CO)-16*R*-Aminoratjadone**26**	9.20	25.07	13.58	12.53	
2-PySS$(CH_2)_2$(CO)-16*R*-Aminoratjadone **31**	8.80	30.6	24.6	26.0	
HCC$(CH_2)_2$(CO)-NH-$(CH_2)_2$SS$(CH_2)_2$(CO)-16*R*-Aminoratjadone **29**	1.86	0.86	1.09	0.17	
19-Aminoratjadone **33**	6.67	4.39	1.40	2.10	
N-Propargyl-19-Aminoratjadone **35**	1.23	1.12	2.63	1.31	

Table S3: Antiproliferative activities of Carrier molecules.

Compound	IC_{50} [nM]			
	**KB-3.1**	**A-549**	**SK-OV-3**	**MCF-7**
**FA-N_3-5**	>8.64x10^4	>8.64x10^4		
**FA-N_3-6**	>1.18x10^5	>1.18x10^5		
**FA-N_3-9**	>1.40x10^5	>1.40x10^5		
**LHRH**	>4.05x10^5	>4.05x10^5	>4.05x10^5	>4.05x10^5
**L-Orn-N_3-LHRH**	>7.27x10^5	>7.27x10^5	>7.27x10^5	>7.27x10^5
**D-Orn-N_3-LHRH**	1106.3	318.4	189.7	47.4
**D-Orn-N_3-Gose**	>6.63x10^5	>6.63x10^5	>6.63x10^5	>6.63x10^5

Table S4: Antiproliferative activity of novel Gonadoliberin-Ratjadone Conjugates

Compound	IC_{50} [nM]
	**A-549**
**L-Orn-LHRH-16*R*-Aminoratjadone**	1600
**L-Orn-LHRH-Val-Cit-*p*ABA-16*R*-Aminoratjadone**	1200
**D-Orn-LHRH-Val-Cit-*p*ABA-16*R*-Aminoratjadone**	23.0
**D-Orn-Gose-Val-Cit-*p*ABA-16*R*-Aminoratjadone**	12.8

Cell proliferation assay under folate free conditions

KB 3.1 (DSMZ ACC 158) cells were cultivated at 37 °C and 10 % CO_2 in RMPI-medium without folic acid (Gibco) with 10% fetal calf serum (Gibco). 120 µL of suspended cells (100.000/mL) were seeded in in wells of 96-well plates and after 24 h at 37 °C and 10 % CO_2 the medium was removed, the cells were washed with PBS and RPMI-medium without folic acid (Gibco) + 10% dialyzed fetal calf serum (Sigma) were added to the cells, before after additional 24 h at 37°C and 10 % CO_2 60 µL of serial dilutions of the test compound were given to cells. After 1, 2 and 5 days of incubation growth inhibition (IC_{50}) was determined using an MTT assay.[9]

Table S5: Antiproliferative activity of novel Folate-Ratjadone Conjugates.

**Compound**	**IC_{50} [nM]**<br>**KB 3.1**
**FA-1-Val-Cit-*p*ABA-16*R*-Aminoratjadone**	168.9
**FA-2-Val-Cit-*p*ABA-16*R*-Aminoratjadone**	336.3
**FA-3-Val-Cit-*p*ABA-16*R*-16R-Aminoratjadone**	209.8
**FA-4-(Val-Cit-*p*ABA-16*R*-Aminoratjadone)$_2$**	147.6
**FA-5-Val-Cit-*p*ABA-16*R*-Aminoratjadone**	237.0
**FA-6-Val-Cit-*p*ABA-16*R*-Aminoratjadone**	223.3
**FA-7-Val-Cit-*p*ABA-16*R*-Aminoratjadone**	34.3
**FA-8-Val-Cit-*p*ABA-16*R*-Aminoratjadone**	39.9
**FA-9-Val-Cit-*p*ABA-16*R*-Aminoratjadone**	50.9
**FA-10-Val-Cit-*p*ABA-16*R*-Aminoratjadone**	35.3
**FA-11-(Val-Cit-*p*ABA-16*R*-Aminoratjadone)$_3$**	45.0
**FA-3-L-16*R*-Aminoratjadone**	29.8
**FA-3-L-19-Aminoratjadone**	48.3
**FA-3b-L-16*R*-Aminoratjadone**	94.0
**FA-5-L-16*R*-Aminoratjadone**	50.3
**FA-6-L-16*R*-Aminoratjadone**	150.5
**FA-7-L-16*R*-Aminoratjadone**	190.1
**FA-8-L-16*R*-Aminoratjadone**	129.1
**FA-9-L-16*R*-Aminoratjadone**	47.6
**FA-10-L-16*R*-Aminoratjadone**	707.7
**FA-SS-16*R*-Aminoratjadone**	294.6

Monitoring export inhibitory activity

Export inhibitory ability of compounds was evaluated with the translocation biosensor system. This cellular assay depends on a recombinantly expressed fusion protein consisting of a nuclear localization signal (SV40-NLS), glutathione S-transferase (GST), green fluorescent protein (GFP) and a nuclear export signal (HIV1-RevNES). Due to the two transport signals (NLS/NES), the biosensor is permanently shuttling between nucleus and cytoplasm but resides prominently in the cytoplasm due to a comparatively stronger NES. Export inhibiting compound induce a nuclear accumulation of the GFP-signal[11,12] Cell lines were maintained as recommended by the American Type Culture Collection in DMEM containing 5% glutamine (Thermo Fisher Scientific, Waltham, USA) supplemented with 10% FBS (Thermo Fisher Scientific, Waltham, USA). For quantifying the nuclear export inhibitory effect, HeLa cells stably expressing a fluorescent translocation biosensor ($HeLa_{RevBio}$)[13] were seeded into black 96well µclear plates (Greiner, Germany). They next day compounds were added covering an appropriate concentration range. After 1h of incubation, cells were fixed in 4% PFA for 10 min and permeabilized with 0.1% Triton X 100 in PBS for 5min. After washing with PBS nuclei were labeled with Hoechst H33342 (30min 10µg/ml). The intracellular distribution of the biosensor-dependent GFP signal was quantitatively evaluated with the high content imaging system ImageXpress MicroXLS (Molecular Devices, Sunnyvale, USA). By using the translocation enhanced application module (Molecular Devices, Sunnyvale, USA) the GFP-intensity was quantified in the cytoplasm and nuclear region. As final readout the difference of Mean Inner and Mean Outer Intensity was calculated. IC_{50}s of nuclear export inhibition were calculated using Sigma Plot with four parameter logistics curve regression (Systat Software GmbH, Erkrath, Germany). For confocal microscopy cells were seeded at a density of $0.2*10^5$ cells/well into 8 well microscopy chambers (Ibidi GmbH, Martinsried, Germany). The following day compounds were added at 125nM final concentration. After 1h the cells were imaged on an ECLIPSE Ti (Nikon) equipped with UltraVIEW VoX spinning disc (Perkin Elmer, Waltham, US), ORCA-R2 camera (Ham Hamamatsu Photonics, Japan) and Volocity software 6.1.1 (Perkin Elmer, Waltham, US).

Table S6: CRM1 Inhibitory activities of novel Ratjadone derivatives

**Compound**	**IC_{50} [nM]** **CRM1**
Ratjadone A **1**	1.2
16-Oxoratjadone **13**	52.5
19-Oxoratjadone **14**	13.3
16,19-Dioxoratjadone **15**	17.5
16*R*-Aminoratjadone **16**	2.7
16*R*-Aminoratjadone **17**	2.8
19-Aminoratjadone **33**	70.0
N-Propargyl-19-aminoratjadone **35**	18.3
Compound **20**	23.2
2-PySS(CH2)2(CO)-16*R*-Aminoratjadone **31**	44.1
Compound **21**	46.1
Compound **22**	12.0
BCN-O(CO)HN-Val-Cit-*p*ABO(CO)-16*R*-Aminoratjadone **25**	346
**L-Orn-LHRH-16*R*-Aminoratjadone**	1060
Biotin-PEG_3-16*S*-Aminoratjadone **36**	152

Analysis of cell labeling with fluorescein-labeled conjugates

All cell lines were maintained as recommended by the American Type Culture Collection in DMEM or RPMI containing 5% glutamine (Thermo Fisher Scientific, Waltham, USA) supplemented with 10% FBS (Thermo Fisher Scientific, Waltham, USA). For flow cytometric analysis cells were detached by trypsin and the density was adjusted to 2 x 10^6 cells/ml. The cell suspension was incubated with compounds at 1µM final concentration for 30min at 37°C, followed by two washing steps in PBS. For analysis of fluorescein intensity a LSRFortessa™ with FACSDiva™ software (BD Biosciences, Heidelberg, Germany) with the 488nm laser in combination with 525/50nm band pass filter was used. Data was evaluated using FlowJo software (FLOWJO LLC, Oregon, US).

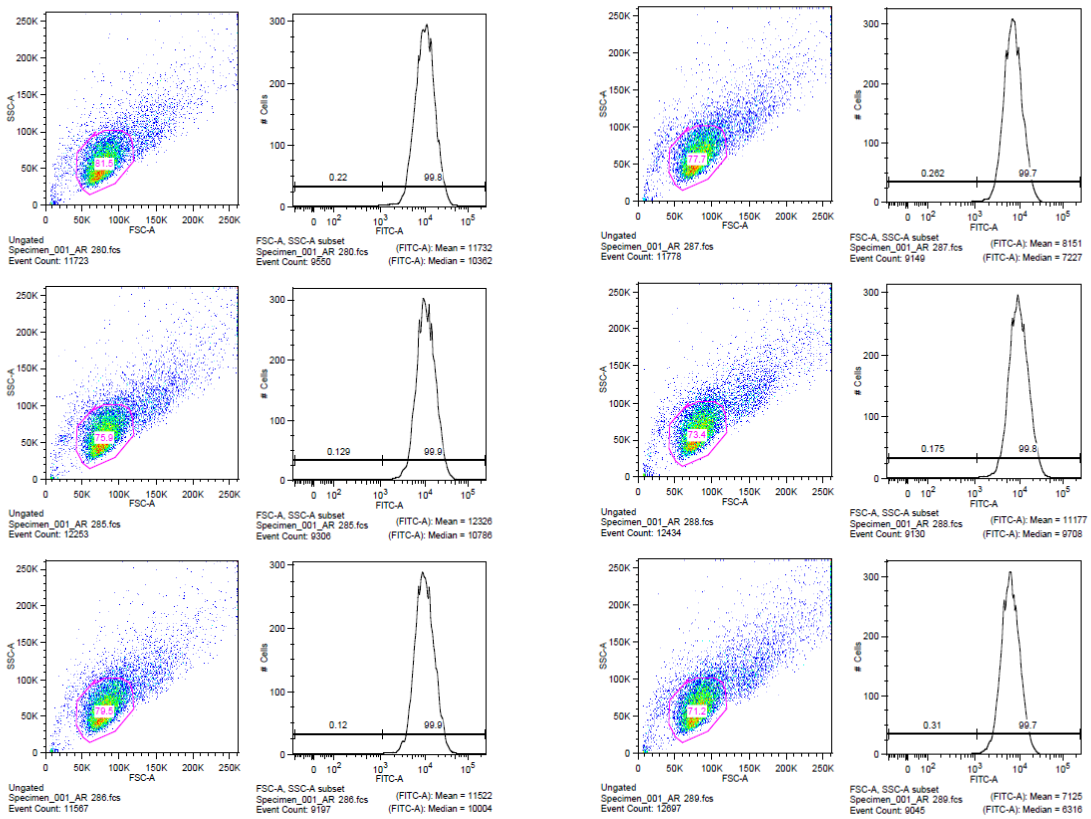

Figure S9: Left columns: Flow cytometry of KB 3.1 cells labeled with different Folate-Fluorescein Conjugates (AR280 = **FA-1a-FITC**, AR285 = **FA-10b-FITC**, AR286 = **FA-3a-FITC**, AR287 = **FA-6b-FITC**, AR288 = **FA-3b-FITC**, AR289 = **FA-4a-(FITC)$_2$**) shown in a dotplot, (SSC = side scatter, FSC = forward scatter), right columns: Fluoresence intensity per cell number.

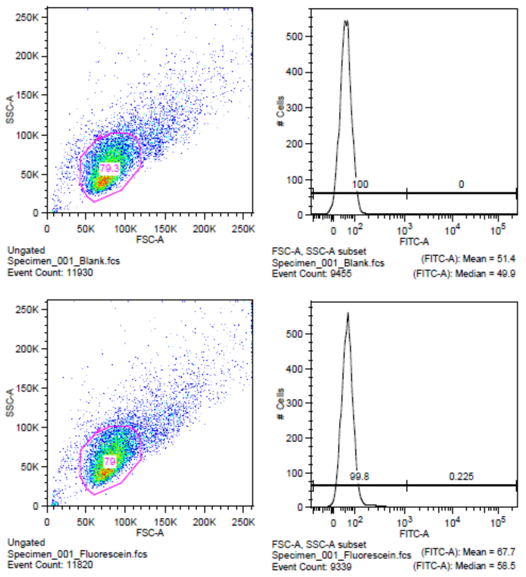

Figure S10: Left columns: Flow cytometry of KB 3.1 cells with Fluorescein as blank shown in a dotplot, (SSC = side scatter, FSC = forward scatter), right columns: Fluorescence intensity per cell number.

For confocal microscopy cells were seeded at a density of 0.2*10^5 cells/well into 8 well microscopy chambers (Ibidi GmbH, Martinsried, Germany). The following day compounds were added at 1µM final concentration. Cells were imaged after indicated timepoints on an ECLIPSE Ti (Nikon) equipped with UltraVIEW VoX spinning disc (Perkin Elmer, Waltham, US), ORCA-R2 camera (Ham Hamamatsu Photonics, Japan) and Volocity software 6.1.1 (Perkin Elmer, Waltham, US).

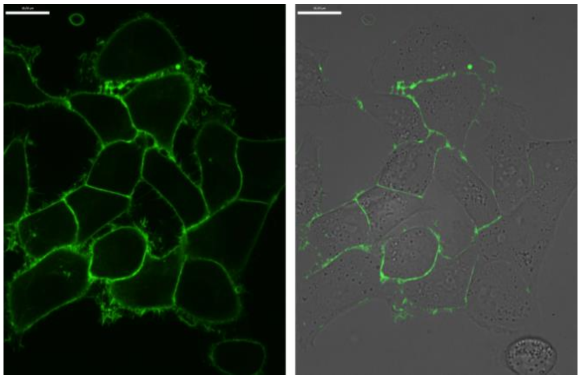

Figure S11: Cell imaging of KB 3.1 cells treated with 70 nM FA-1a-FITC (AR280) in in folate-free RPMI medium with 10% folate-containing FCS.

Streptavidin-Pull-down with Ratjadone-biotin and identification of exportin by LC-MSMS

A chemical pull down was performed to verify binding of ratjadone to Crm1. HeLa cells were treated with ratjadone-biotin, ratjadone or both at 0.1µg/ml final concentration for 5h. Cells were detached by scraping, washed twice in PBS and lysed in MPER-buffer (Thermo Fisher, Waltham, USA) supplemented with complete protease inhibitor (Roche, Mannheim, Germany). After 5min incubation on ice the lysate was centrifuged (20min, 13000rpm, 4°C) and the supernatant was incubated with streptavidin sepharose high performance (GE healthcare, Freiburg, Germany) overnight at 4°C. The next day beads were washed twice with PBS and resuspended in elution buffer (Roth) and heated to 95°C for 10min. 5% were tested on WesternBlot with a biotin-specific antibody.

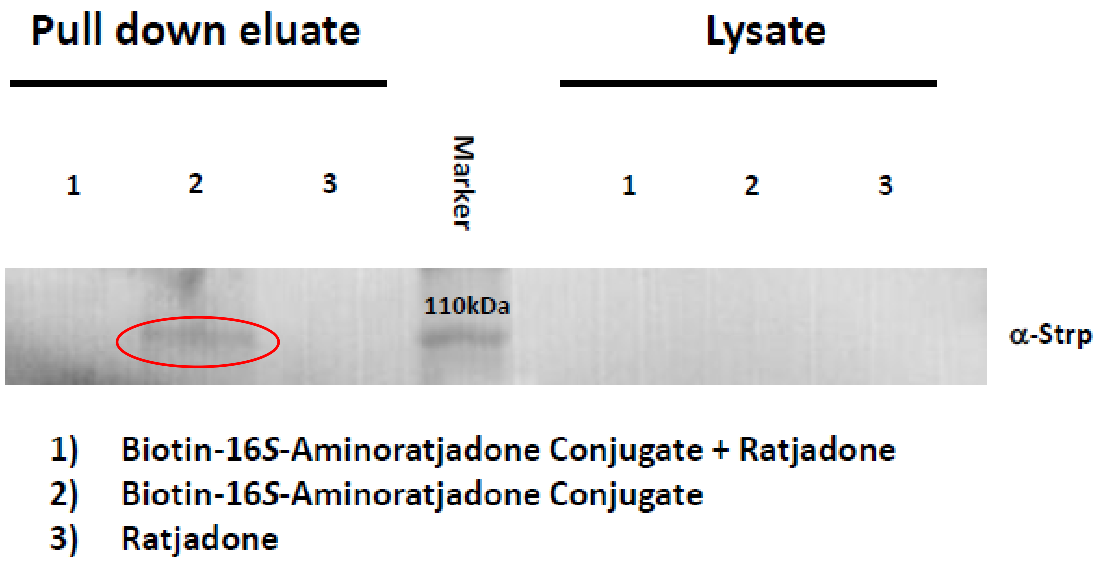

Figure S12: Gel of α-Streptavidin pulldown experiment: Identification of protein band in the eluat of **Biotin-PEG$_3$-16*S*-Aminoratjadone**.

Proteins from the remaining sample were extracted according to a procedure of *Wessel* and *Flügge*[14] and digested in 50 mM triethylammonium bicarbonate (TEAB) containing 10 % acetonitrile (ACN). The proteins were reduced in 10 mM TCEP (triscarboxyethylphosphine) for 30 min at 56°C and alkylated with 20 mM MMTS (methyl methanethiosulfonate). A protein/protease ratio of about 50:1 was applied and digestion was performed at 37°C overnight. Peptides were vacuum dried, resolved in 0.2 % trifluoroacetic acid (TFA) in water, desalted on self-packed Lichroprep RP18 (10 µL, Merck), eluted with 0.2 % TFA in 60 % ACN and dried again. Samples were resuspended in 0.2 % TFA (Trifluoro acetic acid) with 3% acetonitrile and injected.

LC-MS/MS analyses of and data interpretation–LC-MS/MS analyses of purified and desalted peptides were performed on a Dionex UltiMate 3000 n-RSLC system connected to an Orbitrap Fusion™ Tribrid™ mass spectrometer (Thermo Scientific). Peptides were loaded onto a C_{18} pre-column (3 µm RP18 beads, Acclaim, 75 µm x 20 mm), washed for 3 min at a flow rate of 6 µL/min and separated on a C_{18} analytical column (3-µm, Acclaim PepMap RSLC, 75 µm x 50 cm, Dionex) at a flow rate of 200 nl/min via a linear 30 min gradient from 97% buffer A (buffer A = 0.1% formic acid in water) to 25% B (buffer B = 0.1% formic acid in 80% acetonitrile), followed by a 15 min gradient from 25% buffer B to 62% buffer B. The LC system was operated with the Chromeleon software (version 6.8, Dionex) embedded in the Xcalibur software suite (version 3.0.63, Thermo Scientific, Dreieich, Germany). The effluent was electro-sprayed by a stainless steel emitter (Thermo). The mass spectrometer was controlled and operated in the data-dependent mode, using the Xcalibur software allowing the automatic selection of 2-4 fold charged peptides and their subsequent fragmentation using CID for fragmentation and the ion trap (IT) for detection of ions. (top speed mode). Every 3 seconds a new MS survey scan was performed. The maximum collection time for peptides was set to 100 ms. Dynamic exclusion was set to 6 sec.

MS/MS raw data files were processed via the Proteome Discoverer program Version 2.1 (Thermo Scientific, Dreieich, Germany) on a Mascot server (V. 2.4, Matrix Science) using UniProtKB database (release 2018_01, taxonomy: *Homo sapiens*).

The following search parameters were used: enzyme, trypsin; maximum missed cleavages, 1; fixed modification: Methylthio (C); variable modification: oxidation (M); peptide tolerance, 7 ppm; MS/MS tolerance, 0,4 Da. The Proteome Discoverer result file, containing all masterproteins with 3 or more unique peptides were exported to Excel after removal of keratin contaminants.

		Abundancies										
Accession	Description	Bio Ratj + Ratj	Bio Ratj	Ratj	No. of Unique Peptides	Sum PEP Score	Coverage [%]	No. of Peptides	# PSMs	Score Mascot	Gene Symbol	Modifications
P60709	Actin, cytoplasmic 1 OS=Homo sapiens GN=ACTB PE=1 SV=1	7,8E+08	1,2E+09	1,1E+09	9	203,262	58	24	218	4403	ACTB	Methylthio [C285]
P11498	Pyruvate carboxylase, mitochondrial OS=Homo sapiens GN=PC PE=1 SV=2	7,5E+08	1,1E+09	1,1E+09	60	351,274	54	60	353	8064	PC	Methylthio [C56; C131; C739]
**O14980**	**Exportin-1 OS=Homo sapiens GN=XPO1 PE=1 SV=1**	**6,0E+08**	**4,0E+06**	**4,2E+06**	**31**	**257,404**	**33**	**31**	**117**	**2918**	**XPO1**	**Methylthio [C164; C1070]**
Q13085	Acetyl-CoA carboxylase 1 OS=Homo sapiens GN=ACACA PE=1 SV=2	2,6E+08	3,9E+08	3,5E+08	70	389,837	34	76	321	5992	ACACA	Methylthio [C500; C813; C1297; C1396; C1769]
P15924	Desmoplakin OS=Homo sapiens GN=DSP PE=1 SV=3	2,3E+08	5,2E+08	5,1E+07	82	255,068	27	82	173	2579	DSP	Methylthio [C57; C682; C1069; C1805]
P11142	Heat shock cognate 71 kDa protein OS=Homo sapiens GN=HSPA8 PE=1 SV=1	1,9E+08	5,3E+08	4,6E+07	13	64,318	28	17	34	835	HSPA8	
P81605	Dermcidin OS=Homo sapiens GN=DCD PE=1 SV=2	8,4E+07	8,3E+07	4,1E+07	5	30,083	46	5	32	772	DCD	
P68371	Tubulin beta-4B chain OS=Homo sapiens GN=TUBB4B PE=1 SV=1	8,2E+07	8,7E+07	1,0E+08	4	152,447	66	24	102	2417	TUBB4B	Methylthio [C12; C239; C303]
Q86YZ3	Hornerin OS=Homo sapiens GN=HRNR PE=1 SV=2	8,1E+07	8,4E+07	2,8E+07	23	179,492	27	23	87	2506	HRNR	
F5H5D3	Tubulin alpha chain OS=Homo sapiens GN=TUBA1C PE=1 SV=1	6,5E+07	9,2E+07	1,1E+08	7	92,358	39	18	88	1951	TUBA1C	Methylthio [C365; C417]
P68871	Hemoglobin subunit beta OS=Homo sapiens GN=HBB PE=1 SV=2	6,4E+07	2,0E+07	2,8E+07	7	28,866	48	7	20	515	HBB	
P10809	60 kDa heat shock protein, mitochondrial OS=Homo sapiens GN=HSPD1 PE=1 SV=2	5,9E+07	5,7E+07	5,6E+07	26	102,939	48	26	72	1751	HSPD1	Methylthio [C447]
P29401	Transketolase OS=Homo sapiens GN=TKT PE=1 SV=3	5,7E+07	1,0E+08	9,7E+07	20	81,162	31	20	67	1285	TKT	
P14618	Pyruvate kinase PKM OS=Homo sapiens GN=PKM PE=1 SV=4	5,6E+07	7,5E+07	6,7E+07	24	115,573	49	24	86	1555	PKM	Methylthio [C49]
Q96RQ3	Methylcrotonoyl-CoA carboxylase subunit alpha, mitochondrial OS=Homo sapiens GN=MCCC1 PE=	4,5E+07	6,7E+07	7,0E+07	22	129,969	42	22	71	1369	MCCC1	Methylthio [C190; C332]
P69905	Hemoglobin subunit alpha OS=Homo sapiens GN=HBA1 PE=1 SV=2	4,4E+07	2,8E+07	4,0E+07	4	24,953	36	4	20	347	HBA2; HBA1	
P35658	Nuclear pore complex protein Nup214 OS=Homo sapiens GN=NUP214 PE=1 SV=2	4,2E+07	8,5E+07	1,6E+07	8	23,839	7	8	12	218	NUP214	Methylthio [C1003]
Q06830	Peroxiredoxin-1 OS=Homo sapiens GN=PRDX1 PE=1 SV=1	4,2E+07	6,3E+07	4,0E+07	8	33,032	49	9	34	681	PRDX1	Methylthio [C173]
P07355	Annexin A2 OS=Homo sapiens GN=ANXA2 PE=1 SV=2	3,6E+07	5,0E+07	4,7E+07	20	88,144	55	20	62	1412	ANXA2	Methylthio [C133]
E7EX59	Propionyl-CoA carboxylase beta chain, mitochondrial OS=Homo sapiens GN=PCCB PE=1 SV=1	3,6E+07	4,8E+07	4,2E+07	16	84,476	37	16	58	1242		Methylthio [C300; C322]
P08670	Vimentin OS=Homo sapiens GN=VIM PE=1 SV=4	3,6E+07	8,8E+07	9,5E+07	28	131,675	63	31	100	1672	VIM	
P05165	Propionyl-CoA carboxylase alpha chain, mitochondrial OS=Homo sapiens GN=PCCA PE=1 SV=4	3,5E+07	5,0E+07	4,7E+07	21	100,662	28	21	72	1494	PCCA	
P04792	Heat shock protein beta-1 OS=Homo sapiens GN=HSPB1 PE=1 SV=2	3,3E+07	4,4E+07	3,0E+07	10	36,107	53	10	36	787	HSPB1	
P08238	Heat shock protein HSP 90-beta OS=Homo sapiens GN=HSP90AB1 PE=1 SV=4	3,0E+07	5,6E+07	5,0E+07	14	107,368	31	20	64	1423	HSP90AB1	
P02647	Apolipoprotein A-I OS=Homo sapiens GN=APOA1 PE=1 SV=1	2,8E+07	7,1E+05	3,0E+06	10	34,342	39	10	16	269	APOA1	
P07437	Tubulin beta chain OS=Homo sapiens GN=TUBB PE=1 SV=2	2,5E+07	3,1E+07	3,4E+07	3	149,03	56	23	104	2645	TUBB	Methylthio [C12; C239; C303]
J3KPF3	4F2 cell-surface antigen heavy chain OS=Homo sapiens GN=SLC3A2 PE=1 SV=1	2,4E+07	1,9E+07	2,3E+07	8	37,846	16	8	28	732	SLC3A2	
Q9HCC0	Methylcrotonoyl-CoA carboxylase beta chain, mitochondrial OS=Homo sapiens GN=MCCC2 PE=1 S	2,1E+07	4,2E+07	3,9E+07	14	76,879	25	14	45	1257	MCCC2	Methylthio [C267]
Q02413	Desmoglein-1 OS=Homo sapiens GN=DSG1 PE=1 SV=2	2,0E+07	2,6E+07	5,9E+06	12	50,609	16	12	33	612	DSG1	
P68104	Elongation factor 1-alpha 1 OS=Homo sapiens GN=EEF1A1 PE=1 SV=1	1,7E+07	2,1E+07	1,5E+07	6	20,046	16	6	17	298	EEF1A1	
P68431	Histone H3.1 OS=Homo sapiens GN=HIST1H3A PE=1 SV=2	1,5E+07	1,8E+07	2,0E+07	3	5,636	15	3	4	84	HIST1H3F; HIST1H3C	
Q9NZT1	Calmodulin-like protein 5 OS=Homo sapiens GN=CALML5 PE=1 SV=2	1,5E+07	1,4E+07	1,0E+07	8	27,382	59	8	21	390	CALML5	
P68032	Actin, alpha cardiac muscle 1 OS=Homo sapiens GN=ACTC1 PE=1 SV=1	1,4E+07	3,1E+07	1,6E+07	4	127,465	42	18	167	3215	ACTC1	
P25705	ATP synthase subunit alpha, mitochondrial OS=Homo sapiens GN=ATP5A1 PE=1 SV=1	1,4E+07	1,3E+07	2,6E+07	16	49,683	32	16	33	602	ATP5A1	
P06702	Protein S100-A9 OS=Homo sapiens GN=S100A9 PE=1 SV=1	1,2E+07	2,5E+07	5,0E+06	4	19,794	35	4	17	282	S100A9	
P47929	Galectin-7 OS=Homo sapiens GN=LGALS7 PE=1 SV=2	1,2E+07	1,9E+06	2,7E+05	4	22,899	39	4	9	346	LGALS7; LGALS7B	
P62937	Peptidyl-prolyl cis-trans isomerase A OS=Homo sapiens GN=PPIA PE=1 SV=2	1,1E+07	2,7E+07	1,4E+07	7	35,532	39	7	25	485	PPIA	Methylthio [C62; C161]
P22528	Cornifin-B OS=Homo sapiens GN=SPRR1B PE=1 SV=2	9,9E+06	7,6E+07	2,4E+06	4	7,34	52	4	8	74	SPRR1B	Methylthio [C25; C33; C41; C57; C109; C109; C109; C109; C109]
P20930	Filaggrin OS=Homo sapiens GN=FLG PE=1 SV=3	8,7E+06	1,6E+07	5,8E+06	7	54,6	9	7	28	627	FLG	
Q6UWP8	Suprabasin OS=Homo sapiens GN=SBSN PE=1 SV=2	8,5E+06	9,4E+06	4,7E+06	4	32,893	20	4	19	564	SBSN	
A0A024R6I7	Alpha-1-antitrypsin OS=Homo sapiens GN=SERPINA1 PE=1 SV=1	8,1E+06		7,6E+05	5	9,123	11	5	6	68	SERPINA1	
Q5D862	Filaggrin-2 OS=Homo sapiens GN=FLG2 PE=1 SV=1	7,6E+06	3,1E+07	3,9E+06	15	72,843	16	15	27	589	FLG2	Methylthio [C267]
P62805	Histone H4 OS=Homo sapiens GN=HIST1H4A PE=1 SV=2	7,3E+06	4,4E+06	1,1E+07	5	16,423	40	5	14	223	HIST1H4A; HIST1H4F	
P05109	Protein S100-A8 OS=Homo sapiens GN=S100A8 PE=1 SV=1	7,1E+06	6,4E+06	6,9E+05	5	18,687	45	5	11	106	S100A8	Methylthio [C42]
P00338	L-lactate dehydrogenase A chain OS=Homo sapiens GN=LDHA PE=1 SV=2	6,5E+06	6,7E+06	4,2E+06	4	19,76	17	5	16	393	LDHA	Methylthio [C163]
P06576	ATP synthase subunit beta, mitochondrial OS=Homo sapiens GN=ATP5B PE=1 SV=3	6,5E+06	8,1E+06	8,1E+06	8	45,602	25	8	27	414	ATP5B	
G8JLG2	CDSN OS=Homo sapiens GN=CDSN PE=1 SV=1	6,5E+06	9,6E+06	1,4E+06	6	16,637	14	6	14	174	CDSN	Methylthio [C391; C448]
Q08554	Desmocollin-1 OS=Homo sapiens GN=DSC1 PE=1 SV=2	6,4E+06	9,3E+06	1,9E+06	6	34,556	10	6	18	304	DSC1	Methylthio [C196; C454]
U3KQK0	Histone H2B OS=Homo sapiens GN=HIST1H2BN PE=1 SV=1	6,0E+06	2,8E+06	3,6E+06	3	17,496	21	3	12	456	HIST1H2BN	
P06733	Alpha-enolase OS=Homo sapiens GN=ENO1 PE=1 SV=2	5,7E+06	3,6E+07	6,1E+06	8	25,926	20	8	12	260	ENO1	
O43175	D-3-phosphoglycerate dehydrogenase OS=Homo sapiens GN=PHGDH PE=1 SV=4	5,7E+06	5,5E+06	6,1E+06	6	20,268	13	6	9	251	PHGDH	Methylthio [C281]
P62979	Ubiquitin-40S ribosomal protein S27a OS=Homo sapiens GN=RPS27A PE=1 SV=2	5,2E+06	5,6E+06	5,2E+06	5	15,065	34	5	19	292	RPS27A	
P38646	Stress-70 protein, mitochondrial OS=Homo sapiens GN=HSPA9 PE=1 SV=2	5,0E+06	5,5E+06	4,8E+06	8	29,369	15	8	17	526	HSPA9	
P14923	Junction plakoglobin OS=Homo sapiens GN=JUP PE=1 SV=3	5,0E+06	1,2E+07	8,6E+05	6	22,153	10	6	10	224	JUP	Methylthio [C457]
P04406	Glyceraldehyde-3-phosphate dehydrogenase OS=Homo sapiens GN=GAPDH PE=1 SV=3	4,9E+06	1,6E+07	1,9E+06	5	15,444	22	5	6	168	GAPDH	Methylthio [C247]
P21333	Filamin-A OS=Homo sapiens GN=FLNA PE=1 SV=4	4,9E+06	1,2E+07	1,1E+07	17	65,828	10	17	37	576	FLNA	
P62633	Cellular nucleic acid-binding protein OS=Homo sapiens GN=CNBP PE=1 SV=1	4,4E+06	6,8E+06	4,3E+06	5	10,979	27	5	10	228	CNBP	Methylthio [C54; C57; C158; C161]
Q99714	3-hydroxyacyl-CoA dehydrogenase type-2 OS=Homo sapiens GN=HSD17B10 PE=1 SV=3	3,9E+06	3,6E+06	4,0E+06	7	31,758	45	7	18	530	HSD17B10	
P01834	Immunoglobulin kappa constant OS=Homo sapiens GN=IGKC PE=1 SV=2	3,7E+06	8,1E+05	7,7E+05	6	23,201	81	6	10	124	IGKC	Methylthio [C27; C87]
P11021	78 kDa glucose-regulated protein OS=Homo sapiens GN=HSPA5 PE=1 SV=2	3,7E+06	6,2E+06	4,0E+06	12	50,161	27	14	23	334	HSPA5	
P04083	Annexin A1 OS=Homo sapiens GN=ANXA1 PE=1 SV=2	3,6E+06	2,7E+06	5,7E+05	7	28,744	22	7	15	434	ANXA1	
P35579	Myosin-9 OS=Homo sapiens GN=MYH9 PE=1 SV=4	3,4E+06	7,6E+06	1,1E+07	21	70,03	14	21	30	461	MYH9	Methylthio [C917]
Q9BUF5	Tubulin beta-6 chain OS=Homo sapiens GN=TUBB6 PE=1 SV=1	3,4E+06	2,9E+06	5,5E+06	5	55,218	27	12	43	633	TUBB6	Methylthio [C12; C303]
Q9NQC3	Reticulon-4 OS=Homo sapiens GN=RTN4 PE=1 SV=2	3,3E+06	6,3E+05	3,1E+06	3	8,672	3	3	3	64	RTN4	
P10599	Thioredoxin OS=Homo sapiens GN=TXN PE=1 SV=3	3,2E+06	7,6E+06	2,6E+06	3	14,273	30	3	15	169	TXN	
P06748	Nucleophosmin OS=Homo sapiens GN=NPM1 PE=1 SV=2	2,8E+06	3,8E+06	3,5E+06	6	30,584	28	6	15	355	NPM1	
P31947	14-3-3 protein sigma OS=Homo sapiens GN=SFN PE=1 SV=1	2,4E+06	4,0E+05	1,2E+05	3	8,61	16	3	5	95	SFN	Methylthio [C38]
A0A0G2JIW1	Heat shock 70 kDa protein 1B OS=Homo sapiens GN=HSPA1B PE=1 SV=1	2,3E+06	4,5E+06	2,1E+06	7	39,794	19	10	20	492		
P32119	Peroxiredoxin-2 OS=Homo sapiens GN=PRDX2 PE=1 SV=5	2,3E+06	3,3E+06	2,2E+06	4	14,887	27	5	12	231	PRDX2	
P01891	HLA class I histocompatibility antigen, A-68 alpha chain OS=Homo sapiens GN=HLA-A PE=1 SV=4	2,1E+06	2,3E+06	2,8E+06	3	11,947	21	5	6	112	HLA-A	
P80723	Brain acid soluble protein 1 OS=Homo sapiens GN=BASP1 PE=1 SV=2	2,1E+06	7,9E+06	3,8E+06	6	24,901	54	6	8	212	BASP1	
P35232	Prohibitin OS=Homo sapiens GN=PHB PE=1 SV=1	2,0E+06	2,1E+06	4,4E+05	5	12,712	21	5	8	107	PHB	
P13639	Elongation factor 2 OS=Homo sapiens GN=EEF2 PE=1 SV=4	1,9E+06	8,5E+05	3,2E+06	4	12,519	6	4	8	131	EEF2	
B7Z6Z4	Myosin light polypeptide 6 OS=Homo sapiens GN=MYL6 PE=1 SV=1	1,4E+06	2,5E+06	2,5E+06	4	13,028	20	4	11	154	MYL6	
P40227	T-complex protein 1 subunit zeta OS=Homo sapiens GN=CCT6A PE=1 SV=3	1,3E+06	1,9E+06	9,1E+05	3	7,179	6	3	6	69	CCT6A	
P14174	Macrophage migration inhibitory factor OS=Homo sapiens GN=MIF PE=1 SV=4	1,1E+06	8,8E+05		3	10,482	21	3	4	111	MIF	Methylthio [C81]
Q14697	Neutral alpha-glucosidase AB OS=Homo sapiens GN=GANAB PE=1 SV=3	1,0E+06	1,3E+06	4,8E+05	6	19,847	8	6	9	154	GANAB	
P31327	Carbamoyl-phosphate synthase [ammonia], mitochondrial OS=Homo sapiens GN=CPS1 PE=1 SV=2	8,6E+05	4,1E+06	2,0E+06	7	22,65	7	7	14	209	CPS1	Methylthio [C816]
P27824	Calnexin OS=Homo sapiens GN=CANX PE=1 SV=2	8,0E+05	6,4E+05	1,1E+06	4	17,45	10	4	10	164	CANX	
P68366	Tubulin alpha-4A chain OS=Homo sapiens GN=TUBA4A PE=1 SV=1	7,7E+05	9,1E+05	9,1E+05	5	90,499	43	16	81	1613	TUBA4A	Methylthio [C54; C295; C347]
Q15149	Plectin OS=Homo sapiens GN=PLEC PE=1 SV=3	7,5E+05	2,3E+06	2,9E+06	10	31,56	3	13	21	184	PLEC	
P46109	Crk-like protein OS=Homo sapiens GN=CRKL PE=1 SV=1	7,3E+05	1,7E+06	1,5E+06	3	8,659	14	3	8	106	CRKL	
P05186	Alkaline phosphatase, tissue-nonspecific isozyme OS=Homo sapiens GN=ALPL PE=1 SV=4	7,3E+05	3,1E+05	4,6E+05	3	6,723	5	3	4	64	ALPL	
P19338	Nucleolin OS=Homo sapiens GN=NCL PE=1 SV=3	5,3E+05	1,2E+06	6,8E+05	3	9,161	5	3	6	95	NCL	
P02545	Prelamin-A/C OS=Homo sapiens GN=LMNA PE=1 SV=1	4,8E+05	1,3E+06		4	10,684	7	4	5	64	LMNA	
P07900	Heat shock protein HSP 90-alpha OS=Homo sapiens GN=HSP90AA1 PE=1 SV=5	3,6E+05	2,1E+06	9,4E+05	3	36,205	12	8	18	430	HSP90AA1	
P49411	Elongation factor Tu, mitochondrial OS=Homo sapiens GN=TUFM PE=1 SV=2	2,9E+05	3,9E+06	7,0E+06	5	20,572	17	5	15	160	TUFM	Methylthio [C290]
P62857	40S ribosomal protein S28 OS=Homo sapiens GN=RPS28 PE=1 SV=1	2,8E+05	7,3E+05	3,0E+05	3	11,294	39	3	5	118	RPS28	Methylthio [C27]
P55072	Transitional endoplasmic reticulum ATPase OS=Homo sapiens GN=VCP PE=1 SV=4	8,0E+04	2,1E+06	1,3E+06	4	10,985	6	4	8	110	VCP	

Figure 13: Result of the LC-MSMS analysis for target identification: CRM1 (Exportin) is the only high abundant protein which is highly enriched in the Bio-Ratjadone + Ratjadone (Bio Ratj + Ratj) sample. The enrichment factor is at least 100 fold. With 3 and more unique peptides 95 proteins were detected.

Plasma stability assay

FA-7-Val-Cit-pABA-16R-Aminoratjadone (PK008a), **FA-SS-16R-Aminoratjadone** (PK026a) and **FA-3b-L-Aminoratjadone** (PK058a) were dissolved in DMSO were added to human plasma (pH 7.4, 37°C) to yield a final concentration of 10 µg/ml. In addition, procaine, propoxycaine and procainamide (dissolved in DMSO) were added to human plasma (pH 7.4, 37°C) to yield a final concentration of 250 µM. Procaine and propoxycaine served as positive controls as they are known to be unstable in mouse plasma. Procainamide served as negative control as it is known to be stable in mouse plasma. The samples were incubated for 0 min, 30 min, 60 min, 90 min and 120 min at 37°C. At each time point 10 µl of the respective sample was extracted with 50 µl acetonitrile containing an internal standard for 15 min on ice. Then samples were centrifuged for 10 min at 1.000 rpm and the supernatants were transferred to greiner V-bottom 96well plates. All samples were analyzed via HPLC-MS using an Agilent 1290 Inifinity II HPLC system coupled to an AB Sciex QTrap 6500plus mass spectrometer. HPLC conditions were as follows: column: Agilent Zorbax Eclipse Plus C18, 50x2.1 mm, 1.8 µm; temperature: 30°C; injection volume: 10 µl; flow rate 700 µl/min; solvent A: water + 0.1 % HCOOH; solvent B: acetonitrile + 0.1 % HCOOH; gradient pump 1: 99 % A at 0 min, 99 % - 0% A from 0.1 min to 5.50 min, 0 % A until 6.00 min, then 99 % A until 6.40 min, then 99 % A until 6.50 min; gradient pump 2: 99 % A at 0 min, 99-0 % A from 0.20 to 1 min, 0 % A until 4.50 min, 0-99 % A from 4.50 to 5.20 min, 99 % A until 6.50 min. Mass spectrometric conditions were as follows: Scan type: MRM. All compounds were detected in positive scan mode. Peak areas of each compound and of the internal standard were analyzed using MultiQuant 3.0 software (AB Sciex). Peaks were quantified using the transitions indicated in table S7. Peak areas of the respective compound were normalized to the respective peak areas at time point 0 min: B/A*100 with A: peak area of the respective compound at time point 0 min, B: peak area of the respective internal standard at the respective time point. Every experiment was at least repeated three times independently.

Table S7: Transitions for the compounds

Compound	Q1 Mass [Da]	Q3 Mass [Da]	DP [volts]	CE [volts]	CXP [volts]
FA-7-Val-Cit-pABA-16R-Aminoratjadone $[M]^{2+}$	874.195	459.0	131	17	22
FA-7-Val-Cit-pABA-16R-Aminoratjadone $[M]^{2+}$	874.195	796.2	131	17	38
FA-SS-16R-Aminoratjadone $[M]^{2+}$	543.100	465.1	76	15	24
FA-SS-16R-Aminoratjadone $[M]^{2+}$	543.100	459.1	76	15	24
FA-3b-L-Aminoratjadone $[M]^{2+}$	798.324	459.0	61	19	36
FA-3b-L-Aminoratjadone $[M]^{2+}$	798.324	720.2	61	59	22
Procainamid	235.744	163.0	80	21	18
Procainamid	235.744	120.0	80	39	12
Propoxycain	294.738	100.1	80	17	12
Propoxycain	294.738	178.1	80	21	20
Procain	236.773	100.0	80	21	12
Procain	236.773	120.0	80	31	14

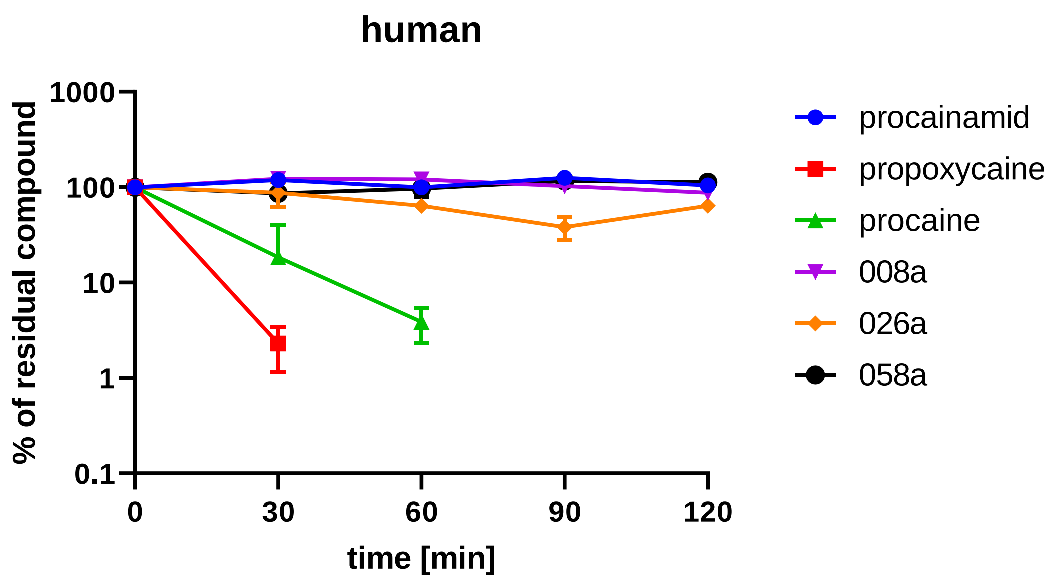

Figure S14: Logarithmic graph for stability in human plasma over time

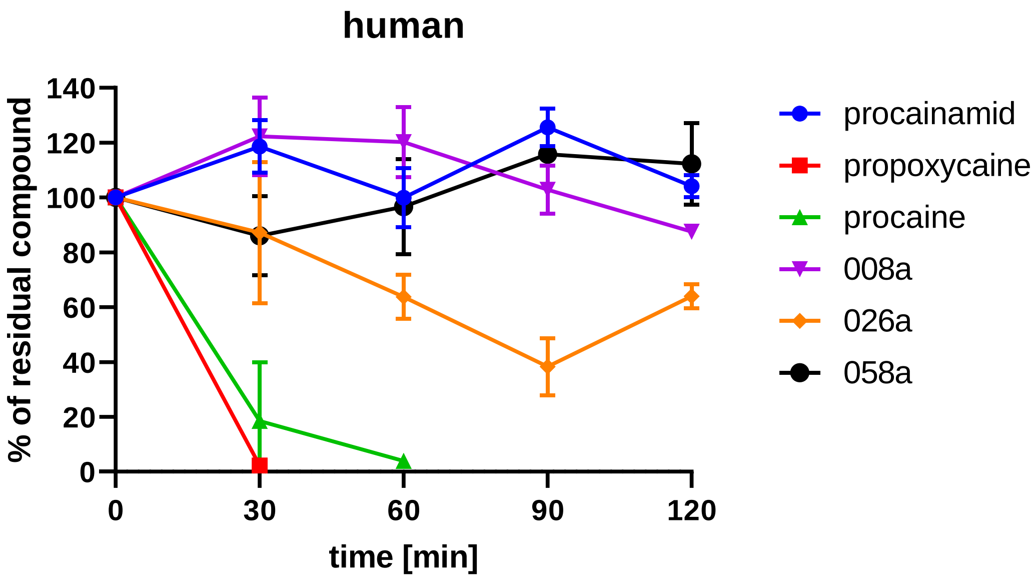

Figure 15: Linear graph for stability in human plasma over time

References

1 D. Schummer, K. Gerth, H. Reichenbach and G. Höfle, *Liebigs Ann.*, 1995, 685–688.

2 K. Gerth, D. Schummer, G. Höfle, H. Irschik and H. Reichenbach, *J. Antibiot.*, 1995, **48**, 973–976.

3 WO1998011084, 1998.

4 T. R. Hoye and M. K. Renner, *J. Org. Chem.*, 1996, **7**, 8489–8495.

5 J. A. Dale, D. L. Dull and H. Mosher, *J. Org. Chem.*, 1969, **34**, 2543–2549.

6 D. A. Allen, A. E. Tomaso, O. P. Priest and W. S. Colleges, *J. Chem. Educ.*, 2008, **85**, 698–700.

7 M. Galibert, P. Dumy and D. Boturyn, *Angew. Chem. Int. Ed.*, 2009, **48**, 2576–2579.

8 WO2014/065661 A1, 2014.

9 T. Mosmann, *J. Immunol. Methods*, 1983, **65**, 55–63.

10 M. Köster, S. Lykke-Andersen, Y. A. Elnakady, K. Gerth, P. Washausen, G. Höfle, F. Sasse, J. Kjems and H. Hauser, *Exp. Cell Res.*, 2003, **286**, 321–331.

11 S. K. Knauer, S. Moodt, T. Berg, U. Liebel, R. Pepperkok and R. H. Stauber, *Traffic*, 2005, **6**, 594–606.

12 S. K. Knauer and R. H. Stauber, *Anal. Chem.*, 2005, **77**, 4815–4820.

13 V. Fetz, S. K. Knauer, C. Bier, J. P. von Kries and R. H. Stauber, *Sensors*, 2009, **9**, 5423–5445.

14 D. Wessel and U. I. Flugge, *Anal. Biochem.*, 1984, **138**, 141–143.

15 D. A. Hartmann, *Curr. Protoc. Pharmacol.*, 2002, **19**, 7.6.1-7.6.8.

16 M. Frigerio, M. Santagostino and S. Sputore, *J. Org. Chem.*, 1999, **64**, 4537–4538.

17 J. Dommerholt, S. Schmidt, R. Temming, L. J. A. Hendriks, F. P. J. T. Rutjes, J. C. M. Van Hest, D. J. Lefeber, P. Friedl and F. L. Van Delft, *Angew. Chem. Int. Ed.*, 2010, **49**, 9422–9425.

18 C. B. Vu, R. J. Bridges, C. Pena-Rasgado, A. E. Lacerda, C. Bordwell, A. Sewell, A. J. Nichols, S. Chandran, P. Lonkar, D. Picarella, A. Ting, A. Wensley, M. Yeager and F. Liu, *J. Med. Chem.*, 2017, **60**, 458–473.

19 S. H. L. Verhelst, M. D. Witte, S. Arastu-Kapur, M. Fonovic and M. Bogyo, *ChemBioChem*, 2006, **7**, 943–950.

Appendix

^{1}H-NMR spectra, ^{13}C-NMR spectra, IR spectra

Ratjadone A (1)- (R)-6-((1E,3Z,5R,7E,9E,11R)-11-hydroxy-11-((2S,4R,5S,6S)-4-hydroxy-5-methyl-6-((E)-prop-1-en-1-yl)tetrahydro-2H-pyran-2-yl)-3,5,7-trimethylundeca-1,3,7,9-tetraen-1-yl)-5,6-dihydro-2H-pyran-2-one

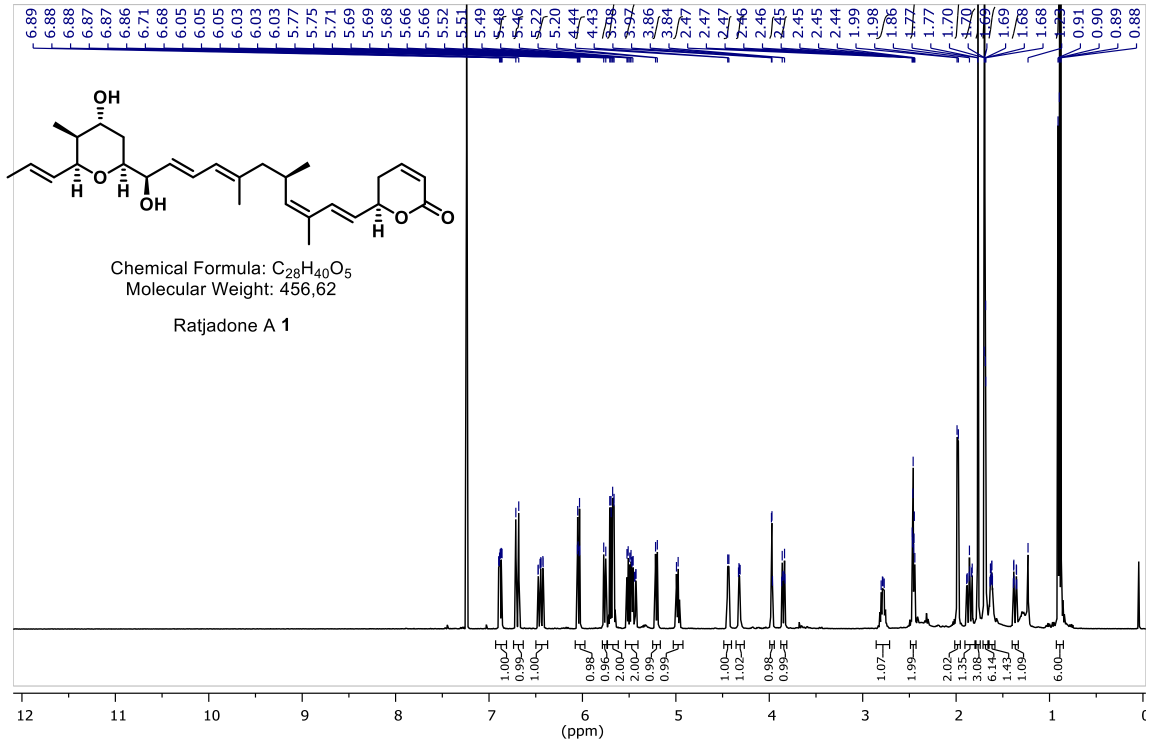

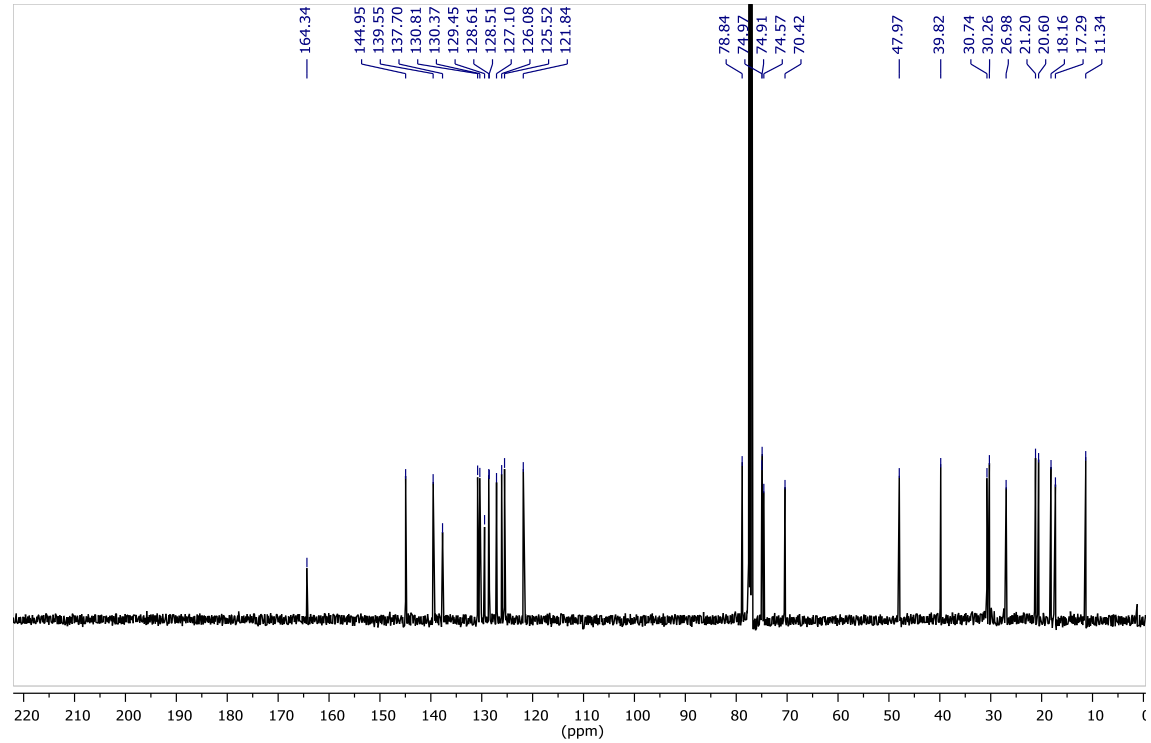

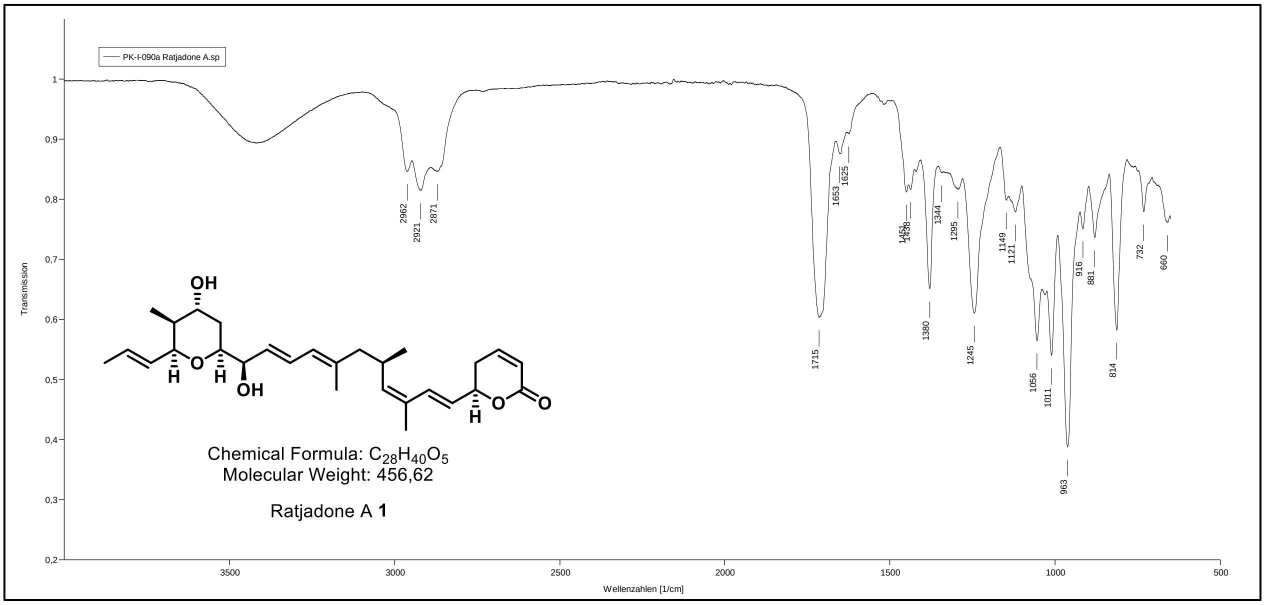
PK-I-090a Ratjadone A.sp
Transmission
Wellenzahlen [1/cm]
Chemical Formula: $C_{28}H_{40}O_5$
Molecular Weight: 456,62
Ratjadone A **1**
2962
2921
2871
1715
1653
1625
1451
1438
1380
1344
1295
1245
1149
1121
1056
1011
963
916
881
814
732
660

16-Oxo-Ratjadone (13) - (R)-6-((R,1E,3Z,7E,9E)-11-((2S,4R,5S,6S)-4-hydroxy-5-methyl-6-((E)-prop-1-en-1-yl)tetrahydro-2H-pyran-2-yl)-3,5,7-trimethyl-11-oxoundeca-1,3,7,9-tetraen-1-yl)-5,6-dihydro-2H-pyran-2-one

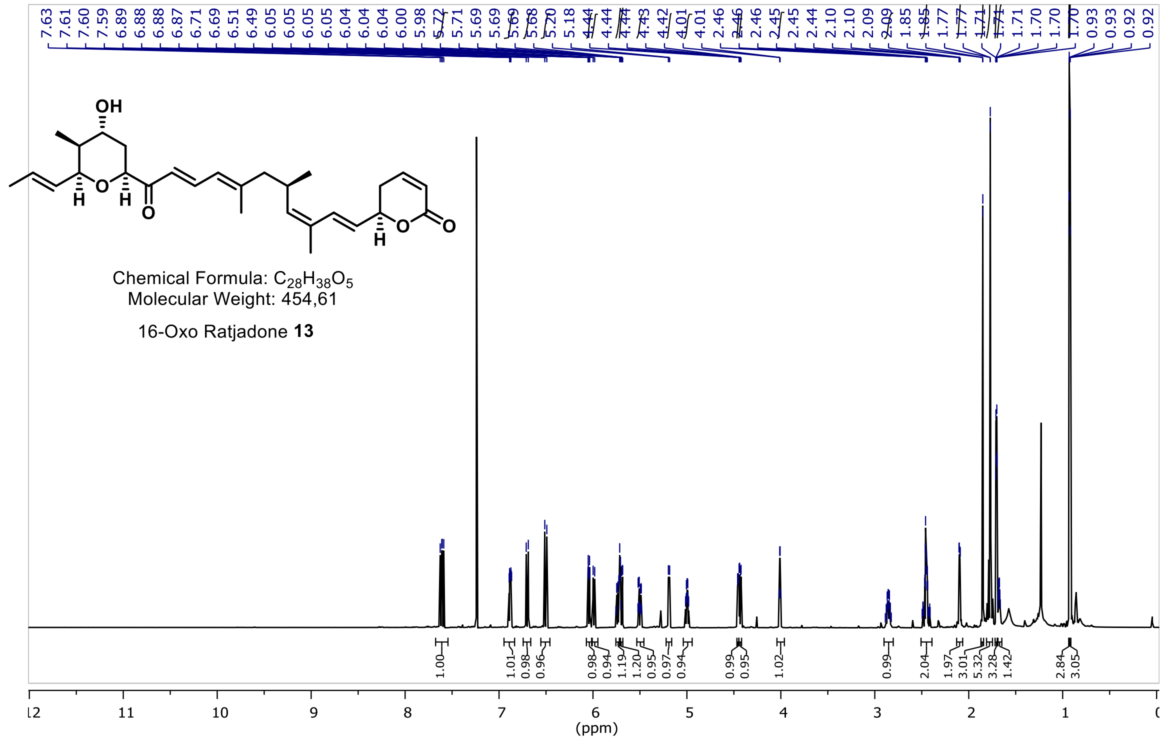

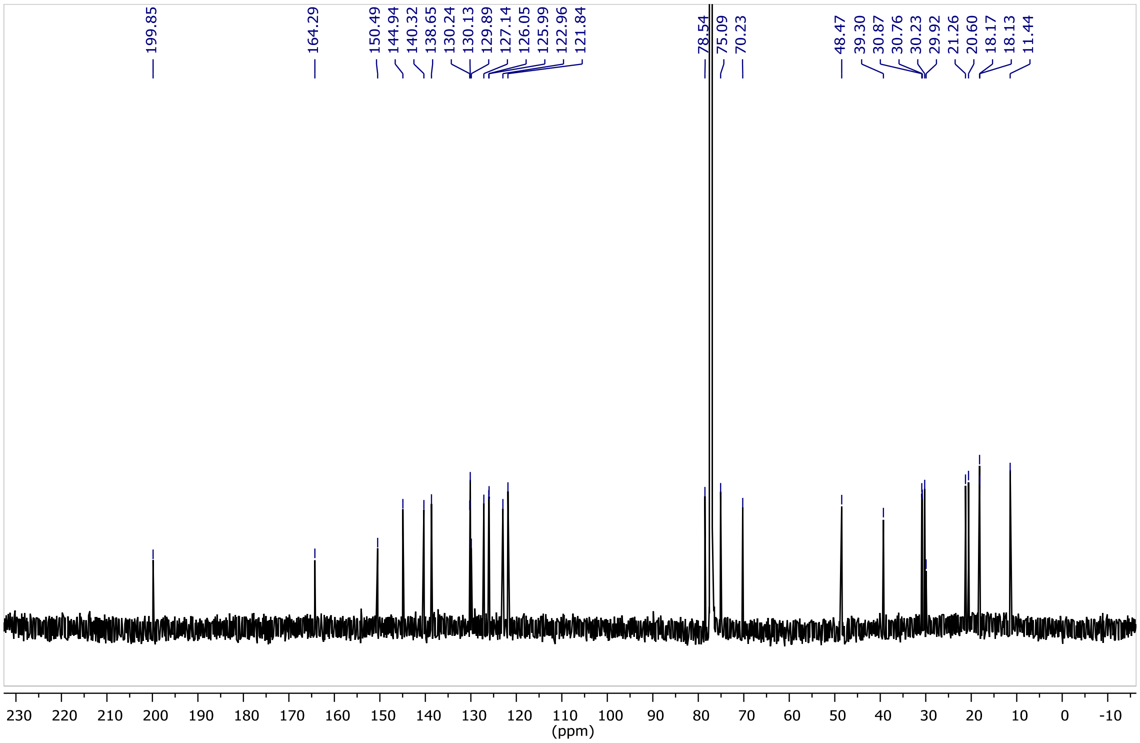

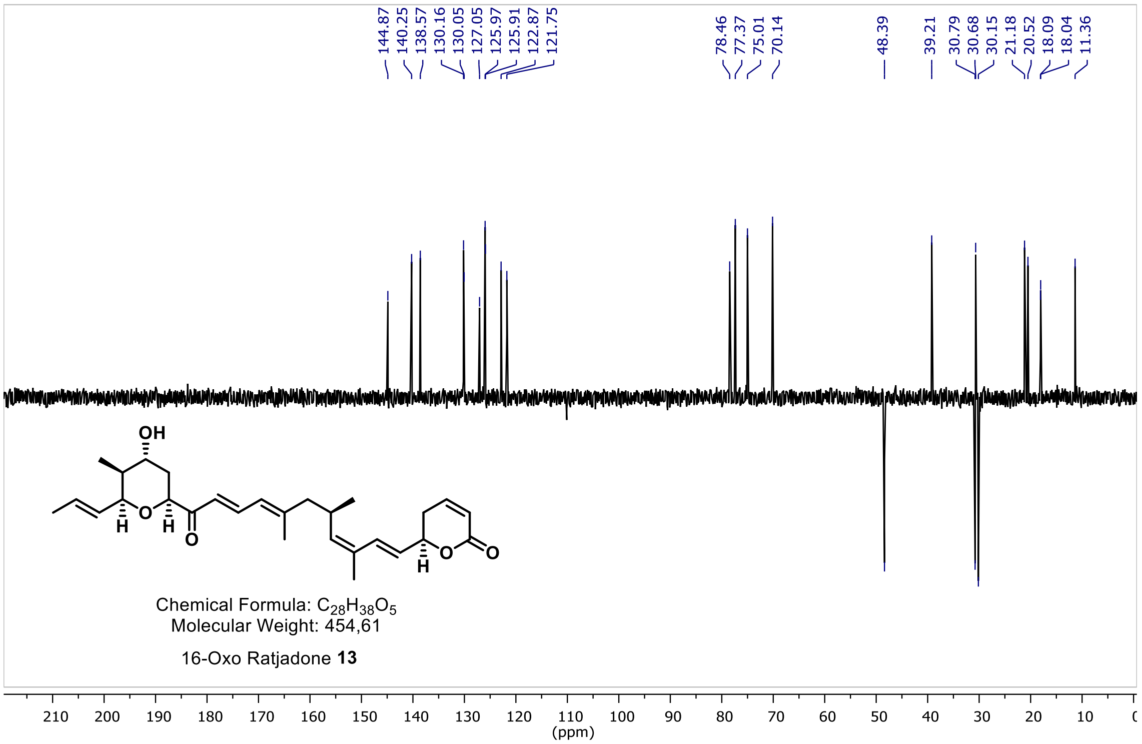
144.87
140.25
138.57
130.16
130.05
127.05
125.97
125.91
122.87
121.75
78.46
77.37
75.01
70.14
48.39
39.21
30.79
30.68
30.15
21.18
20.52
18.09
18.04
11.36
OH
H
O
H
O
H
O
O
Chemical Formula: C28H38O5
Molecular Weight: 454,61
16-Oxo Ratjadone 13
(ppm)

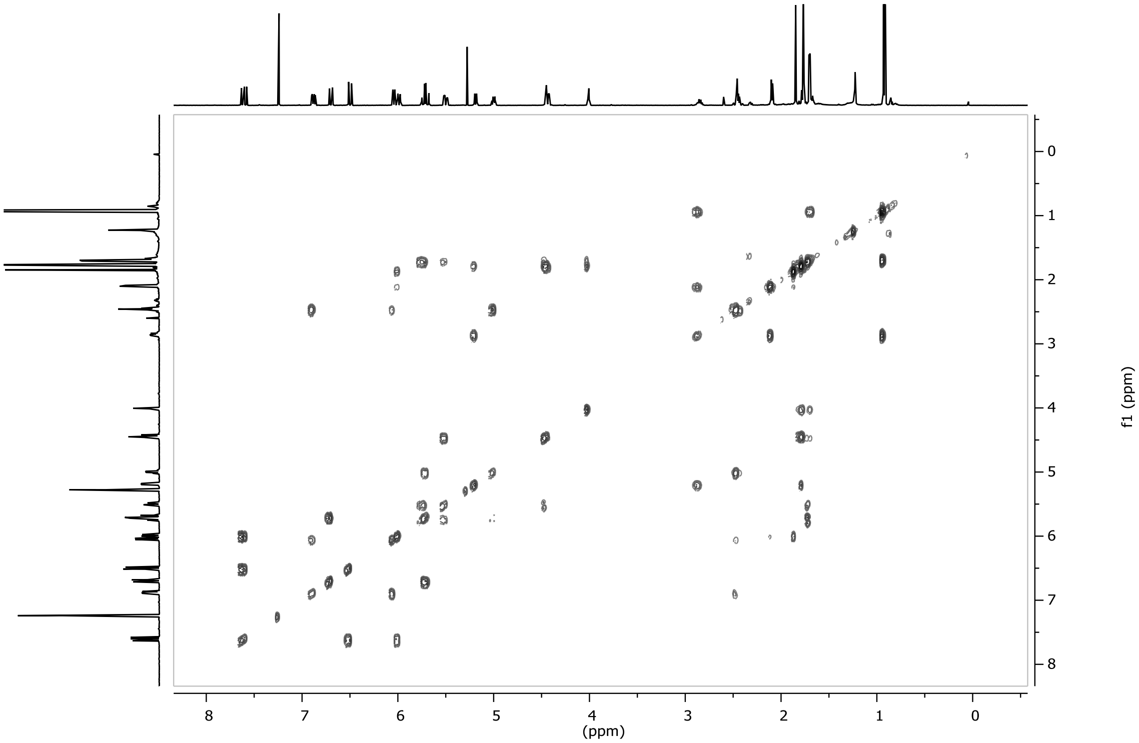
(ppm)
f1 (ppm)

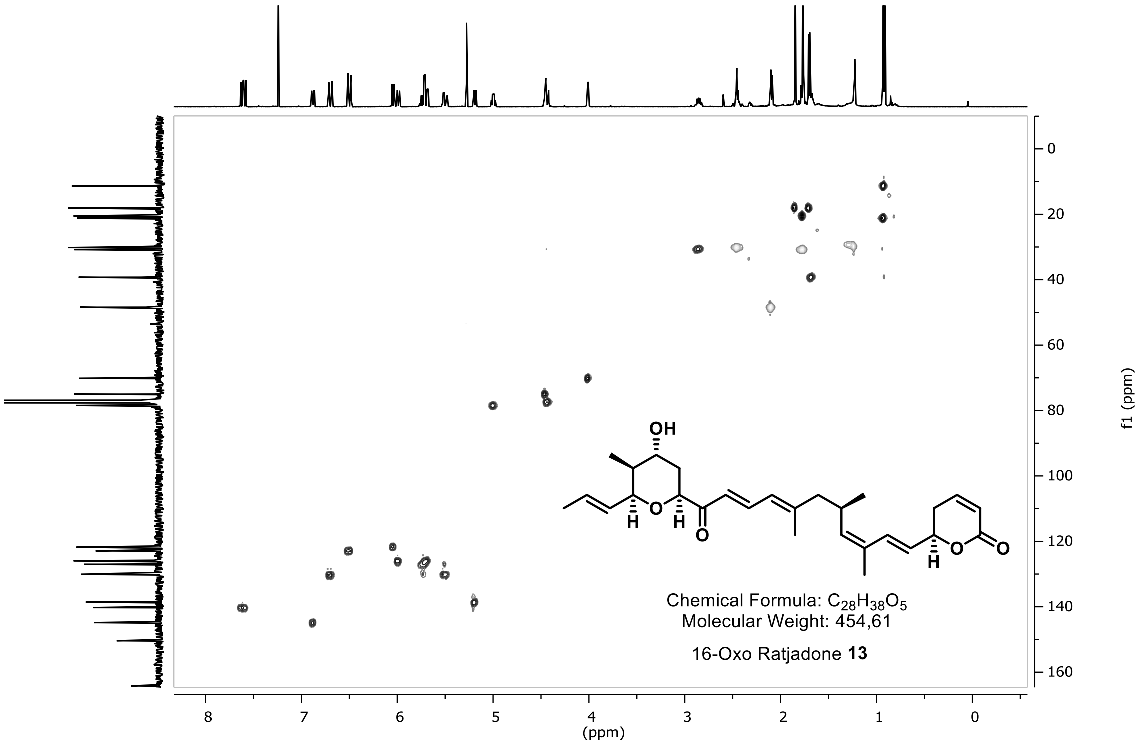
OH
H
O
H
O
H
O
O
Chemical Formula: $C_{28}H_{38}O_5$
Molecular Weight: 454,61
16-Oxo Ratjadone **13**
f1 (ppm)
(ppm)

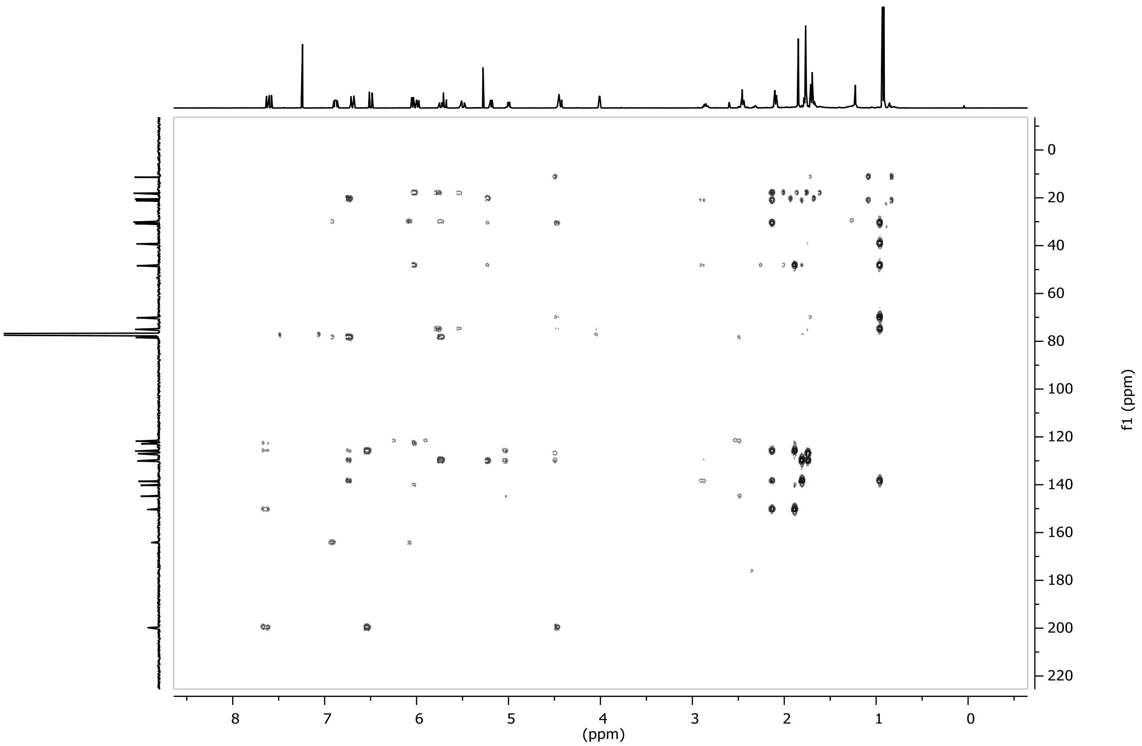
f1 (ppm)
(ppm)

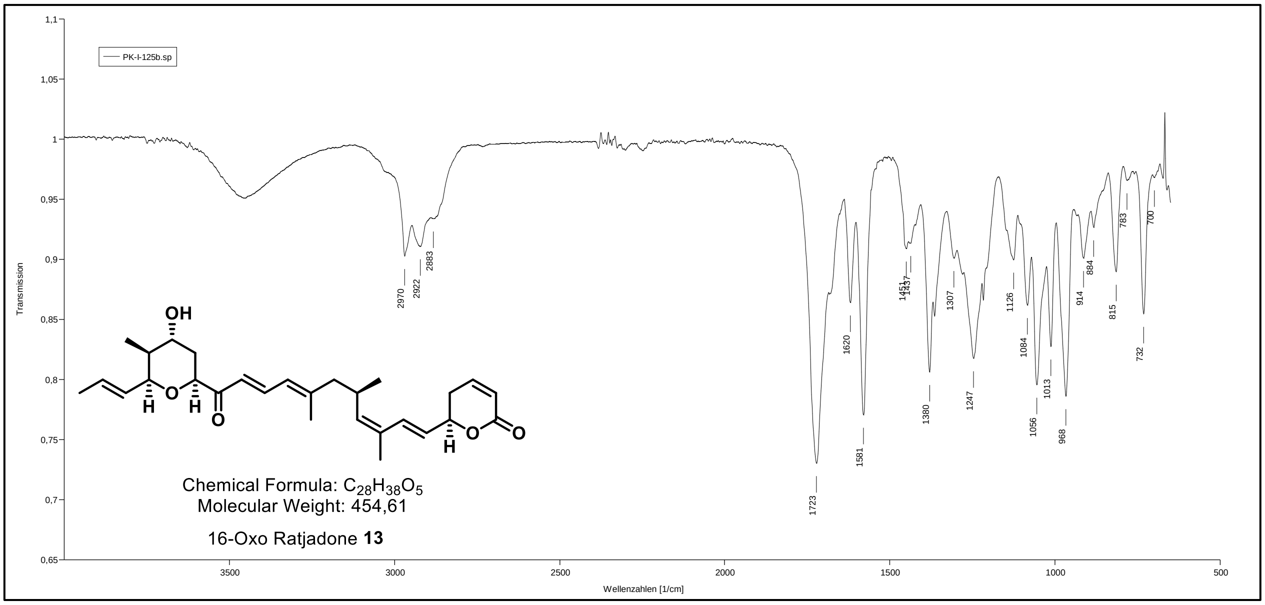
PK-I-125b.sp
Transmission
Wellenzahlen [1/cm]
2970
2922
2883
1723
1620
1581
1451
1437
1380
1307
1247
1126
1084
1056
1013
968
914
884
815
783
732
700
Chemical Formula: C28H38O5
Molecular Weight: 454,61
16-Oxo Ratjadone 13

19-Oxo-Ratjadone (14) - (R)-6-((1E,3Z,5R,7E,9E,11R)-11-hydroxy-3,5,7-trimethyl-11-((2S,5R,6S)-5-methyl-4-oxo-6-((E)-prop-1-en-1-yl)tetrahydro-2H-pyran-2-yl)undeca-1,3,7,9-tetraen-1-yl)-5,6-dihydro-2H-pyran-2-one

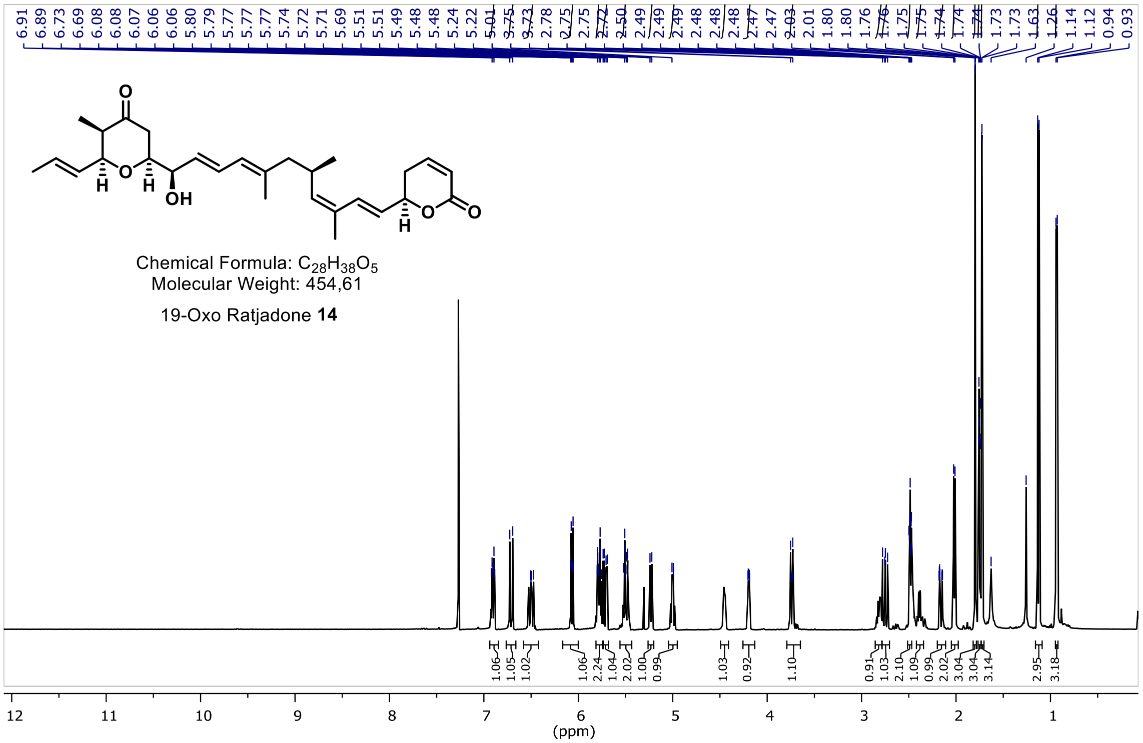

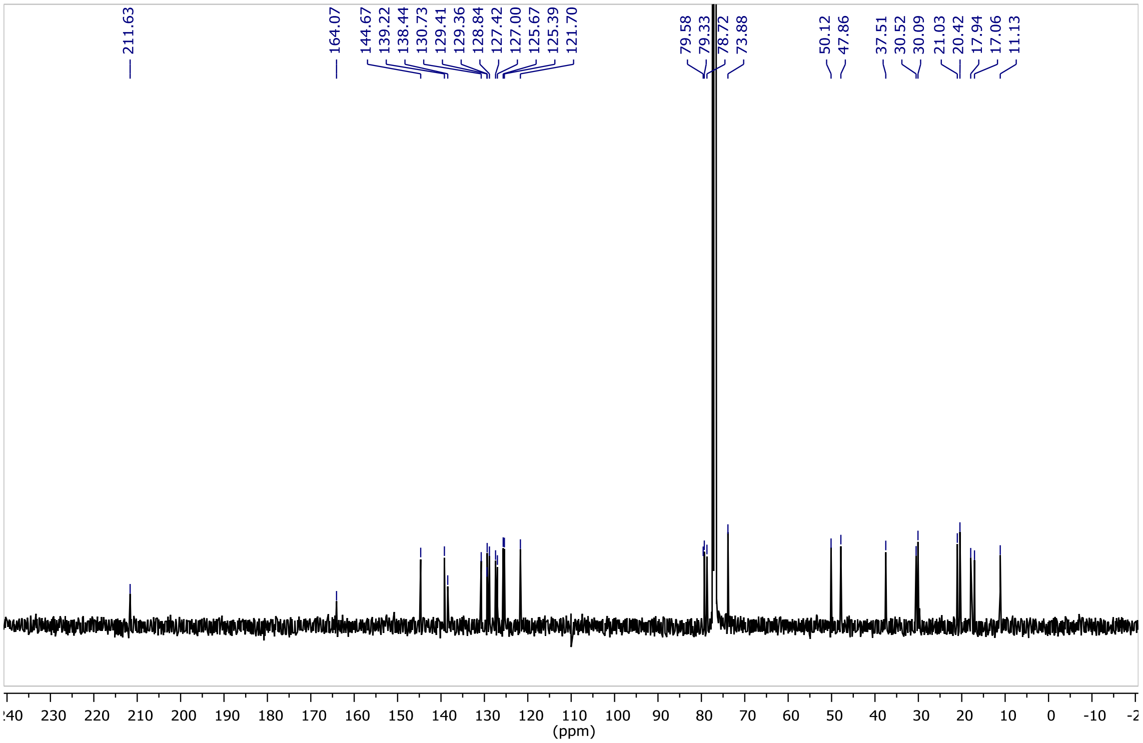

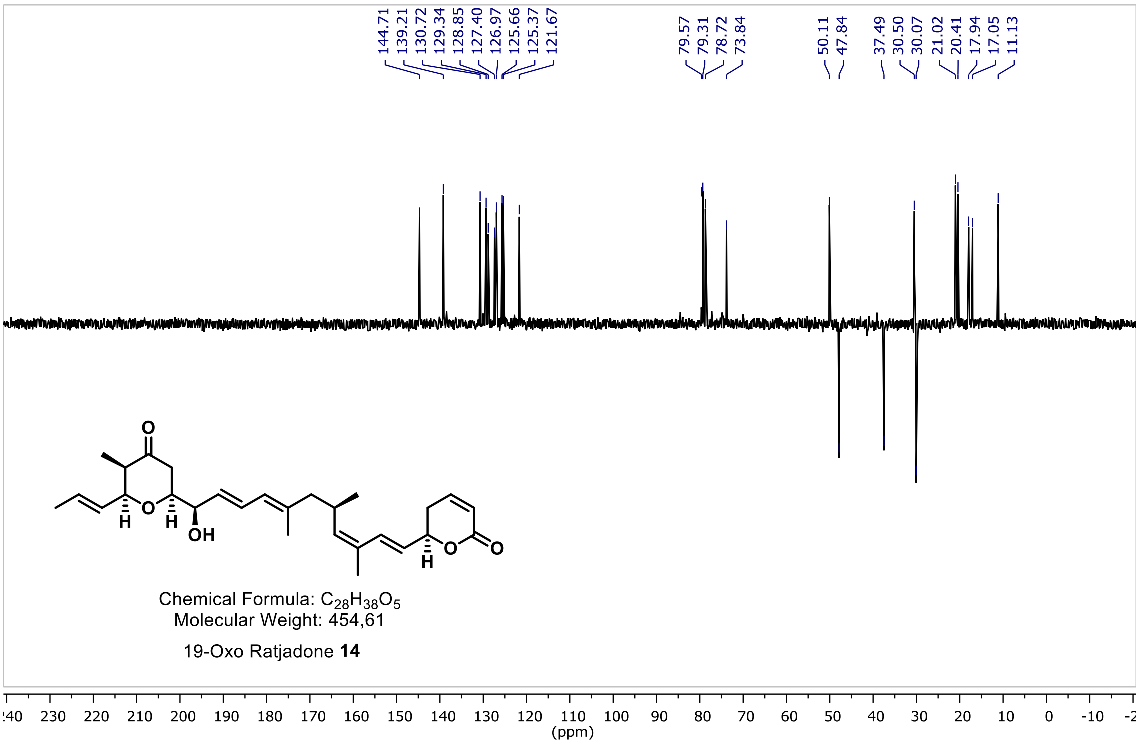
144.71
139.21
130.72
129.34
128.85
127.40
126.97
125.66
125.37
121.67
79.57
79.31
78.72
73.84
50.11
47.84
37.49
30.50
30.07
21.02
20.41
17.94
17.05
11.13
O
H
O
H
OH
O
H
O
Chemical Formula: $C_{28}H_{38}O_5$
Molecular Weight: 454,61
19-Oxo Ratjadone **14**
(ppm)

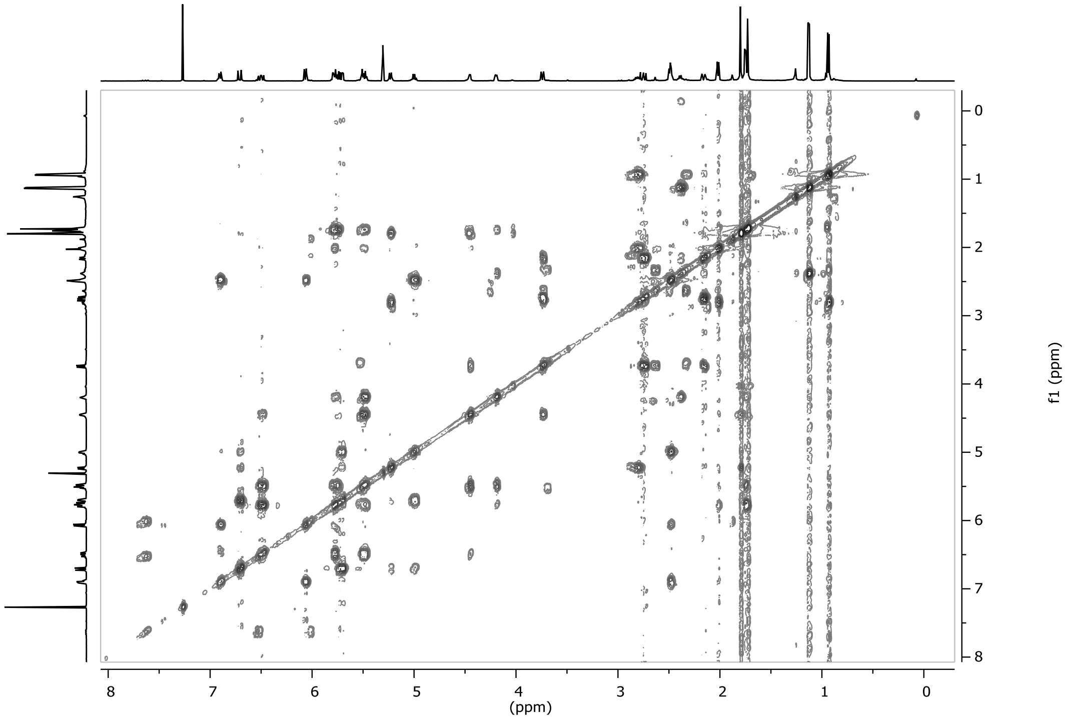
(ppm)
f1 (ppm)

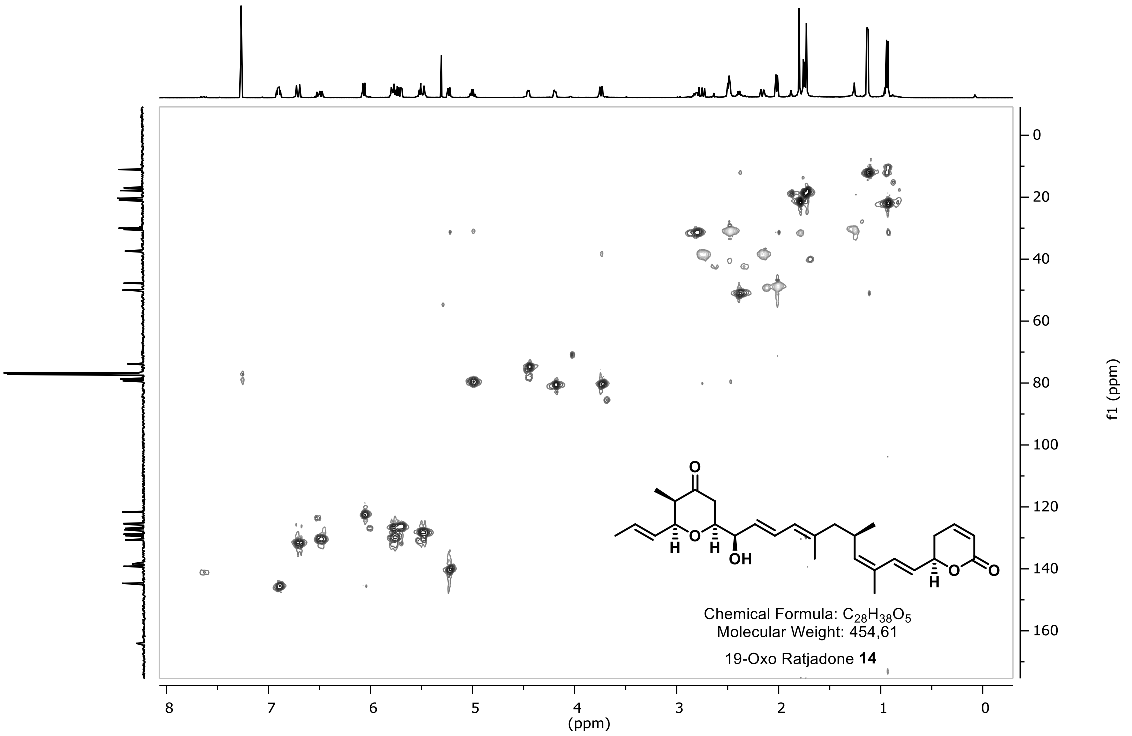

O
H
O
H
OH
H
O
O
Chemical Formula: $C_{28}H_{38}O_5$
Molecular Weight: 454,61
19-Oxo Ratjadone **14**
f1 (ppm)
(ppm)

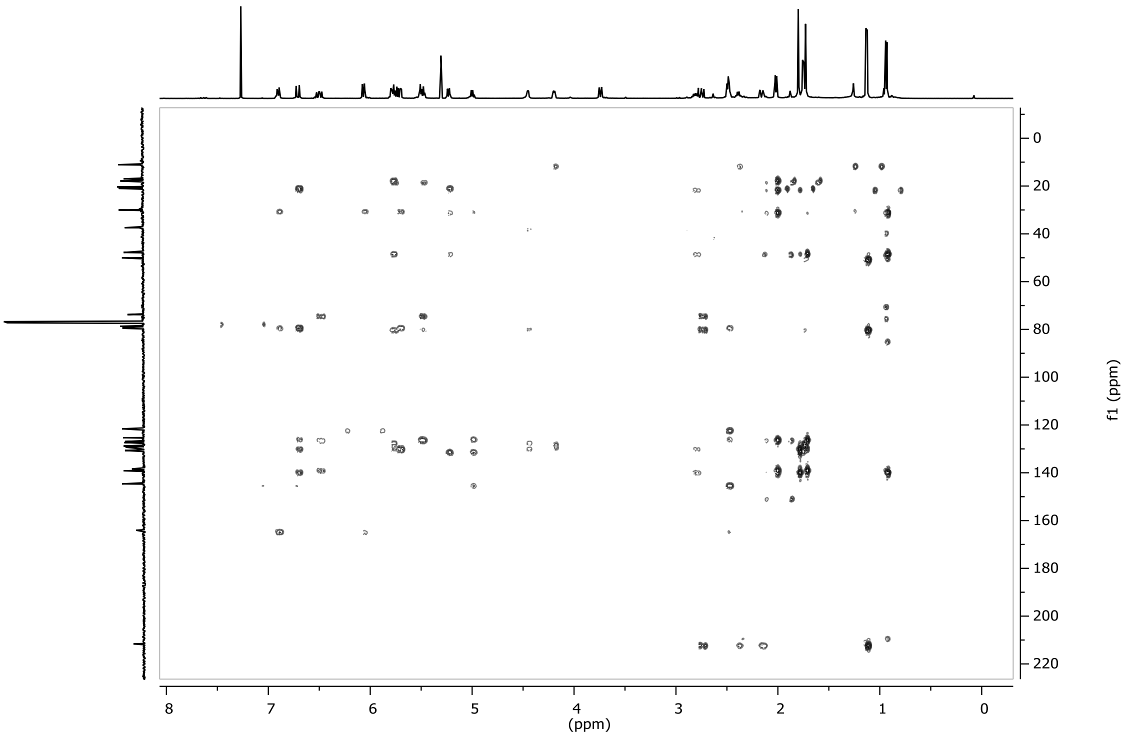

f1 (ppm)
(ppm)

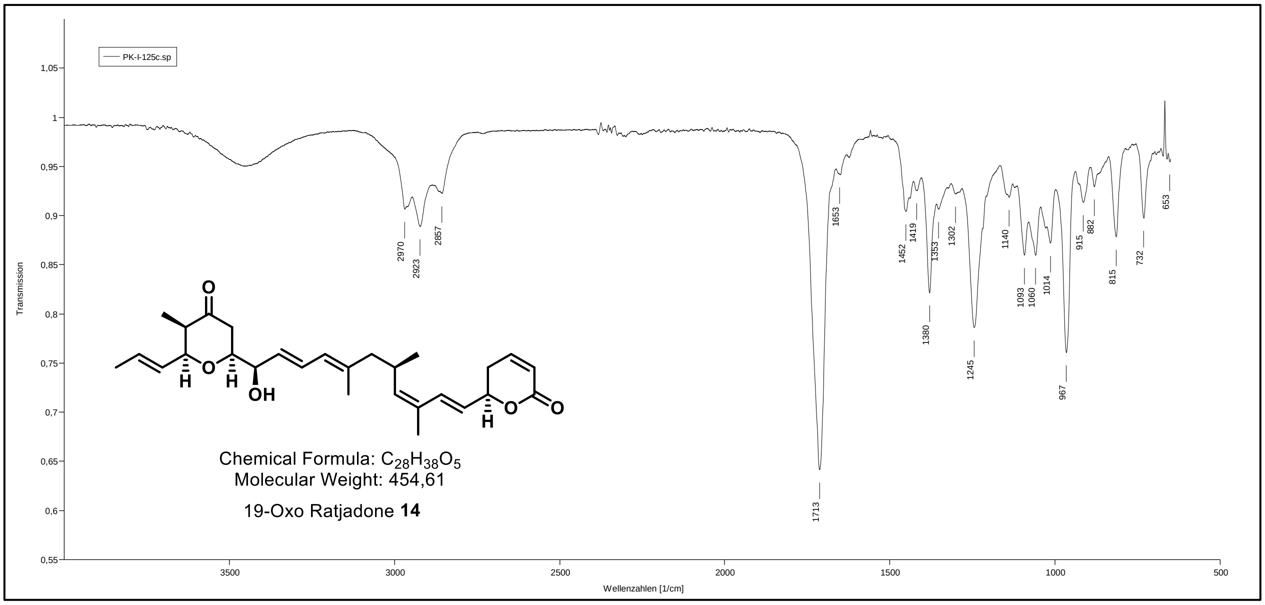
PK-I-125c.sp
Transmission
Wellenzahlen [1/cm]
1,05
1
0,95
0,9
0,85
0,8
0,75
0,7
0,65
0,6
0,55
3500
3000
2500
2000
1500
1000
500
2970
2923
2857
1713
1653
1452
1419
1380
1353
1302
1245
1140
1093
1060
1014
967
915
882
815
732
653
O
H
OH
Chemical Formula: $C_{28}H_{38}O_5$
Molecular Weight: 454,61
19-Oxo Ratjadone **14**

16,19-Dioxo-Ratjadone (15) – (R)-6-((R,1E,3Z,7E,9E)-3,5,7-trimethyl-11-((2S,5R,6S)-5-methyl-4-oxo-6-((E)-prop-1-en-1-yl)tetrahydro-2H-pyran-2-yl)-11-oxoundeca-1,3,7,9-tetraen-1-yl)-5,6-dihydro-2H-pyran-2-one

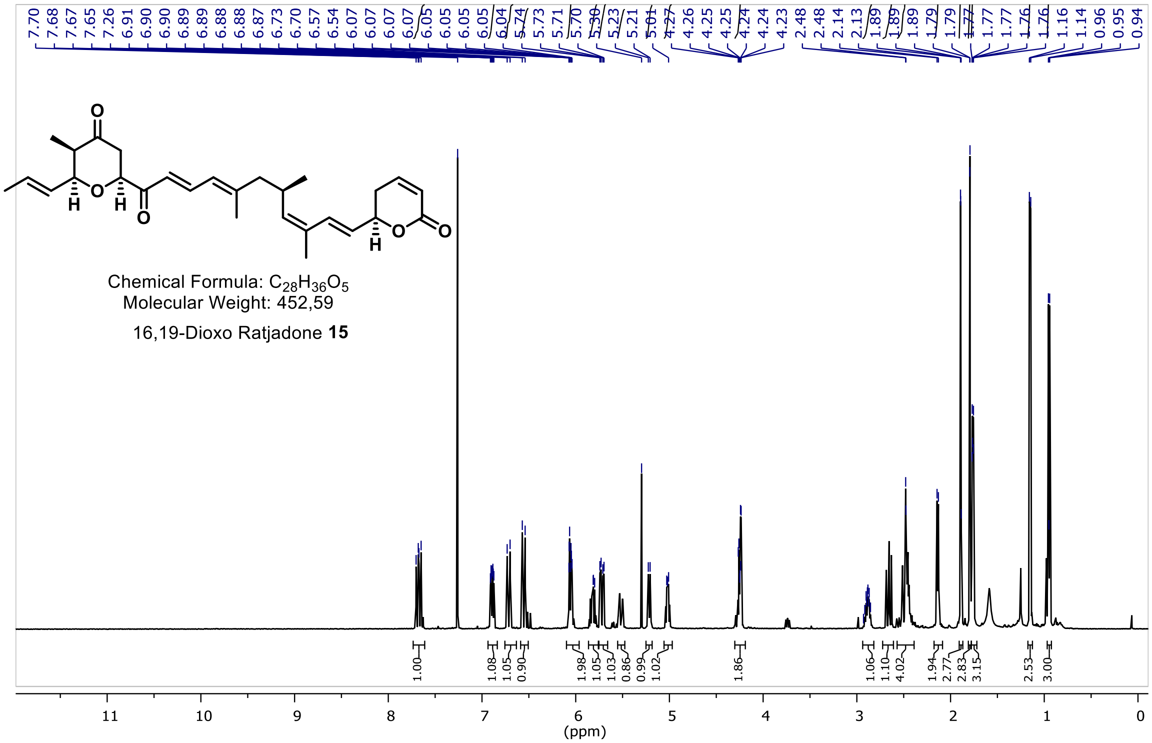

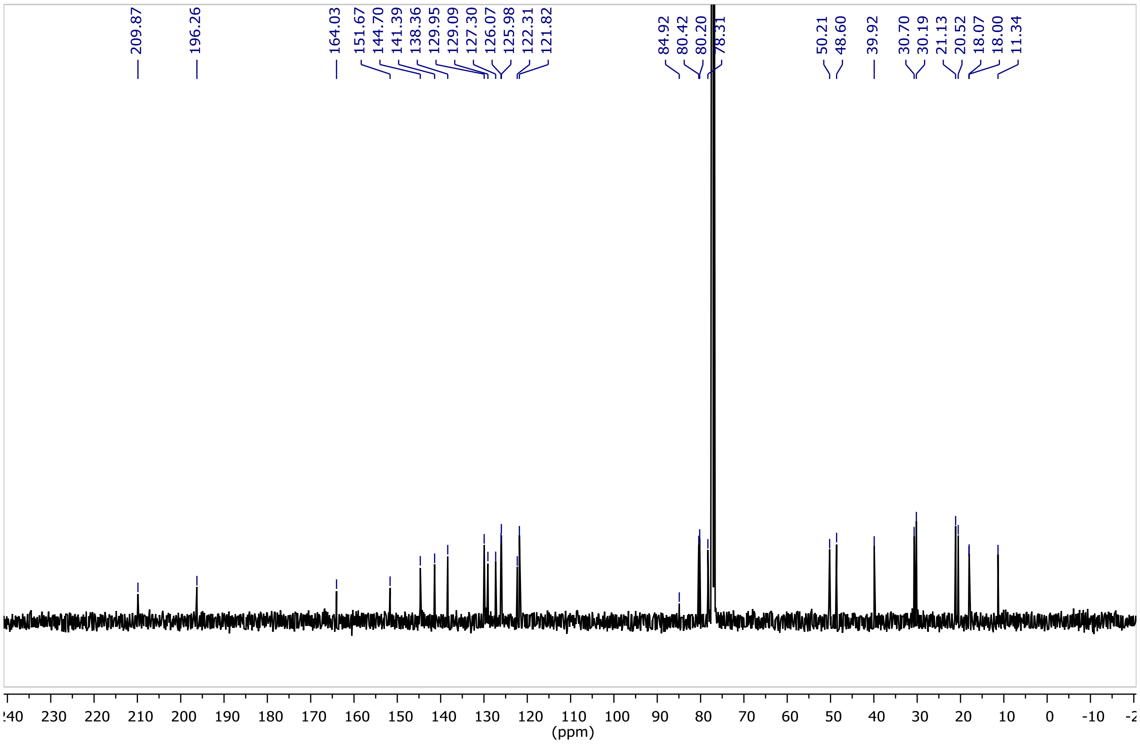

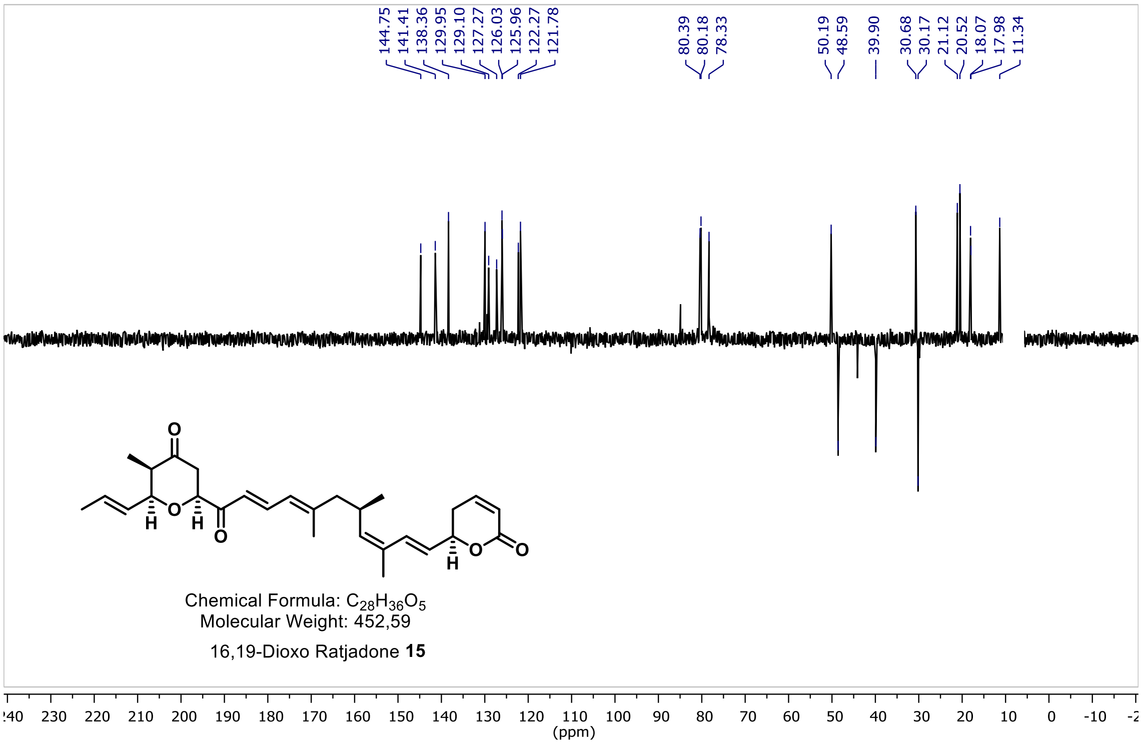
144.75
141.41
138.36
129.95
129.10
127.27
126.03
125.96
122.27
121.78
80.39
80.18
78.33
50.19
48.59
39.90
30.68
30.17
21.12
20.52
18.07
17.98
11.34
Chemical Formula: $C_{28}H_{36}O_5$
Molecular Weight: 452,59
16,19-Dioxo Ratjadone **15**
(ppm)

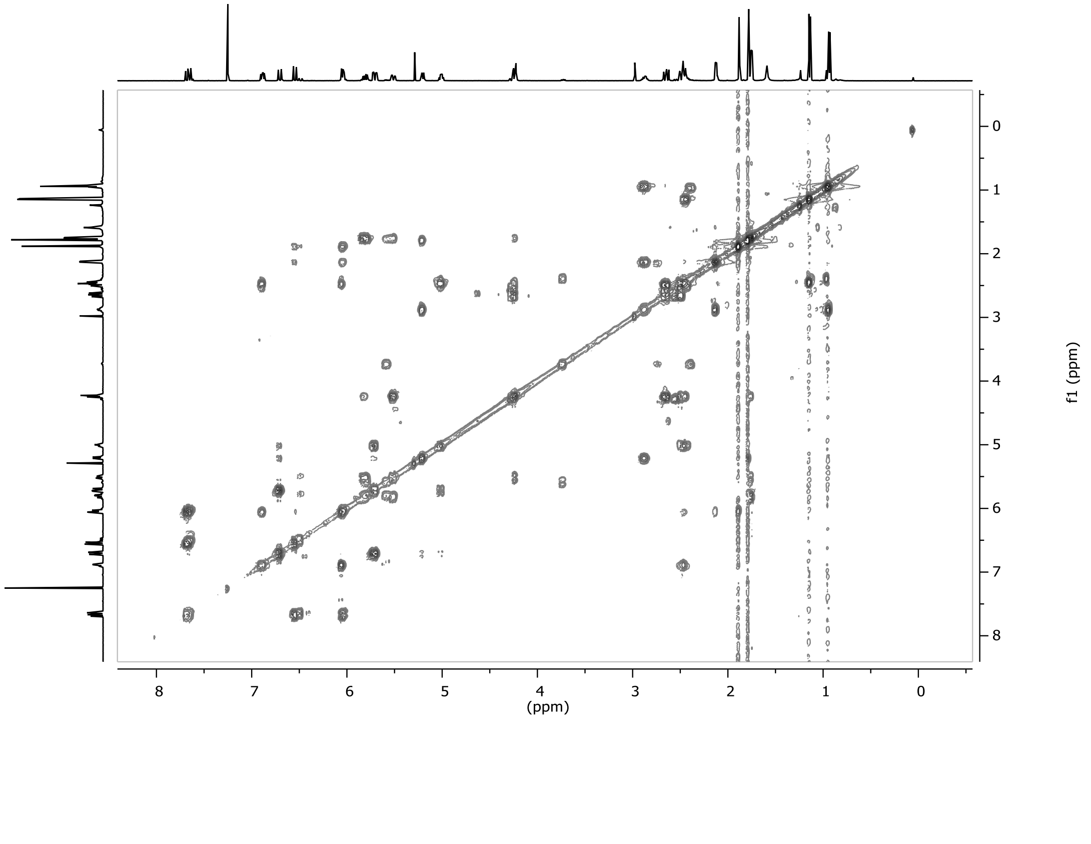
(ppm)
f1 (ppm)

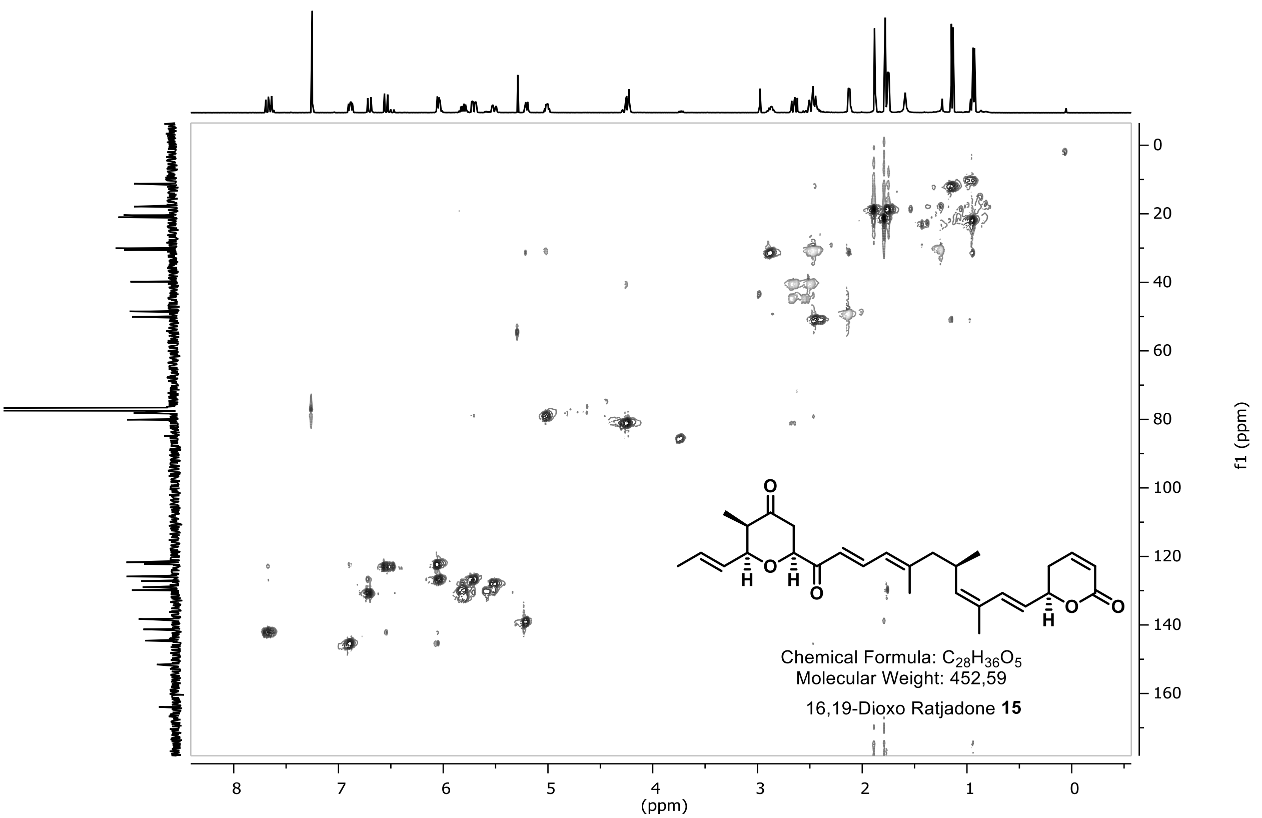
Chemical Formula: $C_{28}H_{36}O_5$
Molecular Weight: 452,59
16,19-Dioxo Ratjadone **15**
f1 (ppm)
(ppm)

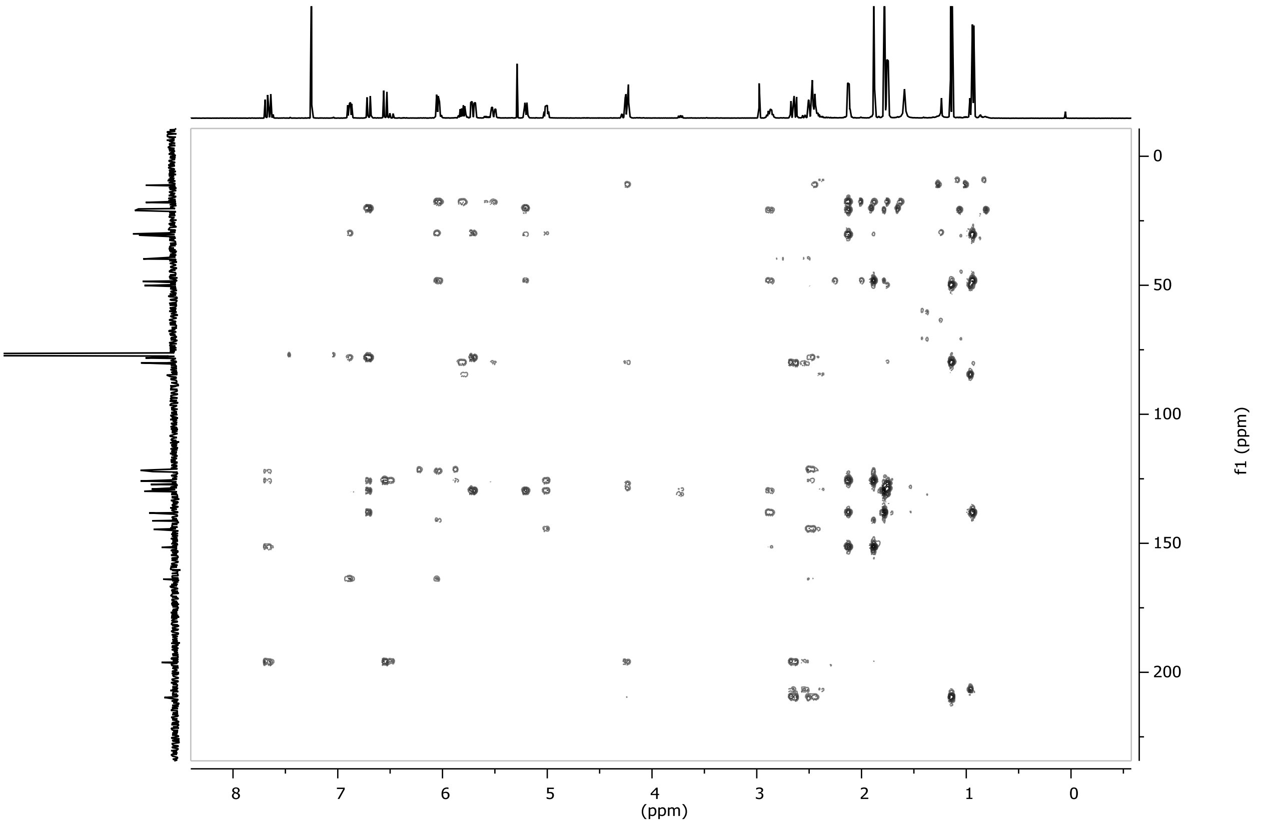
f1 (ppm)
(ppm)

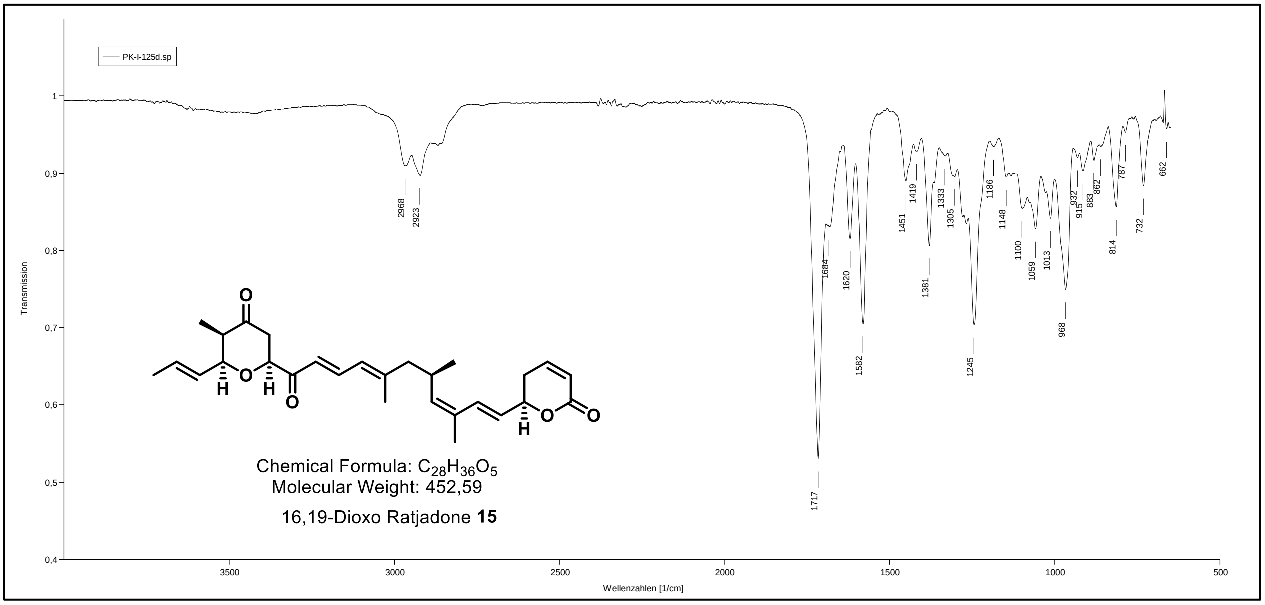
PK-I-125d.sp
Transmission
Wellenzahlen [1/cm]
2968
2923
1717
1684
1620
1582
1451
1419
1381
1333
1305
1245
1186
1148
1100
1059
1013
968
932
915
883
862
814
787
732
662
Chemical Formula: C28H36O5
Molecular Weight: 452,59
16,19-Dioxo Ratjadone 15

16*R*-Amino-Ratjadone (16) - (R)-6-((1E,3Z,5R,7E,9E,11R)-11-amino-11-((2S,4R,5S,6S)-4-hydroxy-5-methyl-6-((E)-prop-1-en-1-yl)tetrahydro-2H-pyran-2-yl)-3,5,7-trimethylundeca-1,3,7,9-tetra-en-1-yl)-5,6-dihydro-2H-pyran-2-one

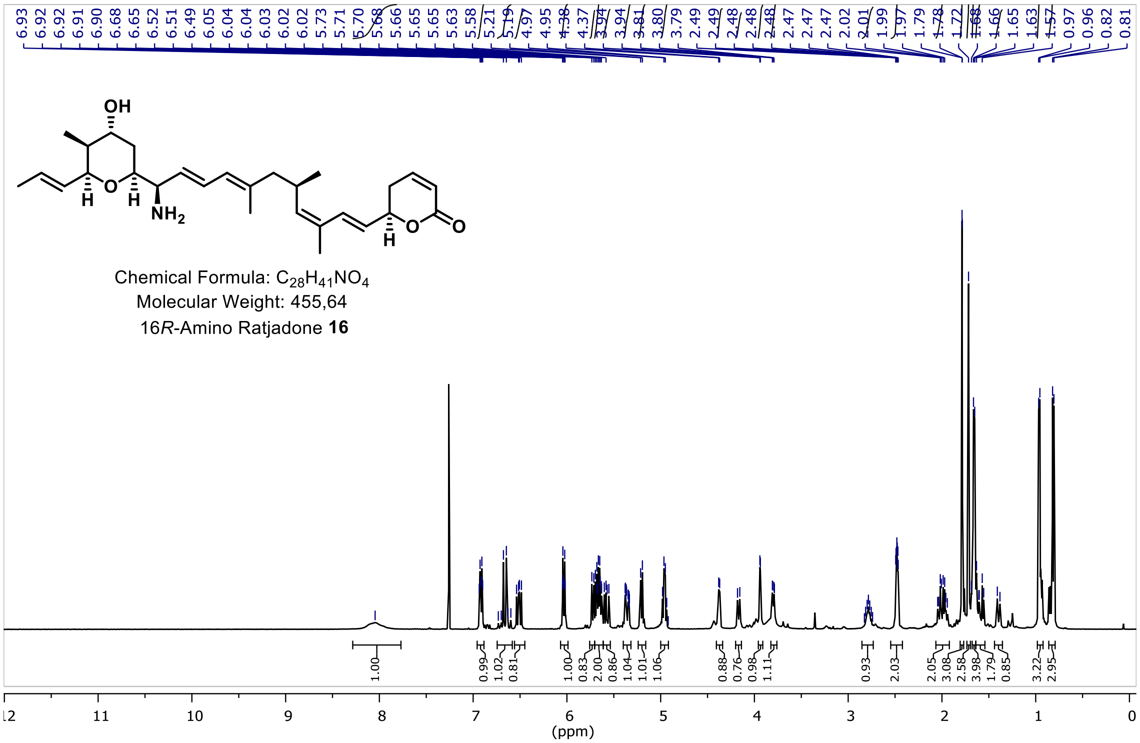

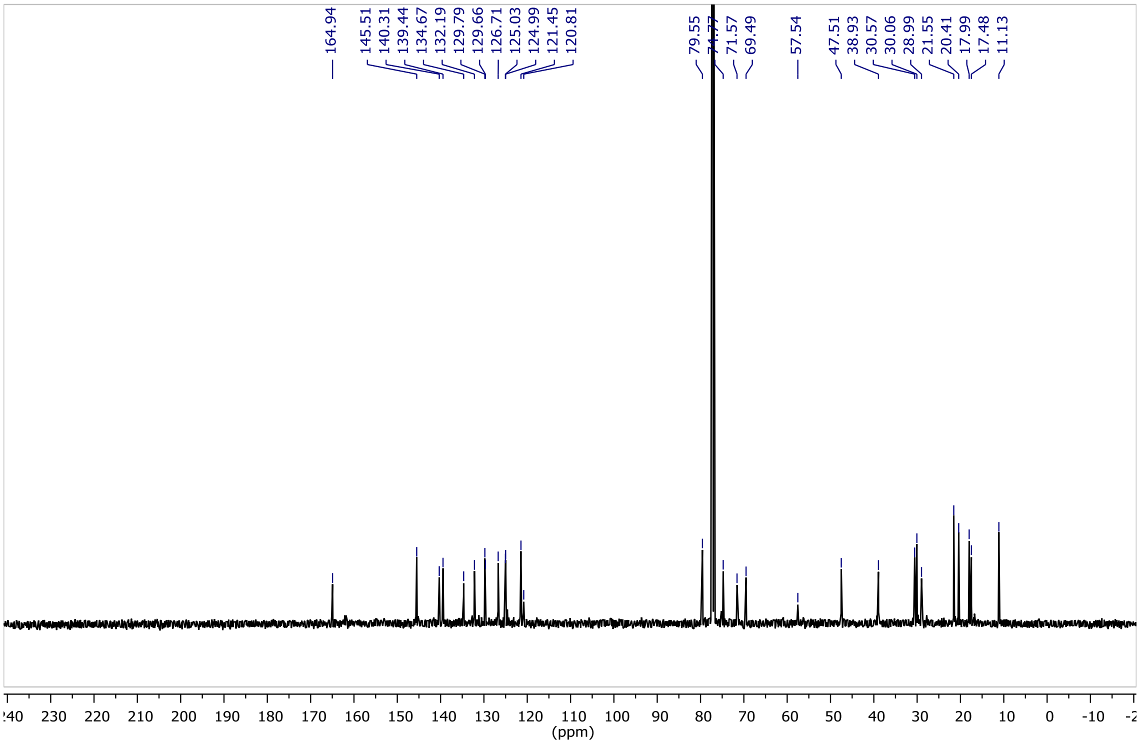

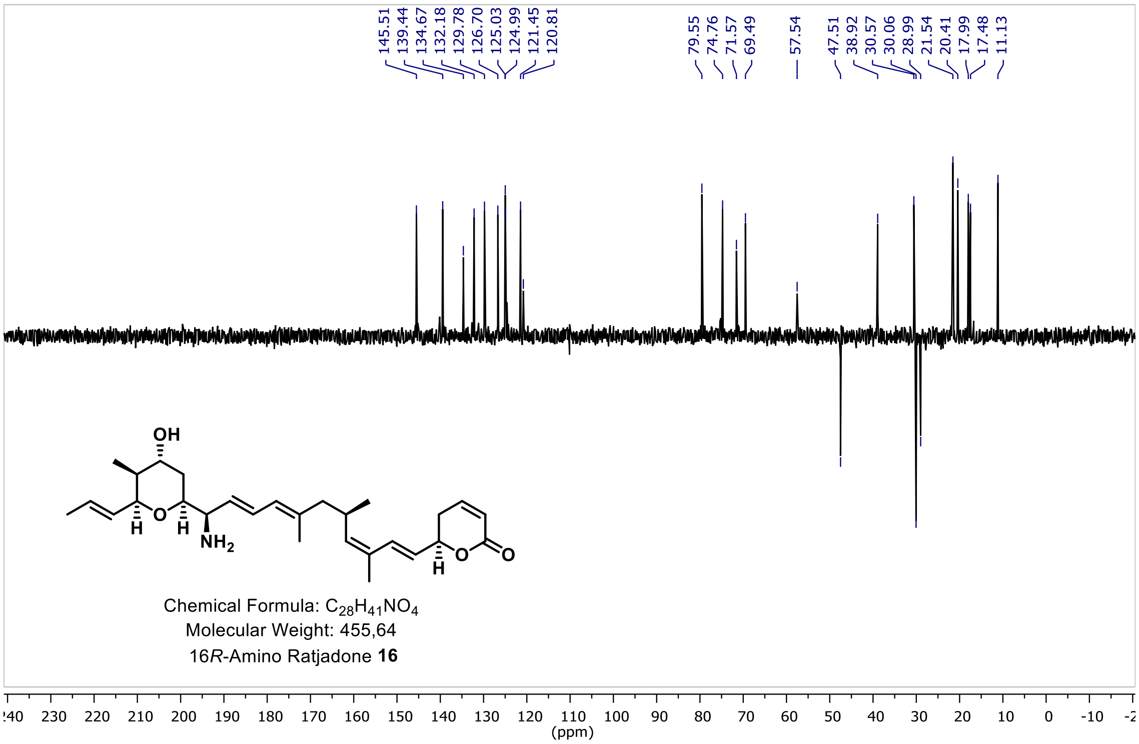
145.51
139.44
134.67
132.18
129.78
126.70
125.03
124.99
121.45
120.81
79.55
74.76
71.57
69.49
57.54
47.51
38.92
30.57
30.06
28.99
21.54
20.41
17.99
17.48
11.13
OH
O
H
H
NH2
H
O
O
Chemical Formula: $C_{28}H_{41}NO_4$
Molecular Weight: 455,64
16*R*-Amino Ratjadone **16**
240 230 220 210 200 190 180 170 160 150 140 130 120 110 100 90 80 70 60 50 40 30 20 10 0 -10 -2
(ppm)

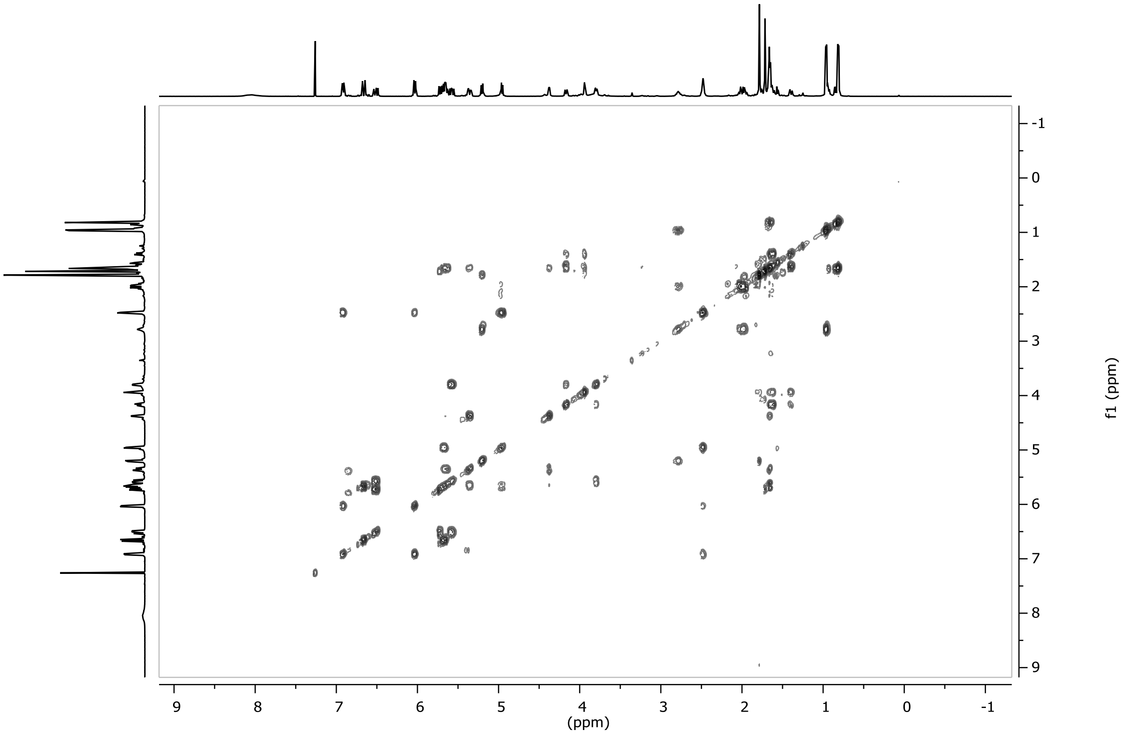
-1
0
1
2
3
4
5
6
7
8
9
f1 (ppm)
9 8 7 6 5 4 3 2 1 0 -1
(ppm)

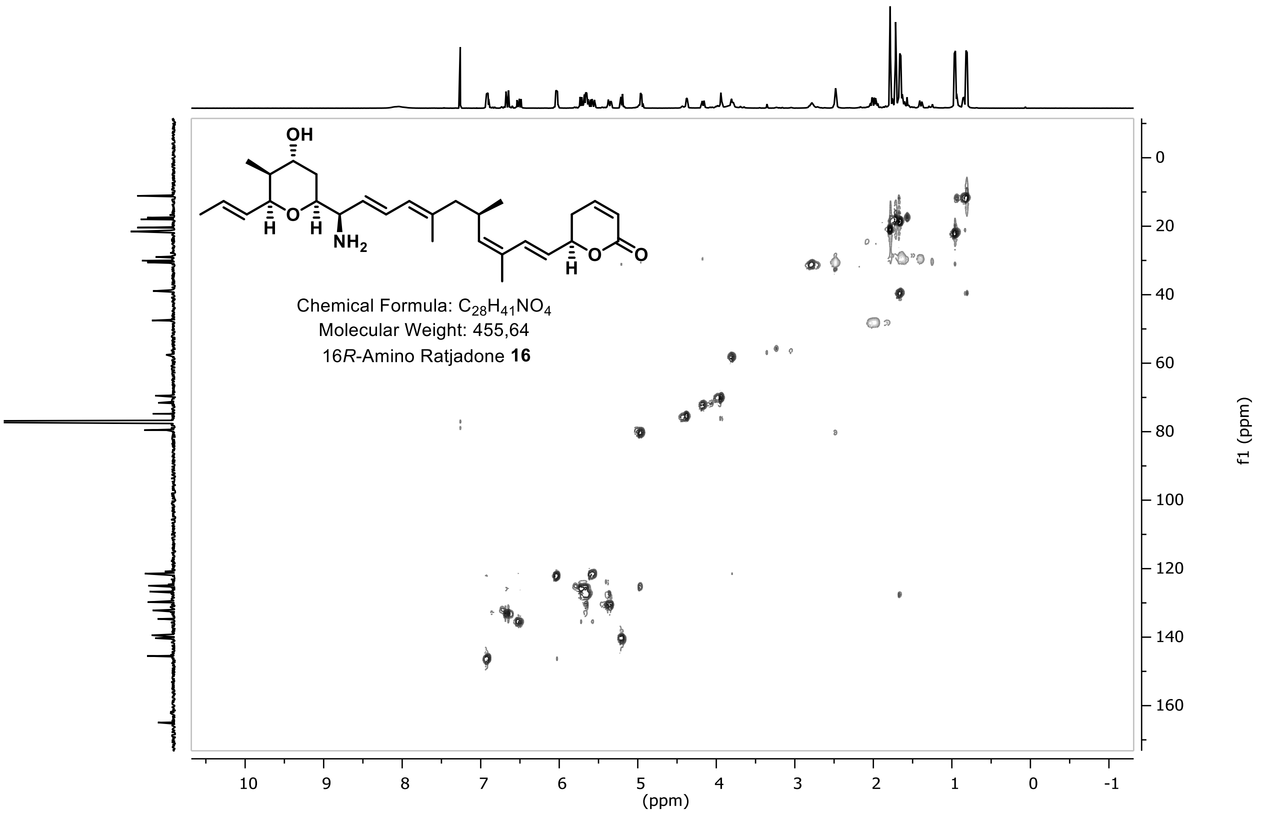
OH
O
H
H
NH2
O
O
H
Chemical Formula: C28H41NO4
Molecular Weight: 455,64
16R-Amino Ratjadone 16
0
20
40
60
80
100
120
140
160
f1 (ppm)
10
9
8
7
6
5
4
3
2
1
0
-1
(ppm)

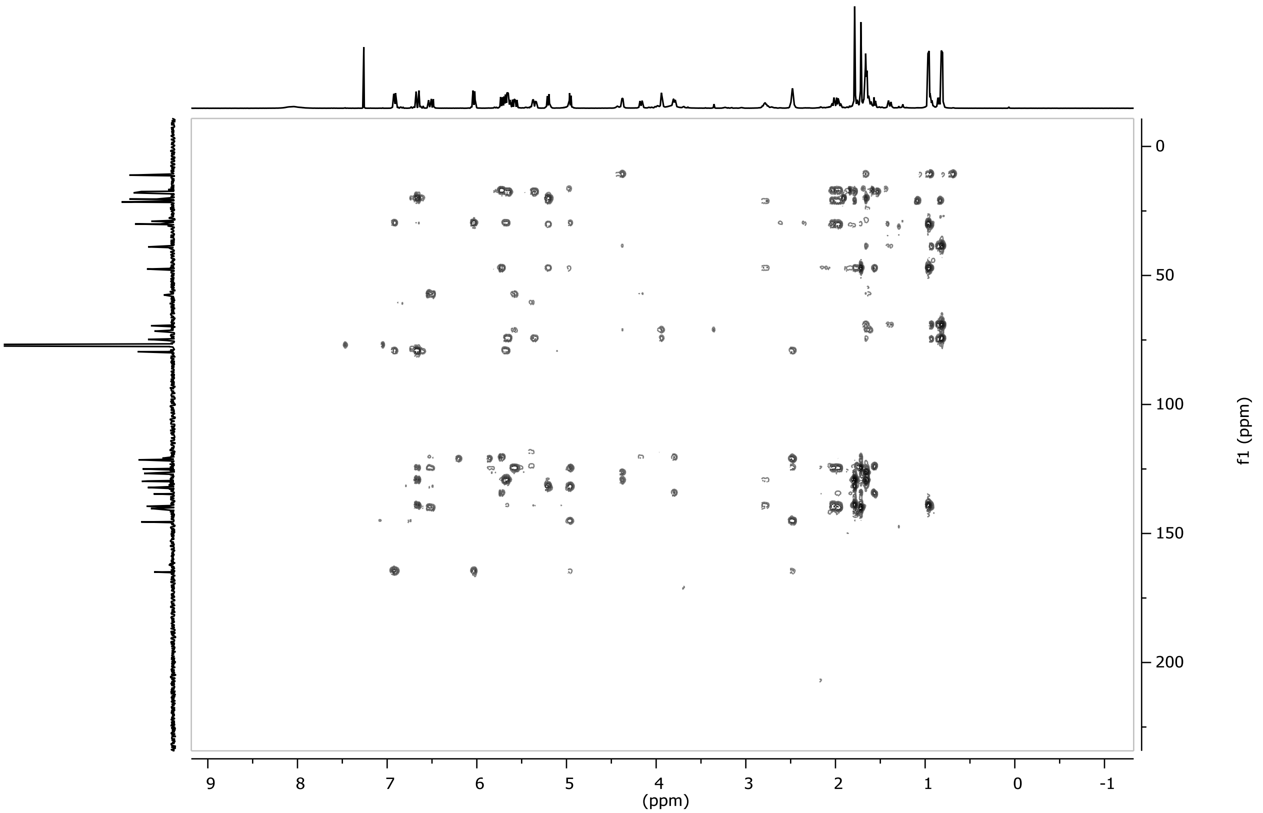
0
50
100
150
200
f1 (ppm)
9
8
7
6
5
4
3
2
1
0
-1
(ppm)

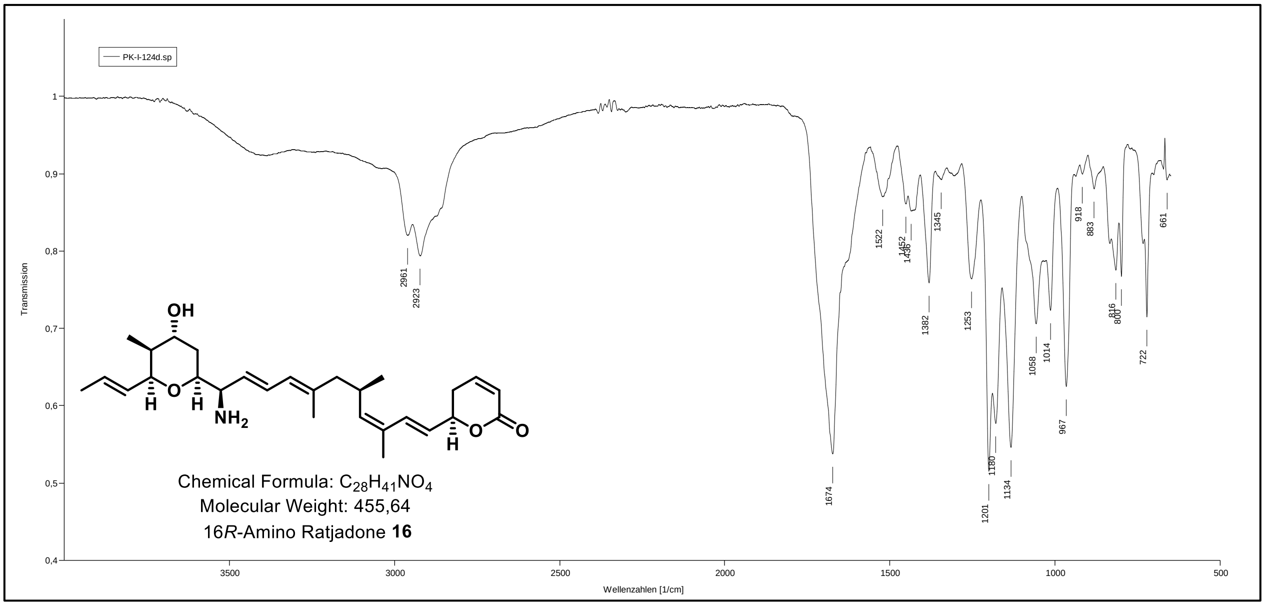
PK-I-124d.sp
Transmission
Wellenzahlen [1/cm]
Chemical Formula: C28H41NO4
Molecular Weight: 455,64
16R-Amino Ratjadone 16
2961
2923
1674
1522
1452
1436
1382
1345
1253
1201
1180
1134
1058
1014
967
918
883
816
800
722
661

16*S*-Amino-Ratjadone (17) - (R)-6-((1E,3Z,5R,7E,9E,11S)-11-amino-11-((2S,4R,5S,6S)-4-hydroxy-5-methyl-6-((E)-prop-1-en-1-yl)tetrahydro-2H-pyran-2-yl)-3,5,7-trimethylun-deca-1,3,7,9-tetraen-1-yl)-5,6-dihydro-2H-pyran-2-one

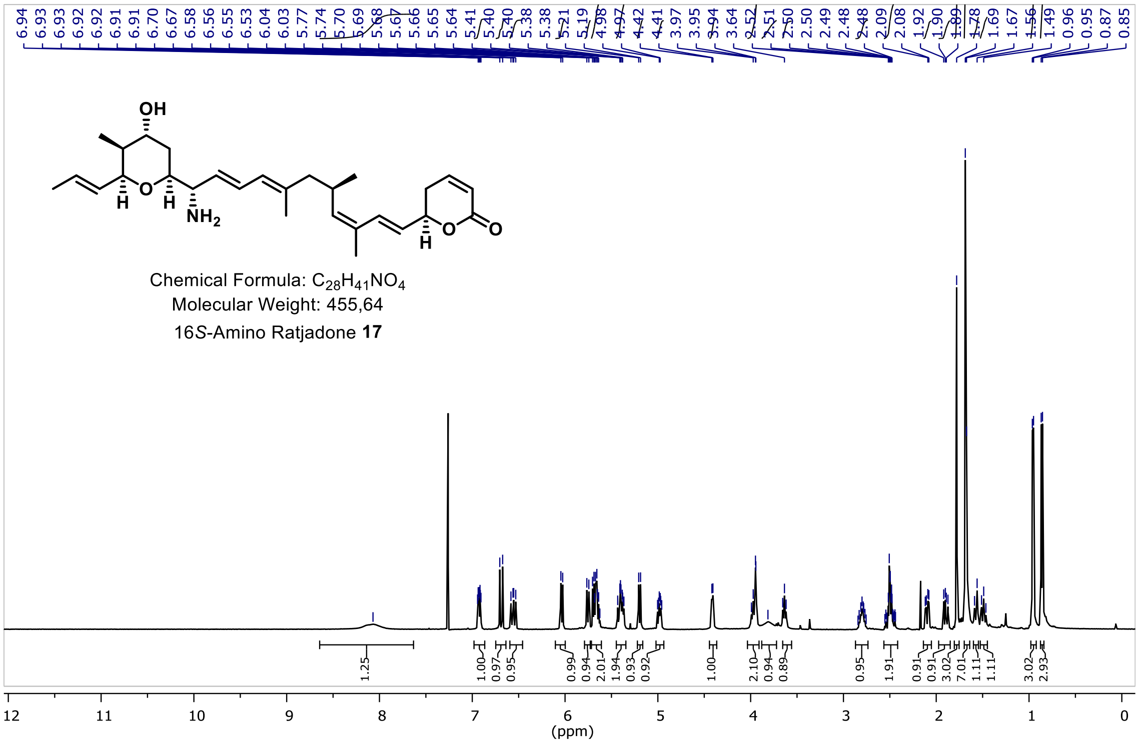

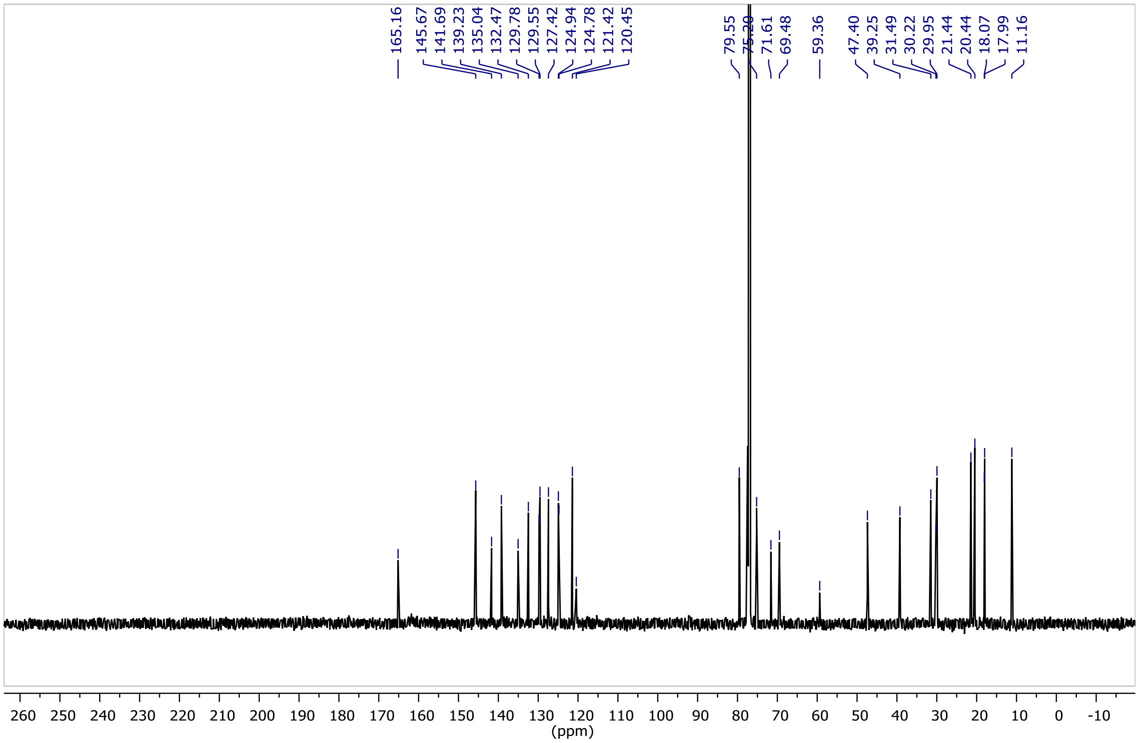

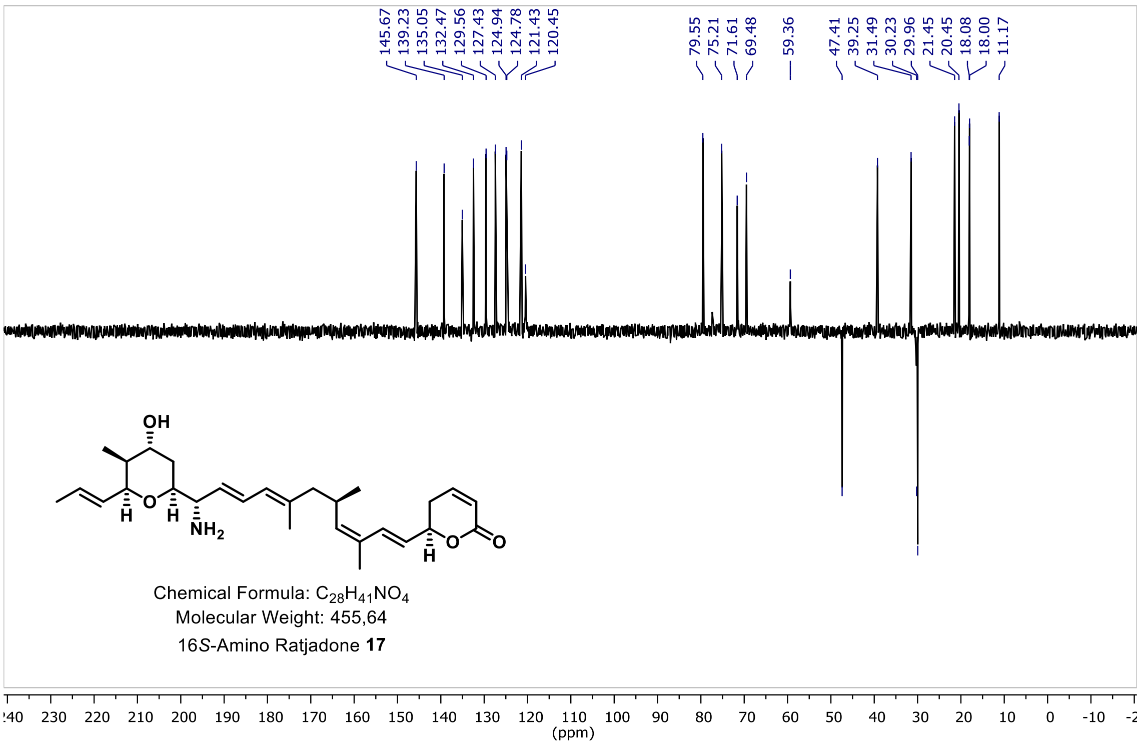
145.67
139.23
135.05
132.47
129.56
127.43
124.94
124.78
121.43
120.45
79.55
75.21
71.61
69.48
59.36
47.41
39.25
31.49
30.23
29.96
21.45
20.45
18.08
18.00
11.17
OH
H
O
H
NH2
H
O
O
Chemical Formula: $C_{28}H_{41}NO_4$
Molecular Weight: 455,64
16*S*-Amino Ratjadone **17**
(ppm)

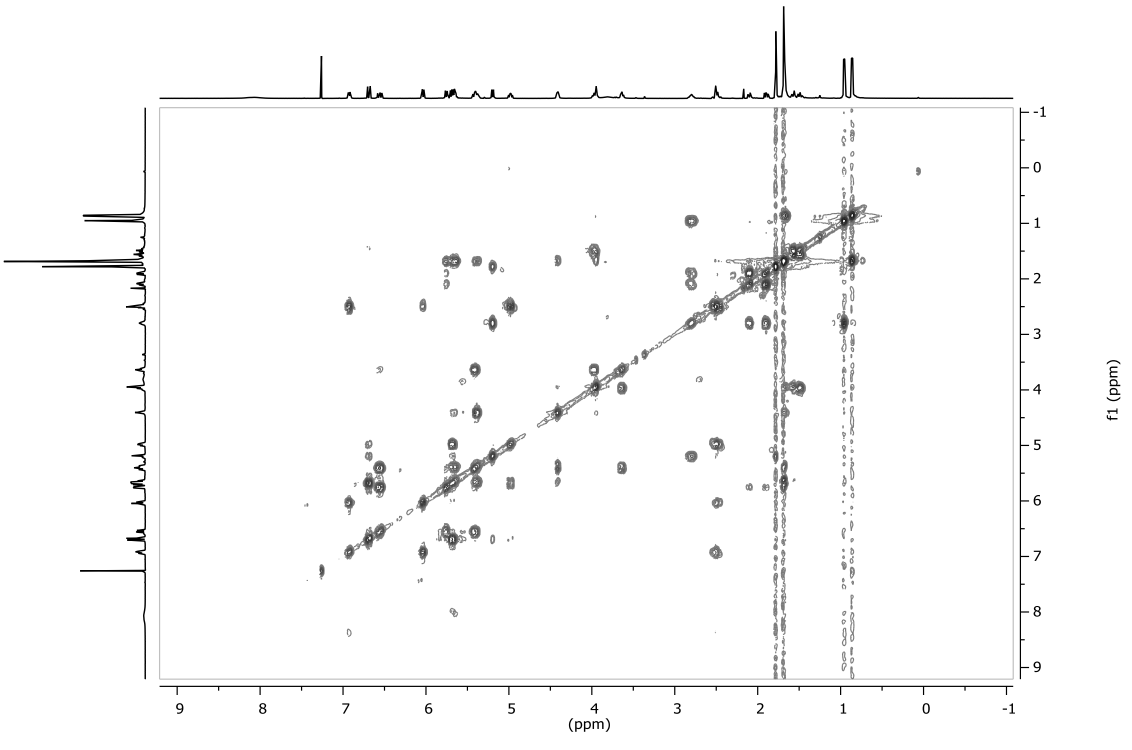
(ppm)
f1 (ppm)

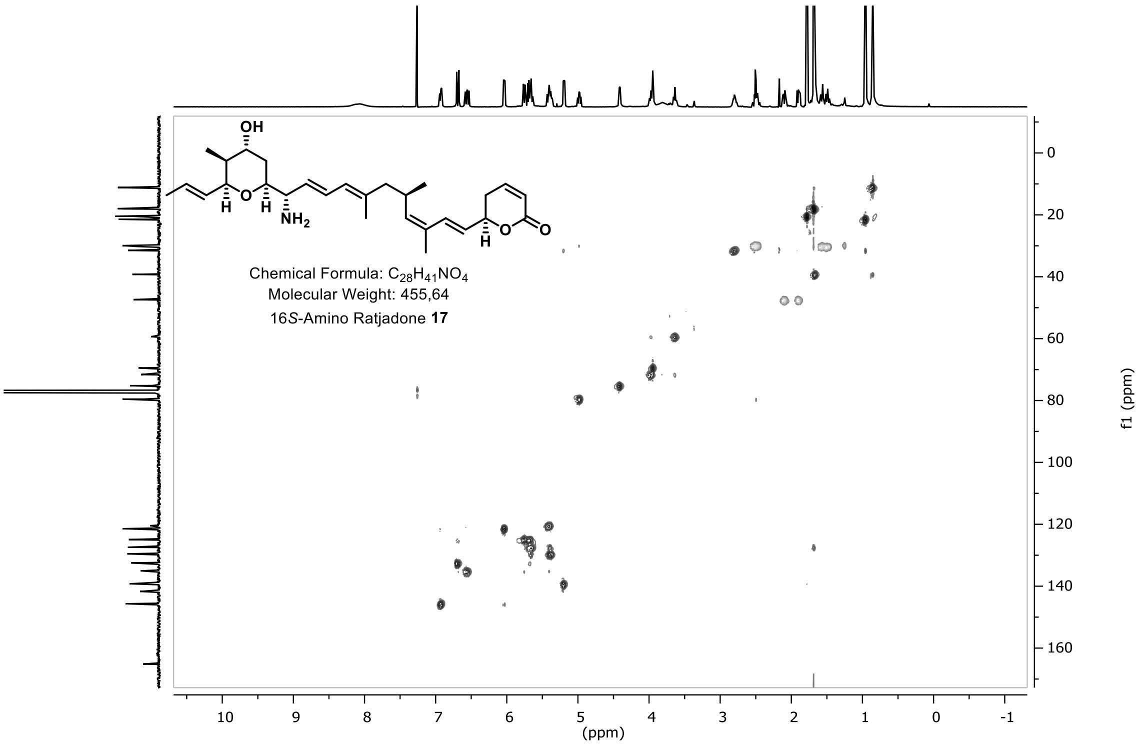
OH
O
H
H
NH2
H
O
O
Chemical Formula: C28H41NO4
Molecular Weight: 455,64
16S-Amino Ratjadone 17
f1 (ppm)
(ppm)

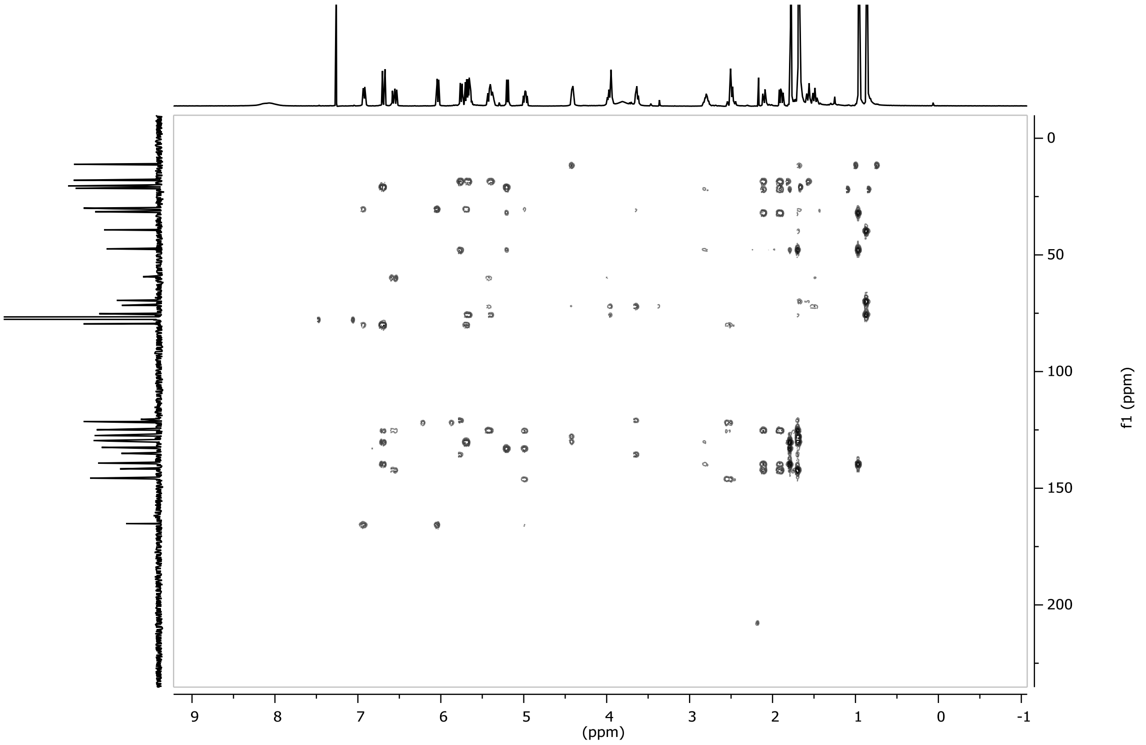
f1 (ppm)
(ppm)

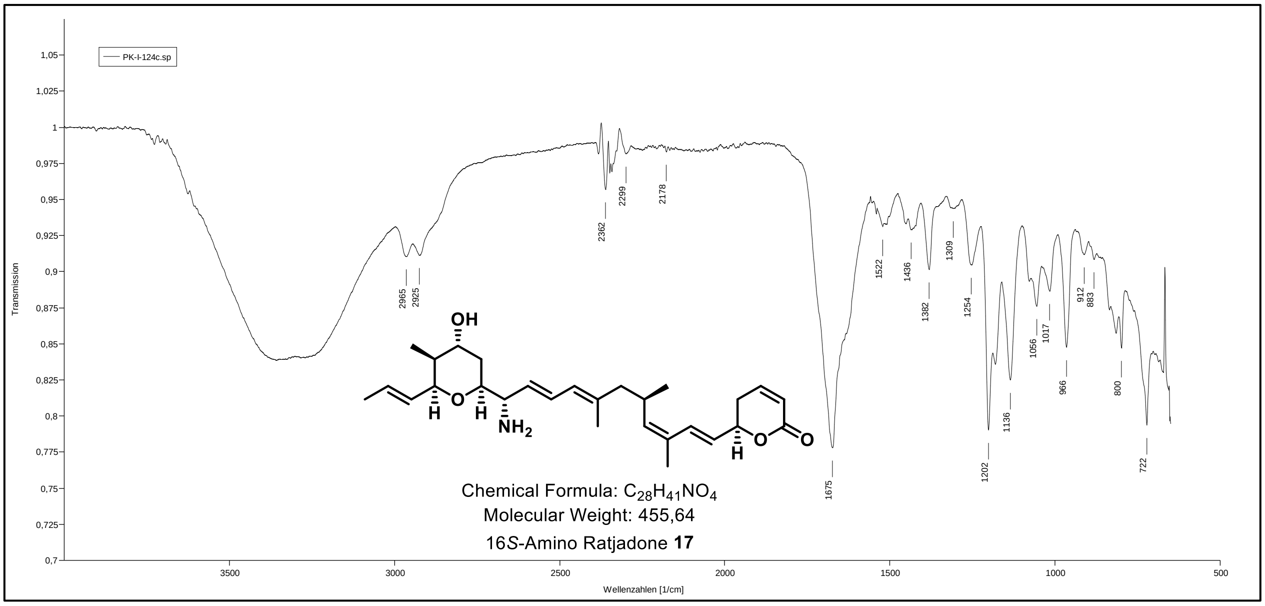

PK-I-124c.sp
Transmission
Wellenzahlen [1/cm]
1,05
1,025
1
0,975
0,95
0,925
0,9
0,875
0,85
0,825
0,8
0,775
0,75
0,725
0,7
3500
3000
2500
2000
1500
1000
500
2965
2925
2362
2299
2178
1675
1522
1436
1382
1309
1254
1202
1136
1056
1017
966
912
883
800
722
OH
O
H
H
NH2
O
H
O
Chemical Formula: C28H41NO4
Molecular Weight: 455,64
16S-Amino Ratjadone 17

Compound 18 - methyl (5R,6E,8Z,10R,12E,14E,16R)-3,16-diamino-5-hydroxy-16-((2S,4R,5S,6S)-4-hydroxy-5-methyl-6-((E)-prop-1-en-1-yl)tetrahydro-2H-pyran-2-yl)-8,10,12-trimethylhexadeca-6,8,12,14-tetraenoate

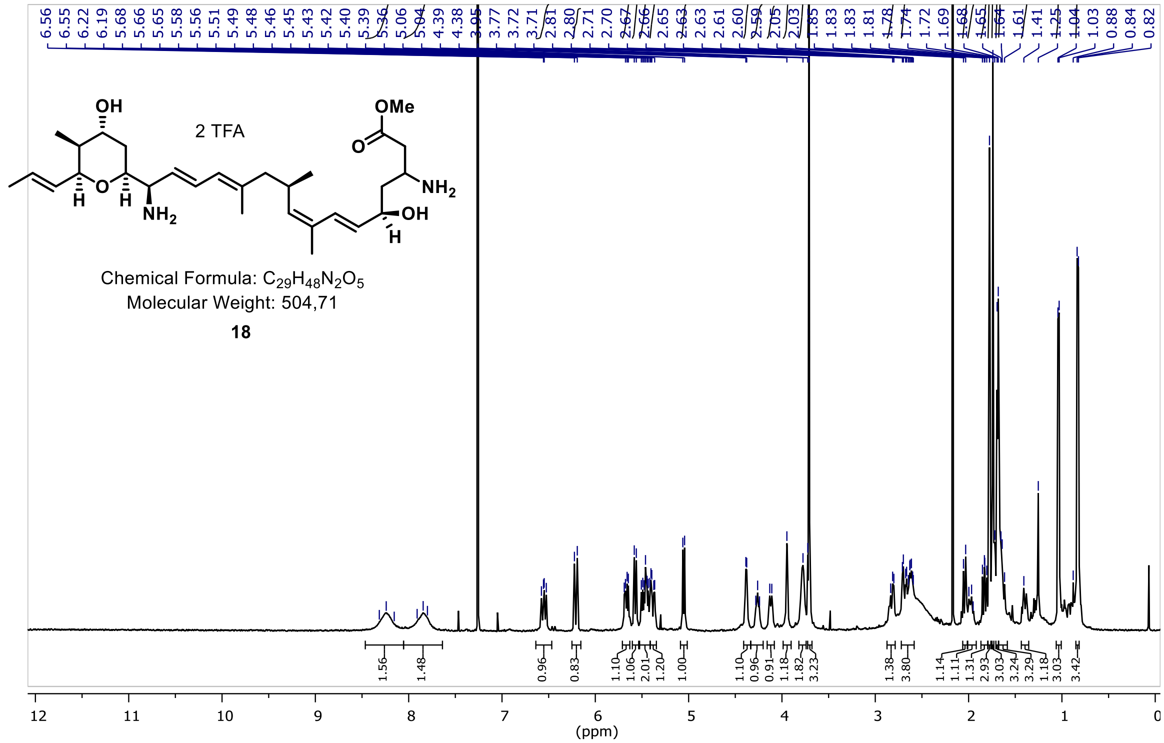

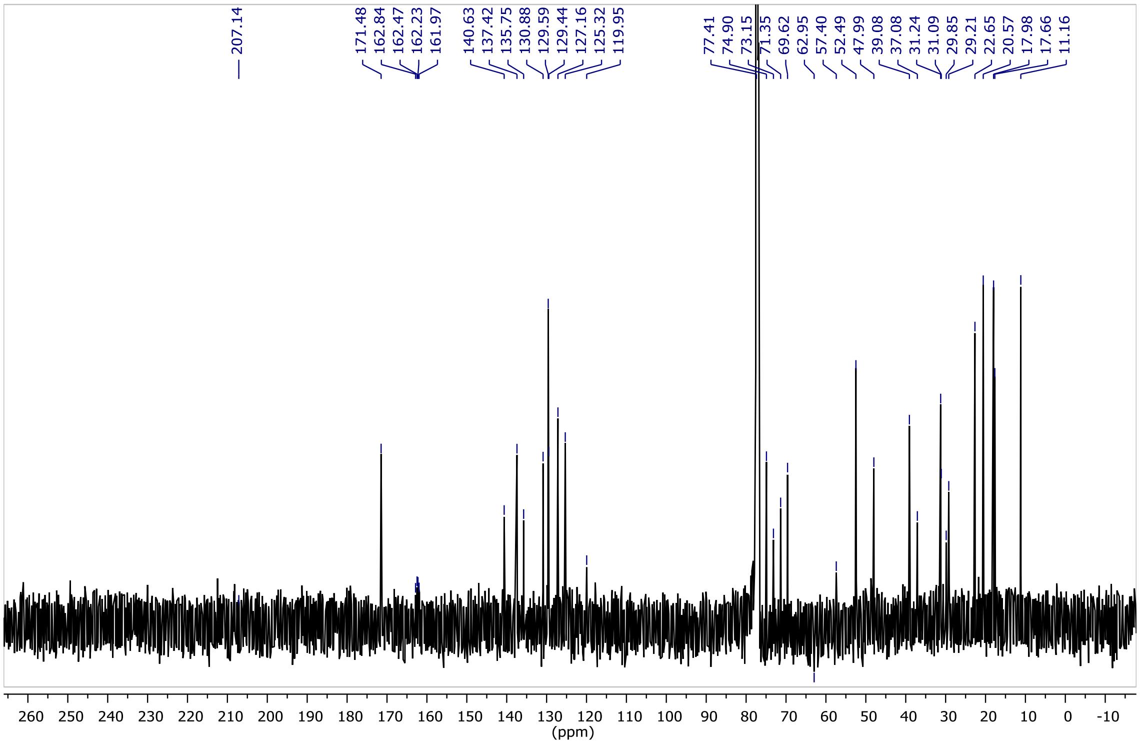

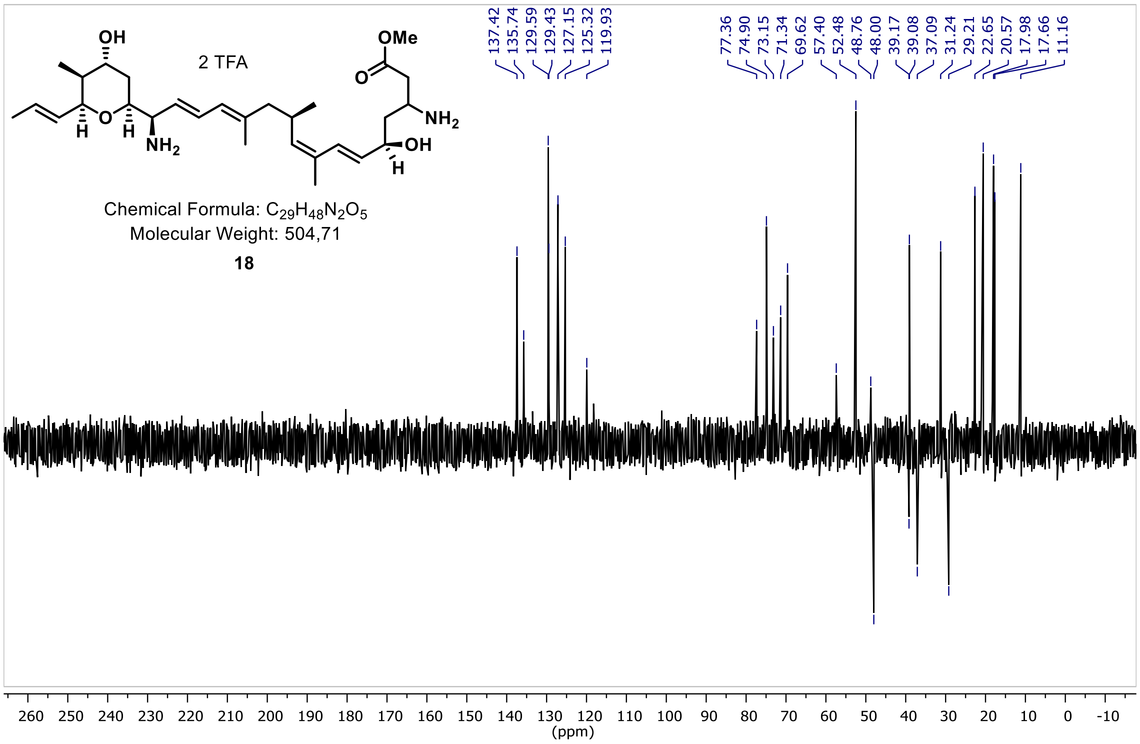
2 TFA
Chemical Formula: $C_{29}H_{48}N_2O_5$
Molecular Weight: 504,71
18
137.42
135.74
129.59
129.43
127.15
125.32
119.93
77.36
74.90
73.15
71.34
69.62
57.40
52.48
48.76
48.00
39.17
39.08
37.09
31.24
29.21
22.65
20.57
17.98
17.66
11.16
(ppm)

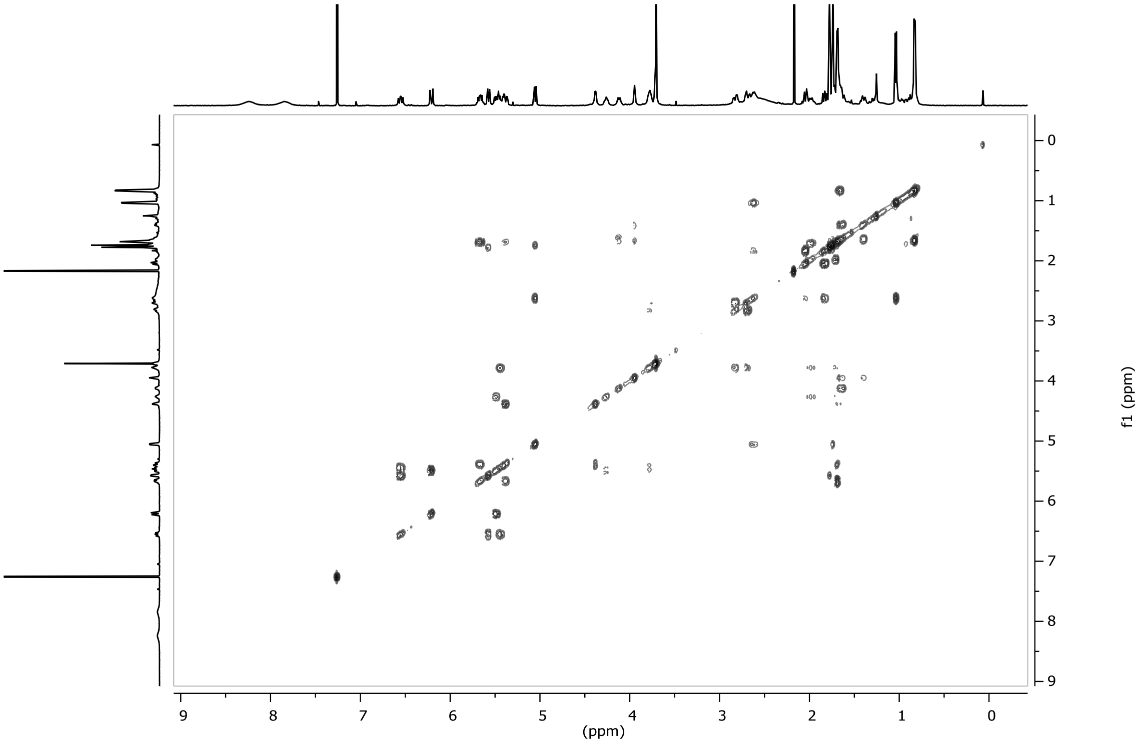
(ppm)
f1 (ppm)

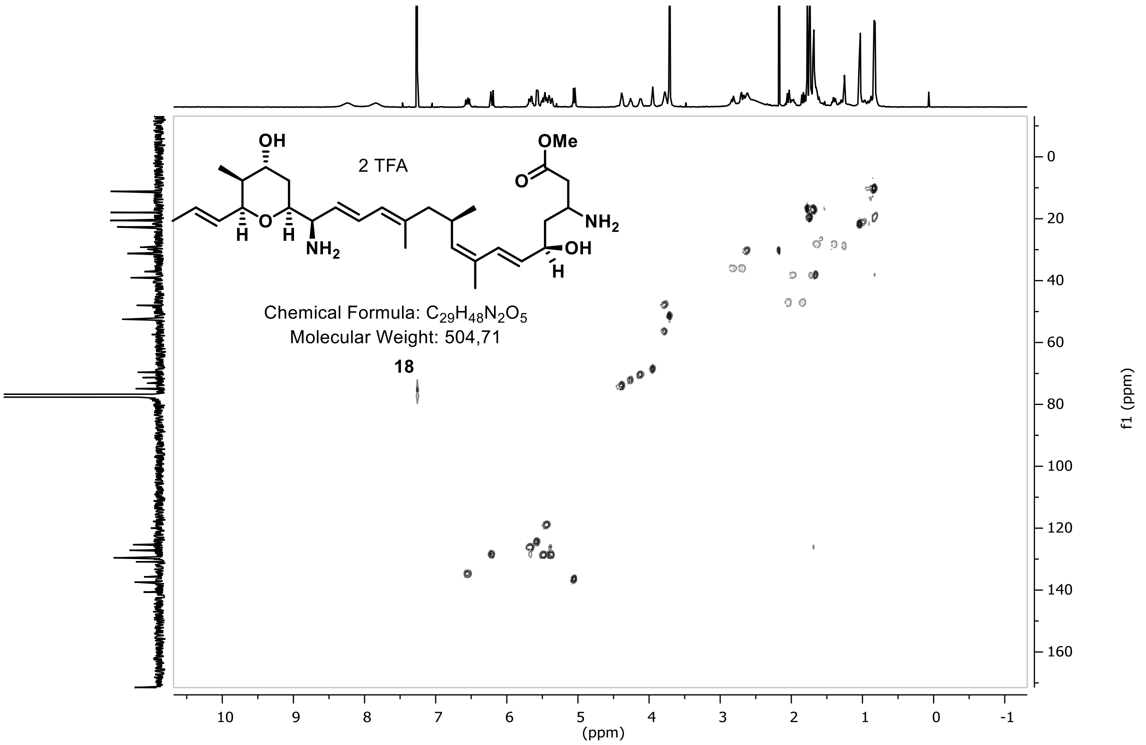
OH
OMe
2 TFA
O
O
NH2
H
H
NH2
OH
H
Chemical Formula: C29H48N2O5
Molecular Weight: 504,71
18
f1 (ppm)
(ppm)

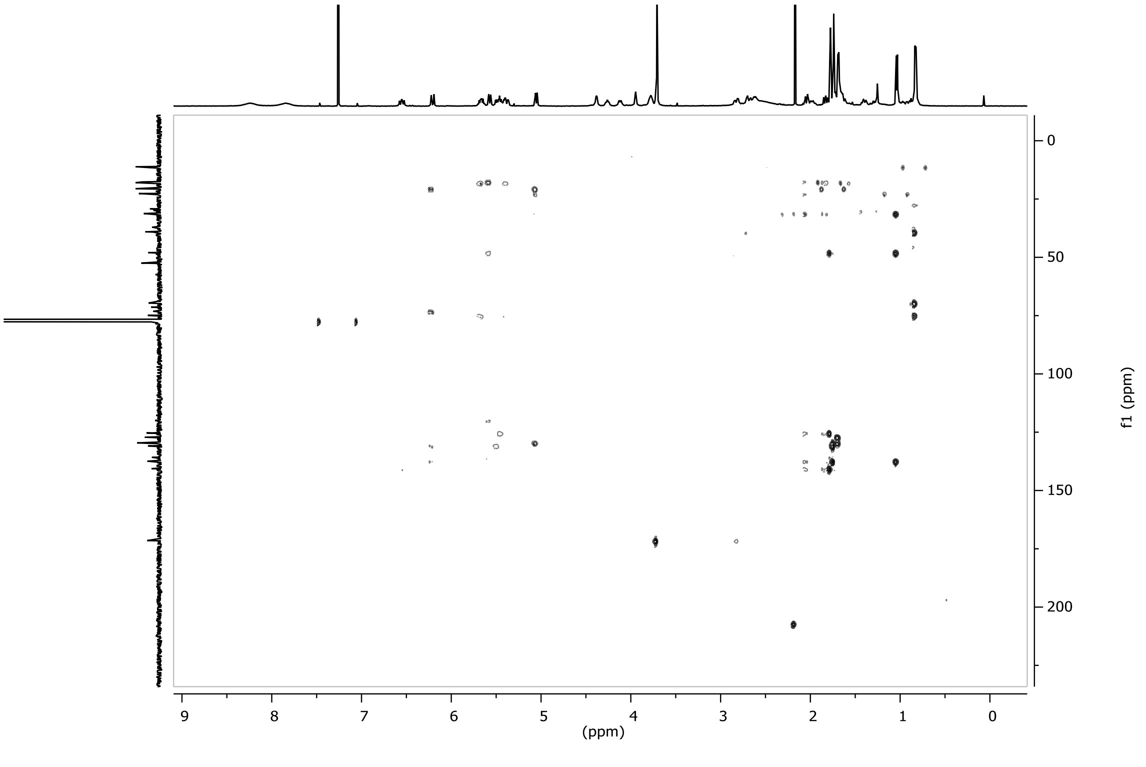
f1 (ppm)
(ppm)

Compound 19 – methyl (5R,6E,8Z,10R,12E,14E,16S)-3,16-diamino-5-hydroxy-16-((2S,4R,5S,6S)-4-hydroxy-5-methyl-6-((E)-prop-1-en-1-yl)tetrahydro-2H-pyran-2-yl)-8,10,12-trimethylhexadeca-6,8,12,14-tetraenoate

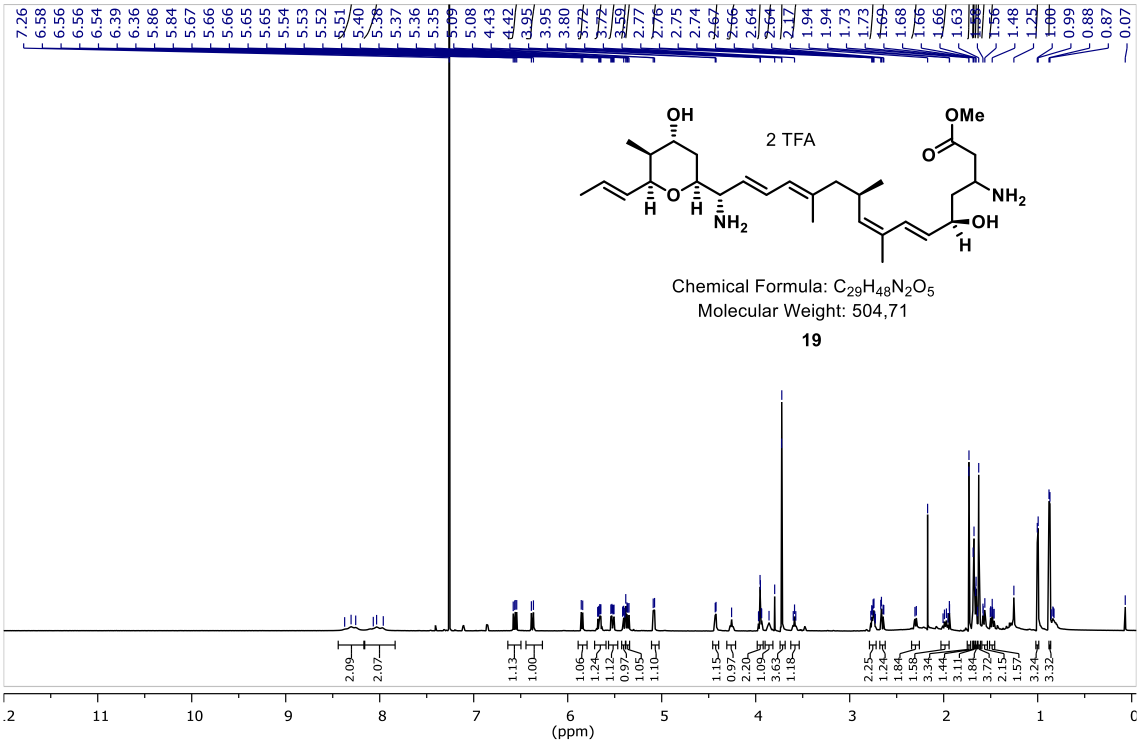

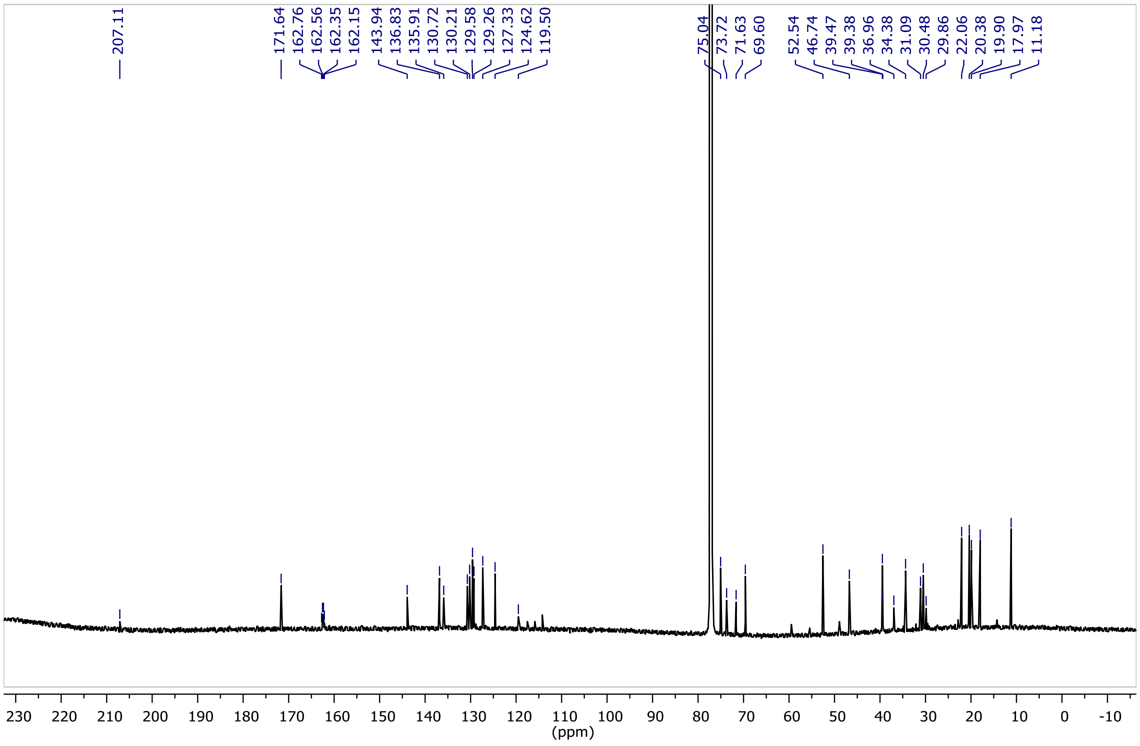

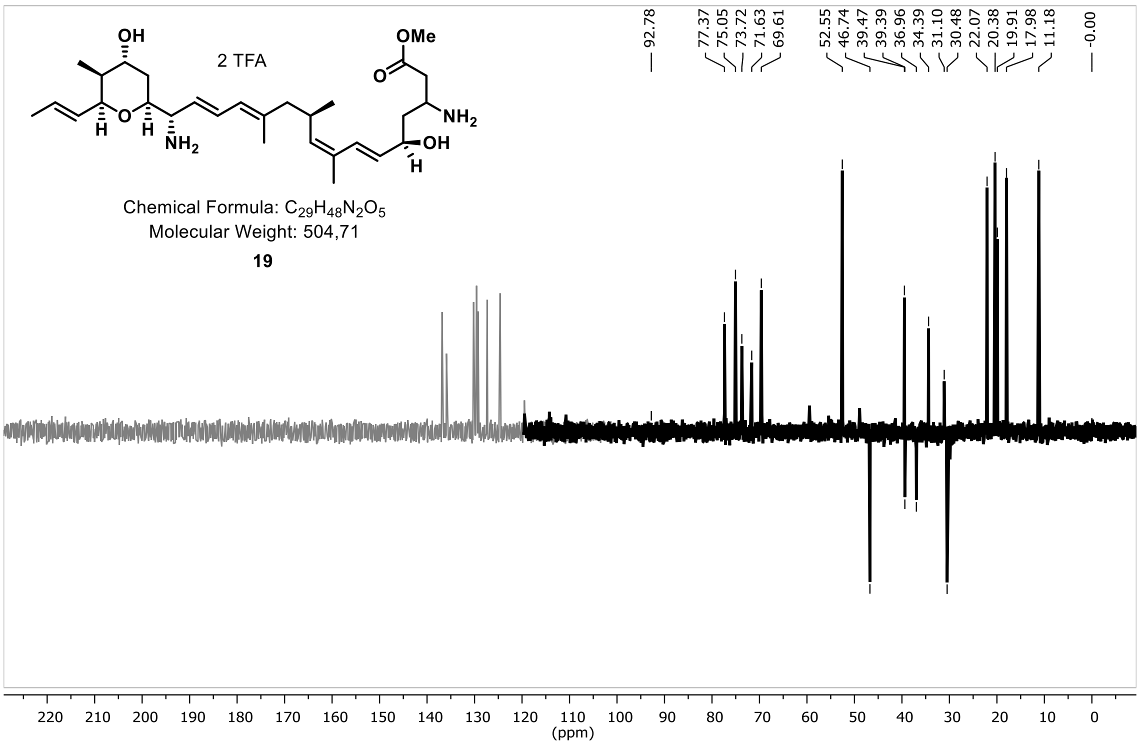
OH
2 TFA
OMe
O
O
H
H
NH2
NH2
OH
H
Chemical Formula: C29H48N2O5
Molecular Weight: 504,71
19
92.78
77.37
75.05
73.72
71.63
69.61
52.55
46.74
39.47
39.39
36.96
34.39
31.10
30.48
22.07
20.38
19.91
17.98
11.18
-0.00
(ppm)

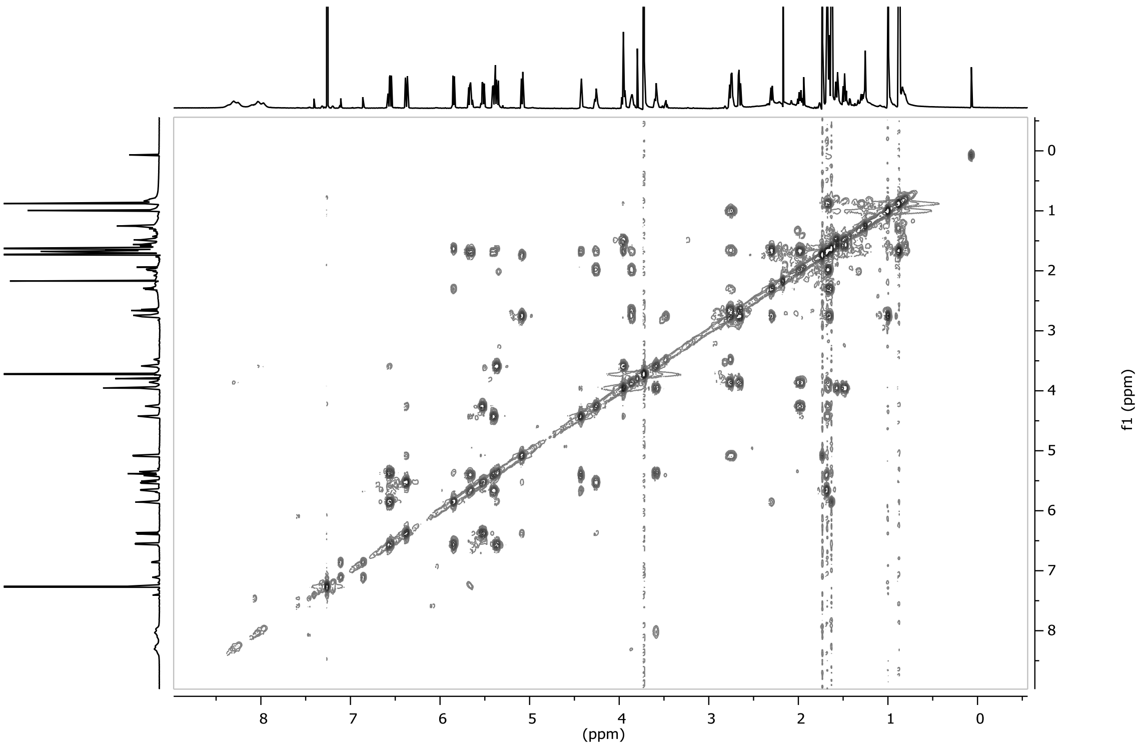
(ppm)
f1 (ppm)

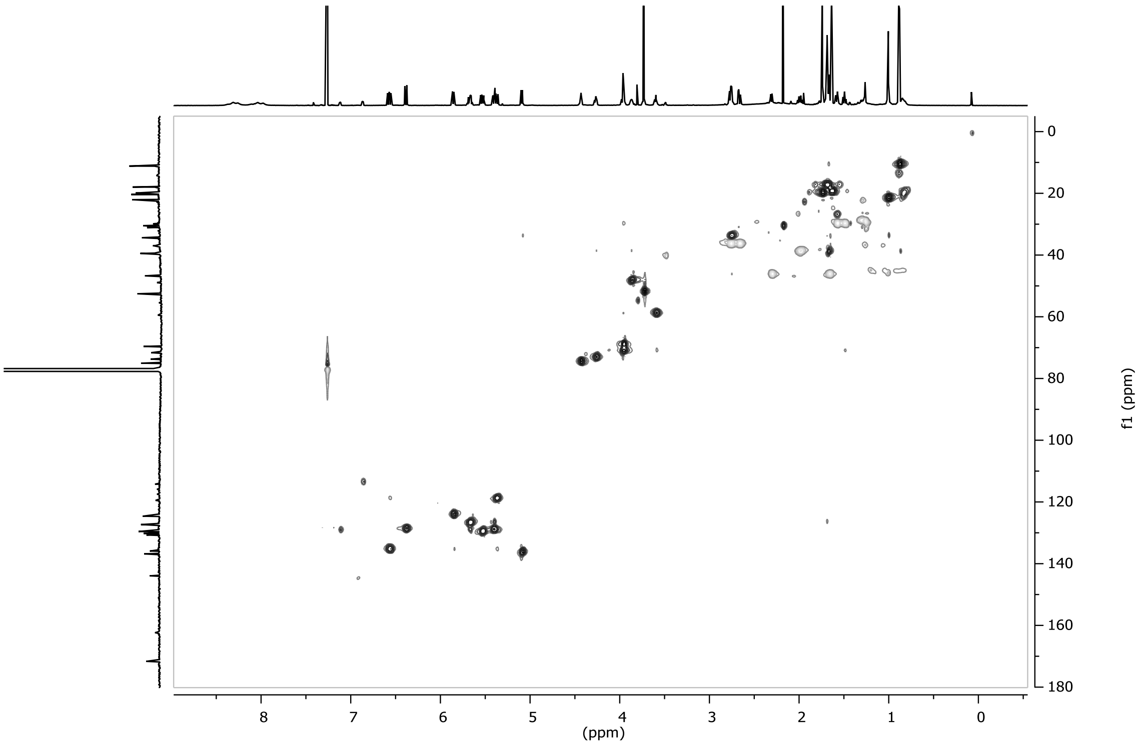
f1 (ppm)
(ppm)

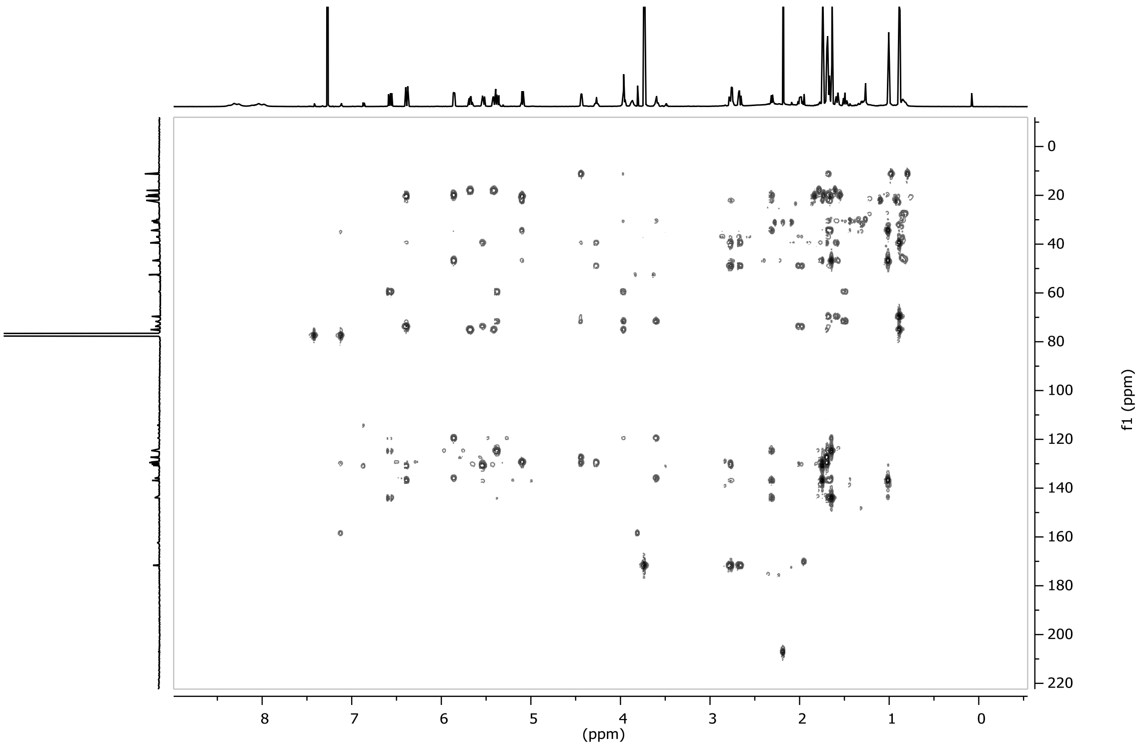
f1 (ppm)
(ppm)

16-*R*-Mosher - (R)-3,3,3-trifluoro-N-((1R,2E,4E,7R,8Z,10E)-1-((2S,4R,5S,6S)-4-hydroxy-5-methyl-6-((E)-prop-1-en-1-yl)tetrahydro-2H-pyran-2-yl)-5,7,9-trimethyl-11-((R)-6-oxo-3,6-dihydro-2H-pyran-2-yl)undeca-2,4,8,10-tetraen-1-yl)-2-methoxy-2-phenylpropanamide

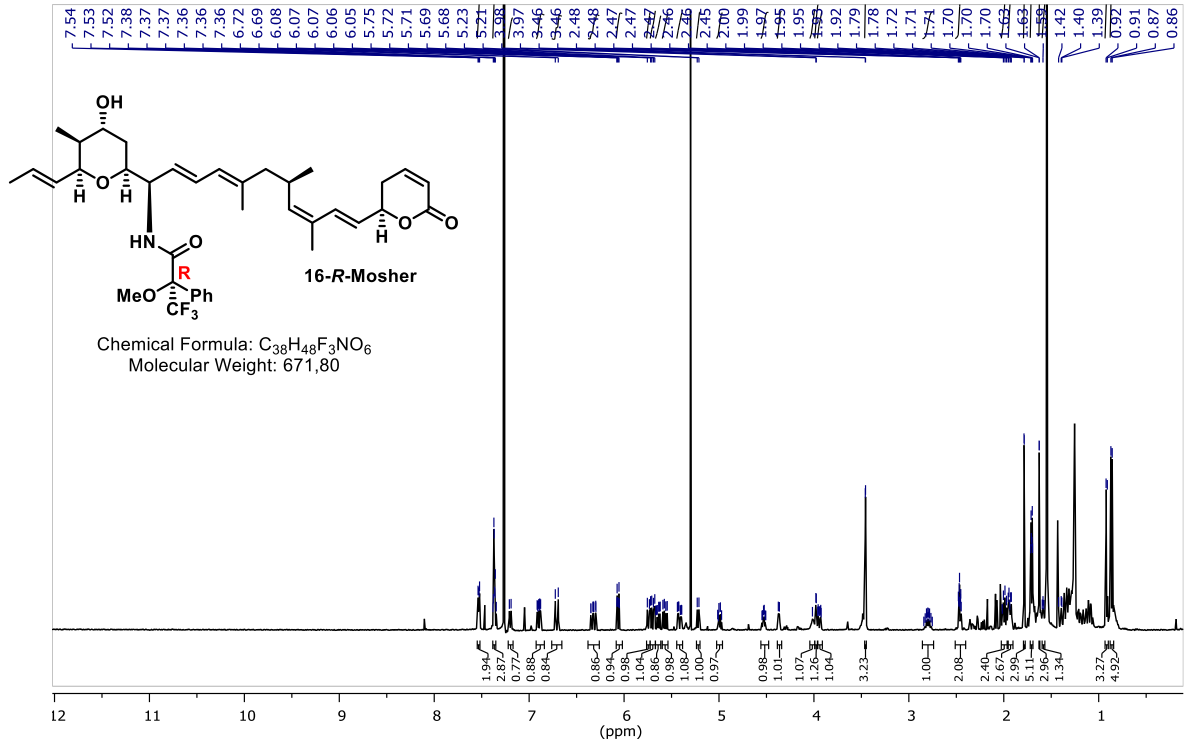

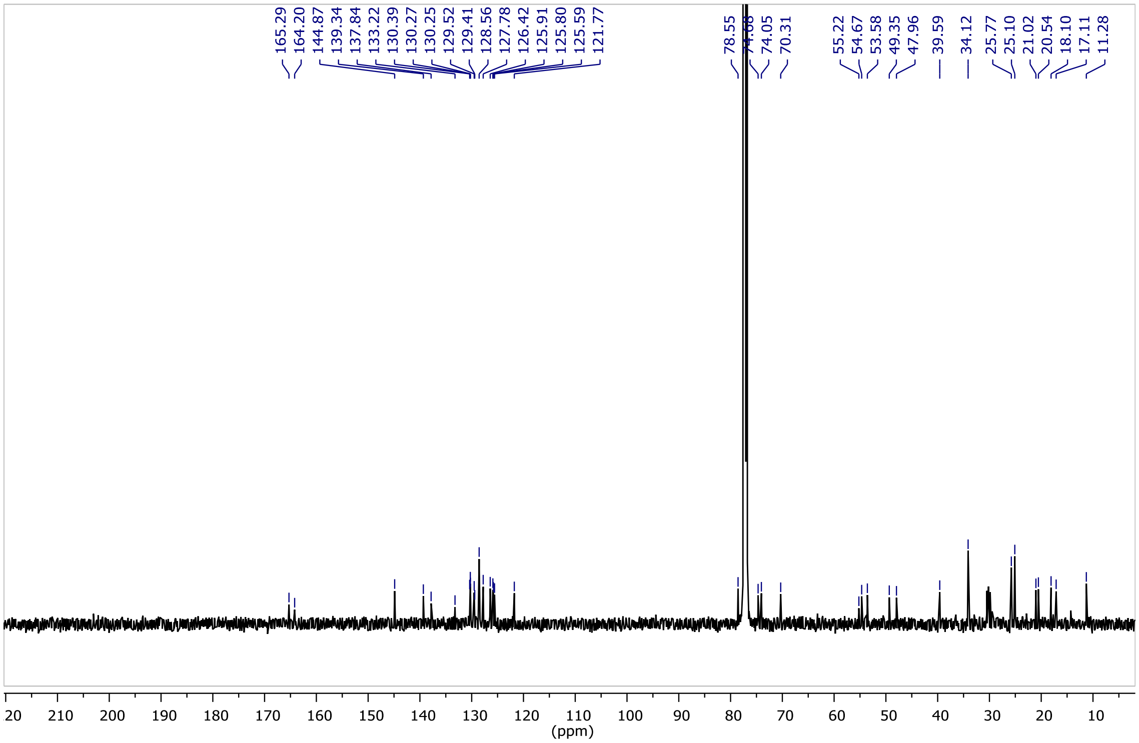

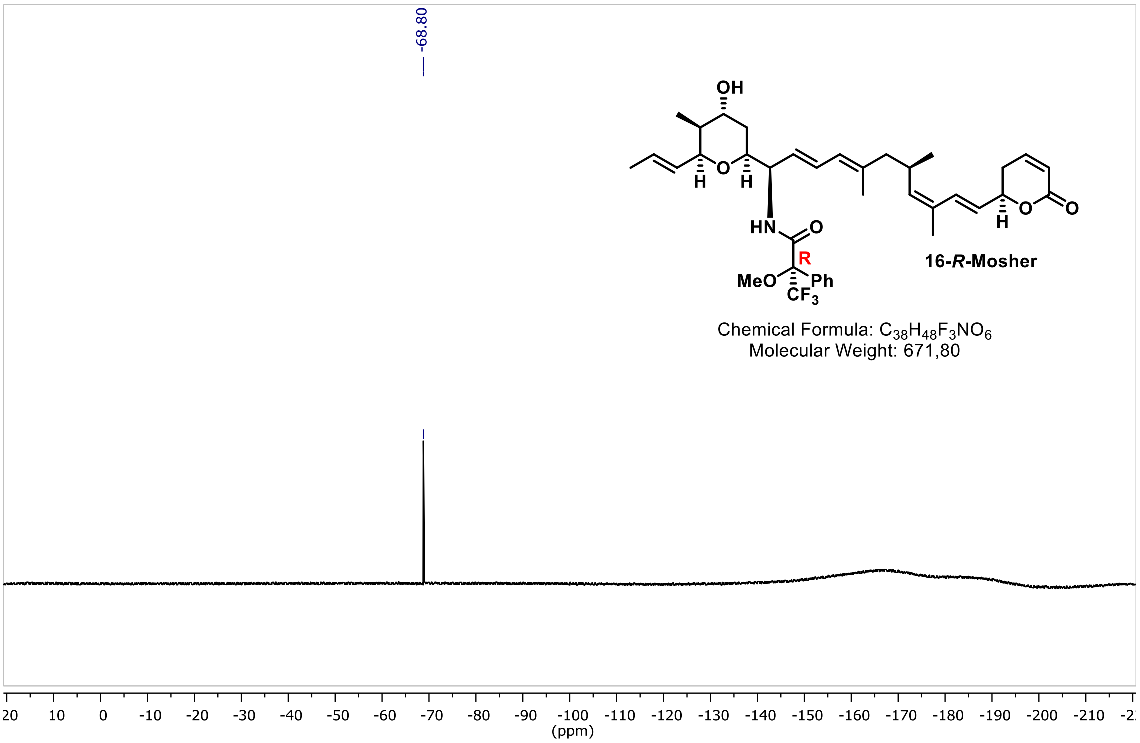
-68.80
OH
O
H
H
HN
O
R
MeO
Ph
CF3
O
O
H
16-R-Mosher
Chemical Formula: C38H48F3NO6
Molecular Weight: 671,80
20
10
0
-10
-20
-30
-40
-50
-60
-70
-80
-90
-100
-110
-120
-130
-140
-150
-160
-170
-180
-190
-200
-210
(ppm)

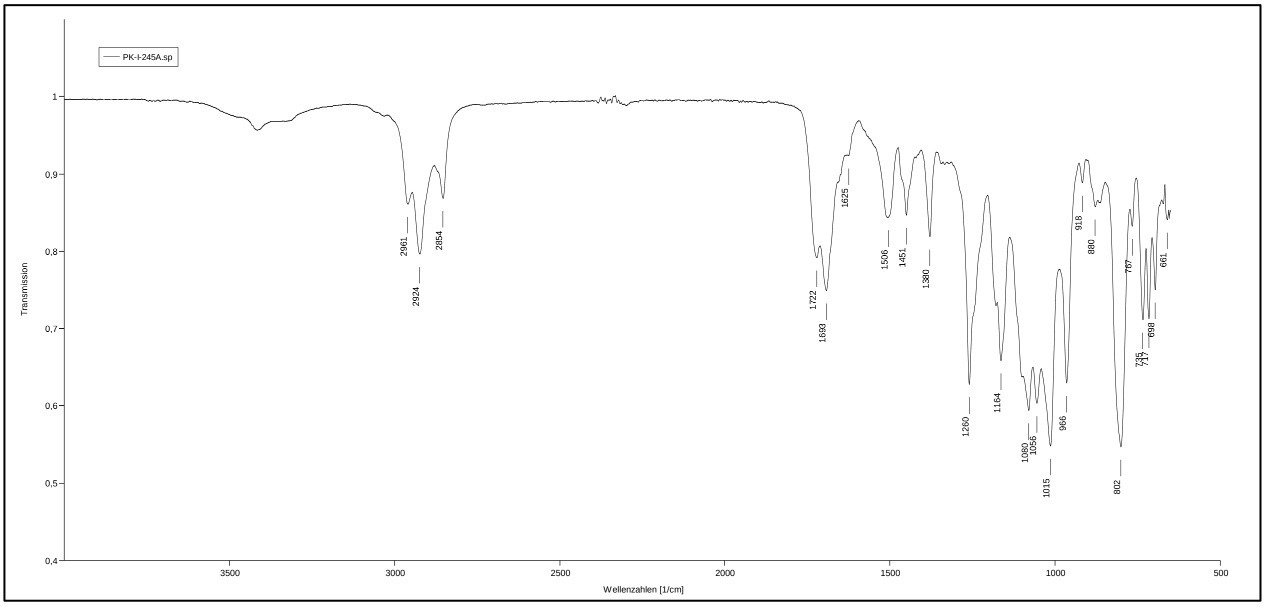
PK-I-245A.sp
Transmission
Wellenzahlen [1/cm]
1
0,9
0,8
0,7
0,6
0,5
0,4
3500
3000
2500
2000
1500
1000
500
2961
2924
2854
1722
1693
1625
1506
1451
1380
1260
1164
1080
1056
1015
966
918
880
802
767
735
717
698
661

17-*R*-Mosher - (S)-3,3,3-trifluoro-N-((1R,2E,4E,7R,8Z,10E)-1-((2S,4R,5S,6S)-4-hydroxy-5-methyl-6-((E)-prop-1-en-1-yl)tetrahydro-2H-pyran-2-yl)-5,7,9-trimethyl-11-((R)-6-oxo-3,6-dihydro-2H-pyran-2-yl)undeca-2,4,8,10-tetraen-1-yl)-2-methoxy-2-phenylpropanamide

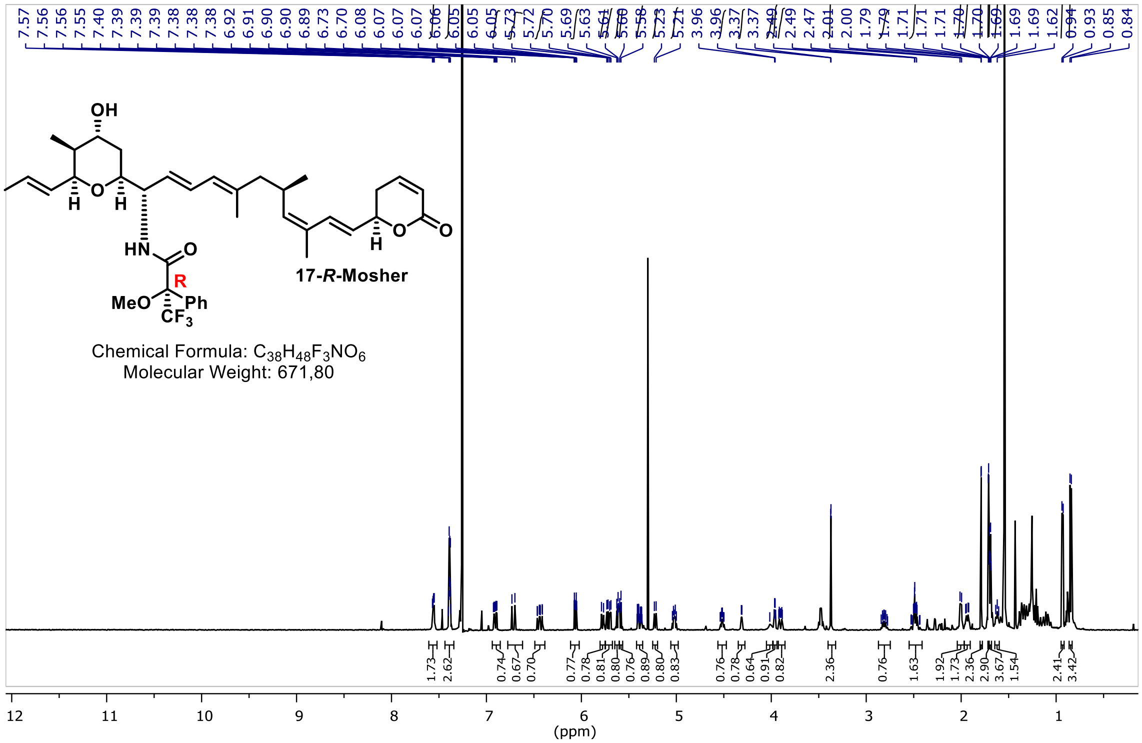

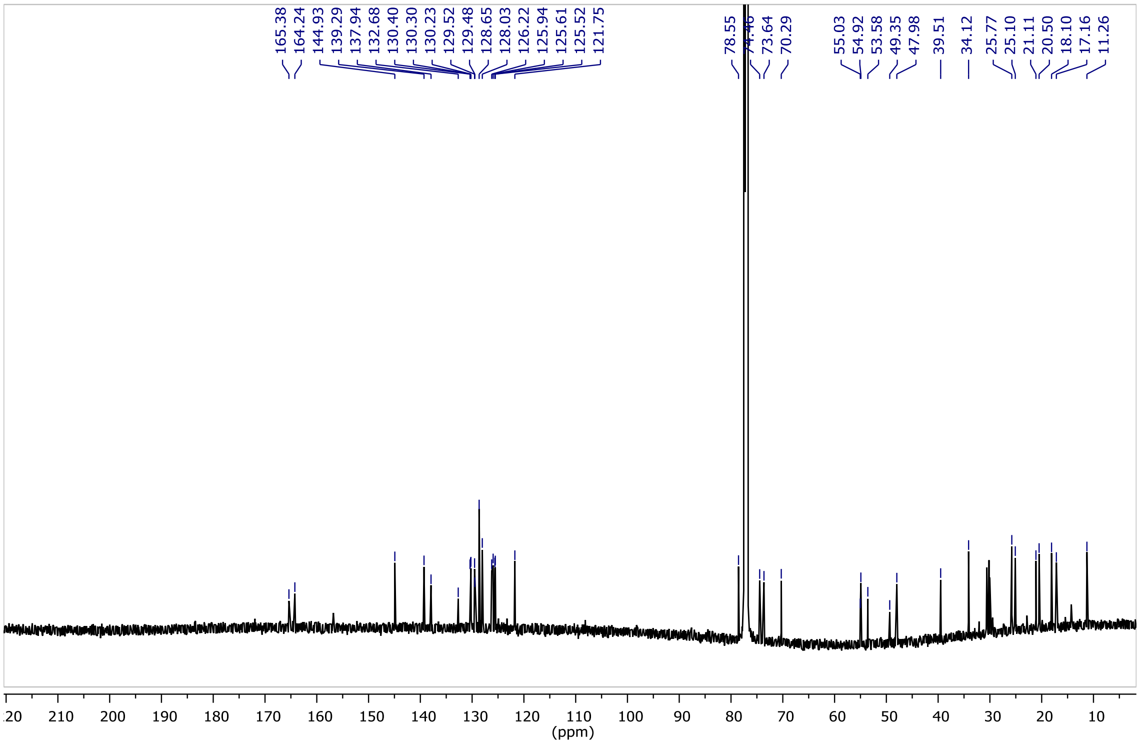

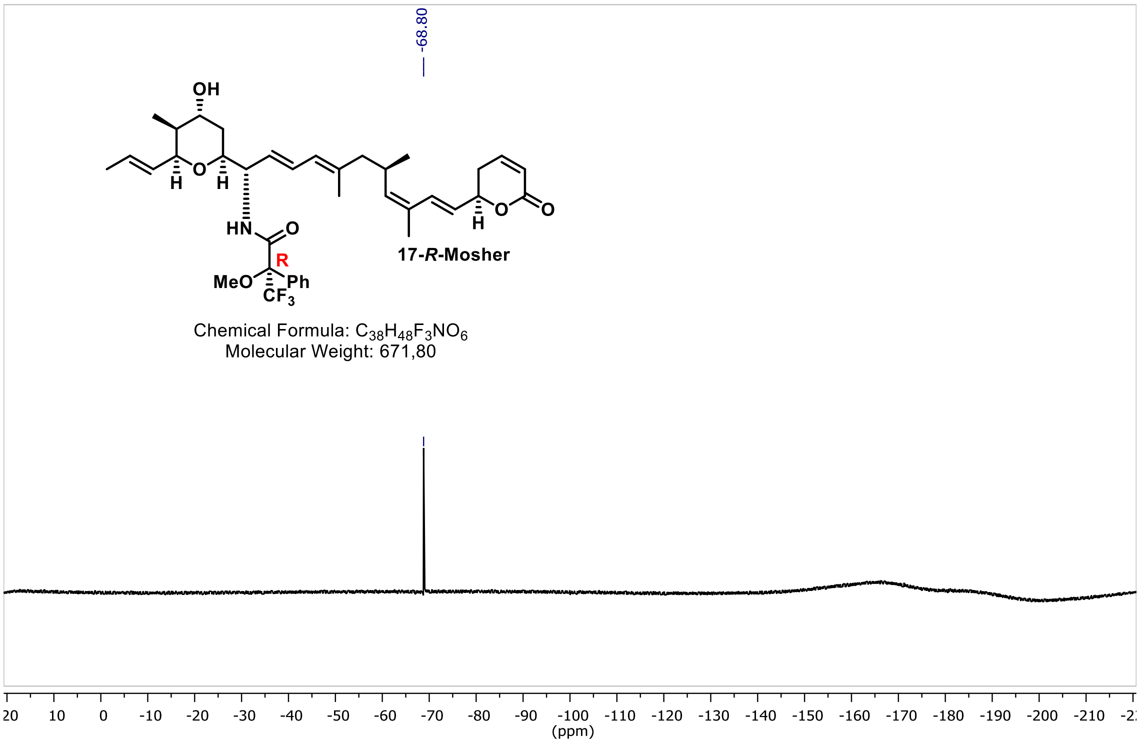
-68.80
OH
HN
O
R
MeO
Ph
CF3
17-R-Mosher
Chemical Formula: C38H48F3NO6
Molecular Weight: 671,80
(ppm)

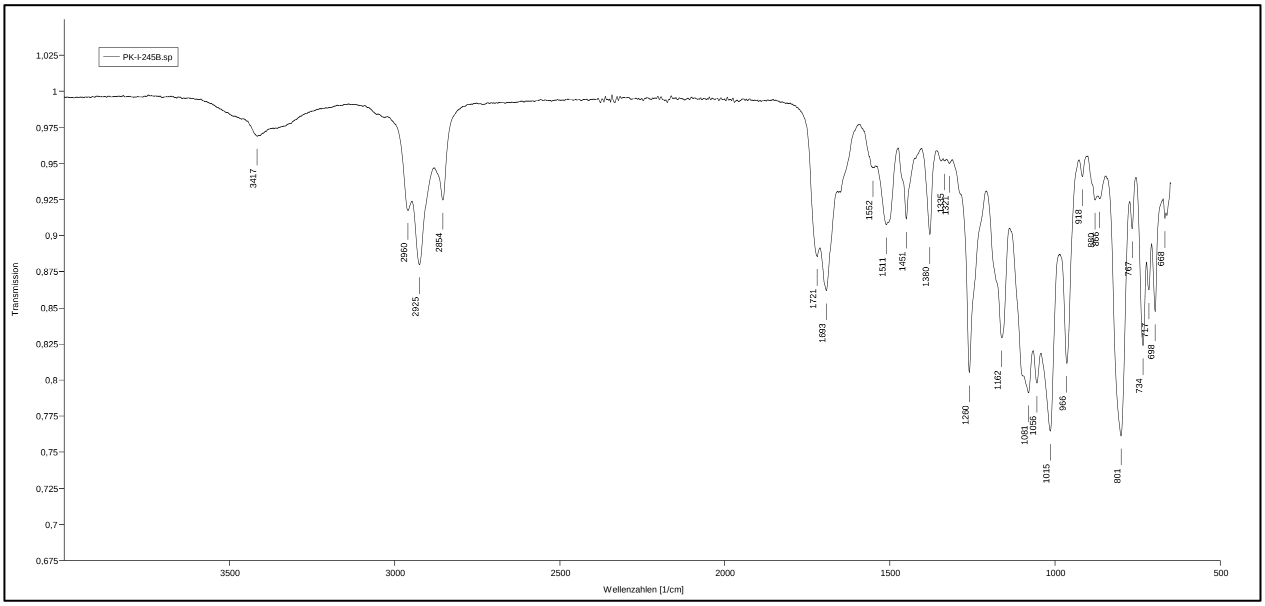
PK-I-245B.sp
Transmission
Wellenzahlen [1/cm]
3417
2960
2925
2854
1721
1693
1552
1511
1451
1380
1335
1321
1260
1162
1081
1056
1015
966
918
880
865
801
767
734
717
698
668

16-*S*-Mosher - (R)-3,3,3-trifluoro-N-((1S,2E,4E,7R,8Z,10E)-1-((2S,4R,5S,6S)-4-hydroxy-5-methyl-6-((E)-prop-1-en-1-yl)tetrahydro-2H-pyran-2-yl)-5,7,9-trimethyl-11-((R)-6-oxo-3,6-dihydro-2H-pyran-2-yl)undeca-2,4,8,10-tetraen-1-yl)-2-methoxy-2-phenylpropanamide

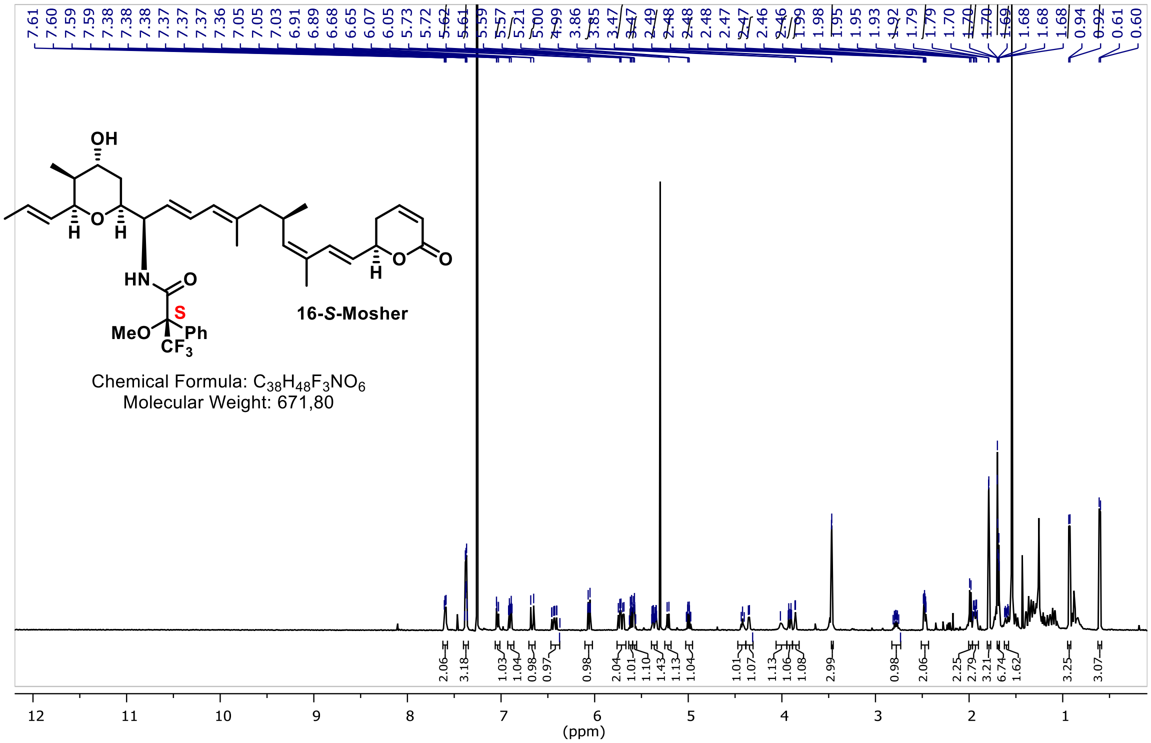

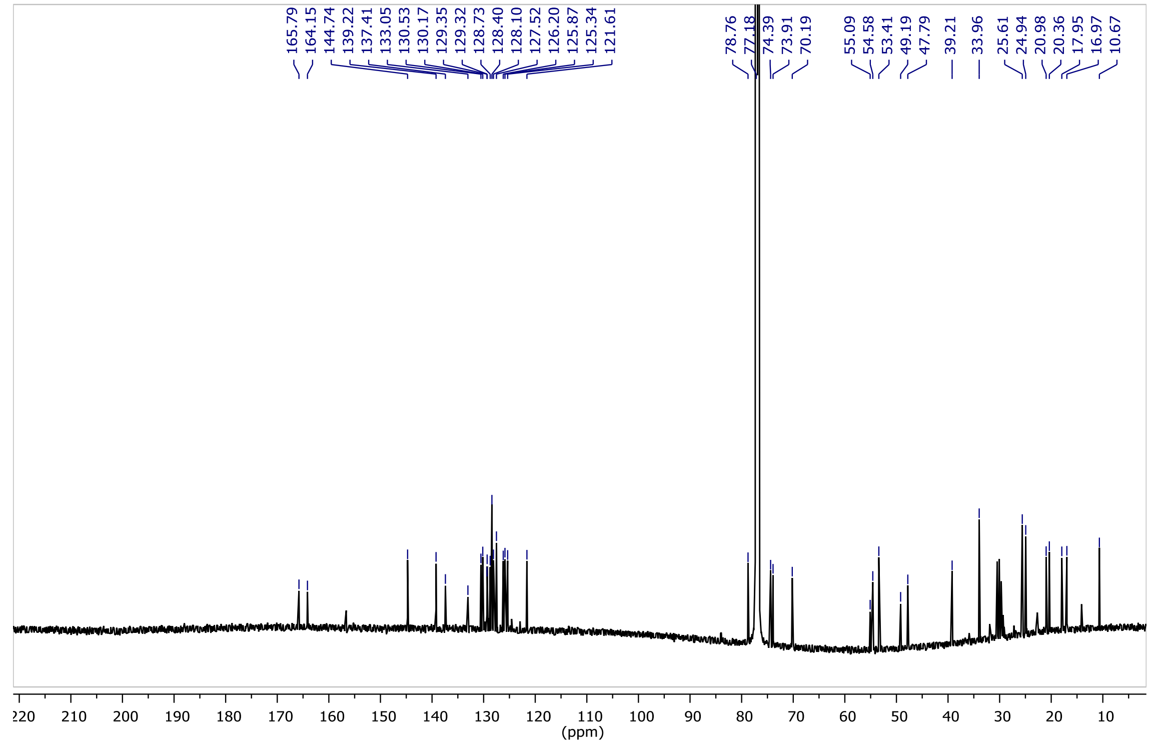

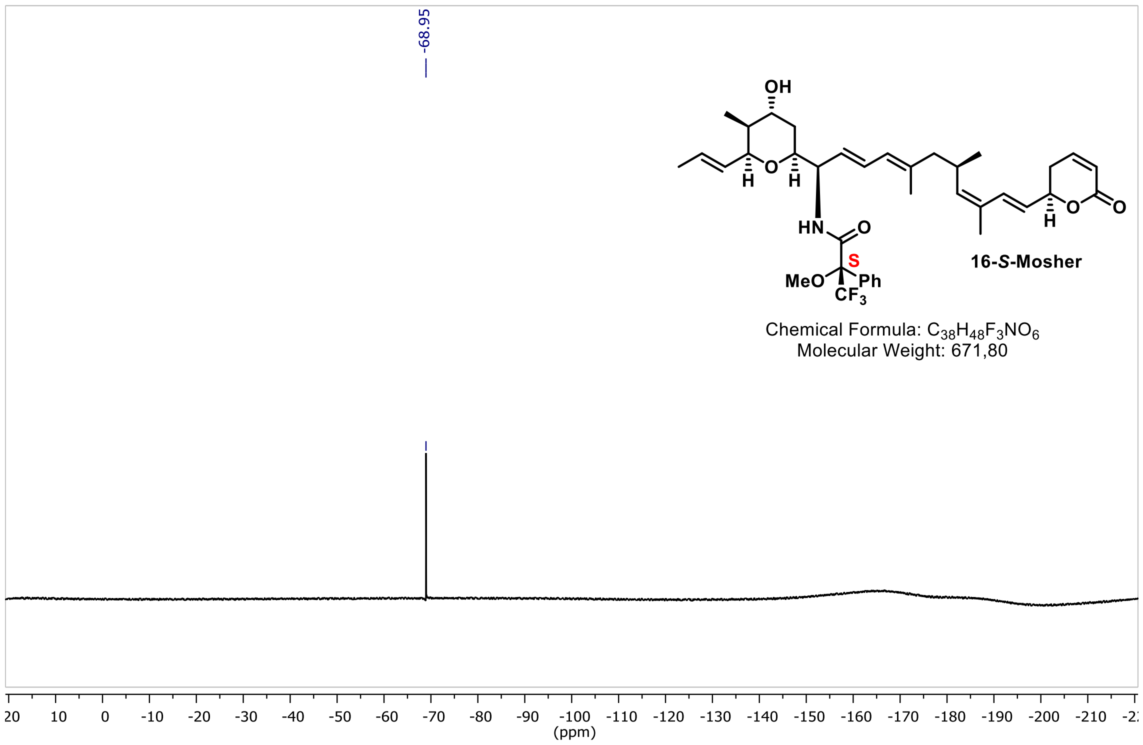
-68.95
OH
O
H
H
HN
O
S
MeO
Ph
CF3
O
O
H
16-S-Mosher
Chemical Formula: C38H48F3NO6
Molecular Weight: 671,80
20
10
0
-10
-20
-30
-40
-50
-60
-70
-80
-90
-100
-110
-120
-130
-140
-150
-160
-170
-180
-190
-200
-210
(ppm)

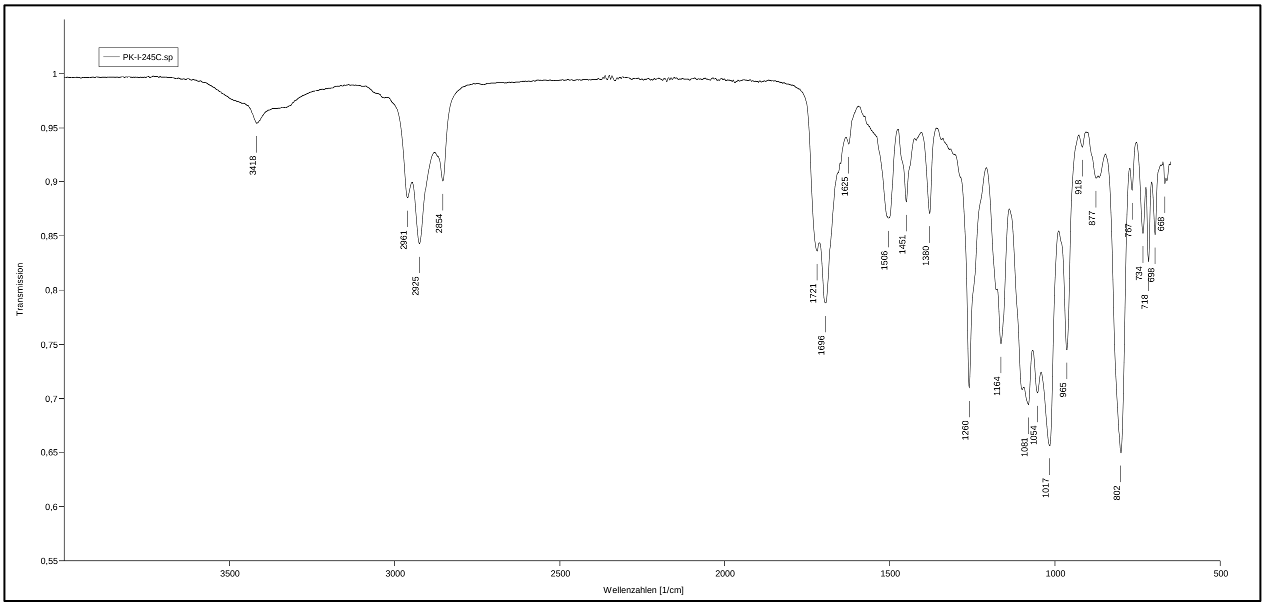
PK-I-245C.sp
Transmission
Wellenzahlen [1/cm]
3418
2961
2925
2854
1721
1696
1625
1506
1451
1380
1260
1164
1081
1054
1017
965
918
877
802
767
734
718
698
668

17-*S*-Mosher - (S)-3,3,3-trifluoro-N-((1S,2E,4E,7R,8Z,10E)-1-((2S,4R,5S,6S)-4-hydroxy-5-methyl-6-((E)-prop-1-en-1-yl)tetrahydro-2H-pyran-2-yl)-5,7,9-trimethyl-11-((R)-6-oxo-3,6-dihydro-2H-pyran-2-yl)undeca-2,4,8,10-tetraen-1-yl)-2-methoxy-2-phenylpropanamid

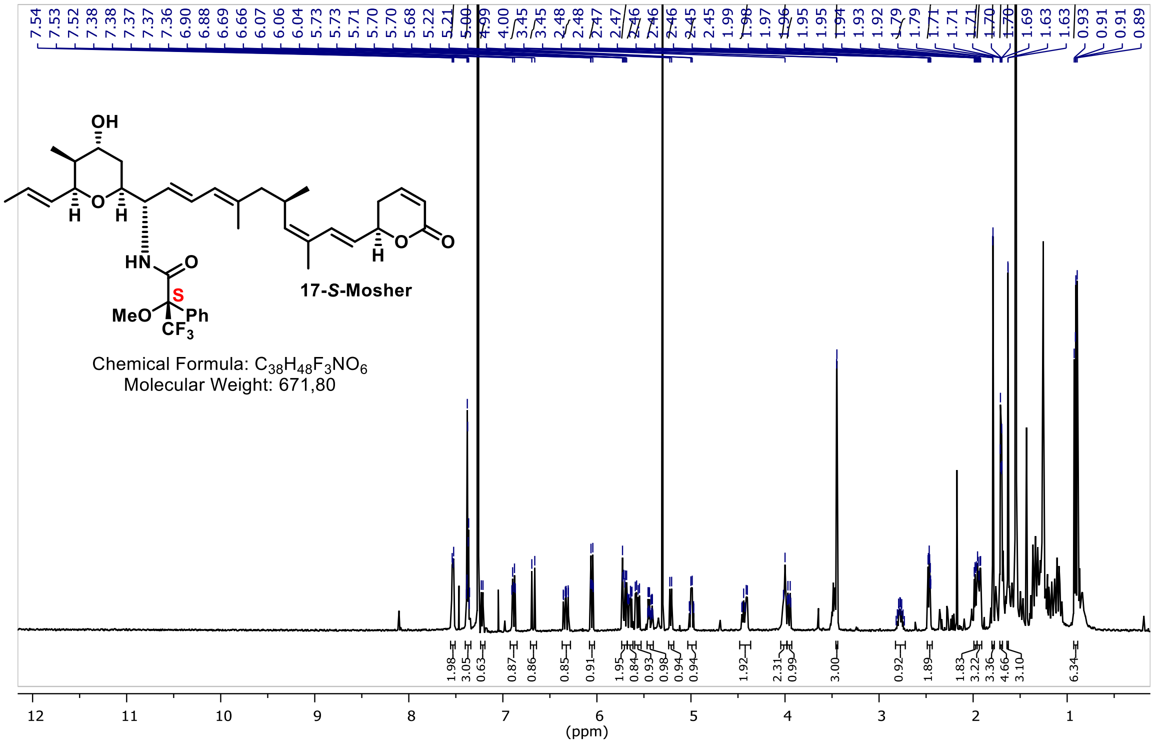

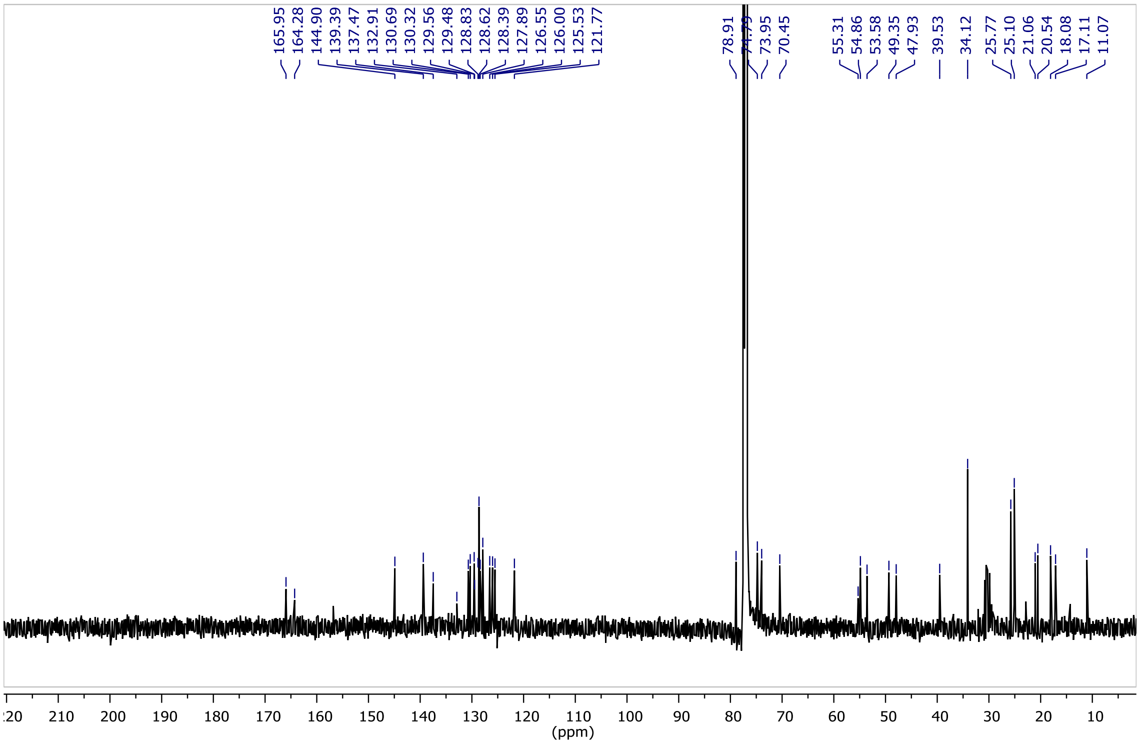

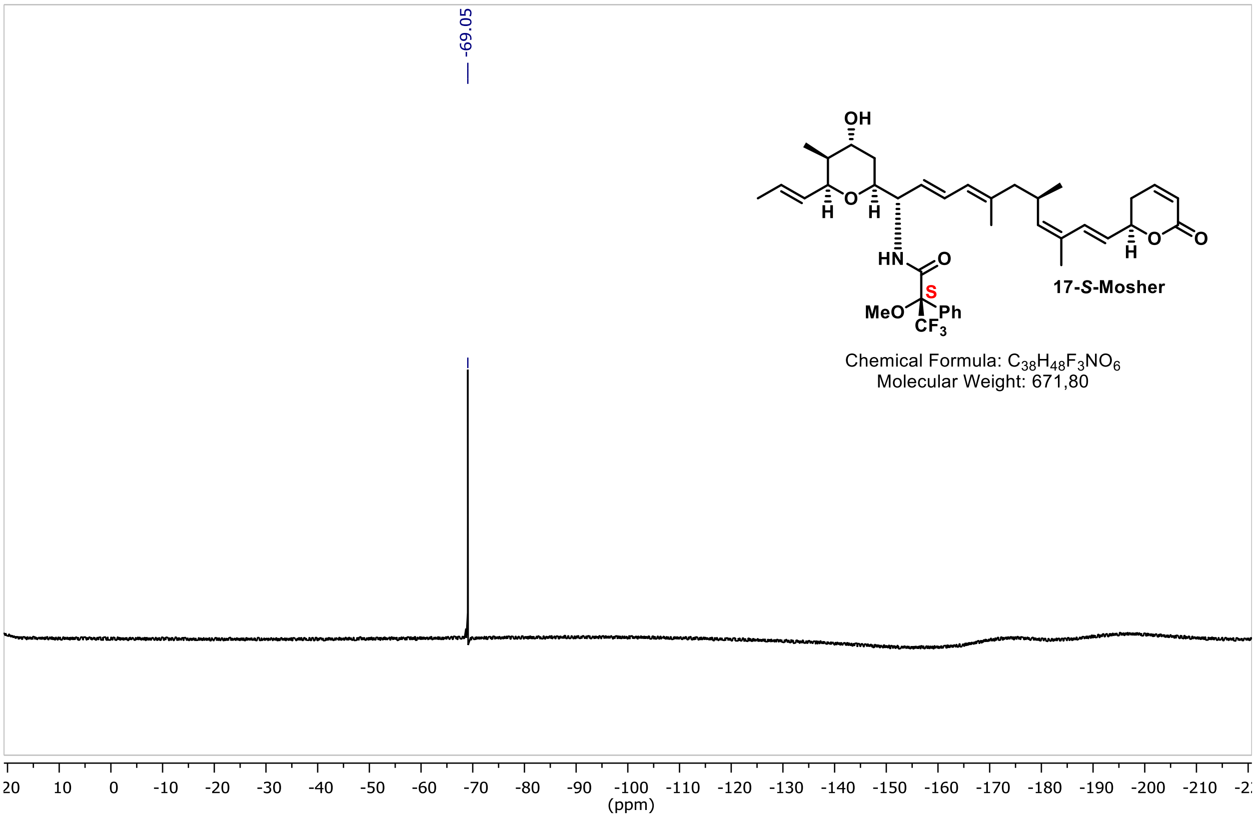
-69.05
17-S-Mosher
Chemical Formula: $C_{38}H_{48}F_3NO_6$
Molecular Weight: 671,80
20 10 0 -10 -20 -30 -40 -50 -60 -70 -80 -90 -100 -110 -120 -130 -140 -150 -160 -170 -180 -190 -200 -210
(ppm)

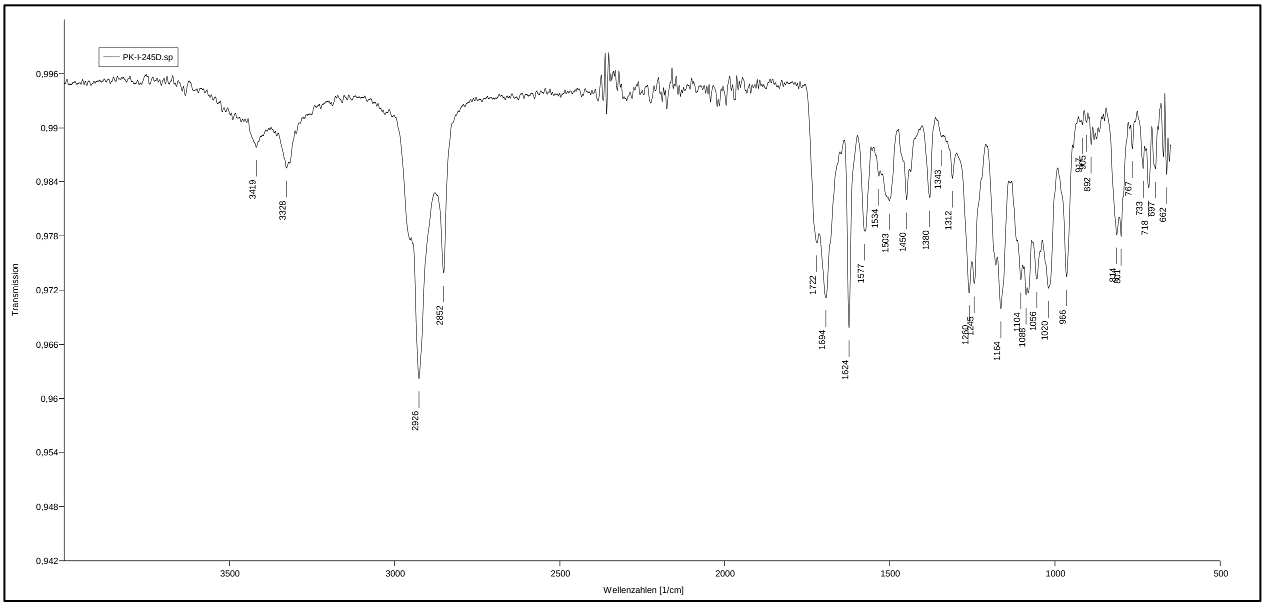
PK-I-245D.sp
Transmission
Wellenzahlen [1/cm]
3419
3328
2926
2852
1722
1694
1624
1577
1534
1503
1450
1380
1343
1312
1260
1245
1164
1104
1088
1056
1020
966
917
905
892
814
801
767
733
718
697
662

Pent-4-ynoic acid N-hydroxysuccinimid ester (23)

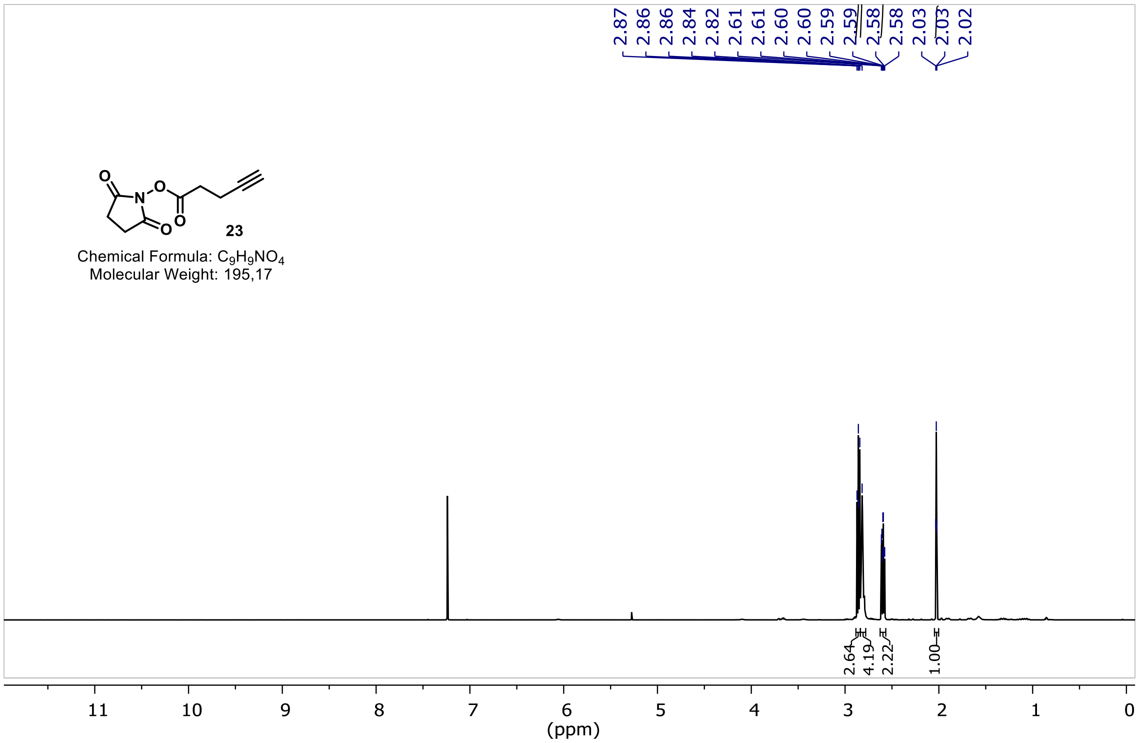

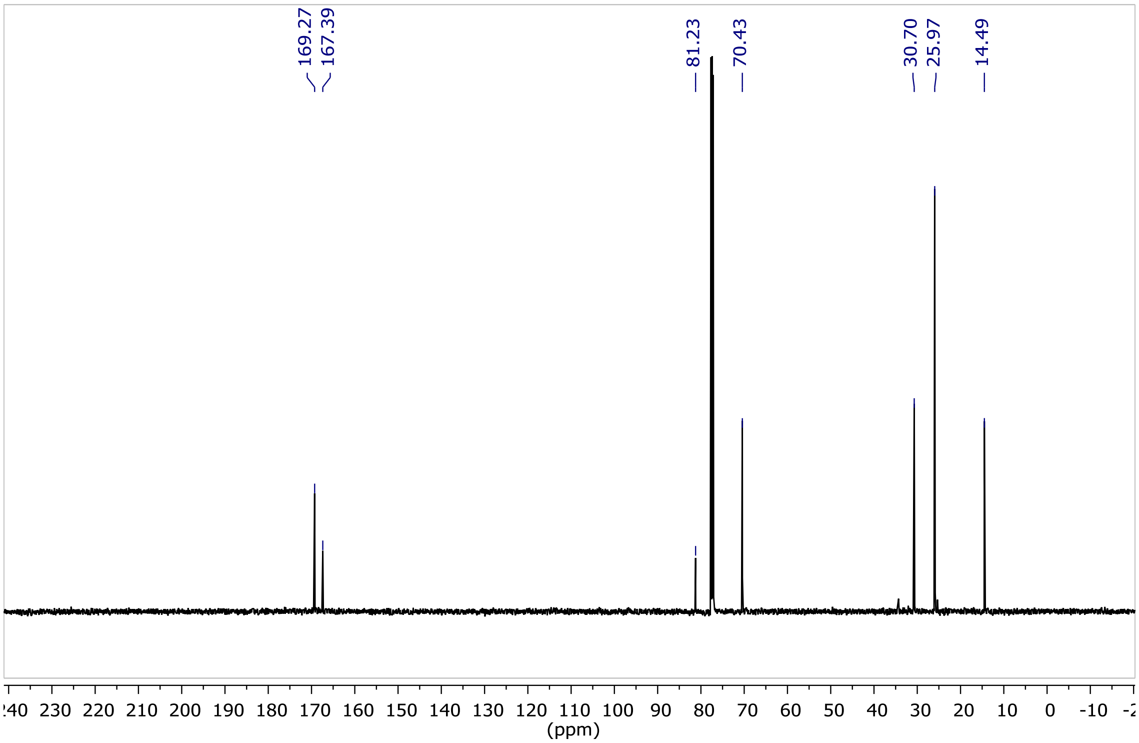

Compound 20 - N-((1R,2E,4E,7R,8Z,10E)-1-((2S,4R,5S,6S)-4-hydroxy-5-methyl-6-((E)-prop-1-en-1-yl)tetrahydro-2H-pyran-2-yl)-5,7,9-trimethyl-11-((R)-6-oxo-3,6-dihydro-2H-pyran-2-yl)undeca-2,4,8,10-tetraen-1-yl)pent-4-ynamide

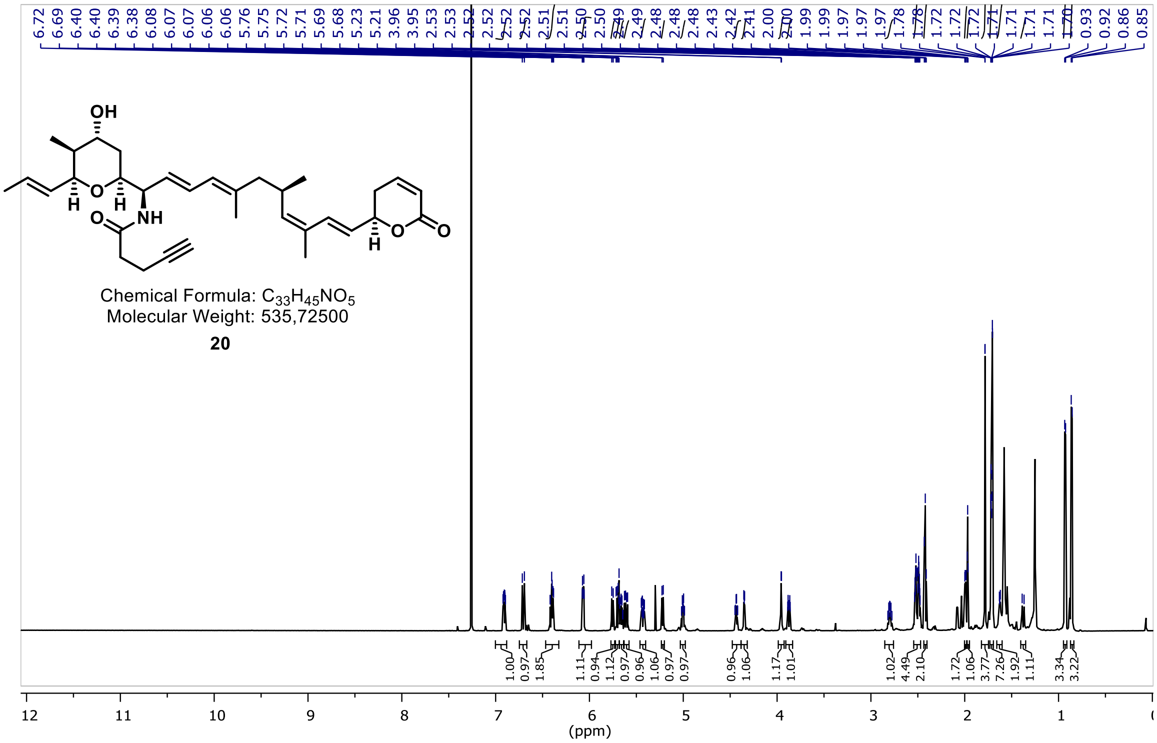

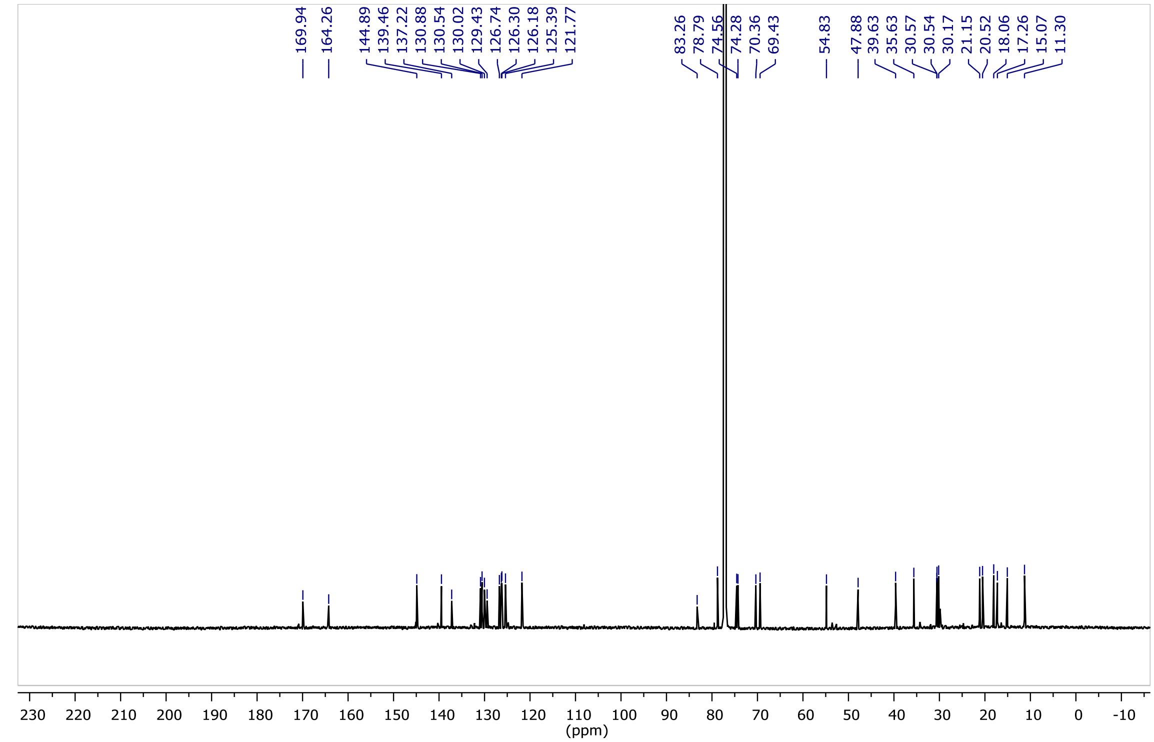

But-3-yn-1-yl (4-nitrophenyl) carbonate (23)

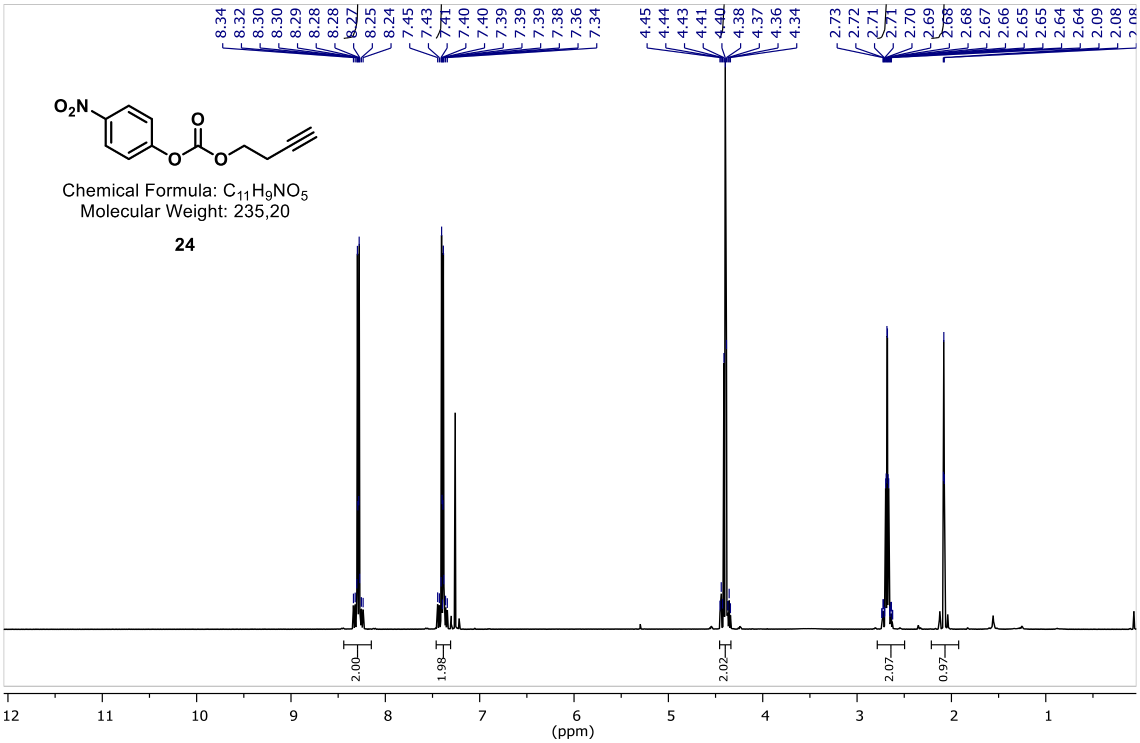

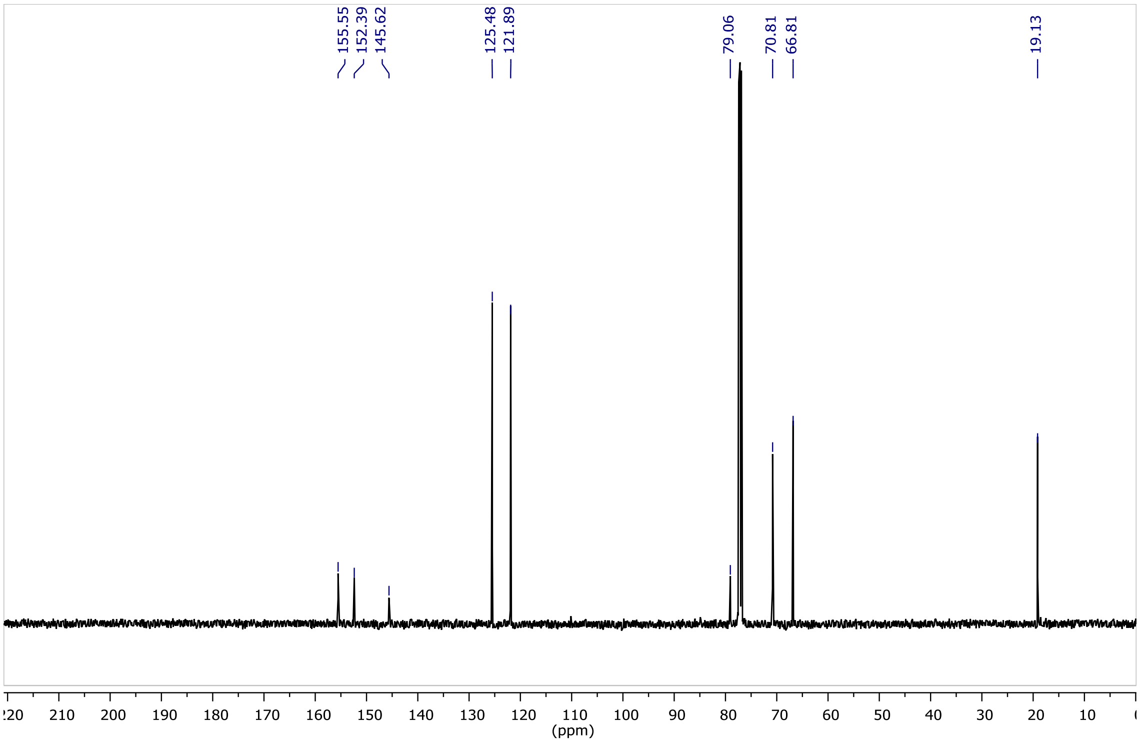

Compound 21 - But-3-yn-1-yl ((1R,2E,4E,7R,8Z,10E)-1-((2S,4R,5S,6S)-4-hydroxy-5-methyl-6-((E)-prop-1-en-1-yl)tetrahydro-2H-pyran-2-yl)-5,7,9-trimethyl-11-((R)-6-oxo-3,6-dihydro-2H-pyran-2-yl)undeca-2,4,8,10-tetraen-1-yl)carbamate

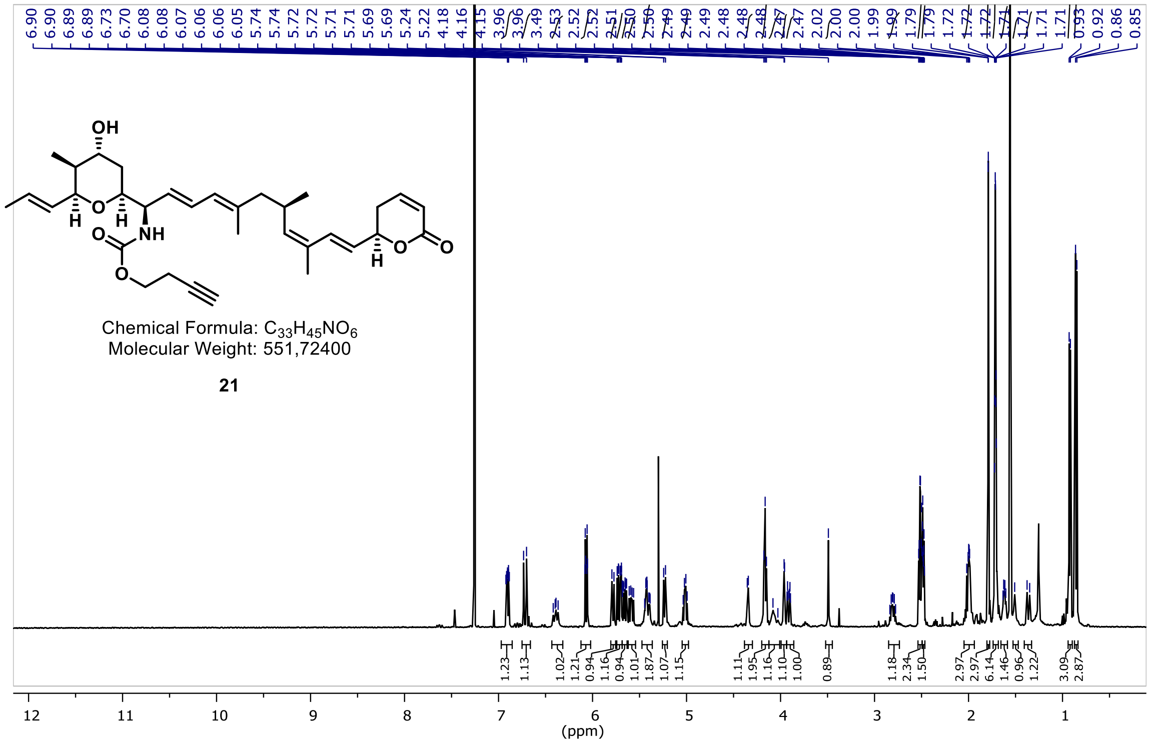

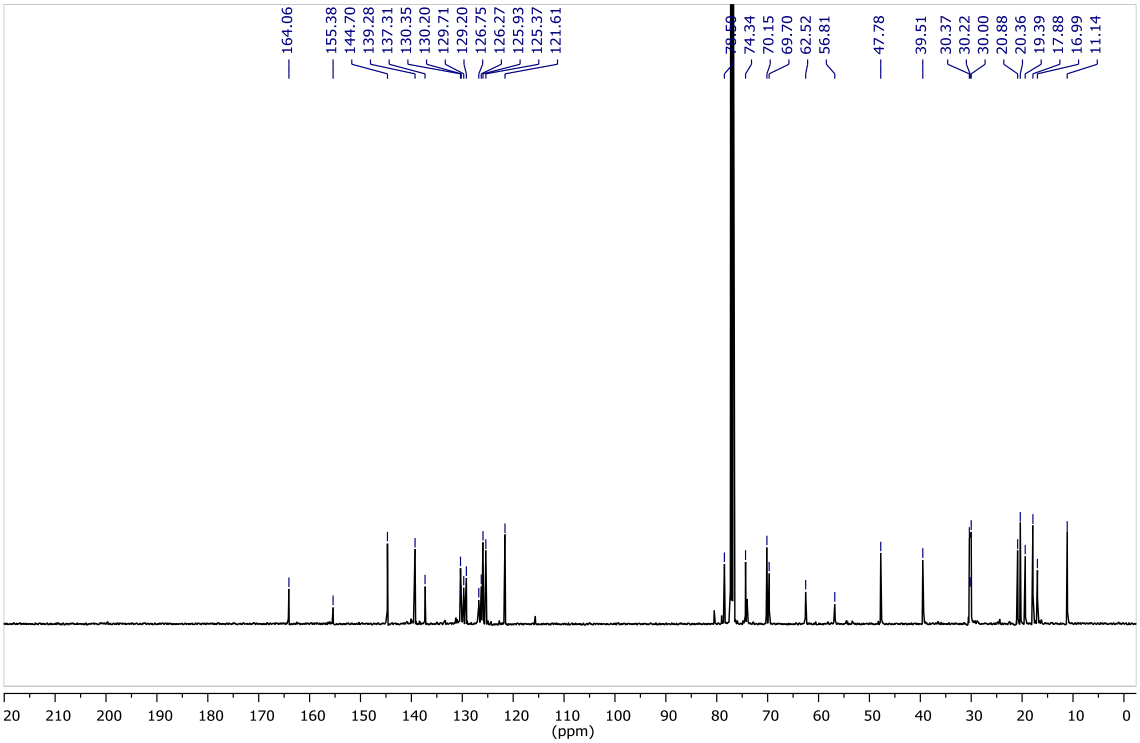

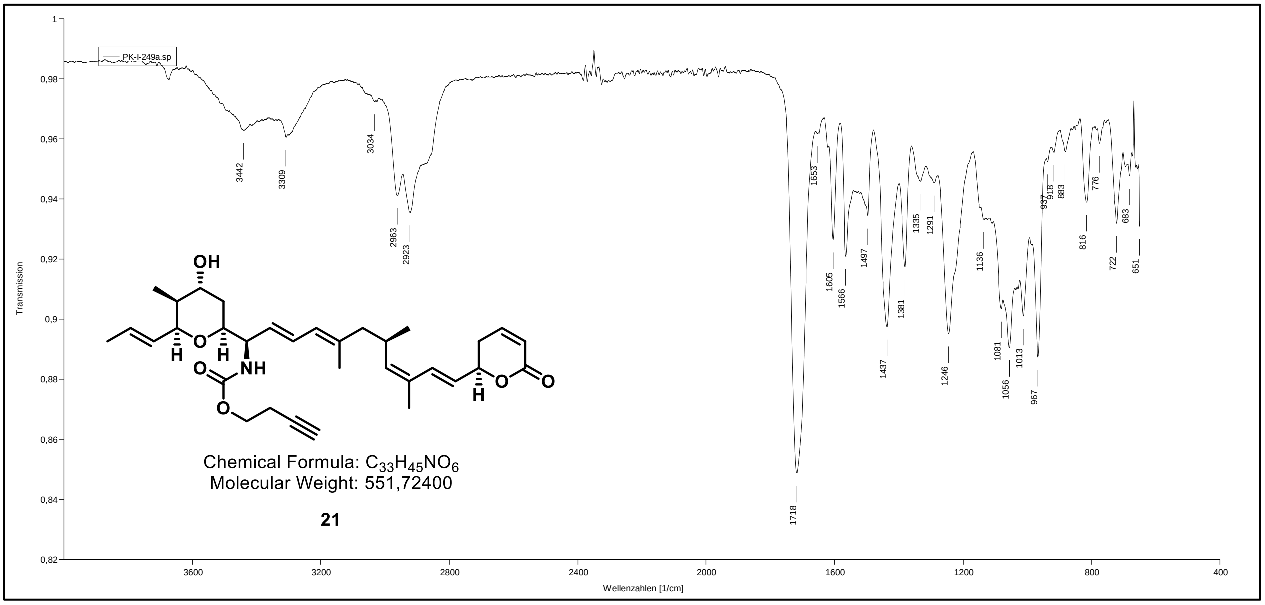
PK-I-249a.sp
Transmission
Wellenzahlen [1/cm]
3442
3309
3034
2963
2923
1718
1653
1605
1566
1497
1437
1381
1335
1291
1246
1136
1081
1056
1013
967
937
918
883
816
776
722
683
651
OH
O
H
NH
Chemical Formula: C33H45NO6
Molecular Weight: 551,72400
21

Compound 22 - But-3-yn-1-yl ((1S,2E,4E,7R,8Z,10E)-1-((2S,4R,5S,6S)-4-hydroxy-5-methyl-6-((E)-prop-1-en-1-yl)tetrahydro-2H-pyran-2-yl)-5,7,9-trimethyl-11-((R)-6-oxo-3,6-dihydro-2H-pyran-2-yl)undeca-2,4,8,10-tetraen-1-yl)carbamate

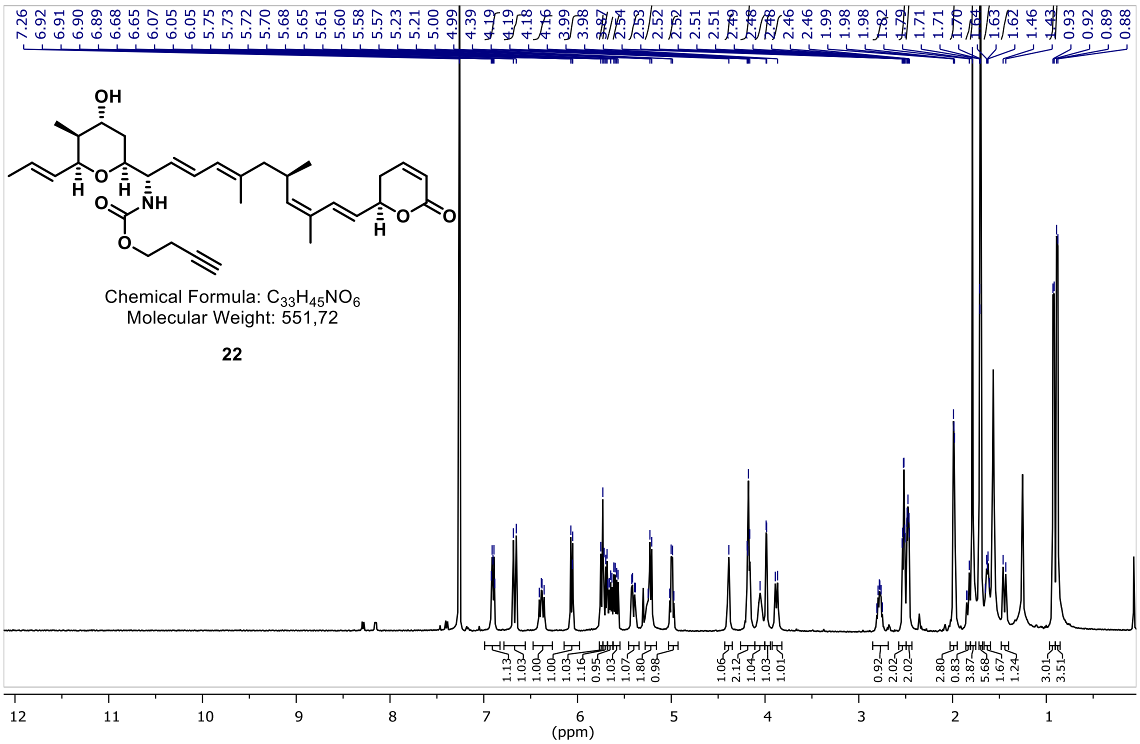

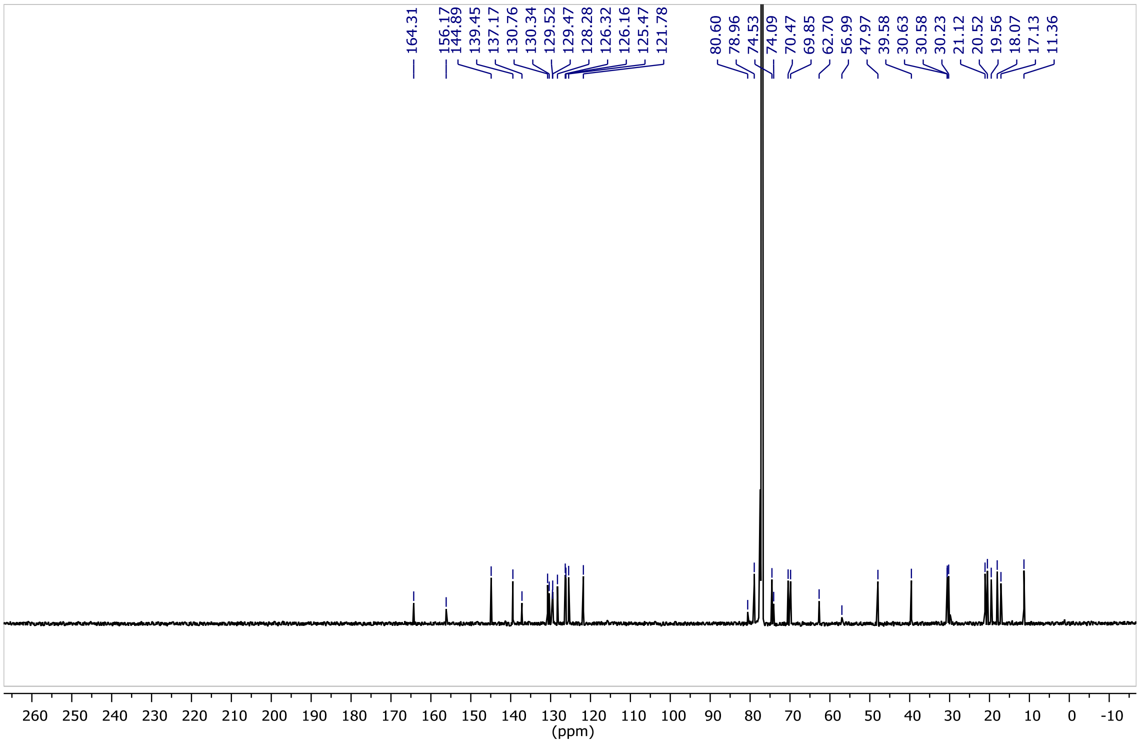

Fmoc-Cit-*p*ABA – (9H-fluoren-9-yl)methyl (S)-(1-((4-(hydroxymethyl)phenyl)amino)-1-oxo-5-ureidopentan-2-yl)carbamate

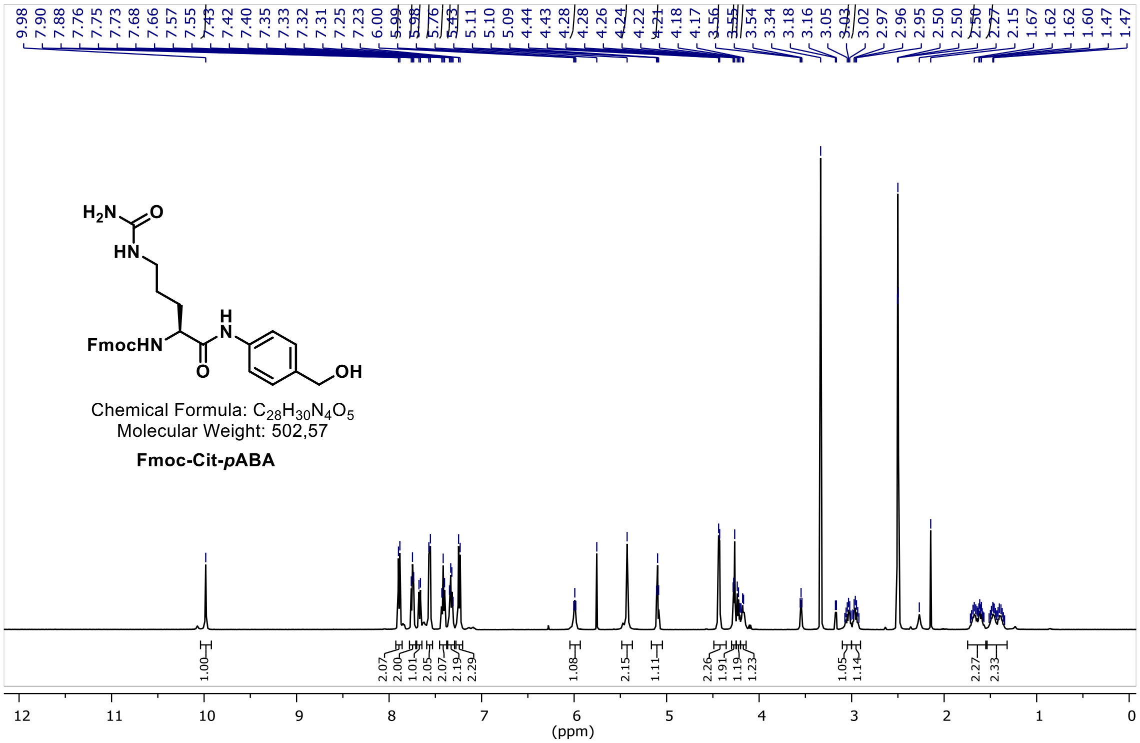

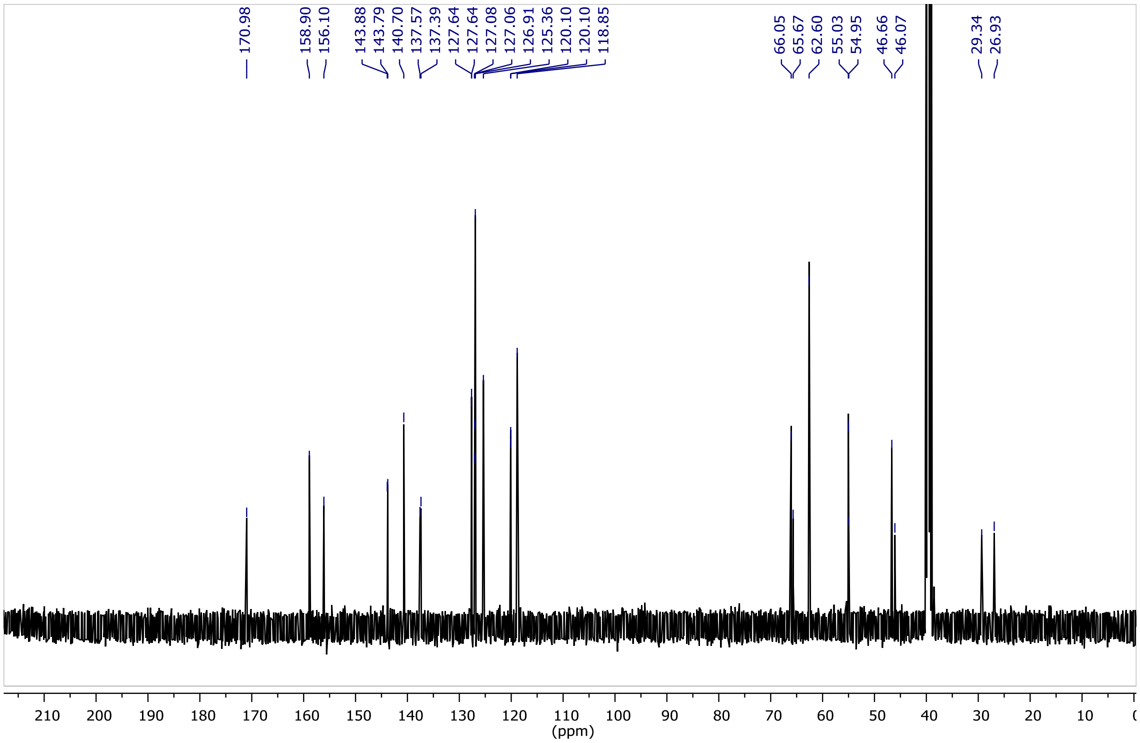

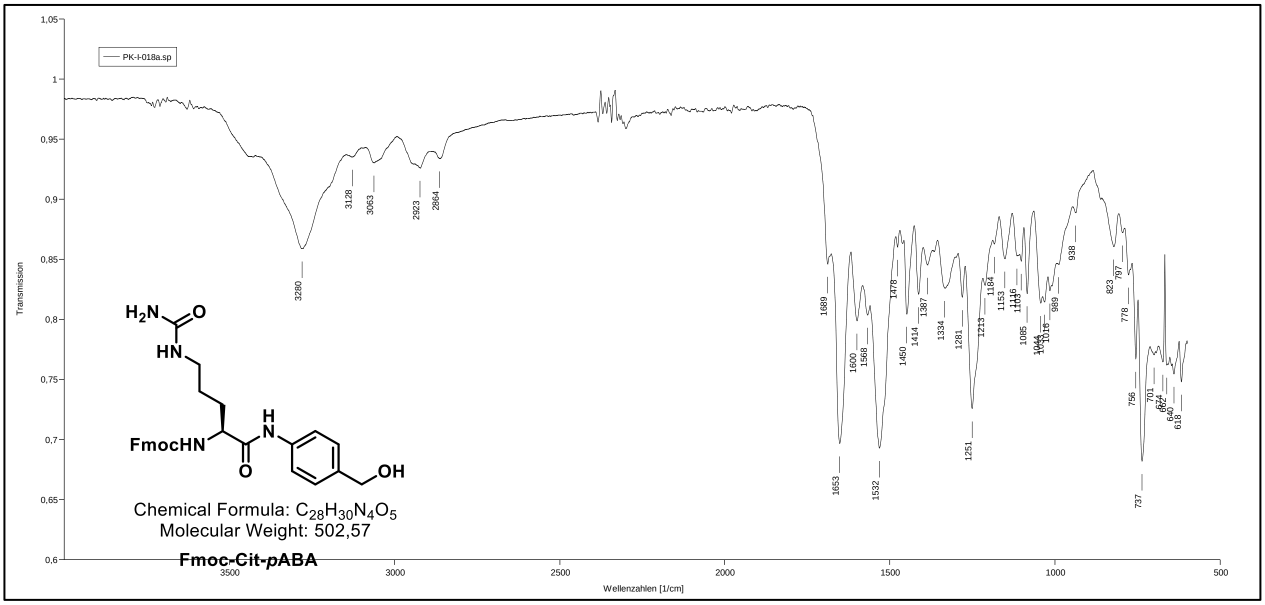
PK-I-018a.sp
Transmission
Wellenzahlen [1/cm]
1,05
1
0,95
0,9
0,85
0,8
0,75
0,7
0,65
0,6
3500
3000
2500
2000
1500
1000
500
3280
3128
3063
2923
2864
1689
1653
1600
1568
1532
1478
1450
1414
1387
1334
1281
1251
1213
1184
1153
1116
1103
1085
1044
1016
989
938
823
797
778
756
737
701
674
640
618
H2N
O
HN
FmocHN
H
N
O
OH
Chemical Formula: $C_{28}H_{30}N_4O_5$
Molecular Weight: 502,57
Fmoc-Cit-*p*ABA

H₂N-Val-Cit-*p*ABA – S)-2-((S)-2-amino-3-methylbutanamido)-N-(4-(hydroxymethyl)phenyl)-5-ureidopentanamide

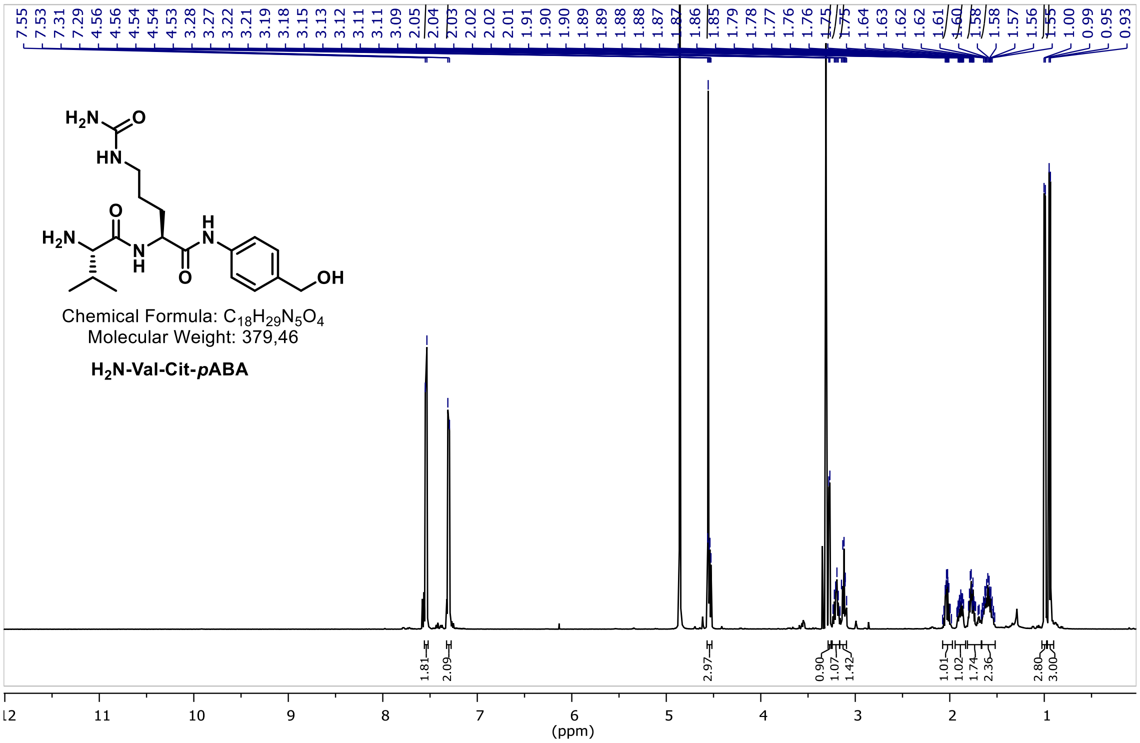

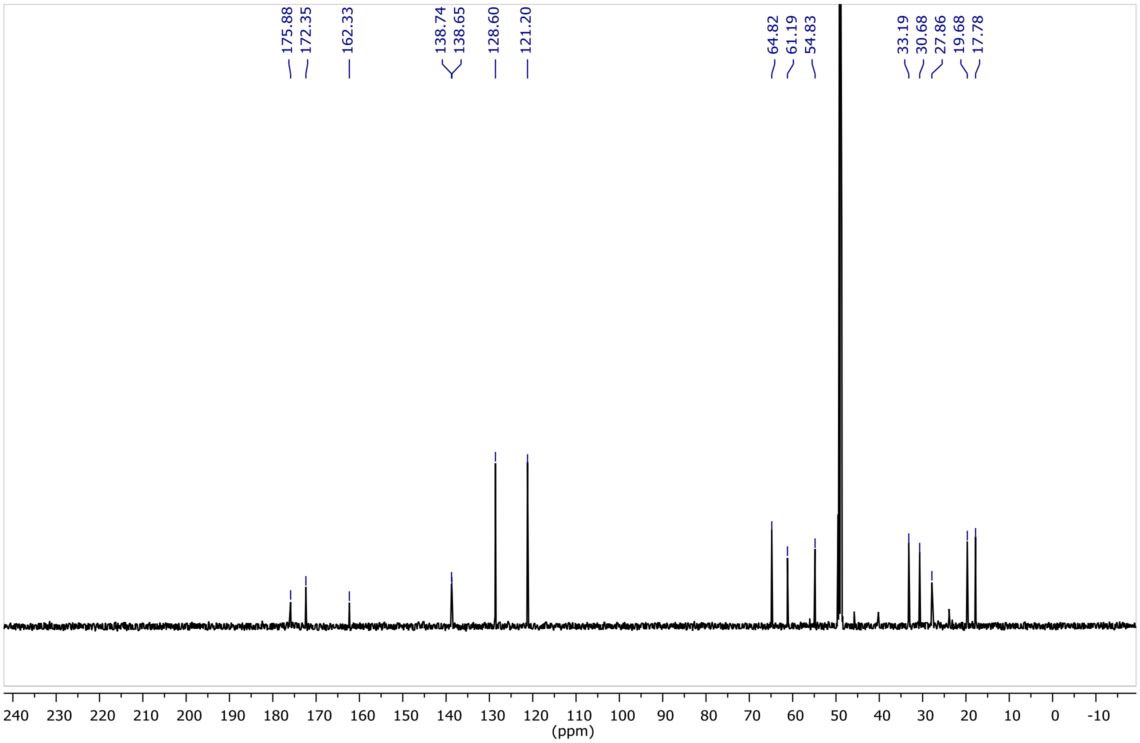

BCN-*p*NPC – ((1R,8S,9s)-bicyclo[6.1.0]non-4-yn-9-yl)methyl (4-nitrophenyl) carbonate

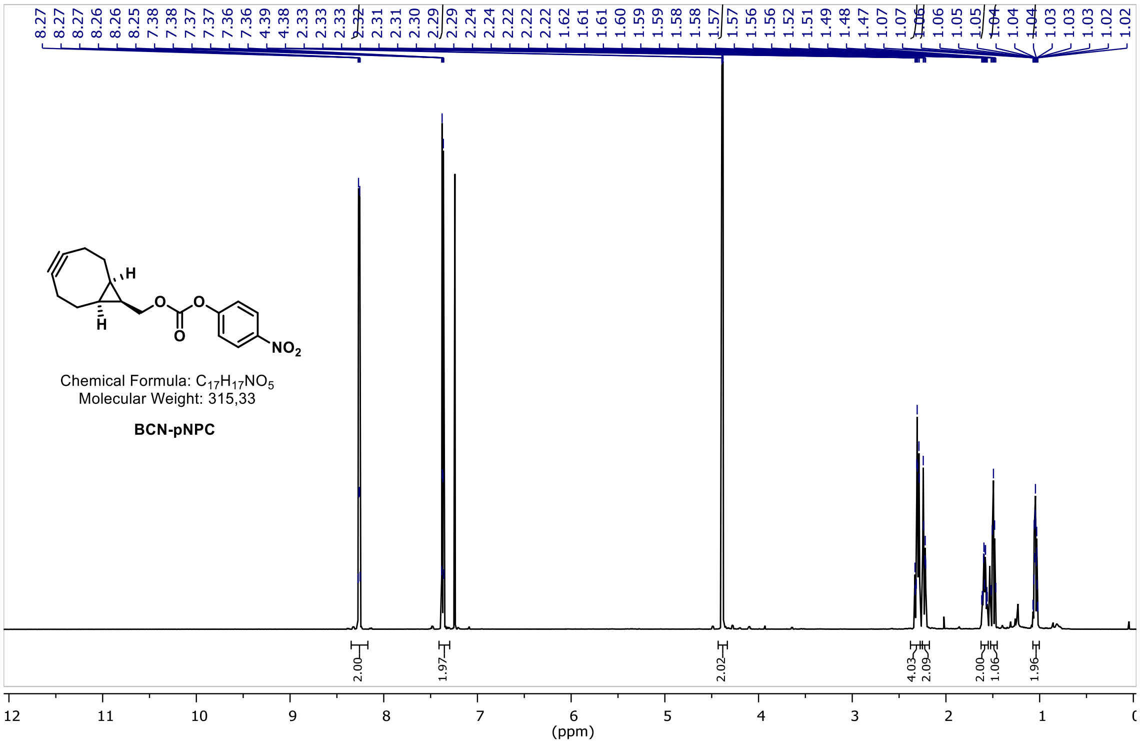

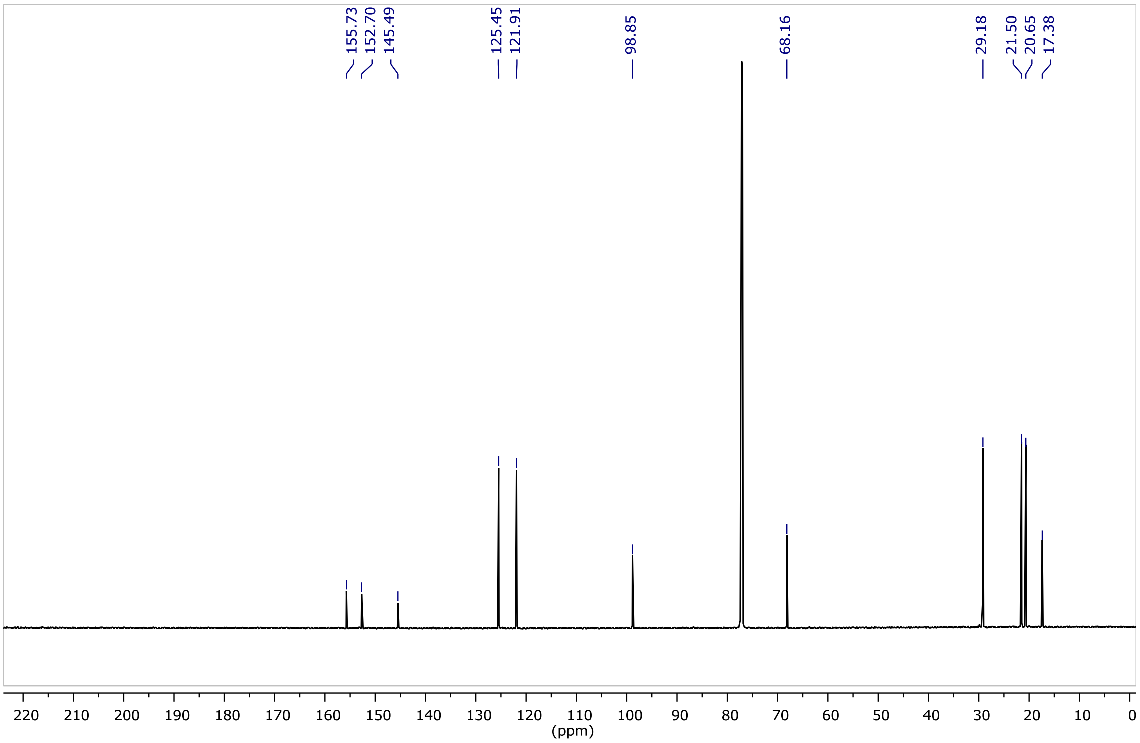

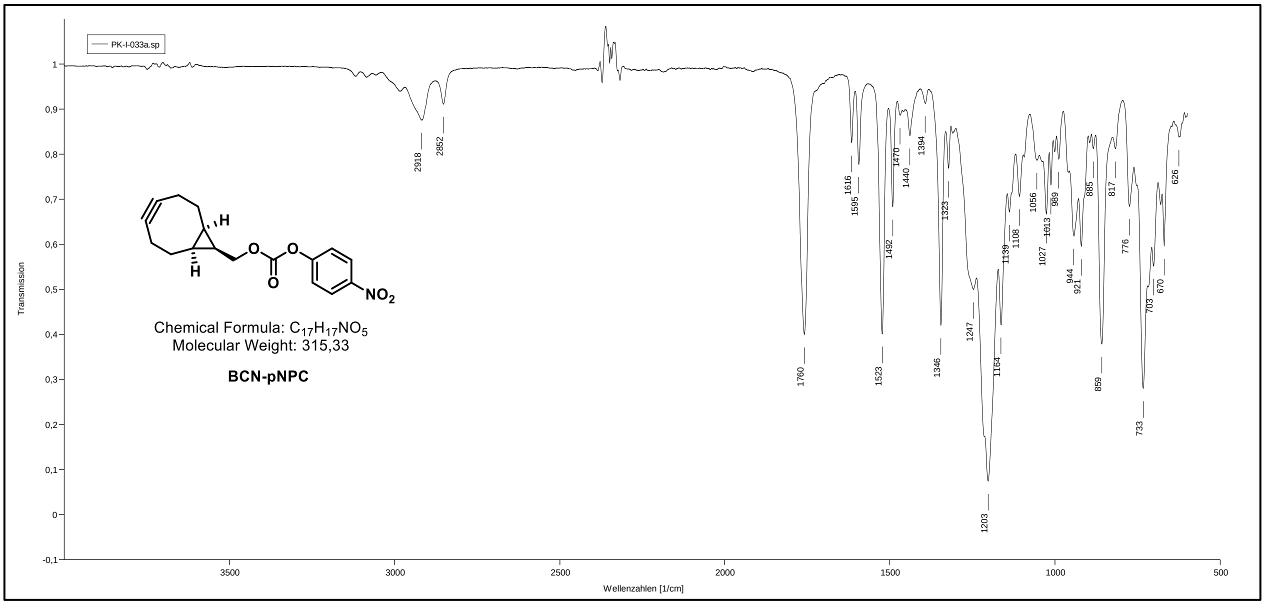
PK-I-033a.sp
Transmission
Wellenzahlen [1/cm]
Chemical Formula: $C_{17}H_{17}NO_5$
Molecular Weight: 315,33
BCN-pNPC
2918
2852
1760
1616
1595
1523
1492
1470
1440
1394
1346
1323
1247
1203
1164
1139
1108
1056
1027
1013
989
944
921
885
859
817
776
733
703
670
626

BCN-O(CO)HN-Val-Cit-*p*ABA – (S)-2-((S)-2-amino-3-methylbutanamido)-N-(4-(hydroxymethyl)-phenyl)-5-ureidopentanamide

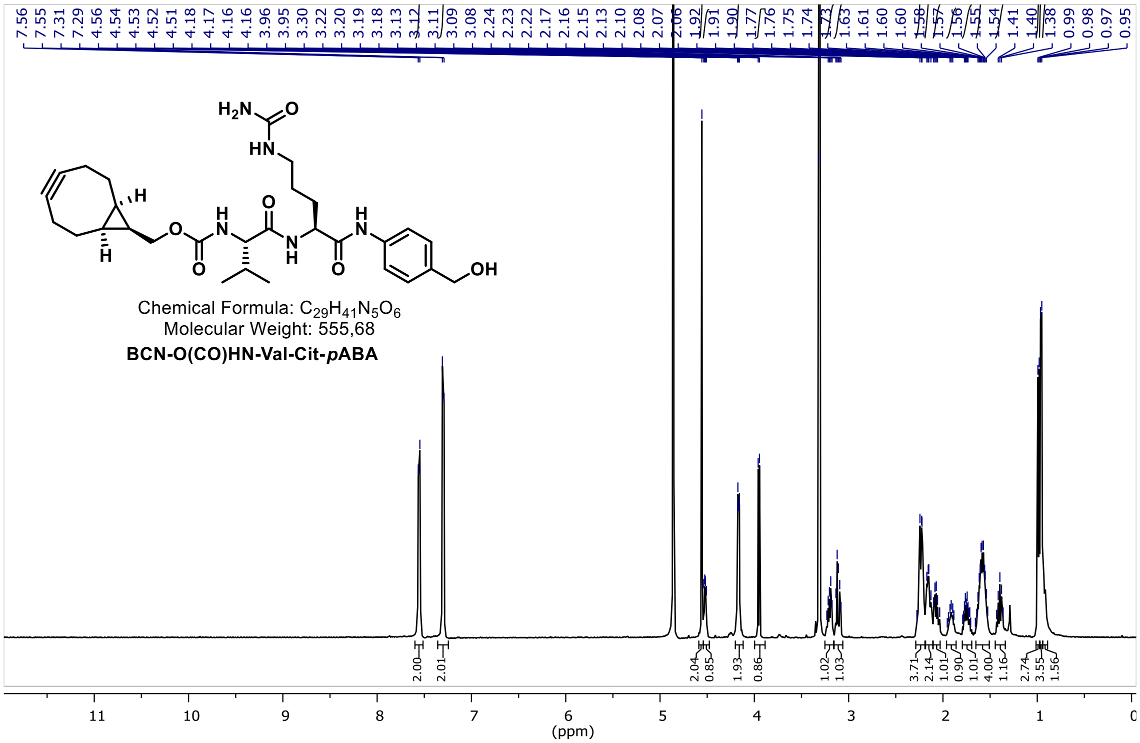

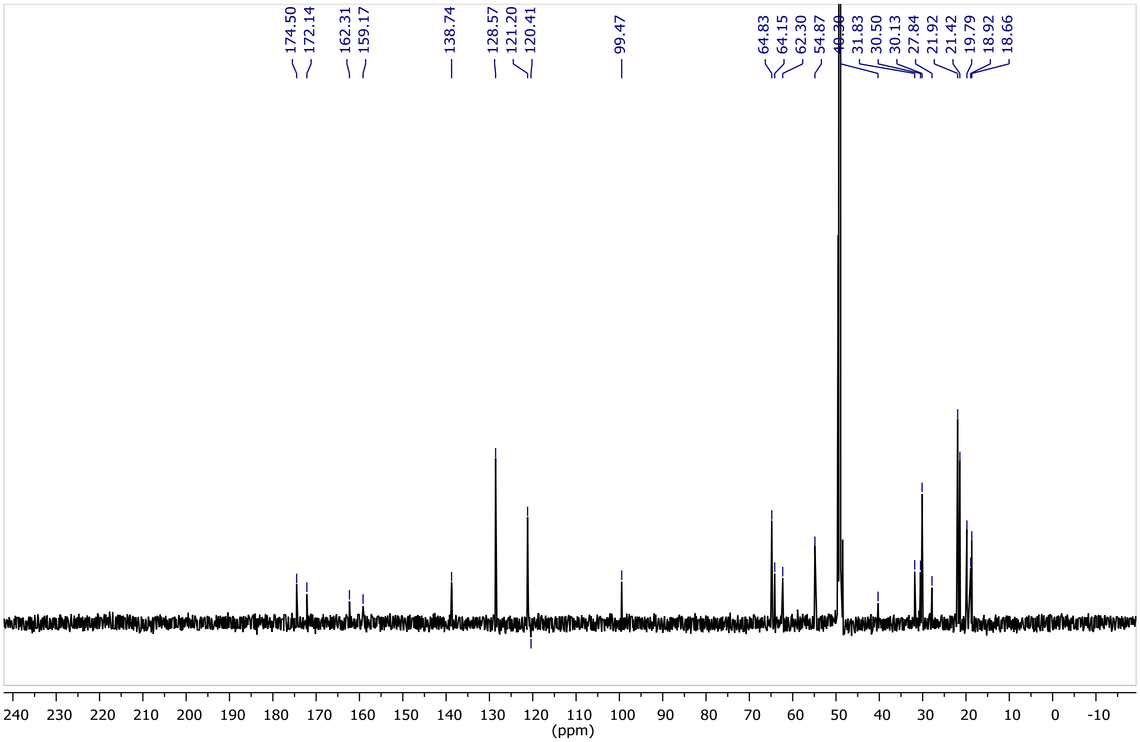

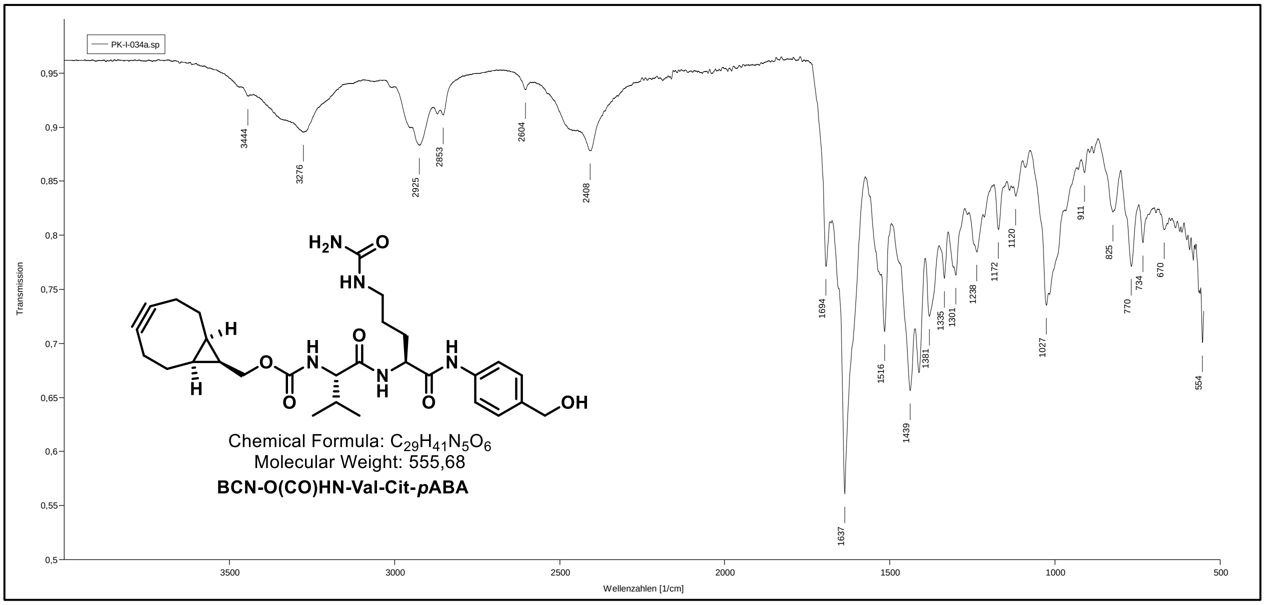
PK-I-034a.sp
Transmission
Wellenzahlen [1/cm]
0,95
0,9
0,85
0,8
0,75
0,7
0,65
0,6
0,55
0,5
3500
3000
2500
2000
1500
1000
500
3444
3276
2925
2853
2604
2408
1694
1637
1516
1439
1381
1335
1301
1238
1172
1120
1027
911
825
770
734
670
554
Chemical Formula: C29H41N5O6
Molecular Weight: 555,68
BCN-O(CO)HN-Val-Cit-pABA

BCN-O(CO)HN-Val-Cit-*p*ABO(CO)O(4-NO₂-Ph) – ((1R,8S,9s)-bicyclo[6.1.0]non-4-yn-9-yl)methyl ((S)-3-methyl-1-(((S)-1-((4-((((4-nitrophenoxy)carbonyl)oxy)methyl)phenyl)amino)-1-oxo-5-ureidopentan-2-yl)amino)-1-oxobutan-2-yl)carbamate

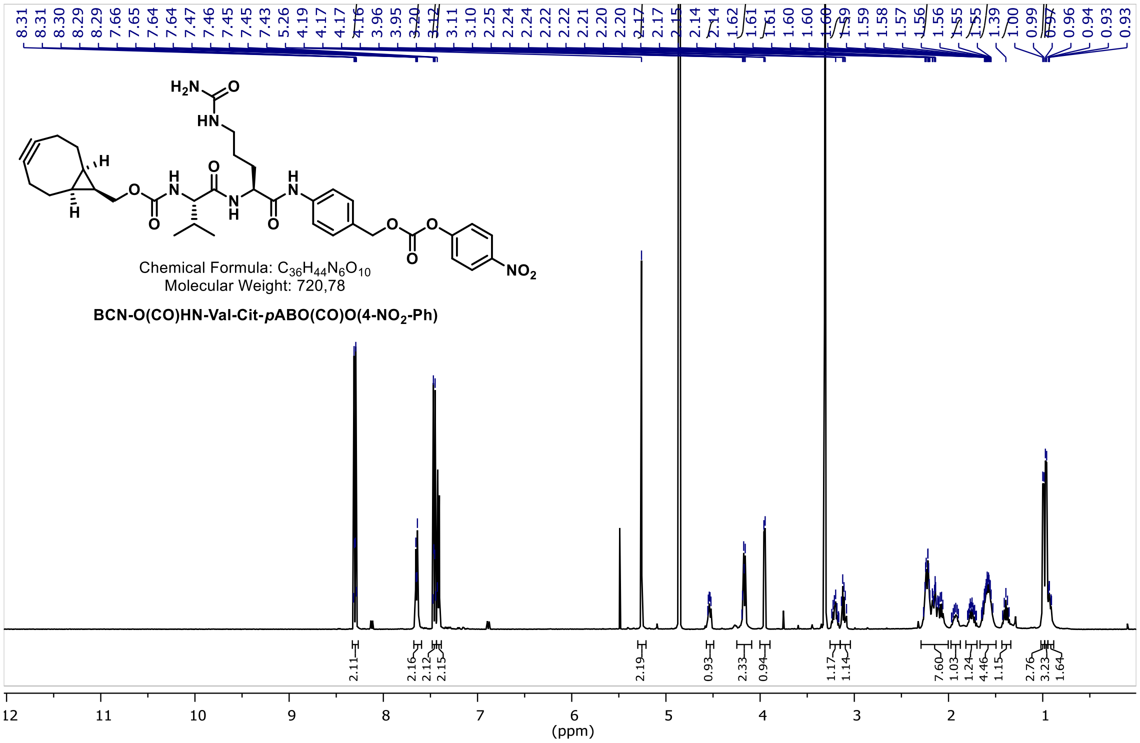

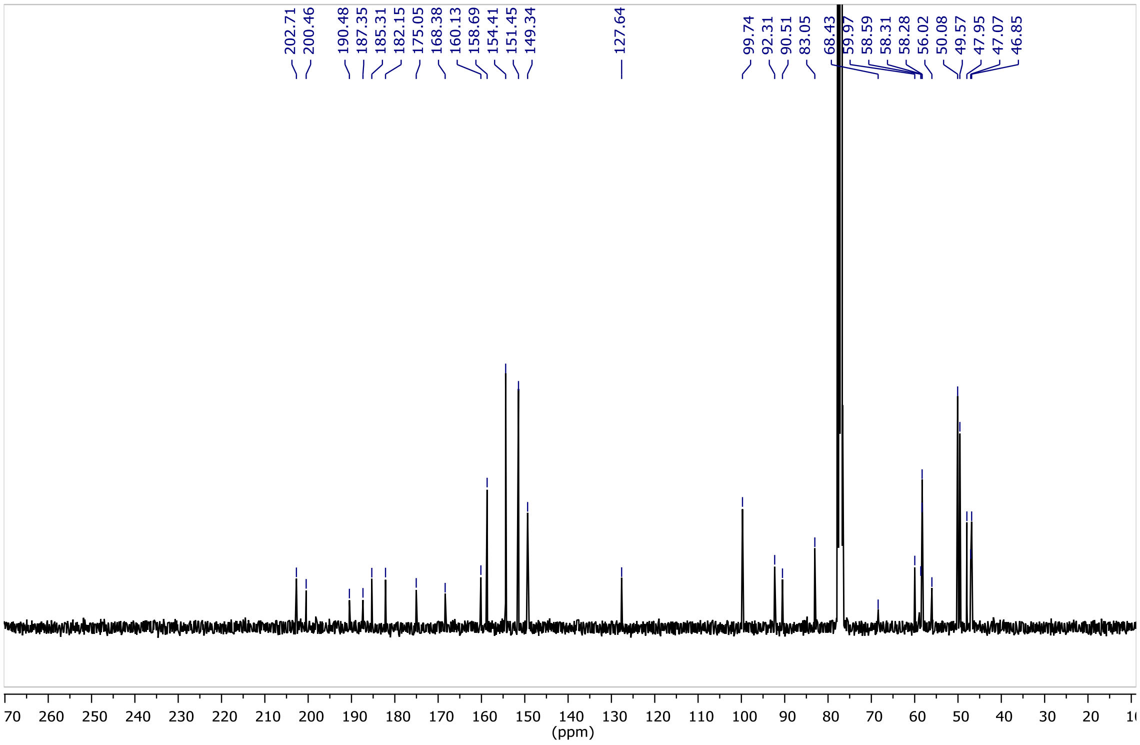

BCN-O(CO)HN-Val-Cit-*p*ABO(CO)-16*R*-Aminoratjadone (25) – ((1R,8S,9s)-bicyclo[6.1.0]non-4-yn-9-yl)methyl ((S)-1-(((S)-1-((4-(((((1R,2E,4E,7R,8Z,10E)-1-((2S,4R,5S,6S)-4-hydroxy-5-methyl-6-((E)-prop-1-en-1-yl)tetrahydro-2H-pyran-2-yl)-5,7,9-trimethyl-11-((R)-6-oxo-3,6-dihydro-2H-pyran-2-yl)undeca-2,4,8,10-tetraen-1-yl)carbamoyl)oxy)methyl)phenyl)amino)-1-oxo-5-ureidopentan-2-yl)amino)-3-methyl-1-oxobutan-2-yl)carbamate

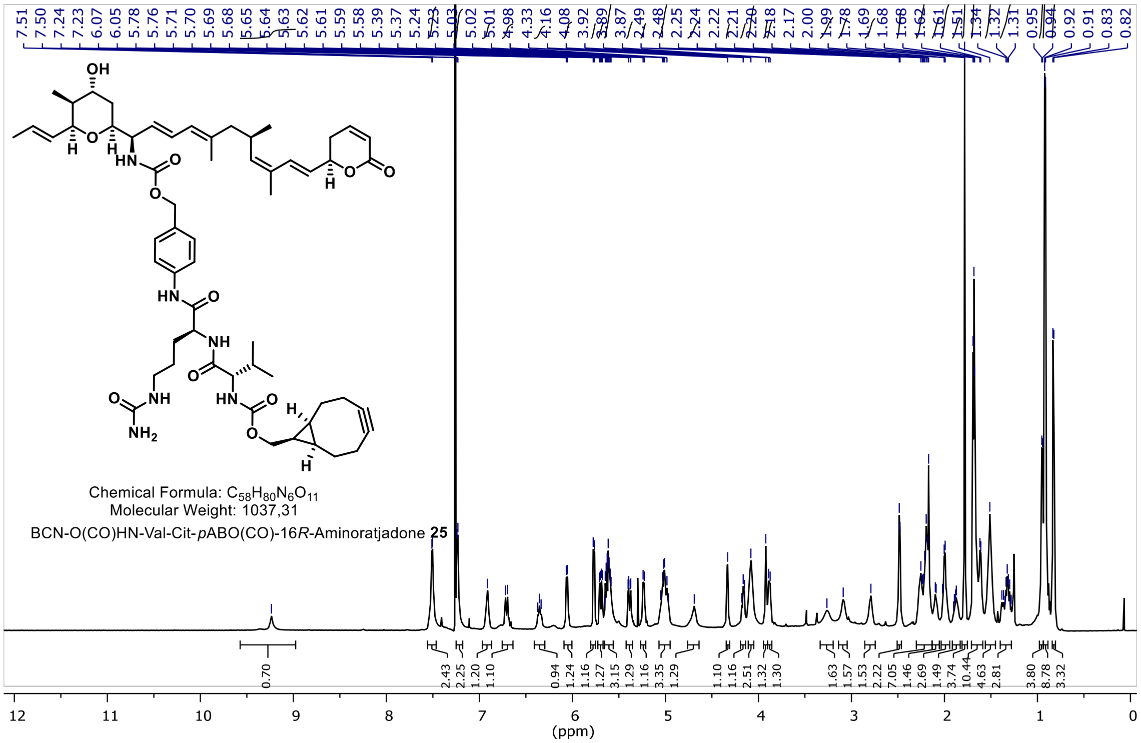

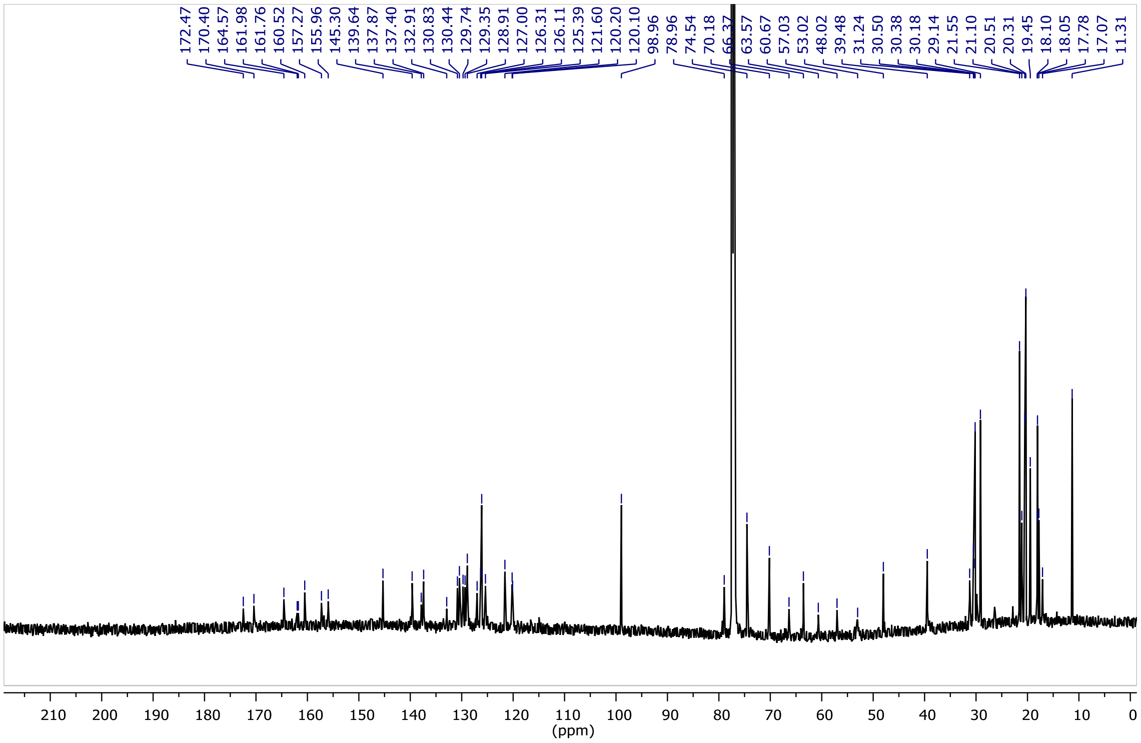

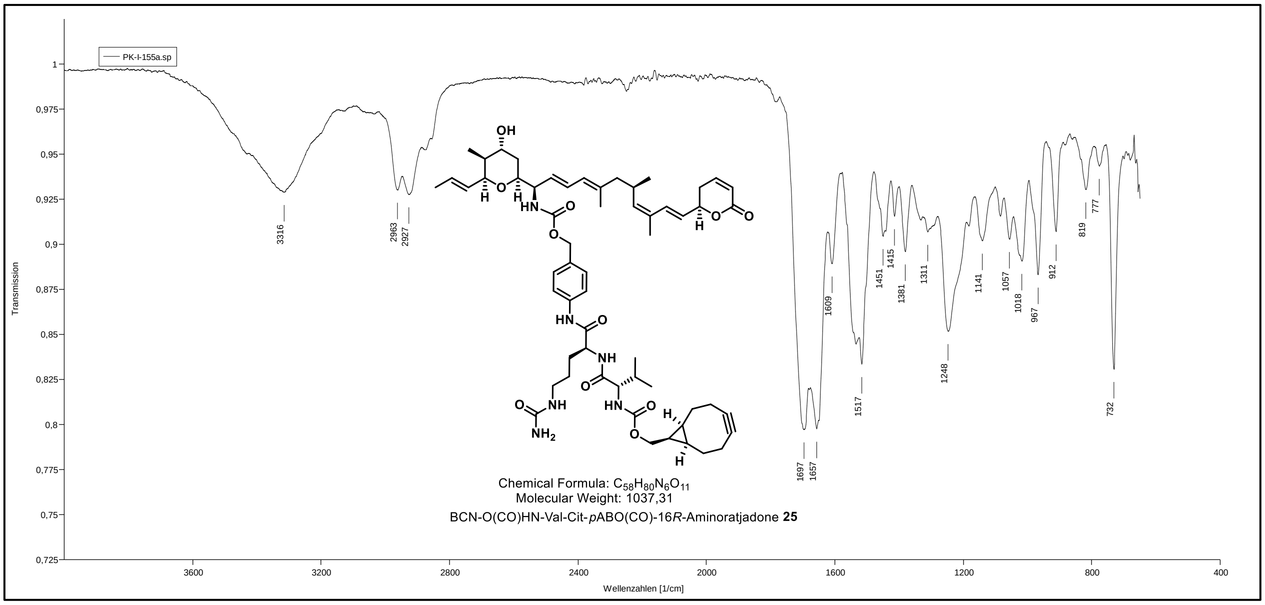
PK-I-155a.sp
Transmission
Wellenzahlen [1/cm]
3316
2963
2927
1697
1657
1609
1517
1451
1415
1381
1311
1248
1141
1057
1018
967
912
819
777
732
Chemical Formula: C58H80N6O11
Molecular Weight: 1037,31
BCN-O(CO)HN-Val-Cit-pABO(CO)-16R-Aminoratjadone 25

Homopropargyl-O(CO)HN-Val-Cit-*p*ABA – But-3-yn-1-yl ((S)-1-(((S)-1-((4-(hydroxymethyl)phenyl)-amino)-1-oxo-5-ureidopentan-2-yl)amino)-3-methyl-1-oxobutan-2-yl)carbamate

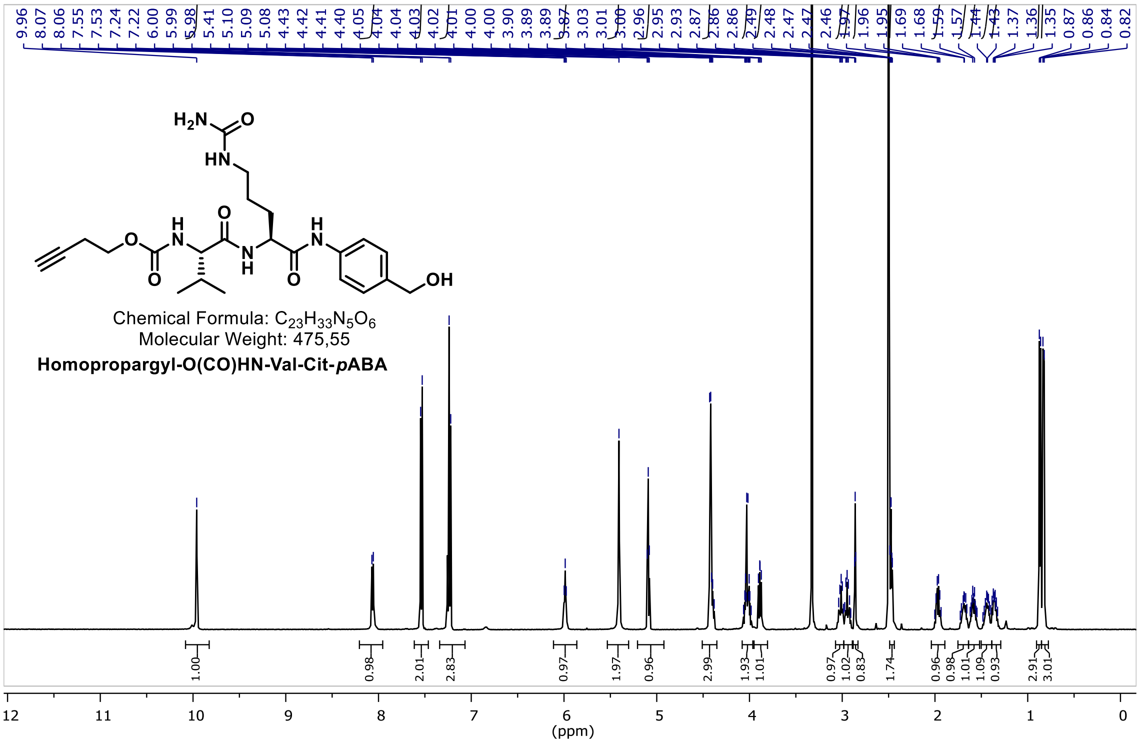

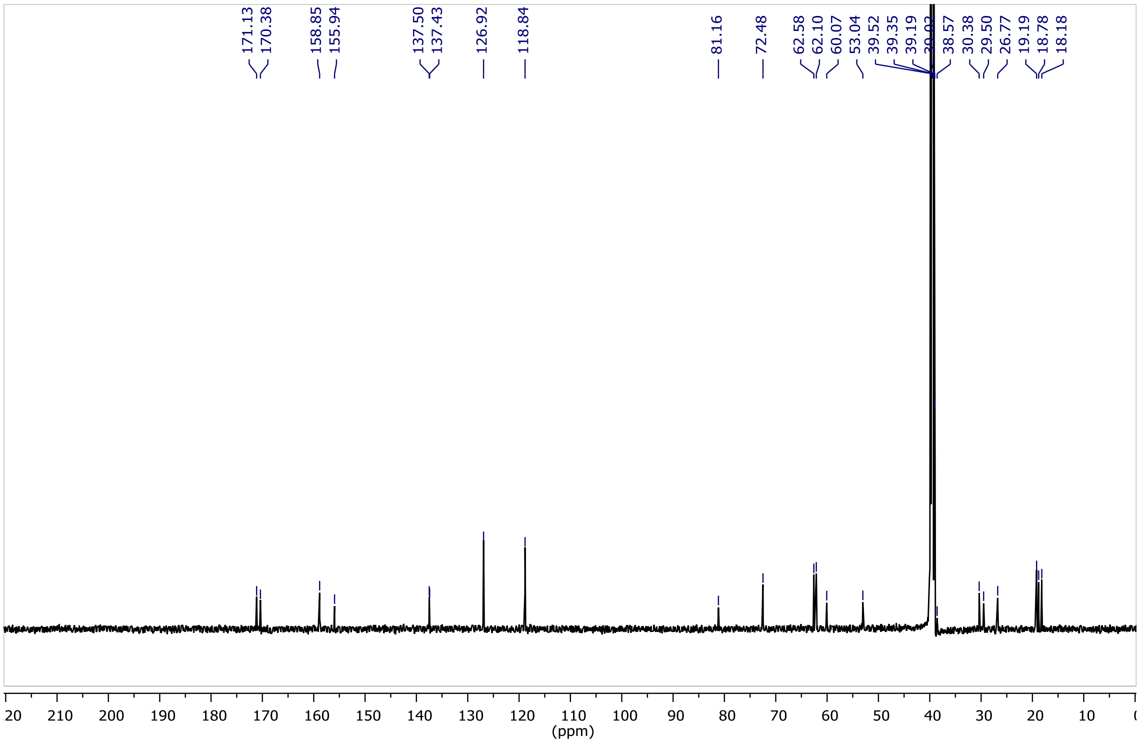

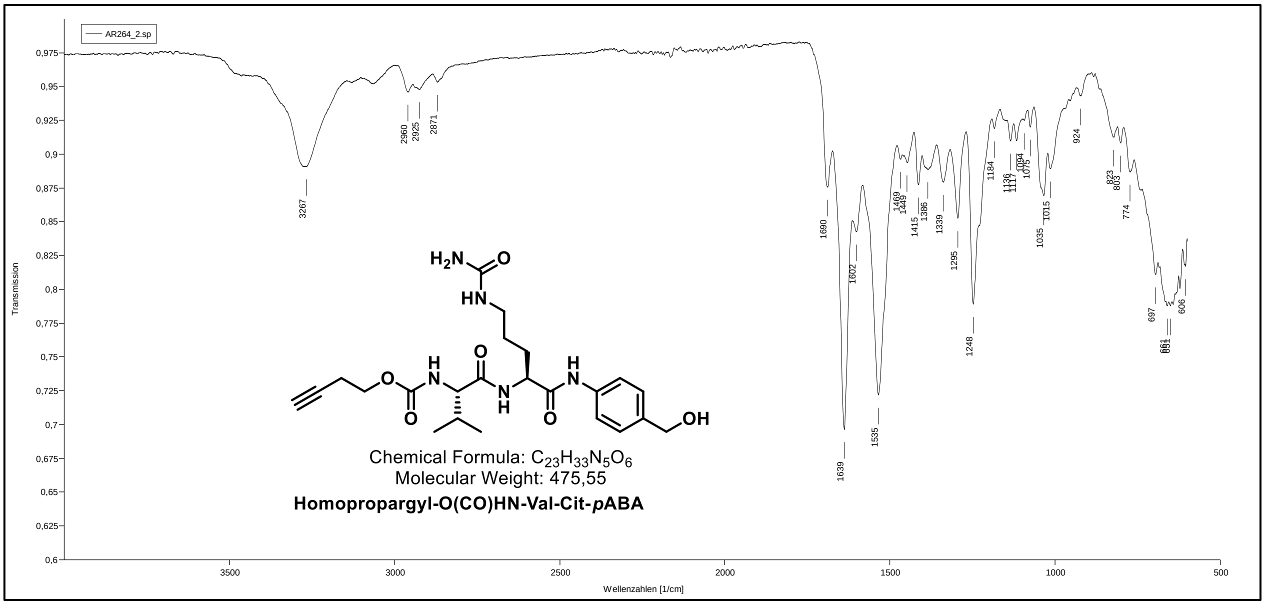
AR264_2.sp
Transmission
Wellenzahlen [1/cm]
3267
2960
2925
2871
1690
1639
1602
1535
1459
1449
1415
1386
1339
1295
1248
1184
1136
1117
1094
1075
1035
1015
924
823
803
774
697
661
651
606
Chemical Formula: $C_{23}H_{33}N_5O_6$
Molecular Weight: 475,55
Homopropargyl-O(CO)HN-Val-Cit-*p*ABA

Homopropargyl-O(CO)HN-Val-Cit-*p*ABO(CO)O(4-NO$_2$-Ph) (28) – But-3-yn-1-yl ((S)-3-methyl-1-(((S)-1-((4-((((4-nitrophenoxy)carbonyl)oxy)methyl)phenyl)amino)-1-oxo-5-ureidopentan-2-yl)amino)-1-oxobutan-2-yl)carbamate

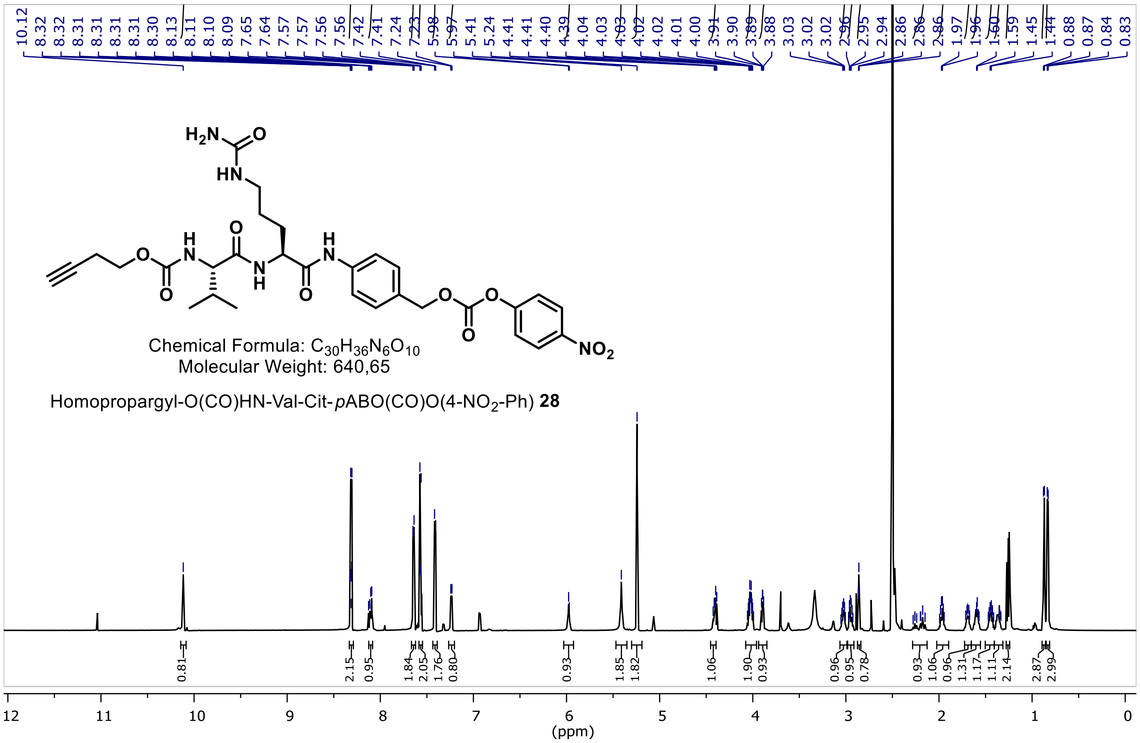

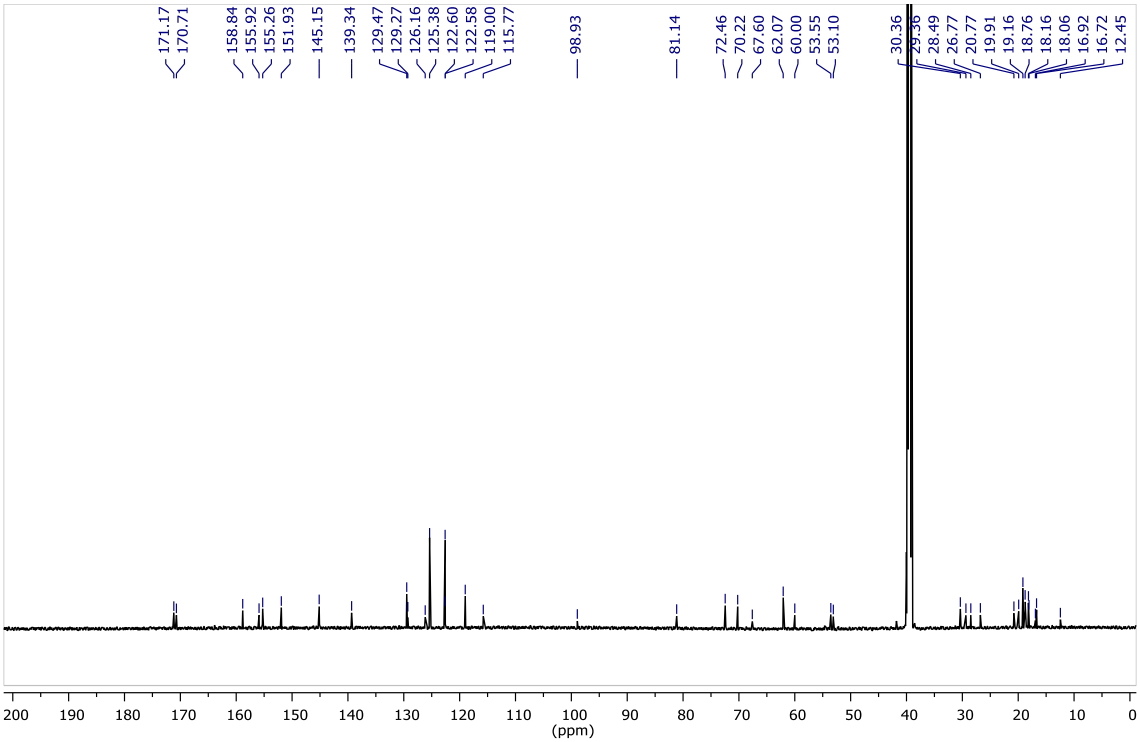

Homopropargyl-O(CO)HN-Val-Cit-*p*ABO(CO)-16R-Aminoratjadone (26) – But-3-yn-1-yl ((S)-1-(((S)-1-((4-(((((1R,2E,4E,7R,8Z,10E)-1-((2S,4R,5S,6S)-4-hydroxy-5-methyl-6-((E)-prop-1-en-1-yl)tetrahydro-2H-pyran-2-yl)-5,7,9-trimethyl-11-((R)-6-oxo-3,6-dihydro-2H-pyran-2-yl)undeca-2,4,8,10-tetraen-1-yl)carbamoyl)-oxy)methyl)phenyl)amino)-1-oxo-5-ureidopentan-2-yl)amino)-3-methyl-1-oxobutan-2-yl)carbamate

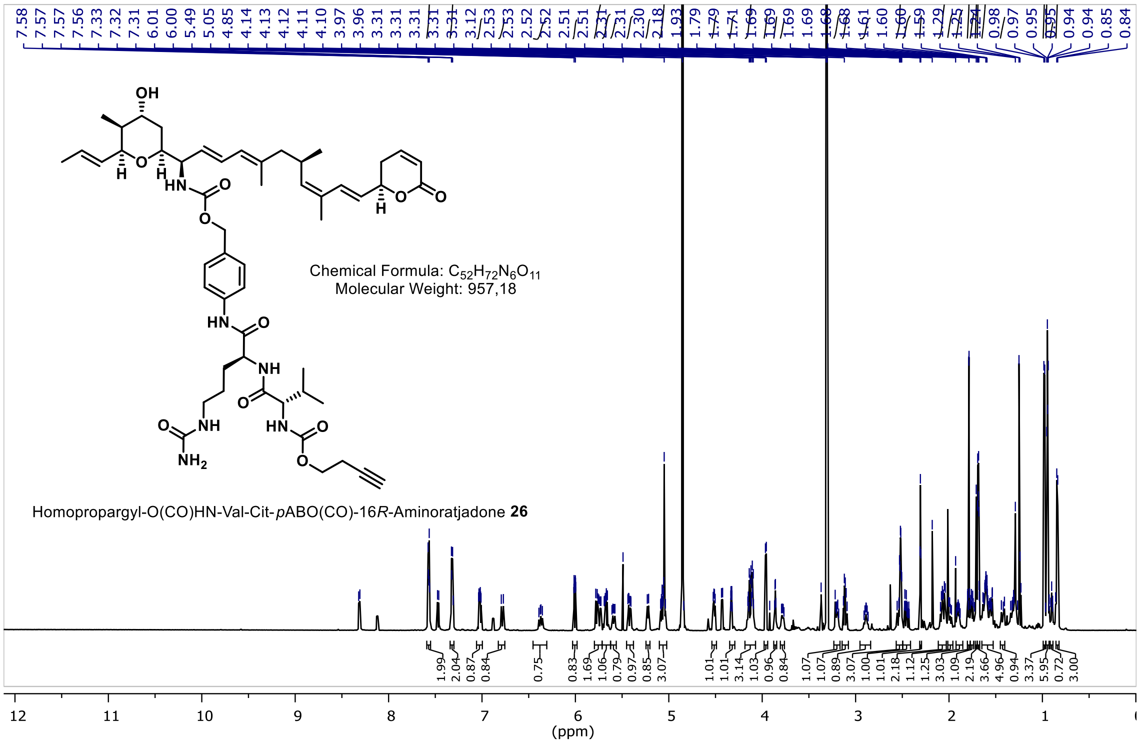

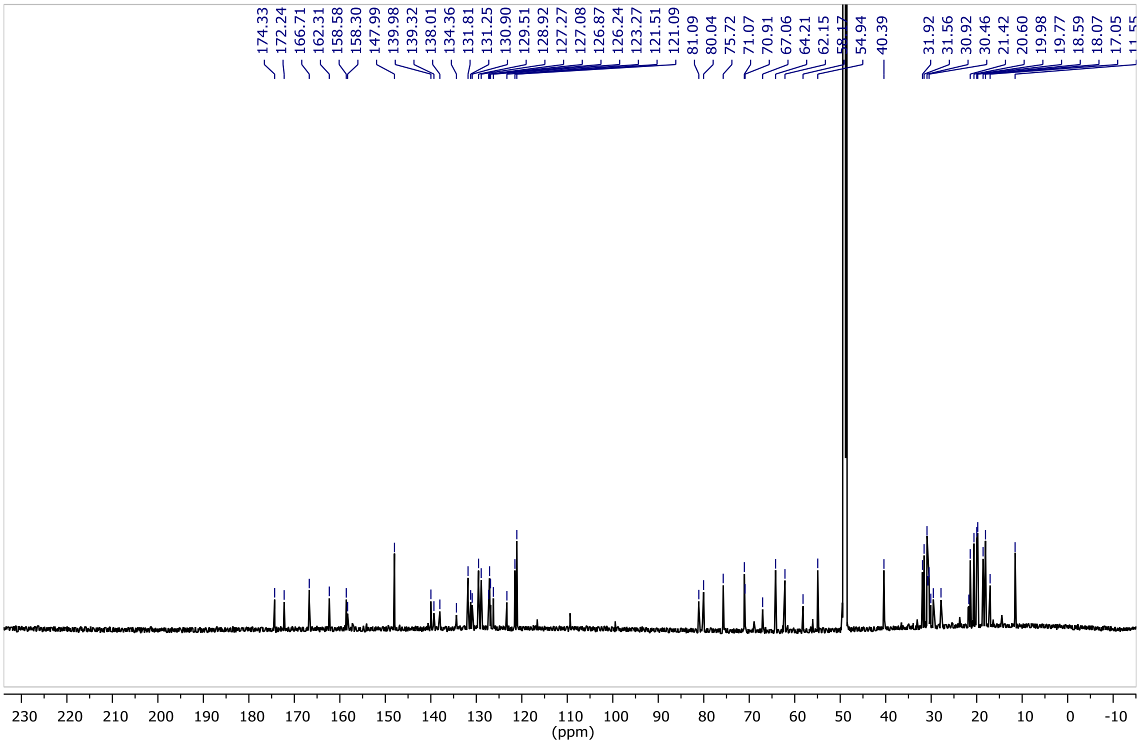

2-PySS(CH₂)₂(CO)-16*R*-Aminoratjadone (31) – N-((1R,2E,4E,7R,8Z,10E)-1-((2S,4R,5S,6S)-4-Hydroxy-5-methyl-6-((E)-prop-1-en-1-yl)tetrahydro-2H-pyran-2-yl)-5,7,9-trimethyl-11-((R)-6-oxo-3,6-dihydro-2H-pyran-2-yl)undeca-2,4,8,10-tetraen-1-yl)-3-(pyridin-2-yldisulfaneyl)propenamide

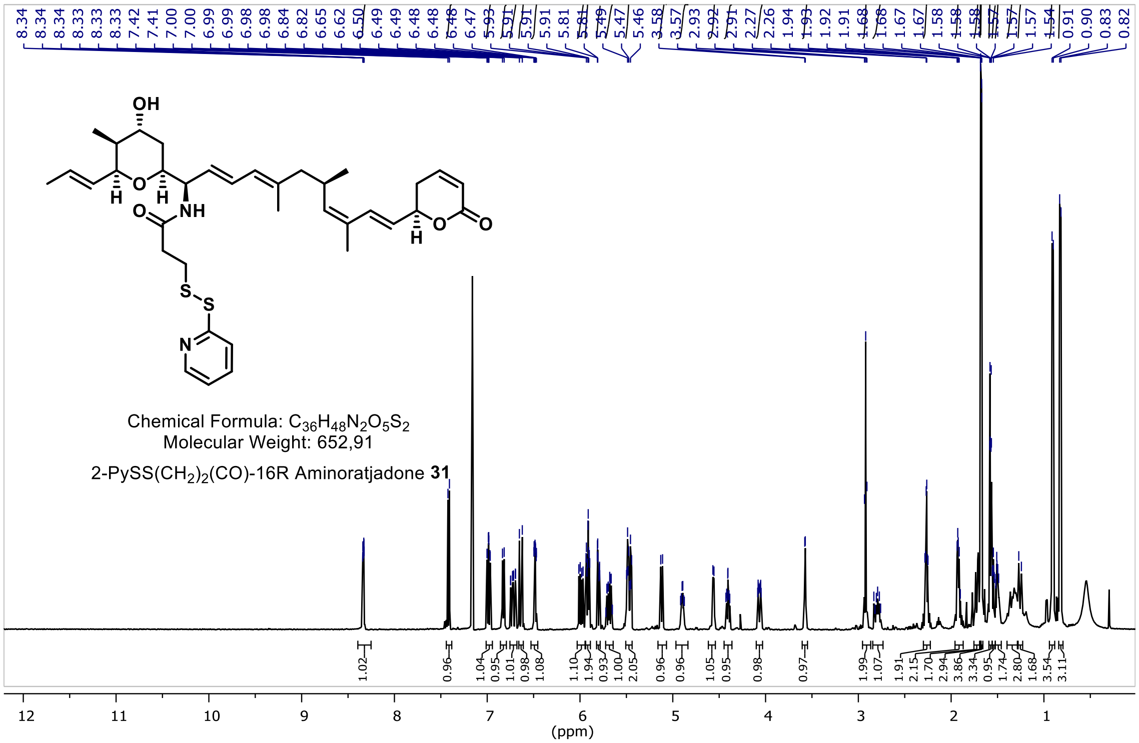

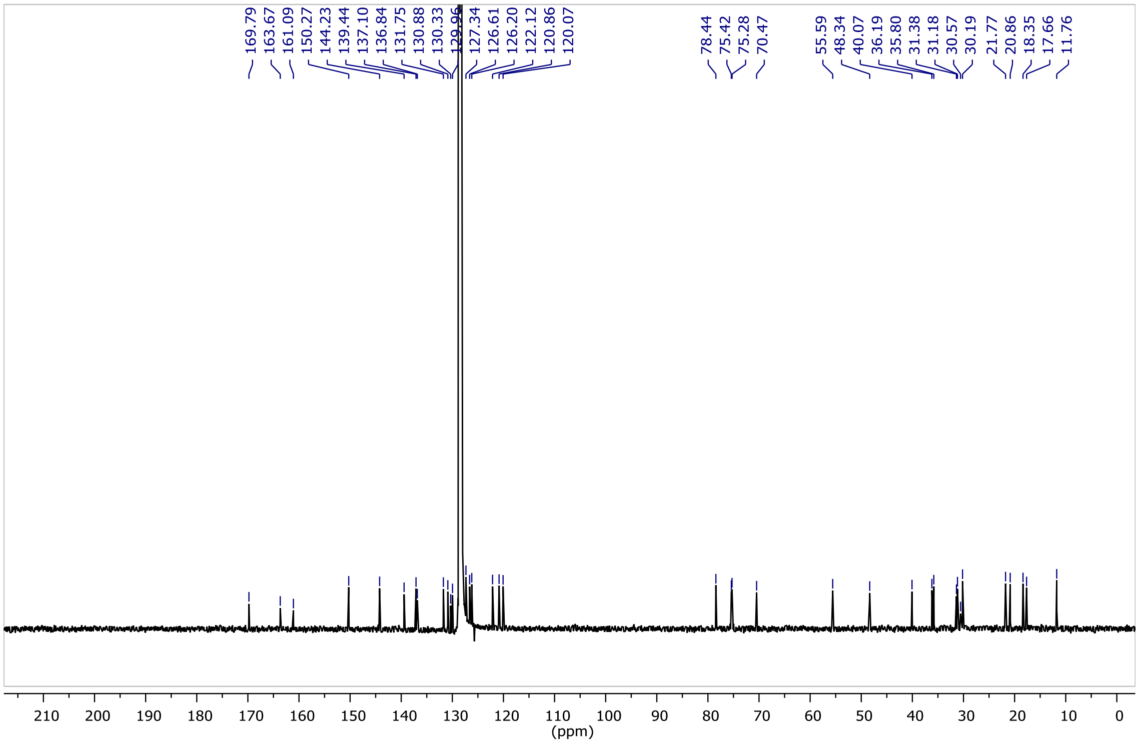

2-PySS(CH$_2$)$_2$NH$_2$ – 2-(pyridin-2-yldisulfaneyl)ethan-1-amine hydrochloride

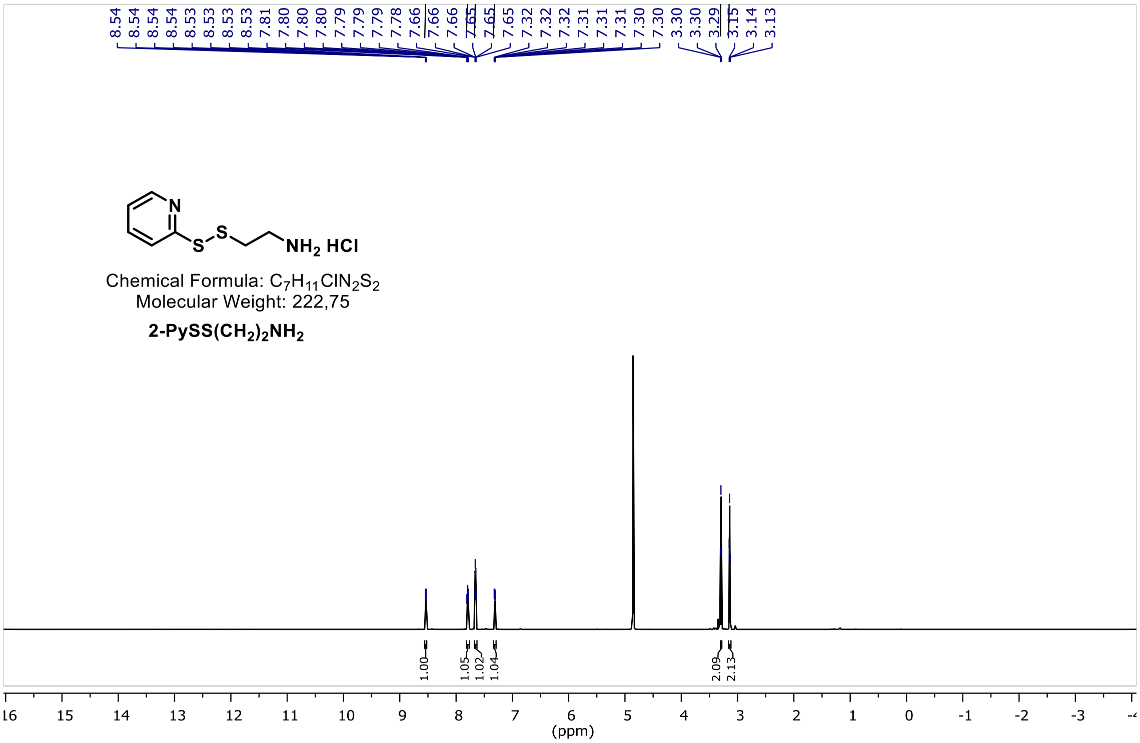

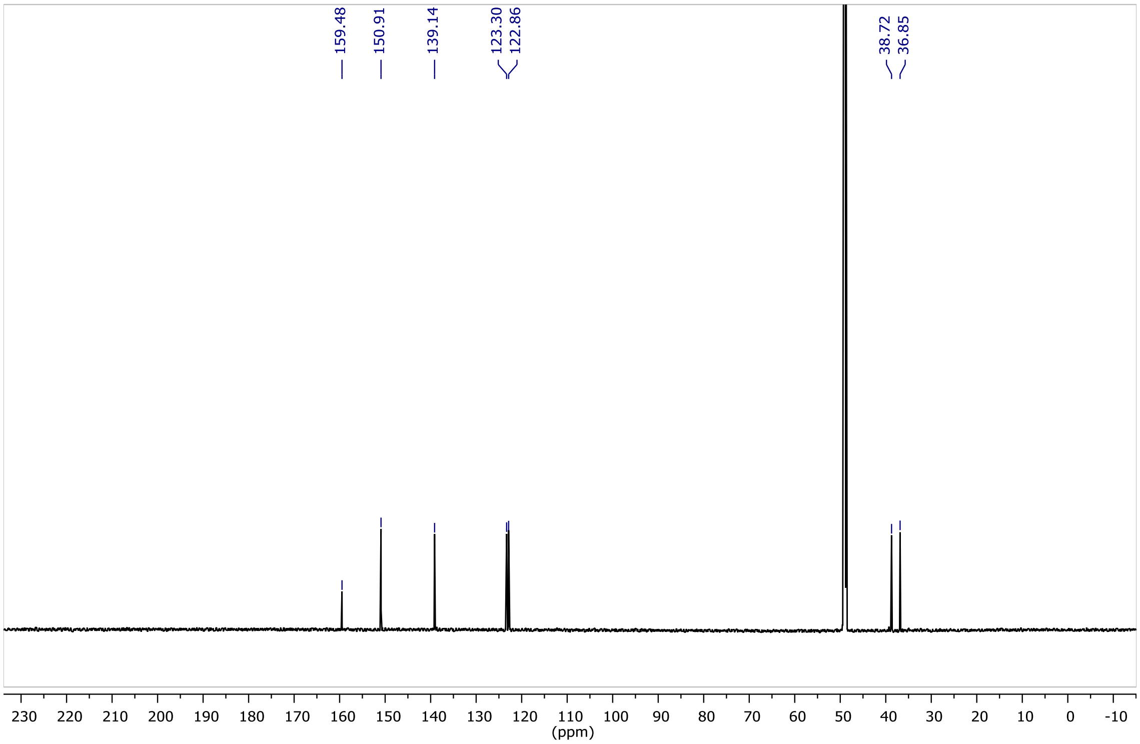

2-PySS(CH2)2NH-(CO)(CH2)2CCH (32) – N-(2-(pyridin-2-yldisulfaneyl)ethyl)pent-4-ynamide

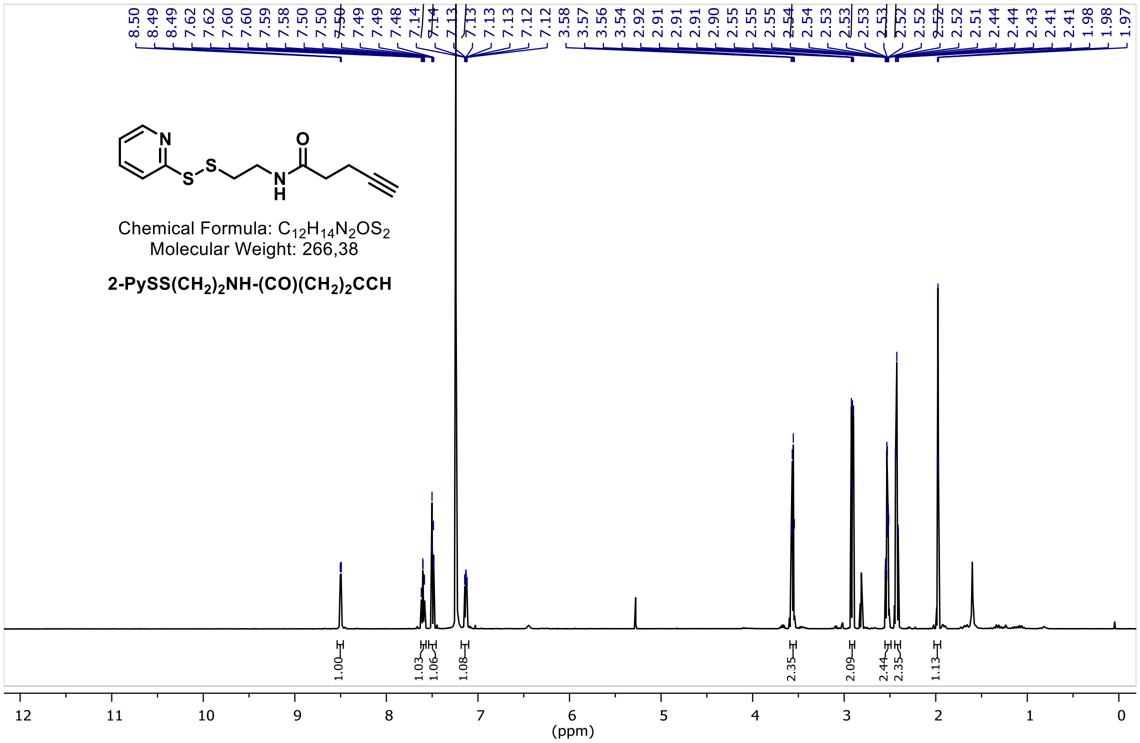

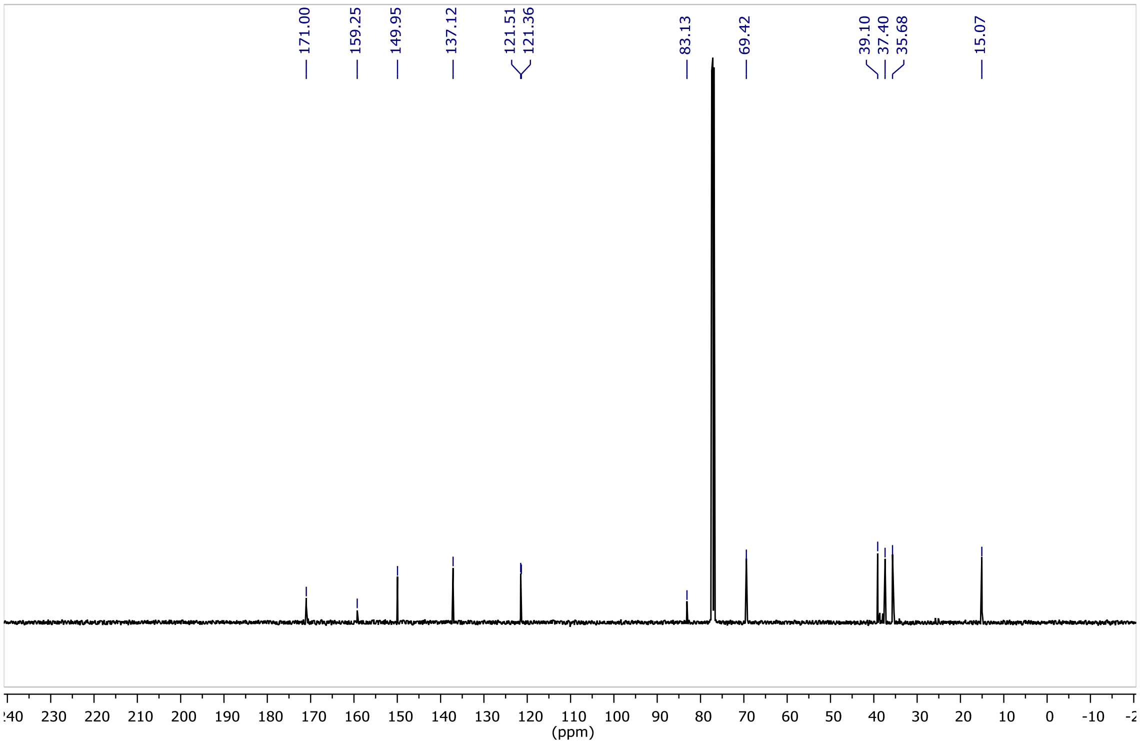

$HO_2C(CH_2)_2SS(CH_2)_2NH$-(CO)$(CH_2)_2CCH$ (30) – 3-((2-(pent-4-ynamido)ethyl)-disulfaneyl)propanoic acid

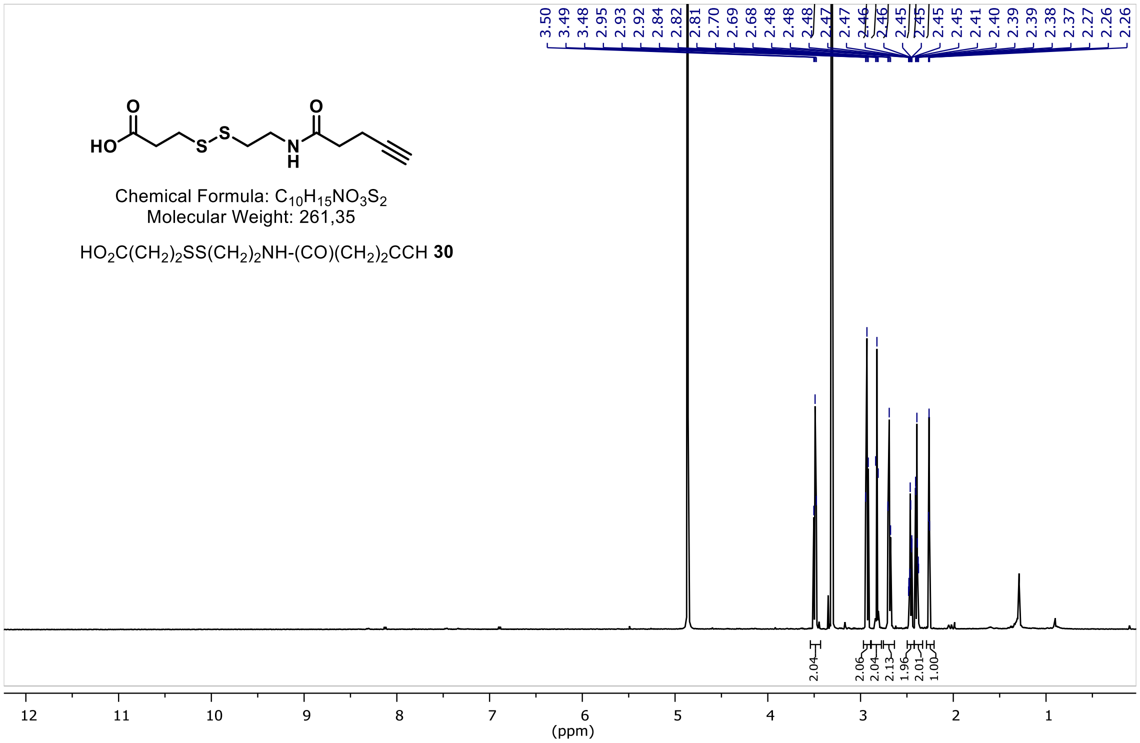

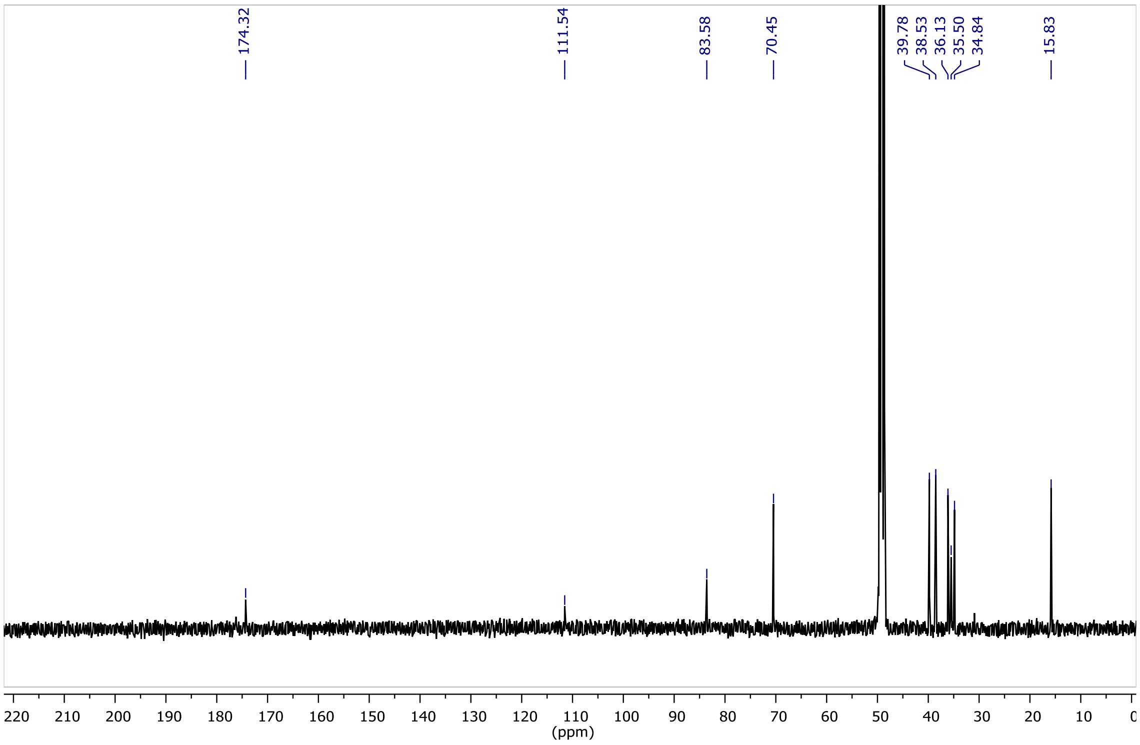

HCC(CH$_2$)$_2$(CO)-NH-(CH$_2$)$_2$SS(CH$_2$)$_2$(CO)-16*R*-Aminoratjadone (29) – N-(2-((3-(((1R,2E,4E,7R,8Z,10E)-1-((2S,4R,5S,6S)-4-hydroxy-5-methyl-6-((E)-prop-1-en-1-yl)tetrahydro-2H-pyran-2-yl)-5,7,9-trimethyl-11-((R)-6-oxo-3,6-dihydro-2H-pyran-2-yl)undeca-2,4,8,10-tetraen-1-yl)amino)-3-oxopropyl)disulfaneyl)-ethyl)pent-4-ynamide

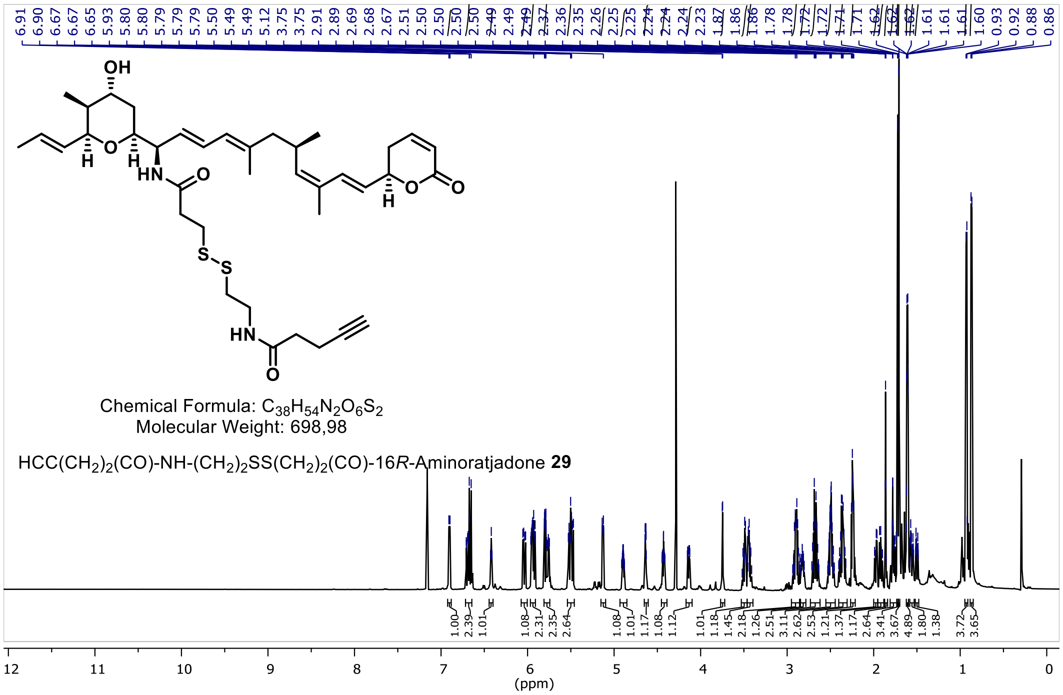

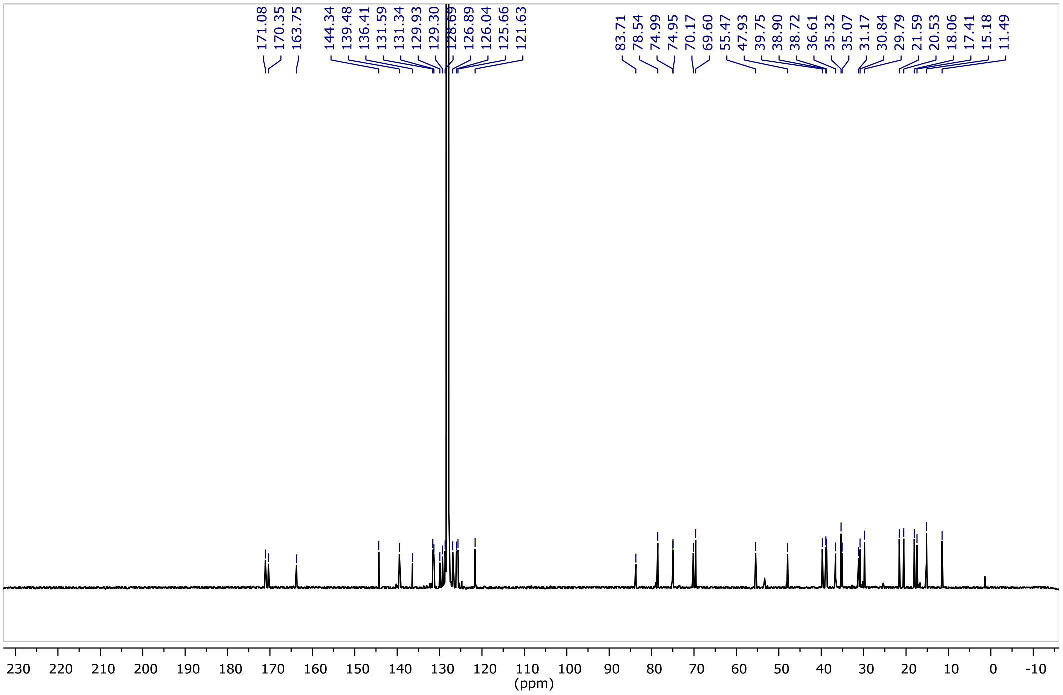

19-Aminoratjadone (33) - (6R)-6-((1E,3Z,5R,7E,9E,11R)-11-((2S,5S,6S)-4-amino-5-methyl-6-((E)-prop-1-en-1-yl)tetrahydro-2H-pyran-2-yl)-11-hydroxy-5,7-dimethylundeca-1,3,7,9-tetraen-1-yl)-5,6-dihydro-2H-pyran-2-one

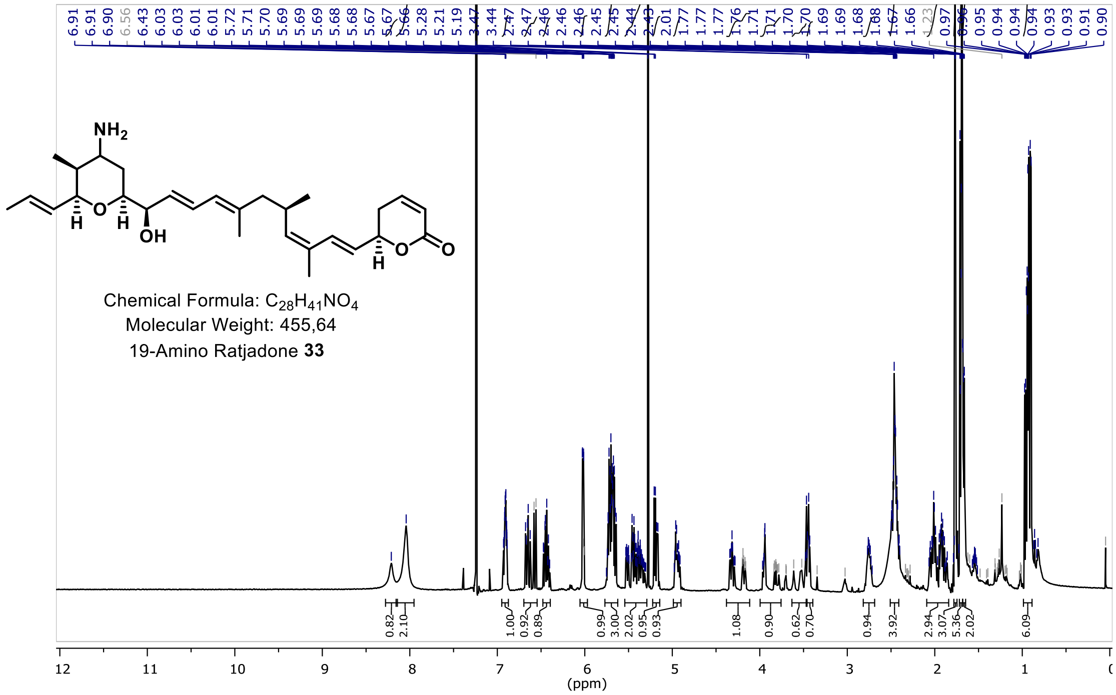

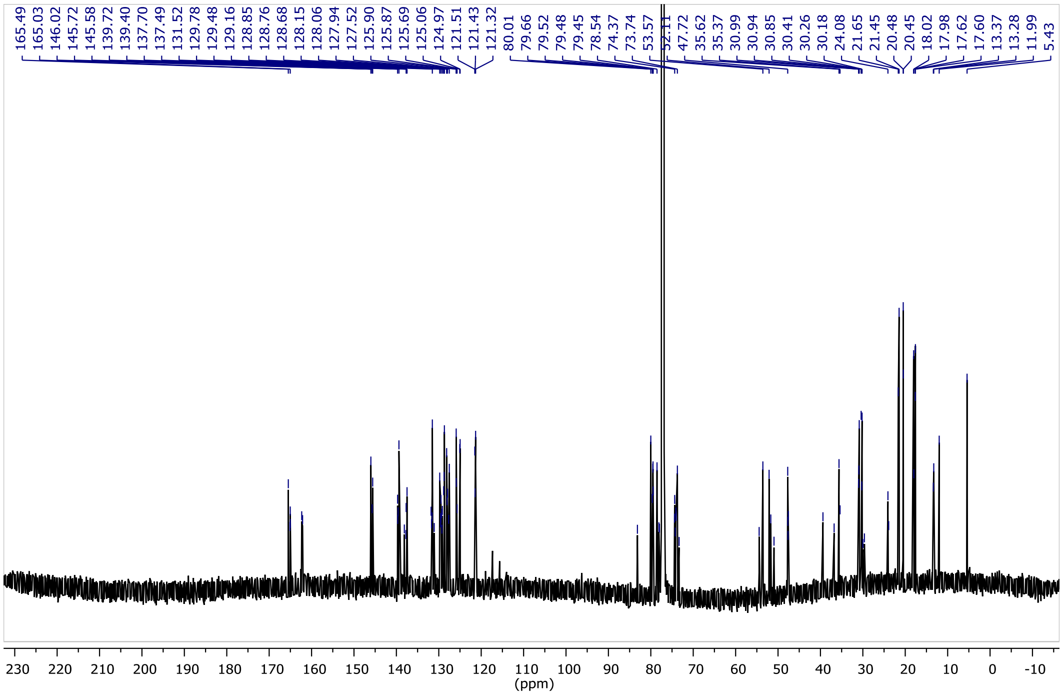

Compound 34 - But-3-yn-1-yl ((2S,3S,6S)-6-((1S,2E,4E,7R,8Z,10E)-1-hydroxy-5,7,9-trimethyl-11-((R)-6-oxo-3,6-dihydro-2H-pyran-2-yl)undeca-2,4,8,10-tetraen-1-yl)-3-methyl-2-((E)-prop-1-en-1-yl)tetrahydro-2H-pyran-4-yl)carbamate

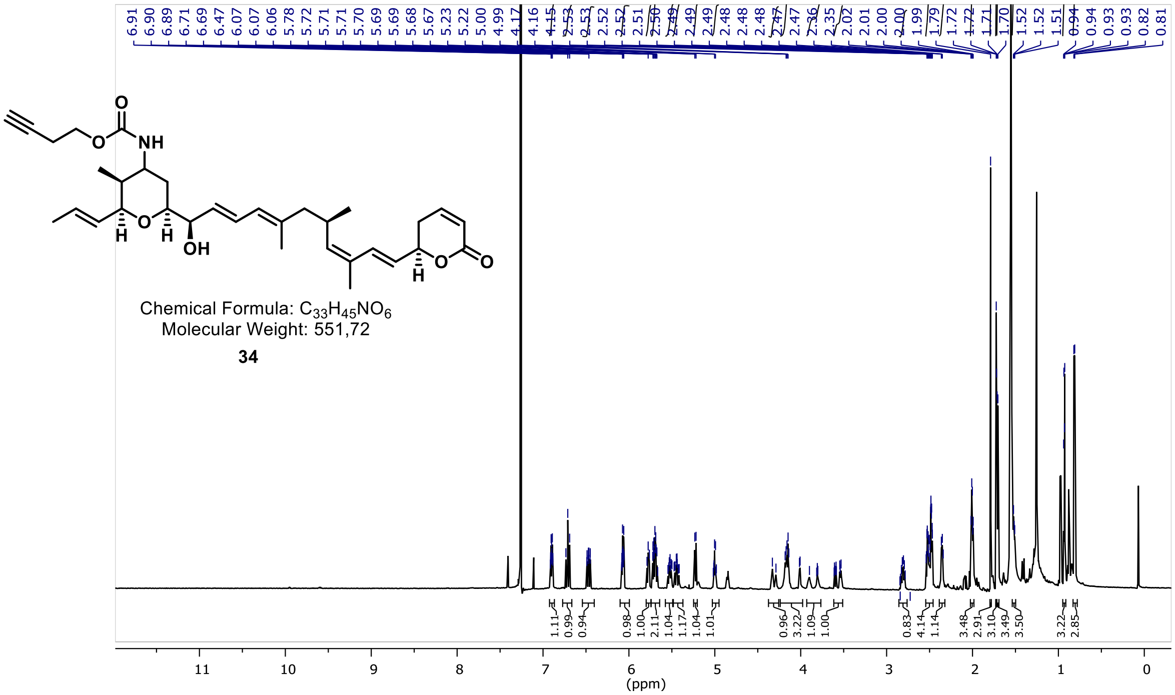

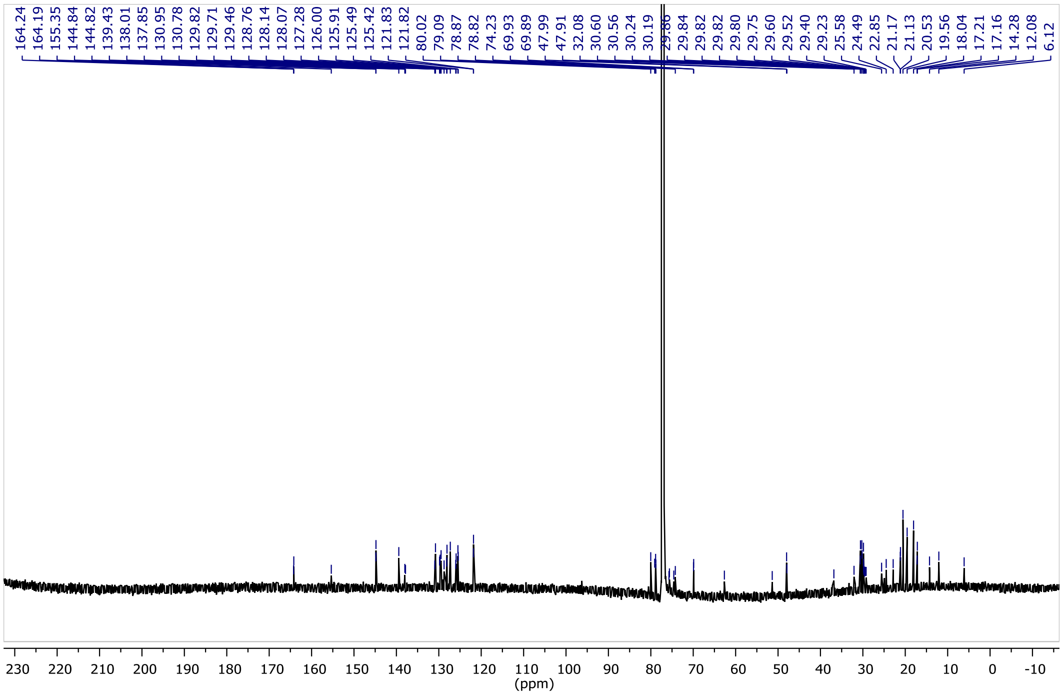

N-Propargyl-19-Aminoratjadone (35) - (6R)-6-((1E,3Z,5R,7E,9E,11R)-11-hydroxy-3,5,7-trimethyl-11-((2S,5S,6S)-5-methyl-6-((E)-prop-1-en-1-yl)-4-(prop-2-yn-1-ylamino)tetrahydro-2H-pyran-2-yl)undeca-1,3,7,9-tetraen-1-yl)-5,6-dihydro-2H-pyran-2-one

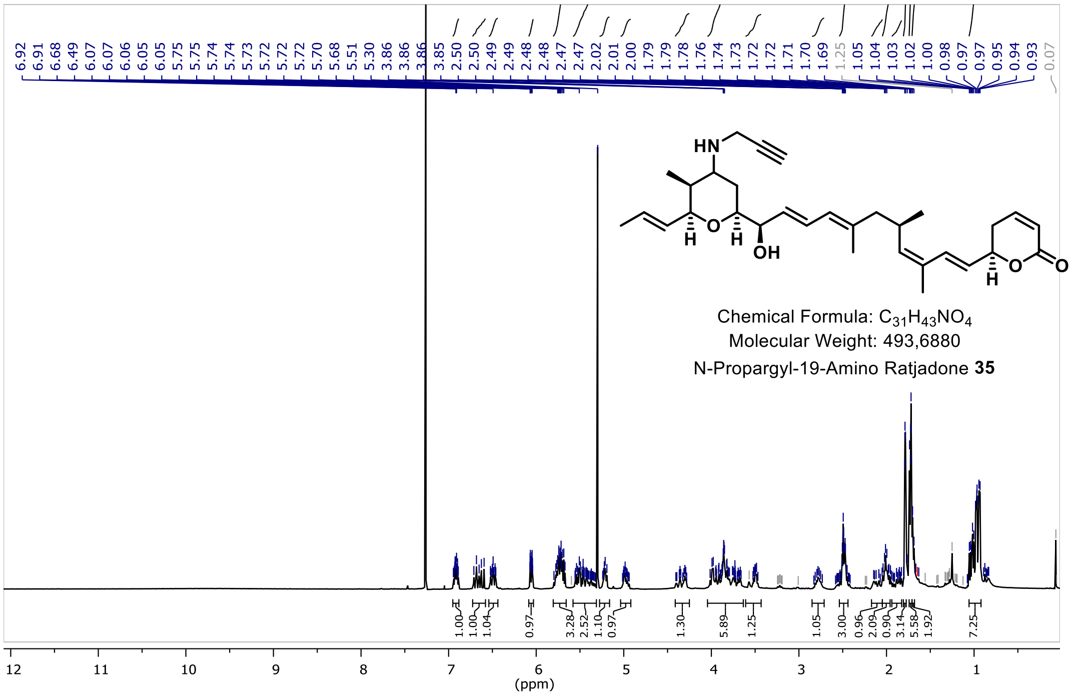

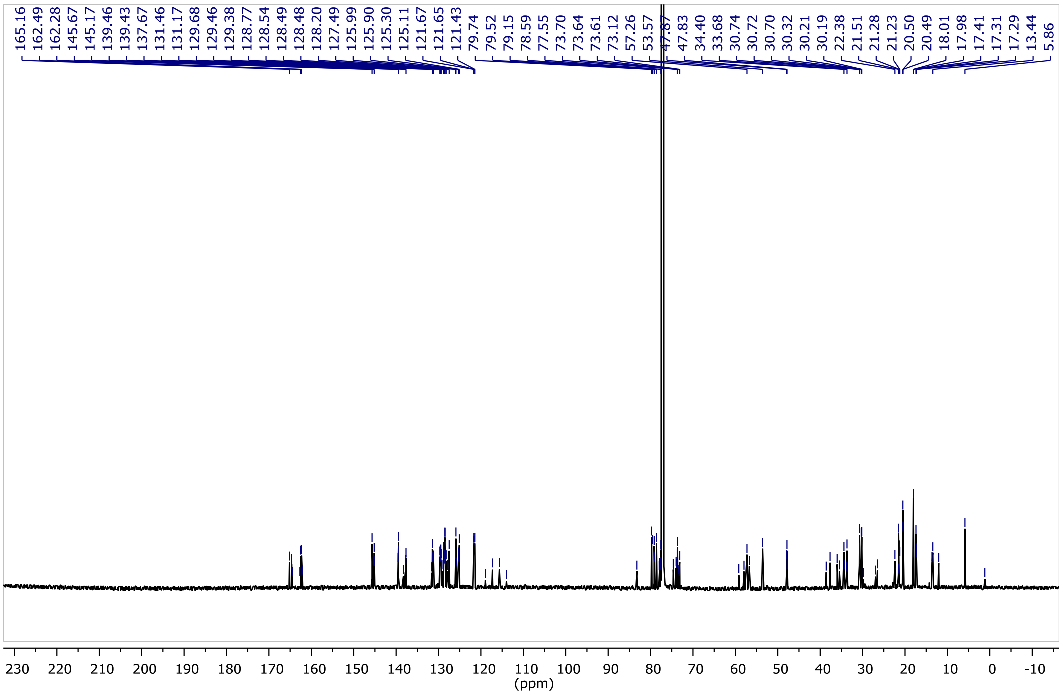

Methyl N²-(((9H-fluoren-9-yl)methoxy)carbonyl)-N⁶-(tert-butoxycarbonyl)-L-lysinate (37)

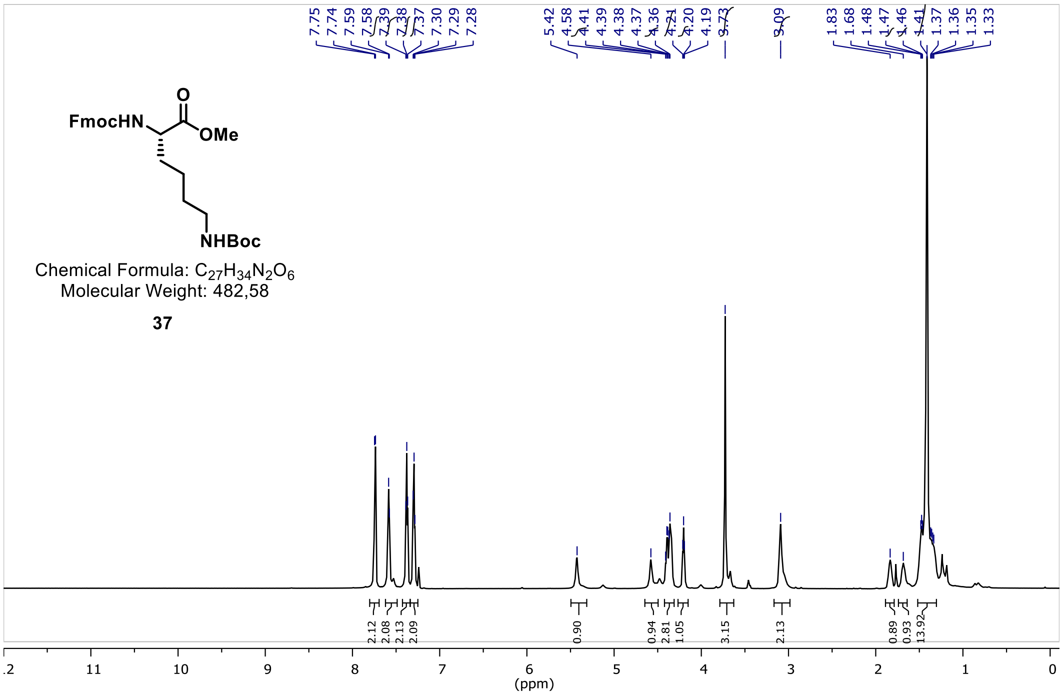

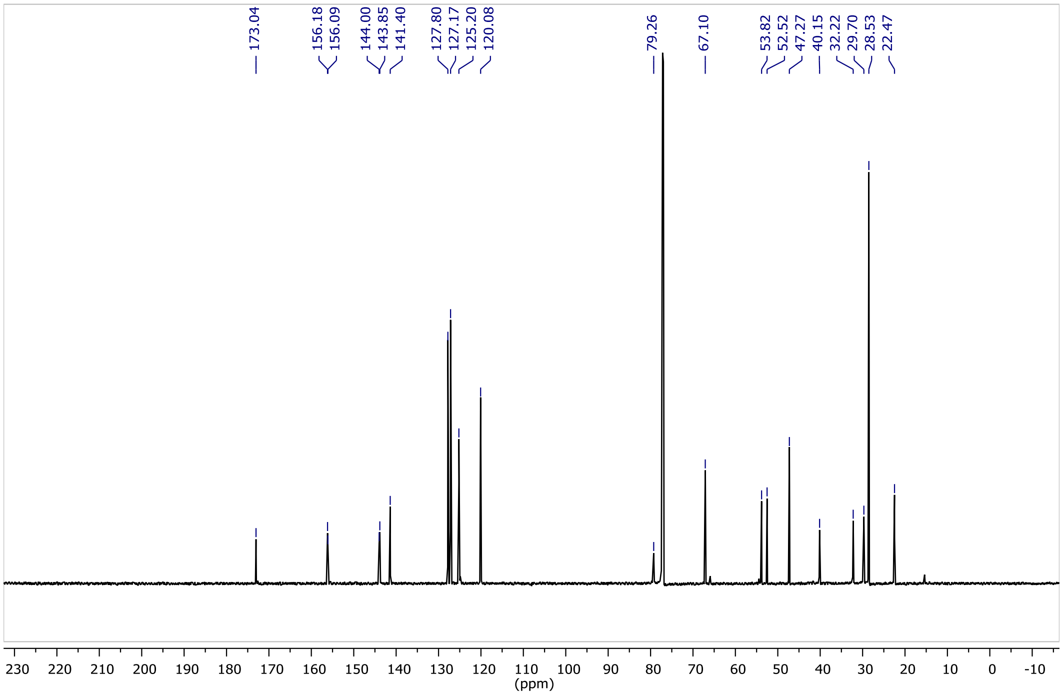

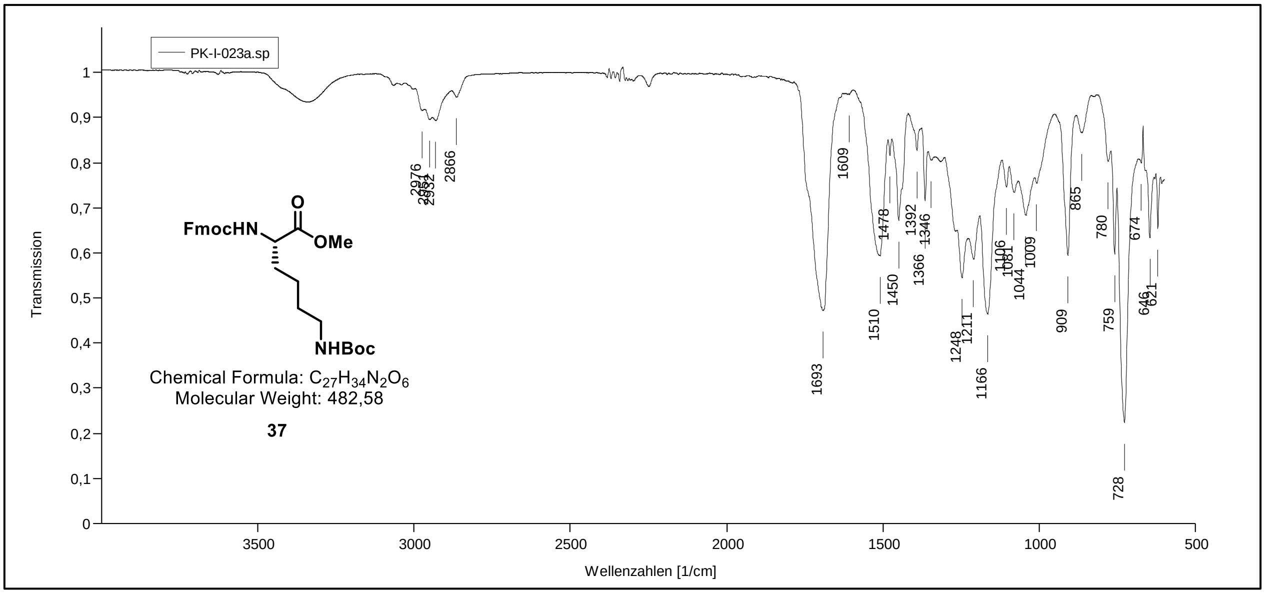
PK-I-023a.sp
Transmission
Wellenzahlen [1/cm]
FmocHN
O
OMe
NHBoc
Chemical Formula: $C_{27}H_{34}N_2O_6$
Molecular Weight: 482,58
37
2976
2952
2932
2866
1693
1609
1510
1478
1450
1392
1366
1346
1248
1211
1166
1106
1081
1044
1009
909
865
780
759
728
674
646
621

Methyl N[2]-((S)-4-((((9H-fluoren-9-yl)methoxy)carbonyl)amino)-5-(tert-butoxy)-5-oxopentanoyl)-N[6]-(tert-butoxycarbonyl)-L-lysinate (38)

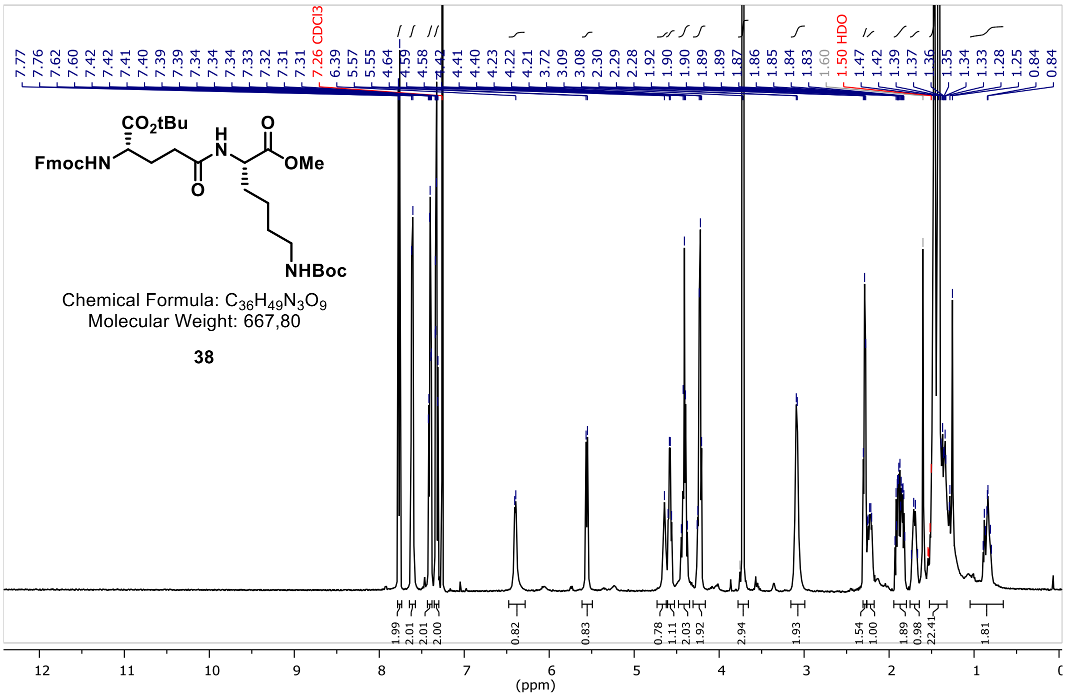

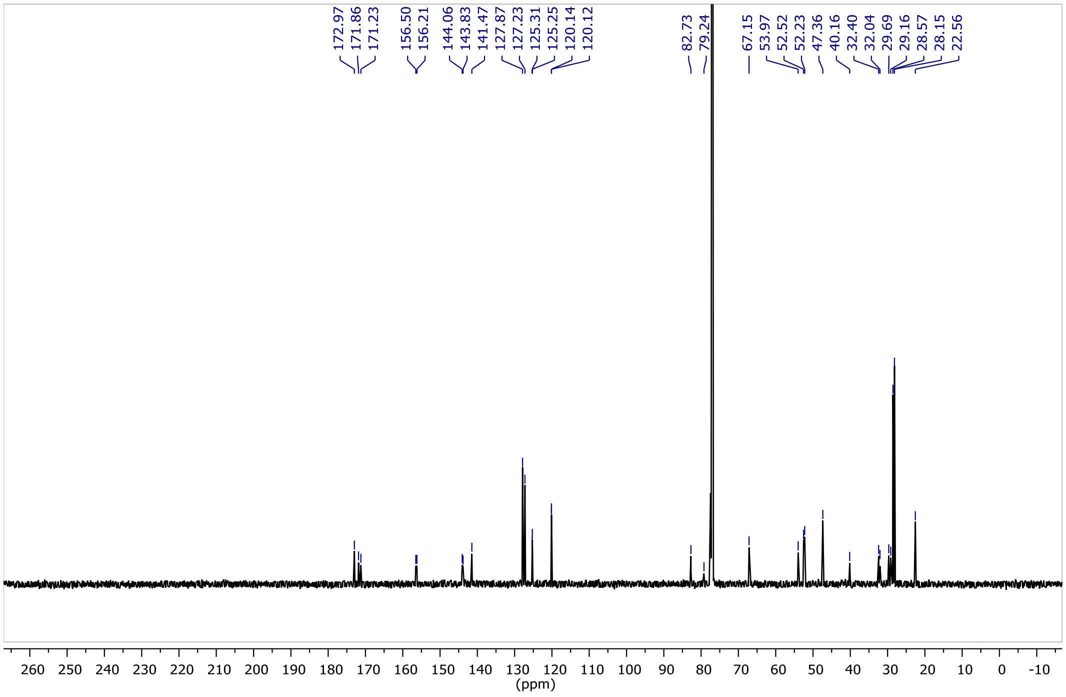

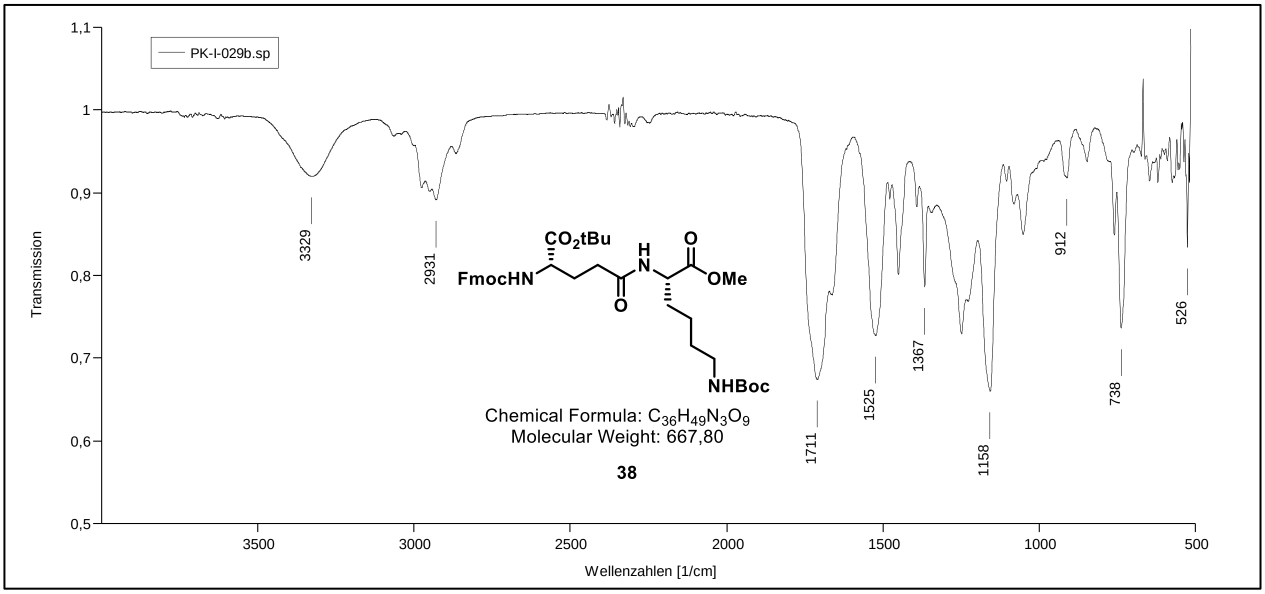
PK-I-029b.sp
Transmission
Wellenzahlen [1/cm]
1,1
1
0,9
0,8
0,7
0,6
0,5
3500
3000
2500
2000
1500
1000
500
3329
2931
1711
1525
1367
1158
912
738
526
CO2tBu
FmocHN
H
N
O
OMe
O
NHBoc
Chemical Formula: C36H49N3O9
Molecular Weight: 667,80
38

Methyl N[2]-(((9H-fluoren-9-yl)methoxy)carbonyl)-N[5]-((S)-6-(4-azidobenzamido)-1-methoxy-1-oxohexan-2-yl)-L-glutamine (39)

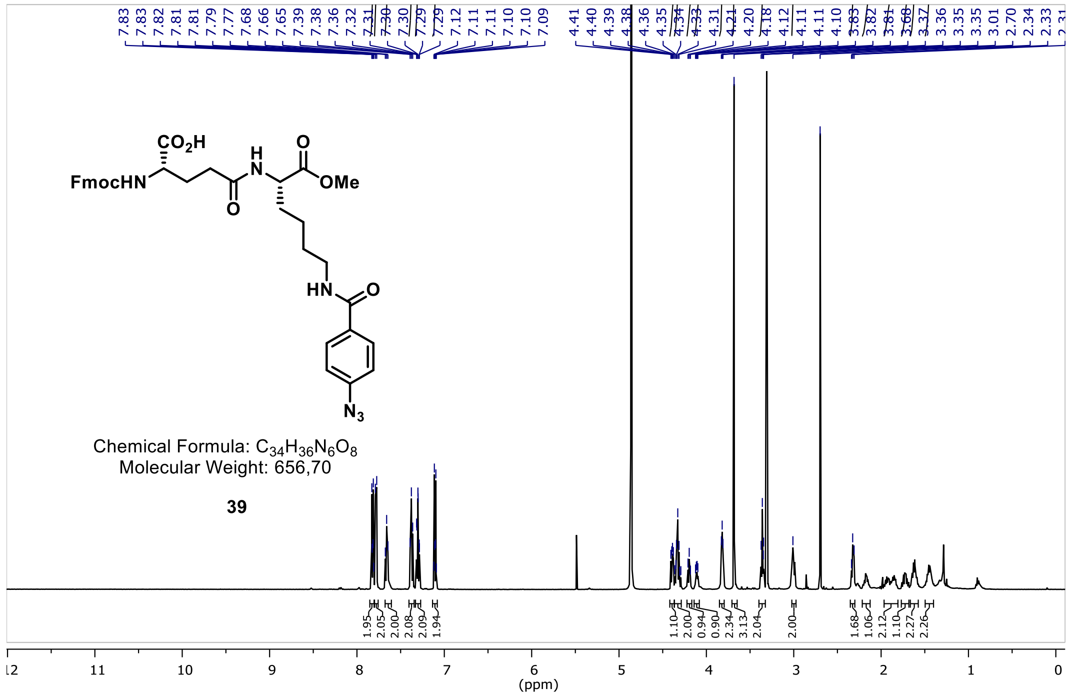

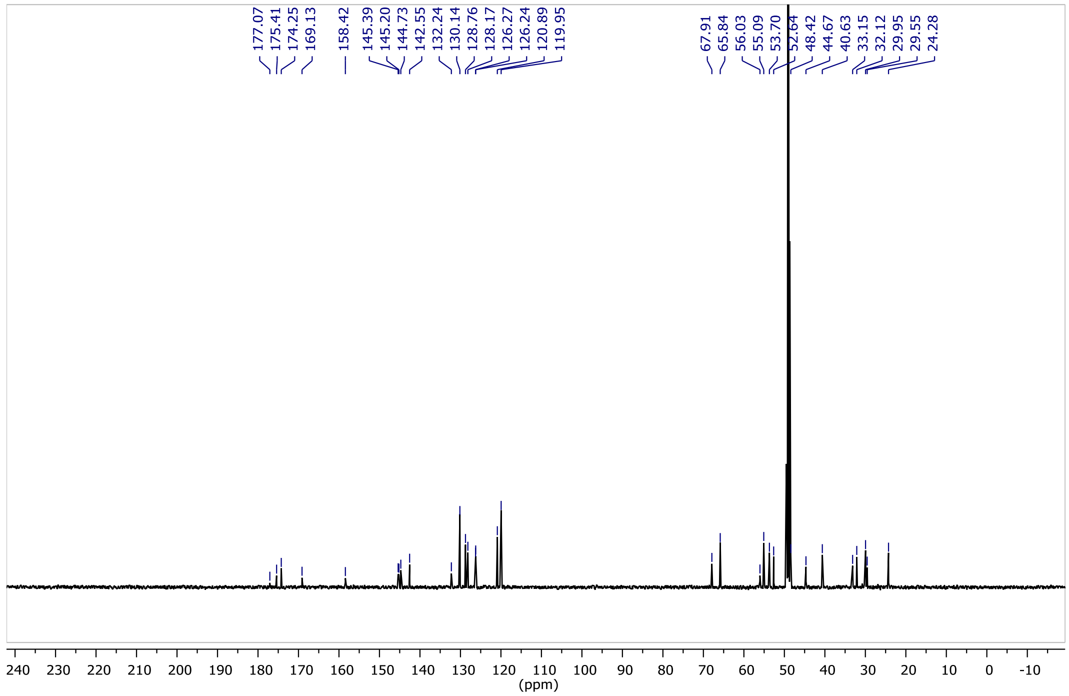

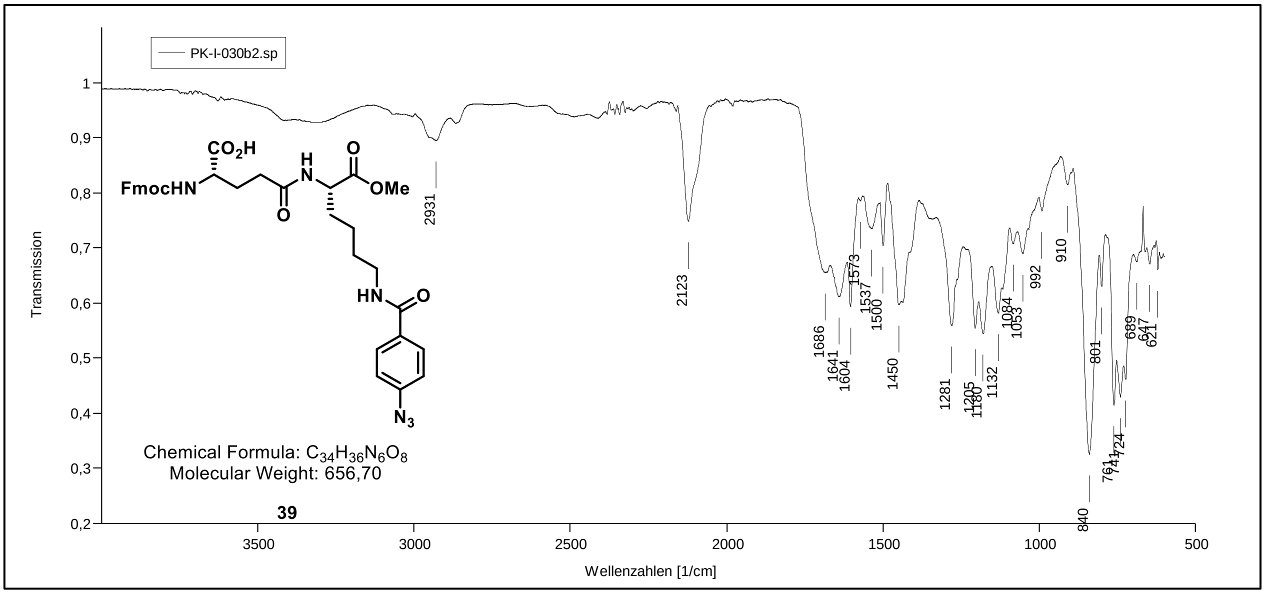
PK-I-030b2.sp
Transmission
Wellenzahlen [1/cm]
FmocHN
CO2H
OMe
HN
N3
Chemical Formula: C34H36N6O8
Molecular Weight: 656,70
39
2931
2123
1686
1641
1604
1573
1537
1500
1450
1281
1205
1180
1132
1084
1053
992
910
840
801
761
747
724
689
647
621

Fa-N$_3$-1 - N^2-(4-(((2-amino-4-hydroxypteridin-6-yl)methyl)amino)benzoyl)-N^5-((S)-4-(((S)-6-(4-azidobenzamido)-1-methoxy-1-oxohexan-2-yl)amino)-1-carboxy-4-oxobutyl)-L-glutamine

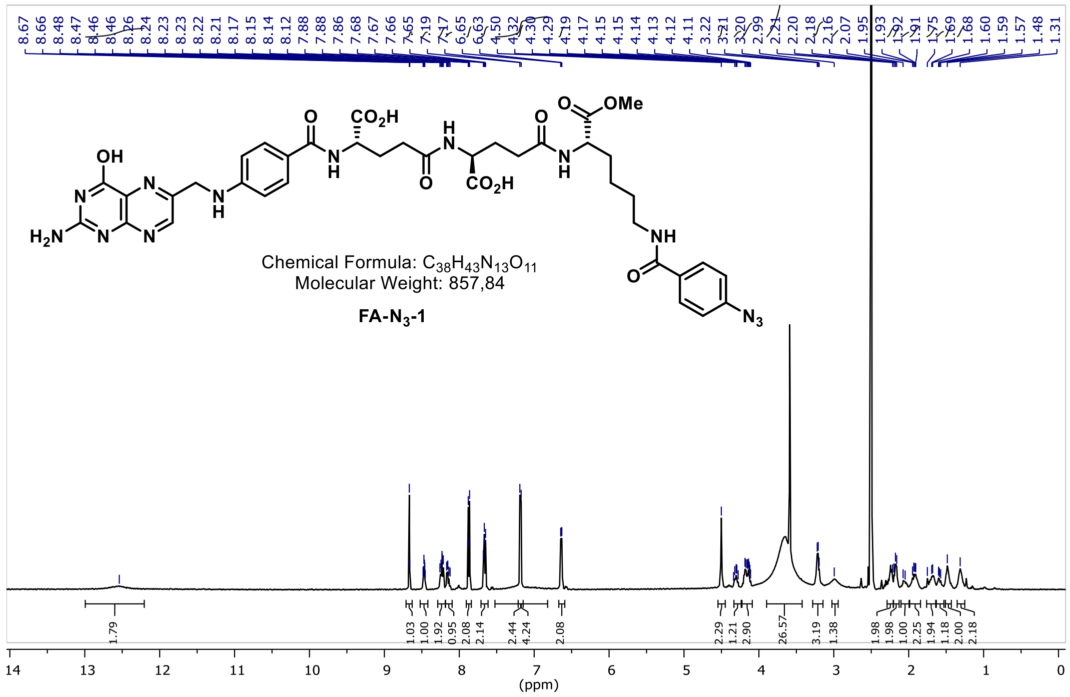

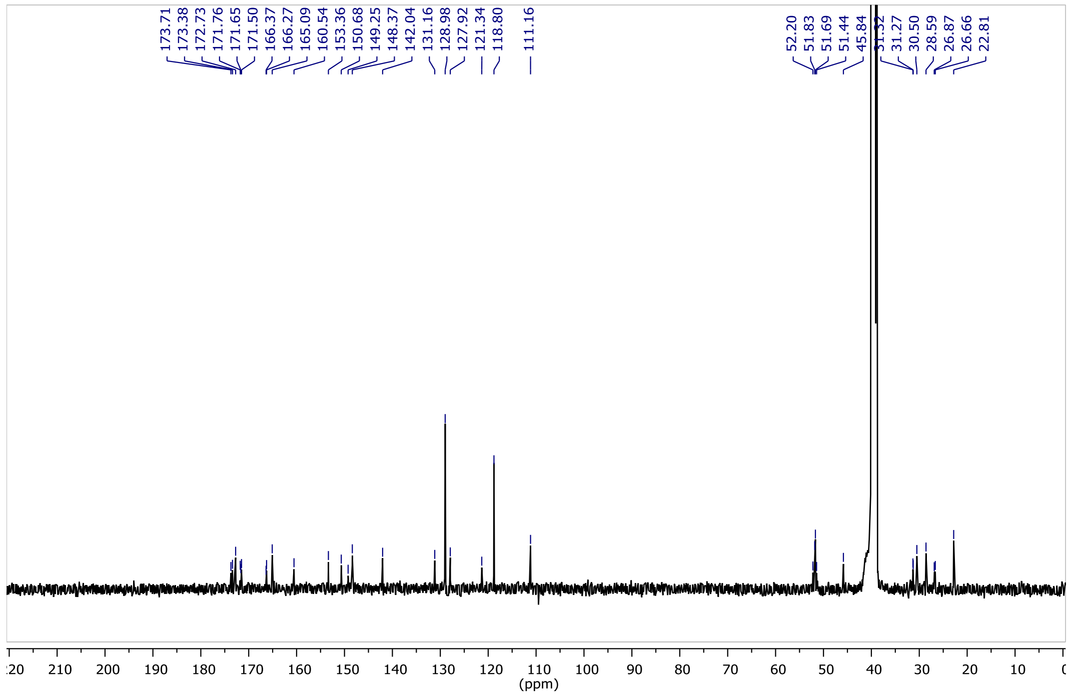

Methyl N[2]-(((9H-fluoren-9-yl)methoxy)carbonyl)-N[6]-(4-azidobenzoyl)-L-lysinate (40)

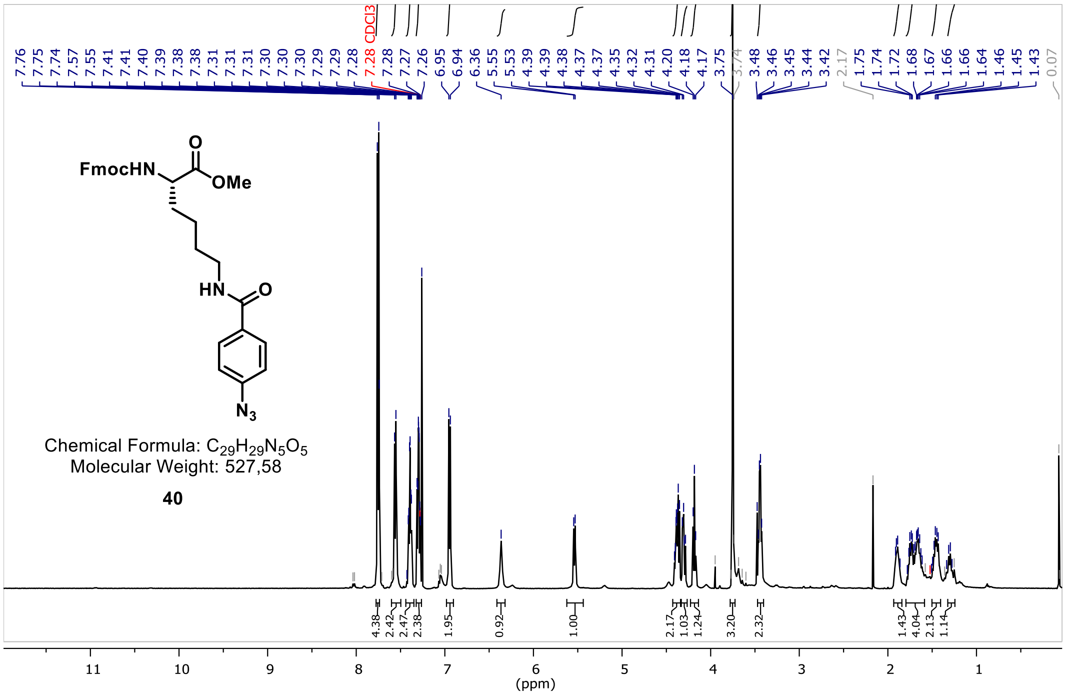

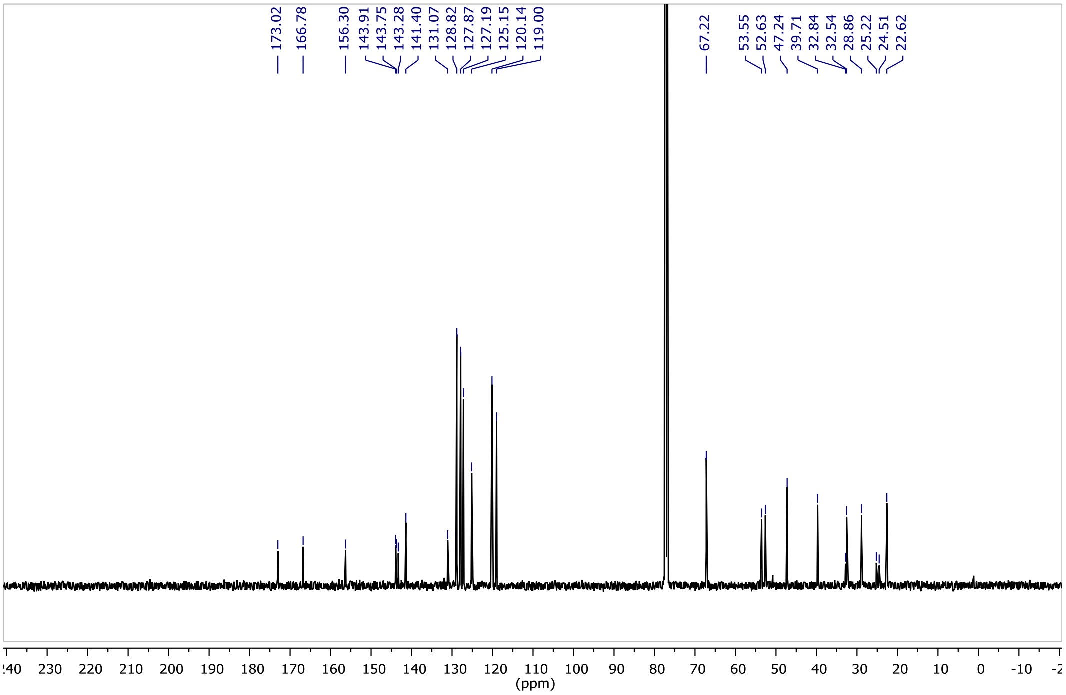

Fmoc-(Asp(OtBu))$_3$-OH - (5S,8S,11S)-5,8,11-tris(2-(tert-butoxy)-2-oxoethyl)-1-(9H-fluoren-9-yl)-3,6,9-trioxo-2-oxa-4,7,10-triazadodecan-12-oic acid

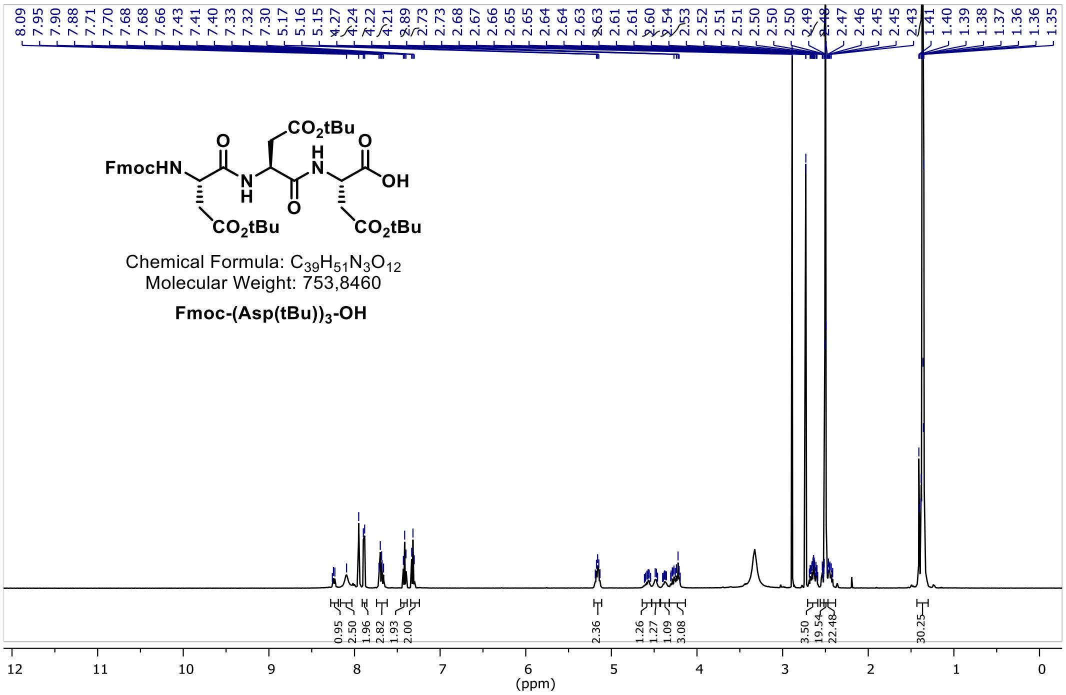

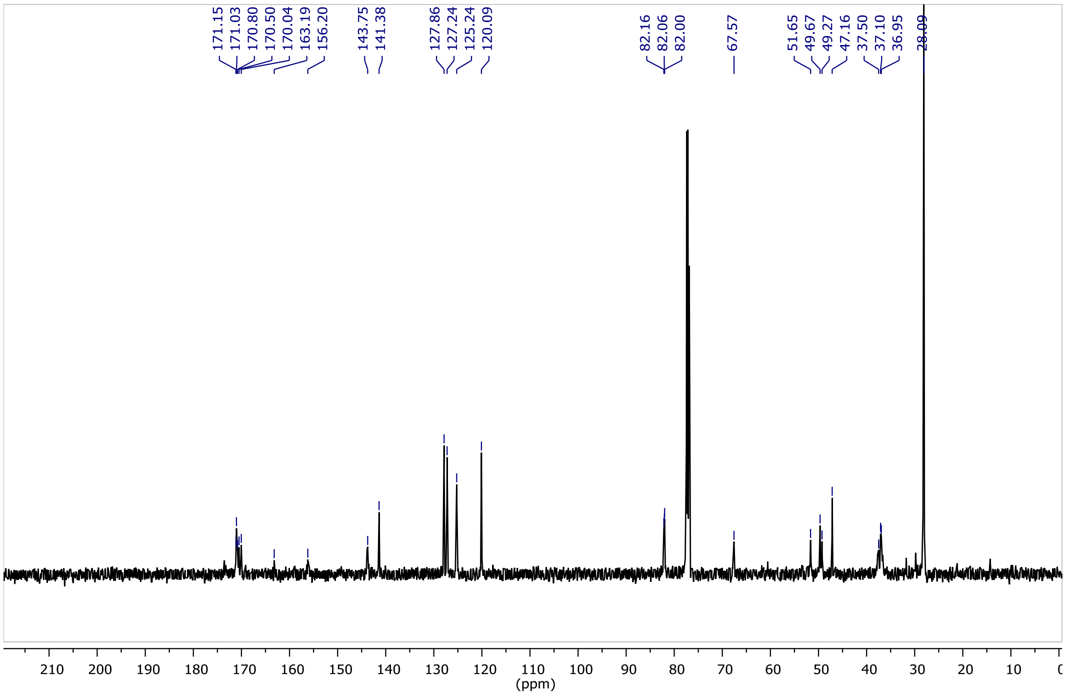

(7S,10S,13S,16S)-16-((((9H-fluoren-9-yl)methoxy)carbonyl)amino)-1-(4-azidophenyl)-10,13-bis(carboxymethyl)-7-(methoxycarbonyl)-1,9,12,15-tetraoxo-2,8,11,14-tetraazaoctadecan-18-oic acid (41)

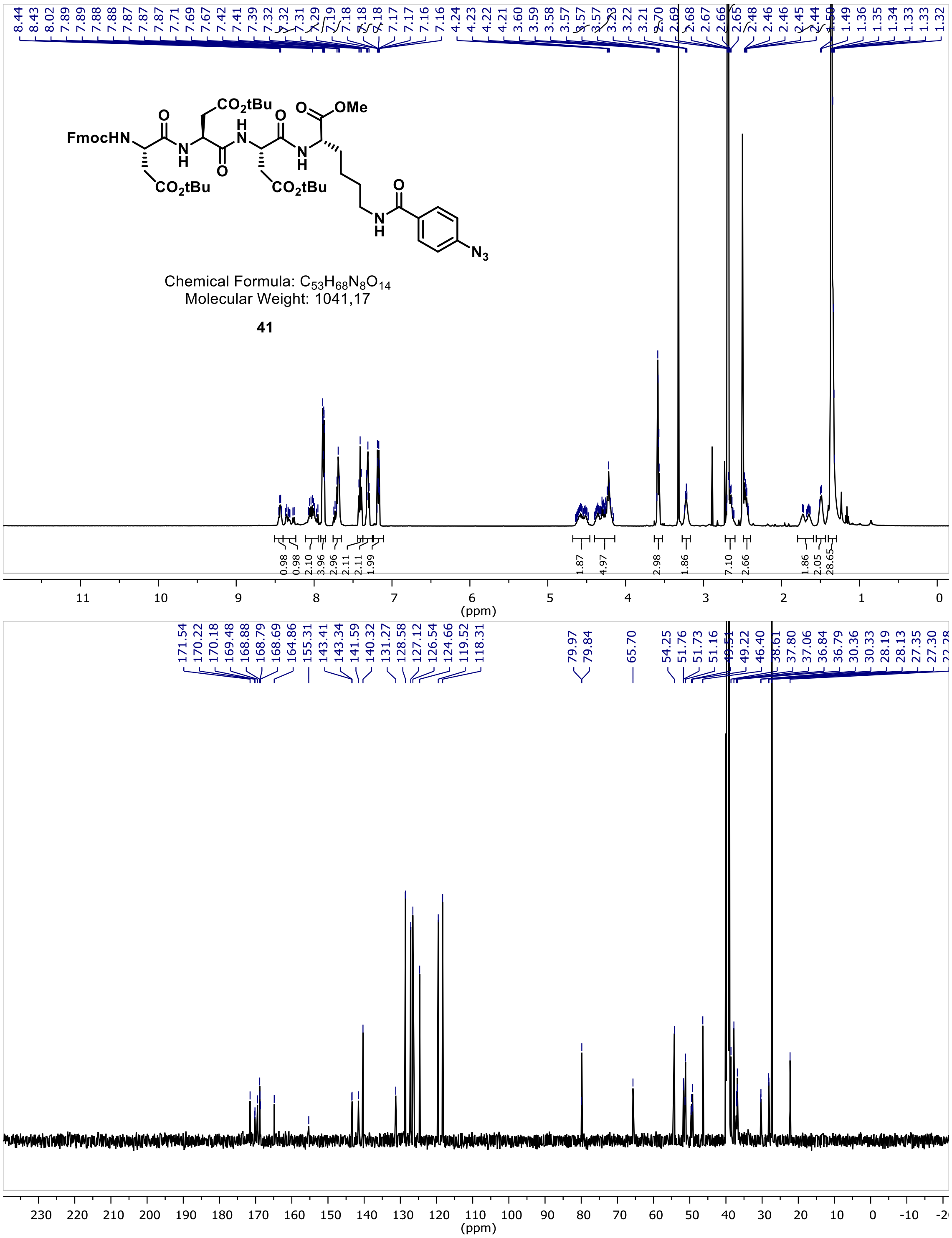

FA-N$_3$-2 - N^2-(4-(((2-amino-4-hydroxypteridin-6-yl)methyl)amino)benzoyl)-N^5-((S)-1-(((S)-1-(((S)-1-(((S)-6-(4-azidobenzamido)-1-methoxy-1-oxohexan-2-yl)amino)-3-carboxy-1-oxopropan-2-yl)amino)-3-carboxy-1-oxopropan-2-yl)amino)-3-carboxy-1-oxopropan-2-yl)-L-glutamine

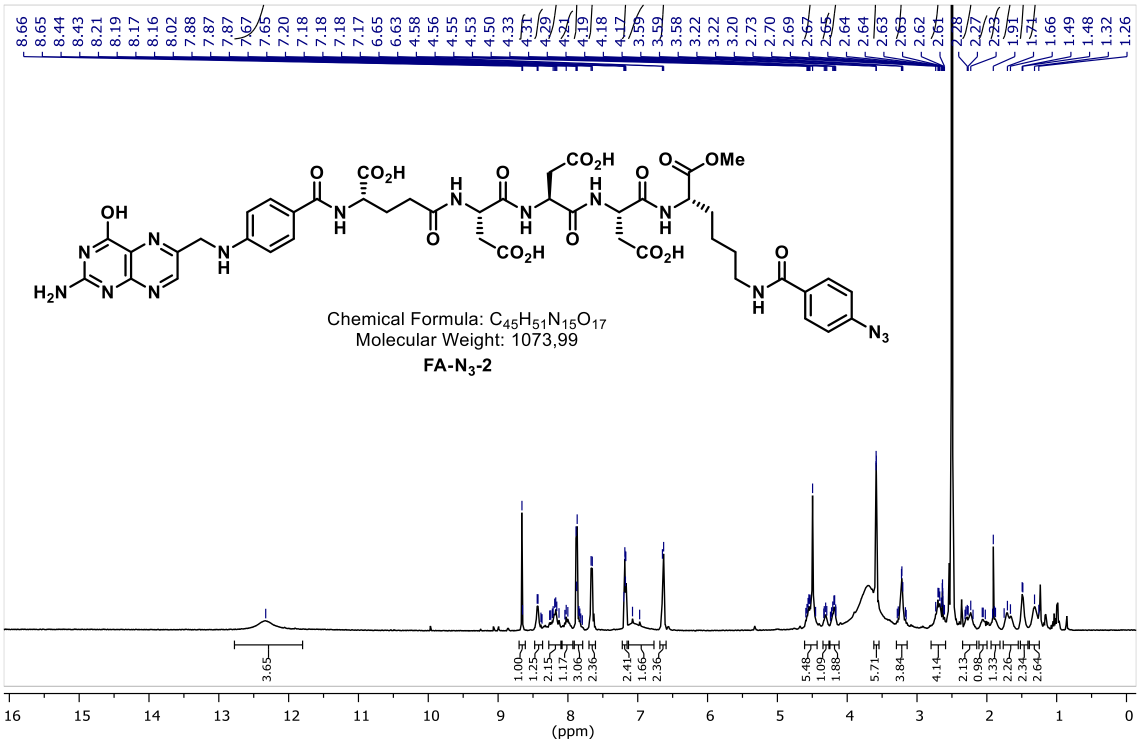

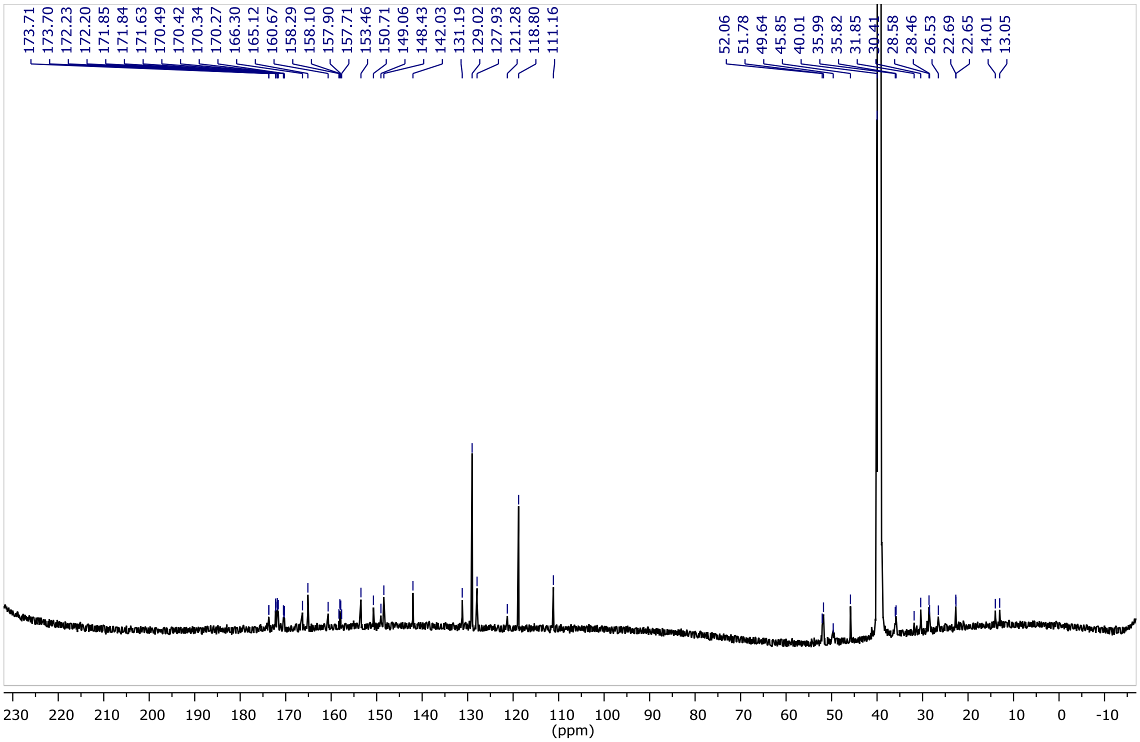

N[2]-(((9H-fluoren-9-yl)methoxy)carbonyl)-N[6]-(4-azidobenzoyl)-L-lysine

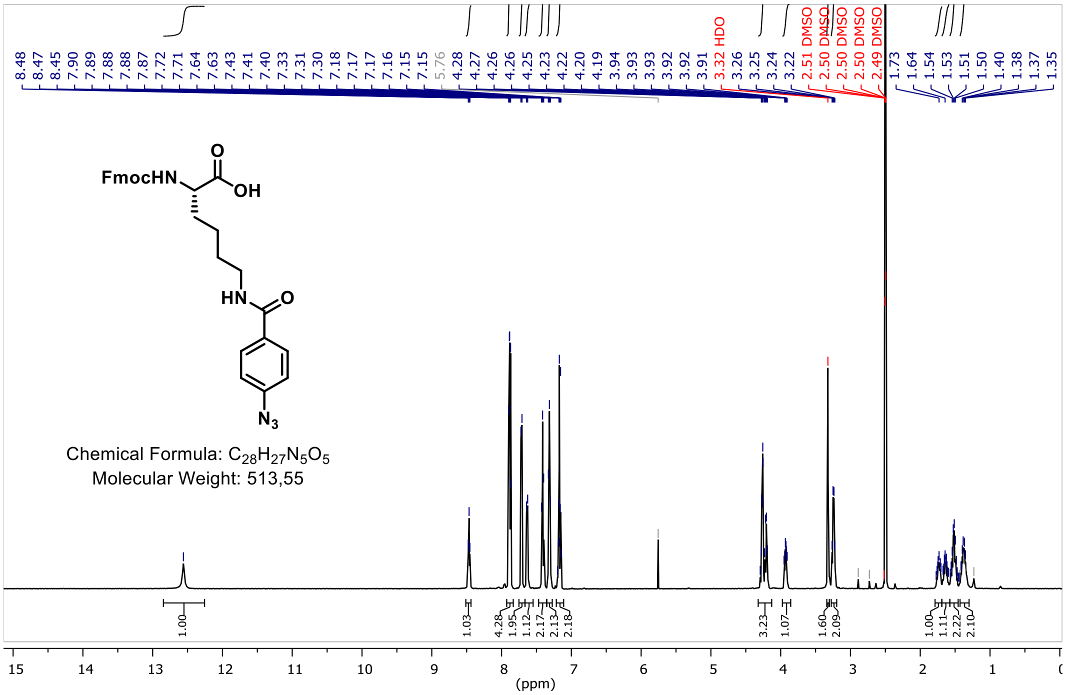

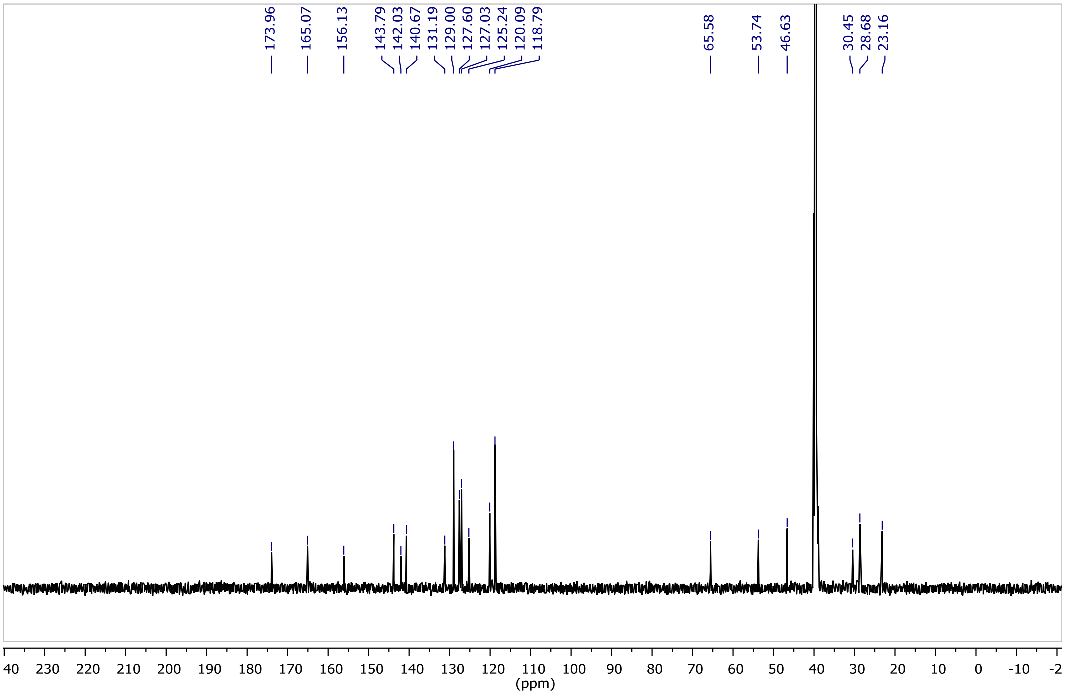

FA-N_3-3 - N^2-((S)-4-(4-(((2-amino-4-hydroxypteridin-6-yl)methyl)amino)benzamido)-4-carboxybutanoyl)-D-aspartyl-L-aspartyl-L-aspartyl-N^6-(4-azidobenzoyl)-L-lysine

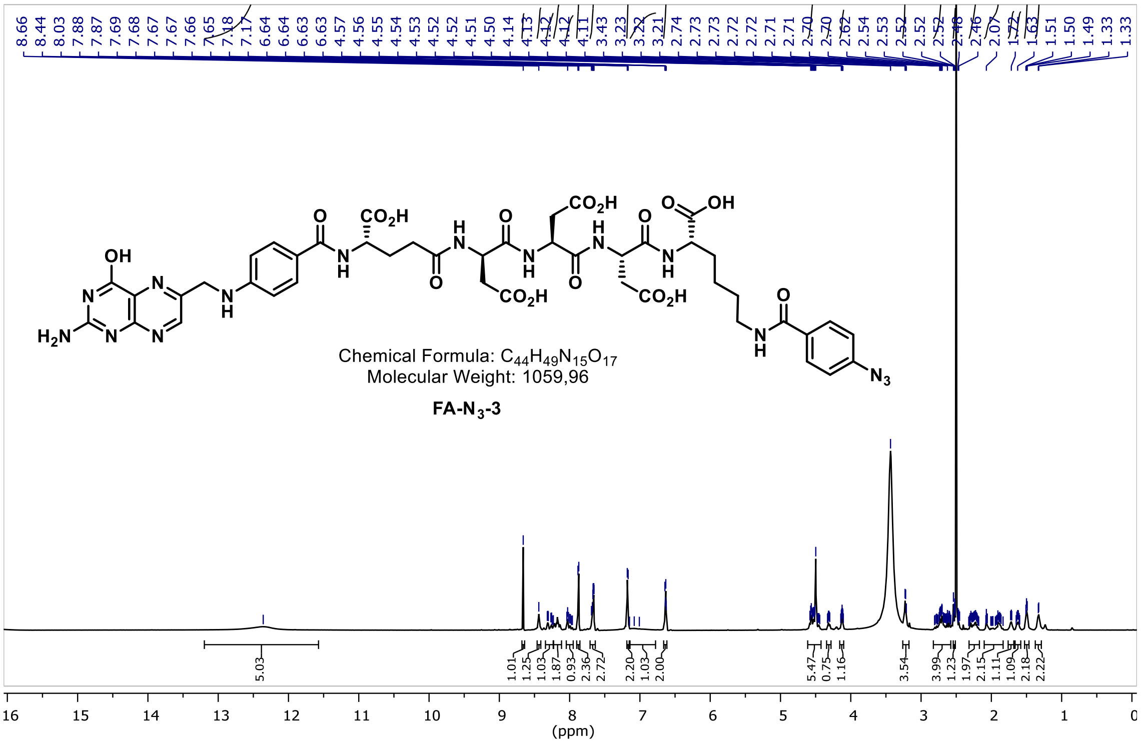

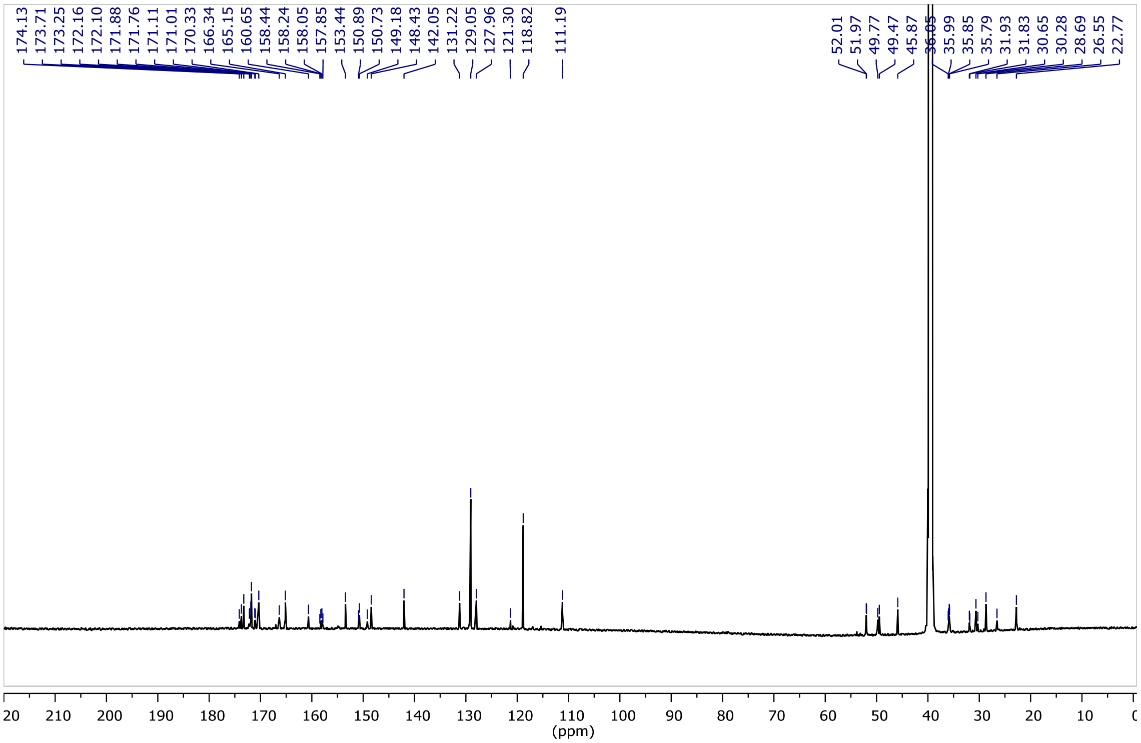

FA-N_3-4 - N^2-N^2-((S)-4-(4-(((2-amino-4-hydroxypteridin-6-yl)methyl)amino)benzamido)-4-carboxybutanoyl)-D-aspartyl-D-aspartyl-N^6-(4-azidobenzoyl)-L-lysyl-L-aspartyl-L-aspartyl-N^6-(4-azidobenzoyl)-L-lysine

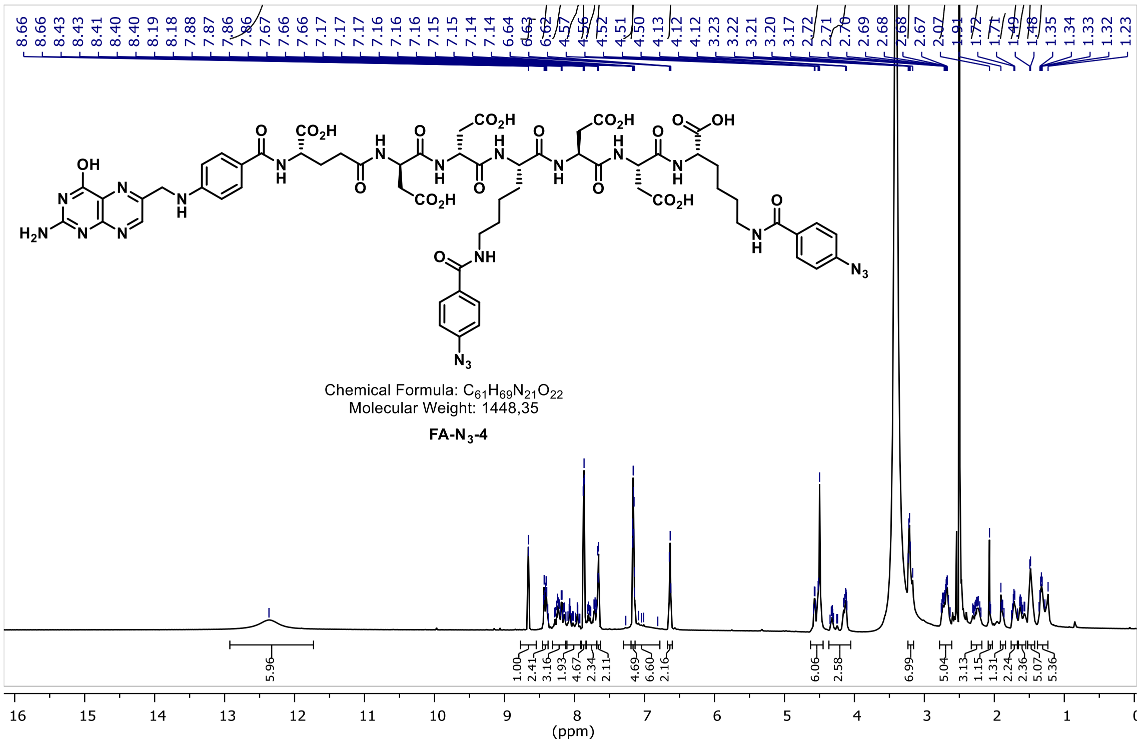

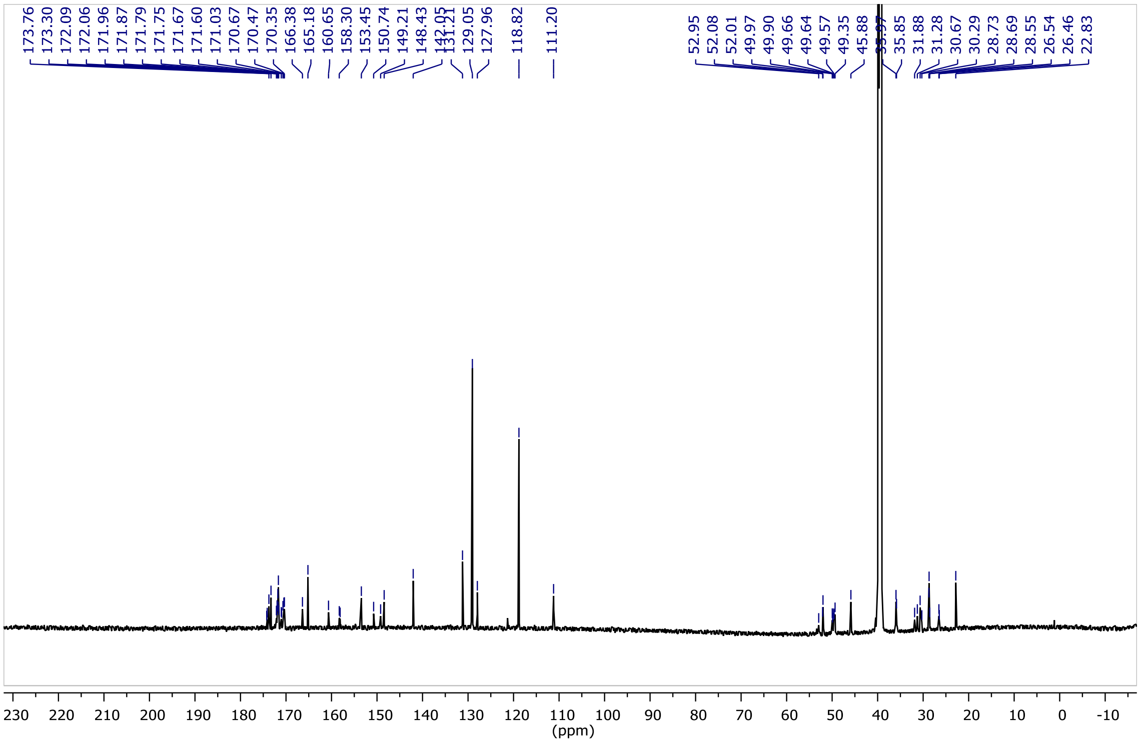

FA-N$_3$-5 - N^2-(4-(((2-amino-4-hydroxypteridin-6-yl)methyl)amino)benzoyl)-N5-((R)-1-(((R)-1-(((R)-1-(((R)-1-(((R)-1-(((S)-4-azido-1-carboxybutyl)amino)-3-carboxy-1-oxopropan-2-yl)amino)-3-carboxy-1-oxopropan-2-yl)amino)-3-carboxy-1-oxopropan-2-yl)amino)-3-carboxy-1-oxopropan-2-yl)amino)-3-carboxy-1-oxopropan-2-yl)-L-glutamine ^{1}H (top), ^{13}C (bottom)

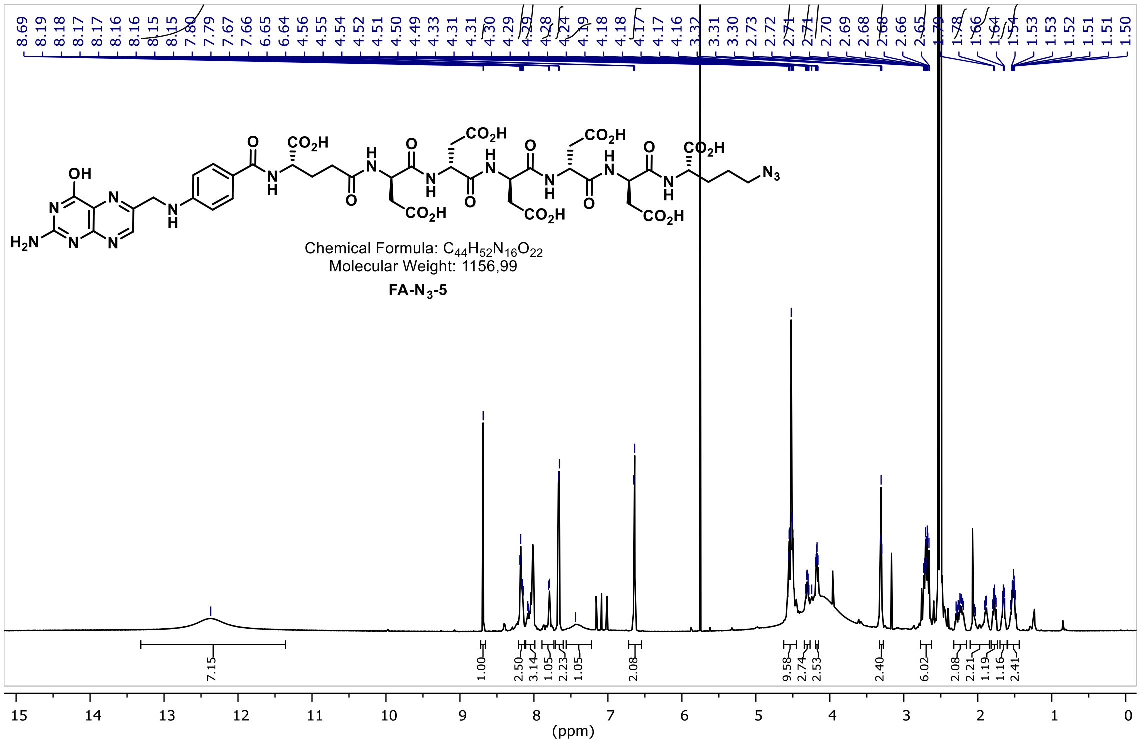

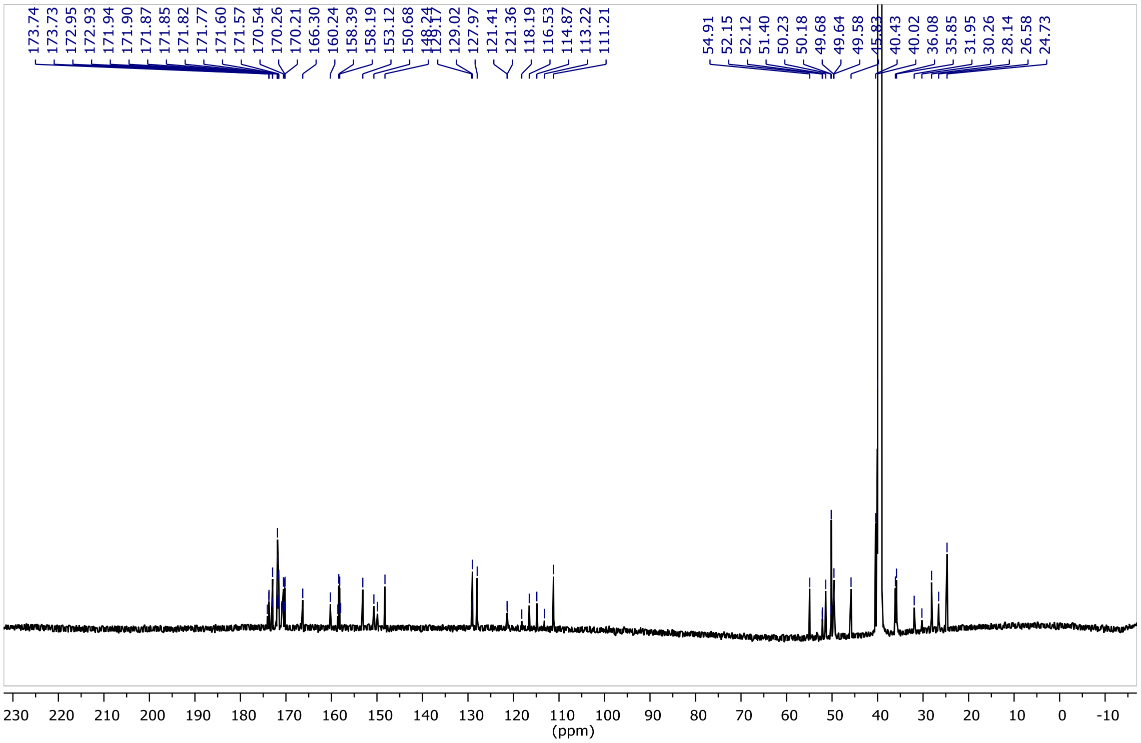

FA-N_3-6 - (3S,8S,13S)-1-(4-(((2-amino-4-hydroxypteridin-6-yl)methyl)amino)phenyl)-19-(4-azidophenyl)-1,6,11,19-tetraoxo-2,7,12,18-tetraazanonadecane-3,8,13-tricarboxylic acid

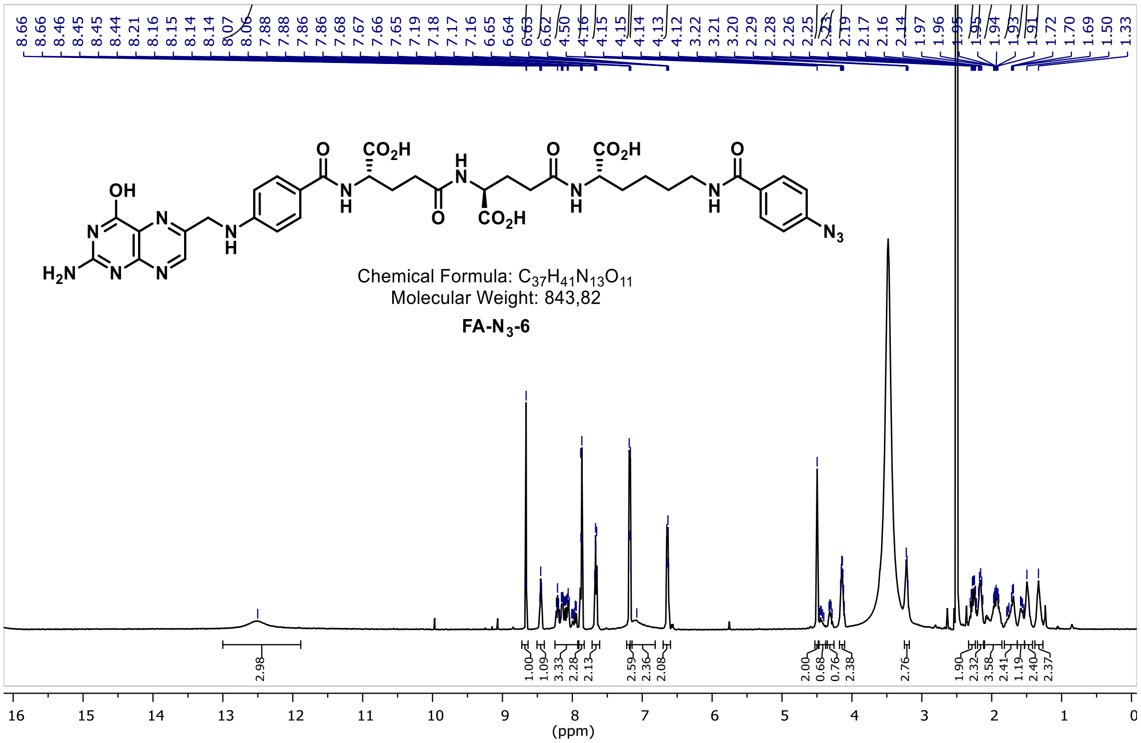

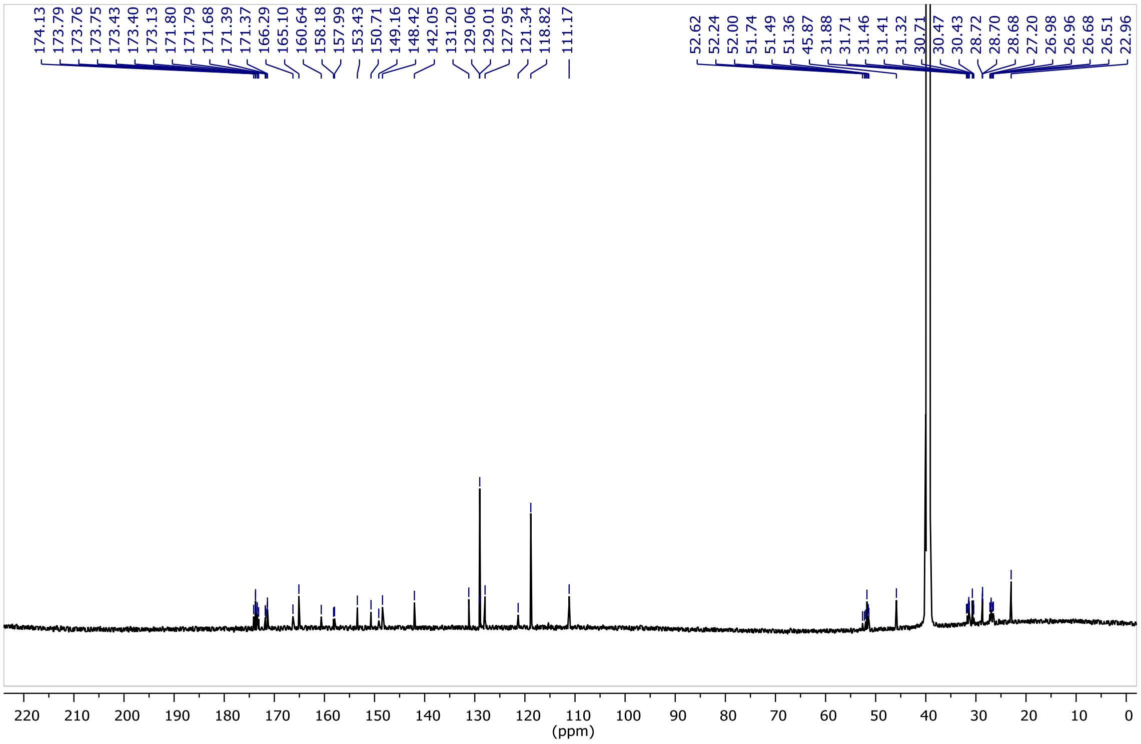

FA-N$_3$-7 - N^2-(4-(((2-amino-4-hydroxypteridin-6-yl)methyl)amino)benzoyl)-N^5-((S)-4-(((S)-4-azido-1-carboxybutyl)amino)-1-carboxy-4-oxobutyl)-L-glutamine

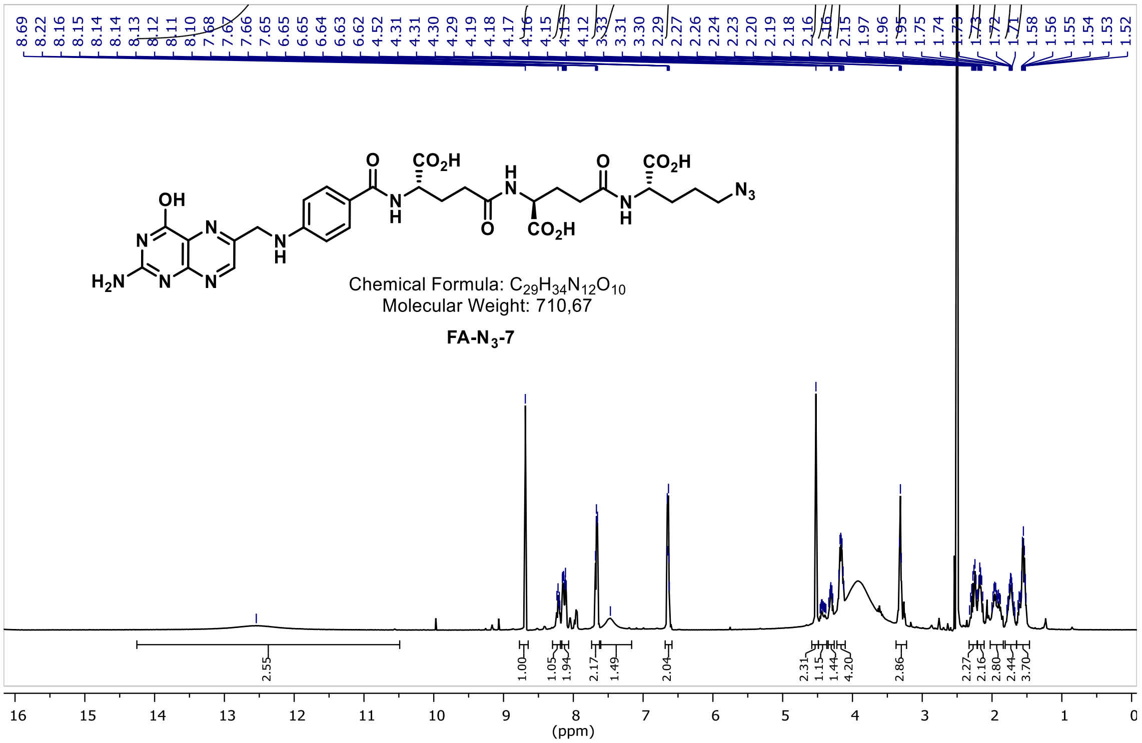

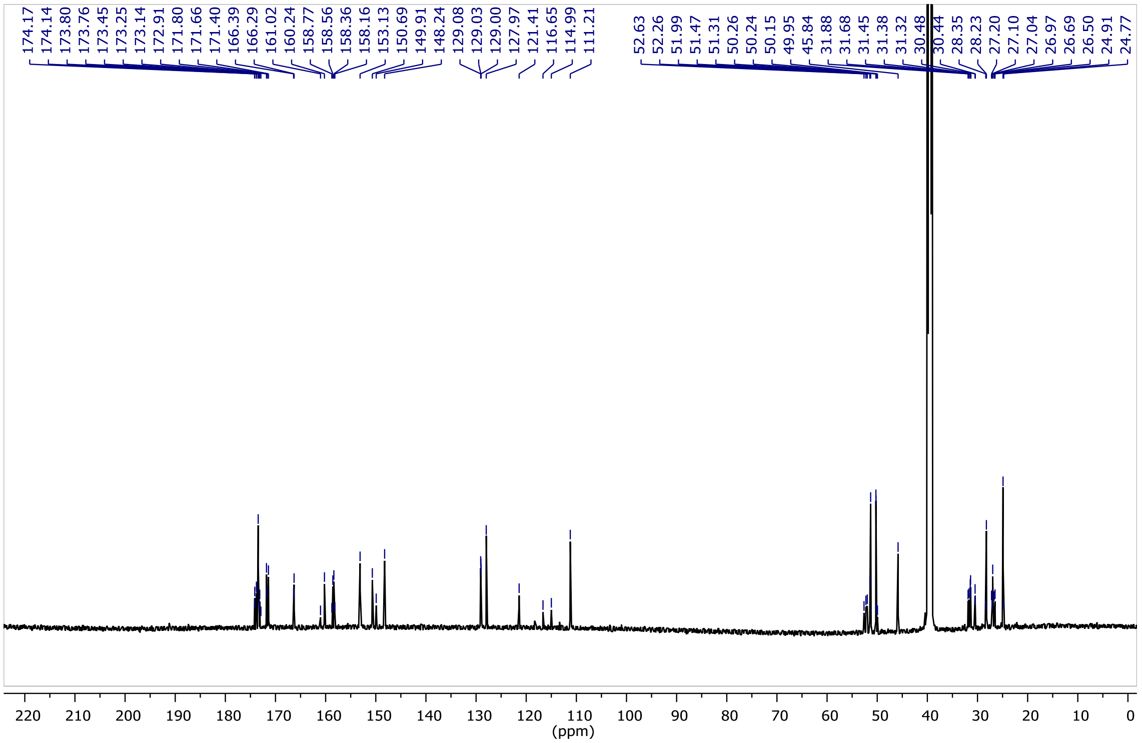

FA-N_3-8 - N^2-((S)-4-(4-(((2-amino-4-hydroxypteridin-6-yl)methyl)amino)benzamido)-4-carboxybutanoyl)-N^6-(4-azidobenzoyl)-L-lysyl-D-lysine

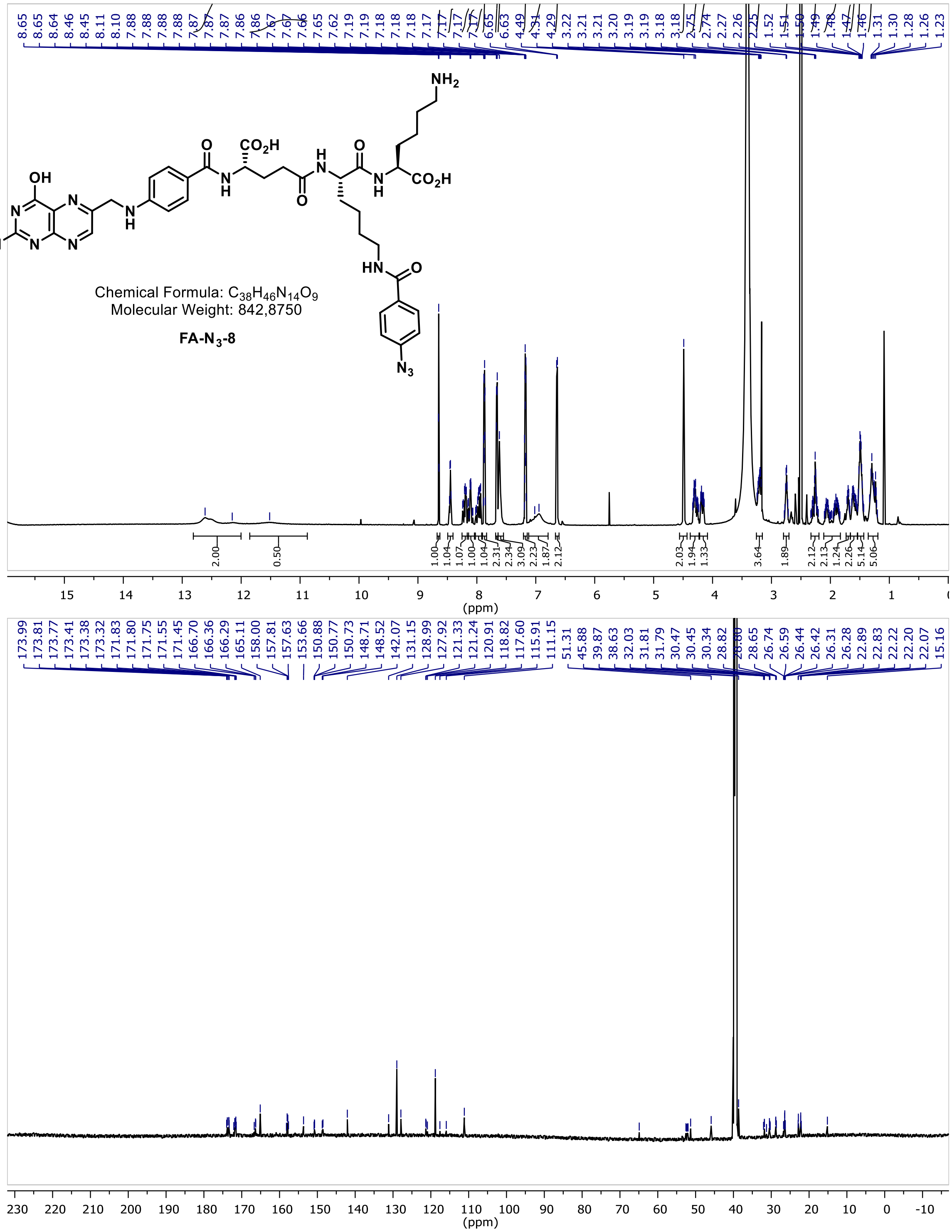

FA-N_3-9 - ((S)-2-((S)-4-(4-(((2-amino-4-hydroxypteridin-6-yl)methyl)amino)benzamido)-4-carboxybutanamido)-5-azidopentanoyl)-D-lysine

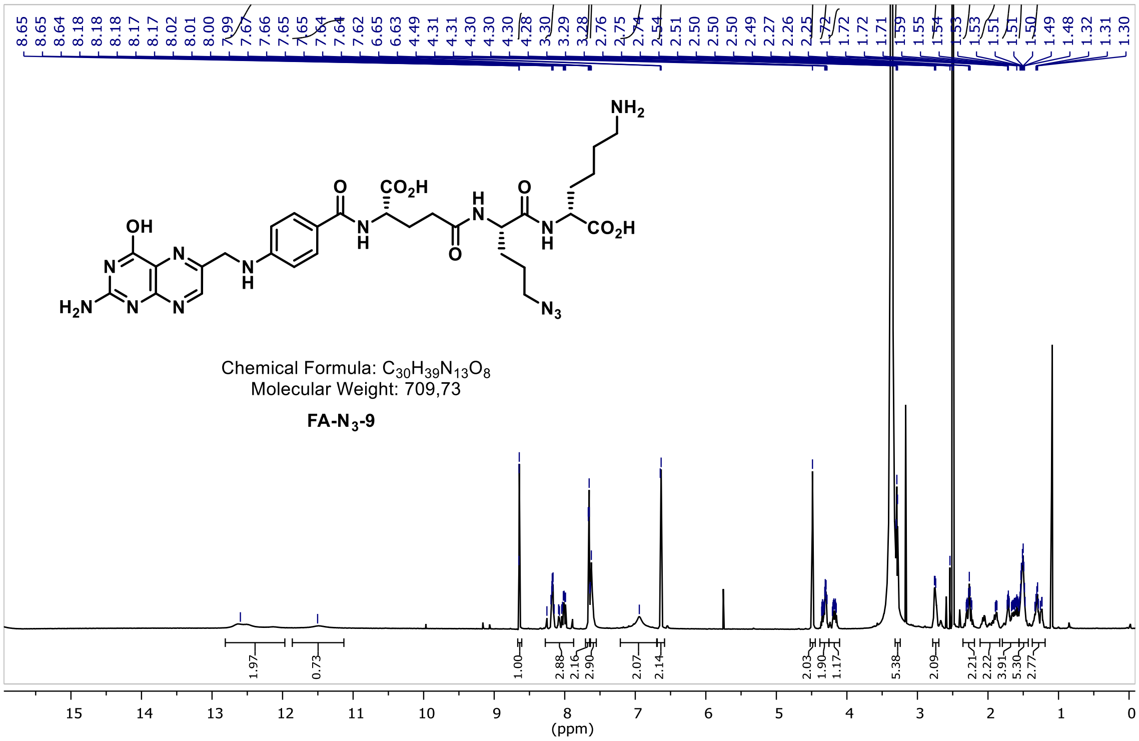

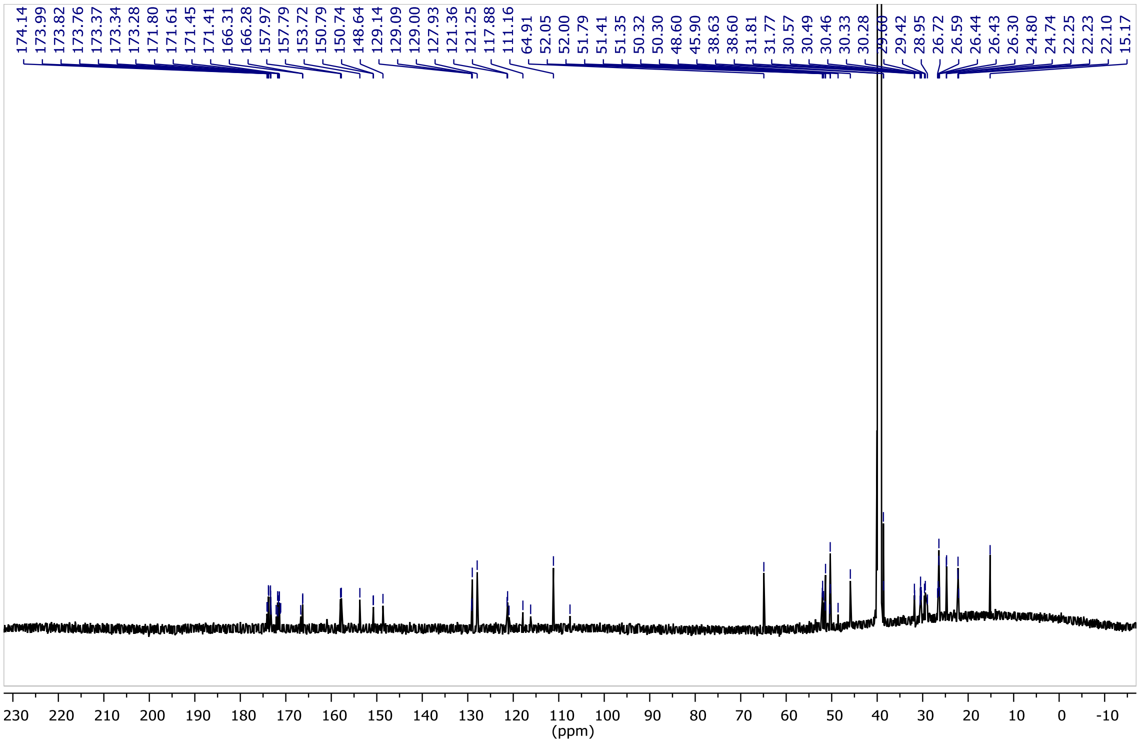

FA-N$_3$-10 - ((S)-2-((R)-2-((R)-2-((R)-2-((S)-4-(4-(((2-amino-4-hydroxypteridin-6-yl)methyl)amino)benzamido)-4-carboxybutanamido)-3-carboxypropanamido)-3-carboxypropanamido)-3-carboxypropanamido)-5-azidopentanoyl)-D-aspartyl-D-aspartyl-D-lysine

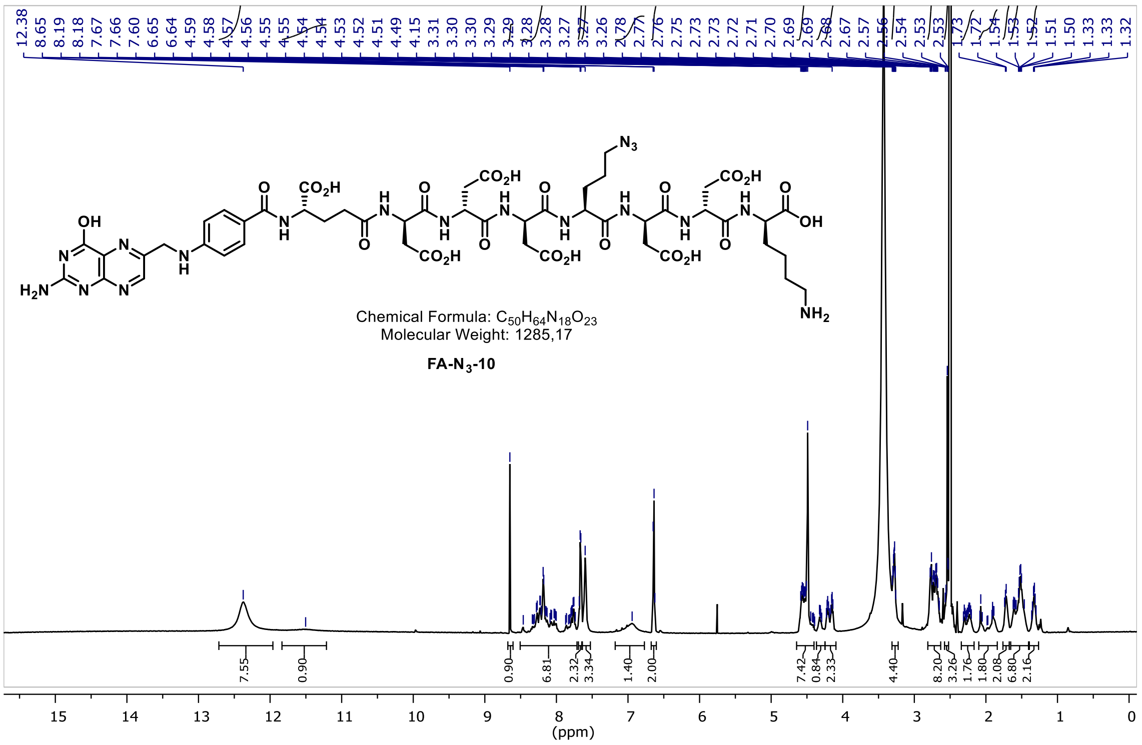

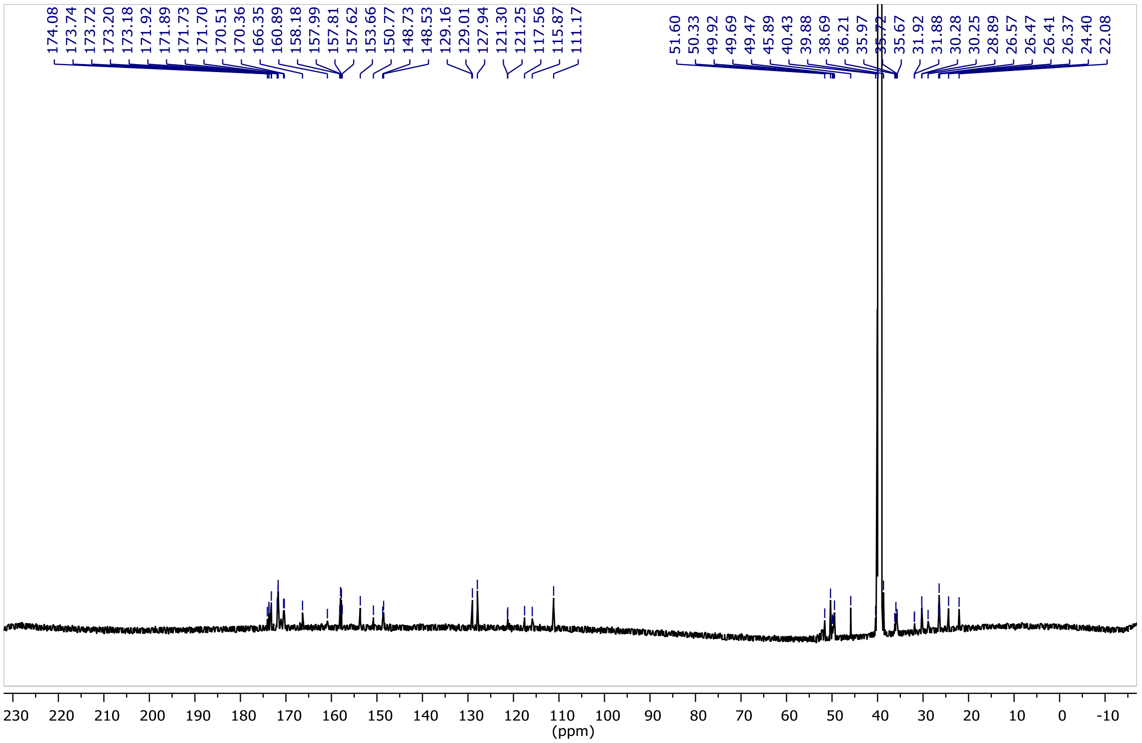

FA-N$_3$-11 - N^2-(4-(((2-amino-4-hydroxypteridin-6-yl)methyl)amino)benzoyl)-N^5-((R)-1-(((R)-1-(((S)-5-azido-1-(((R)-1-(((R)-1-(((S)-5-azido-1-(((R)-1-(((R)-1-(((S)-4-azido-1-carboxybutyl)amino)-3-carboxy-1-oxopropan-2-yl)amino)-3-carboxy-1-oxopropan-2-yl)amino)-1-oxopentan-2-yl)amino)-3-carboxy-1-oxopropan-2-yl)amino)-3-carboxy-1-oxopropan-2-yl)amino)-1-oxopentan-2-yl)amino)-3-carboxy-1-oxopropan-2-yl)amino)-3-carboxy-1-oxopropan-2-yl)-L-glutamine

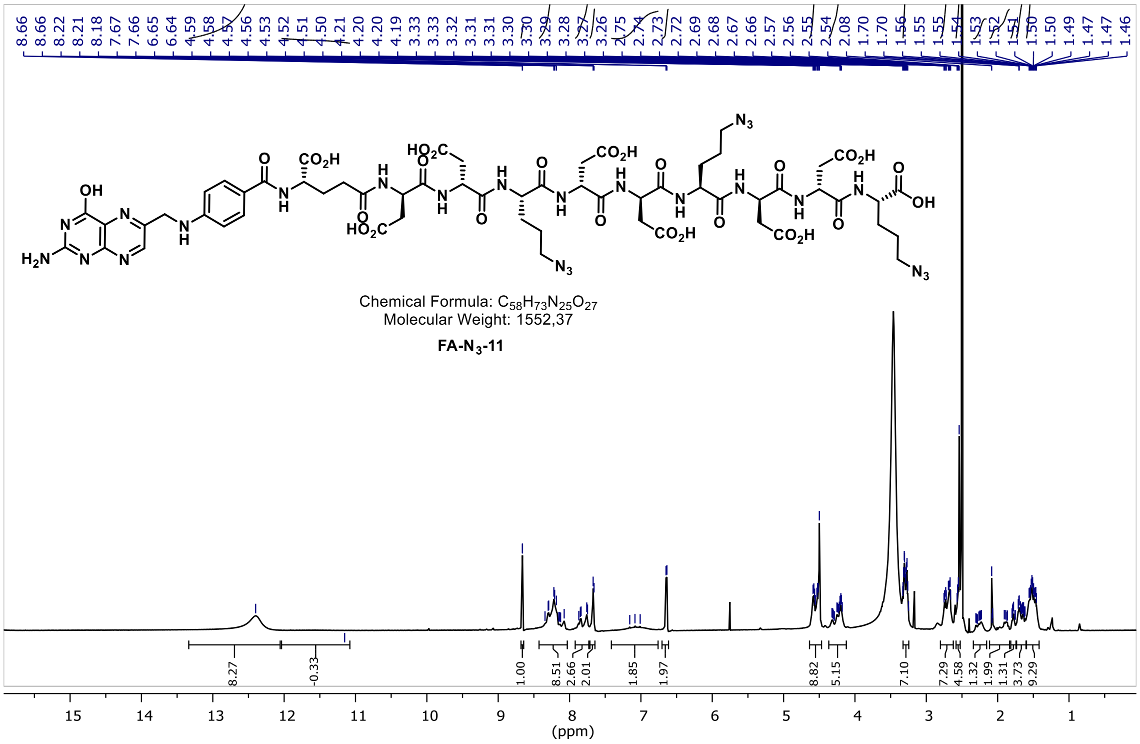

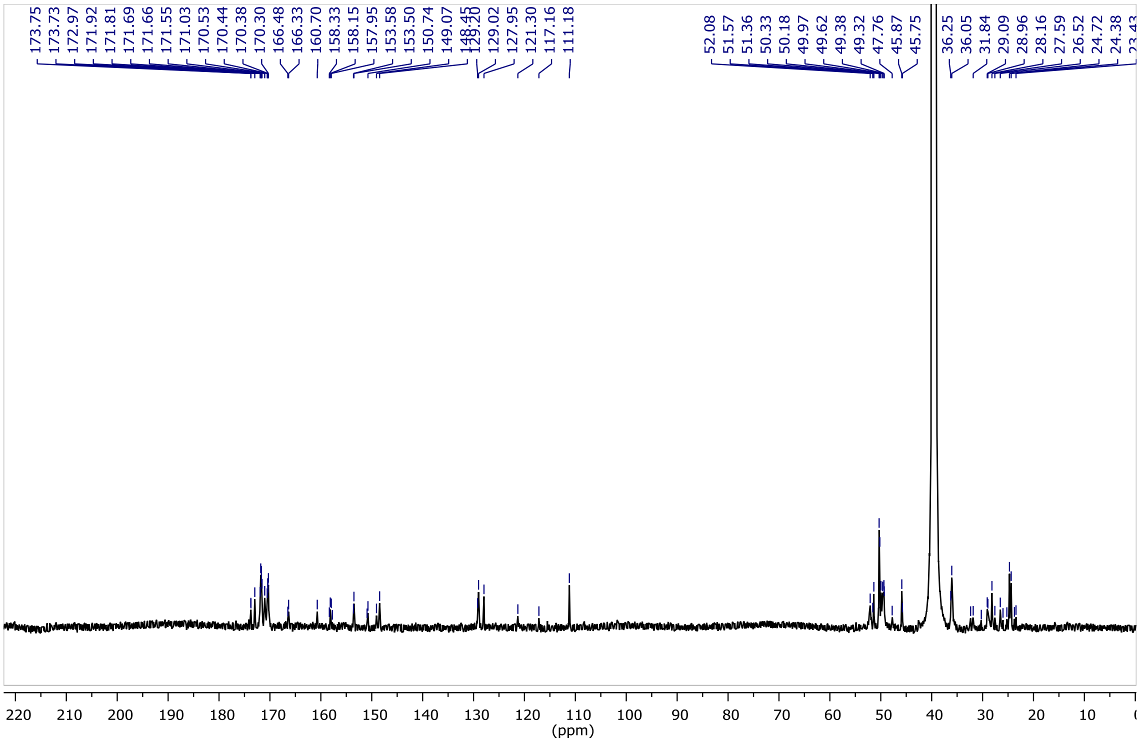

FA-SH-1 - N[2]-(4-(((2-amino-4-hydroxypteridin-6-yl)methyl)amino)benzoyl)-N[5]-((R)-1-carboxy-2-mercaptoethyl)-L-glutamine

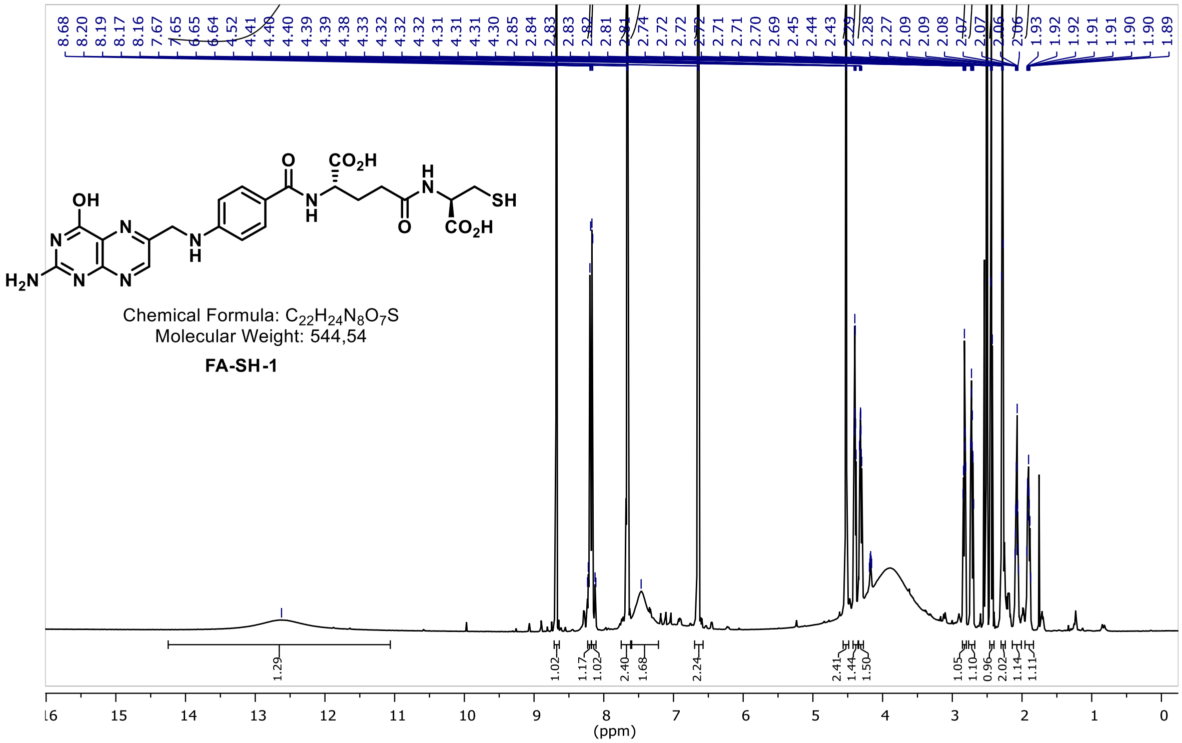

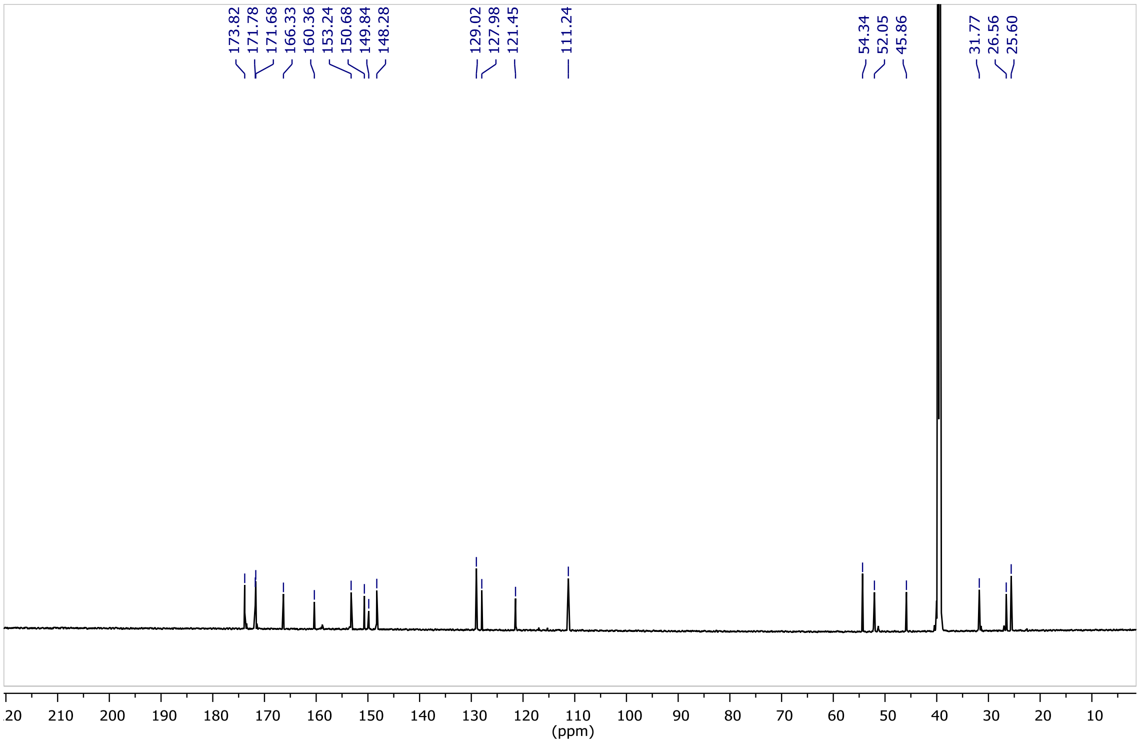

LHRH

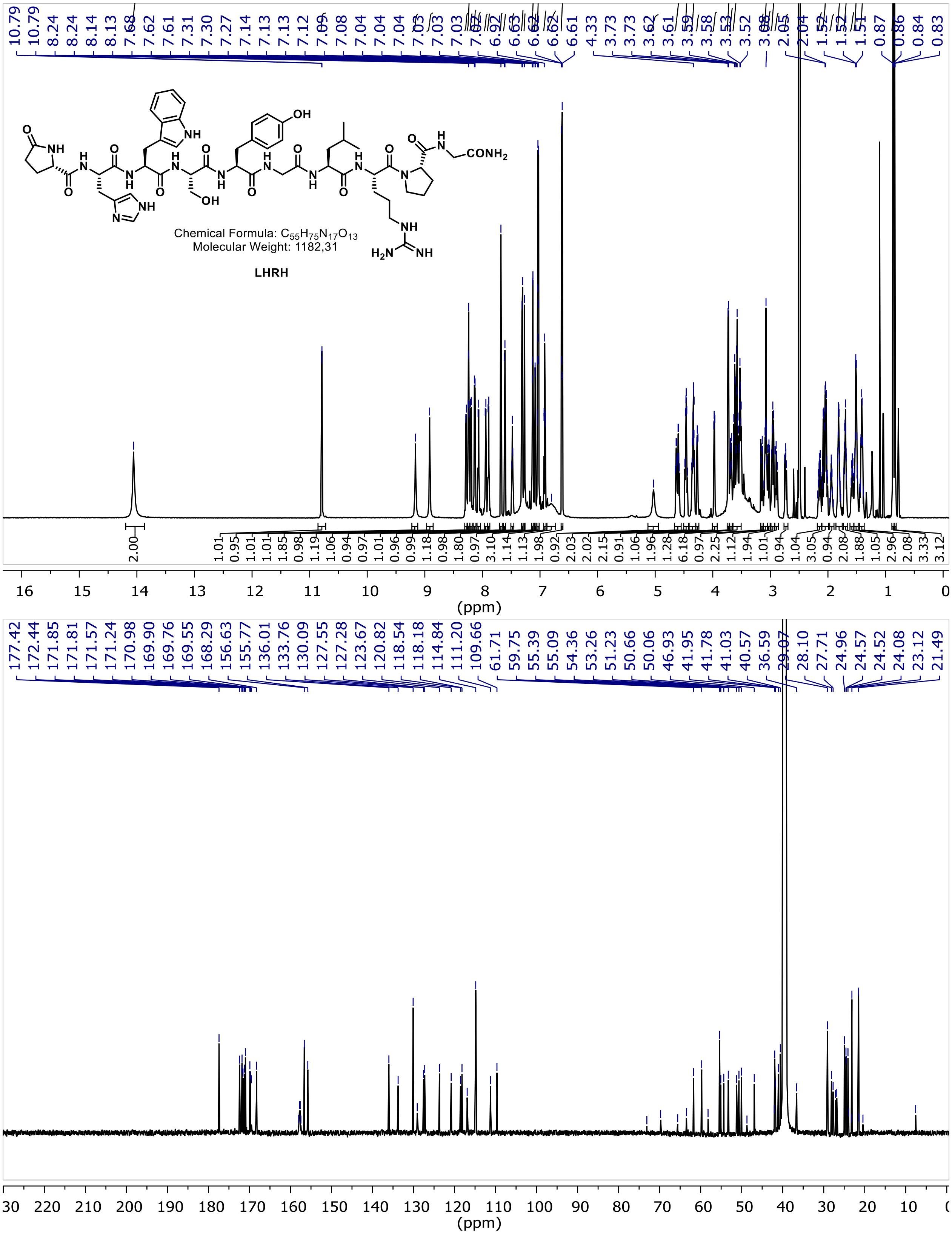
Chemical Formula: $C_{55}H_{75}N_{17}O_{13}$
Molecular Weight: 1182,31
LHRH

L-N$_3$-Orn-LHRH

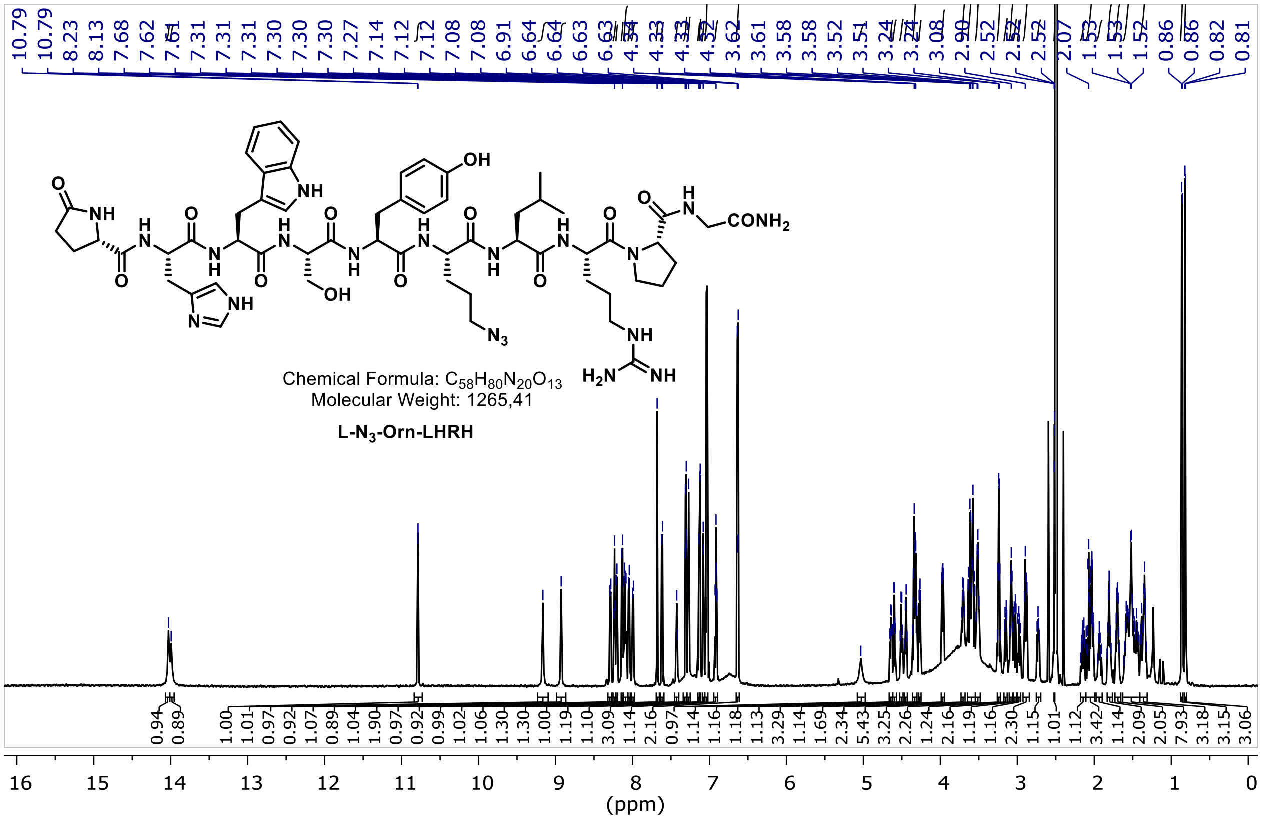
Chemical Formula: C58H80N20O13
Molecular Weight: 1265,41
L-N3-Orn-LHRH
(ppm)

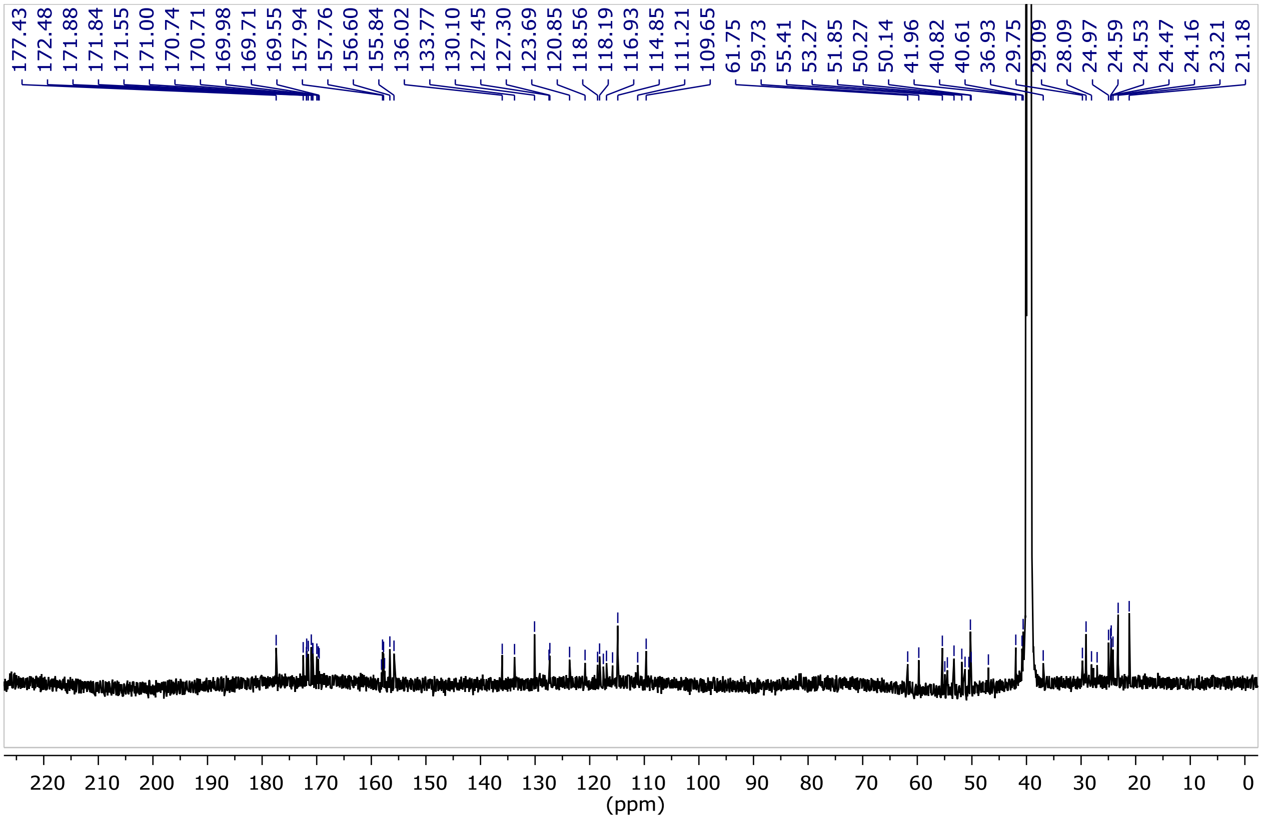
(ppm)

D-N$_3$-Orn-LHRH

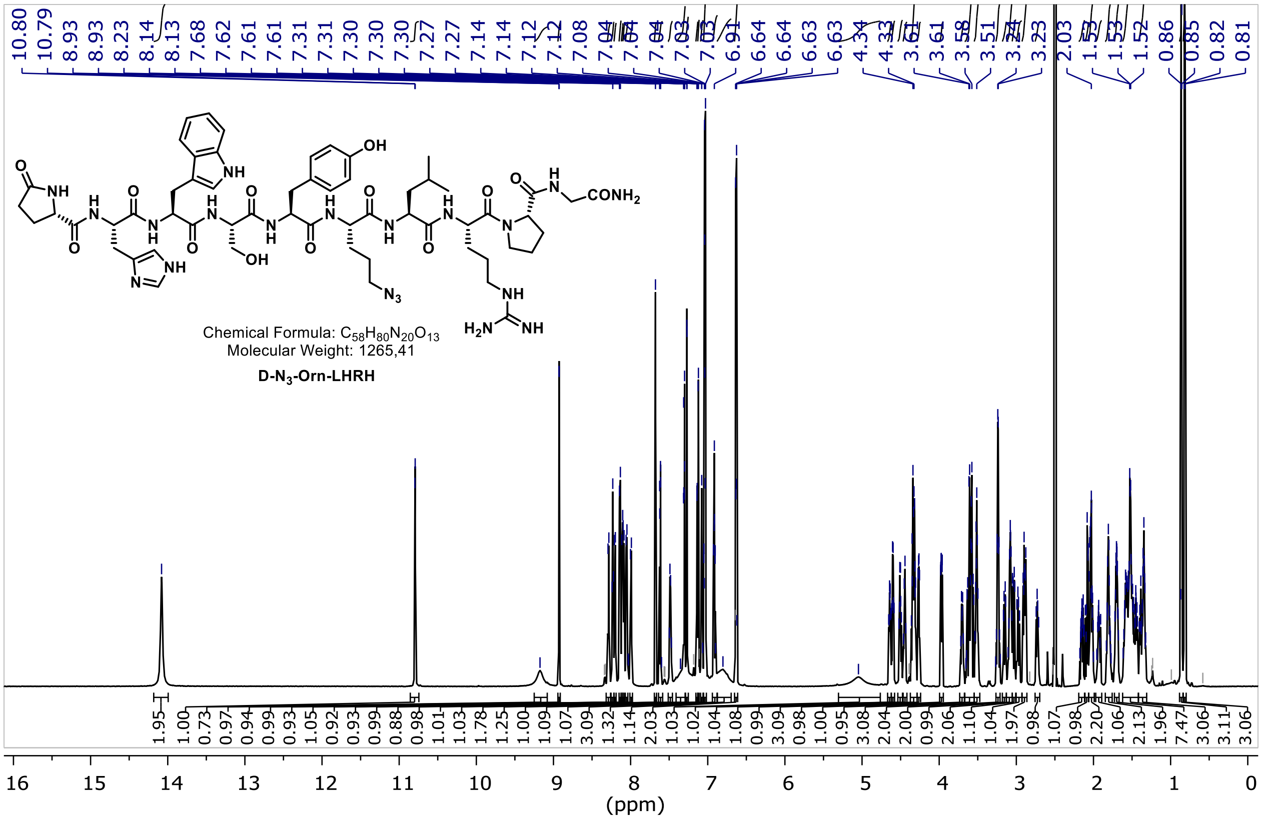
Chemical Formula: C58H80N20O13
Molecular Weight: 1265,41
D-N3-Orn-LHRH
(ppm)

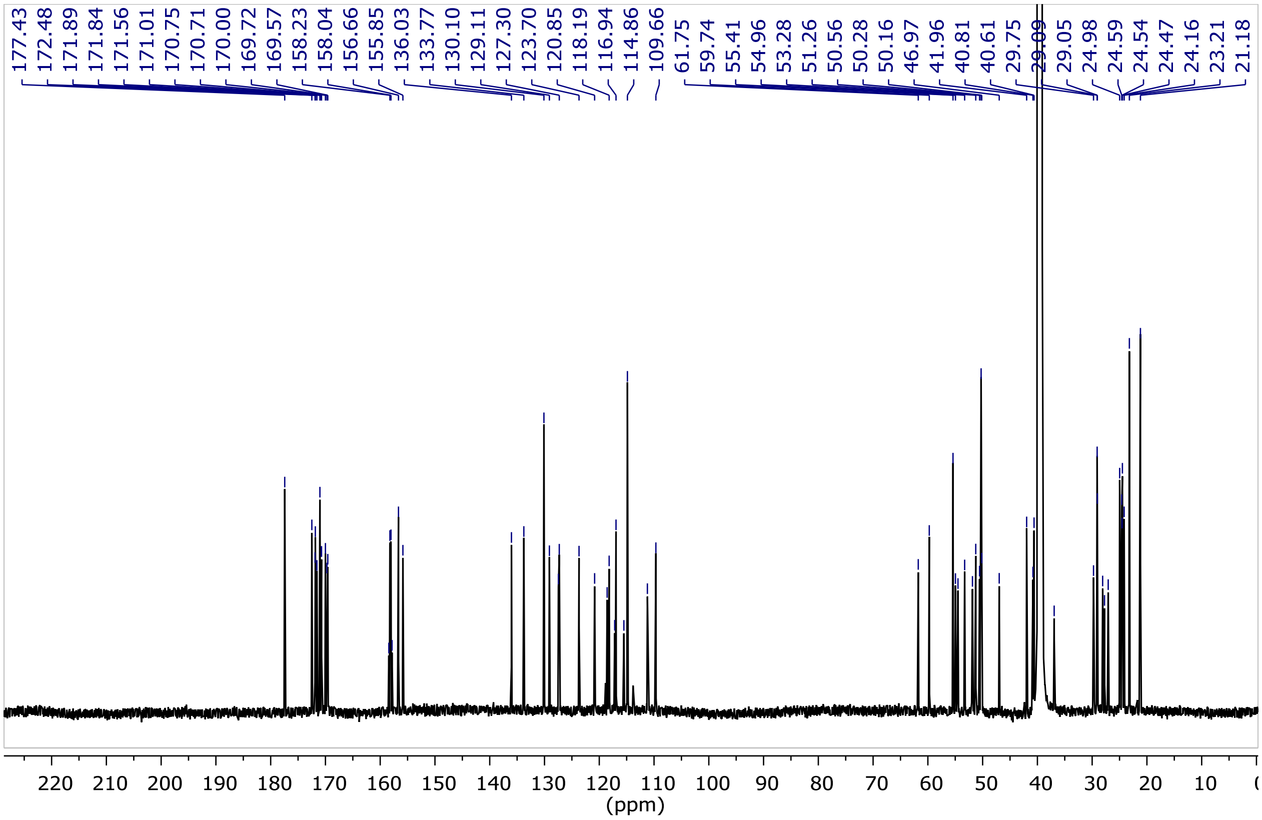
(ppm)

D-N$_3$-Orn-Gose

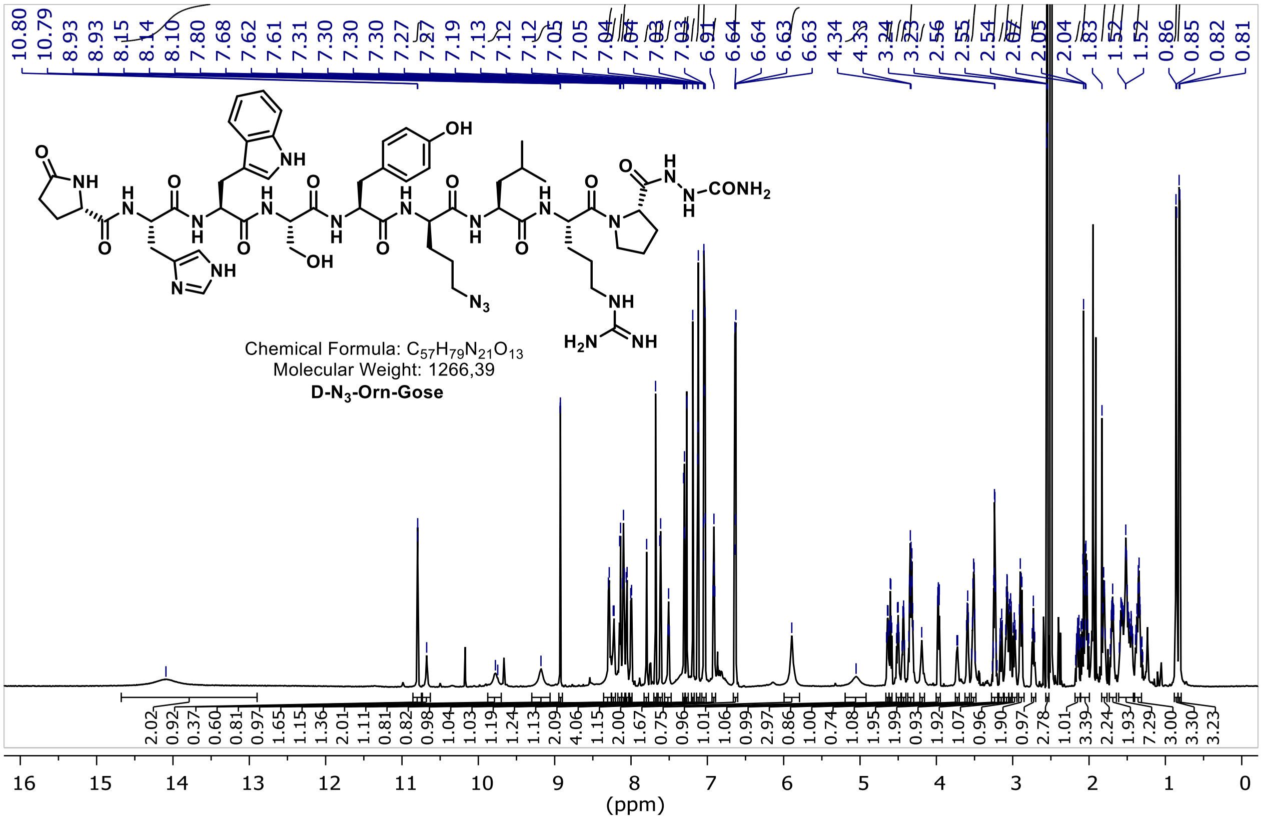
Chemical Formula: C57H79N21O13
Molecular Weight: 1266,39
D-N3-Orn-Gose
(ppm)

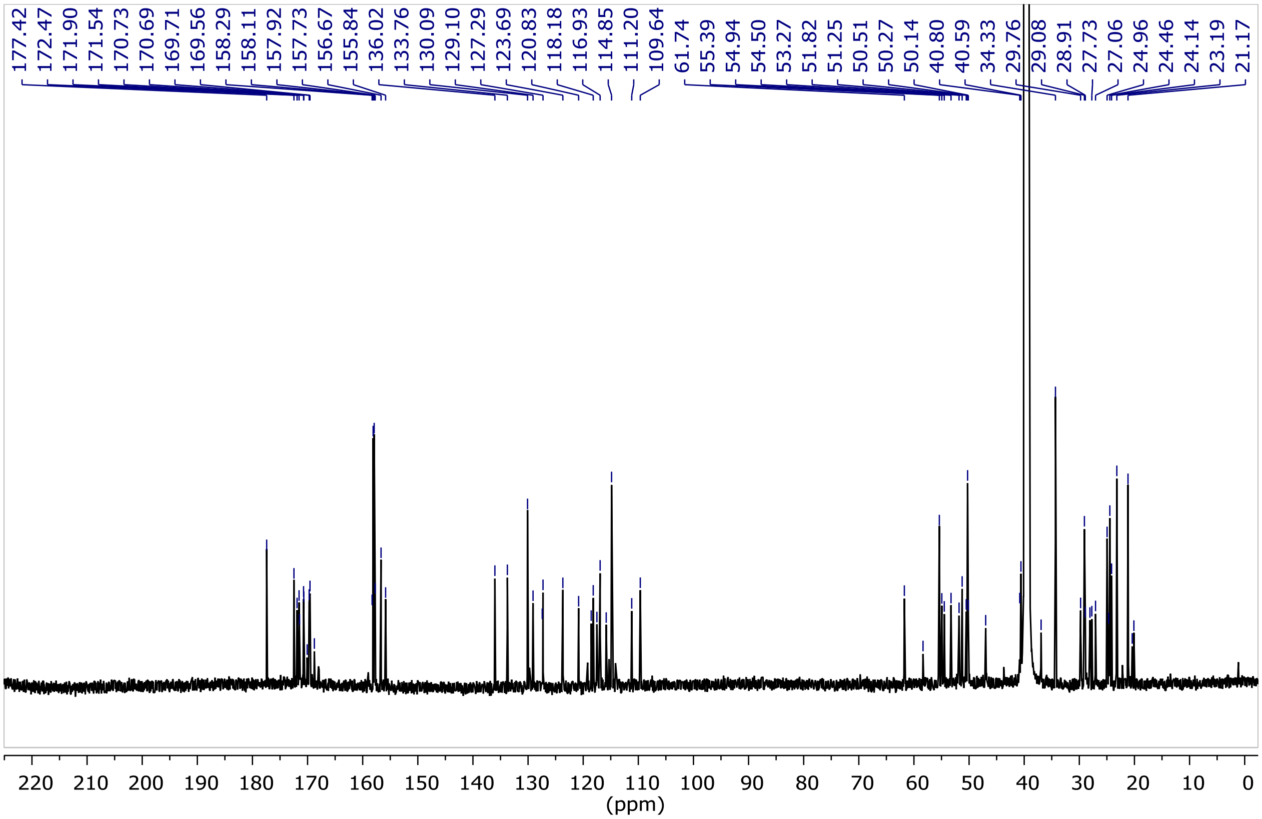
(ppm)

BCN-FTIC (43) - ((1R,8S,9s)-bicyclo[6.1.0]non-4-yn-9-yl)methyl (2-(3-(3',6'-dihydroxy-3-oxo-3H-spiro[isobenzofuran-1,9'-xanthen]-5/6-yl)thioureido)ethyl)carbamate

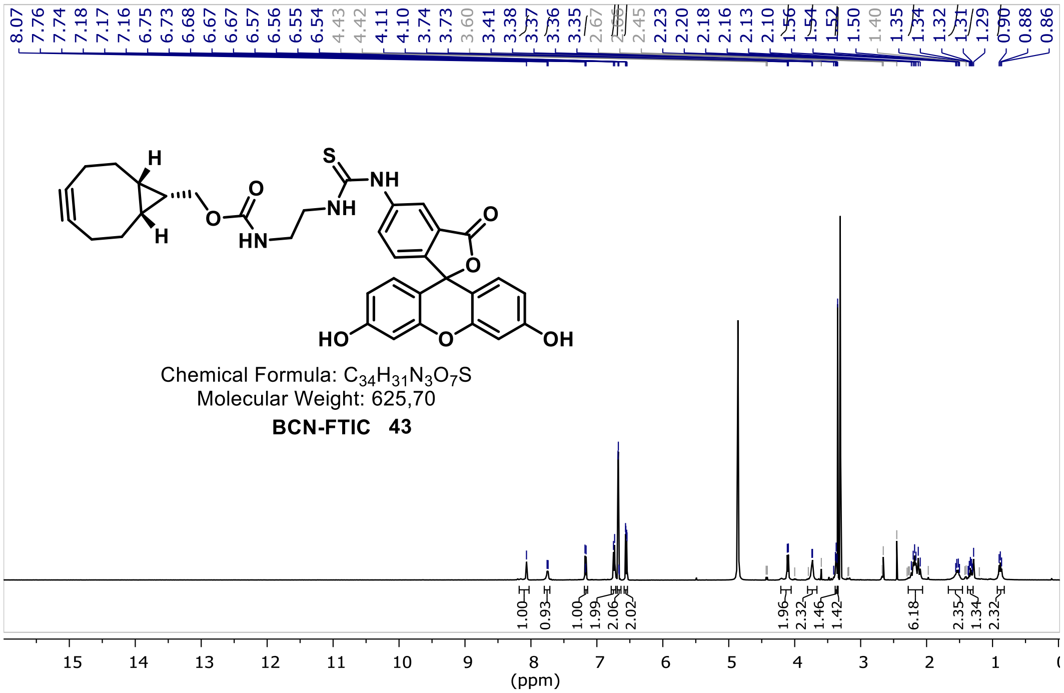

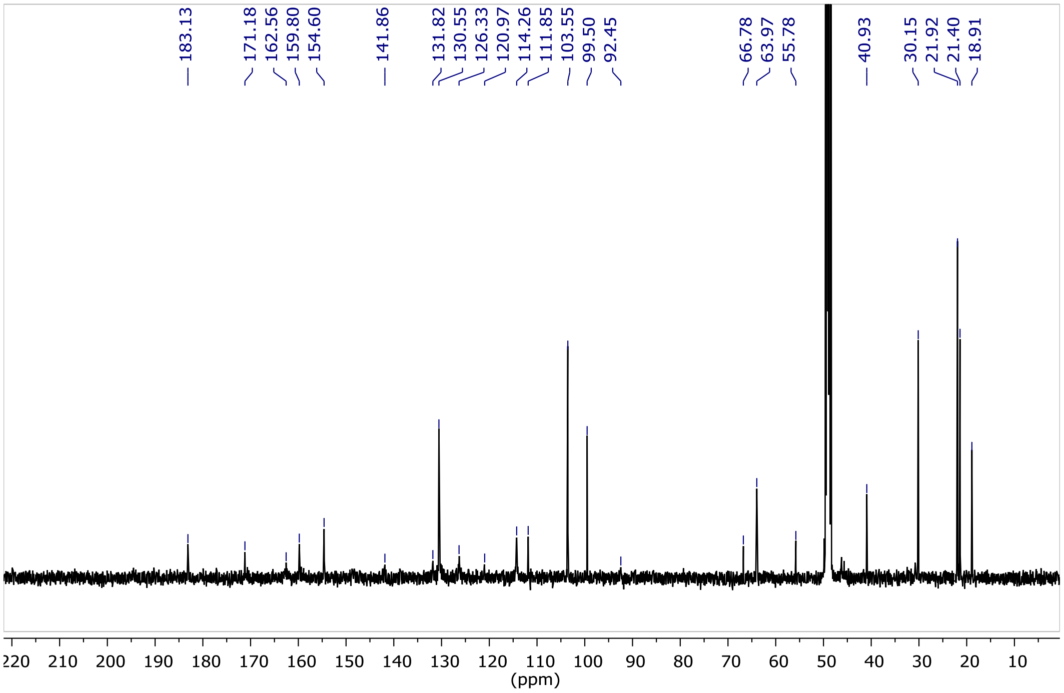

Homopropargyl-FTIC (**42**) - But-3-yn-1-yl (2-(3-(3',6'-dihydroxy-3-oxo-3H-spiro[isobenzofuran-1,9'-xanthen]-5/6-yl)thioureido)ethyl)carbamate

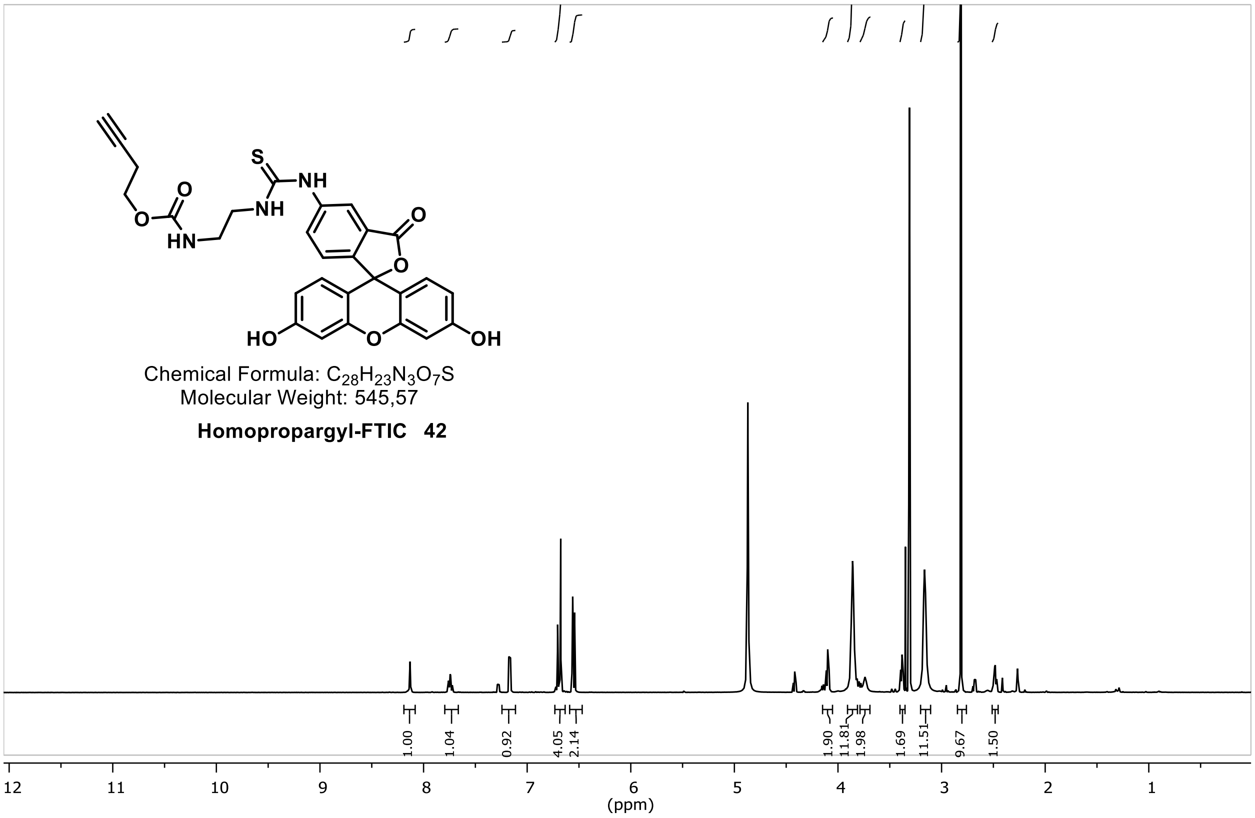

Biotin-PEG$_3$-16S-Aminoratjadone (36)

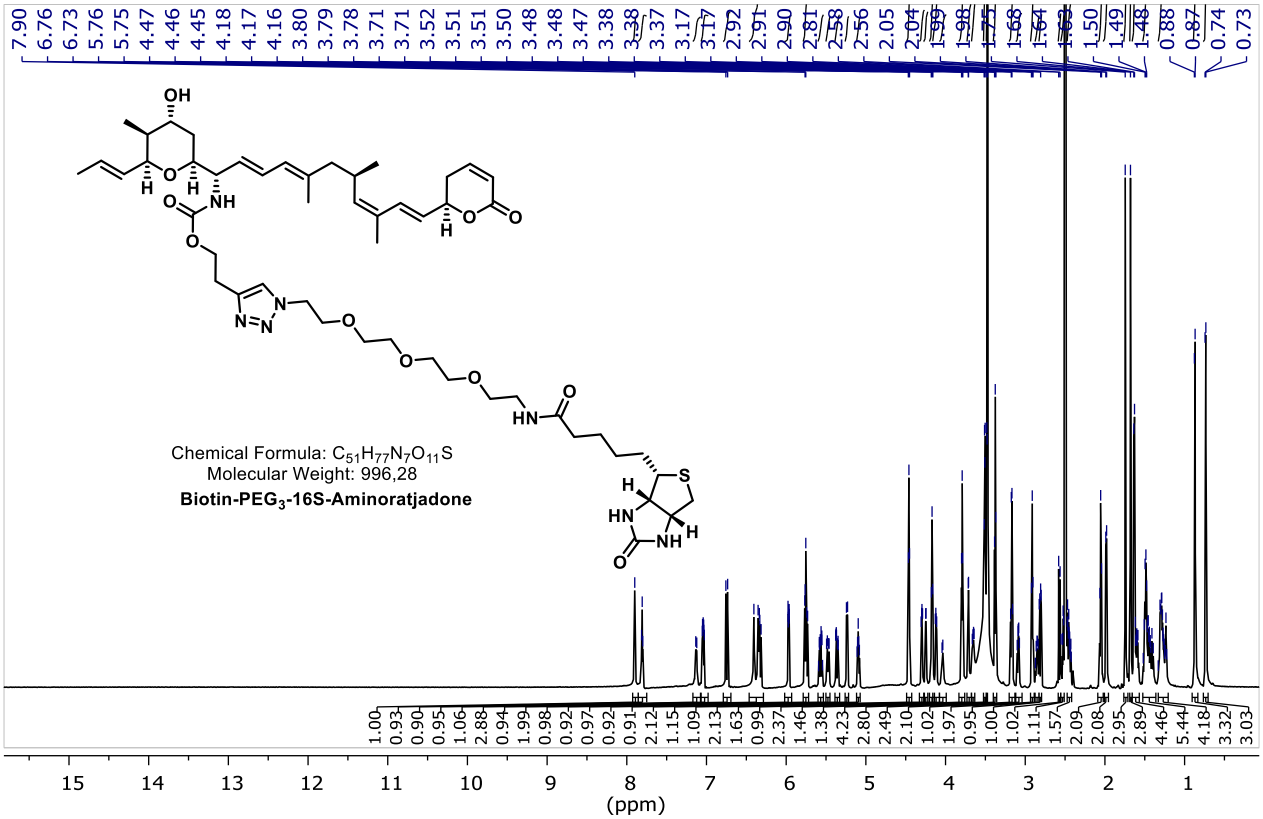

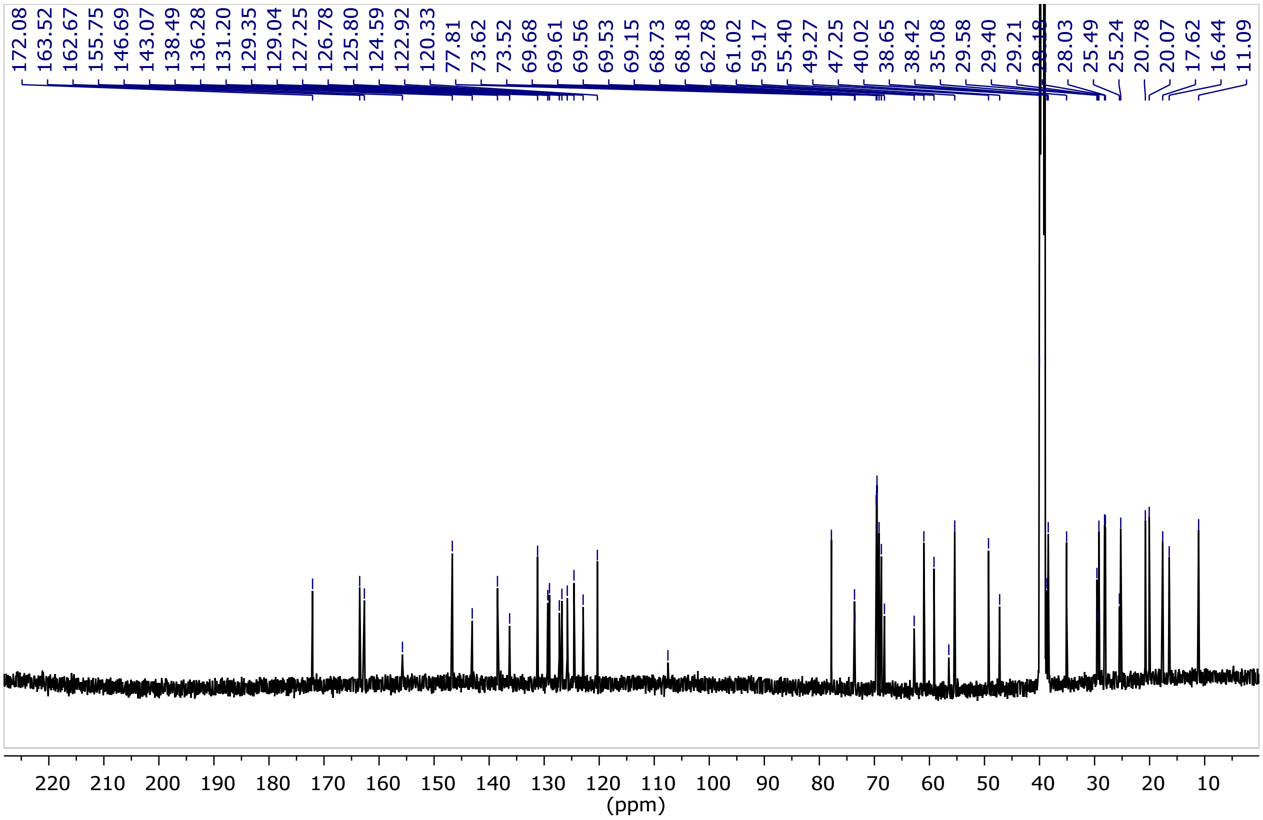

FA-1-Val-Cit-*p*ABA-16*R*-Aminoratjadone

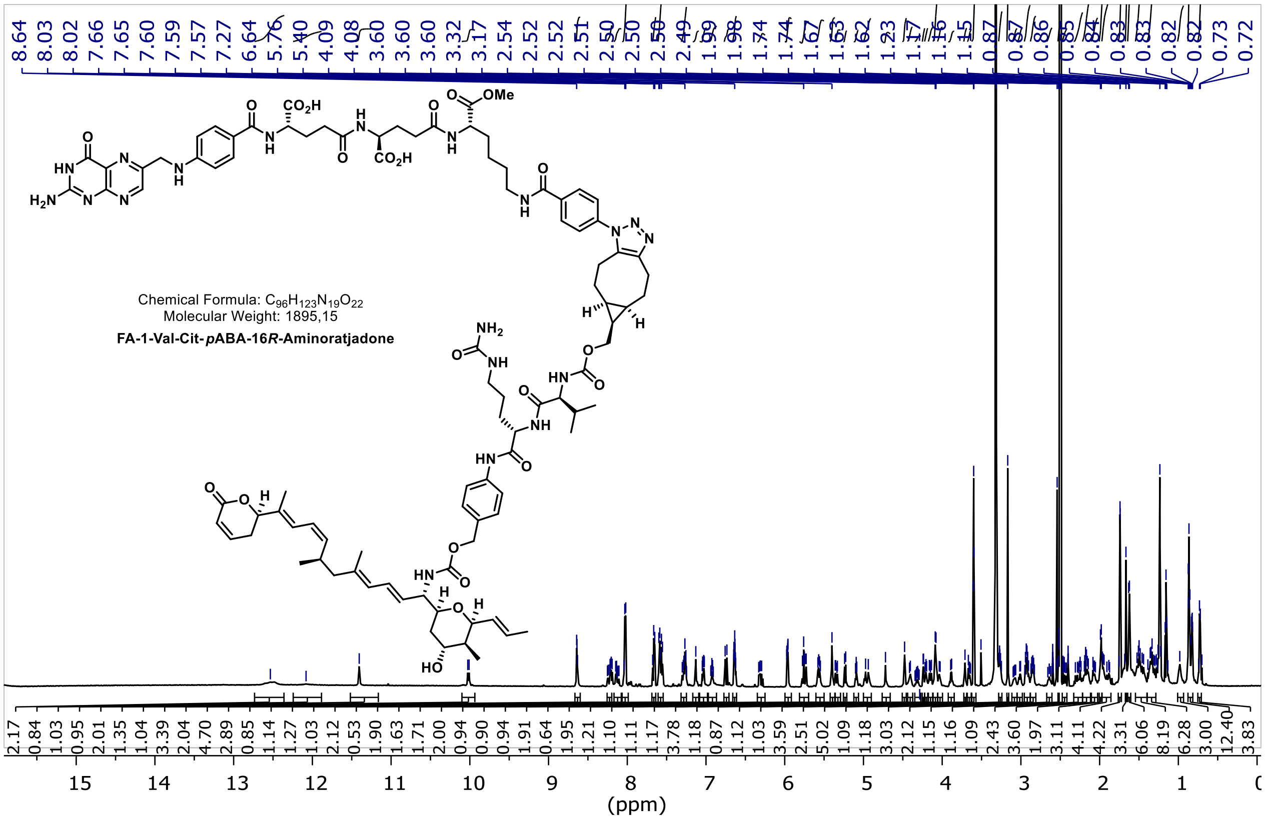

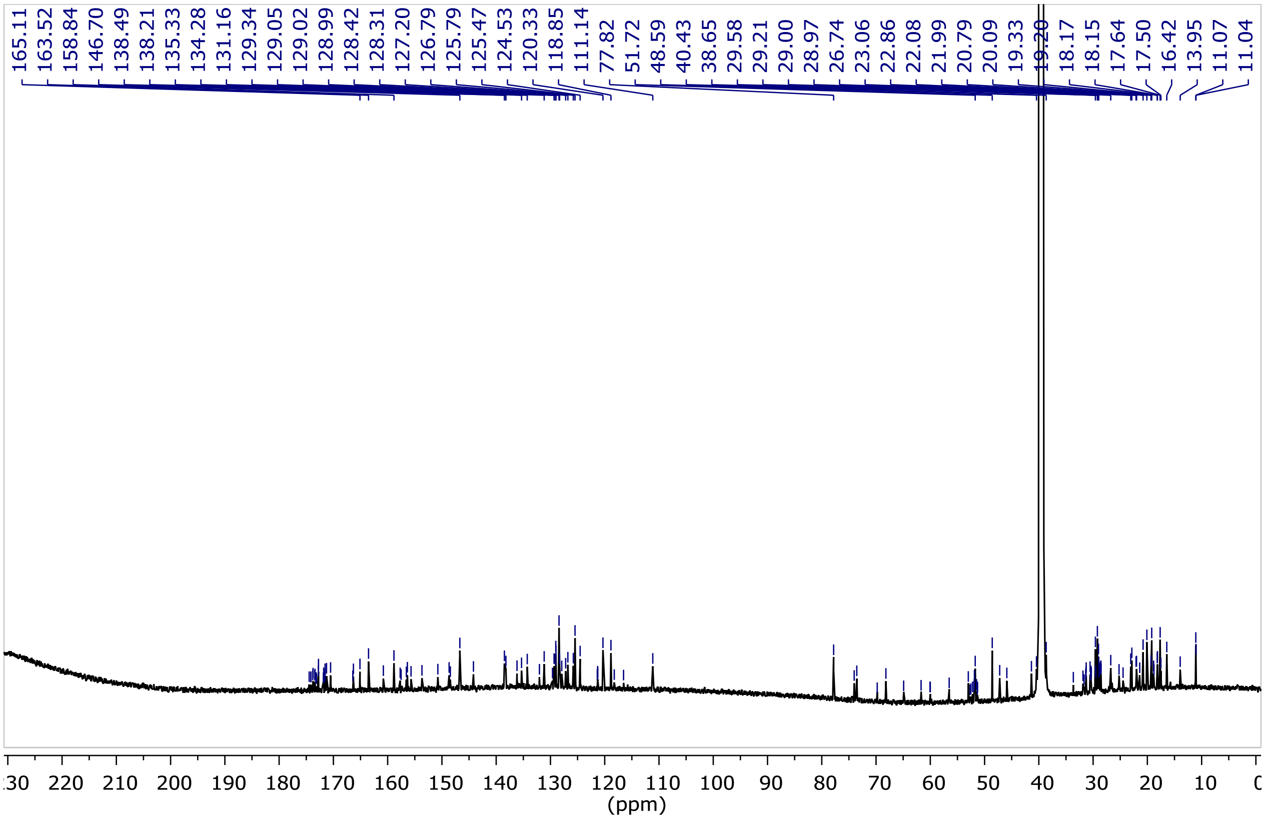

FA-3-Val-Cit-*p*ABA-16*R*-Aminoratjadone

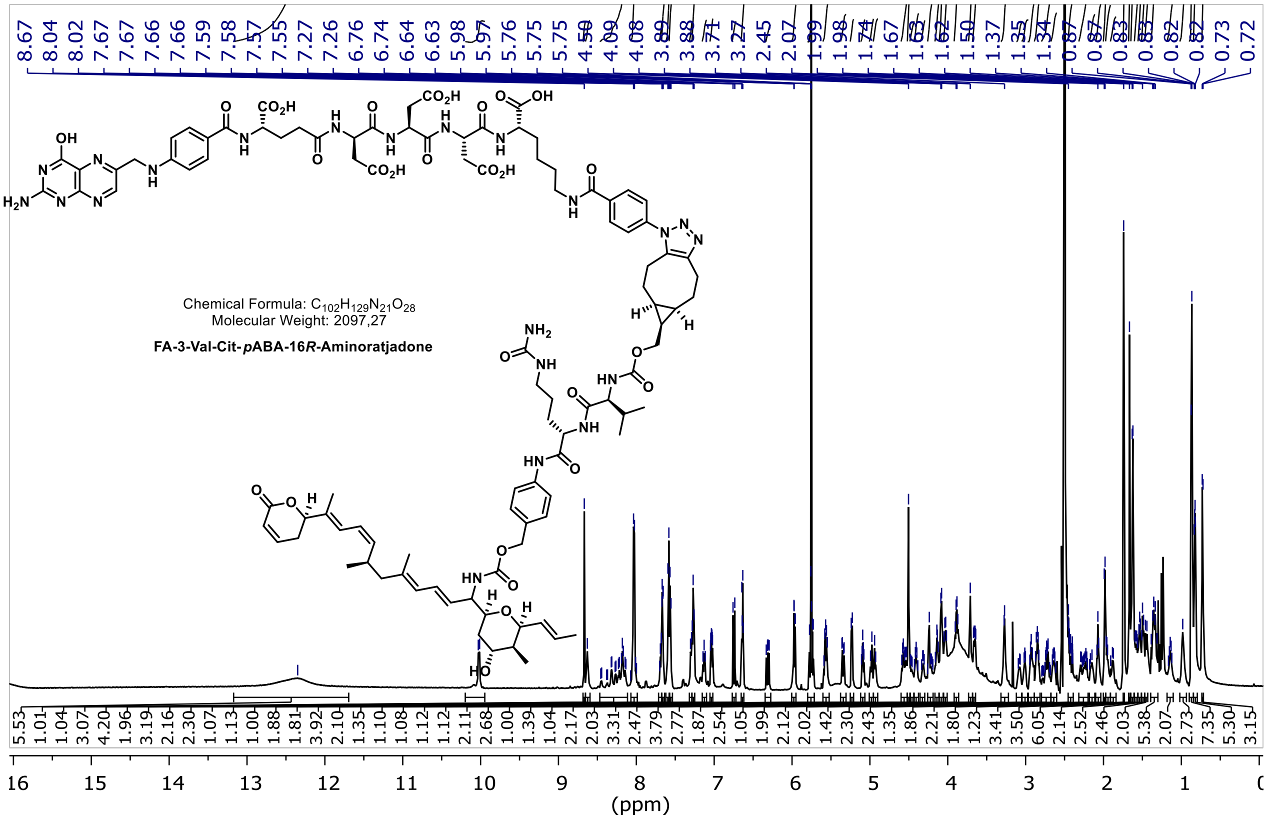

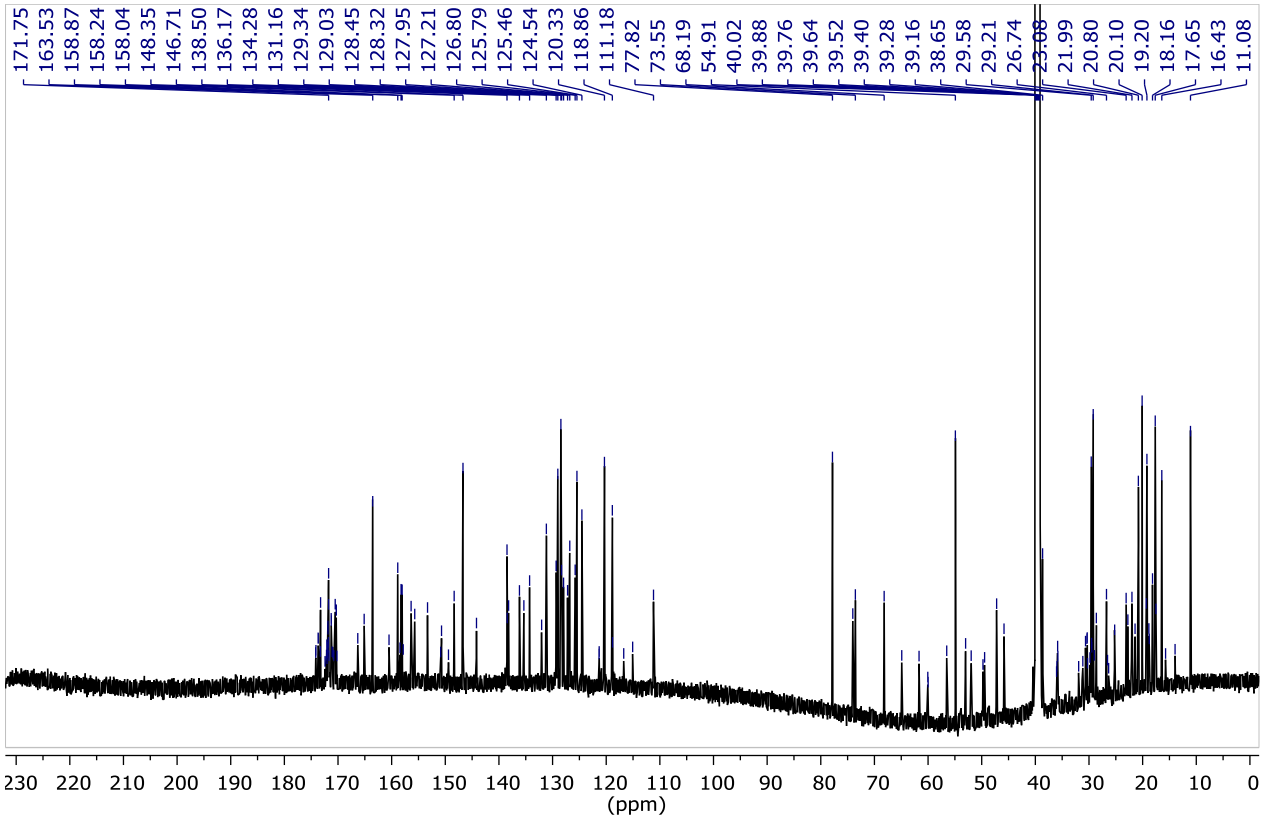

FA-4-(Val-Cit-*p*ABA-16*R*-Aminoratjadone)$_2$

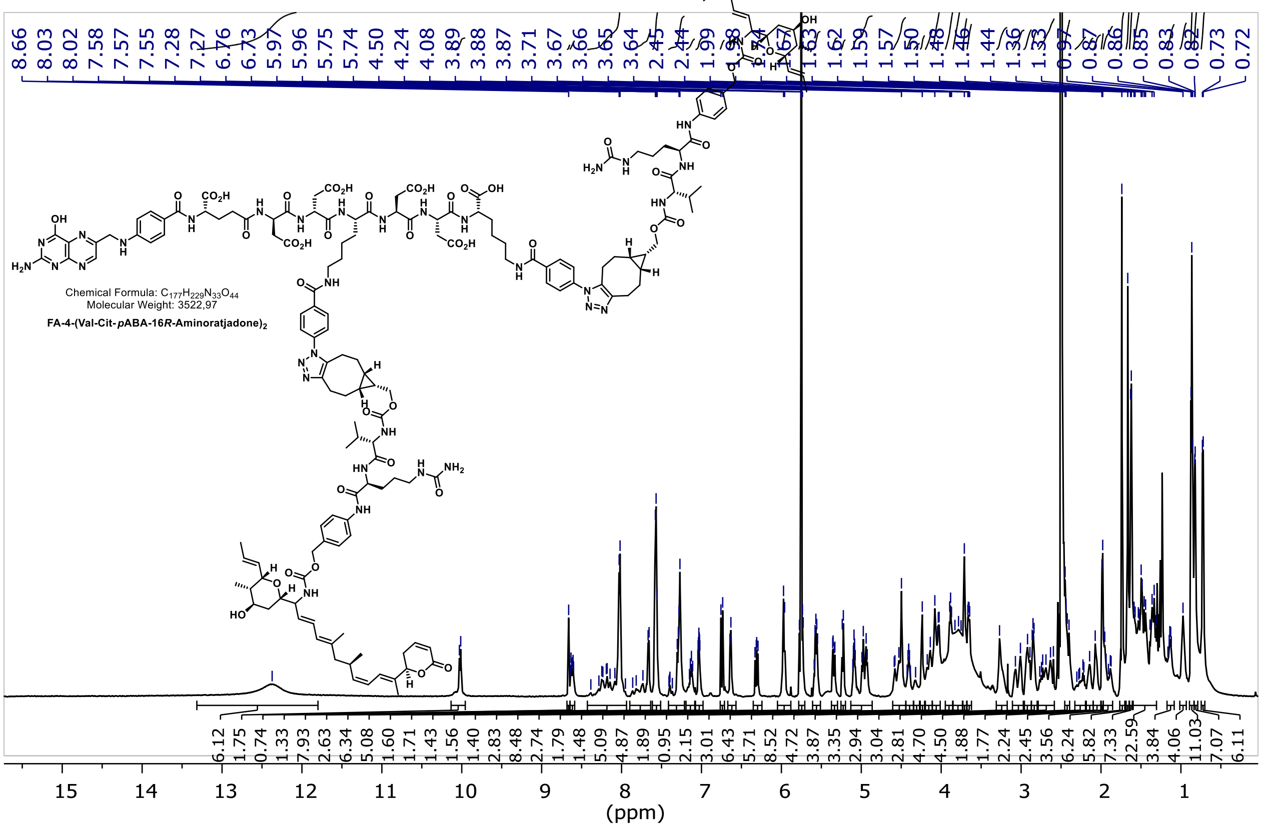

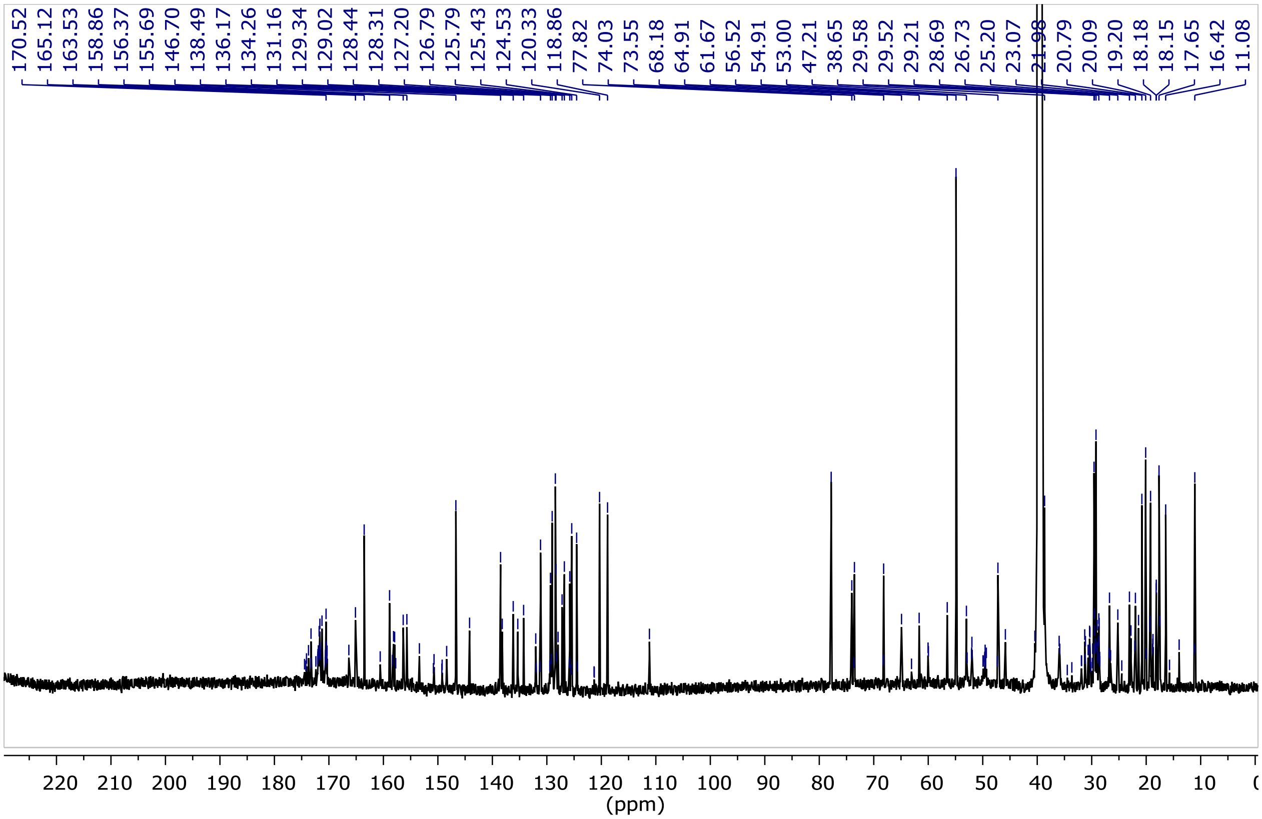

FA-5-Val-Cit-*p*ABA-16*R*-Aminoratjadone

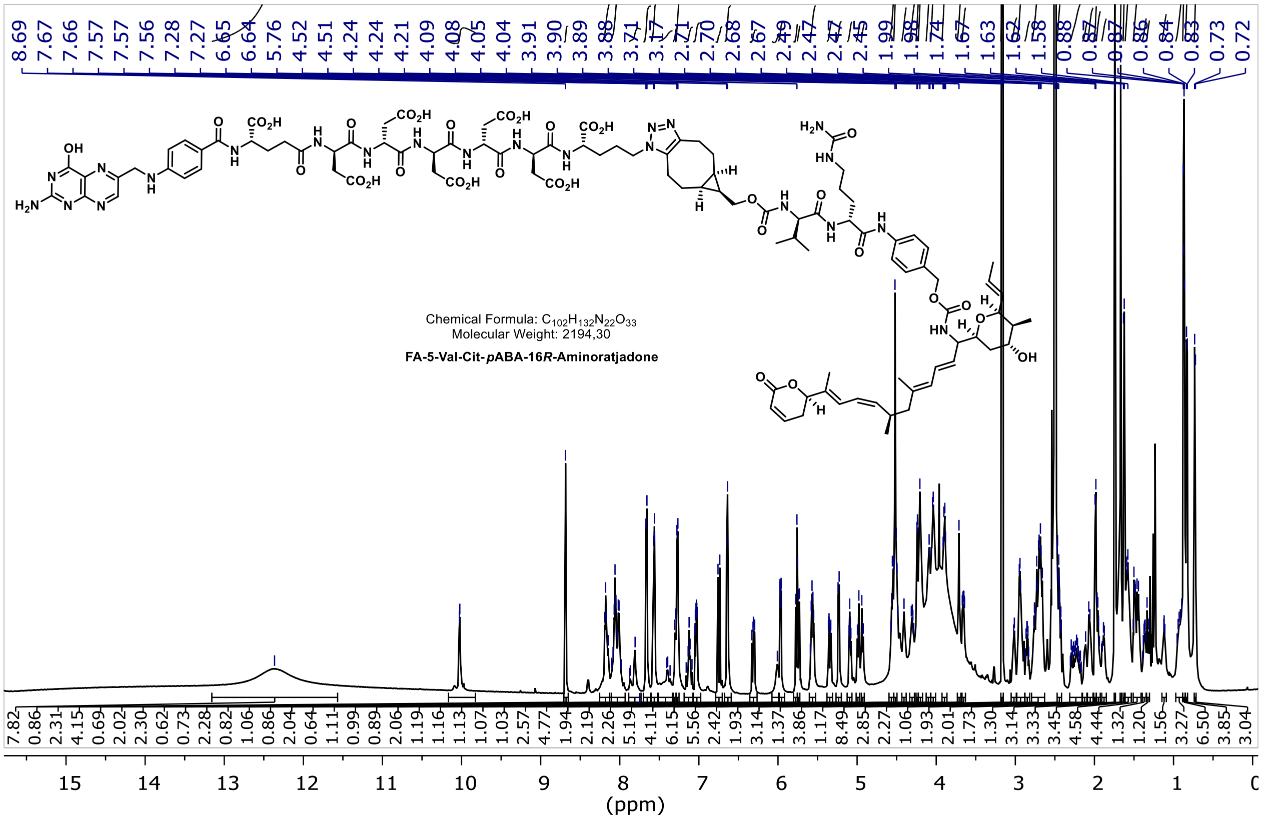

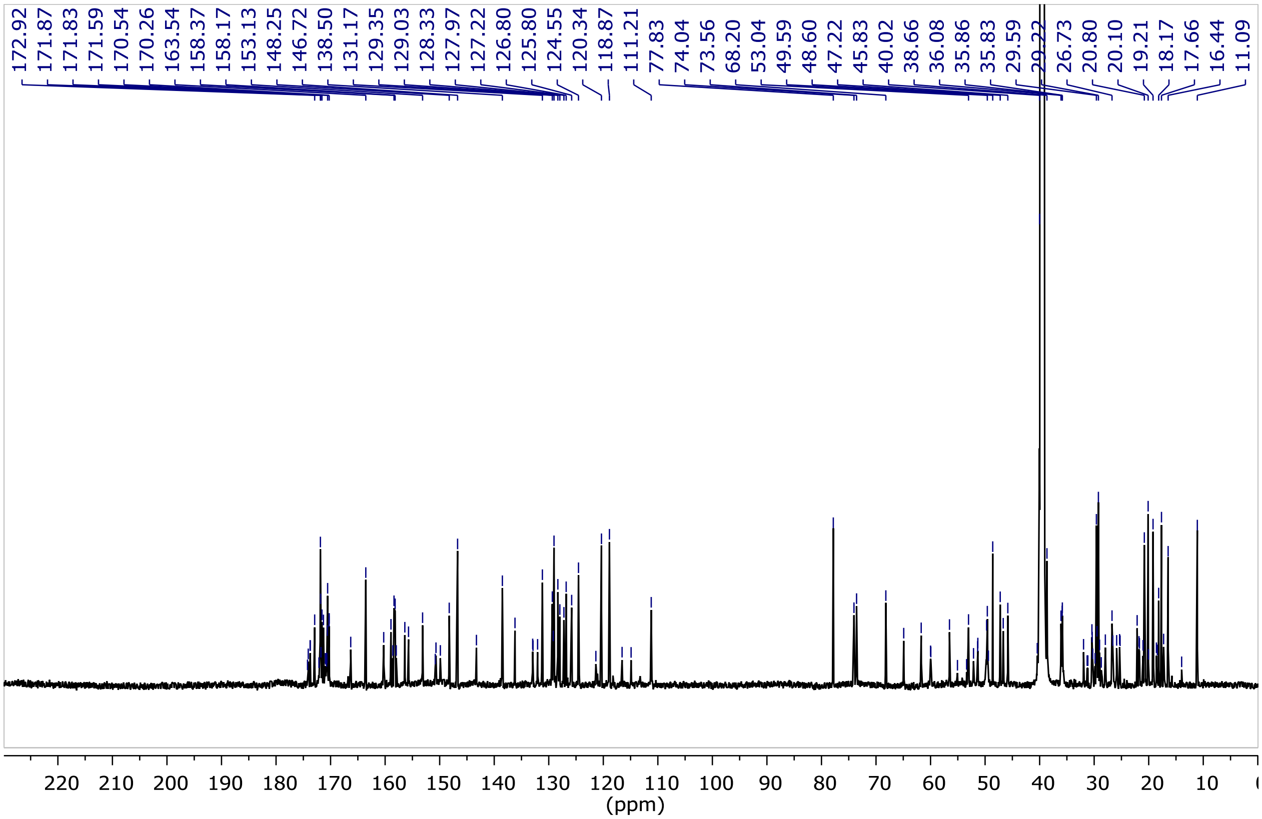

FA-6-Val-Cit-*p*ABA-16*R*-Aminoratjadone

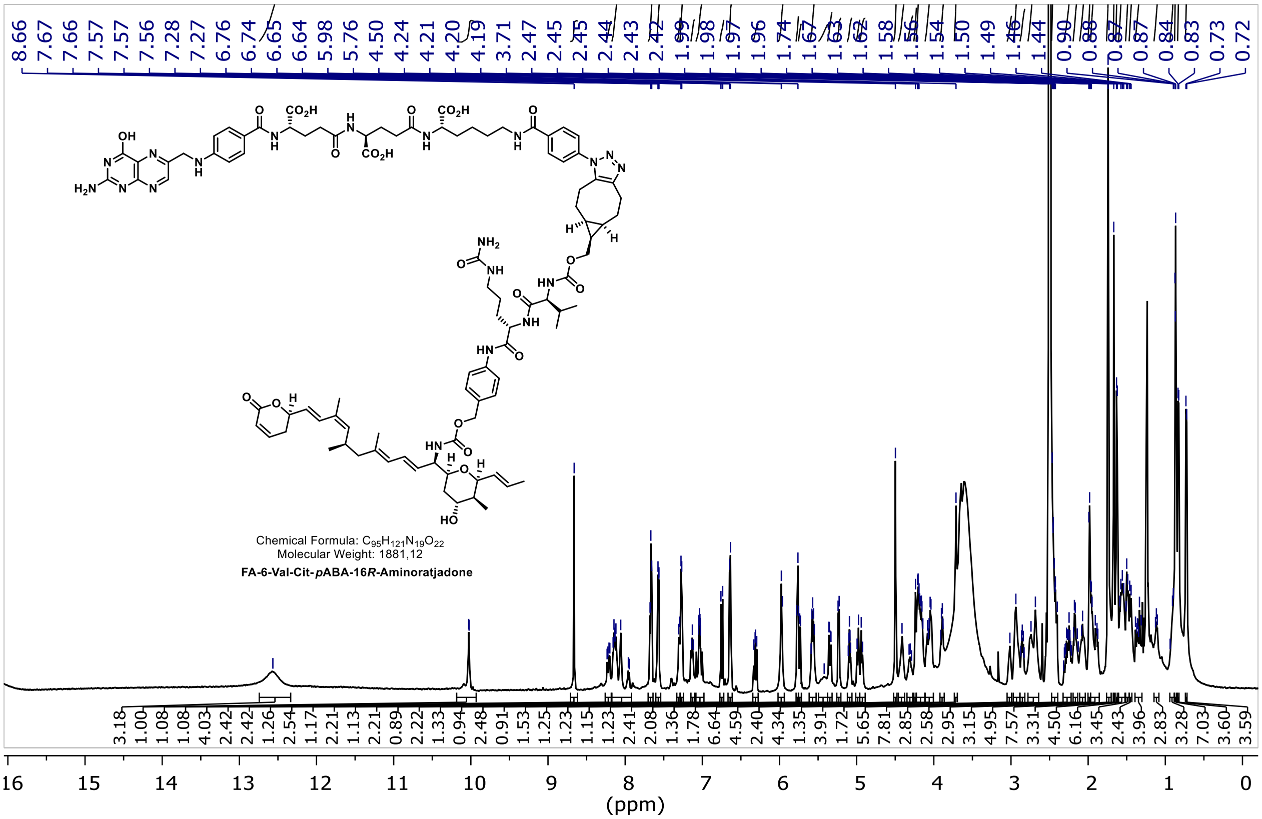

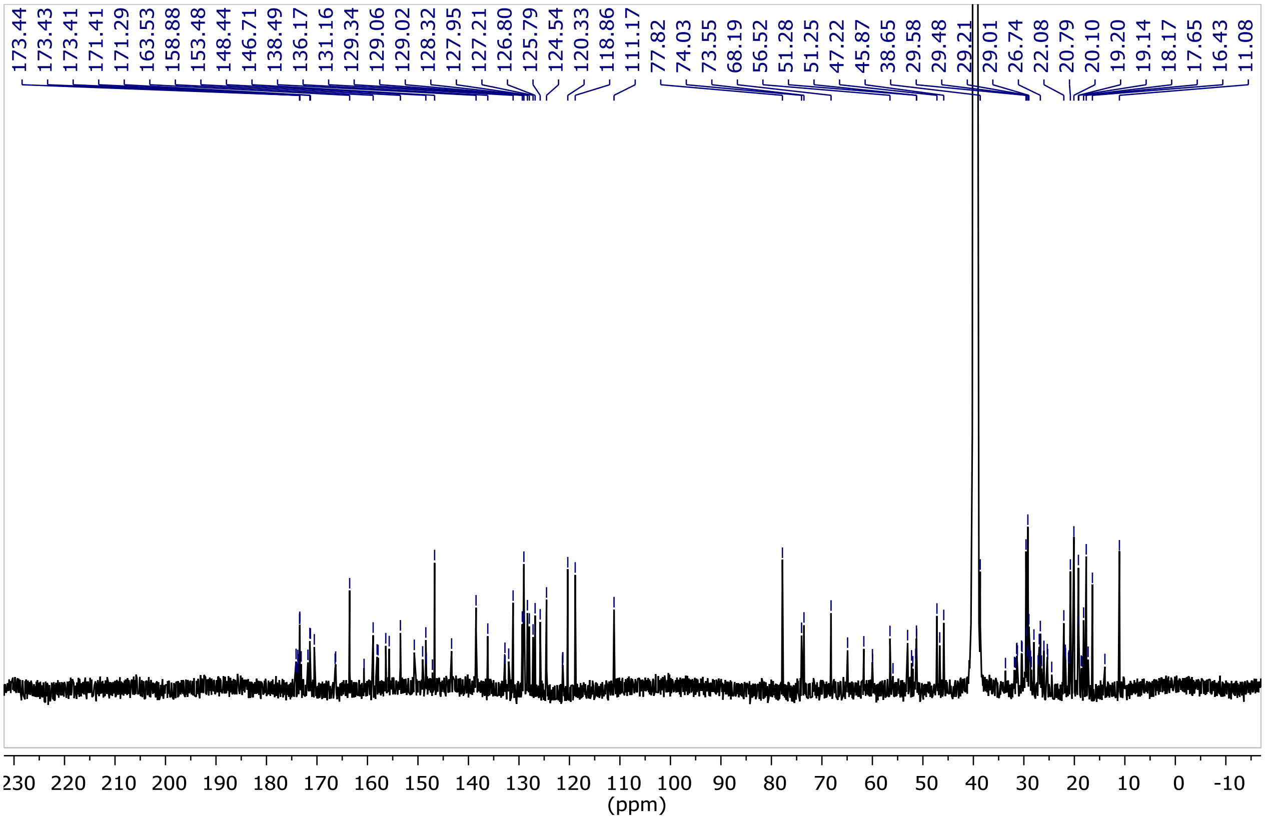

FA-7-Val-Cit-*p*ABA-16*R*-Aminoratjadone

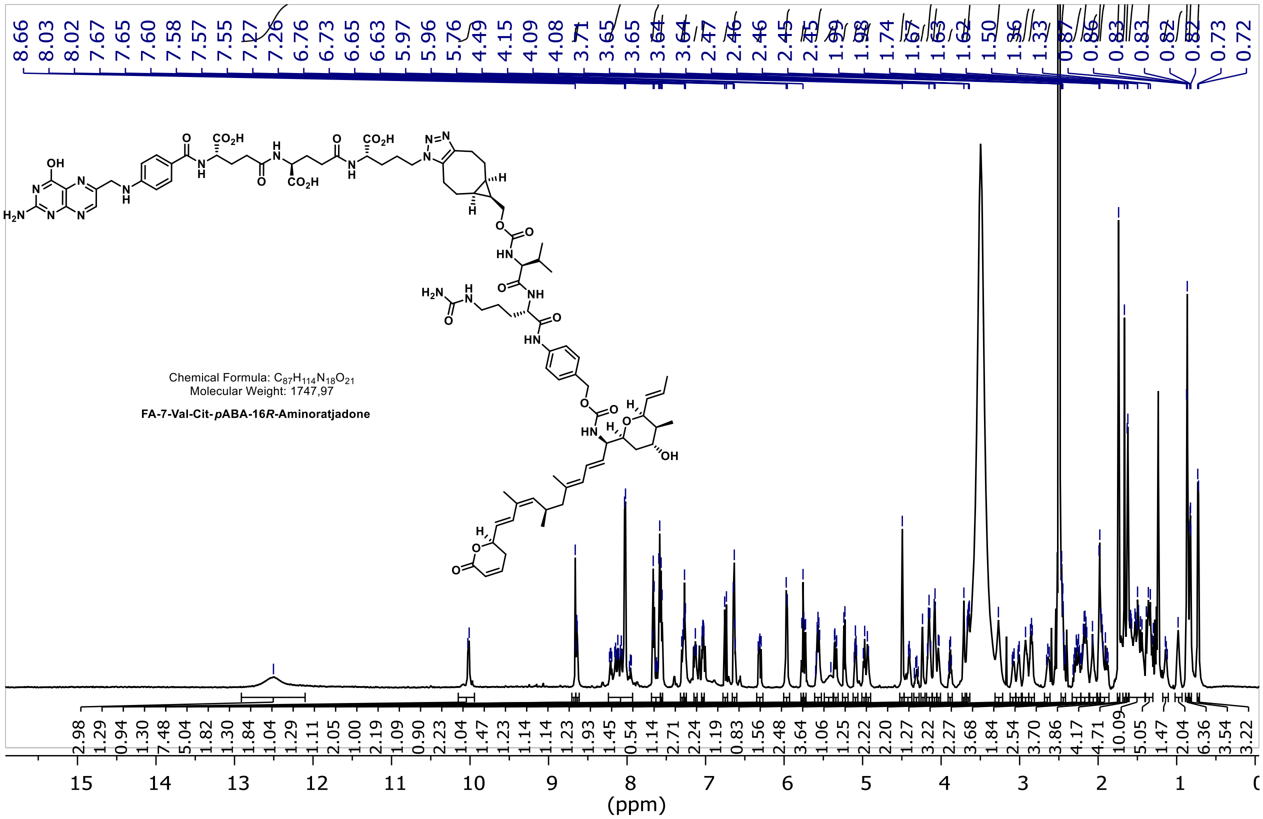

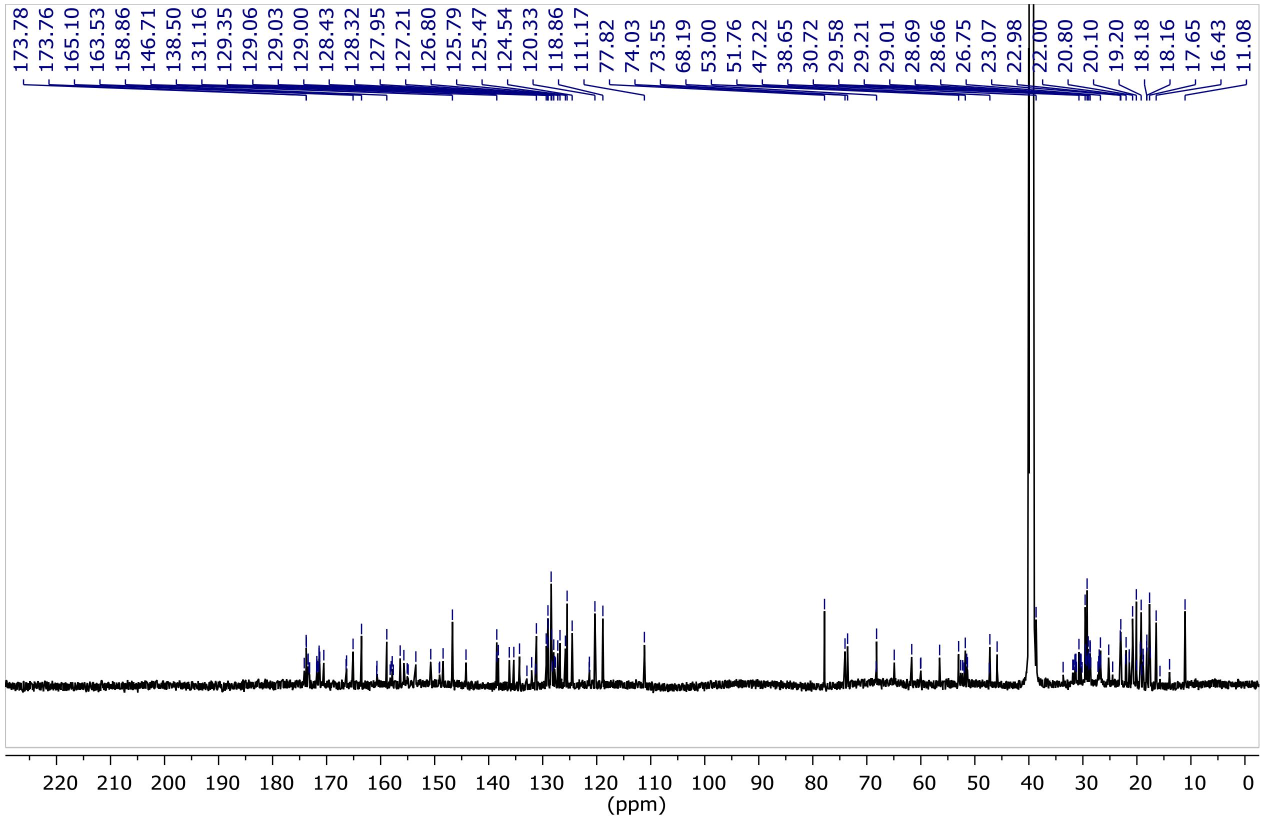

FA-8-Val-Cit-*p*ABA-16*R*-Aminoratjadone

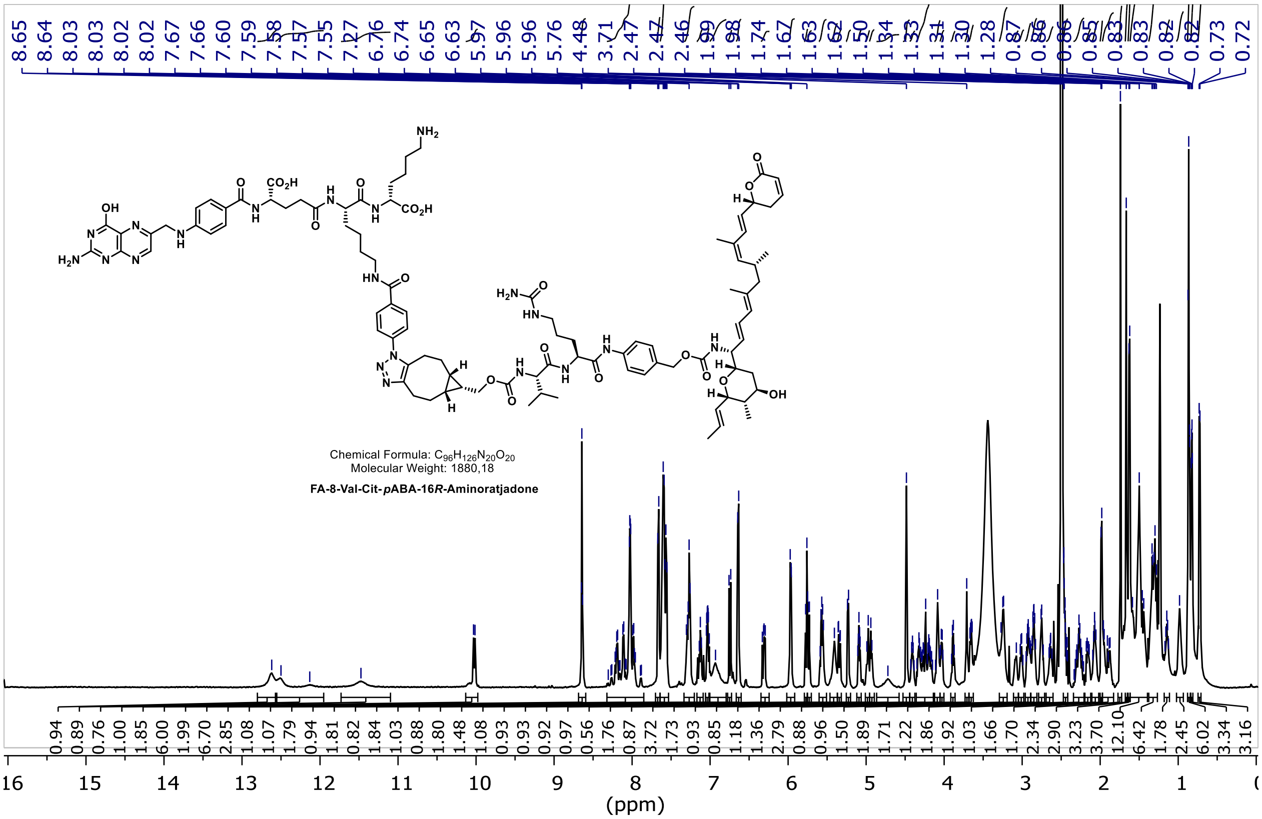

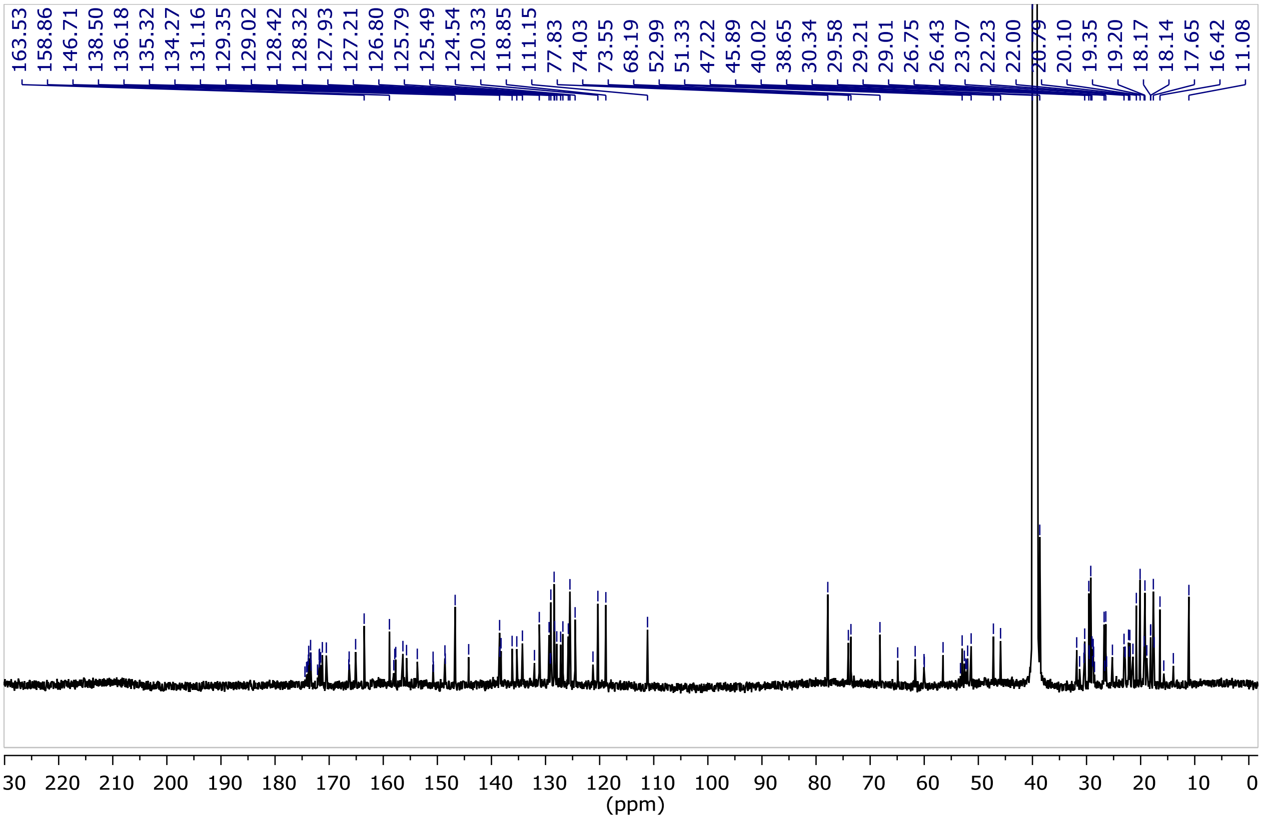

FA-9-Val-Cit-*p*ABA-16*R*-Aminoratjadone

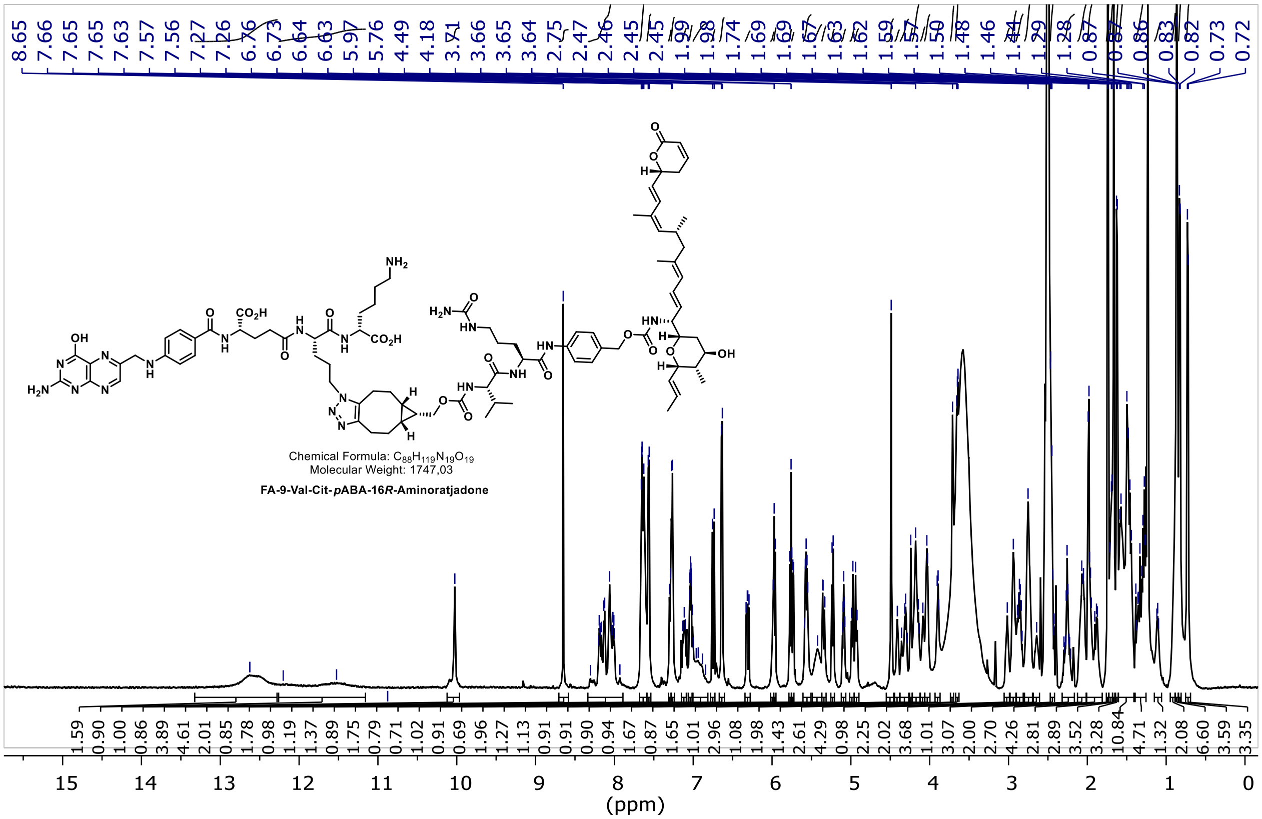

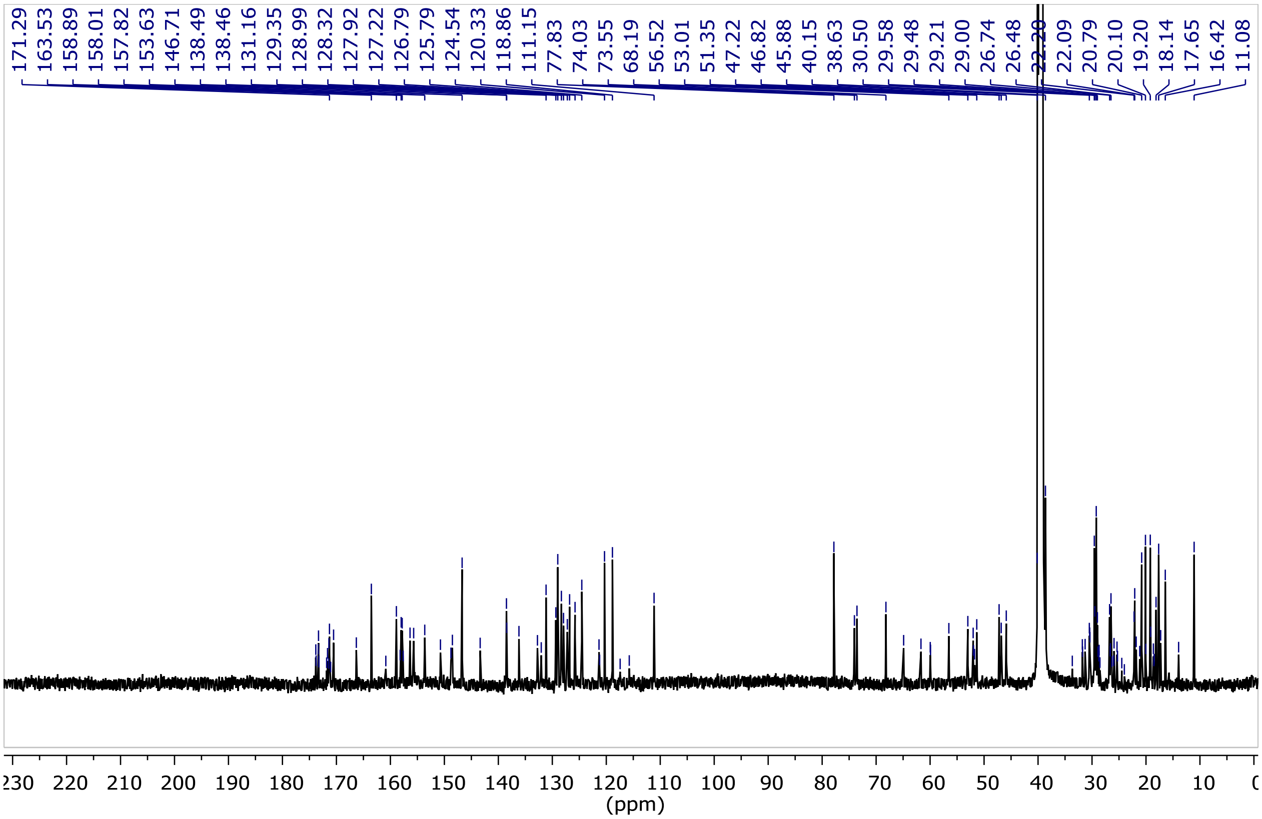

FA-10-Val-Cit-*p*ABA-16*R*-Aminoratjadone

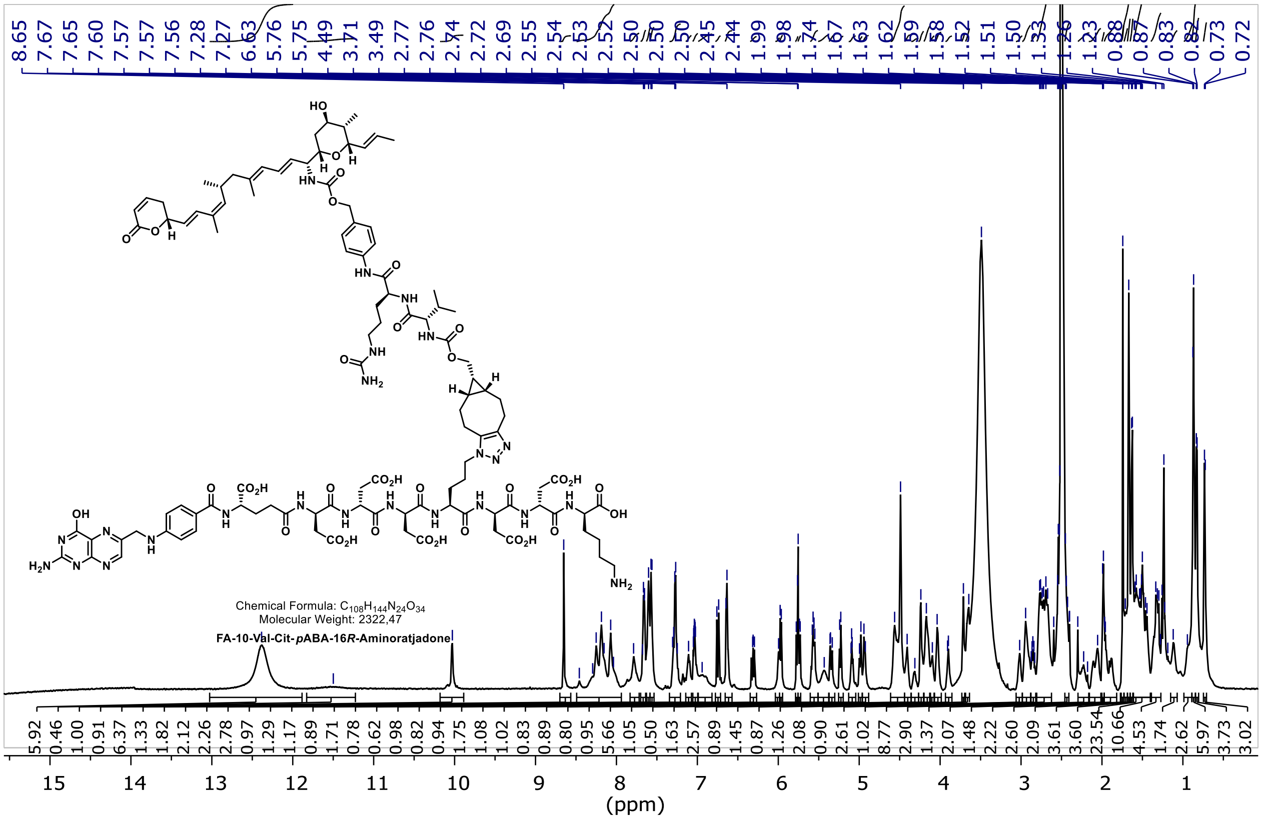

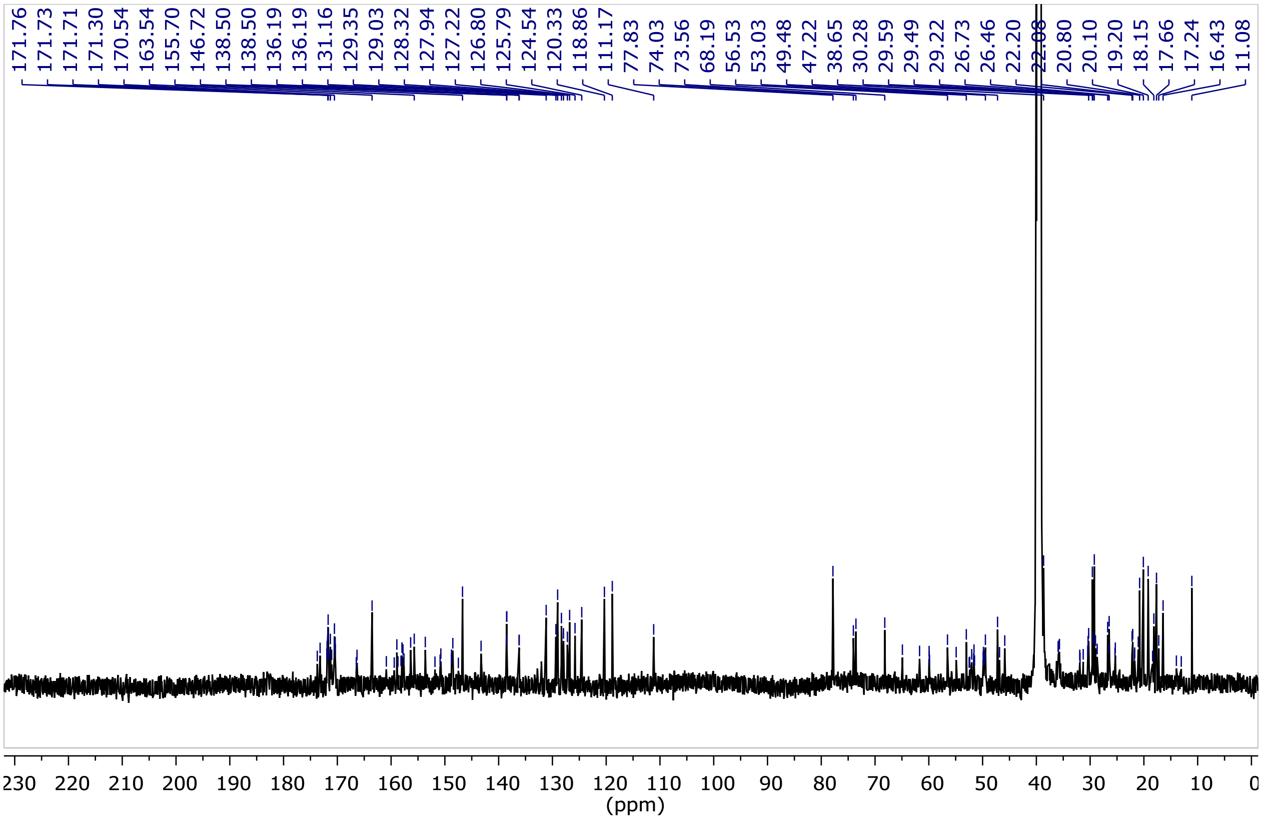

FA-11-(Val-Cit-*p*ABA-16*R*-Aminoratjadone)$_3$

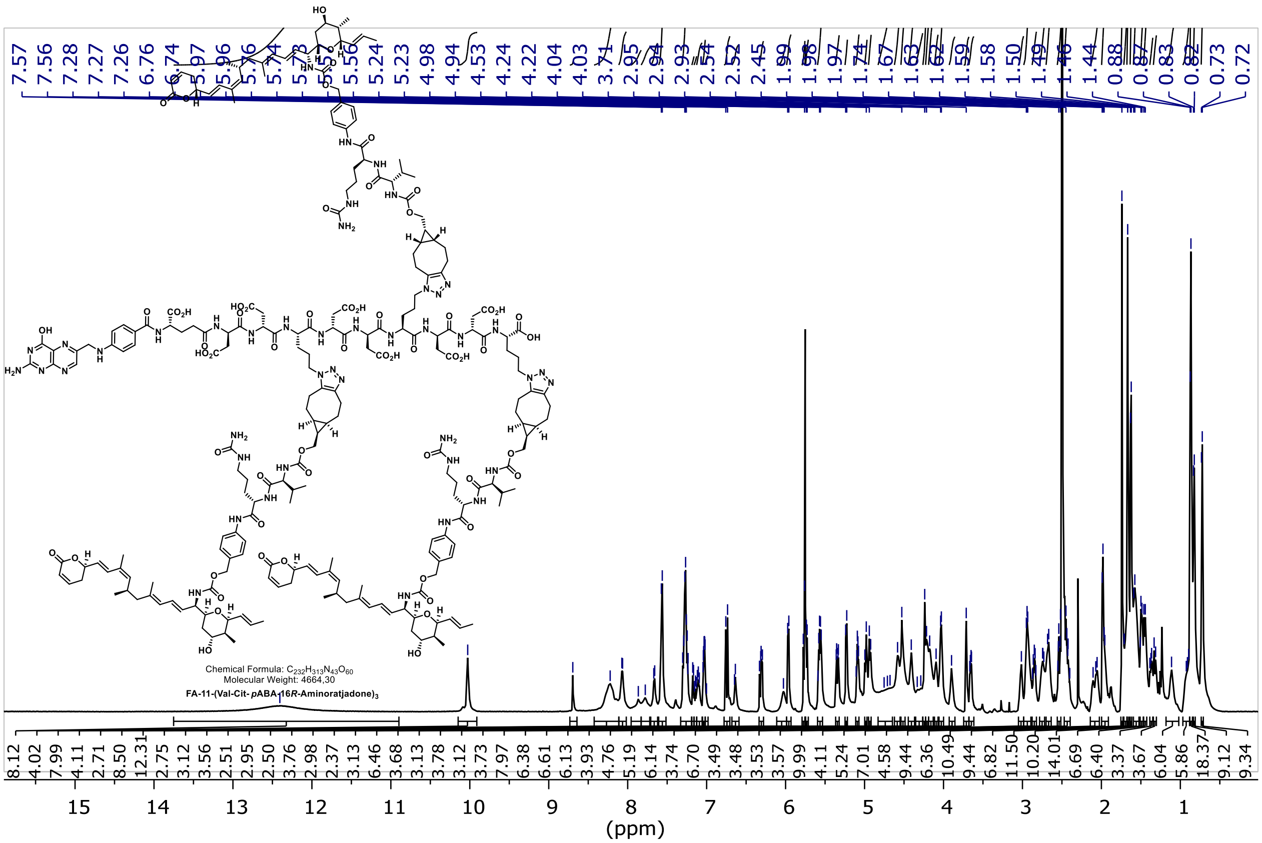

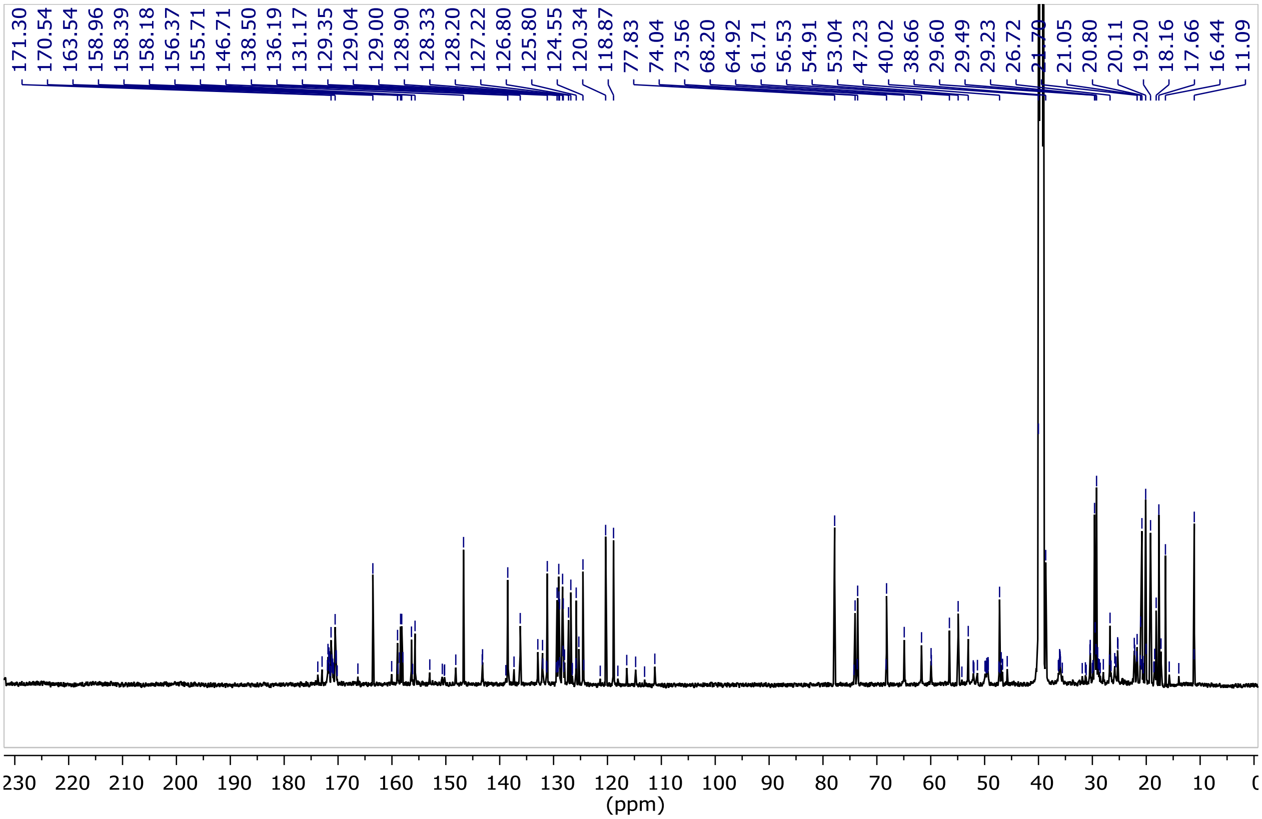

FA-3-L-16*R*-Aminoratjadone

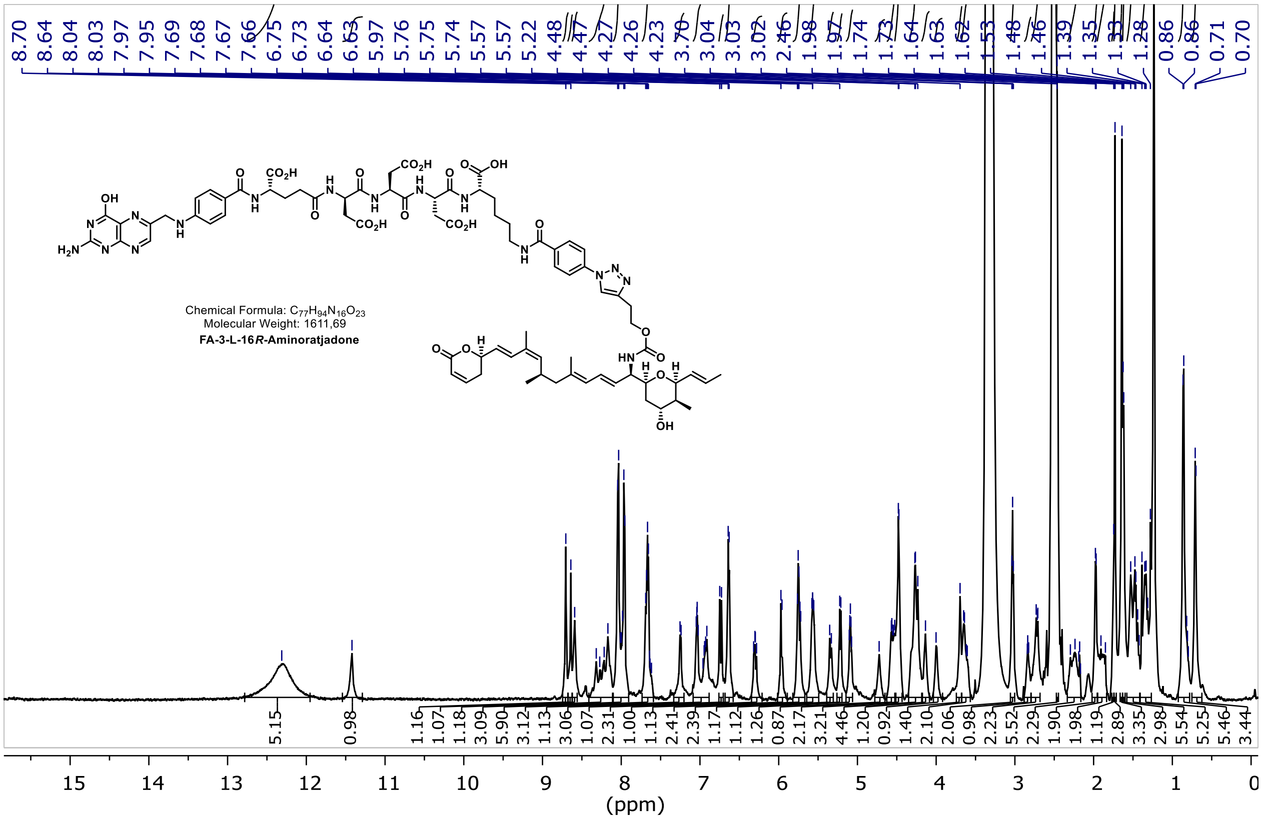

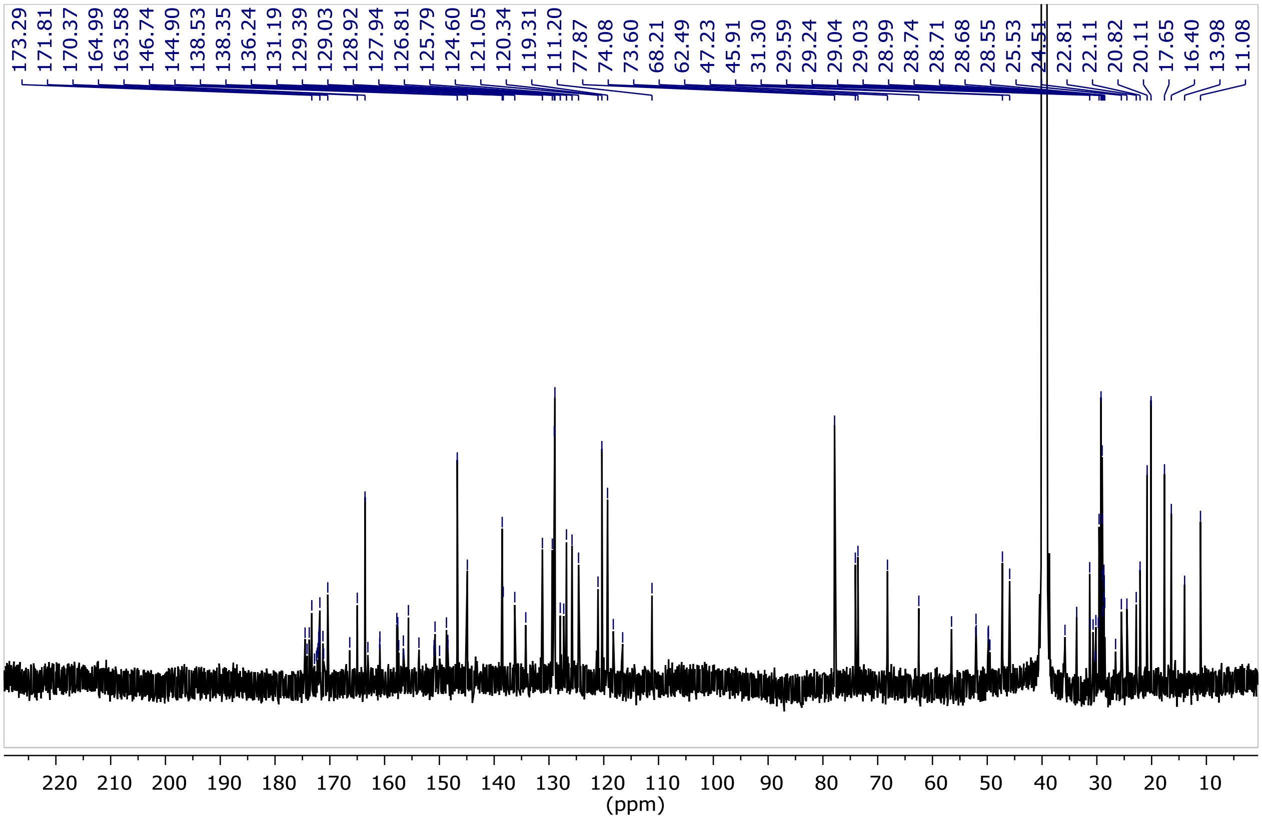

FA-3b-L-16*R*-Aminoratjadone

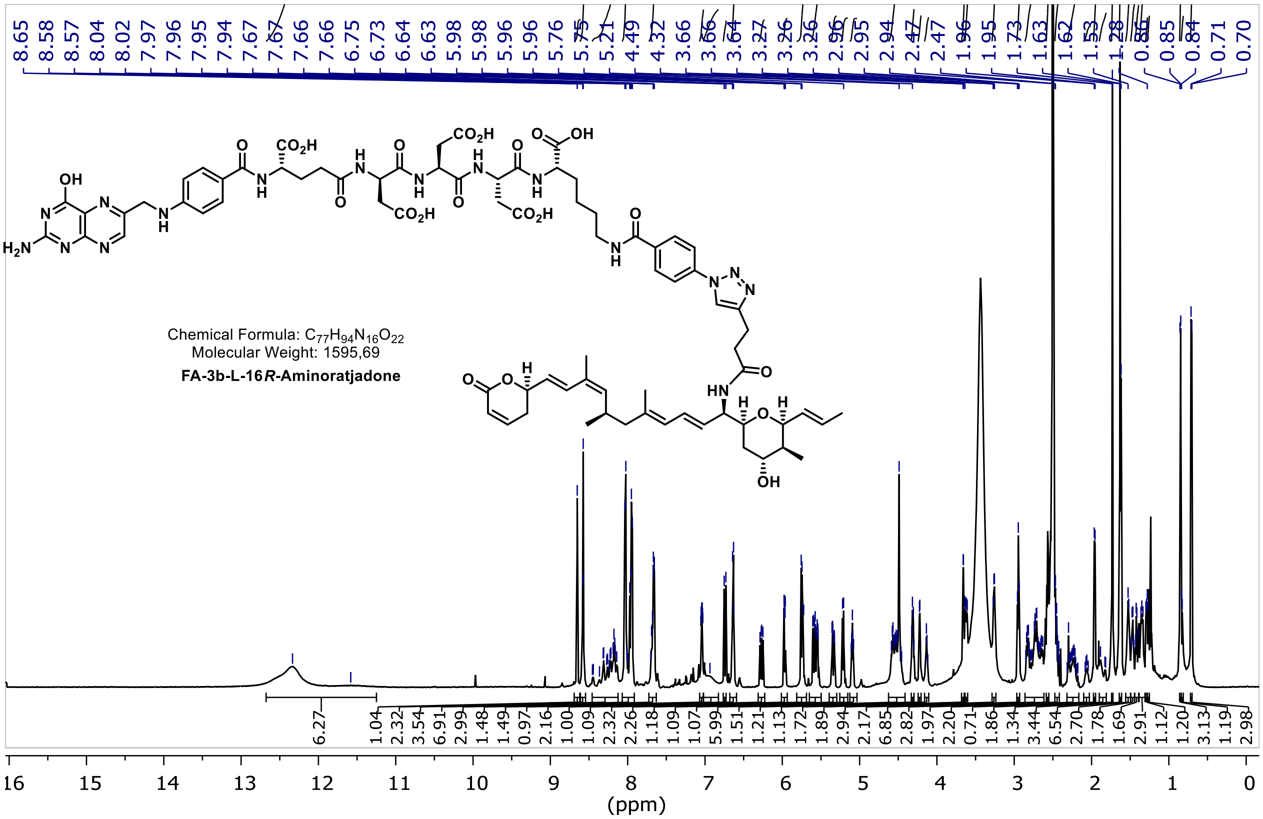

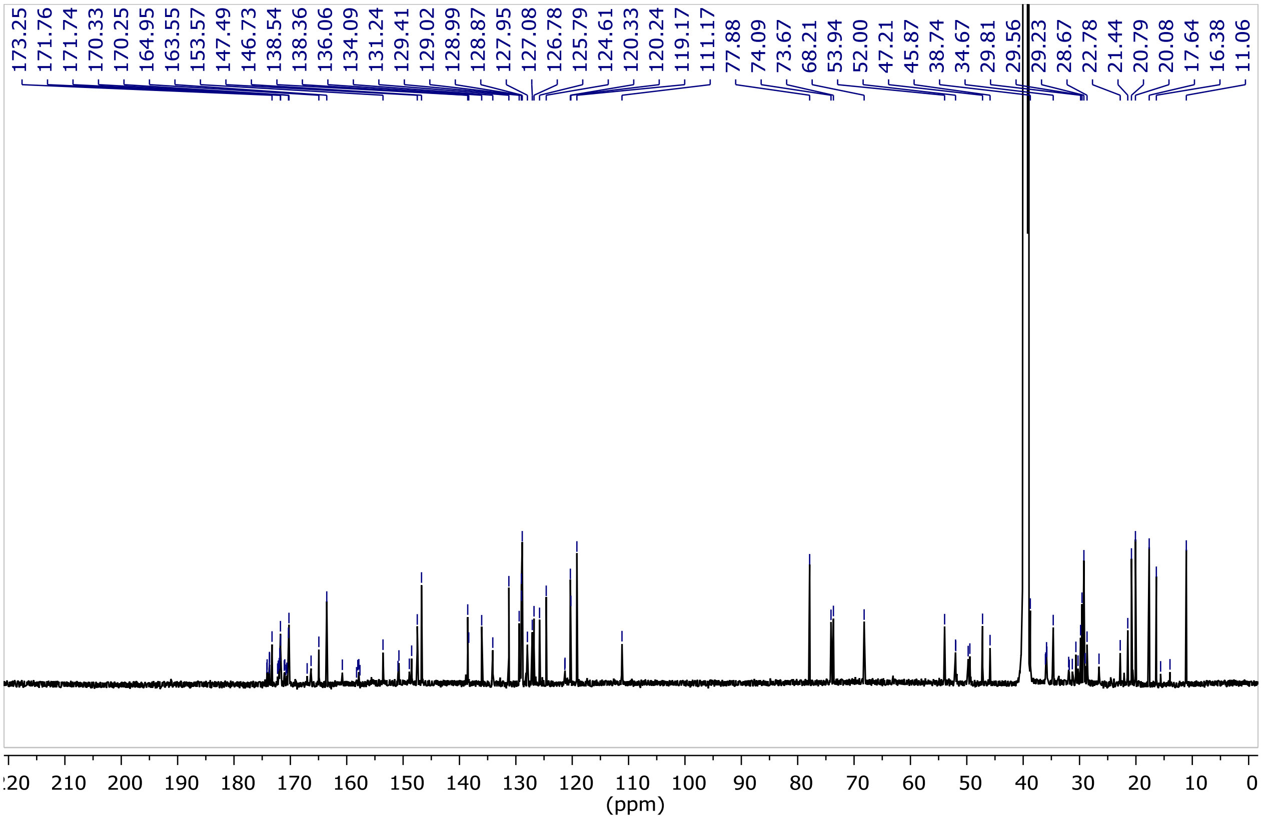

FA-5-L-16*R*-Aminoratjadone

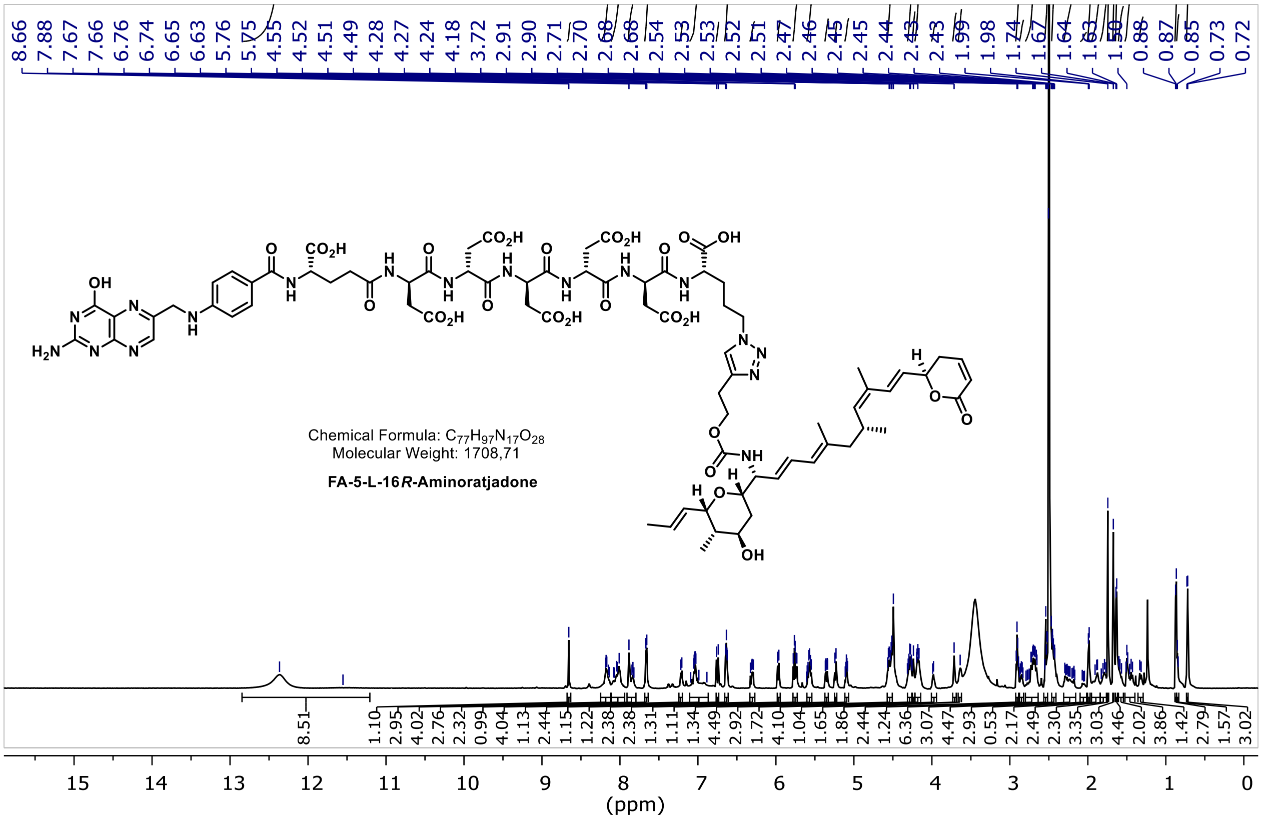

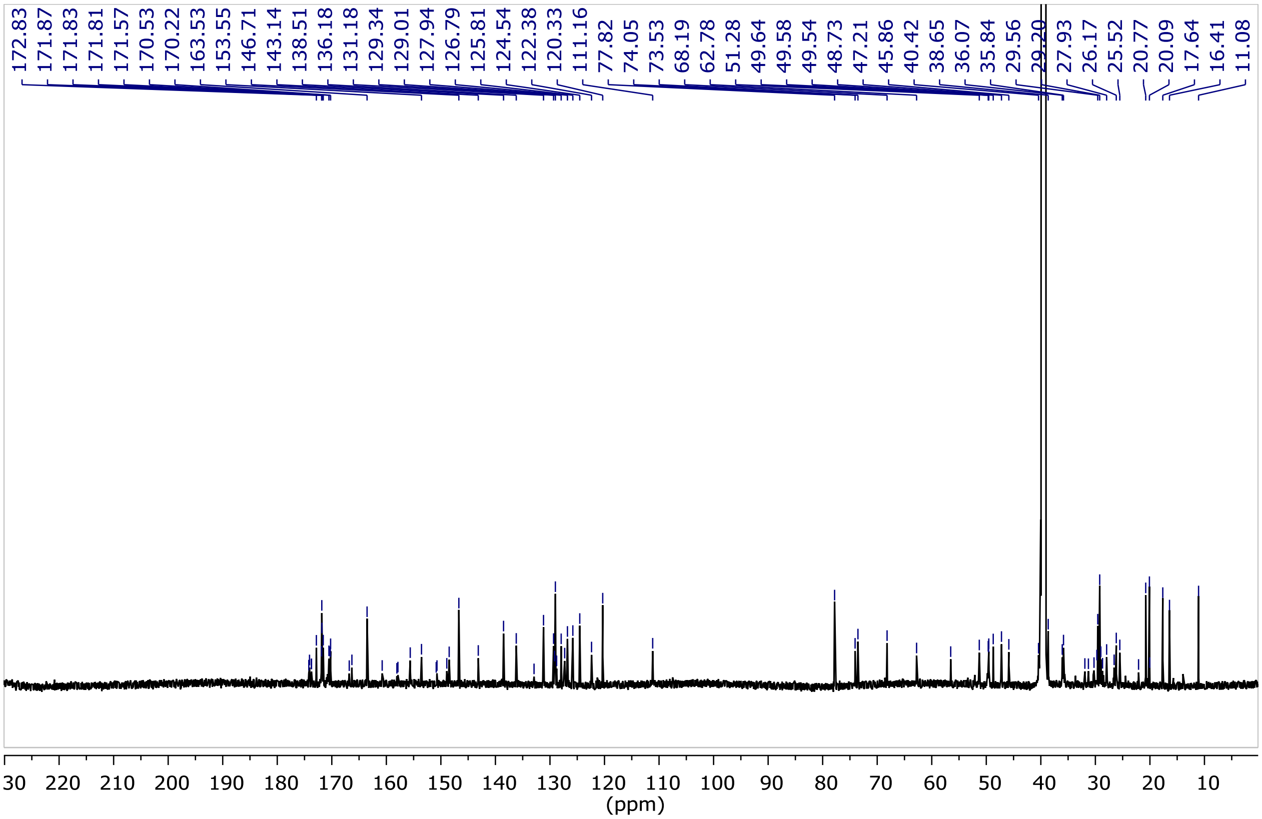

FA-6-L-16R-Aminoratjadone

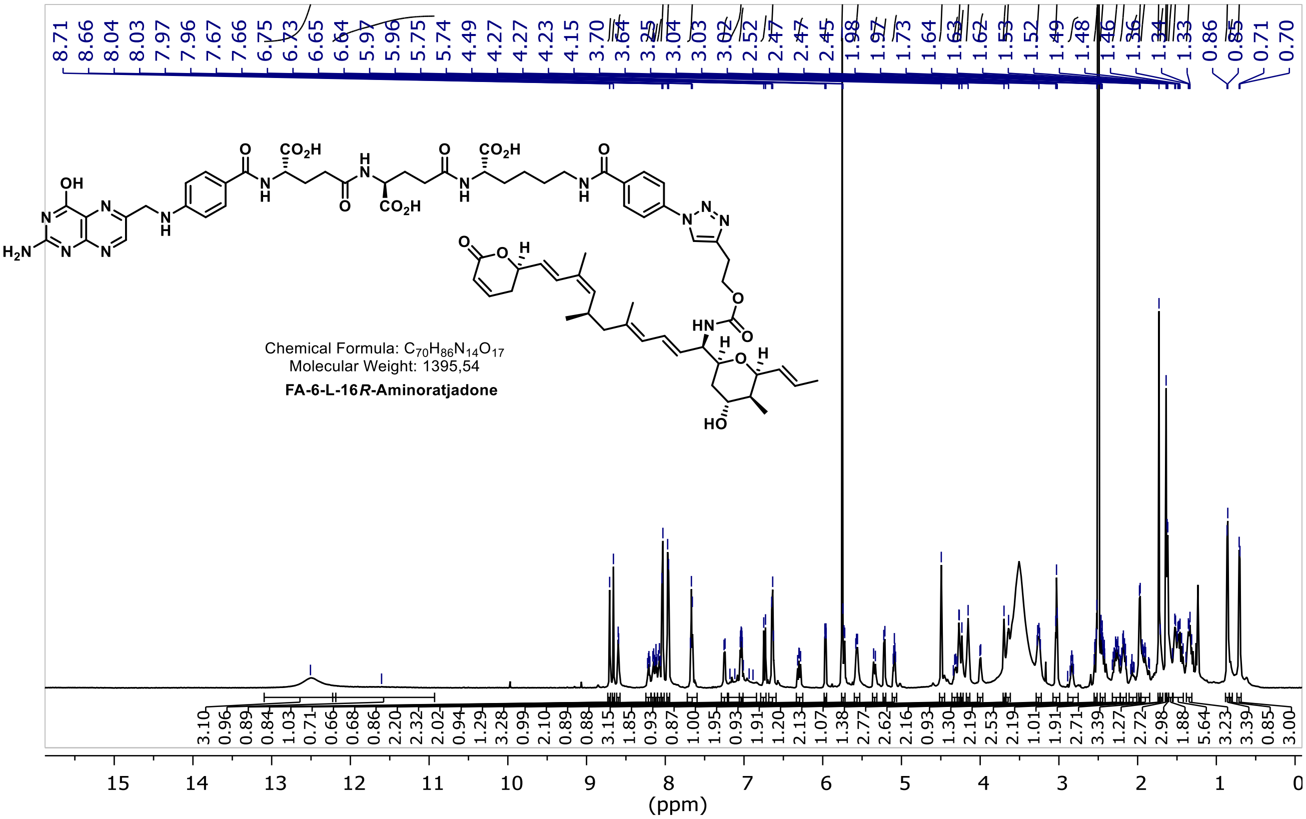

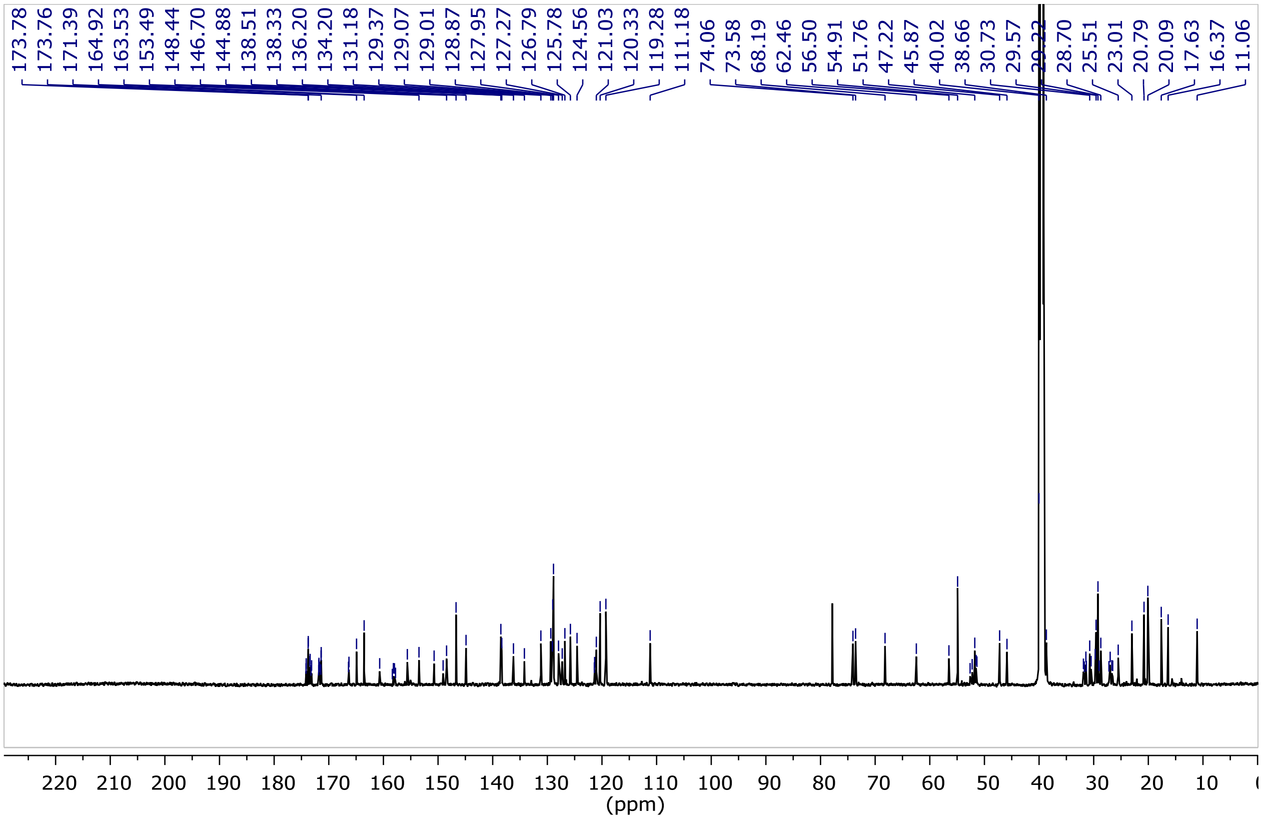

FA-7-L-16R-Aminoratjadone

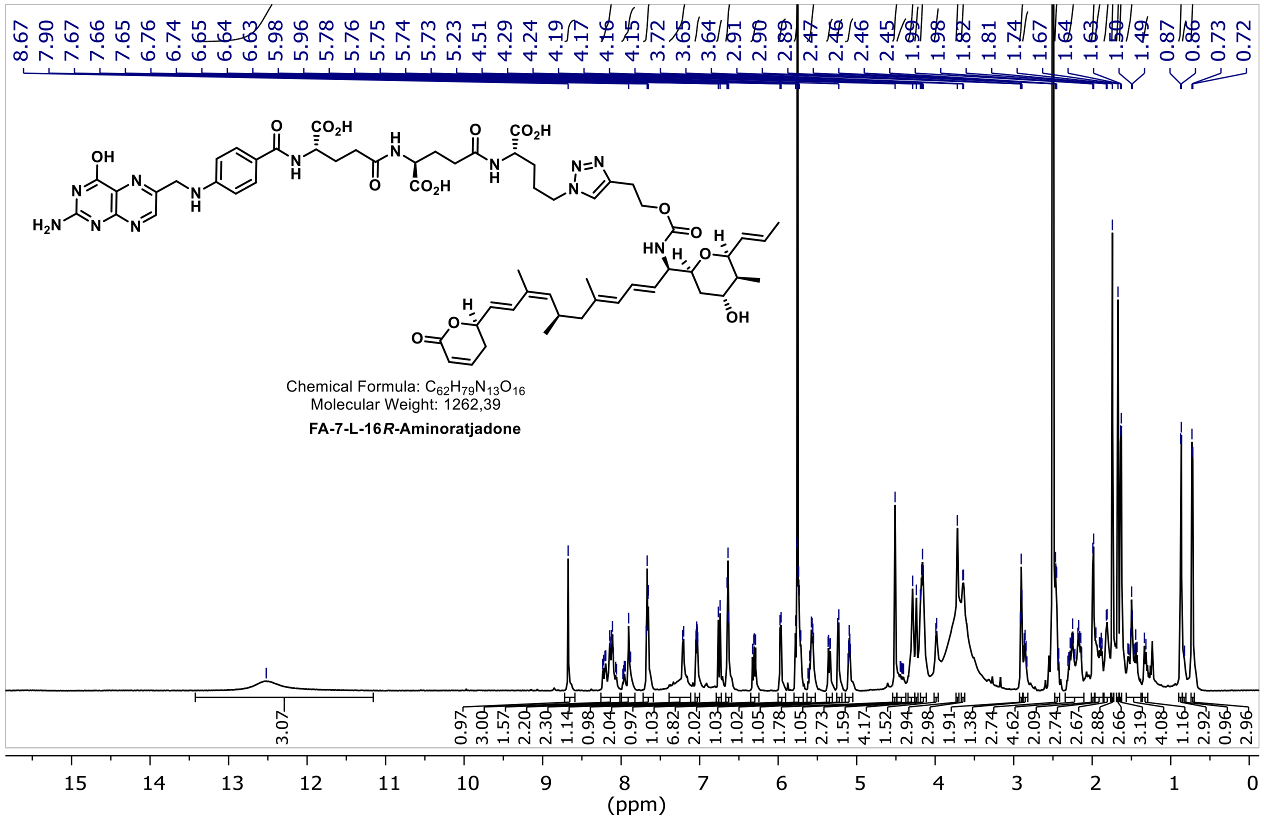

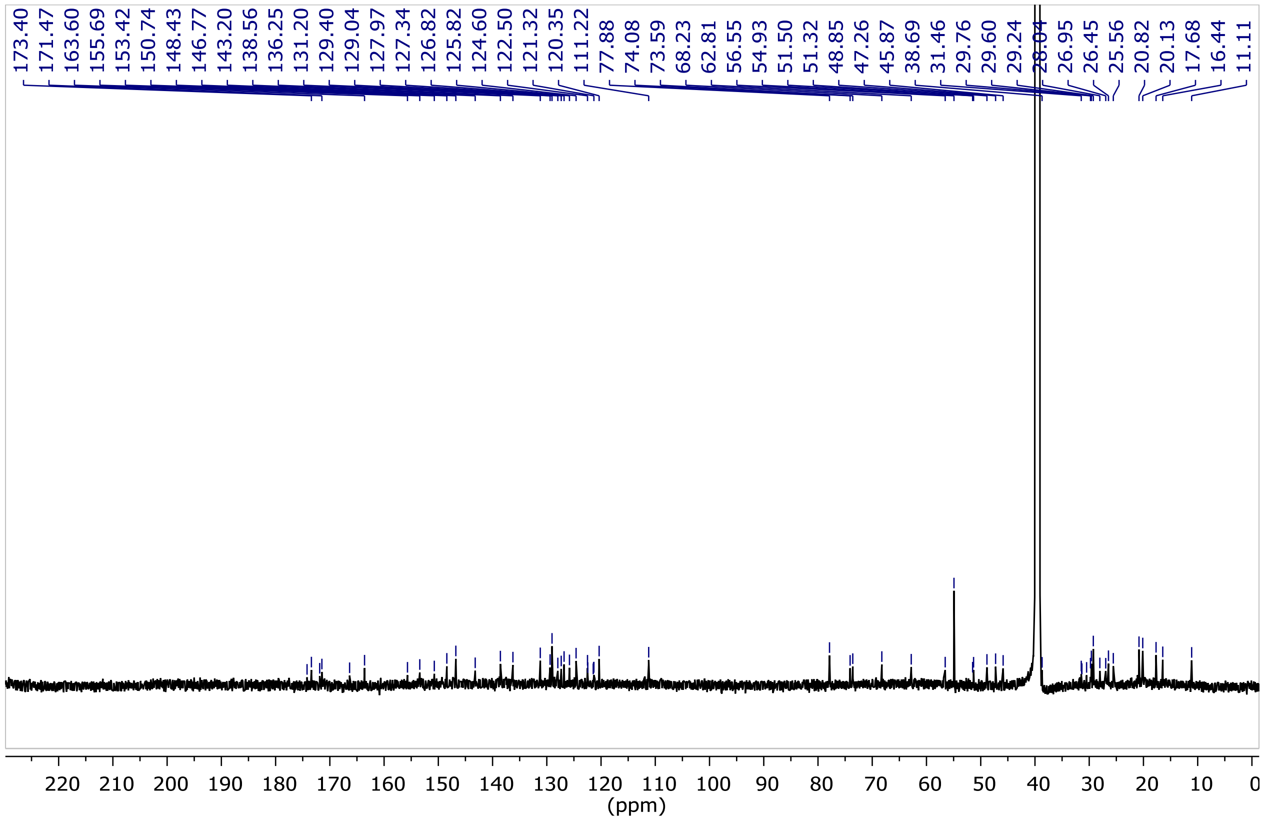

FA-10-L-16R-Aminoratjadone

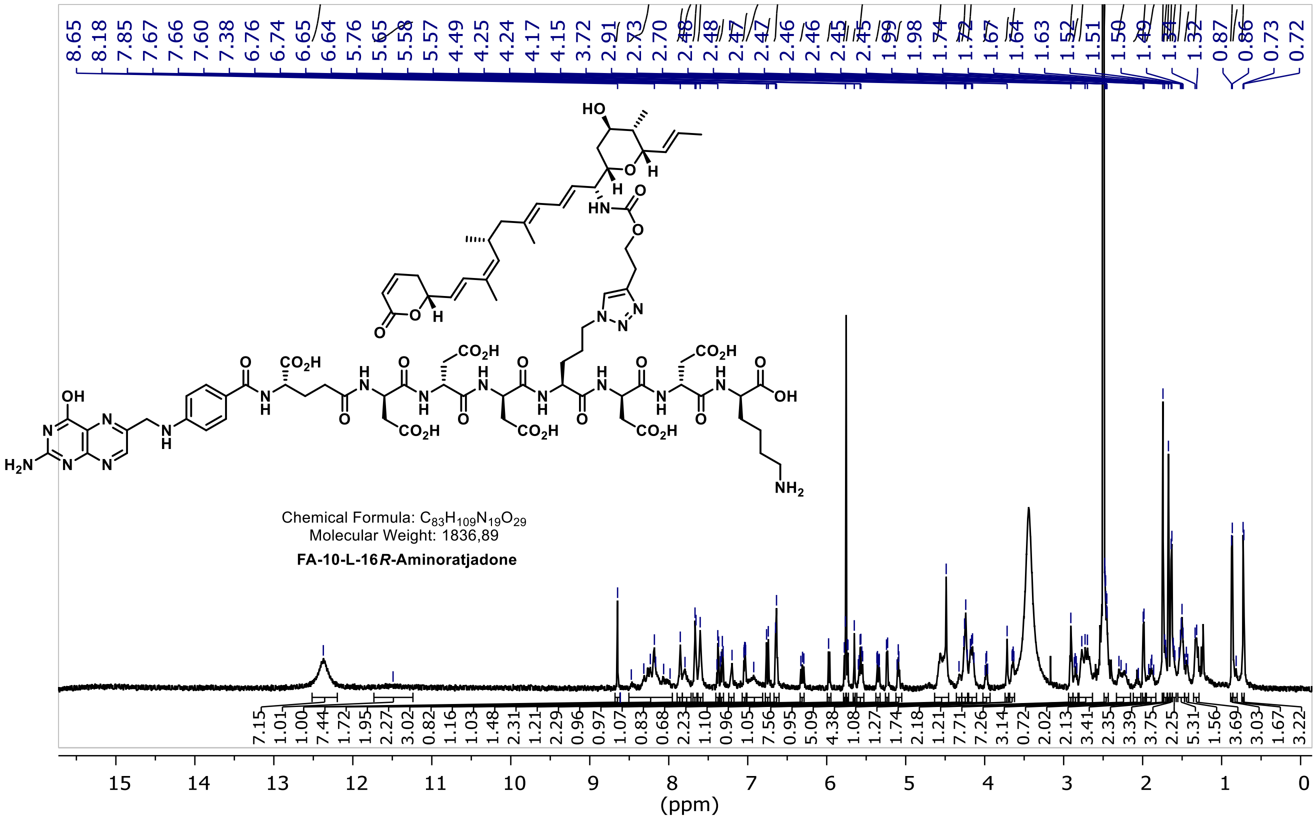

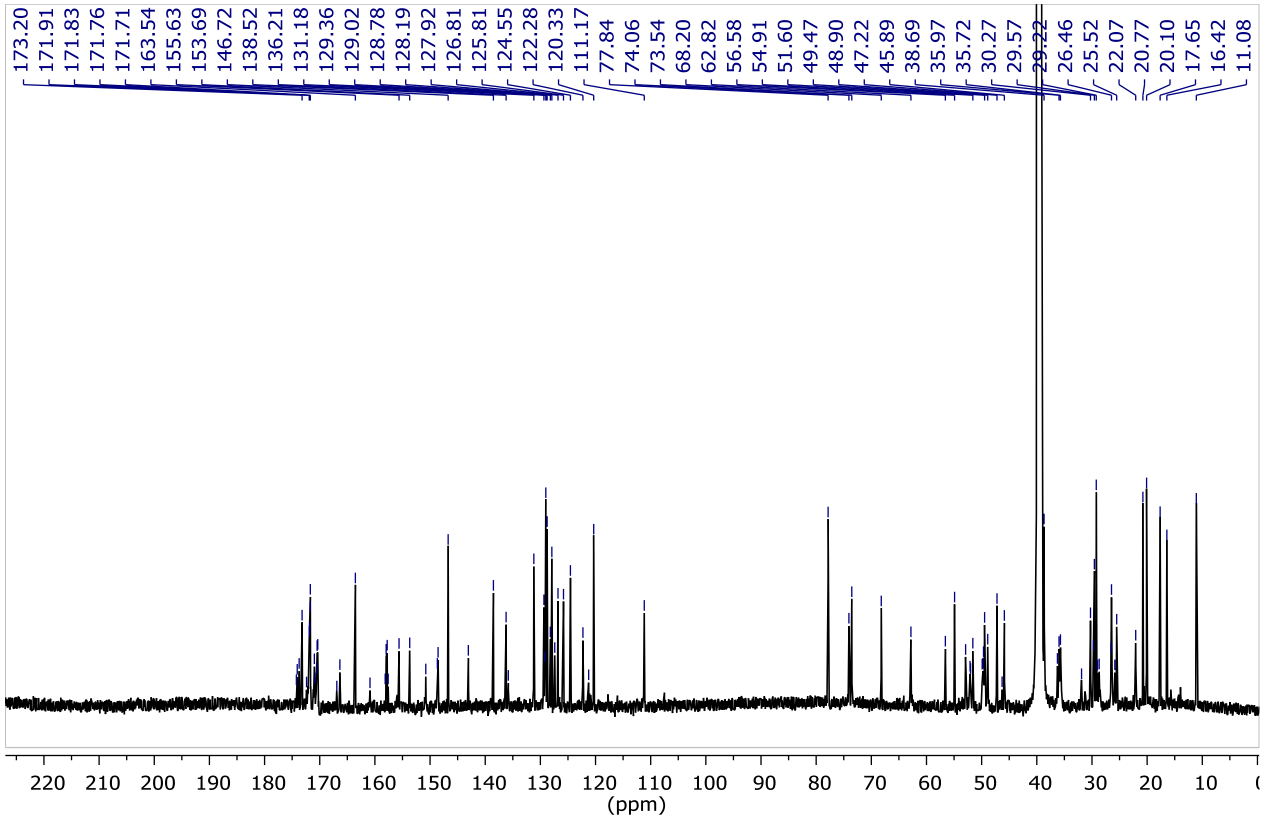

FA-SS-16*R*-Aminoratjadone

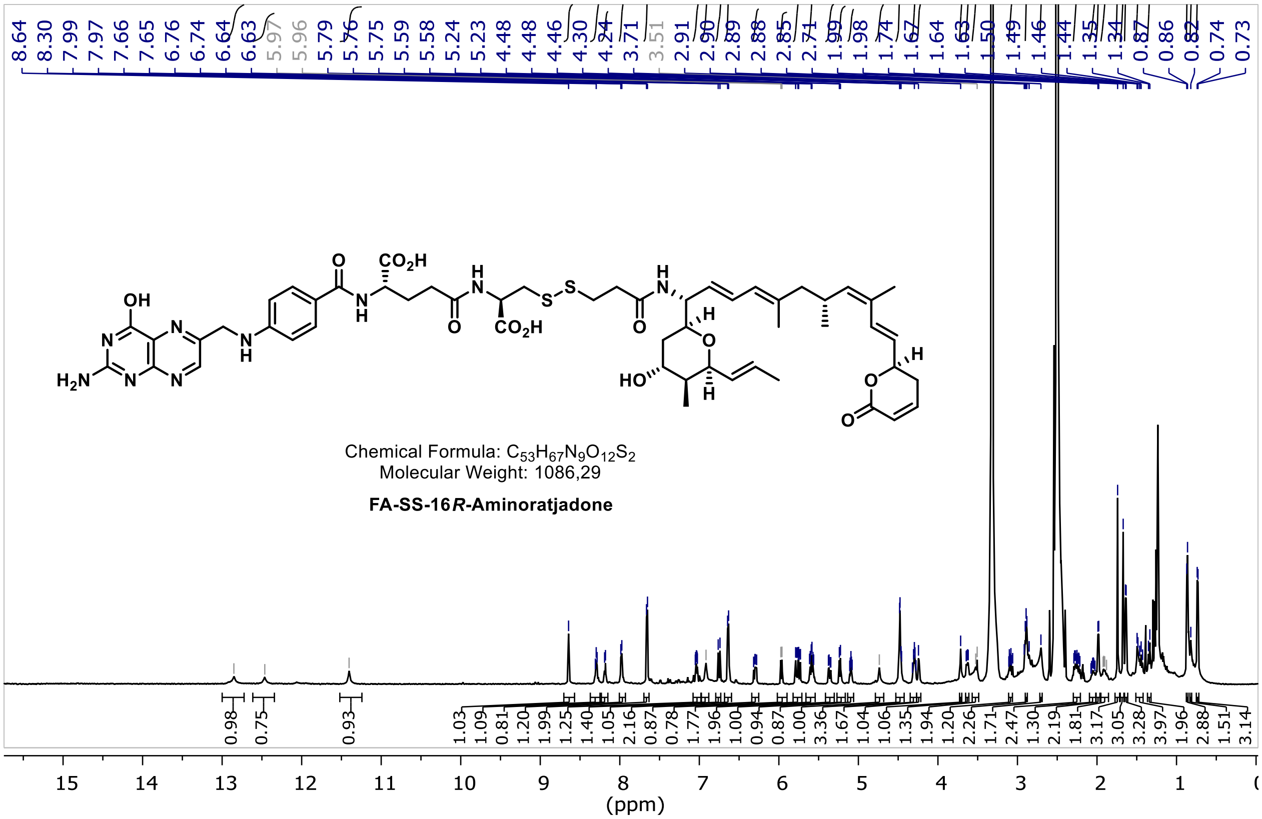

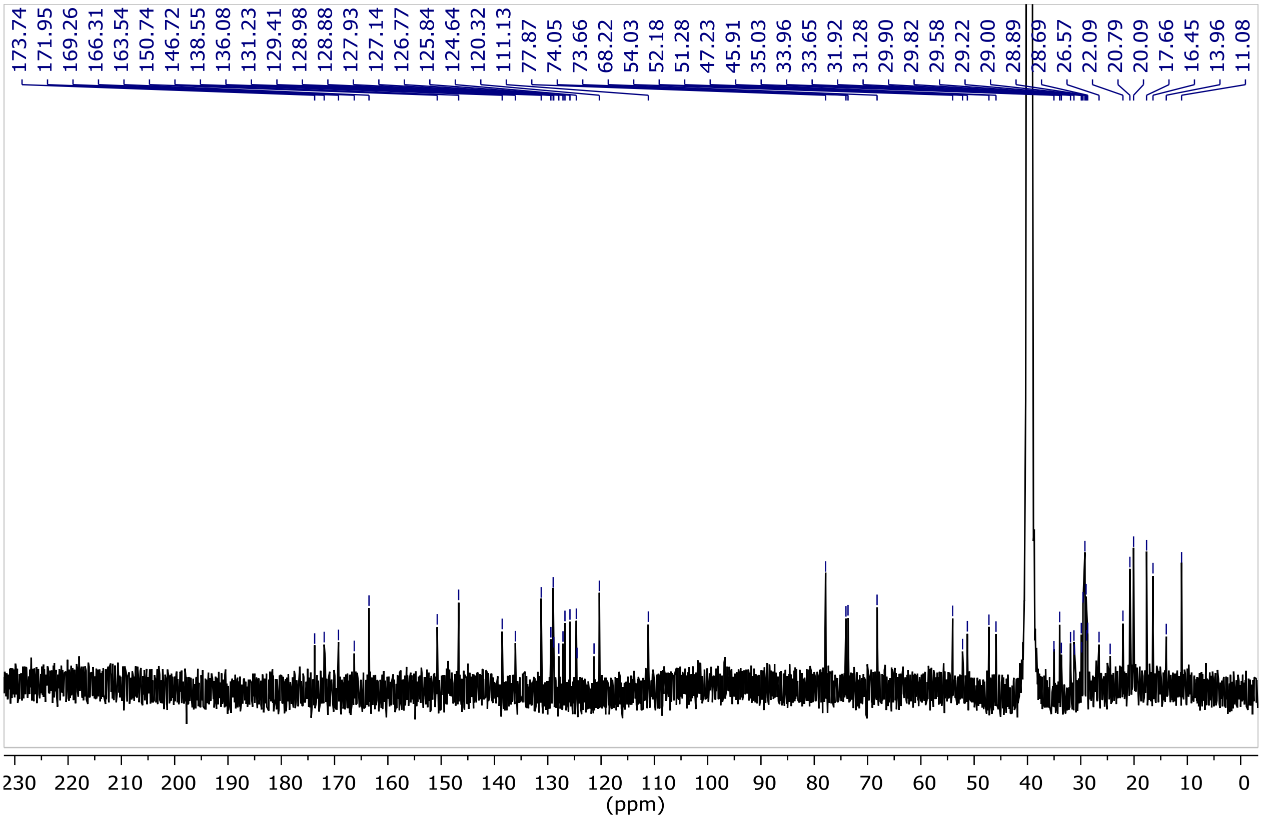

L-Orn-LHRH-16*R*-Aminoratjadone

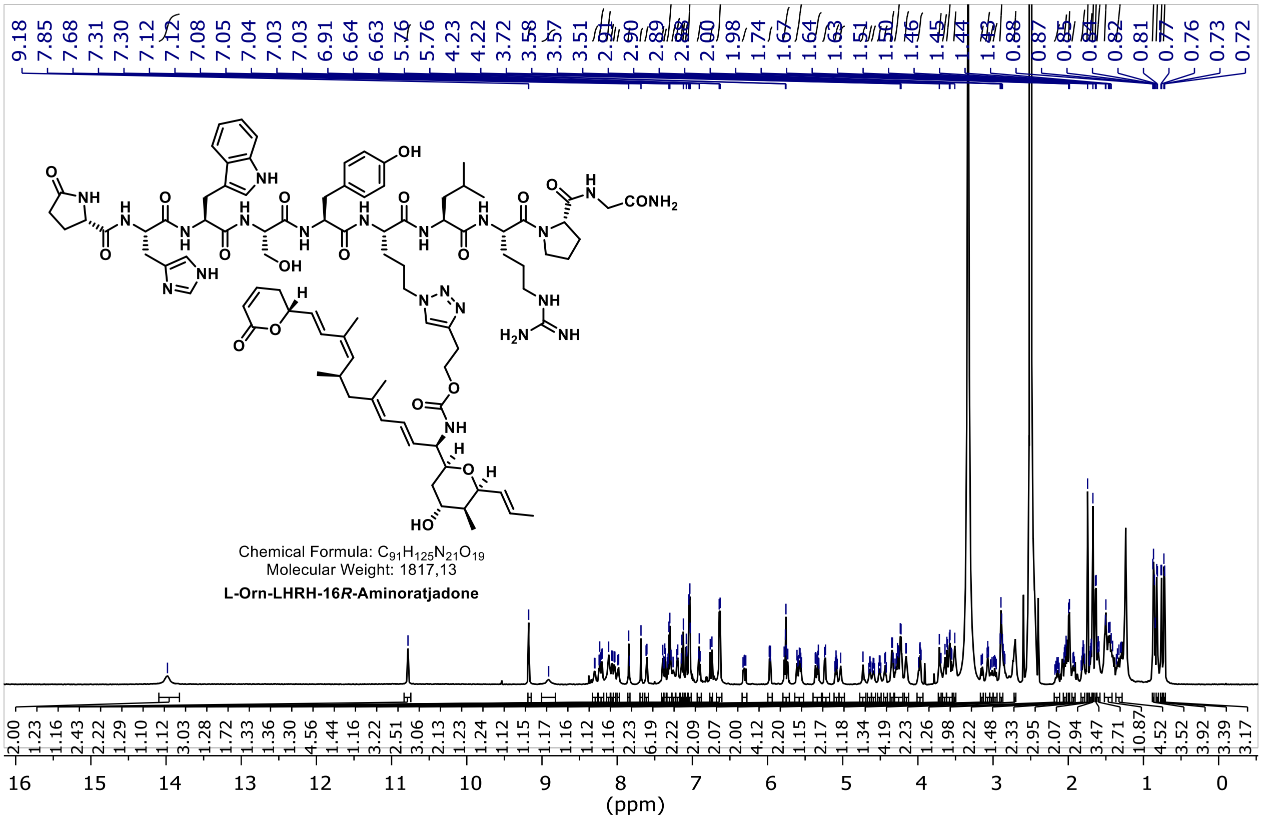

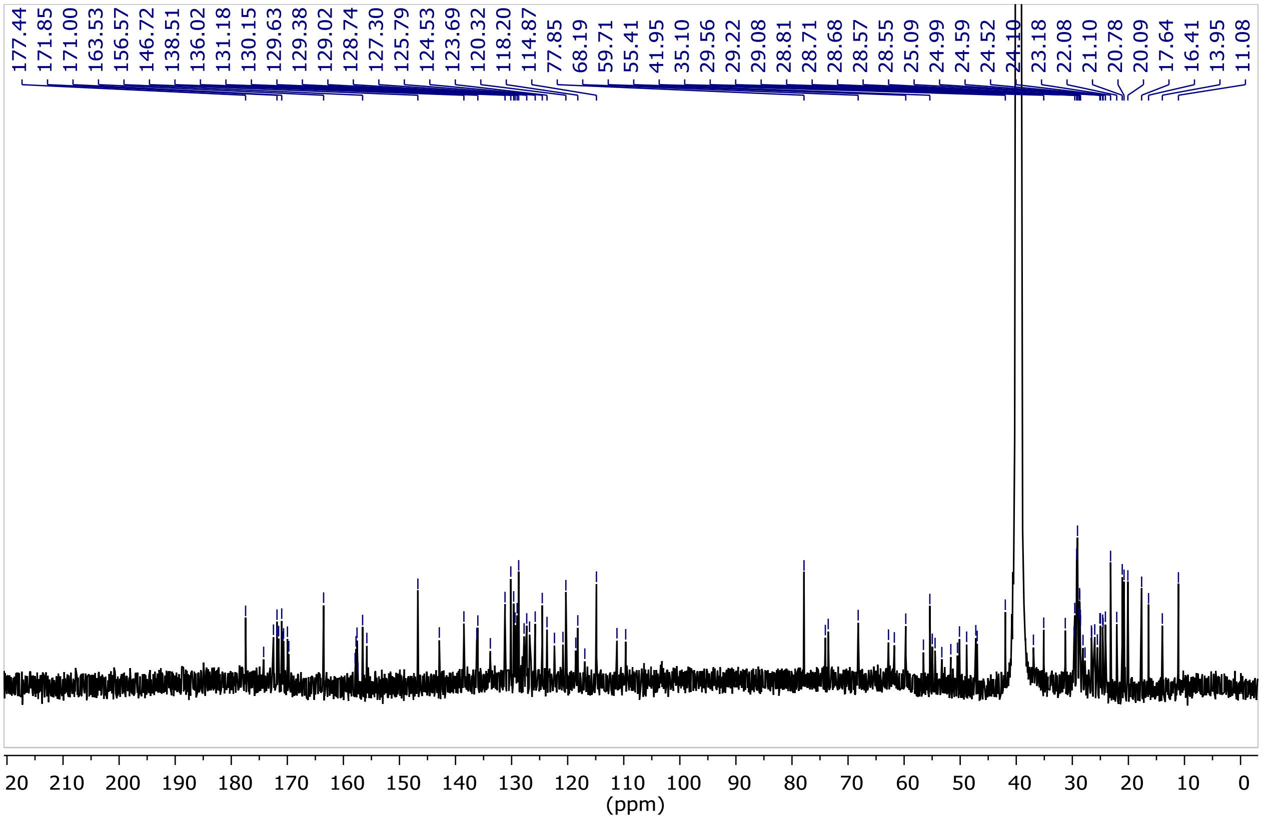

L-Orn-LHRH-Val-Cit-*p*ABA-16*R*-Aminoratjadone

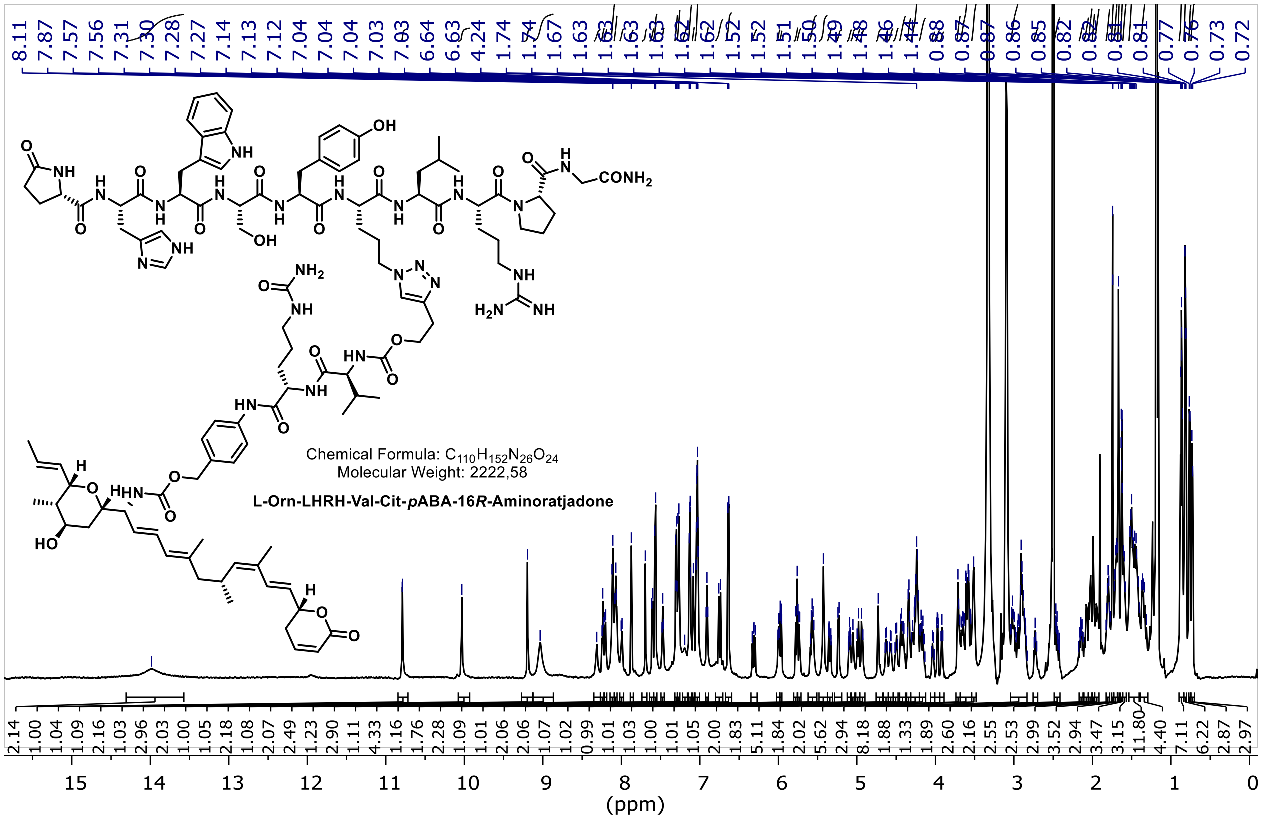

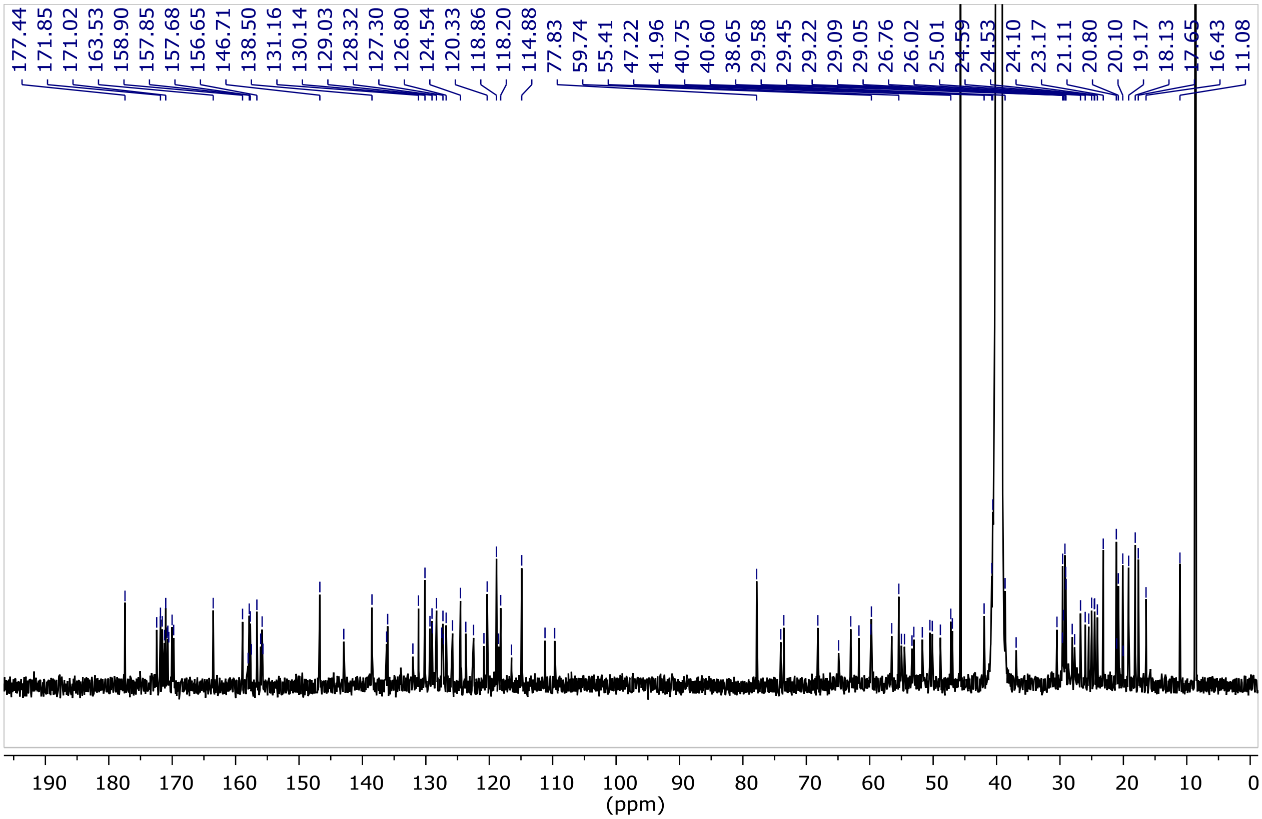

D-Orn-LHRH-Val-Cit-*p*ABA-16*R*-Aminoratjadone

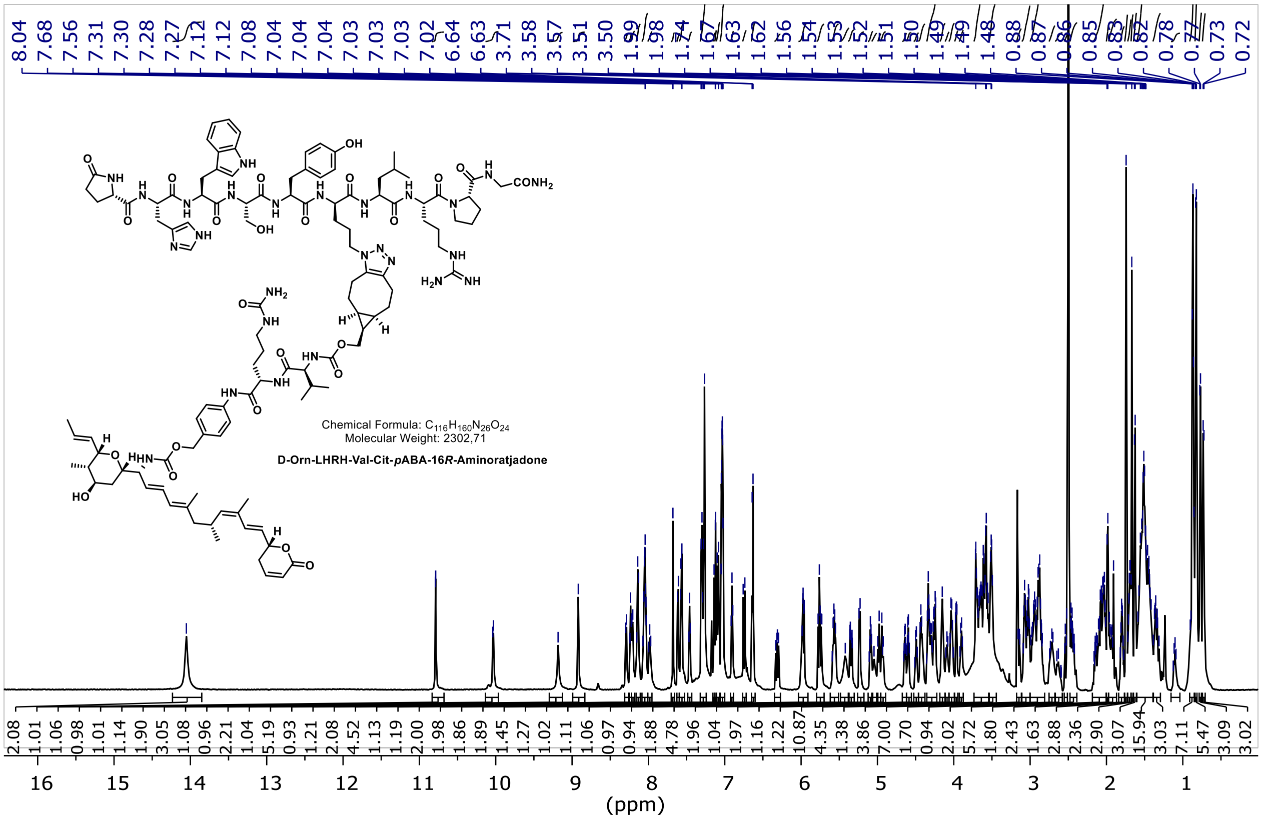

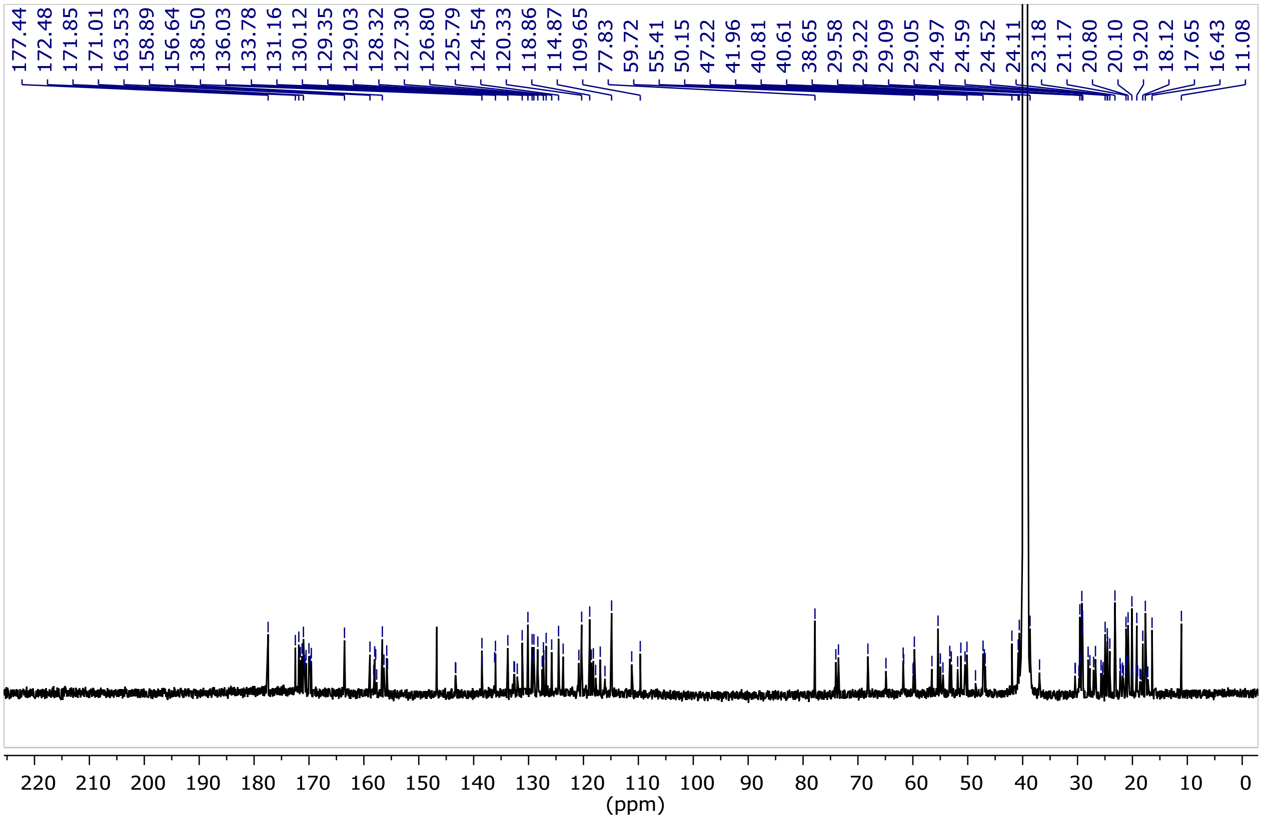

D-Orn-Goserellin-Val-Cit-*p*ABA-16*R*-Aminoratjadone

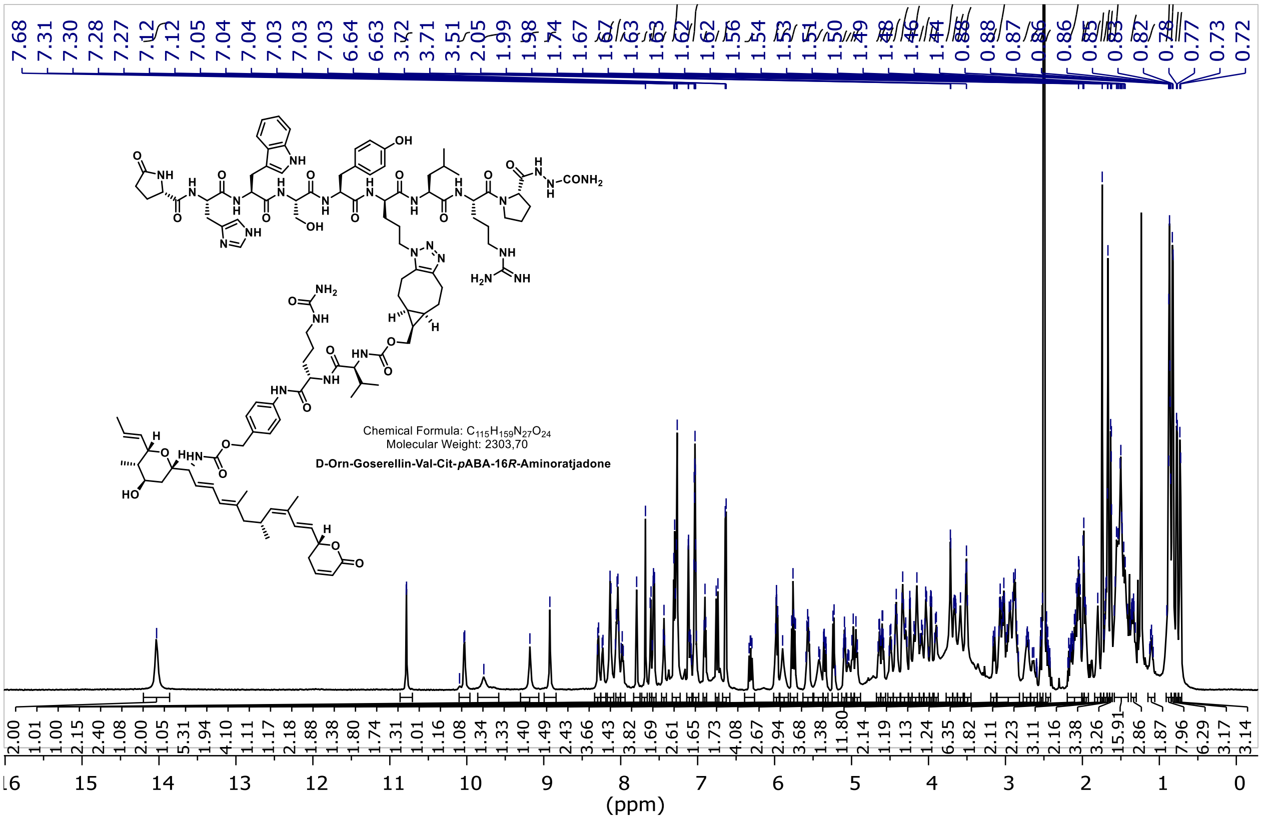

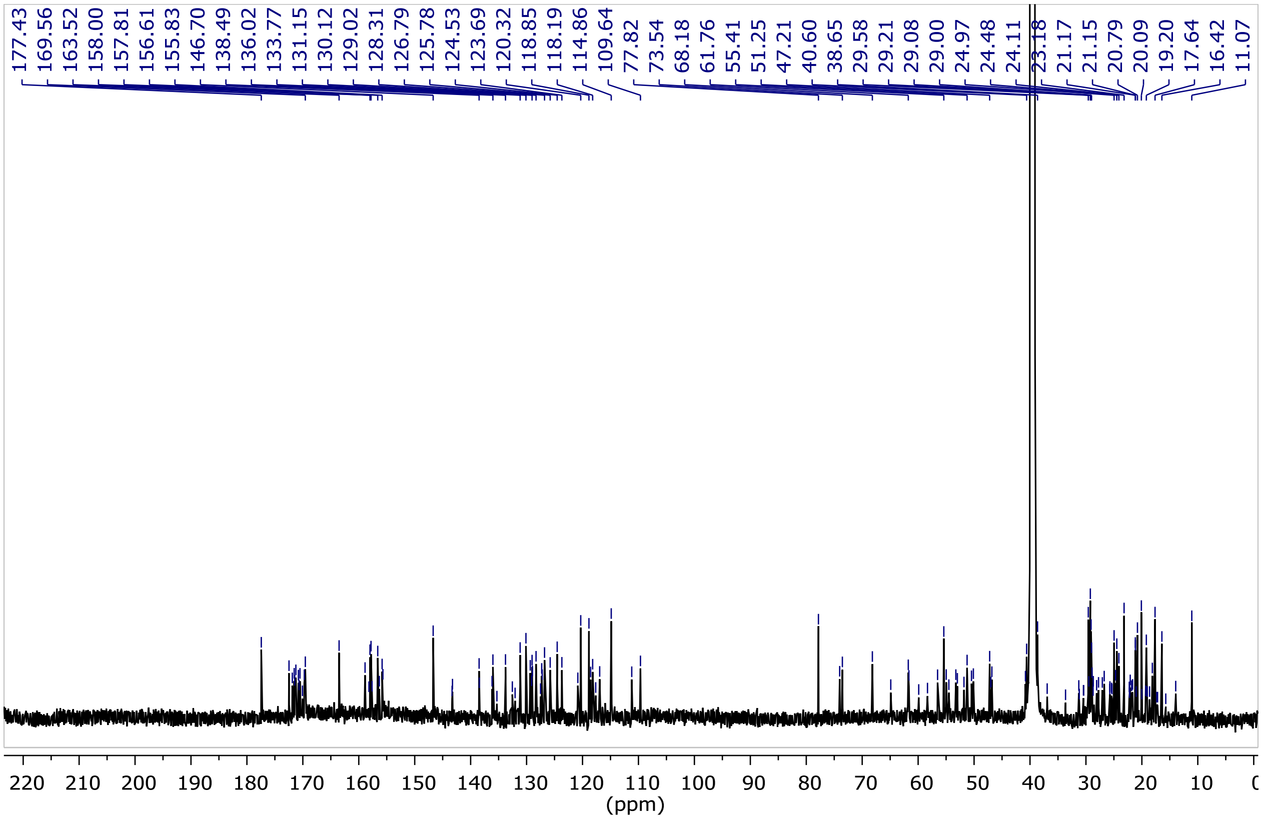

Supplement: Supplementary file 1 [file SC-010-C8SC05542D-s001.pdf]
